# Halogen bonding-induced 1,3-carbohydroxylation of allyl carboxylates *via* 1,2-cationic acyloxy migration (1,2-CAM)

**DOI:** 10.1039/d5sc08514d

**Published:** 2025-12-24

**Authors:** Sahil Sharma, Gaoyuan Zhao, Loay Bedda, Arman Khosravi, Djamaladdin G. Musaev, Ming-Yu Ngai

**Affiliations:** a James Tarpo Jr. and Margaret Tarpo Department of Chemistry, Purdue University West Lafayette Indiana 47907 USA mngai@purdue.edu; b Center for Scientific Computation, Department of Chemistry, Emory University Atlanta GA 30322 USA dmusaev@emory.edu

## Abstract

Halogen bonding has emerged as a powerful yet underexplored tool for modulating radical reactivity. Here we demonstrate that halogen-bonding interactions between alkyl iodides and water can lower the C–I bond dissociation energy, enabling visible-light-induced photolysis to generate alkyl radicals under mild conditions. Harnessing this activation mode, we achieved a previously unknown 1,3-carbohydroxylation of allyl carboxylates, wherein radical addition is coupled with 1,2-cationic acyloxy migration (CAM) to furnish β-acyloxy alcohols. The transformation exhibits broad structural tolerance, accommodating diverse esters, thioesters, amides, and perfluoroalkyl iodides, and is effective in the late-stage diversification of natural products and drug-derived scaffolds. Mechanistic studies, including isotopic labeling, radical trapping, UV-vis spectroscopy, and DFT calculations, reveal a pathway in which halogen bonding initiates radical alkene addition, followed by rearrangement and carbocation capture. These findings showcase halogen-bonding-assisted photochemistry as a viable platform for radical–cationic cascades, opening new opportunities for reaction development.

## Abstract 2

Halogen-bond-assisted visible-light activation of alkyl iodide C–I bonds by H_2_O initiates a radical–cationic cascade that converts allyl carboxylates into β-acyloxy alcohols *via* 1,2-cationic acyloxy migration, enabling formal 1,3-carbohydroxylation.

## Graphical abstract

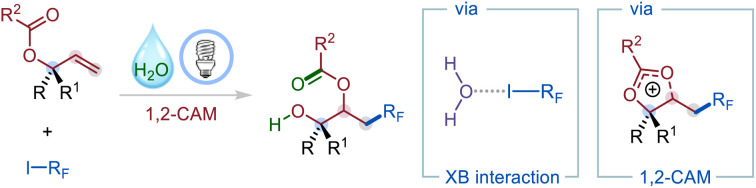

## Introduction

**Fig. 1:**
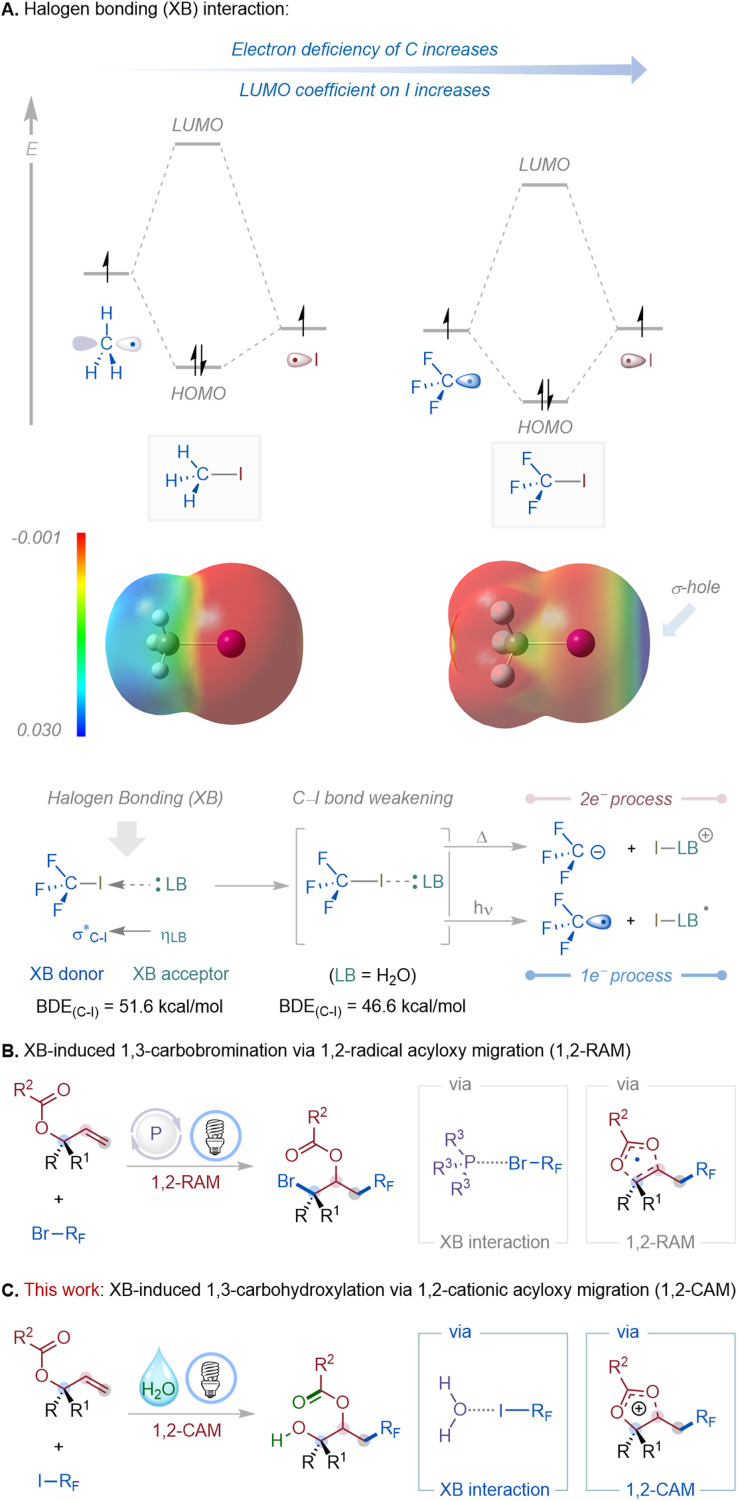
Halogen bonding (XB) in C–X bond activation and acyloxy migration-enabled alkene functionalization. (A) Electronic origin of halogen bonding in alkyl iodides and its effect on C–I bond weakening. (B) Previous work: visible-light-induced phosphine-catalyzed 1,3-carbobromination of allyl carboxylates *via* 1,2-radical acyloxy migration (1,2-RAM). (C) This work: visible-light-induced halogen-bonding-assisted formal 1,3-carbohydroxylation of allyl carboxylates *via* 1,2-cationic acyloxy migration (1,2-CAM).

Halogen bonding (XB), a directional noncovalent interaction between a Lewis base (LB) and an electrophilic region on a halogen atom (σ-hole), has emerged as a powerful activation mode in molecular design and synthesis.^1^ The σ-hole strength depends strongly on the substituents: electron-withdrawing groups, like fluorines, in CF_3_I lower the carbon orbital energy, shifting the LUMO coefficient onto iodine and creating a pronounced σ-hole (Fig. 1A).^2^ Consequently, CF_3_I forms stronger XB interactions than CH_3_I, whose σ-hole is negligible. In a typical XB interaction, the lone-pair of the Lewis base (XB acceptor) donates electron density into the antibonding σ*(C–X) orbital of the XB donor, lowering the bond dissociation energy (BDE) of the C–X bond and enabling selective activation under mild conditions.^1*b*^ Depending on the energy input, the weakened bond can undergo either a two-electron (thermal heterolysis) or a one-electron (photolysis) process, generating cationic or radical intermediates, respectively.^3^ This simple yet powerful interaction has emerged as a general activation strategy in organic synthesis, enabling molecular recognition, catalysis, and selective bond transformations.^3^ For example, Ritter and co-workers demonstrated condensed-phase XB adducts of CF_3_I and C_2_F_5_I for perfluoroalkylation under visible light,^4^ while Chen and colleagues showed that water itself can act as an XB acceptor to iodoperfluoro compounds, triggering radical generation.^5^ Yamaguchi and Itoh showed that photoirradiation of halogen-bonded complexes between haloarenes or haloalkanes and amines or phenols efficiently generates carbon radicals without metal catalysts or oxidants.^6^ Collectively, these studies highlight halogen bonding as a versatile platform for C–X bond activation and molecular functionalization.

Allyl carboxylates have continued to serve as privileged scaffolds in organic synthesis due to their diverse reactivity.^7^ They participate in transition-metal-catalyzed nucleophilic (*e.g.*, Tsuji–Trost and decarboxylative allylations)^7*a*–*f*,*j*,*n*,8^ and electrophilic substitutions,^7*g*,*h*,*k*,9^ and undergo anionic acyloxy migrations and rearrangements through two-electron pathways.^10^ More recently, radical-based strategies have enabled novel 1,2-functionalizations and rearrangements, expanding the synthetic utility of this substrate class beyond traditional sigmatropic and substitution manifolds.^11^ Despite these advances, formal 1,3-difunctionalization reactions, wherein two distinct groups add across the allyl unit in a 1,3-relationship accompanied by acyloxy migration, remain rare and mechanistically challenging. To address this limitation, our laboratory recently reported a visible-light-induced, phosphine-catalyzed 1,3-carbobromination of allyl carboxylates, which proceeded *via* a 1,2-radical acyloxy migration (RAM) mechanism (Fig. 1B).^12^ This transformation exploited the interaction between bisphosphine (dppm) catalysts and bromodifluoroacetates to promote photolytic C–Br bond cleavage under visible light irradiation.

During these investigations, we observed an unexpected divergent pathway: iododifluoroacetates furnished 1,3-carbohydroxylation products even in the absence of a phosphine catalyst. Mechanistic studies indicated that water forms a halogen-bonded complex with the iododifluoroacetate, promoting visible-light-induced C–I bond photolysis. The resulting perfluoroalkyl radical adds regioselectively to allyl carboxylates, followed by a 1,2-cationic acyloxy migration and hydration to afford β-acyloxy alcohols (Fig. 1C). This transformation is significant because it (i) establishes a radical–cationic cascade reaction platform with broad substrate scope, including late-stage modification of bioactive molecules, (ii) expands allyl carboxylate chemistry beyond classical two-electron pathways and radical 1,2-migrations, (iii) demonstrates that water serves as a simple yet powerful halogen-bond acceptor for C–I activation, (iv) enables site-selective synthesis of mono-protected 1,2-diol products, (v) represents the first example of XB-induced 1,3-carbohydroxylation of allyl carboxylates, and (vi) leverages perfluoroalkyl iodides, valuable precursors to fluorinated motifs of pharmaceutical importance,^13^ under mild, photocatalyst-free conditions.

## Results and discussion

Building on these mechanistic insights, we next optimized the reaction conditions to validate the proposed pathway and establish general parameters for this transformation. To this end, 2-methylbut-3-en-2-yl benzoate (1a) and ethyl iododifluoroacetate (2a) were selected as model substrates (Table 1). The optimal conditions were identified as the coupling of 1a with 2a under 100 W blue LED irradiation in 1,2-dichloroethane (0.10 M) at 90 °C for 24 h, in the presence of Na_2_CO_3_ (2.5 equiv.) and degassed H_2_O (10 equiv.), furnishing the desired 1,3-carbohydroxylated product 3a in 99% NMR yield (entry 1). The reaction completely shut down in the absence of Na_2_CO_3_ (entry 2), and substitution with other carbonate bases delivered diminished efficiencies (entries 3 and 4), underlining a pronounced counter-cation effect, likely by influencing ion pairing and solvation in DCE.^14^ A non-carbonate ionic base was less effective (entry 5). Concentration studies revealed that dilution to 0.05 M had little effect on yield, whereas increasing the concentration to 0.20 M diminished product formation (entries 6 and 7). Control experiments confirmed that light, heat, and an oxygen-free environment are essential for achieving high reaction efficiency (entries 8–10).

**Table 1:** Selected optimization experiments^a^

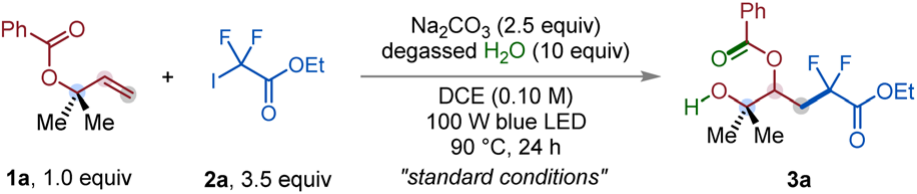		
Entry	Deviation from the standard conditions	NMR-yield (%)
1	None	99
2	Without Na_2_CO_3_	<2
3	K_2_CO_3_ instead of Na_2_CO_3_	60
4	NaHCO_3_ instead of Na_2_CO_3_	83
5	^*t*^BuOK instead of Na_2_CO_3_	<2
6	0.05 M instead of 0.10 M	99
7	0.20 M instead of 0.10 M	61
8	Without light	<2
9	Without heat	15
10	In air	<2

a See SI for experimental details. Reaction yields were determined by ^1^H-NMR using CH_2_Br_2_ as an internal standard.

With the optimized conditions in hand, we evaluated the substrate scope of the halogen-bonding-induced formal 1,3-carbohydroxylation of allyl carboxylates (Table 2). A broad array of acyclic and cyclic allyl carboxylates underwent efficient 1,3-difunctionalization, furnishing the corresponding β-acyloxy alcohols (3a–3k) in good yield (Table 2A). Symmetrical acyclic allyl carboxylates, such as 1a, furnished the desired product 3a in 76% yield. Importantly, the reaction was readily scalable to the 5 mmol level, affording 0.97 g of 3a in 63% yield. Cyclic systems ranging from five- to twelve-membered rings, including those incorporating a cyclic ether (3c) or an ethylene ketal (3d), were well tolerated, affording products 3b–3f in 55–75% yield. Furthermore, nonsymmetrical allyl carboxylates bearing dialkyl groups (3g), additional ester units (3h), halogens (3i, 3j), and a phthalimide motif (3k) also participated smoothly, highlighting the broad functional group compatibility of the transformation.

**Table 2:** Halogen bonding-induced 1,3-carbohydroxylation of allyl carboxylates *via* 1,2-CAM^a^

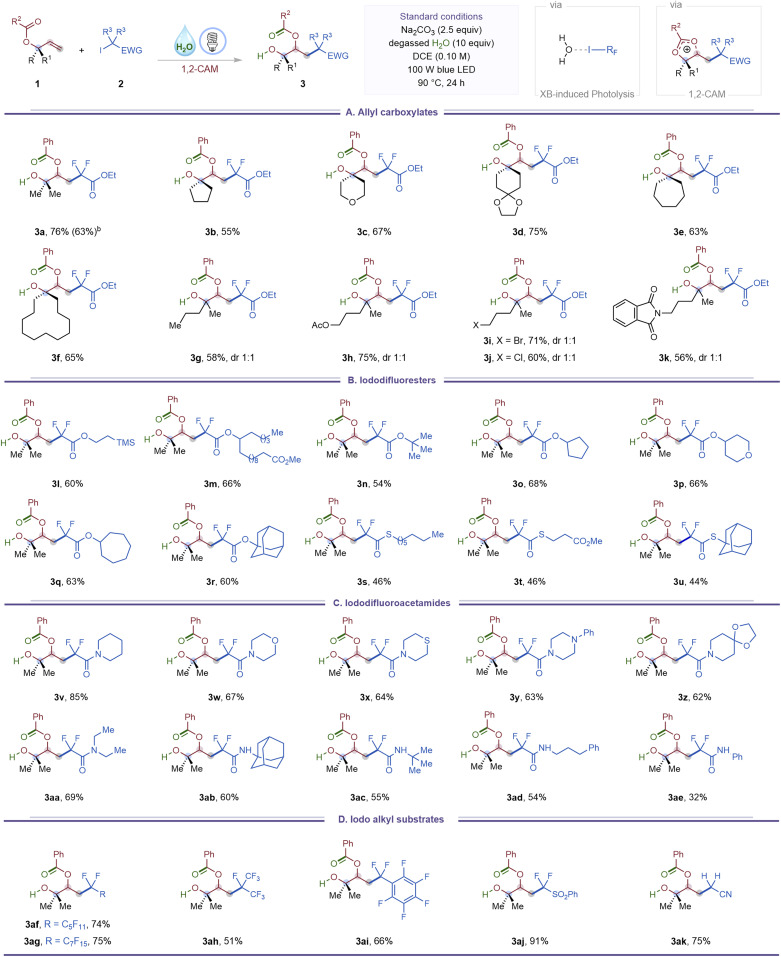

a See SI for experimental details. Standard reaction conditions: 1 (0.20 mmol, 1.0 equiv.), 2 (0.70 mmol, 3.5 equiv.), Na_2_CO_3_ (0.50 mmol, 2.5 equiv.) and degassed H_2_O (2.0 mmol, 10 equiv.), DCE (0.10 M), blue LED, 90 °C, 24 h.

b Parenthetical yields are from gram-scale experiments.

The reaction also proved effective with a variety of difluoroester partners (Table 2B). Acyclic esters spanning primary, secondary, and tertiary substitution were all competent, including those bearing a trimethylsilyl group (3l), long-chain substituents containing ester functionalities (3m), and a sterically demanding *tert*-butyl group (3n), affording the desired products in good yield. Secondary and tertiary cyclic esters within 5–7-membered ring systems, including the bulky adamantyl group (3r), furnished the corresponding β-acyloxy alcohols in 60–68% yield. Notably, thioesters bearing linear alkyl chains, additional ester substituents, or adamantyl were also effective (3s–3u), thereby extending the scope to sulfur-containing substrates.

Encouraged by these results, we next investigated iododifluoroacetamides, which provided the desired β-acyloxy alcohols (3v–3ae) in moderate to excellent yield (Table 2C). Both symmetrical and nonsymmetrical amides, encompassing cyclic and acyclic frameworks, were tolerated. Six-membered cyclic amides incorporating ether (3w), thioether (3x), *N*-phenyl (3y), or ethylene ketal (3z) functionalities afforded the desired products in 62–85% yield. Acyclic diethyl-substituted derivatives (3aa) as well as *N*-substituted systems, such as adamantyl (3ab), *tert*-butyl (3ac), phenylpropyl (3ad), and phenyl (3ae) were also compatible.

We further expanded the scope to iodoalkyl derivatives, which delivered β-acyloxy alcohols (3af–3ak) in good to excellent yield (Table 2D). Perfluoroalkyl iodides, including perfluorohexyl (3af), perfluorooctyl (3ag), perfluoroisopropyl (3ah), and perfluorobenzyl (3ai), were successfully engaged to give products in 51–75% yield. Notably, a fluoroalkyl iodide bearing a phenylsulfonyl group (3aj) exhibited excellent reactivity and afforded the desired product in 91% yield. Even iodoacetonitrile, without fluorine substituents, participated efficiently to afford the product in good yield (3ak).

We next investigated the effect of substituents on the migrating carboxylate group (Table 3A). Aryl esters bearing electron-donating substituents at the *para*- or *meta*-positions provided β-acyloxy alcohols (3al–3an) in 62–80% yield. Electron-withdrawing groups, including halogens and trifluoromethyl, were also tolerated across *para*, *meta*, and *ortho* positions (3ao–3as), with yields remaining largely unaffected by substitution pattern. A heteroaryl ester such as a thiophene derivative furnished the desired product (3at) in 57% yield, while an aliphatic ester migrated effectively to give product (3au) in 53% yield.

**Table 3:** Halogen bonding-induced 1,3-carbohydroxylation of allyl carboxylates *via* 1,2-CAM^a^

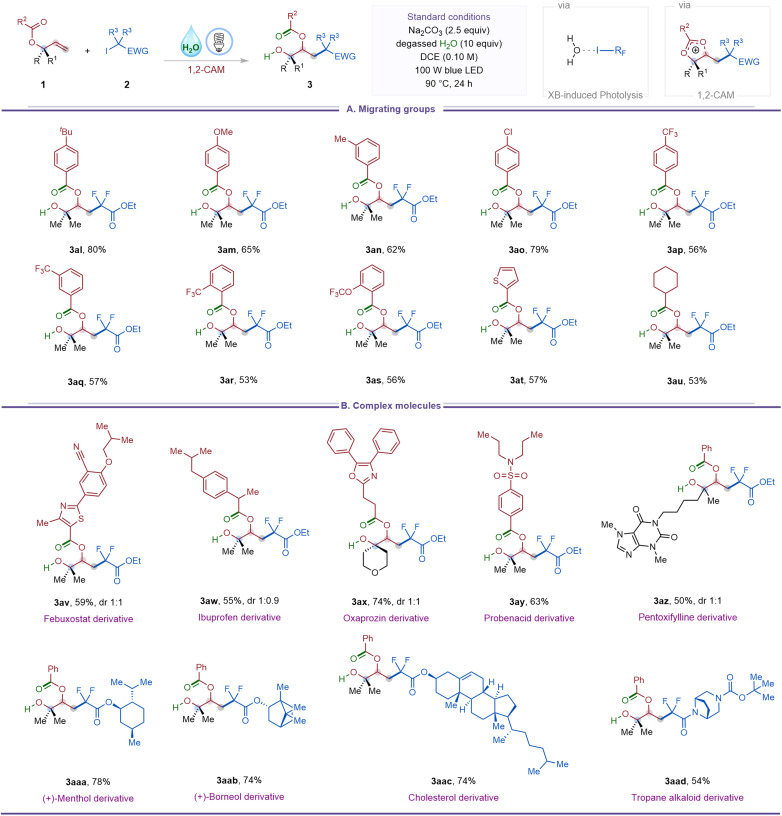

a See SI for experimental details. Standard reaction conditions: 1 (0.20 mmol, 1.0 equiv.), 2 (0.70 mmol, 3.5 equiv.), Na_2_CO_3_ (0.50 mmol, 2.5 equiv.) and degassed H_2_O (2.0 mmol, 10 equiv.), DCE (0.10 M), blue LED, 90 °C, 24 h.

Late-stage modification of bioactive, structurally complex molecules is a powerful strategy for the discovery of new medicinal agents.^15^ To demonstrate the applicability of the halogen-bonding-induced 1,3-carbohydroxylation of allyl carboxylates to late-stage diversification, a series of natural product- and drug-derived substrates were subjected to the standard conditions (Table 3B). Substrates derived from febuxostat (gout preventive), ibuprofen (analgesic/antipyretic), oxaprozin (anti-arthritic), and probenecid (gout treatment) smoothly underwent 1,3-carbohydroxylation, affording products 3av–3ay in 55–74% yield. An allyl benzoate derived from pentoxifylline (vasodilator) furnished product (3az) in 50% yield. Iododifluoroacetate derivatives of naturally occurring alcohols such as (+)-menthol, (+)-borneol, and cholesterol were also competent, affording products 3aaa–3aac in 74–78% yield. Notably, an amide incorporating the tropane alkaloid scaffold, a motif common in CNS-active agents, participated to provide the corresponding β-acyloxy alcohol (3aad) in 54% yield.

**Fig. 2:**
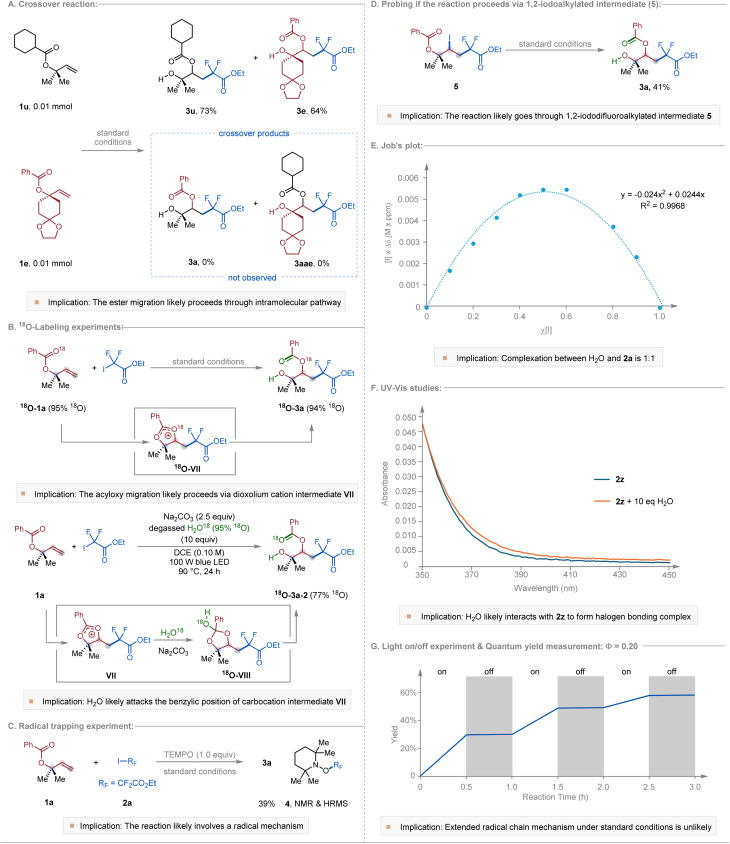
Mechanistic studies of halogen-bonding-induced 1,3-carbohydroxylation of allyl carboxylates. (A) Crossover experiment demonstrating intramolecular acyloxy migration. (B) ^18^O-Labeling experiments supporting formation of a dioxolium cation intermediate. (C) Radical-trapping experiment with TEMPO. (D) Reactivity of a preformed 1,2-iododifluoroalkylated intermediate. (E) Job's plot indicating 1 : 1 complexation between iododifluoro reagent and H_2_O. (F) UV-vis absorption changes consistent with halogen-bonding interactions. (G) Light on/off and quantum-yield experiments probing radical chain propagation.

To gain further insight into the reaction mechanism, we performed a series of experimental and computational studies (Fig. 2). Crossover experiments with substrates 1u and 1e yielded exclusively the corresponding non-crossover products 3u and 3e, indicating that the acyloxy migration proceeds *via* an intramolecular pathway (Fig. 2A). ^18^O-Labeling provided additional mechanistic evidence: reaction of ^18^O-labeled substrate ^18^O-1a (95% ^18^O incorporation) furnished product ^18^O-3a with 94% ^18^O retention, consistent with a five-membered [2,3]-acyloxy shift involving a dioxolium carbocation VII (Fig. 2B). Complementarily, conducting the standard reaction in the presence of degassed H_2_^18^O afforded ^18^O-3a-2 with 77% ^18^O incorporation in the benzoyl carbonyl group, implicating nucleophilic attack of H_2_O at the benzylic position of carbocation intermediate VII. The slight decrease in ^18^O incorporation is likely attributable to the hygroscopic nature of Na_2_CO_3_, which may introduce unlabeled water into the reaction medium.

Further evidence for a radical pathway was obtained by radical-trapping experiments. The addition of TEMPO markedly suppressed product formation, and the corresponding TEMPO-difluoroacetate adduct (4) was detected by ^19^F NMR and HRMS (Fig. 2C). In addition, a pre-synthesized 1,2-iododifluoroalkylated intermediate (5) could be converted to the desired 1,3-carbohydroxylated product 3a in 41% yield under the standard conditions (Fig. 2D), suggesting that the transformation may proceed *via* an initial 1,2-iododifluoroalkylation followed by intramolecular substitution reaction to give the dioxolium carbocation VII.

To further probe the presence of halogen bonding, Job's plot analysis revealed a 1 : 1 complexation between iododifluoroacetate 2a and H_2_O (Fig. 2E). Consistently, UV-vis absorption spectra of iododifluoroamide 2z in the presence of water exhibited an enhancement in absorption (Fig. 2F), indicative of halogen-bonding interactions between water and the iododifluoro reagent. We propose that this H_2_O⋯I–R_F_ complex serves as the key precursor to visible-light-induced generation of the difluoroacetate radical under the reaction conditions. Nevertheless, we cannot rule out the possibility that Na_2_CO_3_ may also engage in halogen bonding and contribute to halide activation. Furthermore, to assess whether the transformation proceeds *via* a radical chain process, we conducted light on/off experiments and quantum-yield measurements. As shown in Fig. 2G, the combined yield of the 1,2- and 1,3-substituted products did not increase upon cessation of irradiation, and the quantum yield measurement gave *Φ* = 0.20. These observations suggest that extended radical chain propagation pathway is unlikely.

**Fig. 3:**
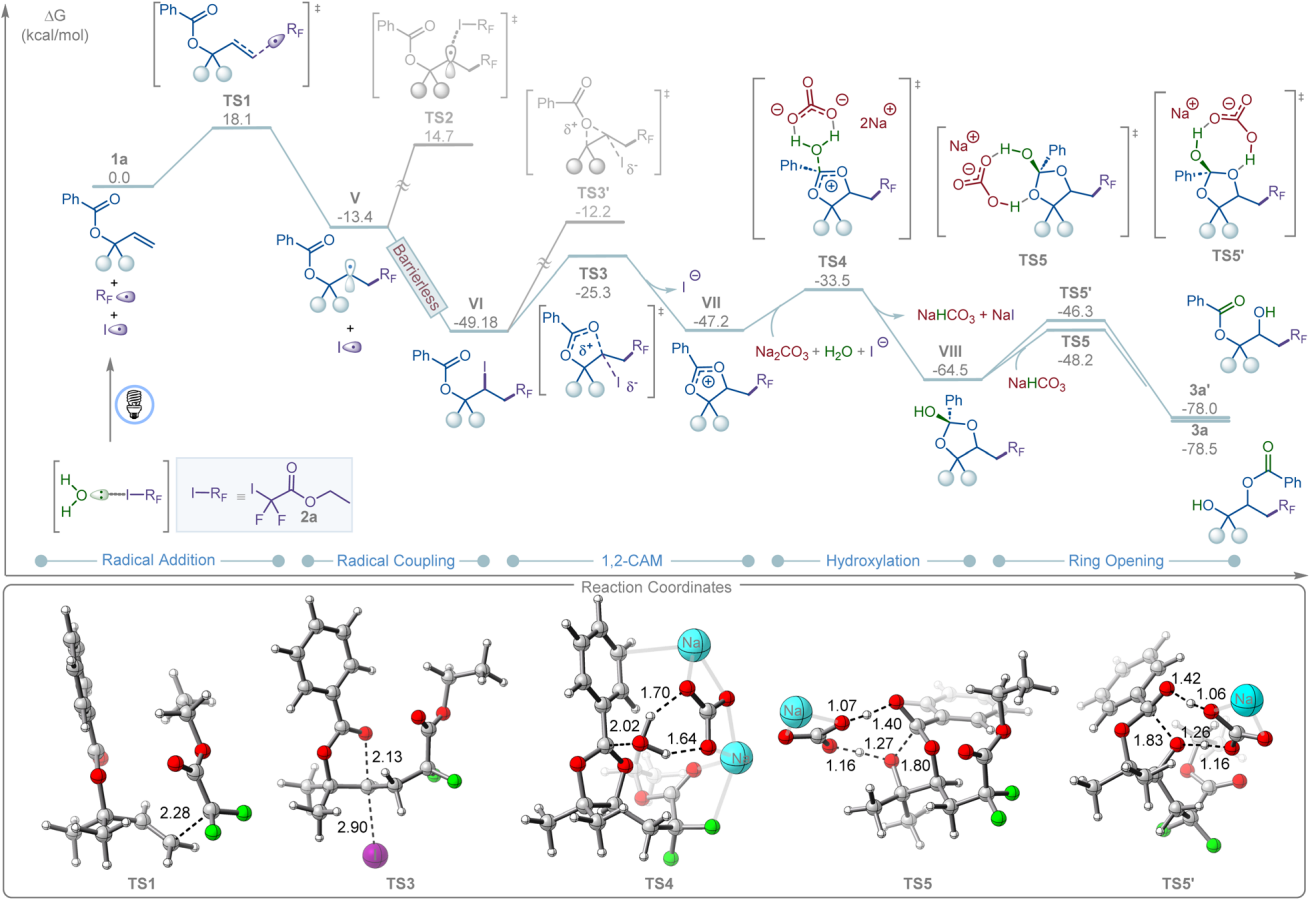
Energy profile of halogen bonding-induced 1,3-carbohydroxylation of allyl carboxylates 1a with iododifluoroester 2a*via* 1,2-CAM. DFT calculations were performed at the M06/6-311+G(d,p)-SDD/SMD(dichloroethane)//B3LYP-D3(BJ)/6-31 G(d)-SDD/SMD(dichloroethane) level of theory. The 3D representation was prepared by using CYLview.^16^

DFT calculations revealed that, upon water-assisted photolysis of 2a, the resulting R_F_ radical II can add to allyl carboxylate 1a to form the more stable secondary radical V (Fig. 3). This step is exergonic by Δ*G* = −13.4 kcal mol^−1^, with an associated transition state (TS1) barrier of Δ*G*^‡^ = 18.1 kcal mol^−1^. Recombination of V with the iodine radical is barrierless and highly exergonic (Δ*G* = −35.8 kcal mol^−1^). In contrast, a radical-chain pathway is disfavored due to the high transition state barrier for halogen atom transfer (TS2, Δ*G*^‡^ = 28.1 kcal mol^−1^), consistent with the light on/off experiments and quantum yield measurements (Fig. 2G). Intermediate VI subsequently undergoes a 1,2-cationic acyloxy migration (1,2-CAM), forming a dioxolium ion intermediate (VII) *via* a moderate barrier (TS3, Δ*G*^‡^ = 23.8 kcal mol^−1^). The alternative 3-membered TS3′ is significantly less favorable by 13.1 kcal mol^−1^. The resulting VII then undergoes carbonate-promoted hydrolysis through a six-membered transition-state complex (TS4, Δ*G*^‡^ = 13.7 kcal mol^−1^) to afford intermediate VIII, which is in good agreement with the ^18^O-labeling experiments (Fig. 2B). Finally, intermediate VIII undergoes NaHCO_3_-assisted ring opening and proton transfer to yield the desired and kinetically favored 1,3-product (3a) *via*TS5 (Δ*G*^‡^ = 16.3 kcal mol^−1^) in an overall thermodynamically favorable step (Δ*G* = −13.95 kcal mol^−1^). In contrast, the competing ring-opening pathway leading to the 1,2-product (3a′) is kinetically disfavored, with a higher barrier (TS5′, Δ*G*^‡^ = 18.2 kcal mol^−1^). This computational finding aligns with experimental results, where only the 1,3-product (3a) was observed.

## Conclusions

In summary, we have developed the first halogen-bonding-induced, visible-light-mediated formal 1,3-carbohydroxylation of allyl carboxylates *via* a 1,2-CAM pathway, furnishing β-acyloxy alcohols in good yields with broad functional-group tolerance. Mechanistic and computational studies reveal that water serves not only as the halogen-bond acceptor enabling photocatalyst-free C–I activation, but also as the source of the oxygen atom incorporated into the carbonyl group of the acyl fragment in the acyloxy group. This work establishes a radical–cationic cascade that bridges halogen-bonding activation with ionic rearrangement. We anticipate that the 1,2-CAM reactivity of allyl carboxylates and halogen-bonding-induced radical activation will inspire new strategies for tandem radical–cationic transformations in organic synthesis.

## Author contributions

S. S., G. Z. and A. K. performed the experiments, synthesized starting materials, developed the substrate scope, and conducted detailed mechanistic studies. L. B. and D. G. M. designed and performed the DFT calculations. G. Z., S. S. and M.-Y. N. conceived the idea and designed the research. S. S., L. B. and M.-Y. N. wrote the manuscript. All the authors commented on the final draft of the manuscript and contributed to the analysis and interpretation of the data.

## Conflicts of interest

There are no conflicts to declare.

## Supplementary Material

SC-OLF-D5SC08514D-s001

## Data Availability

Additional data are available from the corresponding author upon reasonable request. All experimental procedures, characterization data (^1^H, ^13^C, and ^19^F NMR spectra), and computational details supporting the findings of this study are provided in the supplementary information (SI). Supplementary information: Cartesian coordinates and energies for all computed structures. See DOI: https://doi.org/10.1039/d5sc08514d.
