# Supplementary material for: Halogen bonding-induced 1,3-carbohydroxylation of allyl carboxylates via 1,2-cationic acyloxy migration (1,2-CAM)
Source: Chem Sci. 2025 Dec 24;17(7):3618–26. doi: 10.1039/d5sc08514d (PMC12728568; doi:10.1039/d5sc08514d)
Supplement: SC-017-D5SC08514D-s001 [file SC-017-D5SC08514D-s001.pdf]

Supplementary Information (SI) for Chemical Science.
This journal is © The Royal Society of Chemistry 2025

Electronic Supplementary Information

Halogen Bonding-Induced 1,3-Carbohydroxylation of Allyl Carboxylates via 1,2-Cationic Acyloxy Migration (1,2-CAM)

Sahil Sharma,[a] Gaoyuan Zhao,[a] Loay Bedda,[a] Arman Khosravi,[a] Djamaladdin G. Musaev,[b] and Ming-Yu Ngai*[a]

mngai@purdue.edu
dmusaev@emory.edu

[a]James Tarpo Jr. and Margaret Tarpo Department of Chemistry, Purdue University, West Lafayette, Indiana, 47907, United States.
[b]Cherry L. Emerson Center for Scientific Computation and Department of Chemistry,
Emory University, Atlanta, GA 30322, United States.

Table of Contents

General Information 5

Reaction Setup 6

Experimental Data 7

General Procedure A (for the synthesis of iododifluoroacetates/amides): 7
General Procedure B (for the synthesis of iododifluoroacetamides): 7
General Procedure C (for the synthesis of iododifluoroacetamides): 7
Starting material 8
2-(trimethylsilyl)ethyl 2,2-difluoro-2-iodoacetate (2l) 9
methyl 12-(2,2-difluoro-2-iodoacetoxy)octadecanoate (2m) 9
Tert-butyl 2,2-difluoro-2-iodoacetate (2n) 9
cyclopentyl 2,2-difluoro-2-iodoacetate (2o) 9
tetrahydro-2*H*-pyran-4-yl 2,2-difluoro-2-iodoacetate (2p) 10
cycloheptyl 2,2-difluoro-2-iodoacetate (2q) 10
(3s,5s,7s)-adamantan-1-yl 2,2-difluoro-2-iodoacetate (2r) 10
S-octyl 2,2-difluoro-2-iodoethanethioate (2s) 11
methyl 3-((2,2-difluoro-2-iodoacetyl)thio)propanoate (2t) 11
S-((3s,5s,7s)-adamantan-1-yl) 2,2-difluoro-2-iodoethanethioate (2u) 11
2,2-difluoro-2-iodo-1-(piperidin-1-yl)ethan-1-one (2v) 11
2,2-difluoro-2-iodo-1-morpholinoethan-1-one (2w) 12
2,2-difluoro-2-iodo-1-thiomorpholinoethan-1-one (2x) 12
2,2-difluoro-2-iodo-1-(4-phenylpiperazin-1-yl)ethan-1-one (2y) 12
2,2-difluoro-2-iodo-1-(1,4-dioxa-8-azaspiro[4.5]decan-8-yl)ethan-1-one (2z) 12
N,*N*-diethyl-2,2-difluoro-2-iodoacetamide (2aa) 13
N-((3s,5s,7s)-adamantan-1-yl)-2,2-difluoro-2-iodoacetamide (2ab) 13
N-(tert-butyl)-2,2-difluoro-2-iodoacetamide (2ac) 13
2,2-difluoro-2-iodo-*N*-(3-phenylpropyl)acetamide (2ad) 14
2,2-difluoro-2-iodo-*N*-phenylacetamide (2ae) 14
((difluoroiodomethyl)sulfonyl)benzene (2aj) 14
(1R,2S,5R)-2-isopropyl-5-methylcyclohexyl 2,2-difluoro-2-iodoacetate (2aaa) 14
(2S,4R)-4,7,7-trimethylbicyclo[2.2.1]heptan-2-yl 2,2-difluoro-2-iodoacetate (2aab) 15
(3R,8R,9R,10S,13S,14R,17S)-10,13-dimethyl-17-((S)-6-methylheptan-2-yl)-2,3,4,7,8,9,10,11,12,13,14,15,16,17-tetradecahydro-1H-cyclopenta[a]phenanthren-3-yl 2,2-difluoro-2-iodoacetate (2aac) 15
tert-butyl 8-(2,2-difluoro-2-iodoacetyl)-3,8-diazabicyclo[3.2.1]octane-3-carboxylate (2aad) 15
6-ethoxy-5,5-difluoro-3-iodo-2-methyl-6-oxohexan-2-yl benzoate (5) 16
General Procedure D (for the 1,3-carbohydroxylation of allyl esters): 16
6-ethoxy-5,5-difluoro-2-hydroxy-2-methyl-6-oxohexan-3-yl benzoate (3a) 17

4-ethoxy-3,3-difluoro-1-(1-hydroxycyclopentyl)-4-oxobutyl benzoate (3b) 17
4-ethoxy-3,3-difluoro-1-(4-hydroxytetrahydro-2H-pyran-4-yl)-4-oxobutyl benzoate (3c) 17
4-ethoxy-3,3-difluoro-1-(8-hydroxy-1,4-dioxaspiro[4.5]decan-8-yl)-4-oxobutyl benzoate (3d) ... 18
4-ethoxy-3,3-difluoro-1-(1-hydroxycycloheptyl)-4-oxobutyl benzoate (3e) 18
4-ethoxy-3,3-difluoro-1-(1-hydroxycyclododecyl)-4-oxobutyl benzoate (3f) 19
1-(1-bromocycloheptyl)-4-ethoxy-3,3-difluoro-4-oxobutyl benzoate (3g) 19
8-acetoxy-1-ethoxy-2,2-difluoro-5-hydroxy-5-methyl-1-oxooctan-4-yl benzoate (3h) 20
8-bromo-1-ethoxy-2,2-difluoro-5-hydroxy-5-methyl-1-oxooctan-4-yl benzoate (3i) 20
8-chloro-1-ethoxy-2,2-difluoro-5-hydroxy-5-methyl-1-oxooctan-4-yl benzoate (3j) 21
8-(1,3-dioxoisoindolin-2-yl)-1-ethoxy-2,2-difluoro-5-hydroxy-5-methyl-1-oxooctan-4-yl benzoate (3k) 21
5,5-difluoro-2-hydroxy-2-methyl-6-oxo-6-(2-(trimethylsilyl)ethoxy)hexan-3-yl benzoate (3l) 22
5,5-difluoro-2-hydroxy-6-((18-methoxy-18-oxooctadecan-7-yl)oxy)-2-methyl-6-oxohexan-3-yl benzoate (3m) 22
6-(tert-butoxy)-5,5-difluoro-2-hydroxy-2-methyl-6-oxohexan-3-yl benzoate (3n) 23
6-(cyclopentyloxy)-5,5-difluoro-2-hydroxy-2-methyl-6-oxohexan-3-yl benzoate (3o) 23
5,5-difluoro-2-hydroxy-2-methyl-6-oxo-6-((tetrahydro-2H-pyran-4-yl)oxy)hexan-3-yl benzoate (3p) 23
6-(cycloheptyloxy)-5,5-difluoro-2-hydroxy-2-methyl-6-oxohexan-3-yl benzoate (3q) 24
6-(((3s,5s,7s)-adamantan-1-yl)oxy)-5,5-difluoro-2-hydroxy-2-methyl-6-oxohexan-3-yl benzoate (3r) 24
5,5-difluoro-2-hydroxy-2-methyl-6-(octylthio)-6-oxohexan-3-yl benzoate (3s) 25
5,5-difluoro-2-hydroxy-6-((3-methoxy-3-oxopropyl)thio)-2-methyl-6-oxohexan-3-yl benzoate (3t) 25
6-(((3s,5s,7s)-adamantan-1-yl)thio)-5,5-difluoro-2-hydroxy-2-methyl-6-oxohexan-3-yl benzoate (3u) 26
5,5-difluoro-2-hydroxy-2-methyl-6-oxo-6-(piperidin-1-yl)hexan-3-yl benzoate (3v) 26
5,5-difluoro-2-hydroxy-2-methyl-6-morpholino-6-oxohexan-3-yl benzoate (3w) 27
5,5-difluoro-2-hydroxy-2-methyl-6-oxo-6-thiomorpholinohexan-3-yl benzoate (3x) 27
5,5-difluoro-2-hydroxy-2-methyl-6-oxo-6-thiomorpholinohexan-3-yl benzoate (3y) 27
5,5-difluoro-2-hydroxy-2-methyl-6-oxo-6-(1,4-dioxa-8-azaspiro[4.5]decan-8-yl)hexan-3-yl benzoate (3z) 28
6-(diethylamino)-5,5-difluoro-2-hydroxy-2-methyl-6-oxohexan-3-yl benzoate (3aa) 28
6-(((3s,5s,7s)-adamantan-1-yl)amino)-5,5-difluoro-2-hydroxy-2-methyl-6-oxohexan-3-yl benzoate (3ab) 29
6-(tert-butylamino)-5,5-difluoro-2-hydroxy-2-methyl-6-oxohexan-3-yl benzoate (3ac) 29
5,5-difluoro-2-hydroxy-2-methyl-6-oxo-6-(phenethylamino)hexan-3-yl benzoate (3ad) 30
5,5-difluoro-2-hydroxy-2-methyl-6-oxo-6-(phenylamino)hexan-3-yl benzoate (3ae) 30
5,5,6,6,7,7,8,8,9,9,10,10,10-tridecafluoro-2-hydroxy-2-methyldecan-3-yl benzoate (3af) 30

5,5,6,6,7,7,8,8,9,9,10,10,11,11,12,12,12-heptadecafluoro-2-hydroxy-2-methyldodecan-3-yl benzoate (3ag) 31
5,6,6,6-tetrafluoro-2-hydroxy-2-methyl-5-(trifluoromethyl)hexan-3-yl benzoate (3ah) 31
1,1-difluoro-4-hydroxy-4-methyl-1-(perfluorophenyl)pentan-3-yl benzoate (3ai)........ 32
1,1-difluoro-4-hydroxy-4-methyl-1-(phenylsulfonyl)pentan-3-yl benzoate (3aj) 32
1-cyano-4-hydroxy-4-methylpentan-3-yl benzoate (3ak) 33
6-ethoxy-5,5-difluoro-2-hydroxy-2-methyl-6-oxohexan-3-yl 4-(tert-butyl)benzoate (3al) 33
6-ethoxy-5,5-difluoro-2-hydroxy-2-methyl-6-oxohexan-3-yl 4-methoxybenzoate (3am)........ 33
6-ethoxy-5,5-difluoro-2-hydroxy-2-methyl-6-oxohexan-3-yl 3-methylbenzoate (3an)........ 34
6-ethoxy-5,5-difluoro-2-hydroxy-2-methyl-6-oxohexan-3-yl 4-chlorobenzoate (3ao) 34
6-ethoxy-5,5-difluoro-2-hydroxy-2-methyl-6-oxohexan-3-yl 4-(trifluoromethyl)benzoate (3ap) . 35
6-ethoxy-5,5-difluoro-2-hydroxy-2-methyl-6-oxohexan-3-yl 3-(trifluoromethyl)benzoate (3aq) . 35
6-ethoxy-5,5-difluoro-2-hydroxy-2-methyl-6-oxohexan-3-yl 2-(trifluoromethyl)benzoate (3ar) .. 36
6-ethoxy-5,5-difluoro-2-hydroxy-2-methyl-6-oxohexan-3-yl 2-(trifluoromethoxy)benzoate (3as)36
6-ethoxy-5,5-difluoro-2-hydroxy-2-methyl-6-oxohexan-3-yl thiophene-2-carboxylate (3at) 36
6-ethoxy-5,5-difluoro-2-hydroxy-2-methyl-6-oxohexan-3-yl cyclohexanecarboxylate (3au) 37
6-ethoxy-5,5-difluoro-2-hydroxy-2-methyl-6-oxohexan-3-yl 2-(3-cyano-4-isobutoxyphenyl)-4-methylthiazole-5-carboxylate (3av) 37
ethyl 2,2-difluoro-5-hydroxy-4-((2-(4-isobutylphenyl)propanoyl)oxy)-5-methylhexanoate (3aw)38
ethyl 4-((3-(4,5-diphenyloxazol-2-yl)propanoyl)oxy)-2,2-difluoro-4-(4-hydroxytetrahydro-2H-pyran-4-yl)butanoate (3ax) 39
6-ethoxy-5,5-difluoro-2-hydroxy-2-methyl-6-oxohexan-3-yl 4-(N,N-dipropylsulfamoyl)benzoate (3ay) 39
9-(3,7-dimethyl-2,6-dioxo-2,3,6,7-tetrahydro-1H-purin-1-yl)-1-ethoxy-2,2-difluoro-5-hydroxy-5-methyl-1-oxononan-4-yl benzoate (3az) 40
5,5-difluoro-2-hydroxy-6-(((1R,2S,5R)-2-isopropyl-5-methylcyclohexyl)oxy)-2-methyl-6-oxohexan-3-yl benzoate (3aaa) 40
5,5-difluoro-2-hydroxy-2-methyl-6-oxo-6-(((2S,4R)-4,7,7-trimethylbicyclo[2.2.1]heptan-2-yl)oxy)hexan-3-yl benzoate (3aab) 41
6-(((3S,8S,9S,10R,13R,14S,17R)-10,13-dimethyl-17-((R)-6-methylheptan-2-yl)-2,3,4,7,8,9,10,11,12,13,14,15,16,17-tetradecahydro-1H-cyclopenta[a]phenanthren-3-yl)oxy)-5,5-difluoro-2-hydroxy-2-methyl-6-oxohexan-3-yl benzoate (3aac) 41
tert-butyl 8-(4-(benzoyloxy)-2,2-difluoro-5-hydroxy-5-methylhexanoyl)-3,8-diazabicyclo[3.2.1]octane-3-carboxylate (3aad) 42
Gram-scale Reaction 42

Mechanistic Studies 44

Radical Trapping Experiment 44
1,2-Iododifluoroalkylation intermediate as the substrate 45
Cross-over experiment 45

Light On/Off experiment 46
Quantum Yield Experiment 47
Job's plot........ 50
UV-Vis absorption spectrometry........ 51
^{18}O-Labeling experiment........ 52
Effect of Water Equivalents on the Yield of the 1,3-Carbohydroxylation Reaction 55
Effect of reaction temperature on the Yield of the 1,3-Carbohydroxylation Reaction........ 56
DFT Calculations 57
Computational Details 57
Computational Investigation of Reaction Mechanism........ 57
Spectroscopic Data 63
Cartesian Coordinates........ 226
References........ 249

General Information

All air- and moisture-insensitive reactions were carried out under an ambient atmosphere, magnetically stirred, and monitored by thin-layer chromatography (TLC) using Agela Technologies TLC plates pre-coated with 250 μm thickness silica gel 60 F254 plates and visualized by fluorescence quenching under UV light. Flash column chromatography was performed on SiliaFlash® Silica Gel 40-63μm 60 Å particle size using a forced flow of eluent at 0.3–0.5 bar pressure.[1] Preparative TLC was performed on Uniplate® UV254 (20 x 20 cm) with 1000 μm thickness and visualized fluorescence quenching under UV light.

All air and moisture-sensitive manipulations were performed using oven-dried glassware, including standard Schlenk and glovebox techniques under an atmosphere of nitrogen. All reaction vials were capped using green caps with F-217 PTFE liners. Isopropyl acetate was distilled from calcium chloride $CaCl_2$. Diethyl ether and THF were distilled from deep purple sodium benzophenone ketyl. Acetonitrile was dried ver CaH_2 and distilled. Isopropyl acetate and acetonitrile were degassed *via* three freeze-pump-thaw cycles. All other chemicals were used as received.

All deuterated solvents were purchased from Cambridge Isotope Laboratories. NMR spectra were recorded on either a Bruker Ascend 700 spectrometer operating at 700 MHz for ^{1}H acquisitions and 175 MHz for ^{13}C acquisitions, a Bruker 500 Advance spectrometer operating at 500 MHz for ^{1}H acquisitions and 125 MHz for ^{13}C acquisitions. A Bruker 400 Nanobay spectrometer was operating at 400 MHz, 100 MHz, and 376 MHz for ^{1}H, ^{13}C, and ^{19}F acquisitions, respectively. Chemical shifts were referenced to the residual proton solvent peaks (^{1}H: $CDCl_3$, δ7.26; CD_3CN, δ1.94) and ^{13}C solvent signals ($CDCl_3$, δ 77.16; CD_3CN, δ118.26).[2] Signals are listed in ppm, and multiplicity identified as s = singlet, br = broad, d = doublet, t = triplet, q = quartet, m = multiplet; coupling constants in Hz; integration.

UV-Vis Absorptions were measured on a Cary 100 UV-Vis spectrophotometer from Agilent Technologies. Emission intensities were recorded using a Perkin Elmer LS50B Luminescence spectrometer. High-resolution mass spectra were performed at Mass Spectrometry Services at Stony Brook University and were obtained using an Agilent LC-UV-TOF mass spectrometer. Concentration under reduced pressure was performed by rotary evaporation at 25–30 °C at the appropriate pressure. Purified compounds were further dried under a high vacuum (0.01–0.05 Torr). Yields refer to purified and spectroscopically pure compounds.

The blue light-emitting diodes used for the quantum yield measurements: 100 W Blue LEDs (LEDs, 100 W Royal Blue 456 nm, chip size = 45.0 x 45.0 mm) and the heat sink (diameter: 90.0 mm) were purchased from Babaoshop on eBay (https://www.ebay.com/usr/babaoshop).

Abbreviations: DCM = dichloromethane; THF = tetrahydrofuran; DIAD = Diisopropyl azodicarboxylate; DMAP = 4-dimethylaminopyridine; DCC = *N,N'*-Dicyclohexylcarbodiimide; EDCI·HCl = *N*-(3-Dimethylaminopropyl)-*N'*-ethylcarbodiimide hydrochloride; DIPEA = *N,N*-Diisopropylethylamine.

Reaction Setup

LED Light: 100 W Blue LED flood lamps from Babaoshop on eBay.

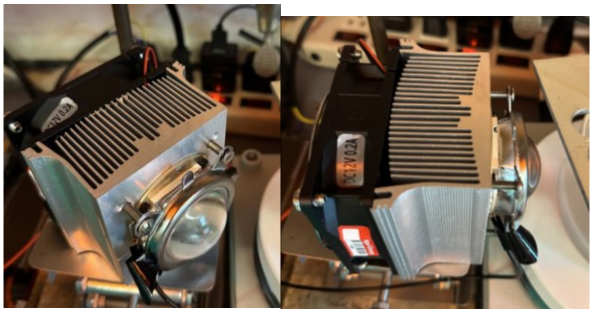

LED Light

The reaction set up: An oil bath was preheated to 90 °C, the 20 mL reaction vial was capped and sealed with grease, and black tape, and then the vial was placed into the oil bath. A 100 W Blue LED flood lamp was placed perpendicular to the reaction vial (shown in the picture below). The distance between the blue LED lamp and the vial was 3.00 cm.

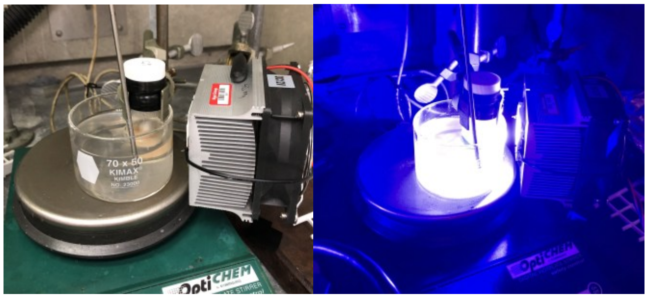

Reaction set up

Experimental Data

General Procedure A (for the synthesis of iododifluoroacetates/amides):

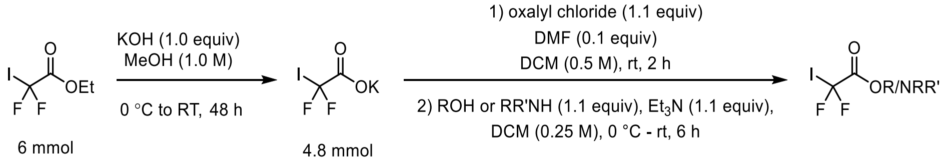

The iododifluoroacetates/amides were synthesized according to the literature procedure.[3] Potassium hydroxide (0.34 g, 6 mmol, 1.0 equiv) was dissolved in MeOH (6 mL) at 0 °C, then ethyl 2,2-difluoro-2-iodoacetate (1.50 g, 6 mmol, 1.0 equiv) was added. After stirring at 0 °C for 30 min, the mixture was warmed to room temperature and stirred for 48 h. The solvent was removed in vacuo to provide potassium 2,2-difluoro-2-iodoacetate (1.24 g, 80%) as a yellow solid. For the next step, potassium 2,2-difluoro-2-iodoacetate (1.24 g, 4.8 mmol) was dissolved in DCM (9.6 mL, 0.5 M), and was slowly added oxalyl chloride (0.44 mL, 5.28 mmol, 1.1 equiv) and DMF (35.0 µL, 0.1 equiv) at room temperature. After stirring at rt for 2 h, the reaction mixture was cooled to 0 °C, and then a mixture of ROH/RR'NH (5.28 mmol, 1.1 equiv) and Et_3N (0.73 mL, 5.28 mmol, 1.1 equiv) dissolved in DCM (9.6 mL) was added dropwise. The reaction mixture stirred at room temperature for 6 h, then quenched with saturated $NaHCO_3$ solution (20 mL) and extracted with DCM (3×30 mL). The organic layer was collected, washed with brine, dried with anhydrous $MgSO_4$ and filtered. The filtrate was concentrated *in vacuo*. The residue was purified by flash column chromatography on silica gel, eluting with Hexanes: EtOAc [10:1 (v/v)] to afford the title compound.

General Procedure B (for the synthesis of iododifluoroacetamides):

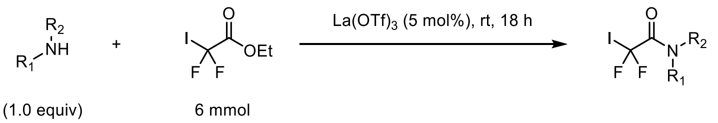

The iododifluoroacetamides were synthesized according to the literature procedure.[3b] To ethyl 2,2-difluoro-2-iodoacetate (1.50 g, 6 mmol, 1.0 equiv) was added amine (6 mmol, 1.0 equiv). The neat reaction mixture was stirred at room temperature for 18 h, then quenched with saturated water (20 mL) and extracted with diethyl ether (3×30 mL). The organic layer was collected, washed with brine, dried with anhydrous $MgSO_4$ and filtered. The filtrate was concentrated *in vacuo*. The residue was purified by flash column chromatography on silica gel, eluting with Hexanes: EtOAc to afford the title compound.

General Procedure C (for the synthesis of iododifluoroacetamides):

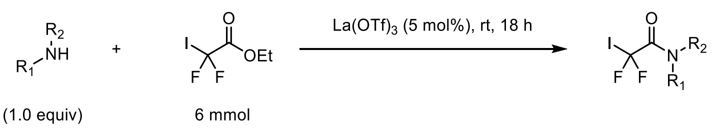

The iododifluoroacetamides were synthesized according to the literature procedure.[3b] To a mixture of ethyl 2,2-difluoro-2-iodoacetate (1.50 g, 6 mmol, 1.0 equiv) and amine (6 mmol, 1.0 equiv) added $La(OTf)_3$

(175.8 mg, 0.30 mmol, 5 mol%). The reaction mixture was stirred at room temperature for 18 h, then quenched with saturated water (20 mL) and extracted with diethyl ether (3×30 mL). The organic layer was collected, washed with brine, dried with anhydrous $MgSO_4$ and filtered. The filtrate was concentrated *in vacuo*. The residue was purified by flash column chromatography on silica gel, eluting with Hexanes: EtOAc to afford the title compound.

Starting material

All of the following starting material were prepared following our previous reports[4,5,6] and NMR data matched the reported data.

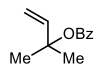
1a

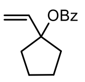
1b

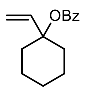
1c

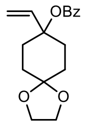
1d

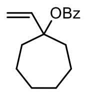
1e

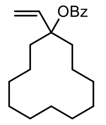
1f

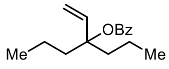
1g

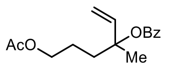
1h

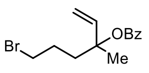
1i

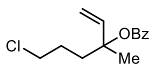
1j

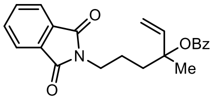
1k

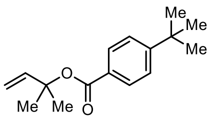
1al

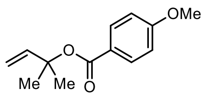
1am

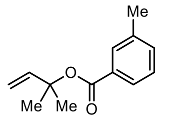
1an

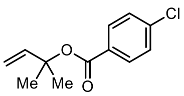
1ao

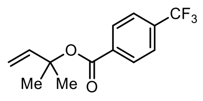
1ap

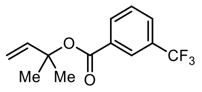
1aq

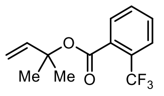
1ar

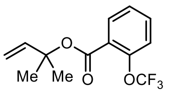
1as

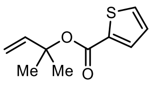
1at

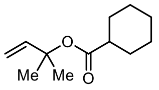
1au

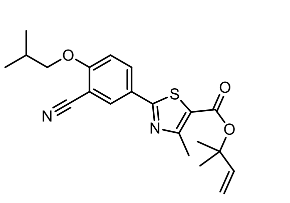
1av

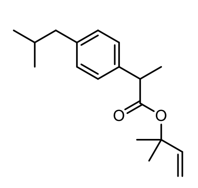
1aw

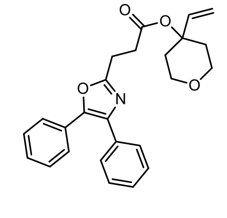
1ax

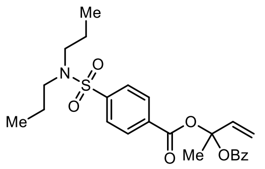
1ay

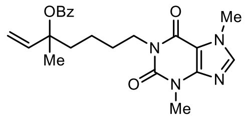
1az

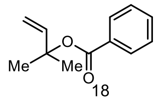
^{18}O-1a

2-(trimethylsilyl)ethyl 2,2-difluoro-2-iodoacetate (2l)

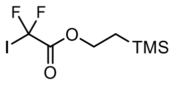

2l

Prepared according to General Procedure A, the title compound was obtained as a faint yellow oil (1.00 g, 3.12 mmol, 52% yield). **R_f** = 0.65 [Hexanes: EtOAc 10:1 (v/v)]. **^{1}H NMR** (500 MHz, $CDCl_3$) δ 4.48 – 4.38 (m, 2H), 1.17 – 1.08 (m, 2H), 0.08 (s, 9H). **^{13}C NMR** (126 MHz, $CDCl_3$) δ 160.62, 87.11 (t, J = 322.1 Hz), 67.35, 17.18, -1.40. **^{19}F NMR** (470 MHz, $CDCl_3$) δ -57.16.

methyl 12-(2,2-difluoro-2-iodoacetoxy)octadecanoate (2m)

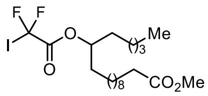

2m

Prepared according to General Procedure A, the title compound was obtained as a faint yellow oil (0.93 g, 1.8 mmol, 30% yield). **R_f** = 0.56 [Hexanes: EtOAc 10:1 (v/v)]. **^{1}H NMR** (500 MHz, $CDCl_3$) δ 5.01 (ddd, J = 12.7, 6.4, 3.8 Hz, 1H), 3.69 – 3.60 (m, 3H), 2.28 (q, J = 7.1 Hz, 2H), 1.70 – 1.60 (m, 4H), 1.42 – 1.20 (m, 24H), 0.86 (tt, J = 12.3, 5.9 Hz, 3H). **^{13}C NMR** (126 MHz, $CDCl_3$) δ 174.44, 160.29, 88.32 (d, J = 322.1 Hz), 79.94, 51.56, 34.24, 33.85, 32.07, 31.77, 29.71 (d, J = 30.0 Hz), 29.45, 29.36, 29.27, 29.15, 25.22 – 24.95 (m), 22.83, 22.67, 14.21 (d, J = 10.4 Hz). **^{19}F NMR** (470 MHz, $CDCl_3$) δ -57.01.

Tert-butyl 2,2-difluoro-2-iodoacetate (2n)

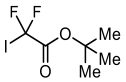

2n

Prepared according to General Procedure A, the title compound was obtained as a faint yellow oil (0.70 g, 2.52 mmol, 42% yield). **R_f** = 0.48 [Hexanes: EtOAc 10:1 (v/v)]. **^{1}H NMR** (500 MHz, $CDCl_3$) δ 1.55 (s, 9H). **^{13}C NMR** (126 MHz, $CDCl_3$) δ 159.19 (t, J = 27.3 Hz), 90.69, 89.60 – 82.83 (m), 27.53. **^{19}F NMR** (470 MHz, $CDCl_3$) δ -57.19.

cyclopentyl 2,2-difluoro-2-iodoacetate (2o)

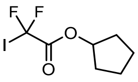

2o

Prepared according to General Procedure A, the title compound was obtained as a yellow oil (0.96 g, 3.3 mmol, 55% yield). **R*f*** = 0.50 [Hexanes: EtOAc 10:1 (v/v)]. **^{1}H NMR** (500 MHz, $CDCl_3$) δ 4.97 (tt, J = 8.8, 3.8 Hz, 1H), 1.96 – 1.85 (m, 2H), 1.79 (dddd, J = 13.5, 7.0, 3.4, 1.7 Hz, 2H), 1.61 (ddd, J = 13.0, 8.3, 3.7 Hz, 2H), 1.43 (tt, J = 7.1, 4.9 Hz, 2H). **^{13}C NMR** (126 MHz, $CDCl_3$) δ 159.97, 87.35 (t, J = 322.3 Hz), 77.52, 30.84, 25.23, 23.33. **^{19}F NMR** (470 MHz, $CDCl_3$) δ -57.30.

tetrahydro-2*H*-pyran-4-yl 2,2-difluoro-2-iodoacetate (2p)

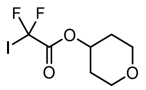

2p

Prepared according to General Procedure A, the title compound was obtained as a yellow solid (1.12 g, 3.66 mmol, 61% yield). **R*f*** = 0.50 [Hexanes: EtOAc 10:1 (v/v)]. **^{1}H NMR** (500 MHz, $CDCl_3$) δ 5.15 (tt, J = 8.0, 4.0 Hz, 1H), 3.94 (ddd, J = 11.9, 6.2, 3.9 Hz, 2H), 3.61 (ddd, J = 11.7, 8.1, 3.4 Hz, 2H), 2.07 – 1.96 (m, 2H), 1.87 – 1.76 (m, 2H). **^{13}C NMR** (126 MHz, $CDCl_3$) δ 159.80 (t, J = 28.5 Hz), 86.80 (t, J = 322.0 Hz), 73.78, 64.82, 30.96. **^{19}F NMR** (470 MHz, $CDCl_3$) δ -57.66. **HRMS** (ESI+) *m/z* calcd for $C_7H_{10}F_2IO_3$ [(M + H)$^+$], 306.9642, found, 306.9648.

cycloheptyl 2,2-difluoro-2-iodoacetate (2q)

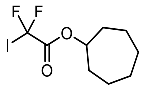

2q

Prepared according to General Procedure A, the title compound was obtained as a colorless oil (0.89 g, 2.82 mmol, 47% yield). **R*f*** = 0.6 [Hexanes: EtOAc 10:1 (v/v)]. **^{1}H NMR** (500 MHz, $CDCl_3$) δ 5.12 (tt, J = 8.1, 4.5 Hz, 1H), 2.03 – 1.95 (m, 2H), 1.81 (dddd, J = 14.2, 9.4, 8.0, 2.9 Hz, 2H), 1.76 – 1.68 (m, 2H), 1.66 – 1.55 (m, 4H), 1.53 – 1.44 (m, 2H). **^{13}C NMR** (126 MHz, $CDCl_3$) δ 159.86 (t, J = 27.8 Hz), 87.55 (t, J = 322.4 Hz), 80.25, 33.16, 28.25, 22.66. **^{19}F NMR** (470 MHz, $CDCl_3$) δ -57.29.

(3s,5s,7s)-adamantan-1-yl 2,2-difluoro-2-iodoacetate (2r)

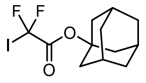

2r

Prepared according to General Procedure A, the title compound was obtained as a yellow oil (0.89 g, 2.52 mmol, 42% yield). **R*f*** = 0.55 [Hexanes: EtOAc 10:1 (v/v)]. **^{1}H NMR** (500 MHz, $CDCl_3$) δ 2.27 – 2.16 (m, 9H), 1.75 – 1.62 (m, 6H). **^{13}C NMR** (126 MHz, $CDCl_3$) δ 158.79, 92.07 – 83.75 (m), 41.95, 41.07, 40.78, 36.16, 35.99, 31.13. **^{19}F NMR** (470 MHz, $CDCl_3$) δ -57.08.

S-octyl 2,2-difluoro-2-iodoethanethioate (2s)

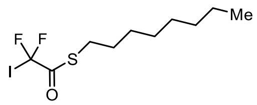

2s

Prepared according to General Procedure A, the title compound was obtained as a yellow oil (1.26 g, 3.6 mmol, 60% yield). **R$_f$** = 0.68 [Hexanes: EtOAc 10:1 (v/v)]. **^{1}H NMR** (500 MHz, $CDCl_3$) δ 3.03 (t, J = 7.4 Hz, 2H), 1.72 – 1.56 (m, 2H), 1.40 (qd, J = 7.8, 4.0 Hz, 2H), 1.33 – 1.23 (m, 8H), 0.92 – 0.85 (m, 3H). **^{13}C NMR** (126 MHz, $CDCl_3$) ^{13}C NMR (126 MHz, $CDCl_3$) δ 188.71 (t, *J* = 27.8 Hz), 93.90 (t, *J* = 326.7 Hz), 29.65, 29.14 (d, *J* = 13.7 Hz), 28.80 (d, *J* = 4.3 Hz), 22.75, 14.21. **^{19}F NMR** (470 MHz, $CDCl_3$) δ -54.85.

methyl 3-((2,2-difluoro-2-iodoacetyl)thio)propanoate (2t)

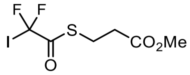

2t

Prepared according to General Procedure A, the title compound was obtained as a faint yellow oil (1.03 g, 3.18 mmol, 53% yield). **R$_f$** = 0.52 [Hexanes: EtOAc 10:1 (v/v)]. **^{1}H NMR** (500 MHz, $CDCl_3$) δ 3.73 (s, 3H), 3.28 (t, J = 6.9 Hz, 2H), 2.71 (t, J = 6.9 Hz, 2H). **^{13}C NMR** (126 MHz, $CDCl_3$) δ 188.55, 171.54, 93.44 (t, J = 326.4 Hz), 52.28, 33.35, 24.58. **^{19}F NMR** (470 MHz, $CDCl_3$) δ -55.30.

S-((3s,5s,7s)-adamantan-1-yl) 2,2-difluoro-2-iodoethanethioate (2u)

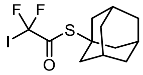

2u

Prepared according to General Procedure A, the title compound was obtained as a yellow oil (0.31 g, 0.84 mmol, 14% yield). **R$_f$** = 0.56 [Hexanes: EtOAc 10:1 (v/v)]. **^{1}H NMR** (500 MHz, $CDCl_3$) δ 2.20 (d, J = 2.9 Hz, 2H), 2.08 (ddd, J = 23.0, 6.2, 3.2 Hz, 3H), 1.82 (d, J = 2.9 Hz, 4H), 1.79 – 1.72 (m, 2H), 1.68 – 1.61 (m, 4H).**^{13}C NMR** (126 MHz, $CDCl_3$) δ 187.52, 94.99, 53.12, 47.85, 43.19, 41.59, 36.24 (d, J = 8.7 Hz), 30.21, 30.02. **^{19}F NMR** (470 MHz, $CDCl_3$) δ -54.68.

2,2-difluoro-2-iodo-1-(piperidin-1-yl)ethan-1-one (2v)

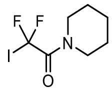

2v

Prepared according to General Procedure B, the title compound was obtained as a faint yellow oil (1.11 g, 3.84 mmol, 64% yield). **R$_f$** = 0.6 [Hexanes: EtOAc 10:1 (v/v)]. **^{1}H NMR** (500 MHz, $CDCl_3$) δ 3.62 – 3.52

(m, 4H), 1.68 (dt, J = 6.4, 3.6 Hz, 6H). **^{13}C NMR** (126 MHz, $CDCl_3$) δ 158.63 (t, J = 23.6 Hz), 90.26 (t, J = 321.7 Hz), 48.24 (t, J = 4.5 Hz), 45.18, 25.62 (d, J = 6.1 Hz), 24.26. **^{19}F NMR** (470 MHz, $CDCl_3$) δ -49.98. **HRMS** (ESI+) *m/z* calcd for $C_7H_{11}F_2INO$ [(M + H)$^+$], 289.9853, found, 289.9851.

2,2-difluoro-2-iodo-1-morpholinoethan-1-one (2w)

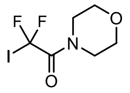

2w

Prepared according to General Procedure B, the title compound was obtained as a yellow oil (1.15 g, 3.96 mmol, 66% yield). **R_f** = 0.54 [Hexanes: EtOAc 10:1 (v/v)]. **^{1}H NMR** (500 MHz, $CDCl_3$) δ 3.74 (q, J = 3.7 Hz, 5H), 3.69 (dd, J = 5.4, 3.6 Hz, 5H). **^{13}C NMR** (126 MHz, $CDCl_3$) δ 158.95 (d, J = 24.0 Hz), 89.70 (t, J = 321.9 Hz), 66.68, 65.88, 47.81, 44.02. **^{19}F NMR** (470 MHz, $CDCl_3$) δ -50.87.

2,2-difluoro-2-iodo-1-thiomorpholinoethan-1-one (2x)

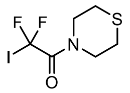

2x

Prepared according to General Procedure C, the title compound was obtained as a white solid (0.81 g, 2.64 mmol, 44% yield). **R_f** = 0.50 [Hexanes: EtOAc 10:1 (v/v)]. **^{1}H NMR** (500 MHz, $CDCl_3$) δ 3.93 (dt, J = 7.5, 2.7 Hz, 4H), 2.77 – 2.67 (m, 4H). **^{13}C NMR** (126 MHz, $CDCl_3$) δ 158.87, 89.75, 49.92, 46.75, 27.42 (d, J = 21.6 Hz). **^{19}F NMR** (470 MHz, $CDCl_3$) δ -50.38. **HRMS** (ESI+) *m/z* calcd for $C_6H_9F_2INOSH$ [(M + H)$^+$], 307.9417, found, 307.9417.

2,2-difluoro-2-iodo-1-(4-phenylpiperazin-1-yl)ethan-1-one (2y)

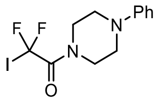

2y

Prepared according to General Procedure C, the title compound was obtained as a yellow oil (0.66 g, 1.8 mmol, 30% yield). **R_f** = 0.42 [Hexanes: EtOAc 10:1 (v/v)]. **^{1}H NMR** (500 MHz, $CDCl_3$) δ 7.34 – 7.26 (m, 2H), 6.98 – 6.91 (m, 3H), 3.89 – 3.82 (m, 4H), 3.26 (dt, J = 22.1, 5.2 Hz, 4H). **^{13}C NMR** (126 MHz, $CDCl_3$) δ 158.74 (d, J = 24.0 Hz), 150.68, 129.48, 121.14, 116.94, 97.61 – 84.37 (m), 49.23 (d, J = 38.0 Hz), 47.09, 43.81. **^{19}F NMR** (470 MHz, $CDCl_3$) δ -50.50. **HRMS** (ESI+) *m/z* calcd for $C_{12}H_{14}F_2IN_2O$ [(M + H)$^+$], 367.0118, found, 367.0014.

2,2-difluoro-2-iodo-1-(1,4-dioxa-8-azaspiro[4.5]decan-8-yl)ethan-1-one (2z)

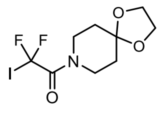

2z

Prepared according to General Procedure C, the title compound was obtained as a white solid (1.32 g, 3.78 mmol, 63% yield). **R$_f$** = 0.30 [Hexanes: EtOAc 10:1 (v/v)]. **^{1}H NMR** (500 MHz, $CDCl_3$) δ 3.98 (s, 1H), 3.73 (dt, J = 11.0, 5.7 Hz, 1H), 1.83 – 1.71 (m, 1H). **^{13}C NMR** (126 MHz, $CDCl_3$) δ 158.70 (t, J = 23.9 Hz), 106.36, 89.89 (t, J = 322.0 Hz), 64.71, 45.09 (t, J = 4.7 Hz), 42.44, 34.72 (d, J = 4.6 Hz). **^{19}F NMR** (470 MHz, $CDCl_3$) δ -50.28. **HRMS** (ESI+) *m/z* calcd for $C_9H_{13}F_2INO_3$ [(M + H)$^+$], 347.9908, found, 347.9911.

***N*,*N*-diethyl-2,2-difluoro-2-iodoacetamide (2aa)**

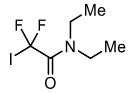

2aa

Prepared according to General Procedure B, the title compound was obtained as a yellow oil (0.8 g, 2.88 mmol, 48% yield). **R$_f$** = 0.6 [Hexanes: EtOAc 10:1 (v/v)]. **^{1}H NMR** (500 MHz, $CDCl_3$) δ 3.49 (qt, J = 7.1, 1.7 Hz, 2H), 3.41 (q, J = 7.1 Hz, 2H), 1.24 (t, J = 7.0 Hz, 3H), 1.17 (t, J = 7.1 Hz, 3H). **^{13}C NMR** (126 MHz, $CDCl_3$) δ 159.70 (t, J = 23.5 Hz), 91.17 (t, J = 322.7 Hz), 43.62 (t, J = 4.2 Hz), 42.39, 13.77, 11.68. **^{19}F NMR** (470 MHz, $CDCl_3$) δ -50.57.

***N*-((3s,5s,7s)-adamantan-1-yl)-2,2-difluoro-2-iodoacetamide (2ab)**

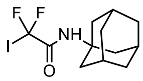

2ab

Prepared according to General Procedure A, the title compound was obtained as a white solid (0.32 g, 0.90 mmol, 15% yield). **R$_f$** = 0.46 [Hexanes: EtOAc 10:1 (v/v)]. **^{1}H NMR** (500 MHz, $CDCl_3$) δ 5.82 (s, 1H), 2.12 – 2.07 (m, 3H), 2.00 (d, J = 3.1 Hz, 6H), 1.67 (t, J = 3.2 Hz, 6H). **^{13}C NMR** (126 MHz, $CDCl_3$) δ 159.86 (t, J = 23.4 Hz), 92.41 (t, J = 325.9 Hz), 40.88, 36.12, 29.35. **^{19}F NMR** (470 MHz, $CDCl_3$) δ -55.95. **HRMS** (ESI+) *m/z* calcd for $C_{12}H_{17}F_2INO$ [(M + H)$^+$], 356.0322, found, 356.0324.

***N*-(tert-butyl)-2,2-difluoro-2-iodoacetamide (2ac)**

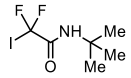

2ac

Prepared according to General Procedure C, the title compound was obtained as a white solid (1.1 g, 3.96 mmol, 66% yield). **R$_f$** = 0.4 [Hexanes: EtOAc 10:1 (v/v)]. **^{1}H NMR** (500 MHz, $CDCl_3$) δ 5.89 (s, 1H), 1.40

(s, 9H). **^{13}C NMR** 13C NMR (126 MHz, $CDCl_3$) δ 160.32, 92.38, 52.65, 28.28. **^{19}F NMR** (470 MHz, $CDCl_3$) δ -56.10.

2,2-difluoro-2-iodo-*N*-(3-phenylpropyl)acetamide (2ad)

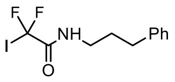

2ad

Prepared according to General Procedure A, the title compound was obtained as a yellowish solid (1.1 g, 3.24 mmol, 54% yield). **R_f** = 0.48 [Hexanes: EtOAc 10:1 (v/v)]. **^{1}H NMR** (500 MHz, $CDCl_3$) δ 7.35 – 7.27 (m, 2H), 7.26 – 7.16 (m, 3H), 6.25 (s, 1H), 3.38 (h, J = 6.6 Hz, 2H), 2.70 (p, J = 7.7 Hz, 2H), 1.94 (tt, J = 7.6, 6.7 Hz, 2H). **^{13}C NMR** (126 MHz, $CDCl_3$) δ 161.51 (t, J = 24.6 Hz), 140.86, 128.77, 128.45, 126.42, 91.26 (t, J = 323.9 Hz), 39.85, 33.18, 30.52. **^{19}F NMR** (470 MHz, $CDCl_3$) δ -56.62. **HRMS** (ESI+) *m/z* calcd for $C_{11}H_{16}F_2IN_2O$ [M + NH4)$^+$], 357.0275, found, 357.0274.

2,2-difluoro-2-iodo-*N*-phenylacetamide (2ae)

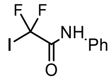

2ae

Prepared according to General Procedure B, the title compound was obtained as a yellow oil (0.43 g, 1.44 mmol, 24% yield). **R_f** = 0.4 [Hexanes: EtOAc 10:1 (v/v)]. **^{1}H NMR** (500 MHz, $CDCl_3$) δ 7.74 (s, 1H), 7.61 – 7.53 (m, 2H), 7.45 – 7.36 (m, 2H), 7.29 – 7.20 (m, 1H). **^{13}C NMR** (126 MHz, $CDCl_3$) δ 159.05 (t, J = 24.8 Hz), 135.39, 129.43, 126.26, 120.66, 90.69 (t, J = 324.6 Hz). **^{19}F NMR** (470 MHz, $CDCl_3$) δ -56.74.

((difluoroiodomethyl)sulfonyl)benzene (2aj)

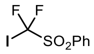

2aj

Prepared according to literature procedure [7], the title compound was obtained as a yellow oil (0.61 g, 1.92 mmol, 32% yield). **R_f** = 0.6 [Hexanes: EtOAc 10:1 (v/v)]. **^{1}H NMR** (500 MHz, $CDCl_3$) δ 8.04 – 7.97 (m, 2H), 7.82 (ddt, J = 8.8, 7.2, 1.3 Hz, 1H), 7.70 – 7.60 (m, 2H). **^{13}C NMR** (126 MHz, $CDCl_3$) δ 136.24, 131.56, 129.82, 128.40, 107.77 – 98.54 (m). **^{19}F NMR** (470 MHz, $CDCl_3$) δ –51.85.

(1R,2S,5R)-2-isopropyl-5-methylcyclohexyl 2,2-difluoro-2-iodoacetate (2aaa)

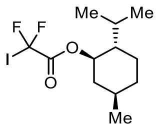

2aaa

Prepared according to General Procedure A, the title compound was obtained as a faint yellow oil (1.36 g, 3.78 mmol, 63% yield). **R$_f$** = 0.6 [Hexanes: EtOAc 10:1 (v/v)]. **^{1}H NMR** (500 MHz, $CDCl_3$) δ 4.83 (td, J = 11.0, 4.4 Hz, 1H), 2.06 (dddd, J = 11.9, 4.4, 3.5, 1.9 Hz, 1H), 1.95 (dtd, J = 14.2, 7.1, 4.3 Hz, 1H), 1.78 – 1.67 (m, 2H), 1.59 – 1.47 (m, 2H), 1.19 – 1.03 (m, 2H), 0.98 – 0.85 (m, 7H), 0.79 (d, J = 7.0 Hz, 3H). **^{13}C NMR** (126 MHz, $CDCl_3$) δ 160.33 (t, J = 27.4 Hz), 87.03 (t, J = 322.2 Hz), 79.41, 47.01, 39.86, 34.10, 31.53, 26.33, 23.49, 22.03, 20.77, 16.26. **^{19}F NMR** (470 MHz, $CDCl_3$) δ -57.29.

(2S,4R)-4,7,7-trimethylbicyclo[2.2.1]heptan-2-yl 2,2-difluoro-2-iodoacetate (2aab)

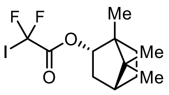

2aab

Prepared according to General Procedure A, the title compound was obtained as a colorless oil (1.63 g, 4.56 mmol, 76% yield). **R$_f$** = 0.62 [Hexanes: EtOAc 10:1 (v/v)]. **^{1}H NMR** (500 MHz, $CDCl_3$) δ 5.07 (ddd, J = 9.9, 3.4, 2.2 Hz, 1H), 2.41 (dddd, J = 14.4, 9.9, 4.7, 3.3 Hz, 1H), 1.98 (ddd, J = 13.4, 9.4, 4.4 Hz, 1H), 1.85 – 1.73 (m, 2H), 1.42 – 1.34 (m, 1H), 1.30 (ddd, J = 11.9, 9.3, 4.5 Hz, 1H), 1.11 (dd, J = 14.0, 3.4 Hz, 1H), 0.96 – 0.77 (m, 9H). **^{13}C NMR** (126 MHz, $CDCl_3$) δ 160.63 (t, J = 27.9 Hz), 87.14 (d, J = 322.3 Hz), 84.81, 49.54, 48.41, 35.93, 28.00, 26.95, 19.76, 18.96, 13.57. **^{19}F NMR** (470 MHz, $CDCl_3$) δ -57.19 (d, J = 12.5 Hz).

(3R,8R,9R,10S,13S,14R,17S)-10,13-dimethyl-17-((S)-6-methylheptan-2-yl)-2,3,4,7,8,9,10,11,12,13,14,15,16,17-tetradecahydro-1H-cyclopenta[a]phenanthren-3-yl 2,2-difluoro-2-iodoacetate (2aac)

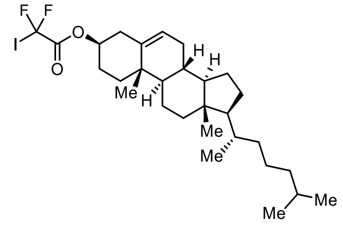

2aac

Prepared according to General Procedure A, the title compound was obtained as a white solid (1.48 g, 2.25 mmol, 42% yield). **R$_f$** = 0.52 [Hexanes: EtOAc 10:1 (v/v)]. **^{1}H NMR** (500 MHz, $CDCl_3$) δ 5.43 (dt, J = 5.4, 2.0 Hz, 1H), 4.84 – 4.74 (m, 1H), 2.52 – 2.37 (m, 2H), 2.09 – 1.90 (m, 4H), 1.87 – 1.69 (m, 2H), 1.63 – 1.51 (m, 7H), 1.44 – 0.90 (m, 19H), 0.87 (dd, J = 6.6, 2.2 Hz, 6H), 0.68 (s, 3H). **^{13}C NMR** (126 MHz, $CDCl_3$) δ 159.96, 138.68, 123.88, 87.28, 78.75, 56.81, 56.29, 50.12, 42.47, 39.84, 39.67, 37.38, 36.85, 36.70, 36.34, 35.94, 32.01 (d, J = 10.3 Hz), 28.37, 28.17, 27.20, 24.43, 23.98, 22.97, 22.71, 21.20, 19.42, 18.87, 12.01. **^{19}F NMR** (470 MHz, $CDCl_3$) δ -57.42.

***tert*-butyl 8-(2,2-difluoro-2-iodoacetyl)-3,8-diazabicyclo[3.2.1]octane-3-carboxylate (2aad)**

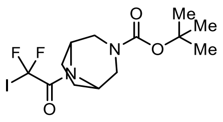

2aad

Prepared according to General Procedure A, the title compound was obtained as a white solid (1.12 g, 2.7 mmol, 45% yield). $\mathbf{R}_f$ = 0.18 [Hexanes: EtOAc 10:1 (v/v)]. **^{1}H NMR** (500 MHz, $CDCl_3$) δ 4.76 – 4.34 (m, 2H), 4.03 – 3.59 (m, 2H), 3.31 – 2.74 (m, 2H), 2.01 (dq, J = 15.1, 8.2 Hz, 1H), 1.92 – 1.63 (m, 3H), 1.39 (s, 9H). **^{13}C NMR** (126 MHz, $CDCl_3$) δ 156.39 (t, J = 25.3 Hz), 155.42, 89.79 (t, J = 322.0 Hz), 80.42, 56.12, 55.83, 53.09, 52.83, 50.31, 50.00, 49.12, 48.83, 28.31, 26.74, 25.12. **^{19}F NMR** (470 MHz, $CDCl_3$) δ -50.57 – -52.79 (m).

6-ethoxy-5,5-difluoro-3-iodo-2-methyl-6-oxohexan-2-yl benzoate (5)

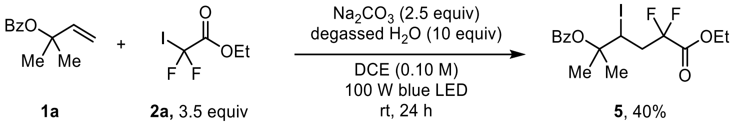

In a glovebox, to an oven-dried 20 mL screw cap vial was added Na_2CO_3 (53 mg, 0.50 mmol, 2.5 equiv), **1a** (38 mg, 0.20 mmol, 1.0 equiv), **2a** (175 mg, 0.70 mmol, 3.5 equiv), and DCE (2 mL, 0.10 M). The vial was equipped with a stir bar, capped and sealed with black tape, then taken out of the glovebox, subsequently degassed H_2O (36 μL, 2.0 mmol, 10 equiv) was added using syringe through septum. The reaction mixture was stirred at room temperature and irradiated with 100 W Blue LEDs for 24 h. The reaction mixture was then concentrated *in vacuo* and residue was purified by flash column chromatography on silica gel to afford the desired product as a colorless oil (35.2 mg, 0.08 mmol, 40% yield). $\mathbf{R}_f$ = 0.6 [Hexanes: EtOAc 3: 1 (v/v)]. **^{1}H NMR** (500 MHz, $CDCl_3$) δ 8.04 – 7.98 (m, 2H), 7.56 (ddt, *J* = 7.8, 7.0, 1.3 Hz, 1H), 7.48 – 7.40 (m, 2H), 4.88 – 4.82 (m, 1H), 4.42 – 4.29 (m, 2H), 3.03 – 2.79 (m, 2H), 1.80 (d, *J* = 4.8 Hz, 6H), 1.37 (t, *J* = 7.2 Hz, 3H). **^{13}C NMR** (126 MHz, $CDCl_3$) δ 165.39, 163.67 (t, *J* = 32.2 Hz), 133.26, 131.00, 128.62, 121.43 – 107.62 (m), 83.13, 63.56, 41.26 (t, *J* = 24.1 Hz), 28.93 (t, *J* = 4.2 Hz), 25.45, 23.78, 14.07. **^{19}F NMR** (470 MHz, $CDCl_3$) δ -102.68 (dt, *J* = 261.8, 14.3 Hz, 1F), -106.48 (dt, *J* = 262.0, 16.3 Hz, 1F).

General Procedure D (for the 1,3-carbohydroxylation of allyl esters):

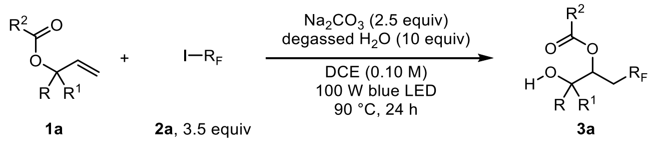

In a glovebox, to an oven-dried 20 mL screw cap vial was added Na_2CO_3 (53 mg, 0.50 mmol, 2.5 equiv), allyl ester (0.20 mmol, 1.0 equiv), alkyl iodide (0.70 mmol, 3.5 equiv), and DCE (2 mL, 0.10 M). The vial was equipped with a stir bar, capped and sealed with black tape, then taken out of the glovebox, subsequently degassed H_2O (36 μL, 2.0 mmol, 10 equiv) was added using syringe through septum. The reaction mixture was stirred at 90 °C and irradiated with 100 W Blue LEDs for 24 h (as the setup described

above). The reaction mixture was then concentrated *in vacuo* and residue was purified by flash column chromatography on silica gel to afford the desired product.

6-ethoxy-5,5-difluoro-2-hydroxy-2-methyl-6-oxohexan-3-yl benzoate (3a)

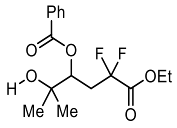

3a

Prepared according to General Procedure D, the title compound was obtained as a colorless oil (50.0 mg, 0.152 mmol, 76% yield). $\mathbf{R}_f$ = 0.16 [Hexanes: EtOAc 3:1 (v/v)]. **^{1}H NMR** (500 MHz, $CDCl_3$) δ 8.09 – 7.96 (m, 2H), 7.62 – 7.54 (m, 1H), 7.51 – 7.40 (m, 2H), 5.41 (dd, J = 9.6, 2.5 Hz, 1H), 4.20 – 4.10 (m, 1H), 4.11 – 4.01 (m, 1H), 2.75 – 2.52 (m, 2H), 1.29 (m, 6H), 1.21 (t, J = 7.1 Hz, 3H). **^{13}C NMR** (126 MHz, $CDCl_3$) δ 13C NMR (126 MHz, $CDCl_3$) δ 165.77, 163.94 (t, J = 32.3 Hz), 133.49, 129.90, 128.61, 115.45 (t, J = 251.5 Hz), 73.68, 72.51, 63.18, 34.90 (t, J = 23.5 Hz), 29.84, 25.85, 13.85. **^{19}F NMR** (470 MHz, $CDCl_3$) δ -102.69 (dt, J = 267.4, 14.8 Hz, 1F), -105.41 (dt, J = 267.2, 16.5 Hz, 1F). **HRMS** (ESI+) *m/z* calcd for $C_{16}H_{21}F_2O_5$ [(M + H)$^+$], 331.1357, found, 331.1353.

4-ethoxy-3,3-difluoro-1-(1-hydroxycyclopentyl)-4-oxobutyl benzoate (3b)

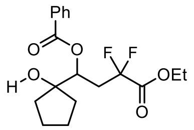

3b

Prepared according to General Procedure D, the title compound was obtained as a colorless oil (39.2 mg, 0.11 mmol, 55% yield). $\mathbf{R}_f$ = 0.23 [Hexanes: EtOAc 3:1 (v/v)]. **^{1}H NMR** (500 MHz, $CDCl_3$) δ 8.08 – 7.97 (m, 2H), 7.64 – 7.54 (m, 1H), 7.50 – 7.41 (m, 2H), 5.50 (dd, J = 10.2, 1.9 Hz, 1H), 4.20 – 4.11 (m, 1H), 4.07 (dq, J = 10.8, 7.2 Hz, 1H), 2.83 (dtd, J = 17.3, 15.2, 10.2 Hz, 1H), 2.56 (qd, J = 15.7, 1.9 Hz, 1H), 1.96 – 1.84 (m, 1H), 1.83 – 1.75 (m, 2H), 1.73 – 1.64 (m, 4H), 1.62 – 1.53 (m, 2H), 1.22 (t, J = 7.2 Hz, 3H).**^{13}C NMR** (126 MHz, $CDCl_3$) δ 165.48, 163.86 (t, J = 32.2 Hz),, 133.35, 129.76, 128.50, 115.35 (t, J = 251.8 Hz), 84.16, 73.29, 63.02, 37.97, 36.47, 35.51 (t, J = 23.5 Hz), 23.94, 23.65, 13.75. **^{19}F NMR** (470 MHz, $CDCl_3$) δ -102.81 (dt, J = 267.4, 15.4 Hz, 1F), -105.30 (dt, J = 267.4, 16.7 Hz, 1F). **HRMS** (ESI+) *m/z* calcd for $C_{18}H_{23}F_2O_5$ [(M + H)$^+$], 357.1513, found, 357.1511.

4-ethoxy-3,3-difluoro-1-(4-hydroxytetrahydro-2H-pyran-4-yl)-4-oxobutyl benzoate (3c)

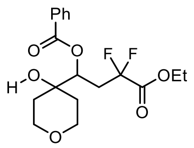

3c

Prepared according to General Procedure D, the title compound was obtained as a yellow oil (49.8 mg, 0.134 mmol, 67% yield). **R$_f$** = 0.10 [Hexanes: EtOAc 3:1 (v/v)]. **^{1}H NMR** (500 MHz, $CDCl_3$) δ 8.03 – 8.01 (m, 2H), 7.62 – 7.55 (m, 1H), 7.45 (ddd, J = 7.7, 6.9, 1.1 Hz, 2H), 5.38 (dd, J = 10.0, 2.2 Hz, 1H), 4.13 (dq, J = 10.7, 7.1 Hz, 1H), 4.06 (dq, J = 10.8, 7.2 Hz, 1H), 3.86 – 3.78 (m, 2H), 3.73 (qd, J = 11.9, 2.4 Hz, 2H), 2.80 – 2.48 (m, 2H), 1.84 (ddd, J = 13.9, 12.1, 5.3 Hz, 1H), 1.76 (ddd, J = 13.7, 11.9, 5.4 Hz, 1H), 1.51 (dt, J = 13.7, 2.5 Hz, 1H), 1.43 (dd, J = 13.9, 2.5 Hz, 1H), 1.21 (t, J = 7.2 Hz, 3H). **^{13}C NMR** (126 MHz, $CDCl_3$) δ 165.67, 163.87 (t, J = 32.3 Hz), 133.66, 129.90, 129.41, 128.66, 115.45 (t, J = 251.9 Hz), 73.66, 70.95, 63.24, 62.99, 34.20 – 33.26 (m), 13.82. **^{19}F NMR** (470 MHz, $CDCl_3$) δ -102.61 (dt, J = 267.9, 15.1 Hz, 1F), -105.09 (dt, J = 267.9, 16.5 Hz, 1F). **HRMS** (ESI+) *m/z* calcd for $C_{18}H_{23}F_2O_6$ [(M + H)$^+$], 373.1462, found, 373.1457.

4-ethoxy-3,3-difluoro-1-(8-hydroxy-1,4-dioxaspiro[4.5]decan-8-yl)-4-oxobutyl benzoate (3d)

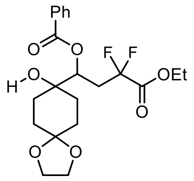

3d

Prepared according to General Procedure D, the title compound was obtained as a yellow oil (64.2 mg, 0.15 mmol, 75% yield). **R$_f$** = 0.1 [Hexanes: EtOAc 3:1 (v/v)]. **^{1}H NMR** (500 MHz, $CDCl_3$) δ 8.03 (dd, J = 8.4, 1.4 Hz, 2H), 7.60 – 7.56 (m, 1H), 7.47 – 7.43 (m, 2H), 5.41 (dd, J = 10.1, 2.1 Hz, 1H), 4.13 (dq, J = 10.8, 7.1 Hz, 1H), 4.05 (dq, J = 10.7, 7.1 Hz, 1H), 3.98 – 3.85 (m, 4H), 2.79 – 2.67 (m, 1H), 2.66 – 2.54 (m, 1H), 1.98 – 1.77 (m, 3H), 1.76 – 1.70 (m, 3H), 1.68 – 1.57 (m, 4H), 1.21 (t, J = 7.2 Hz, 3H). **^{13}C NMR** (126 MHz, $CDCl_3$) δ 165.69, 163.90 (t, J = 32.5 Hz), 133.51, 130.20, 129.94, 129.67, 128.62, 115.49 (t, J = 251.9 Hz), 108.30, 73.70, 72.38, 64.52, 64.34, 63.18, 34.46 (t, J = 23.5 Hz), 31.39, 30.85, 29.88, 13.85. **^{19}F NMR** (470 MHz, $CDCl_3$) δ -102.67 (dt, J = 267.4, 15.0 Hz, 1F), -105.12 (dt, J = 267.6, 16.5 Hz, 1F).

4-ethoxy-3,3-difluoro-1-(1-hydroxycycloheptyl)-4-oxobutyl benzoate (3e)

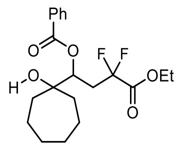

3e

Prepared according to General Procedure D, the title compound was obtained as a colorless oil (48.3 mg, 0.126 mmol, 63% yield). **R*f*** = 0.2 [Hexanes: EtOAc 3:1 (v/v)]. **^{1}H NMR** (500 MHz, $CDCl_3$) 8.07 – 7.98 (m, 2H), 7.61 – 7.54 (m, 1H), 7.50 – 7.40 (m, 2H), 5.43 (dd, J = 9.9, 2.1 Hz, 1H), 4.37 – 4.27 (m, 1H), 4.18 – 3.99 (m, 2H), 2.77 – 2.54 (m, 2H), 1.80 – 1.69 (m, 3H), 1.69 – 1.59 (m, 5H), 1.50 (dtt, J = 9.4, 7.3, 4.9 Hz, 4H), 1.21 (t, J = 7.1 Hz, 3H). **^{13}C NMR** (126 MHz, $CDCl_3$) δ 165.76, 163.96, 133.44, 129.86 (d, J = 11.7 Hz), 128.60, 117.61 – 113.61 (m), 73.89, 63.13, 37.52, 37.13, 34.56 (t, J = 23.3 Hz), 29.89, 29.66, 22.20 (d, J = 9.6 Hz), 13.86. **^{19}F NMR** (470 MHz, $CDCl_3$) δ -97.95 – -103.57 (m, 1F), -103.85 – -108.18 (m, 1F). **HRMS** (ESI+) *m/z* calcd for $C_{20}H_{27}F_2O_5$ [$(M + H)^+$], 385.1826, found, 385.1819.

4-ethoxy-3,3-difluoro-1-(1-hydroxycyclododecyl)-4-oxobutyl benzoate (3f)

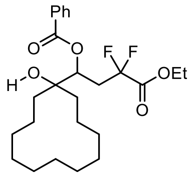

3f

Prepared according to General Procedure D, the title compound was obtained as a white solid (54.4 mg, 0.120 mmol, 60% yield). **R*f*** = 0.10 [Hexanes: EtOAc 3:1 (v/v)]. **^{1}H NMR** (500 MHz, $CDCl_3$) δ 1H NMR (500 MHz, $CDCl_3$) δ 8.04 (dd, J = 8.3, 1.4 Hz, 2H), 7.60 (d, J = 5.6 Hz, 0H), 7.50 – 7.42 (m, 1H), 5.44 (dd, J = 10.4, 1.9 Hz, 1H), 4.14 (dq, J = 10.8, 7.2 Hz, 1H), 4.07 (dq, J = 10.8, 7.2 Hz, 1H), 2.80 (dtd, J = 16.6, 15.2, 10.4 Hz, 1H), 2.60 (qd, J = 15.9, 1.9 Hz, 1H), 1.82 – 1.66 (m, 2H), 1.55 – 1.29 (m, 20H), 1.22 (t, J = 7.2 Hz, 3H). **^{13}C NMR** (126 MHz, $CDCl_3$) δ 165.67, 163.94 (t, J = 32.2 Hz), 133.46, 129.94, 129.81, 128.63, 115.73 (t, J = 251.8 Hz), 72.62, 63.12, 34.20 (t, J = 23.3 Hz), 31.55, 31.52, 26.56, 26.45, 26.12, 22.76, 22.60, 22.24, 22.15, 19.28, 19.22, 13.86. **^{19}F NMR** (470 MHz, $CDCl_3$) δ -102.32 (dt, J = 268.4, 15.6 Hz, 1F), -104.40 (dt, J = 268.2, 16.2 Hz, 1F). **HRMS** (ESI+) *m/z* calcd for $C_{25}H_{37}F_2O_5$ [$(M + H)^+$], 455.2609, found, 455.2599.

1-(1-bromocycloheptyl)-4-ethoxy-3,3-difluoro-4-oxobutyl benzoate (3g)

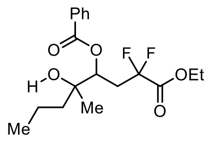

3g

Prepared according to General Procedure D, the title compound was obtained as a colorless oil (41.5 mg, 0.116 mmol, 58% yield, dr 1:0.9). **R*f*** = 0.20 [Hexanes: EtOAc 3:1 (v/v)]. **^{1}H NMR** (500 MHz, $CDCl_3$) δ 8.14 – 7.96 (m, 2H), 7.58 (ddt, J = 8.8, 7.0, 1.3 Hz, 1H), 7.52 – 7.38 (m, 2H), 5.44 (dt, J = 9.7, 1.9 Hz, 1H), 4.24 – 3.91 (m, 2H), 2.81 – 2.44 (m, 2H), 1.83 – 1.68 (m, 1H), 1.60 – 1.34 (m, 4H), 1.24 – 1.18 (m, 6H), 0.97 – 0.89 (m, 3H). **^{13}C NMR** (126 MHz, $CDCl_3$) δ 165.92, 165.67, 163.81 (d, J = 36.8 Hz), 133.46, 129.85 (d, J = 13.5 Hz), 128.61, 115.45, 74.47, 73.07 (d, J = 21.4 Hz), 63.17 (d, J = 3.7 Hz), 40.70, 34.78 (dt, J = 37.7, 23.5 Hz), 23.09, 22.53, 16.62 (d, J = 8.3 Hz), 14.72, 13.85. **^{19}F NMR** (470 MHz, $CDCl_3$) δ -102.58 (ddt, J = 267.6, 101.7, 15.1 Hz, 1F), -105.13 (ddt, J = 267.2, 153.5, 16.4 Hz, 1F). **HRMS** (ESI+) *m/z* calcd for $C_{18}H_{25}F_2O_5$ [(M + H)$^+$], 359.1670, found, 359.1663.

8-acetoxy-1-ethoxy-2,2-difluoro-5-hydroxy-5-methyl-1-oxooctan-4-yl benzoate (3h)

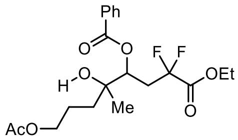

3h

Prepared according to General Procedure D, the title compound was obtained as a colorless oil (62.4 mg, 0.15 mmol, 75% yield, dr 1:1). **R*f*** = 0.21 [Hexanes: EtOAc 3:1 (v/v)]. **^{1}H NMR** (500 MHz, $CDCl_3$) δ 8.05 – 7.98 (m, 2H), 7.57 (ddt, J = 7.8, 7.1, 1.5 Hz, 1H), 7.50 – 7.40 (m, 2H), 5.48 – 5.41 (m, 1H). 4.34 – 3.77 (m, 4H), 2.74 – 2.50 (m, 2H), 1.98 (d, J = 16.3 Hz, 3H), 1.88 – 1.67 (m, 2H), 1.66 – 1.47 (m, 2H), 1.24 (d, J = 2.7 Hz, 3H), 1.20 (td, J = 7.2, 3.6 Hz, 3H). **^{13}C NMR** (126 MHz, $CDCl_3$) δ 171.29, 165.81, 163.87, 133.52 (d, J = 3.9 Hz), 129.87 (d, J = 2.7 Hz), 128.60, 115.38, 74.03, 73.80, 73.13, 72.76, 64.64 (d, J = 5.6 Hz), 37.61 – 30.42 (m), 22.75 (d, J = 14.3 Hz), 22.48, 20.97 (d, J = 5.7 Hz), 13.80. **^{19}F NMR** (470 MHz, $CDCl_3$) δ -102.60 (ddt, J = 267.8, 75.8, 15.0 Hz, 1F), -105.20 (ddt, J = 267.8, 74.6, 16.5 Hz, 1F). **HRMS** (ESI+) *m/z* calcd for $C_{20}H_{27}F_2O_7$ [(M + H)$^+$], 417.1724, found, 417.1717.

8-bromo-1-ethoxy-2,2-difluoro-5-hydroxy-5-methyl-1-oxooctan-4-yl benzoate (3i)

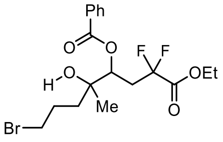

3i

Prepared according to General Procedure D, the title compound was obtained as a faint yellow oil (62.0 mg, 0.142 mmol, 71% yield, dr 1:1). **R*f*** = 0.16 [Hexanes: EtOAc 3:1 (v/v)]. **^{1}H NMR** (500 MHz, $CDCl_3$) δ 8.15 – 7.80 (m, 2H), 7.63 – 7.52 (m, 1H), 7.50 – 7.40 (m, 2H), 5.53 – 5.24 (m, 1H), 4.20 – 4.00 (m, 2H), 3.49 – 3.35 (m, 2H), 2.75 – 2.45 (m, 2H), 2.15 – 1.89 (m, 2H), 1.76 – 1.56 (m, 3H), 1.33 – 1.17 (m, 6H). **^{13}C NMR** (126 MHz, $CDCl_3$) δ 165.86 (d, J = 21.6 Hz), 163.83, 133.60 (d, J = 3.6 Hz), 129.59, 128.60 (d, J = 13.0 Hz), 116.33 (d, J = 251.9 Hz), 74.13, 73.09 (d, J = 33.6 Hz), 63.24, 36.91, 36.54, 35.30 – 34.21 (m), 34.12 (d, J = 4.5 Hz), 26.81 (d, J = 3.3 Hz), 22.57, 13.86. **^{19}F NMR** (470 MHz, $CDCl_3$) δ -101.08 – -103.92 (m, 1F), -105.07 (ddt, J = 268.3, 63.5, 16.4 Hz, 1F). **HRMS** (ESI+) *m/z* calcd for $C_{18}H_{24}BrF_2O_5$ [(M + H)$^+$], 437.0775, found, 437.0768.

8-chloro-1-ethoxy-2,2-difluoro-5-hydroxy-5-methyl-1-oxooctan-4-yl benzoate (3j)

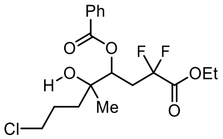

3j

Prepared according to General Procedure D, the title compound was obtained as a yellow oil (47.0 mg, 0.12 mmol, 60% yield, dr 1:1). **R*f*** = 0.10 [Hexanes: EtOAc 3:1 (v/v)]. **^{1}H NMR** (500 MHz, $CDCl_3$) δ 8.20 – 7.88 (m, 2H), 7.69 – 7.49 (m, 1H), 7.49 – 7.35 (m, 2H), 5.48 – 5.38 (m, 1H), 4.18 – 4.09 (m, 1H), 4.09 – 4.00 (m, 1H), 3.67 – 3.31 (m, 2H), 2.82 – 2.45 (m, 2H), 2.09 – 1.80 (m, 3H), 1.80 – 1.54 (m, 2H), 1.25 (d, J = 4.2 Hz, 3H), 1.21 (td, J = 7.2, 3.4 Hz, 3H). **^{13}C NMR** (126 MHz, $CDCl_3$) δ 165.92, 163.85 (t, J = 32.3 Hz), 133.60 (d, J = 3.4 Hz), 129.89, 129.51 (d, J = 3.4 Hz), 128.64, 117.38 – 113.32 (m), 74.08, 73.04 (d, J = 32.6 Hz), 63.25, 45.44, 35.54, 34.98 – 34.13 (m), 26.61 (d, J = 3.7 Hz), 23.09, 22.45, 13.83. **^{19}F NMR** (470 MHz, $CDCl_3$) δ -102.65 (ddt, J = 267.7, 65.8, 15.0 Hz, 1F), -105.29 (ddt, J = 267.8, 72.2, 16.4 Hz, 1F). **HRMS** (ESI+) *m/z* calcd for $C_{18}H_{24}ClF_2O_5$ [(M + H)$^+$], 393.1280, found, 393.1272.

8-(1,3-dioxoisoindolin-2-yl)-1-ethoxy-2,2-difluoro-5-hydroxy-5-methyl-1-oxooctan-4-yl benzoate (3k)

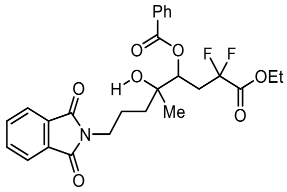

3k

Prepared according to General Procedure D, the title compound was obtained as a yellow oil (56.3 mg, 0.112 mmol, 56% yield, dr 1:1). **R*f*** = 0.5 [Hexanes: EtOAc 3:1 (v/v)]. **^{1}H NMR** (500 MHz, $CDCl_3$) δ 8.05 – 7.90 (m, 2H), 7.84 – 7.72 (m, 2H), 7.71 – 7.64 (m, 2H), 7.58 – 7.50 (m, 1H), 7.45 – 7.35 (m, 2H), 5.41 (td, J = 9.8, 2.5 Hz, 1H), 4.21 – 4.08 (m, 1H), 4.08 – 3.96 (m, 1H), 3.71 (td, J = 6.9, 4.4 Hz, 2H), 2.75 –

2.50 (m, 2H), 2.32 (d, J = 16.3 Hz, 1H), 1.96 – 1.73 (m, 2H), 1.69 – 1.47 (m, 2H), 1.23 (d, J = 2.1 Hz, 3H), 1.20 (q, J = 7.2 Hz, 3H). **^{13}C NMR** 13C NMR (126 MHz, $CDCl_3$) δ 168.63 (d, J = 3.2 Hz), 165.72, 163.96 (d, J = 32.3 Hz), 134.04 (d, J = 4.3 Hz), 132.13 (d, J = 6.3 Hz), 129.87 (d, J = 6.8 Hz), 129.61 (d, J = 10.3 Hz), 128.53 (d, J = 3.7 Hz), 115.46, 74.16, 73.92, 73.25, 72.85, 63.14, 38.17 (d, J = 4.8 Hz), 34.73 (d, J = 9.4 Hz), 22.97, 22.78, 22.52, 22.43, 13.83. **^{19}F NMR** (470 MHz, $CDCl_3$) δ -102.67 (ddt, J = 267.8, 118.1, 15.3 Hz, 1F), -105.14 (ddt, J = 267.3, 91.9, 16.5 Hz, 1F). **HRMS** (ESI+) *m/z* calcd for $C_{26}H_{31}F_2O_7N_2$ [M + $NH_4)^+$], 521.2099, found, 521.2090.

5,5-difluoro-2-hydroxy-2-methyl-6-oxo-6-(2-(trimethylsilyl)ethoxy)hexan-3-yl benzoate (3l)

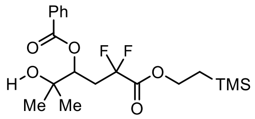

3l

Prepared according to General Procedure D, the title compound was obtained as a colorless oil (48.3 mg, 0.12 mmol, 60% yield). **R_f** = 0.31 [Hexanes: EtOAc 3:1 (v/v)]. **^{1}H NMR** (500 MHz, $CDCl_3$) δ 1H NMR (500 MHz, $CDCl_3$) δ 8.07 – 7.99 (m, 2H), 7.61 – 7.54 (m, 1H), 7.49 – 7.40 (m, 2H), 5.41 (dd, J = 10.0, 2.2 Hz, 1H), 4.13 (td, J = 11.1, 6.2 Hz, 1H), 4.05 (td, J = 11.0, 6.3 Hz, 1H), 2.74 – 2.53 (m, 2H), 1.93 (s, 1H), 1.28 (d, J = 2.6 Hz, 6H), 0.99 – 0.85 (m, 2H), -0.03 (s, 9H). **^{13}C NMR** (126 MHz, $CDCl_3$) δ 165.74, 164.07 (t, J = 32.0 Hz), 133.48, 129.92, 128.60, 121.37 – 110.19 (m), 73.69 (d, J = 4.9 Hz), 72.47, 65.79, 34.91 (t, J = 23.5 Hz), 25.84 (d, J = 10.1 Hz), 17.12, -1.53. **^{19}F NMR** (470 MHz, $CDCl_3$) δ -102.30 (dt, J = 266.3, 14.4 Hz, 1F), -105.93 (dt, J = 266.5, 17.0 Hz, 1F). **HRMS** (ESI+) *m/z* calcd for $C_{19}H_{32}F_2O_5SiN$ [M + $NH_4)^+$], 420.2017, found, 420.2013.

5,5-difluoro-2-hydroxy-6-((18-methoxy-18-oxooctadecan-7-yl)oxy)-2-methyl-6-oxohexan-3-yl benzoate (3m)

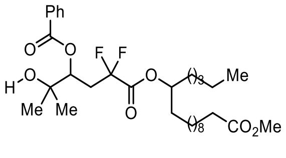

3m

Prepared according to General Procedure D, the title compound was obtained as a yellow oil (78.9 mg, 0.132 mmol, 66% yield). **R_f** = 0.24 [Hexanes: EtOAc 3:1 (v/v)]. **^{1}H NMR** (500 MHz, $CDCl_3$) δ 8.07 – 8.01 (m, 2H), 7.61 – 7.53 (m, 1H), 7.44 (ddt, J = 8.7, 7.4, 1.5 Hz, 2H), 5.46 (dd, J = 9.0, 2.8 Hz, 1H), 4.94 – 4.86 (m, 1H), 3.66 (s, 3H), 2.67 – 2.54 (m, 2H), 2.30 (t, J = 7.6 Hz, 2H), 1.81 (d, J = 1.3 Hz, 1H), 1.67 – 1.48 (m, 5H), 1.35 – 1.19 (m, 29H), 0.87 (td, J = 6.9, 3.4 Hz, 3H). **^{13}C NMR** 13C NMR (126 MHz, $CDCl_3$) δ

174.50, 165.80, 163.67, 133.33, 129.95, 128.55, 115.59, 78.37, 73.55, 72.51, 51.58, 34.25, 33.71, 31.78 (d, J = 3.2 Hz), 29.52, 29.43 – 29.11 (m), 25.93 (d, J = 10.5 Hz), 25.10 (d, J = 3.6 Hz), 22.68, 14.18. **^{19}F NMR** (470 MHz, $CDCl_3$) δ -103.78 (dddd, J = 269.4, 31.5, 17.9, 13.7 Hz, 1F), -104.68 (dtd, J = 269.4, 16.8, 5.4 Hz, 1F). **HRMS** (ESI+) *m/z* calcd for $C_{33}H_{53}F_2O_7$ [(M + H)$^+$], 599.3759, found, 599.3757.

6-(tert-butoxy)-5,5-difluoro-2-hydroxy-2-methyl-6-oxohexan-3-yl benzoate (3n)

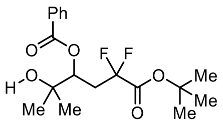

3n

Prepared according to General Procedure D, the title compound was obtained as a colorless oil (38.7 mg, 0.108 mmol, 54% yield). **R_f** = 0.28 [Hexanes: EtOAc 3:1 (v/v)]. **^{1}H NMR** (500 MHz, $CDCl_3$) δ 8.08 – 8.02 (m, 2H), 7.60 – 7.53 (m, 1H), 7.50 – 7.41 (m, 2H), 5.46 – 5.40 (m, 1H), 2.67 – 2.47 (m, 2H), 1.81 (s, 1H), 1.47 (s, 9H), 1.29 (d, J = 1.5 Hz, 6H). **^{13}C NMR** (126 MHz, $CDCl_3$) δ 165.89, 162.77, 133.33, 129.98 (d, J = 5.7 Hz), 128.55, 115.39 (t, J = 251.7 Hz), 84.93, 73.67, 72.56, 34.52 (t, J = 23.2 Hz), 27.81, 25.91. **^{19}F NMR** (470 MHz, $CDCl_3$) δ -103.24 (ddd, J = 267.4, 17.5, 12.9 Hz, 1F), -104.65 (dt, J = 267.3, 17.3 Hz, 1F). **HRMS** (ESI+) *m/z* calcd for $C_{18}H_{28}F_2O_5N$ [M + NH_4)$^+$], 376.1935, found, 376.1926.

6-(cyclopentyloxy)-5,5-difluoro-2-hydroxy-2-methyl-6-oxohexan-3-yl benzoate (3o)

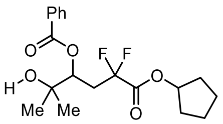

3o

Prepared according to General Procedure D, the title compound was obtained as a colorless oil (50.37 mg, 0.136 mmol, 68% yield). **R_f** = 0.3 [Hexanes: EtOAc 3:1 (v/v)]. **^{1}H NMR** (500 MHz, $CDCl_3$) δ 8.07 – 7.99 (m, 2H), 7.57 (ddt, J = 7.8, 7.0, 1.3 Hz, 1H), 7.48 – 7.40 (m, 2H), 5.42 (dd, J = 8.8, 3.3 Hz, 1H), 4.69 (tt, J = 9.2, 3.8 Hz, 1H), 2.72 – 2.53 (m, 2H), 1.97 (s, 1H), 1.88 – 1.78 (m, 1H), 1.76 – 1.63 (m, 3H), 1.54 – 1.35 (m, 3H), 1.28 (d, J = 2.2 Hz, 6H).**^{13}C NMR** (126 MHz, $CDCl_3$) δ 165.78, 163.27 (t, J = 31.6 Hz), 133.38, 129.90, 129.83, 128.53, 115.46 (t, J = 251.7 Hz), 76.20, 73.67 (d, J = 4.7 Hz), 72.45, 34.69 (t, J = 23.3 Hz), 31.12 (d, J = 8.0 Hz), 25.84, 25.19, 23.53. **^{19}F NMR** (470 MHz, $CDCl_3$) δ -103.40 (dt, J = 267.5, 15.5 Hz, 1F), -104.73 (dt, J = 267.7, 16.4 Hz, 1F).

5,5-difluoro-2-hydroxy-2-methyl-6-oxo-6-((tetrahydro-2H-pyran-4-yl)oxy)hexan-3-yl benzoate (3p)

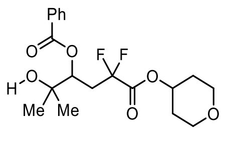

3p

Prepared according to General Procedure D, the title compound was obtained as a colorless oil (51 mg, 0.132 mmol, 66% yield). $\mathbf{R}_f$ = 0.1 [Hexanes: EtOAc 3:1 (v/v)]. **^{1}H NMR** 1H NMR (500 MHz, $CDCl_3$) δ 8.06 – 7.99 (m, 2H), 7.62 – 7.55 (m, 1H), 7.50 – 7.41 (m, 2H), 5.43 – 5.37 (m, 1H), 4.86 (tt, J = 8.3, 4.1 Hz, 1H), 3.90 – 3.80 (m, 2H), 3.45 (dddd, J = 18.0, 11.9, 8.6, 3.2 Hz, 2H), 2.70 – 2.56 (m, 2H), 1.93 – 1.84 (m, 2H), 1.83 – 1.74 (m, 1H), 1.70 (dtd, J = 11.5, 7.9, 3.4 Hz, 1H), 1.63 (ddt, J = 12.8, 8.5, 4.3 Hz, 1H), 1.29 (d, J = 2.8 Hz, 6H). **^{13}C NMR** δ 165.75, 163.22 (t, J = 32.3 Hz), 133.51, 129.83 (d, J = 14.9 Hz), 128.62, 115.47 (t, J = 252.0 Hz), 73.68, 72.49 (d, J = 16.3 Hz), 65.03, 34.74 (t, J = 23.3 Hz), 31.25 (d, J = 8.5 Hz), 25.89 (d, J = 17.4 Hz). **^{19}F NMR** (470 MHz, $CDCl_3$) δ -102.90 (dt, J = 269.7, 15.6 Hz, 1F), -104.23 (dt, J = 269.6, 15.9 Hz, 1F). **HRMS** (ESI+) *m/z* calcd for $C_{19}H_{25}F_2O_6$ [(M + H)$^+$], 387.1619, found, 387.1620.

6-(cycloheptyloxy)-5,5-difluoro-2-hydroxy-2-methyl-6-oxohexan-3-yl benzoate (3q)

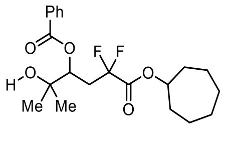

3q

Prepared according to General Procedure D, the title compound was obtained as a colorless oil (50.1 mg, 0.126 mmol, 63% yield). $\mathbf{R}_f$ = 0.25 [Hexanes: EtOAc 3:1 (v/v)]. **^{1}H NMR** (500 MHz, $CDCl_3$) δ 8.07 – 8.00 (m, 2H), 7.61 – 7.54 (m, 1H), 7.49 – 7.40 (m, 2H), 5.42 (dd, *J* = 9.0, 3.1 Hz, 1H), 4.86 (tt, *J* = 8.4, 4.4 Hz, 1H), 2.71 – 2.53 (m, 2H), 1.93 – 1.83 (m, 2H), 1.80 – 1.74 (m, 1H), 1.71 – 1.56 (m, 4H), 1.55 – 1.46 (m, 4H), 1.41 – 1.34 (m, 2H), 1.28 (d, *J* = 2.4 Hz, 6H). **^{13}C NMR** (125 MHz, $CDCl_3$) δ 165.79, 163.19 (t, *J* = 32.0 Hz), 133.40, 129.88 (d, *J* = 9.0 Hz), 128.55, 119.52 – 109.38 (m), 78.83, 73.69 (t, *J* = 4.1 Hz), 72.49, 34.67 (t, *J* = 23.4 Hz), 33.40 (d, *J* = 12.9 Hz), 28.15 (d, *J* = 2.7 Hz), 25.87 (d, *J* = 5.0 Hz), 22.73. **^{19}F NMR** (470 MHz, $CDCl_3$) δ -103.39 (dt, *J* = 267.9, 15.7 Hz, 1F), -104.78 (dt, *J* = 267.9, 16.0 Hz, 1F). **HRMS** (ESI+) *m/z* calcd for $C_{21}H_{28}F_2O_5Na$ [M + Na)$^+$], 421.1802, found, 421.1808.

6-(((3s,5s,7s)-adamantan-1-yl)oxy)-5,5-difluoro-2-hydroxy-2-methyl-6-oxohexan-3-yl benzoate (3r)

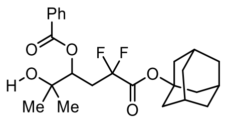

3r

Prepared according to General Procedure D, the title compound was obtained as a colorless oil (52.3 mg, 0.12 mmol, 60% yield). $\mathbf{R}_f$ = 0.29 [Hexanes: EtOAc 3:1 (v/v)]. **^{1}H NMR** (500 MHz, $CDCl_3$) δ 8.09 – 8.00 (m, 2H), 7.56 (ddt, J = 8.7, 7.0, 1.3 Hz, 1H), 7.44 (tt, J = 7.4, 1.6 Hz, 2H), 5.43 (dt, J = 9.3, 2.2 Hz, 1H), 2.63 – 2.48 (m, 2H), 2.27 – 2.04 (m, 9H), 1.95 (d, J = 1.9 Hz, 1H), 1.80 – 1.41 (m, 7H), 1.28 (s, 6H). **^{13}C NMR** (126 MHz, $CDCl_3$) δ 13C NMR (126 MHz, $CDCl_3$) δ 165.87, 162.31 (t, J = 31.7 Hz), 133.27, 129.99 (d, J = 7.0 Hz), 128.49, 115.26 (t, J = 251.7 Hz), 84.92, 73.71, 72.50, 40.98, 35.99, 34.56 (t, J = 23.3 Hz), 31.03, 25.85 (d, J = 4.0 Hz). **^{19}F NMR** (470 MHz, $CDCl_3$) δ -103.22 (ddd, J = 266.3, 16.5, 13.3 Hz, 1F), -104.39 (dt, J = 266.3, 16.9 Hz, 1F). **HRMS** (ESI+) *m/z* calcd for $C_{24}H_{30}F_2O_5Na$ [M + Na)$^+$], 459.1959, found, 459.1965.

5,5-difluoro-2-hydroxy-2-methyl-6-(octylthio)-6-oxohexan-3-yl benzoate (3s)

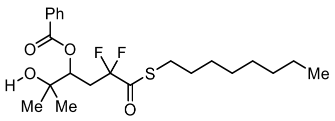

3s

Prepared according to General Procedure D, the title compound was obtained as a colorless oil (39.6 mg, 0.092 mmol, 46% yield). $\mathbf{R}_f$ = 0.3 [Hexanes: EtOAc 3:1 (v/v)]. **^{1}H NMR** (500 MHz, $CDCl_3$) δ 8.06 – 7.99 (m, 2H), 7.61 – 7.54 (m, 1H), 7.50 – 7.42 (m, 2H), 5.41 (dd, J = 10.4, 1.8 Hz, 1H), 2.82 – 2.64 (m, 2H), 2.63 – 2.49 (m, 2H), 1.31 – 1.11 (m, 18H), 0.88 (t, J = 7.0 Hz, 3H). **^{13}C NMR** (126 MHz, $CDCl_3$) δ 195.99 – 191.05 (m), 165.81, 133.35 (d, J = 14.5 Hz), 129.88 (d, J = 5.6 Hz), 128.57, 117.68, 78.35, 73.59, 72.53, 34.28 (t, J = 23.5 Hz), 31.89 (d, J = 6.6 Hz), 29.74 – 27.65 (m), 26.45, 26.00, 25.76, 22.75 (d, J = 4.1 Hz), 14.20. **^{19}F NMR** (470 MHz, $CDCl_3$) δ -99.03 (dt, J = 264.1, 14.4 Hz, 1F), -104.91 (dt, J = 264.2, 17.2 Hz, 1F). **HRMS** (ESI+) *m/z* calcd for $C_{22}H_{36}F_2O_4SN$ [M + NH_4)$^+$], 448.2333, found, 448.2335.

5,5-difluoro-2-hydroxy-6-((3-methoxy-3-oxopropyl)thio)-2-methyl-6-oxohexan-3-yl benzoate (3t)

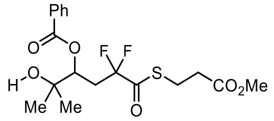

3t

Prepared according to General Procedure D, the title compound was obtained as a colorless oil (37.2 mg, 0.092 mmol, 46% yield). $\mathbf{R}_f$ = 0.3 [Hexanes: EtOAc 3:1 (v/v)]. **^{1}H NMR** (500 MHz, $CDCl_3$) δ 8.46 – 7.87 (m, 2H), 7.77 – 7.34 (m, 3H), 5.44 – 5.36 (m, 1H), 3.68 (d, J = 7.7 Hz, 3H), 3.22 – 3.00 (m, 1H), 2.93 –

2.80 (m, 1H), 2.78 – 2.53 (m, 2H), 2.48 (t, J = 7.1 Hz, 2H), 1.83 (s, 1H), 1.27 (d, J = 4.1 Hz, 6H). **^{13}C NMR** (126 MHz, $CDCl_3$) δ 193.02, 171.70, 165.75, 133.49, 130.31, 129.84 (d, J = 9.2 Hz), 128.64, 117.67, 73.46 (d, J = 5.1 Hz), 72.48, 52.15 (d, J = 9.6 Hz), 34.24 (t, J = 23.3 Hz), 33.30, 26.99, 25.87 (d, J = 2.6 Hz), 24.75, 24.18, 23.63. **^{19}F NMR** (470 MHz, $CDCl_3$) δ -99.54 (dt, J = 265.3, 14.9 Hz, 1F), -104.33 (dt, J = 265.4, 16.9 Hz, 1F). **HRMS** (ESI+) *m/z* calcd for $C_{18}H_{23}F_2O_6S$ [(M + H)$^+$], 405.1183, found, 405.1180.

6-(((3s,5s,7s)-adamantan-1-yl)thio)-5,5-difluoro-2-hydroxy-2-methyl-6-oxohexan-3-yl benzoate (3u)

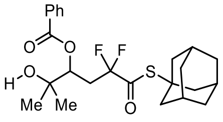

3u

Prepared according to General Procedure D, the title compound was obtained as a colorless oil (39.8 mg, 0.088 mmol, 44% yield). **R*f*** = 0.3 [Hexanes: EtOAc 3:1 (v/v)]. **^{1}H NMR** (500 MHz, $CDCl_3$) δ 1H NMR (500 MHz, $CDCl_3$) δ 8.08 – 8.02 (m, 2H), 7.57 (ddt, J = 7.8, 7.0, 1.3 Hz, 1H), 7.45 (ddt, J = 7.8, 6.5, 1.1 Hz, 2H), 5.42 (dd, J = 9.5, 2.3 Hz, 1H), 2.67 – 2.47 (m, 2H), 2.09 – 1.95 (m, 9H), 1.75 – 1.62 (m, 7H), 1.28 (d, J = 1.2 Hz, 6H). **^{13}C NMR** (126 MHz, $CDCl_3$) δ 192.60, 165.88, 133.31, 130.06, 128.53, 117.33 (t, J = 256.3 Hz), 73.57, 72.63, 52.63, 51.89, 47.49, 43.25, 41.47, 36.26 (d, J = 9.3 Hz), 33.98 (t, J = 23.0 Hz), 30.21, 29.97 (d, J = 14.2 Hz), 25.98, 25.78. **^{19}F NMR** (470 MHz, $CDCl_3$) δ -100.23 (dt, J = 263.5, 16.0 Hz, 1F), -103.49 (dt, J = 263.6, 16.9 Hz, 1F). **HRMS** (ESI+) *m/z* calcd for $C_{24}H_{31}F_2O_4S$ [(M + H)$^+$], 453.1911, found, 453.1915.

5,5-difluoro-2-hydroxy-2-methyl-6-oxo-6-(piperidin-1-yl)hexan-3-yl benzoate (3v)

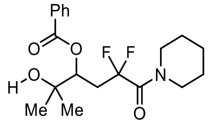

3v

Prepared according to General Procedure D, the title compound was obtained as a colorless oil (62.7 mg, 0.170 mmol, 85% yield). **R*f*** = 0.14 [Hexanes: EtOAc 3:1 (v/v)]. **^{1}H NMR** (500 MHz, $CDCl_3$) δ 8.09 – 8.02 (m, 2H), 7.60 – 7.52 (m, 1H), 7.48 – 7.41 (m, 2H), 5.53 (dd, J = 9.5, 1.5 Hz, 1H), 3.63 – 3.46 (m, 4H), 2.83 (dddd, J = 20.4, 19.0, 15.8, 1.5 Hz, 1H), 2.56 (dddd, J = 21.1, 15.8, 10.7, 9.4 Hz, 1H), 2.16 (s, 1H), 1.62 (q, J = 4.9 Hz, 1H), 1.70 – 1.47 (m, 6H), 1.30 (s, 6H). **^{13}C NMR** (126 MHz, $CDCl_3$) δ 165.96, 161.48, 133.22, 130.25, 129.88, 128.54, 73.99, 72.59, 46.96, 44.67, 35.25 (t, J = 22.8 Hz), 26.58, 25.99, 25.64 (d, J = 13.8 Hz), 24.54. **^{19}F NMR** (470 MHz, $CDCl_3$) δ -96.90 (ddd, J = 285.9, 20.6, 10.9 Hz, 1F), -100.34 (dt, J = 285.9, 20.0 Hz, 1F). **HRMS** (ESI+) *m/z* calcd for $C_{19}H_{26}F_2NO_4$ [(M + H)$^+$], 370.1829, found, 370.1831.

5,5-difluoro-2-hydroxy-2-methyl-6-morpholino-6-oxohexan-3-yl benzoate (3w)

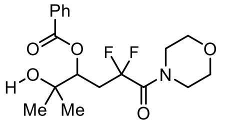

3w

Prepared according to General Procedure D, the title compound was obtained as a colorless oil (49.7 mg, 0.134 mmol, 67% yield). **R***f* = 0.08 [Hexanes: EtOAc 3:1 (v/v)]. **^{1}H NMR** (500 MHz, $CDCl_3$) δ 8.07 – 8.01 (m, 2H), 7.59 – 7.52 (m, 1H), 7.48 – 7.39 (m, 2H), 5.51 (dd, J = 9.7, 1.5 Hz, 1H), 3.73 – 3.54 (m, 8H), 2.82 (dddd, J = 20.3, 18.6, 15.7, 1.5 Hz, 1H), 2.57 (dddd, J = 21.0, 15.7, 11.1, 9.6 Hz, 1H), 2.37 (s, 1H), 1.29 (d, J = 2.1 Hz, 6H). **^{13}C NMR** (126 MHz, $CDCl_3$) δ 165.86, 161.68 (t, J = 29.1 Hz), 133.25, 129.82, 128.52, 123.97 – 113.10 (m), 73.75, 72.37, 66.74 (d, J = 13.0 Hz), 46.54, 43.54, 34.85 (t, J = 22.3 Hz), 26.05, 25.38. **^{19}F NMR** (470 MHz, $CDCl_3$) δ -96.81 (ddd, J = 287.2, 20.8, 11.2 Hz, 1F), -99.89 (dt, J = 287.6, 19.8 Hz, 1F). **HRMS** (ESI+) *m/z* calcd for $C_{18}H_{24}F_2NO_5$ [$(M + H)^+$], 372.1622, found, 372.1626.

5,5-difluoro-2-hydroxy-2-methyl-6-oxo-6-thiomorpholinohexan-3-yl benzoate (3x)

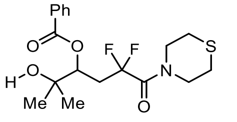

3x

Prepared according to General Procedure D, the title compound was obtained as a colorless oil (49.6 mg, 0.128 mmol, 64% yield). **R***f* = 0.1 [Hexanes: EtOAc 3:1 (v/v)]. **^{1}H NMR** (500 MHz, $CDCl_3$) δ 8.09 – 8.02 (m, 2H), 7.61 – 7.54 (m, 1H), 7.49 – 7.42 (m, 2H), 5.52 (dd, J = 9.7, 1.5 Hz, 1H), 3.97 – 3.77 (m, 4H), 2.82 (tdd, J = 19.7, 15.7, 1.5 Hz, 1H), 2.73 – 2.49 (m, 5H), 1.31 (d, J = 2.9 Hz, 6H). **^{13}C NMR** (126 MHz, $CDCl_3$) δ 165.89, 161.69 (t, J = 29.2 Hz), 133.27, 129.82, 128.55, 123.68 – 115.44 (m), 73.82 (d, J = 4.2 Hz), 72.43, 48.81, 46.23, 35.03 (t, J = 22.5 Hz), 28.14, 27.33, 26.05, 25.46. **^{19}F NMR** (470 MHz, $CDCl_3$) δ -96.64 (ddd, J = 287.4, 20.1, 11.0 Hz), -99.90 (dt, J = 287.1, 20.1 Hz). **HRMS** (ESI+) *m/z* calcd for $C_{18}H_{24}F_2NO_4S$ [$(M + H)^+$], 388.1394, found, 388.1398.

5,5-difluoro-2-hydroxy-2-methyl-6-oxo-6-thiomorpholinohexan-3-yl benzoate (3y)

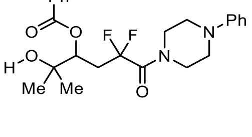

3y

Prepared according to General Procedure D, the title compound was obtained as a colorless oil (56.2 mg, 0.126 mmol, 63% yield). **R$_f$** = 0.08 [Hexanes: EtOAc 3:1 (v/v)]. **^{1}H NMR** (500 MHz, $CDCl_3$) δ 8.09 – 8.03 (m, 2H), 7.61 – 7.54 (m, 1H), 7.45 (ddd, J = 8.7, 5.3, 1.5 Hz, 2H), 7.32 – 7.22 (m, 2H), 6.98 – 6.85 (m, 3H), 5.55 (dd, J = 9.6, 1.5 Hz, 1H), 3.79 (dt, J = 32.8, 4.7 Hz, 4H), 3.25 – 3.07 (m, 4H), 2.85 (dddd, J = 20.3, 18.7, 15.8, 1.5 Hz, 1H), 2.61 (dddd, J = 20.8, 15.8, 10.9, 9.5 Hz, 1H), 2.12 – 1.98 (m, 1H), 1.32 (d, J = 2.5 Hz, 6H). **^{13}C NMR** (126 MHz, $CDCl_3$) δ 165.94, 161.69 (t, J = 32.3 Hz), 150.88, 133.31, 129.89, 129.39, 128.59, 120.81, 116.80, 73.86, 72.58, 49.89, 49.40, 45.80, 43.28, 35.02 (t, J = 22.5 Hz), 26.01, 25.64. **^{19}F NMR** (470 MHz, $CDCl_3$) δ -96.54 (ddd, J = 287.0, 20.2, 10.9 Hz, 1F), -99.72 (dt, J = 287.2, 19.9 Hz, 1F). **HRMS** (ESI+) *m/z* calcd for $C_{24}H_{28}F_2N_2O_4Na$ [M + Na)$^+$], 469.1914, found, 469.1917.

5,5-difluoro-2-hydroxy-2-methyl-6-oxo-6-(1,4-dioxa-8-azaspiro[4.5]decan-8-yl)hexan-3-yl benzoate (3z)

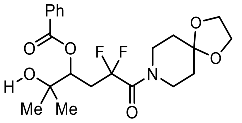

3z

Prepared according to General Procedure D, the title compound was obtained as a colorless oil (53.0 mg, 0.124 mmol, 62% yield). **R$_f$** = 0.07 [Hexanes: EtOAc 3:1 (v/v)]. **^{1}H NMR** (500 MHz, $CDCl_3$) δ 8.08 – 8.01 (m, 2H), 7.60 – 7.53 (m, 1H), 7.49 – 7.41 (m, 2H), 5.53 (dd, J = 9.5, 1.4 Hz, 1H), 4.01 – 3.91 (m, 4H), 3.69 (dt, J = 17.7, 5.9 Hz, 4H), 2.82 (dddd, J = 20.3, 18.6, 15.7, 1.5 Hz, 1H), 2.57 (dddd, J = 21.3, 15.8, 10.6, 9.5 Hz, 1H), 2.04 (s, 1H), 1.77 – 1.61 (m, 4H), 1.30 (d, J = 1.6 Hz, 6H). **^{13}C NMR** (126 MHz, $CDCl_3$) δ 165.96, 161.60, 133.26, 130.17, 129.88, 128.56, 106.74, 73.91, 72.58, 64.63, 43.72, 41.83, 35.75, 35.46 – 34.41 (m), 25.98, 25.62. **^{19}F NMR** (470 MHz, $CDCl_3$) δ -96.79 (ddd, J = 286.8, 20.7, 10.6 Hz, 1F), -100.04 (dt, J = 286.4, 19.9 Hz, 1F). **HRMS** (ESI+) *m/z* calcd for $C_{21}H_{27}F_2NO_6Na$ [M + Na)$^+$], 450.1704, found, 450.1707.

6-(diethylamino)-5,5-difluoro-2-hydroxy-2-methyl-6-oxohexan-3-yl benzoate (3aa)

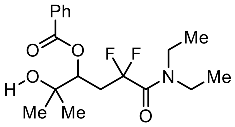

3aa

Prepared according to General Procedure D, the title compound was obtained as a colorless oil (49.3 mg, 0.138 mmol, 69% yield). **R$_f$** = 0.28 [Hexanes: EtOAc 3:1 (v/v)]. **^{1}H NMR** (500 MHz, $CDCl_3$) δ 1H NMR (500 MHz, $CDCl_3$) δ 8.09 – 8.01 (m, 2H), 7.60 – 7.50 (m, 1H), 7.49 – 7.41 (m, 2H), 5.55 – 5.49 (m, 1H),

3.56 – 3.25 (m, 4H), 2.82 (tdd, J = 19.4, 15.7, 1.6 Hz, 1H), 2.57 (dddd, J = 20.4, 15.6, 10.8, 9.5 Hz, 1H), 2.09 (s, 1H), 1.30 (s, 6H), 1.12 (dt, J = 9.0, 7.1 Hz, 6H). **^{13}C NMR** (126 MHz, $CDCl_3$) δ 165.95, 162.52 (t, J = 32.3 Hz), 133.21, 130.25, 129.87, 128.53, 119.37(t, J = 252.1 Hz), 74.00, 72.57, 41.82 (d, J = 26.1 Hz), 35.18 (t, J = 22.7 Hz), 25.98, 25.58, 14.27, 12.37. **^{19}F NMR** (470 MHz, $CDCl_3$) δ -97.37 (ddd, J = 285.5, 19.8, 10.8 Hz, 1F), -100.64 (dt, J = 285.6, 19.9 Hz, 1F). **HRMS** (ESI+) *m/z* calcd for $C_{18}H_{25}F_2NO_4Na$ [M + Na)$^+$], 380.1649, found, 380.1651.

6-(((3s,5s,7s)-adamantan-1-yl)amino)-5,5-difluoro-2-hydroxy-2-methyl-6-oxohexan-3-yl benzoate (3ab)

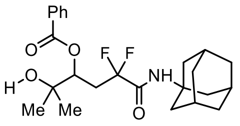

3ab

Prepared according to General Procedure D, the title compound was obtained as a colorless oil (52.3 mg, 0.12 mmol, 60% yield). **R_f** = 0.28 [Hexanes: EtOAc 3:1 (v/v)]. **^{1}H NMR** (500 MHz, $CDCl_3$) δ 8.11 – 8.03 (m, 2H), 7.58 (ddt, J = 7.8, 7.0, 1.3 Hz, 1H), 7.51 – 7.43 (m, 2H), 6.02 (s, 1H), 5.36 (dd, J = 10.1, 1.6 Hz, 1H), 2.86 – 2.41 (m, 2H), 2.04 (h, J = 3.3 Hz, 3H), 2.00 – 1.89 (m, 6H), 1.80 (s, 1H), 1.65 (t, J = 3.1 Hz, 6H), 1.28 (s, 6H). **^{13}C NMR** (126 MHz, $CDCl_3$) δ 166.08, 162.45, 133.44, 130.00, 128.62, 74.01, 72.49, 52.77, 41.07, 36.26, 34.01 (t, J = 23.4 Hz), 29.46, 25.81 (d, J = 7.4 Hz). **^{19}F NMR** (470 MHz, $CDCl_3$) δ -102.07 (dt, J = 257.7, 13.7 Hz, 1F), -104.79 (ddd, J = 258.2, 22.2, 15.0 Hz, 1F). **HRMS** (ESI+) *m/z* calcd for $C_{24}H_{32}F_2NO_4$ [(M + H)$^+$], 436.2299, found, 436.2305.

6-(tert-butylamino)-5,5-difluoro-2-hydroxy-2-methyl-6-oxohexan-3-yl benzoate (3ac)

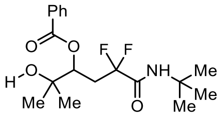

3ac

Prepared according to General Procedure D, the title compound was obtained as a yellow oil (39.3 mg, 0.110 mmol, 55% yield). **R_f** = 0.29 [Hexanes: EtOAc 3:1 (v/v)]. **^{1}H NMR** (500 MHz, $CDCl_3$) δ 8.09 – 8.02 (m, 2H), 7.58 (ddt, J = 7.9, 7.0, 1.3 Hz, 1H), 7.48 – 7.30 (m, 2H), 6.19 (s, 1H), 5.38 – 5.32 (m, 1H), 2.70 (dtd, J = 21.6, 15.3, 1.5 Hz, 1H), 2.53 (tdd, J = 15.6, 12.3, 10.1 Hz, 1H), 1.95 (s, 1H), 1.33 (s, 9H), 1.28 (s, 6H). **^{13}C NMR** (126 MHz, $CDCl_3$) δ 166.09, 162.83 (t, J = 27.0 Hz), 133.43, 129.95, 128.62, 121.75 – 111.18 (m), 73.94 (d, J = 5.4 Hz), 72.42, 52.13, 33.98 (t, J = 23.5 Hz), 28.35, 25.77 (d, J = 14.6 Hz). **^{19}F NMR** (470 MHz, $CDCl_3$) δ -102.08 (dt, J = 258.2, 13.7 Hz, 1F), -103.94 – -106.58 (m, 1F). **HRMS** (ESI+) *m/z* calcd for $C_{18}H_{26}F_2NO_4$ [(M + H)$^+$], 358.1829, found, 358.1835.

5,5-difluoro-2-hydroxy-2-methyl-6-oxo-6-(phenethylamino)hexan-3-yl benzoate (3ad)

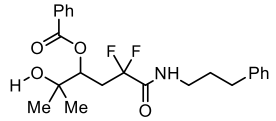

3ad

Prepared according to General Procedure D, the title compound was obtained as a colorless oil (45.3 mg, 0.108 mmol, 54% yield). $\mathbf{R}_f$ = 0.19 [Hexanes: EtOAc 3:1 (v/v)]. ^{1}**H NMR** (500 MHz, $CDCl_3$) δ 1H NMR (500 MHz, $CDCl_3$) δ 7.58 (ddt, J = 7.9, 7.1, 1.3 Hz, 1H), 7.48 – 7.40 (m, 2H), 7.30 – 7.23 (m, 2H), 7.20 – 7.15 (m, 1H), 7.14 – 7.08 (m, 2H), 6.36 (t, J = 6.0 Hz, 1H), 5.39 – 5.32 (m, 1H), 3.26 – 3.16 (m, 1H), 2.95 (ddt, J = 13.6, 7.9, 6.0 Hz, 1H), 2.75 – 2.63 (m, 2H), 2.57 – 2.49 (m, 2H), 1.82 – 1.65 (m, 2H), 1.27 (d, J = 1.5 Hz, 6H). ^{13}**C NMR** (126 MHz, $CDCl_3$) δ 166.10, 163.73 (t, J = 28.0 Hz), 141.03, 133.53, 129.93, 129.67, 128.63 (d, J = 3.1 Hz), 128.44, 126.22, 73.89, 72.33, 39.26, 34.05 (t, J = 23.6 Hz), 33.15, 30.54, 25.85, 25.68. ^{19}**F NMR** (470 MHz, $CDCl_3$) δ -103.22 (ddd, J = 260.1, 18.6, 13.4 Hz, 1F), -104.85 (dt, J = 260.1, 15.4 Hz, 1F). **HRMS** (ESI+) *m/z* calcd for $C_{23}H_{28}F_2NO_4$ [$(M + H)^+$], 420.1986, found, 420.1992.

5,5-difluoro-2-hydroxy-2-methyl-6-oxo-6-(phenylamino)hexan-3-yl benzoate (3ae)

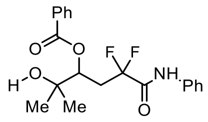

3ae

Prepared according to General Procedure D, the title compound was obtained as a colorless oil (24.1 mg, 0.064 mmol, 32% yield). $\mathbf{R}_f$ = 0.19 [Hexanes: EtOAc 3:1 (v/v)]. ^{1}**H NMR** (500 MHz, $CDCl_3$) δ 8.04 (s, 1H), 7.99 – 7.92 (m, 2H), 7.50 (ddt, J = 7.7, 7.1, 1.3 Hz, 1H), 7.45 – 7.36 (m, 2H), 7.36 – 7.31 (m, 2H), 7.29 – 7.18 (m, 2H), 7.13 – 7.06 (m, 1H), 5.44 – 5.38 (m, 1H), 2.87 – 2.67 (m, 2H), 1.85 (s, 1H), 1.29 (d, J = 2.5 Hz, 6H).^{13}**C NMR** (126 MHz, $CDCl_3$) δ 166.33, 161.58, 136.00, 133.49, 129.92, 129.29, 129.08, 128.54, 125.48, 120.24, 117.65 (t, J = 251.6 Hz), 73.87, 72.37, 34.32, 34.13, 33.94, 29.84, 25.93, 25.69. ^{19}**F NMR** (470 MHz, $CDCl_3$) δ -102.80 (ddd, J = 259.7, 19.5, 12.6 Hz, 1F), -104.34 (dt, J = 259.3, 15.3 Hz, 1F). **HRMS** (ESI+) *m/z* calcd for $C_{20}H_{22}F_2NO_4$ [$(M + H)^+$], 378.1516, found, 378.1520.

5,5,6,6,7,7,8,8,9,9,10,10,10-tridecafluoro-2-hydroxy-2-methyldecan-3-yl benzoate (3af)

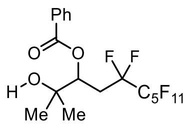

3af

Prepared according to General Procedure D, the title compound was obtained as a colorless oil (77.9 mg, 0.148 mmol, 74% yield). **R*f*** = 0.36 [Hexanes: EtOAc 3:1 (v/v)]. **^{1}H NMR** (500 MHz, $CDCl_3$) δ 8.10 – 8.02 (m, 2H), 7.59 (ddt, J = 7.8, 7.0, 1.3 Hz, 1H), 7.50 – 7.41 (m, 2H), 5.59 (dd, J = 9.7, 1.7 Hz, 1H), 2.75 – 2.49 (m, 2H), 1.98 (s, 1H), 1.31 (d, J = 3.2 Hz, 6H). **^{13}C NMR** (126 MHz, $CDCl_3$) δ 165.70, 133.55, 129.93, 128.66, 118.18, 72.39 (d, J = 12.5 Hz), 31.09 (t, J = 20.9 Hz), 26.30, 25.42. **^{19}F NMR** (470 MHz, $CDCl_3$) δ -80.90 (s, 3F), -113.72 – -114.09 (m, 2F), -121.71 – -121.89 (m, 2F), -122.90 (dp, J = 23.7, 7.8 Hz, 2F), -123.45 (dt, J = 19.2, 10.1 Hz, 2F), -126.20 (tt, J = 13.7, 6.1 Hz, 2F). **HRMS** (ESI+) *m/z* calcd for $C_{18}H_{16}F_{13}O_3$ [$(M + H)^+$], 527.0891, found, 527.0896.

5,5,6,6,7,7,8,8,9,9,10,10,11,11,12,12,12-heptadecafluoro-2-hydroxy-2-methyldodecan-3-yl benzoate (3ag)

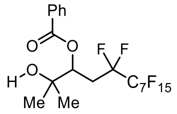

3ag

Prepared according to General Procedure D, the title compound was obtained as a colorless oil (86.4 mg, 0.15 mmol, 75% yield). **R*f*** = 0.36 [Hexanes: EtOAc 3:1 (v/v)]. **^{1}H NMR** (500 MHz, $CDCl_3$) δ 8.10 – 8.03 (m, 2H), 7.58 (ddt, J = 7.8, 7.0, 1.3 Hz, 1H), 7.49 – 7.41 (m, 2H), 5.60 (dd, J = 9.6, 1.6 Hz, 1H), 2.75 – 2.49 (m, 2H), 2.03 (s, 1H), 1.31 (d, J = 3.0 Hz, 7H). **^{13}C NMR** (126 MHz, $CDCl_3$) δ 165.73, 133.54, 129.94, 129.72, 128.66, 119.83 – 115.15 (m), 109.66 (dd, J = 273.9, 33.8 Hz), 72.39 (d, J = 9.6 Hz), 31.10 (t, J = 20.9 Hz), 26.29, 25.38. **^{19}F NMR** (470 MHz, $CDCl_3$) δ -80.93 (t, J = 10.0 Hz, 3F), -113.92 (ddq, J = 65.4, 26.9, 13.5 Hz, 2F), -121.62 (t, J = 16.1 Hz, 2F), -121.99 (tt, J = 21.7, 11.7 Hz, 4F), -122.80 (dq, J = 23.1, 10.8 Hz, 2F), -123.43 (q, J = 11.5 Hz, 2F), -125.86 – -126.77 (m, 2F).

5,6,6,6-tetrafluoro-2-hydroxy-2-methyl-5-(trifluoromethyl)hexan-3-yl benzoate (3ah)

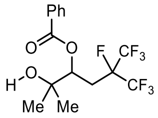

3ah

Prepared according to General Procedure D, the title compound was obtained as a colorless oil (38.3 mg, 0.102 mmol, 51% yield). **R*f*** = 0.36 [Hexanes: EtOAc 3:1 (v/v)]. **^{1}H NMR** (500 MHz, $CDCl_3$) δ 8.07 – 8.01 (m, 2H), 7.63 – 7.55 (m, 1H), 7.51 – 7.43 (m, 2H), 5.53 – 5.47 (m, 1H), 2.75 – 2.56 (m, 2H), 1.80 (d, J = 4.3 Hz, 1H), 1.30 (d, J = 6.5 Hz, 6H). **^{13}C NMR** (126 MHz, $CDCl_3$) δ 165.75, 133.54, 129.89, 128.66, 124.04 – 115.50 (m), 91.98, 72.92, 26.16, 25.67. **^{19}F NMR** (470 MHz, $CDCl_3$) δ -76.64 (p, J = 9.2 Hz, 3F), -77.49 (p, J = 9.1 Hz, 3F), -186.90 (dtp, J = 21.0, 14.1, 7.2 Hz, 1F).

1,1-difluoro-4-hydroxy-4-methyl-1-(perfluorophenyl)pentan-3-yl benzoate (3ai)

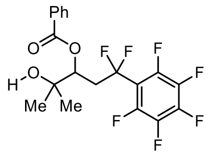

3ai

Prepared according to General Procedure D, the title compound was obtained as a white solid (61.0 mg, 0.105 mmol, 53% yield). **R*f*** = 0.36 [Hexanes: EtOAc 3:1 (v/v)]. **^{1}H NMR** (500 MHz, $CDCl_3$) δ 7.90 – 7.82 (m, 2H), 7.58 (ddt, J = 8.7, 7.1, 1.3 Hz, 1H), 7.47 – 7.39 (m, 2H), 5.38 – 5.32 (m, 1H), 2.89 – 2.77 (m, 2H), 1.81 (s, 1H), 1.29 (d, J = 6.4 Hz, 6H). **^{13}C NMR** (126 MHz, $CDCl_3$) δ 165.51, 145.68, 143.58, 141.06, 138.84, 136.91, 133.74, 129.40, 128.63, 119.92, 74.06 (d, J = 6.9 Hz), 72.46, 38.67 (t, J = 26.3 Hz), 25.79. **^{19}F NMR** (470 MHz, $CDCl_3$) δ -79.91 – -83.87 (m, 1F), -92.98 (dddd, J = 269.2, 35.4, 20.2, 15.4 Hz, 1F), -139.99 (dtq, J = 30.9, 15.2, 5.3 Hz, 2F), -150.67 (t, J = 21.0 Hz, 1F), -160.34 – -160.95 (m, 2F). **HRMS** (ESI+) *m/z* calcd for $C_{19}H_{16}F_7O_3$ $[(M + H)^+]$, 425.0987, found, 425.0989.

1,1-difluoro-4-hydroxy-4-methyl-1-(phenylsulfonyl)pentan-3-yl benzoate (3aj)

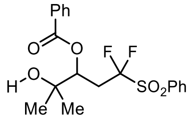

3aj

Prepared according to General Procedure D, the title compound was obtained as a colorless oil (72.5 mg, 0.182 mmol, 91% yield). **R*f*** = 0.26 [Hexanes: EtOAc 3:1 (v/v)]. **^{1}H NMR** (500 MHz, $CDCl_3$) δ 8.07 – 7.96 (m, 2H), 7.96 – 7.91 (m, 2H), 7.71 (ddt, J = 8.7, 7.3, 1.3 Hz, 1H), 7.60 – 7.52 (m, 3H), 5.58 (dd, J = 10.0, 1.6 Hz, 1H), 2.99 (dddd, J = 23.1, 16.4, 14.4, 1.6 Hz, 1H), 2.92 – 2.77 (m, 1H), 2.05 (d, J = 10.5 Hz, 1H), 1.31 (d, J = 1.3 Hz, 6H). **^{13}C NMR** (126 MHz, $CDCl_3$) δ 171.35, 165.63, 135.55, 133.43, 131.79, 130.97, 129.92, 129.63, 129.40, 128.56, 124.11 (t, J = 287.6 Hz), 72.78, 72.36, 60.53, 29.11 (t, J = 19.1 Hz), 26.15, 25.41, 21.13, 14.27. **^{19}F NMR** (470 MHz, $CDCl_3$) δ -103.17 – -103.60 (m, 2F). **HRMS** (ESI+) *m/z* calcd for $C_{19}H_{21}F_2O_5S$ $[(M + H)^+]$, 399.1077, found, 399.1084.

1-cyano-4-hydroxy-4-methylpentan-3-yl benzoate (3ak)

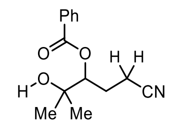

3ak

Prepared according to General Procedure D, the title compound was obtained as a colorless oil (37.1 mg, 0.150 mmol, 75% yield). $\mathbf{R}_f$ = 0.15 [Hexanes: EtOAc 3:1 (v/v)]. **^{1}H NMR** (500 MHz, $CDCl_3$) δ 8.09 – 8.02 (m, 2H), 7.64 – 7.56 (m, 1H), 7.52 – 7.42 (m, 2H), 5.11 (dd, J = 10.0, 2.8 Hz, 1H), 2.44 (dd, J = 8.0, 7.1 Hz, 2H), 2.28 – 2.18 (m, 1H), 2.11 (ddt, J = 14.4, 10.0, 7.2 Hz, 1H), 1.88 – 1.78 (m, 1H), 1.30 (s, 6H). **^{13}C NMR** (126 MHz, $CDCl_3$) δ 166.48, 133.69, 129.92, 129.45, 128.74, 119.34, 78.58, 72.44, 28.47 – 20.54 (m), 14.60. **HRMS** (ESI+) *m/z* calcd for $C_{14}H_{18}NO_3$ [(M + H)$^+$], 248.1286, found, 248.1287.

6-ethoxy-5,5-difluoro-2-hydroxy-2-methyl-6-oxohexan-3-yl 4-(tert-butyl)benzoate (3al)

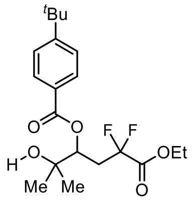

3al

Prepared according to General Procedure D, the title compound was obtained as a yellow oil (61.8 mg, 0.16 mmol, 80% yield). $\mathbf{R}_f$ = 0.36 [Hexanes: EtOAc 3:1 (v/v)]. **^{1}H NMR** (500 MHz, $CDCl_3$) δ 8.08 – 7.90 (m, 2H), 7.57 – 7.33 (m, 2H), 5.52 – 5.25 (m, 1H), 4.22 – 3.95 (m, 2H), 2.82 – 2.43 (m, 2H), 1.81 (s, 1H), 1.34 (s, 9H), 1.28 (m, 6H), 1.20 (t, J = 7.2 Hz, 3H). **^{13}C NMR** δ 165.77, 163.97 (t, J = 32.4 Hz), 157.28, 129.81, 126.94, 125.59, 120.36 – 110.21 (m), 73.48, 72.51, 63.16, 34.90 (t, J = 23.5 Hz), 31.23, 25.88 (d, J = 6.3 Hz), 13. 83. **^{19}F NMR** (470 MHz, $CDCl_3$) δ -102.68 (dt, J = 267.2, 15.1 Hz), -105.32 (dt, J = 267.4, 16.6 Hz). **HRMS** (ESI+) *m/z* calcd for $C_{20}H_{29}F_2O_5$ [(M + H)$^+$], 387.1983, found, 387.1993.

6-ethoxy-5,5-difluoro-2-hydroxy-2-methyl-6-oxohexan-3-yl 4-methoxybenzoate (3am)

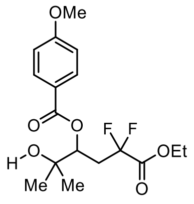

3am

Prepared according to General Procedure D, the title compound was obtained as a colorless oil (46.8 mg, 0.13 mmol, 65% yield). **R*f*** = 0.18 [Hexanes: EtOAc 3:1 (v/v)]. **^{1}H NMR** (500 MHz, $CDCl_3$) δ 7.98 (d, J = 8.9 Hz, 1H), 6.92 (d, J = 8.9 Hz, 1H), 5.39 – 5.33 (m, 1H), 4.32 – 3.97 (m, 2H), 3.86 (s, 3H), 2.71 – 2.52 (m, 2H), 1.89 (s, 2H), 1.27 (d, J = 2.7 Hz, 6H), 1.21 (t, J = 7.2 Hz, 3H). **^{13}C NMR** (126 MHz, $CDCl_3$) δ 13C NMR (126 MHz, $CDCl_3$) δ 165.49, 164.69 – 161.49 (m), 131.97, 122.05, 115.47 (t, J = 252.1 Hz), 113.86, 73.35, 72.49, 63.14, 55.60, 34.94 (t, J = 23.4 Hz), 25.85 (d, J = 11.1 Hz), 13.84. **^{19}F NMR** (470 MHz, $CDCl_3$) δ -102.42 (dt, J = 266.7, 14.6 Hz, 1F), -105.55 (dt, J = 267.0, 16.9 Hz, 1F). **HRMS** (ESI+) *m/z* calcd for $C_{17}H_{23}F_2O_6$ [$(M + H)^+$], 361.1462, found, 361.1468.

6-ethoxy-5,5-difluoro-2-hydroxy-2-methyl-6-oxohexan-3-yl 3-methylbenzoate (3an)

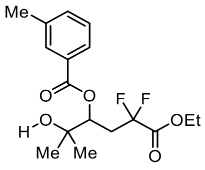

3an

Prepared according to General Procedure D, the title compound was obtained as a colorless oil (42.7 mg, 0.124 mmol, 62% yield). **R*f*** = 0.16 [Hexanes: EtOAc 3:1 (v/v)]. **^{1}H NMR** δ 7.86 – 7.80 (m, 2H), 7.44 – 7.37 (m, 1H), 7.34 (t, J = 7.5 Hz, 1H), 5.40 (dd, J = 9.7, 2.4 Hz, 1H), 4.10 (ddq, J = 38.6, 10.7, 7.1 Hz, 2H), 2.74 – 2.52 (m, 2H), 2.41 (s, 3H), 1.28 (d, J = 4.0 Hz, 6H), 1.22 (t, J = 7.1 Hz, 3H). **^{13}C NMR** (126 MHz, $CDCl_3$) δ 165.94, 163.93 (t, J = 32.1 Hz), 138.44, 134.26, 130.40, 129.66, 128.49, 127.03, 115.45 (t, J = 251.8 Hz), 73.59, 72.51, 63.15, 34.89 (t, J = 23.4 Hz), 25.90 (d, J = 11.5 Hz), 21.40, 13.86. **^{19}F NMR** (470 MHz, $CDCl_3$) δ -102.71 (dt, J = 267.1, 14.9 Hz, 1F), -105.37 (dt, J = 267.2, 16.7 Hz, 1F).

6-ethoxy-5,5-difluoro-2-hydroxy-2-methyl-6-oxohexan-3-yl 4-chlorobenzoate (3ao)

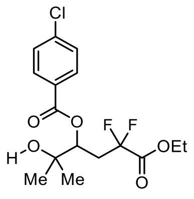

3ao

Prepared according to General Procedure D, the title compound was obtained as a white solid (57.6 mg, 0.158 mmol, 79% yield). **R*f*** = 0.32 [Hexanes: EtOAc 3:1 (v/v)]. **^{1}H NMR** (500 MHz, $CDCl_3$) δ 8.00 – 7.92 (m, 2H), 7.45 – 7.38 (m, 2H), 5.39 (dd, J = 8.2, 3.9 Hz, 1H), 4.21 – 4.05 (m, 2H), 2.68 – 2.56 (m, 2H), 1.95 (s, 1H), 1.27 (d, J = 2.7 Hz, 6H), 1.24 (t, J = 7.2 Hz, 3H). **^{13}C NMR** (126 MHz, $CDCl_3$) δ 164.95, 163.87 (t, J = 32.3 Hz), 139.97, 131.25, 128.96, 128.20, 120.53 – 107.99 (m), 73.90 (t, J = 4.2 Hz), 72.38, 63.20,

34.78 (t, J = 23.4 Hz), 25.81 (d, J = 2.3 Hz), 13.86. **^{19}F NMR** (470 MHz, $CDCl_3$) δ -103.15 (dt, J = 267.3, 15.4 Hz), -105.19 (dt, J = 267.4, 16.6 Hz). **HRMS** (ESI+) *m/z* calcd for $C_{16}H_{20}ClF_2O_5$ $[(M + H)^+]$, 365.0967, found, 365.0976.

6-ethoxy-5,5-difluoro-2-hydroxy-2-methyl-6-oxohexan-3-yl 4-(trifluoromethyl)benzoate (3ap)

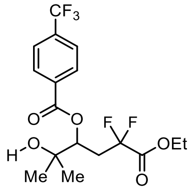

3ap

Prepared according to General Procedure D, the title compound was obtained as a colorless oil (44.6 mg, 0.112 mmol, 56% yield). **R_f** = 0.34 [Hexanes: EtOAc 3:1 (v/v)]. **^{1}H NMR** (500 MHz, $CDCl_3$) δ 8.15 (dp, J = 7.7, 0.8 Hz, 2H), 7.71 (dq, J = 7.5, 0.8 Hz, 2H), 5.44 (dd, J = 7.0, 5.1 Hz, 1H), 4.23 – 4.08 (m, 2H), 2.69 – 2.58 (m, 2H), 1.88 (s, 1H), 1.29 (d, J = 2.4 Hz, 6H), 1.25 (t, J = 7.2 Hz, 3H). **^{13}C NMR** (126 MHz, $CDCl_3$) δ 164.66, 163.85 (t, J = 32.2 Hz), 135.58 – 134.26 (m), 133.05, 130.29, 125.65 (d, J = 3.7 Hz), 124.77, 115.37 (t, J = 252.1 Hz), 74.24 (t, J = 3.9 Hz), 72.38, 63.25, 34.75 (t, J = 23.2 Hz), 25.83 (d, J = 3.2 Hz), 13.88. **^{19}F NMR** (470 MHz, $CDCl_3$) δ -63.21(s, 3F), -103.59 (dt, J = 267.9, 15.8 Hz, 1F), -104.97 (dt, J = 268.0, 16.2 Hz, 1F). **HRMS** (ESI+) *m/z* calcd for $C_{17}H_{20}F_5O_5$ $[(M + H)^+]$, 399.1230, found, 399.1234.

6-ethoxy-5,5-difluoro-2-hydroxy-2-methyl-6-oxohexan-3-yl 3-(trifluoromethyl)benzoate (3aq)

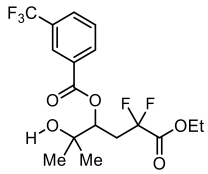

3aq

Prepared according to General Procedure D, the title compound was obtained as a colorless oil (45.4 mg, 0.114 mmol, 57% yield). **R_f** = 0.26 [Hexanes: EtOAc 3:1 (v/v)]. **^{1}H NMR** (500 MHz, $CDCl_3$) δ 1H NMR (500 MHz, $CDCl_3$) δ 8.29 – 8.20 (m, 2H), 7.87 – 7.82 (m, 1H), 7.61 (t, J = 7.8 Hz, 1H), 5.45 (dd, J = 8.8, 3.3 Hz, 1H), 4.16 (qq, J = 10.8, 7.2 Hz, 2H), 2.72 – 2.57 (m, 2H), 1.64 – 1.55 (m, 1H), 1.30 (d, J = 2.6 Hz, 6H), 1.27 – 1.24 (m, 3H). **^{13}C NMR** (126 MHz, $CDCl_3$) δ 164.57, 163.83 (t, J = 32.2 Hz), 133.13, 130.70, 130.01, 129.37, 126.71, 115.36 (t, J = 252.1 Hz), 74.28, 72.45, 63.25, 34.77 (t, J = 23.3 Hz), 29.84, 25.91 (d, J = 14.8 Hz), 13.91. **^{19}F NMR** (470 MHz, $CDCl_3$) δ -62.82 (s, 3F), -102.15 – -104.19 (m, 1F), -105.02 (dt, J = 268.3, 16.2 Hz, 1F).

6-ethoxy-5,5-difluoro-2-hydroxy-2-methyl-6-oxohexan-3-yl 2-(trifluoromethyl)benzoate (3ar)

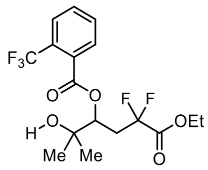

3ar

Prepared according to General Procedure D, the title compound was obtained as a colorless oil (42.2 mg, 0.106 mmol, 53% yield). **R$_f$** = 0.28 [Hexanes: EtOAc 3:1 (v/v)]. **^{1}H NMR** (500 MHz, $CDCl_3$) δ 7.91 – 7.85 (m, 1H), 7.81 – 7.73 (m, 1H), 7.68 – 7.58 (m, 2H), 5.43 (dd, J = 9.5, 2.0 Hz, 1H), 4.24 (q, J = 7.2 Hz, 2H), 2.73 – 2.48 (m, 2H), 1.78 – 1.72 (m, 1H), 1.38 – 1.24 (m, 9H). **^{13}C NMR** (126 MHz, $CDCl_3$) δ 165.56, 163.79 (t, J = 32.2 Hz), 132.00, 131.65, 130.98, 129.56, 128.63, 126.89 (d, J = 5.6 Hz), 125.84 – 112.63 (m), 74.82, 72.37, 63.26, 34.70 (t, J = 23.1 Hz), 25.72, 13.95. **^{19}F NMR** (470 MHz, $CDCl_3$) δ -58.91 (s, 3F), -103.37 – -105.50 (m, 2F). **HRMS** (ESI+) *m/z* calcd for $C_{17}H_{19}F_5O_5Na$ [$(M + Na)^+$], 421.1050, found, 421.1052.

6-ethoxy-5,5-difluoro-2-hydroxy-2-methyl-6-oxohexan-3-yl 2-(trifluoromethoxy)benzoate (3as)

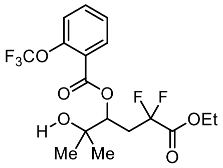

3as

Prepared according to General Procedure D, the title compound was obtained as a colorless oil (46.1 mg, 0.112 mmol, 56% yield). **R$_f$** = 0.14 [Hexanes: EtOAc 3:1 (v/v)]. **^{1}H NMR** (500 MHz, $CDCl_3$) δ 7.97 (dd, J = 7.8, 1.8 Hz, 1H), 7.57 (ddd, J = 8.3, 7.5, 1.8 Hz, 1H), 7.42 – 7.31 (m, 2H), 5.45 (dd, J = 9.4, 2.2 Hz, 1H), 4.20 (q, J = 7.1 Hz, 2H), 2.70 – 2.49 (m, 2H), 1.82 (s, 1H), 1.33 – 1.23 (m, 9H). **^{13}C NMR** (126 MHz, $CDCl_3$) δ 163.86, 163.83 (t, J = 32.2 Hz), 147.86, 133.91, 132.55, 127.09, 124.47, 122.03, 121.91 – 111.34 (m), 74.34, 72.29, 63.16, 34.71 (t, J = 23.2 Hz), 25.74, 13.88. **^{19}F NMR** (470 MHz, $CDCl_3$) δ -56.76 (s, 3F), -104.37 (t, J = 16.1 Hz, 2F).

6-ethoxy-5,5-difluoro-2-hydroxy-2-methyl-6-oxohexan-3-yl thiophene-2-carboxylate (3at)

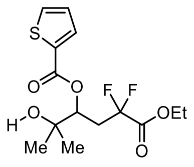

3at

Prepared according to General Procedure D, the title compound was obtained as a faint yellow oil (38.3 mg, 0.114 mmol, 57% yield). $\mathbf{R}_f$ = 0.22 [Hexanes: EtOAc 3:1 (v/v)]. **^{1}H NMR** (500 MHz, $CDCl_3$) δ 7.83 (dd, J = 3.8, 1.3 Hz, 1H), 7.59 (dd, J = 5.0, 1.3 Hz, 1H), 7.12 (dd, J = 5.0, 3.8 Hz, 1H), 5.40 – 5.30 (m, 1H), 4.30 – 4.08 (m, 2H), 2.71 – 2.52 (m, 2H), 1.28 (s, 6H), 1.28 – 1.24 (m, 3H). **^{13}C NMR** (126 MHz, $CDCl_3$) δ 163.89, 161.34, 134.16, 133.10, 128.07, 115.38 (t, J = 252.1 Hz), 73.90, 72.47, 63.22, 34.85 (t, J = 23.4 Hz), 25.83 (d, J = 10.4 Hz), 13.92. **^{19}F NMR** (470 MHz, $CDCl_3$) δ -102.83 (dt, J = 267.2, 15.1 Hz, 1F), -105.35 (dt, J = 267.2, 16.6 Hz, 1F). **HRMS** (ESI+) *m/z* calcd for $C_{14}H_{19}F_5O_5S$ [$(M + H)^+$], 337.0921, found, 337.0927.

6-ethoxy-5,5-difluoro-2-hydroxy-2-methyl-6-oxohexan-3-yl cyclohexanecarboxylate (3au)

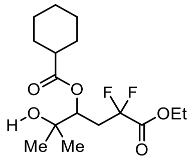

3au

Prepared according to General Procedure D, the title compound was obtained as a colorless oil (50.4 mg, 0.15 mmol, 75% yield). $\mathbf{R}_f$ = 0.16 [Hexanes: EtOAc 3:1 (v/v)]. **^{1}H NMR** (500 MHz, $CDCl_3$) δ 5.14 (dt, J = 9.9, 1.7 Hz, 1H), 4.31 (qd, J = 7.2, 1.9 Hz, 2H), 2.58 – 2.46 (m, 1H), 2.45 – 2.35 (m, 1H), 2.29 (tdd, J = 11.4, 4.7, 2.6 Hz, 1H), 1.94 – 1.87 (m, 2H), 1.81 – 1.69 (m, 3H), 1.67 – 1.58 (m, 1H), 1.50 – 1.38 (m, 2H), 1.31 – 1.22 (m, 3H), 1.20 (dd, J = 5.6, 2.0 Hz, 6H). **^{13}C NMR** (126 MHz, $CDCl_3$) δ 175.20, 163.88 (t, J = 32.5 Hz), 115.50 (t, J = 251.6 Hz), 72.36 (dd, J = 8.4, 4.5 Hz), 63.14, 43.39, 34.57 (t, J = 22.9 Hz), 29.26, 28.75, 26.08 – 25.60 (m), 25.46, 14.04. **^{19}F NMR** (470 MHz, $CDCl_3$) δ -103.09 – -104.07 (m, 1F), -104.62 (dt, J = 268.8, 17.0 Hz, 1F). **HRMS** (ESI+) *m/z* calcd for $C_{16}H_{27}F_2O_5$ [$(M + H)^+$], 337.1826, found, 337.1832.

6-ethoxy-5,5-difluoro-2-hydroxy-2-methyl-6-oxohexan-3-yl 2-(3-cyano-4-isobutoxyphenyl)-4-methylthiazole-5-carboxylate (3av)

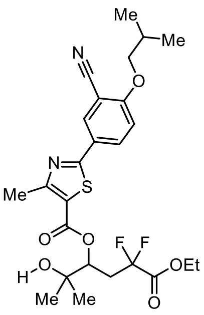

3av

Prepared according to General Procedure D, the title compound was obtained as a yellow oil (61.9 mg, 0.118 mmol, 59% yield, dr 1:1). **R$_f$** = 0.20 [Hexanes: EtOAc 3:1 (v/v)]. **^{1}H NMR** (500 MHz, $CDCl_3$) δ 8.17 (d, J = 2.3 Hz, 1H), 8.09 (dd, J = 8.8, 2.3 Hz, 1H), 7.01 (d, J = 8.9 Hz, 1H), 5.34 (dd, J = 9.4, 2.5 Hz, 1H), 4.31 – 4.17 (m, 2H), 3.90 (d, J = 6.5 Hz, 2H), 2.77 (s, 3H), 2.65 – 2.51 (m, 2H), 2.20 (hept, J = 6.7 Hz, 1H), 1.76 (s, 1H), 1.36 – 1.22 (m, 9H), 1.09 (d, J = 6.7 Hz, 6H). **^{13}C NMR** (126 MHz, $CDCl_3$) δ 167.84, 163.81, 162.75, 162.28, 161.16, 132.73, 132.29, 126.01, 121.00, 115.40 (d, J = 13.8 Hz), 112.79, 103.19, 75.87, 73.95, 72.34, 63.28, 34.82 (t, J = 23.3 Hz), 28.30, 25.83, 19.18, 17.70, 13.97. **^{19}F NMR** (470 MHz, $CDCl_3$) δ -103.68 (dt, J = 268.0, 15.9 Hz, 1F), -104.64 (dt, J = 268.0, 16.2 Hz, 1F). **HRMS** (ESI+) *m/z* calcd for $C_{25}H_{31}F_2N_2O_6S$ [(M + H)$^+$], 525.1870, found, 525.1880.

ethyl 2,2-difluoro-5-hydroxy-4-((2-(4-isobutylphenyl)propanoyl)oxy)-5-methylhexanoate (3aw)

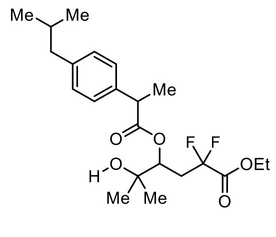

3aw

Prepared according to General Procedure D, the title compound was obtained as a yellow oil (45.6 mg, 0.110 mmol, 55% yield, dr 1:0.9). **R$_f$** = 0.20 [Hexanes: EtOAc 3:1 (v/v)]. **^{1}H NMR** (500 MHz, $CDCl_3$) δ 7.35 – 7.14 (m, 2H), 7.14 – 7.05 (m, 2H), 5.09 (dt, J = 9.8, 2.0 Hz, 1H), 4.29 (dq, J = 42.3, 7.2 Hz, 2H), 3.69 (dq, J = 48.1, 7.2 Hz, 1H), 2.54 – 2.27 (m, 4H), 1.83 (dpd, J = 13.6, 6.8, 4.7 Hz, 1H), 1.59 – 1.47 (m, 3H), 1.41 – 1.27 (m, 3H), 1.06 (d, J = 22.1 Hz, 3H), 0.87 (ddd, J = 6.6, 4.7, 1.1 Hz, 9H). **^{13}C NMR** (126 MHz, $CDCl_3$) δ 173.68, 173.54, 163.81, 140.97, 140.85, 137.71, 136.92, 129.55, 129.36, 127.47, 127.44, 127.13, 115.41 (dt, J = J = 256.3, 15.8 Hz), 73.03, 72.82, 72.28, 72.19, 63.11, 45.64, 45.12 (d, J = 5.3 Hz), 35.06, 34.76 – 33.90 (m), 30.30 (d, J = 4.3 Hz), 25.59, 25.41, 25.17, 24.83, 23.22 – 21.95 (m), 17.73, 17.60, 14.07, 13.99. **^{19}F NMR** (470 MHz, $CDCl_3$) δ -102.50 – -104.12 (m, 1F), -104.12 – -105.48 (m, 1F). **HRMS** (ESI+) *m/z* calcd for $C_{22}H_{37}F_2O_5N$ [(M + NH_4)$^+$], 432.2561, found, 432.2565.

ethyl 4-((3-(4,5-diphenyloxazol-2-yl)propanoyl)oxy)-2,2-difluoro-4-(4-hydroxytetrahydro-2H-pyran-4-yl)butanoate (3ax)

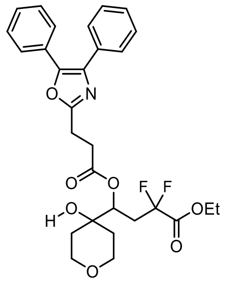

3ax

Prepared according to General Procedure D, the title compound was obtained as a colorless oil (80.4 mg, 0.148 mmol, 74% yield). **R*f*** = 0.08 [Hexanes: EtOAc 3:1 (v/v)]. **^{1}H NMR** (500 MHz, $CDCl_3$) δ 7.67 – 7.50 (m, 4H), 7.46 – 7.28 (m, 6H), 5.16 (dd, J = 10.3, 2.2 Hz, 1H), 4.33 (qd, J = 7.1, 1.9 Hz, 2H), 3.65 (ddd, J = 11.4, 5.2, 2.0 Hz, 1H), 3.52 – 3.40 (m, 2H), 3.30 – 3.12 (m, 2H), 3.01 – 2.83 (m, 2H), 2.57 (qd, J = 15.3, 10.4 Hz, 1H), 2.42 (tdd, J = 16.8, 15.2, 2.2 Hz, 1H), 1.55 (dddd, J = 13.7, 12.2, 8.2, 5.1 Hz, 2H), 1.42 – 1.28 (m, 4H), 1.28 – 1.20 (m, 2H). **^{13}C NMR** (126 MHz, $CDCl_3$) δ 170.49, 162.15, 145.84, 135.14, 132.03, 128.84, 128.76, 128.53, 128.21, 126.56, 115.46, 73.67, 70.64, 63.27, 63.02, 62.85, 34.52, 33.56 (t, J = 23.1 Hz), 32.97, 31.52, 23.50, 14.08. **^{19}F NMR** (470 MHz, $CDCl_3$) δ -104.03 (t, J = 16.2 Hz, 1F), -104.13 (t, J = 15.8 Hz, 1F). **HRMS** (ESI+) *m/z* calcd for $C_{29}H_{32}F_2NO_7$ [$(M + H)^+$], 544.2146, found, 544.2157.

6-ethoxy-5,5-difluoro-2-hydroxy-2-methyl-6-oxohexan-3-yl 4-(N,N-dipropylsulfamoyl)benzoate (3ay)

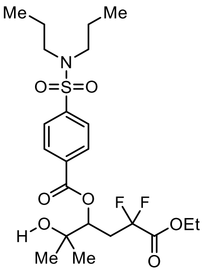

3ay

Prepared according to General Procedure D, the title compound was obtained as a yellow oil (62.2 mg, 0.126 mmol, 63% yield). **R*f*** = 0.20 [Hexanes: EtOAc 3:1 (v/v)]. **^{1}H NMR** (500 MHz, $CDCl_3$) δ 8.18 – 8.11 (m, 2H), 7.91 – 7.84 (m, 2H), 5.46 – 5.40 (m, 1H), 4.24 – 4.10 (m, 2H), 3.14 – 3.06 (m, 4H), 2.63 (td, J = 15.9, 6.0 Hz, 2H), 1.79 (s, 1H), 1.61 – 1.50 (m, 4H), 1.34 – 1.23 (m, 9H), 0.87 (t, J = 7.4 Hz, 6H). **^{13}C NMR** (126 MHz, $CDCl_3$) δ 164.56, 164.07 (t, J = 31.9 Hz), 144.76, 133.08, 130.50, 127.23, 115.35 (t, J = 251.4 Hz), 74.29, 72.41, 63.26, 50.18, 34.76 (t, J = 23.2 Hz), 29.83, 25.93, 25.84, 22.15, 13.94, 11.29. **^{19}F NMR**

(470 MHz, $CDCl_3$) δ -103.77 (dt, J = 268.0, 15.9 Hz, 1F), -104.87 (dt, J = 268.0, 16.1 Hz, 1F). **HRMS** (ESI+) *m/z* calcd for $C_{22}H_{34}F_2NO_7S$ [$(M + H)^+$], 494.2024, found, 494.2024.

9-(3,7-dimethyl-2,6-dioxo-2,3,6,7-tetrahydro-1H-purin-1-yl)-1-ethoxy-2,2-difluoro-5-hydroxy-5-methyl-1-oxononan-4-yl benzoate (3az)

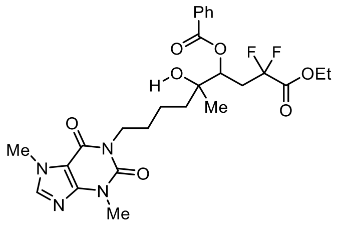

3az

Prepared according to General Procedure D, the title compound was obtained as a yellow oil (55.0 mg, 0.10 mmol, 50% yield). **R*f*** = 0.01 [Hexanes: EtOAc 3:1 (v/v)]. **^{1}H NMR** (500 MHz, $CDCl_3$) δ 8.05 – 7.98 (m, 2H), 7.60 – 7.50 (m, 1H), 7.48 – 7.40 (m, 2H), 7.26 (s, 1H), 5.58 – 5.30 (m, 1H), 4.46 (q, J = 7.2 Hz, 2H), 4.15 (dt, J = 3.8, 1.3 Hz, 3H), 4.06 – 3.94 (m, 2H), 3.51 (s, 3H), 2.73 – 2.55 (m, 2H), 2.17 (d, J = 39.2 Hz, 1H), 1.77 – 1.50 (m, 6H), 1.40 (t, J = 7.1 Hz, 3H), 1.30 – 1.20 (m, 6H).**^{13}C NMR** (126 MHz, $CDCl_3$) δ 165.86, 165.61, 155.73, 151.46, 146.95, 133.41, 129.91, 129.83, 128.58, 109.82, 74.15, 73.83, 73.35, 73.07, 64.24, 63.13 (d, J = 4.9 Hz), 40.90, 40.84, 37.33, 37.18, 34.81 (t, J = 24.7 Hz), 33.49, 29.96, 28.10, 28.02, 22.47, 19.96, 19.89, 14.05, 13.86. **^{19}F NMR** (470 MHz, $CDCl_3$) δ -101.91 – -103.16 (m, 1F), -105.14 (ddt, J = 267.2, 148.5, 16.6 Hz, 1F).

5,5-difluoro-2-hydroxy-6-(((1R,2S,5R)-2-isopropyl-5-methylcyclohexyl)oxy)-2-methyl-6-oxohexan-3-yl benzoate (3aaa)

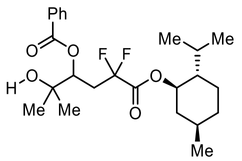

3aaa

Prepared according to General Procedure D, the title compound was obtained as a colorless oil (68.7 mg, 0.156 mmol, 78% yield). **R*f*** = 0.30 [Hexanes: EtOAc 3:1 (v/v)]. **^{1}H NMR** (500 MHz, $CDCl_3$) δ 8.07 – 8.00 (m, 2H), 7.60 – 7.53 (m, 1H), 7.44 (t, J = 7.8 Hz, 2H), 5.50 – 5.42 (m, 1H), 4.75 (td, J = 11.0, 4.5 Hz, 1H), 2.68 – 2.52 (m, 2H), 2.04 – 1.78 (m, 3H), 1.73 – 1.62 (m, 2H), 1.51 – 1.38 (m, 2H), 1.29 (d, J = 2.0 Hz, 6H), 1.10 – 0.93 (m, 2H), 0.92 – 0.82 (m, 6H), 0.74 (dd, J = 11.9, 7.0 Hz, 3H). **^{13}C NMR** (126 MHz, $CDCl_3$) δ 165.82, 163.70, 133.35, 133.32, 129.95 (d, J = 2.7 Hz), 128.55, 118.63 – 111.69 (m), 77.85 (d, J = 2.8 Hz), 73.54 (d, J = 3.8 Hz), 72.50, 46.90 (d, J = 3.1 Hz), 40.28 (d, J = 4.0 Hz), 35.31 – 34.17 (m), 34.11,

31.49, 26.21 (d, J = 3.8 Hz), 26.09 – 25.80 (m), 23.48, 23.42, 21.99 (d, J = 3.2 Hz), 20.76, 16.31, 16.21. [19]**F NMR** (470 MHz, $CDCl_3$) δ -102.74 – -104.02 (m, 1F), -104.02 – -105.12 (m, 1F). **HRMS** (ESI+) *m/z* calcd for $C_{24}H_{35}F_5O_5$ [$(M + H)^+$], 441.2452, found, 441.2464.

5,5-difluoro-2-hydroxy-2-methyl-6-oxo-6-(((2S,4R)-4,7,7-trimethylbicyclo[2.2.1]heptan-2-yl)oxy)hexan-3-yl benzoate (3aab)

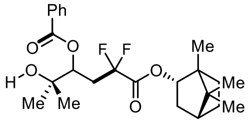

3aab

Prepared according to General Procedure D, the title compound was obtained as a colorless oil (64.9 mg, 0.148 mmol, 74% yield). $\mathbf{R}_f$ = 0.30 [Hexanes: EtOAc 3:1 (v/v)]. [1]**H NMR** (500 MHz, $CDCl_3$) δ 8.03 (dq, J = 7.0, 1.4 Hz, 2H), 7.57 (td, J = 7.3, 1.4 Hz, 1H), 7.44 (td, J = 7.8, 1.6 Hz, 2H), 5.48 – 5.39 (m, 1H), 4.87 (dq, J = 10.1, 2.9 Hz, 1H), 2.64 (td, J = 15.8, 5.8 Hz, 2H), 2.37 – 2.12 (m, 1H), 1.96 – 1.85 (m, 2H), 1.79 – 1.61 (m, 2H), 1.34 – 1.19 (m, 8H), 1.10 – 0.80 (m, 9H). [13]**C NMR** (126 MHz, $CDCl_3$) δ 165.79, 164.06, 133.38, 130.02 – 129.67 (m), 128.54, 119.40 – 106.57 (m), 83.33 (d, J = 4.0 Hz), 73.64 (d, J = 3.9 Hz), 72.45, 49.21 (d, J = 10.6 Hz), 48.12, 44.87 (d, J = 3.2 Hz), 36.27 (d, J = 12.8 Hz), 34.63 (td, J = 23.3, 8.3 Hz), 27.95 (d, J = 3.4 Hz), 27.02 (d, J = 4.1 Hz), 25.90 (d, J = 7.6 Hz), 18.86, 13.53, 13.38. [19]**F NMR** (470 MHz, $CDCl_3$) δ -102.86 – -103.68 (m, 1F), -103.68 – -104.49 (m, 1F). **HRMS** (ESI+) *m/z* calcd for $C_{24}H_{32}F_2O_5Na$ [$(M + Na)^+$], 461.2115, found, 461.2124.

6-(((3S,8S,9S,10R,13R,14S,17R)-10,13-dimethyl-17-((R)-6-methylheptan-2-yl)-2,3,4,7,8,9,10,11,12,13,14,15,16,17-tetradecahydro-1H-cyclopenta[a]phenanthren-3-yl)oxy)-5,5-difluoro-2-hydroxy-2-methyl-6-oxohexan-3-yl benzoate (3aac)

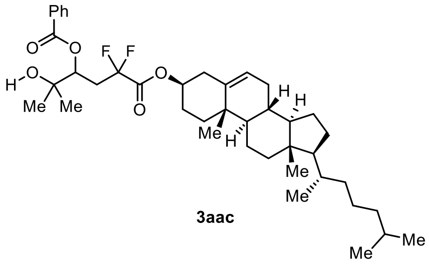

3aac

Prepared according to General Procedure D, the title compound was obtained as a colorless oil (83.2 mg, 0.124 mmol, 62% yield). $\mathbf{R}_f$ = 0.24 [Hexanes: EtOAc 3:1 (v/v)]. [1]**H NMR** (500 MHz, $CDCl_3$) δ 8.04 – 8.02 (m, 2H), 7.57 (dddd, *J* = 7.8, 5.4, 2.3, 1.2 Hz, 1H), 7.48 – 7.41 (m, 2H), 5.46 – 5.37 (m, 1H), 5.36 – 5.20 (m, 1H),), 4.56 (ddd, *J* = 15.8, 8.4, 4.2 Hz, 1H), 2.70 – 2.56 (m, 2H), 2.32 (dddd, *J* = 18.4, 13.1, 5.3, 2.4 Hz, 2H), 2.06 – 1.91 (m, 2H), 1.83 (dddd, *J* = 16.6, 13.0, 9.8, 4.8 Hz, 3H), 1.73 – 1.40 (m, 4H), 1.40 – 1.31

(m, 2H), 1.31 – 1.21 (m, 6H), 1.21 – 0.95 (m, 17H), 0.94 – 0.87 (m, 4H), 0.87 – 0.83 (m, 10H), 0.68 – 0.64 (m, 3H). **^{13}C NMR** (126 MHz, $CDCl_3$) δ 165.78, 163.27, 138.99 (d, J = 6.5 Hz), 133.41, 129.88 (d, J = 11.4 Hz), 128.59, 123.40, 115.44, 73.70, 72.52, 56.81, 56.29, 50.09, 42.45, 39.75 (d, J = 23.0 Hz), 37.60 (d, J = 14.5 Hz), 36.91, 36.33, 35.92, 34.99 – 34.45 (m), 31.98 (d, J = 9.0 Hz), 28.36, 27.39 (d, J = 11.1 Hz), 25.91 (d, J = 12.0 Hz), 24.41, 23.97, 22.96, 22.70, 21.16, 19.38, 18.86, 11.99. **^{19}F NMR** (470 MHz, $CDCl_3$) δ -102.10 – -103.84 (m, 1F), -104.96 (ddt, J = 267.9, 27.1, 16.2 Hz, 1F).

tert-butyl 8-(4-(benzoyloxy)-2,2-difluoro-5-hydroxy-5-methylhexanoyl)-3,8-diazabicyclo[3.2.1]octane-3-carboxylate (3aad)

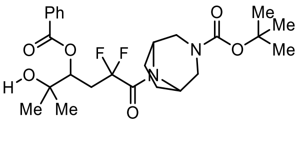

3aad

Prepared according to General Procedure D, the title compound was obtained as a colorless oil (53.6 mg, 0.108 mmol, 54% yield). **R_f** = 0.30 [Hexanes: EtOAc 3:1 (v/v)]. **^{1}H NMR** (500 MHz, $CDCl_3$) δ 8.07 – 8.00 (m, 2H), 7.60 – 7.53 (m, 1H), 7.44 (td, J = 7.8, 1.8 Hz, 2H), 5.51 (ddd, J = 13.7, 9.8, 1.5 Hz, 1H), 4.57 (td, J = 22.7, 9.9 Hz, 2H), 3.89 (dd, J = 21.7, 13.2 Hz, 1H), 3.73 (q, J = 13.3 Hz, 1H), 3.15 – 2.73 (m, 3H), 2.61 (d, J = 14.9 Hz, 1H), 2.30 – 2.22 (m, 1H), 2.02 – 1.60 (m, 4H), 1.43 (d, J = 8.0 Hz, 9H), 1.35 – 1.26 (m, 6H). **^{13}C NMR** (126 MHz, $CDCl_3$) δ 165.87 (t, J = 31.9 Hz), 159.16, 155.73 (d, J = 3.7 Hz), 133.30, 130.08, 129.83, 128.56, 119.09 (t, J = 251.4 Hz), 80.33, 73.76 (d, J = 17.2 Hz), 72.41 (d, J = 6.4 Hz), 54.97, 54.59, 53.37 – 52.05 (m), 51.35, 50.14, 48.96, 34.37 (t, J = 22.3 Hz), 28.45 (d, J = 2.8 Hz), 27.65, 26.02, 25.66 – 24.68 (m). **^{19}F NMR** (470 MHz, $CDCl_3$) δ -95.43 – -98.96 (m, 1F), -99.15 – -103.93 (m, 1F). **HRMS** (ESI+) *m/z* calcd for $C_{25}H_{35}F_2N_2O_6$ [(M + H)$^+$], 497.2463, found, 497.2471.

Gram-scale Reaction

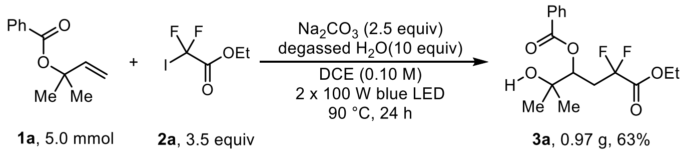

In a glovebox, to an oven-dried 200 mL round bottom flask was added Na_2CO_3 (1.33 g, 12.5 mmol, 2.5 equiv), **1a** (0.95 g, 5.0 mmol, 1.0 equiv), **2a** (4.375 g, 17.5 mmol, 3.5 equiv), and DCE (50 mL, 0.10 M). The vial was equipped with a stir bar, capped and sealed with black tape, then taken out of the glovebox, subsequently degassed H_2O (900 μL, 50.0 mmol, 10 equiv) was added using syringe through septum. The reaction mixture was stirred at room temperature and irradiated with two 100 W Blue LEDs for 24 h. The

reaction mixture was then concentrated *in vacuo* and residue was purified by flash column chromatography on silica gel to afford the desired product as a colorless oil (0.97 g, 3.15 mmol, 63%).

Mechanistic Studies

Radical Trapping Experiment

The procedure is based on General Procedure D: In a glovebox, to an oven-dried 20 mL screw cap vial was added Na_2CO_3 (5.3 mg, 0.05 mmol, 2.5 equiv), TEMPO (3.13 mg, 0.02 mmol, 1.0 equiv), allyl ester (0.02 mmol, 1.0 equiv), alkyl iodide **2a** (17.5 mg, 0.07 mmol, 3.5 equiv), and DCE (0.2 mL, 0.10 M). The vial was equipped with a stir bar, capped and sealed with black tape, then taken out of the glovebox, subsequently degassed H_2O (3.6 μL, 0.20 mmol, 10 equiv) was added using syringe through septum. The reaction mixture was stirred at 90 °C and irradiated with 100 W Blue LEDs for 24 h. The yield was determined based on crude ^{1}H-NMR spectrum with dibromomethane as an internal standard. The mass of TEMPO trapped product **4** was detected on HRMS. HRMS (ESI-TOF) *m/z* calcd for $C_{13}H_{24}F_2NO_3$ [(M + H)$^+$], 280.17188, found, 280.17137. The spectroscopic data correspond to previously reported data.[8]

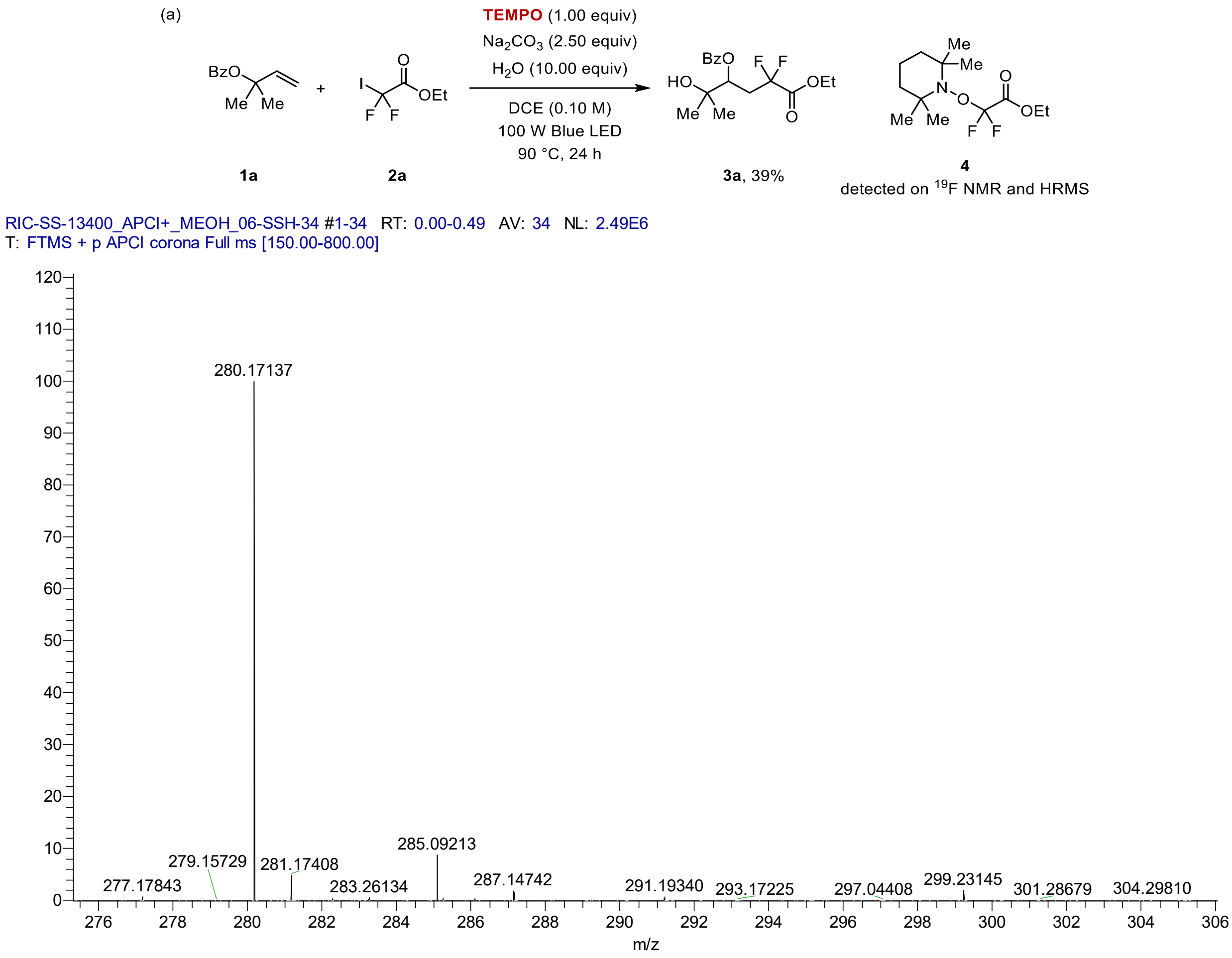

Fig. S1. Radical trapping experiments with TEMPO.

Results and Conclusion: TEMPO-adduct **4** was detected by HRMS and ^{19}F NMR spectrum in the presence of a radical scavenger, 2,2,6,6-tetramethylpiperidine 1-oxyl radical (TEMPO, 1.0 equiv). This suggests that the reaction likely involves a radical mechanism.

1,2-Iododifluoroalkylation intermediate as the substrate

The procedure is based on General Procedure D: In a glovebox, to an oven-dried 4 mL screw cap vial was added 1,2-iododifluoroalkylation intermediate **5** (7.86 mg, 0.02 mmol, 1.0 equiv), iododifluoroacetate **2a** (17.5 mg, 0.07 mmol, 3.5 equiv), Na_2CO_3 (5.3 mg, 0.05 mmol, 2.5 equiv), and DCE (0.2 mL, 0.10 M). The vial was equipped with a stir bar, capped and sealed with black tape, then taken out of the glovebox, subsequently degassed H_2O (3.6 µL, 0.20 mmol, 10 equiv) was added using syringe through septum. The reaction mixture was stirred at 90 °C and irradiated with 100 W Blue LEDs for 24 h. The desired product was observed on the crude ^{1}H-NMR spectrum.

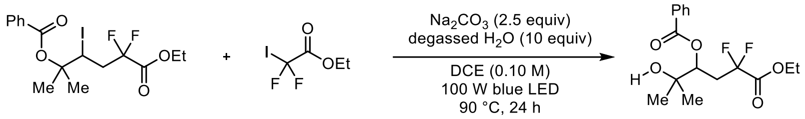

5 **2a** **3a**, 41%

Fig. S2. Reaction using 1,2-iododifluoroalkylated intermediate as the substrate.

Results and Conclusion: The reaction likely goes via 1,2-iododifluoroalkylated intermediate **5**.

Cross-over experiment

The procedure is based on General Procedure D: In a glovebox, to an oven-dried 4 mL screw cap vial was added **1au** (1.96 mg, 0.01 mmol, 0.5 equiv), **1d** (2.88 mg, 0.01 mmol, 0.5 equiv), iododifluoroacetate **2a** (17.5 mg, 0.07 mmol, 3.5 equiv), Na_2CO_3 (5.30 mg, 0.05 mmol, 2.5 equiv), and DCE (0.2 mL, 0.10 M). The vial was equipped with a stir bar, capped and sealed with black tape, then taken out of the glovebox, subsequently degassed H_2O (3.6 µL, 0.20 mmol, 10 equiv) was added using syringe through septum. The reaction mixture was stirred at 90 °C and irradiated with 100 W Blue LEDs for 24 h. At the end of the reaction, **3a** and **3aae** were not detected on both LC-MS spectrum and crude ^{1}H-NMR spectrum. The yield of **3au** and **3d** were determined based on crude ^{1}H-NMR spectrum with dibromomethane as an internal standard.

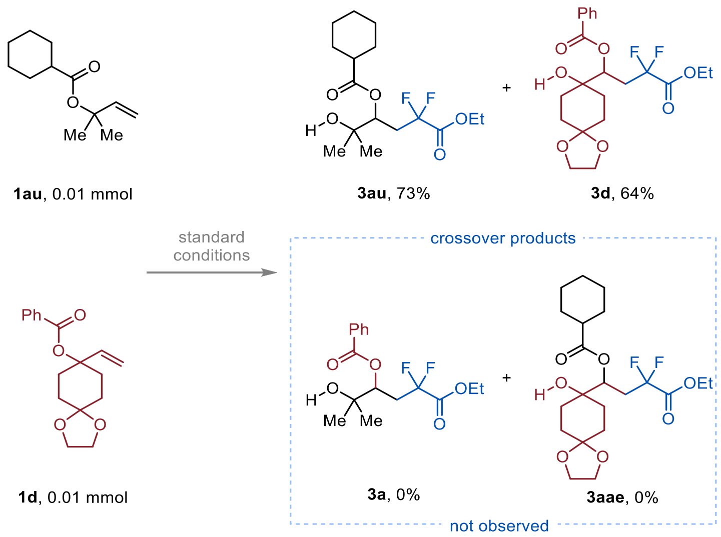

Fig. S3. Cross-over experiments.

Results and Conclusion: No cross-over products (**3a** and **3aae**) were observed. The ester migration likely proceeds through intramolecular pathway.

Light On/Off experiment

The procedure is based on General Procedure D. In a glovebox, to six oven-dried 4 mL screw cap vials were added Na_2CO_3 (5.3 mg, 0.05 mmol, 2.5 equiv), **1a** (0.02 mmol, 1.0 equiv), **2a** (0.07 mmol, 3.5 equiv), and DCE (0.20 mL, 0.10 M). The vial was equipped with a stir bar, capped and sealed with black tape, then taken out of the glovebox, subsequently degassed H_2O (3.6 μL, 0.20 mmol, 10 equiv) was added using syringe through septum. The reaction mixture was stirred at 90 °C and irradiated with 100 W Blue LEDs. After 30 minutes one of the vials was removed then the lights were turned off for 30 minutes then another vial was removed, and lights were turned on for 30 minutes. This sequence (30 minutes lights on, 30 minutes lights off) was continued until all vials were removed, then crude NMR yield was obtained using dibromomethane as internal standard.

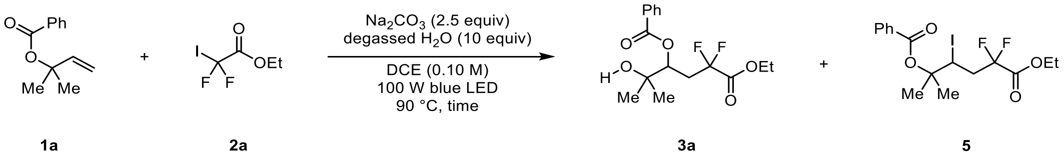

Light condition		on	Off	on	off	on	Off
Time (h)	0	0.5	1	1.5	2	2.5	3
Combined yield (3a + 5)	0	31%	31%	43%	44%	51%	52%

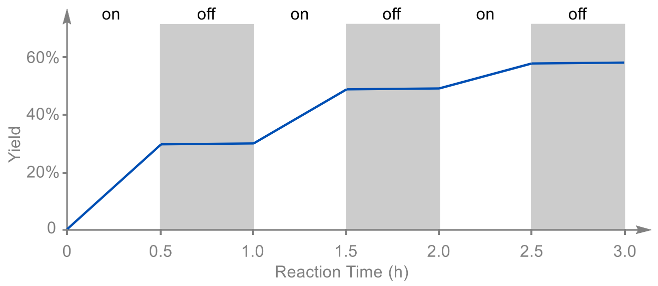

Fig. S4. Light On/Off experiment.

Results and Conclusion: Extended radical chain mechanism under standard conditions is unlikely

Quantum Yield Experiment

The following quantum yield measurements are adapted from the procedure developed by Yoon et al.[9]

Determination of the Light Intensity at 450 nm:

The fraction of light absorbed (f) by ferrioxalate solution was calculated as shown below, where the absorbance of the ferrioxalate solution at 450 nm was measured to be 1.818035 (A), based on the equation ($f = 1\text{-}10^{-A}$), indicating $f = 0.9847$.

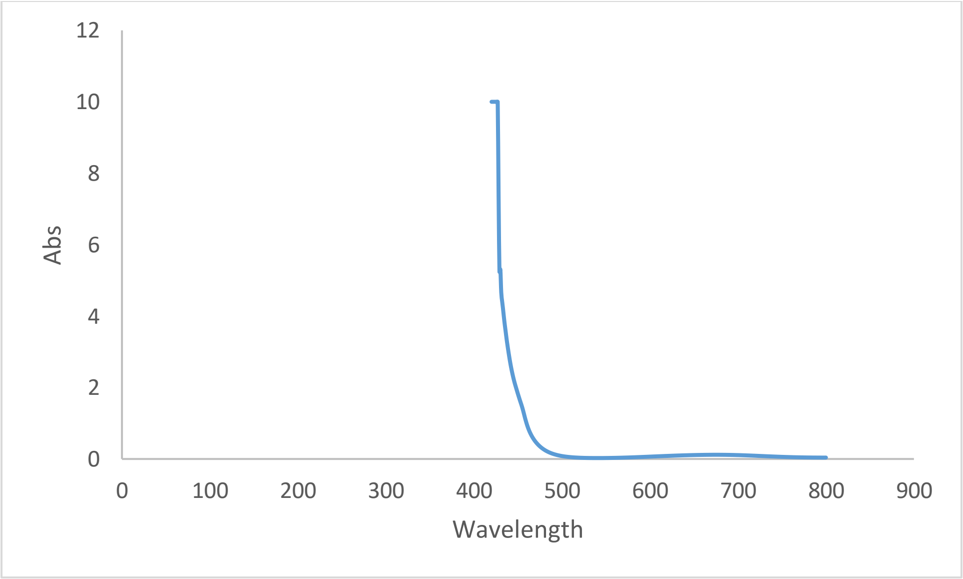

Fig. S5. Absorbance of the ferrioxalate solution at 450 nm (A = 1.818035).

The photon flux of the 100 W Blue LEDs (λmax = 450 nm) was determined by standard ferrioxalate actinometry.[14] A 0.150 M solution of ferrioxalate was prepared by dissolving 2.21 g of potassium ferrioxalate hydrate ($K_3[Fe(C_2O_4)_3]\cdot3H_2O$) in 30.0 mL of 0.0500 M H_2SO_4 (aq). Next, a buffered solution of phenanthroline was prepared by dissolving 50.0 mg of phenanthroline and 11.25 g of sodium acetate in 50.0 mL of 0.500 M H_2SO_4 (aq). Both solutions were stored in an amber vial in the dark. To determine the

photon flux of the 100 W blue LEDs, 2.00 mL of the ferrioxalate solution was placed in a 4.00 ml vial and irradiated for 5.00 seconds at $\lambda = 450$ nm. After irradiation, 0.500 mL of the phenanthroline solution was added to the vial. The solution was then rested for 1 h in the dark to allow the ferrous ions to completely coordinate to the phenanthroline. A non-irradiated sample was also prepared and developed in the dark (*note: after developing the non-irradiated samples they were diluted with a dilution factor of 6.25 to prevent deviation from the Beer-Lambert law at high concentrations A = >2. Thus, to obtain the actual mol of Fe2+ they were multiplied by 6.25*).

1 Ferrioxalate Actinometry

$$\text{mol of Fe}^{2+} = 6.25 \times \left[\frac{V \times \Delta A_{510}}{l \times \varepsilon_{510}}\right]$$

$$\text{mol of Fe}^{2+} = 6.25 \times \left[\frac{0.00250 \times 0.48978}{1 \times 11{,}100}\right] \text{mol}$$

$$= 7.07 \times 10^{-7} \text{ mol}$$

V= 0.00300 L *(total volume)*
ΔA_{510} = 0.418629 *(difference in absorption at 510 nm)*
l = 1.00 cm *(path length)*
ε_{510} = 11,100 L mol^{-1}cm^{-1} *(molar absorptivity at 510 nm)*

2 Determination of photon flux of 100 W blue LED light

$$\text{photon flux} = \left[\frac{\text{mol of Fe}^{2+}}{\phi \times t \times f}\right]$$

$$\text{photon flux} = \left[\frac{7.07 \times 10^{-7}}{1.01 \times 5.00 \times 0.9847}\right] \text{einstein s}^{-1}$$

$$= 1.42 \times 10^{-7} \text{ einstein s}^{-1}$$

ϕ = 1.01 *(quantum yield of ferrioxalate actiometer)*
t = 5.00s *(time)*
f = 0.9847 *(Fraction of light absorbed)*

Fig. S6. Determination of the light intensity (photon flux) at 450 nm via ferrioxalate actinometry (ε = 11,100 L mol^{-1}cm^{-1}).[16a]

Afterward, the absorbance of both solutions was measured at 510 nm and with mol of Fe^{2+} known. Next the photon flux was determined to be 1.42×10^{-7} einstein s^{-1}. We can obtain the quantum yield of our reaction provided if it is irradiated using the same geometry (*note: although Φ = 1.01 at 436 nm was used for the calculation of the photon flux, it is known that the ferrioxalate system varied little with the wavelength as the Φ remained between 0.9 and 1.1 at a wavelength between 400–480 nm*).[16a]

Determination of Quantum Yield:

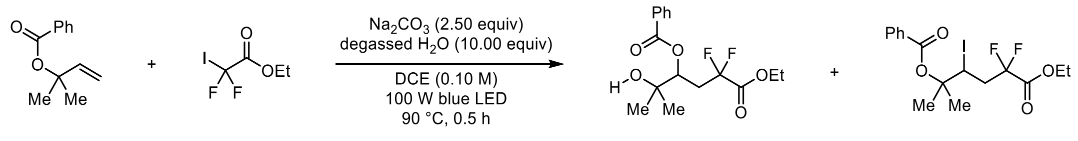

1a **2a** **3a** **3a'**

ϕ = 0.206

To determine the quantum yield, in a glovebox, the cuvette was charged with Na_2CO_3 (26.5 mg, 0.25 mmol, 2.5 equiv), **1a** (0.10 mmol, 1.0 equiv), **2a** (0.35 mmol, 3.5 equiv), and DCE (1.0 mL, 0.10 M). The vial was equipped with a stir bar, capped and sealed with teflon, then taken out of the glovebox, subsequently degassed H_2O (18 μL, 0.10 mmol, 10 equiv) was added using syringe through septum. The cuvette was placed with half of the solvent in an oil bath at 90 °C. The reaction mixture was irradiated (λ_{max} = 450 nm) for 1800 s (30 min) with the same 100 W Blue LEDs. To determine the yield of the product, the solvent is removed under vacuum, an internal standard, dibromomethane (CH_2Br_2) (8.69 mg, 0.05 mmol) was added to the cuvette, followed by 500 μL $CDCl_3$. The reaction was repeated three times with yield to be: 52%, 54%, 50%. The quantum yield was determined using the equation shown below.

1 Quantum Yield

$$\phi = \left[\frac{\text{mol x yield \%}}{\text{photon flux x t x f}}\right]$$

$$\phi = \left[\frac{0.0001 \times 0.52}{1.42 \times 10^{-7} \times 1800 \times 0.9847}\right]$$

$$= 0.206$$

ϕ = quantum yield of the reaction
t = 1800s (time)
f = (Fraction of light absorbed)
yield% = averaged yield of three trials

Fig. S7. Quantum yield calculation.

Results and Conclusion: Quantum yield experiment suggests that an extended radical chain propagation is unlikely.

Job's plot

The binding stoichiometry between ICF_2CO_2Et **2a** and halogen bond acceptors (XB acceptor: H_2O) were evaluated using Job's plot analysis. ^{19}F NMR spectra of ten samples of mixtures of ICF_2CO_2Et and XB acceptor in $CDCl_3$ were recorded at 298 K. Trifluoroluorobenzene (δ F-Ph = -63.7200) was used as internal standard. The total volume of the mixture was 0.6 mL, and the total amount of ICF_2CO_2Et and halogen bond acceptor was kept constant at 0.2 mmol (1 M), while the amount of ICF_2CO_2Et was varied from 0 to 0.2 mmol (0-1 M). The molar ratios of ICF_2CO_2Et /(ICF_2CO_2Et + H_2O) were 0.0, 0.10, 0.20, 0.30, 0.40, 0.50, 0.60, 0.80, 0.90, and 1.0. 19F NMR for each sample was recorded and the chemical shifts differences (Δδ) for -CF_2I were used to draw the plot. The stoichiometry was determined by plotting ratios of [ICF2CO2Et] × Δδ against ratios of [ICF_2CO_2Et] / [ICF_2CO_2Et + H_2O] to afford a maximum at ratio [ICF_2CO_2Et] / [ICF_2CO_2Et + H_2O] = 0.5, which meant a 1:1 complex ratio between ICF_2CO_2Et and H_2O (Figure S8).

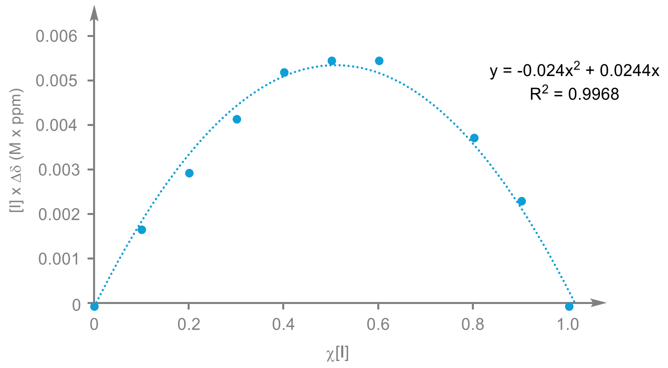

Fig. S8. Job's plot.

Results and Conclusion: Complexation between H_2O and **2a** is 1:1

UV-Vis absorption spectrometry

UV-Vis absorption spectrum of alkyl iodide **2z**, and water was measured with 0.005 M DCE solution. UV–vis absorption spectra of **2z** in the presence of water exhibited an enhancement in absorption (Fig. S9), indicative of halogen-bonding interactions between water and the iododifluoro reagent.

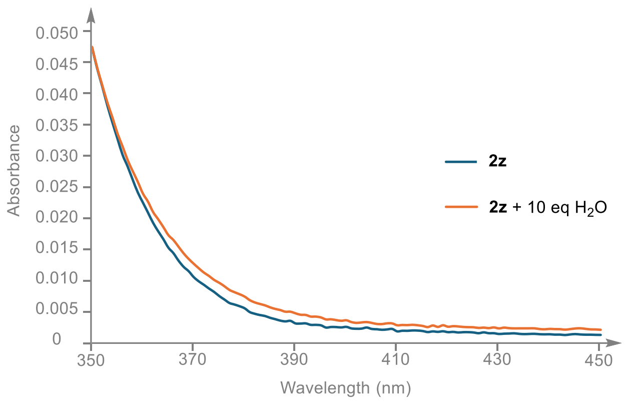

Fig. S9. UV-Vis Spectra

^{18}O-Labeling experiment

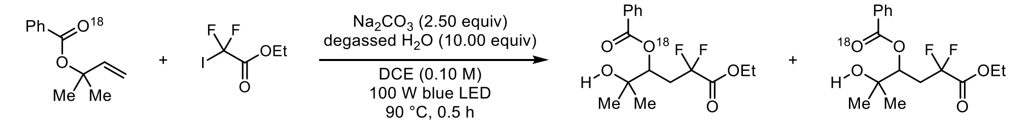

^{18}O-1a (95% ^{18}O) **^{18}O-3a** **^{18}O-3a-3**

Prepared according to General Procedure D, the title compound was obtained as a colorless oil (49 mg, 0.148 mmol, 74% yield), **R*f*** = 0.16 [Hexanes: EtOAc 3:1 (v/v)]. The spectroscopic data corresponds to **3a**. The solution of **^{18}O-3a**, and **^{18}O-3a-3** was injected in GC-MS, and their content was determined based on GC-MS analysis for fragments containing PhCO, and PhC(O)O.

For the fragment containing PhC(O) group with ^{18}O incorporation, where expected masses would be 105, and 107 respectively for **^{18}O-3a**, and **^{18}O-3a-3** respectively, which was calculated to be 92%, and 8% respectively, as shown in the snapshot below.

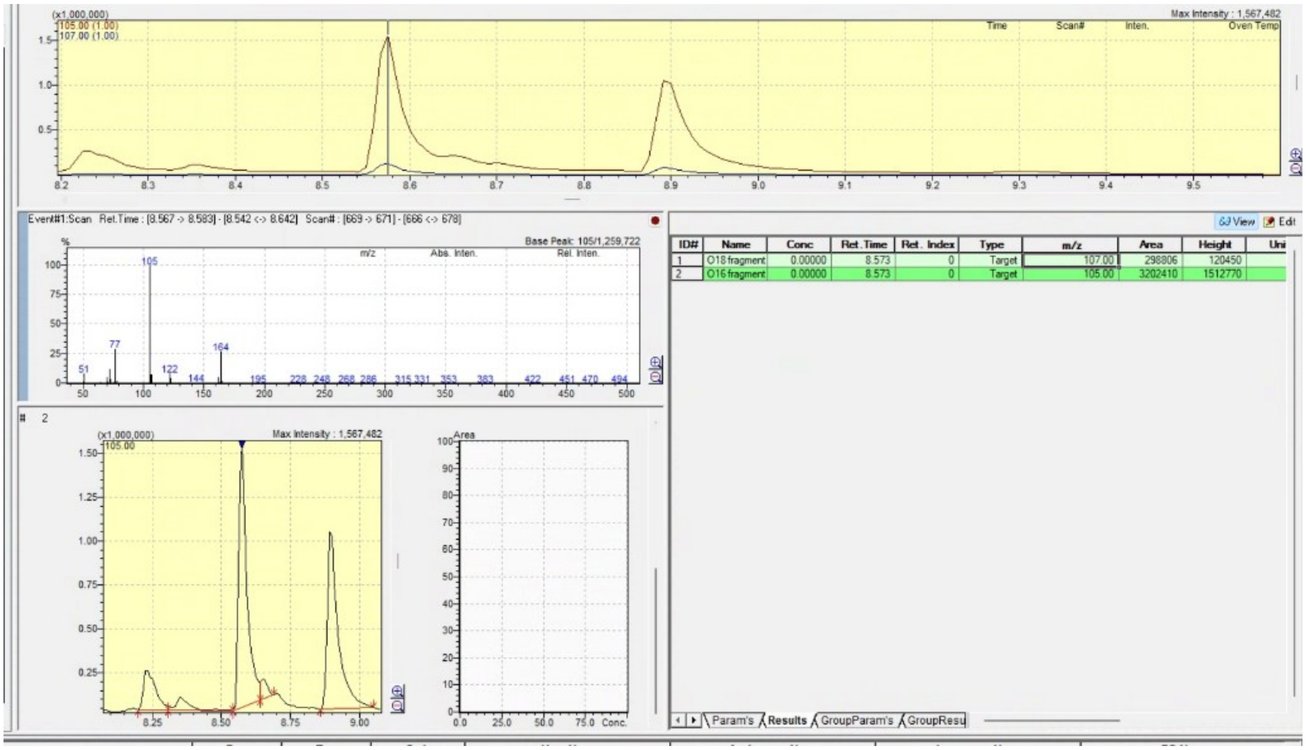

Next for PhC(O)O fragment from the product, whose expected masses would be 123, and 121 for **^{18}O-3a**, and **^{18}O-3a-3** respectively, which was calculated to be 96%, and 4% respectively, as shown in the snapshot

below.

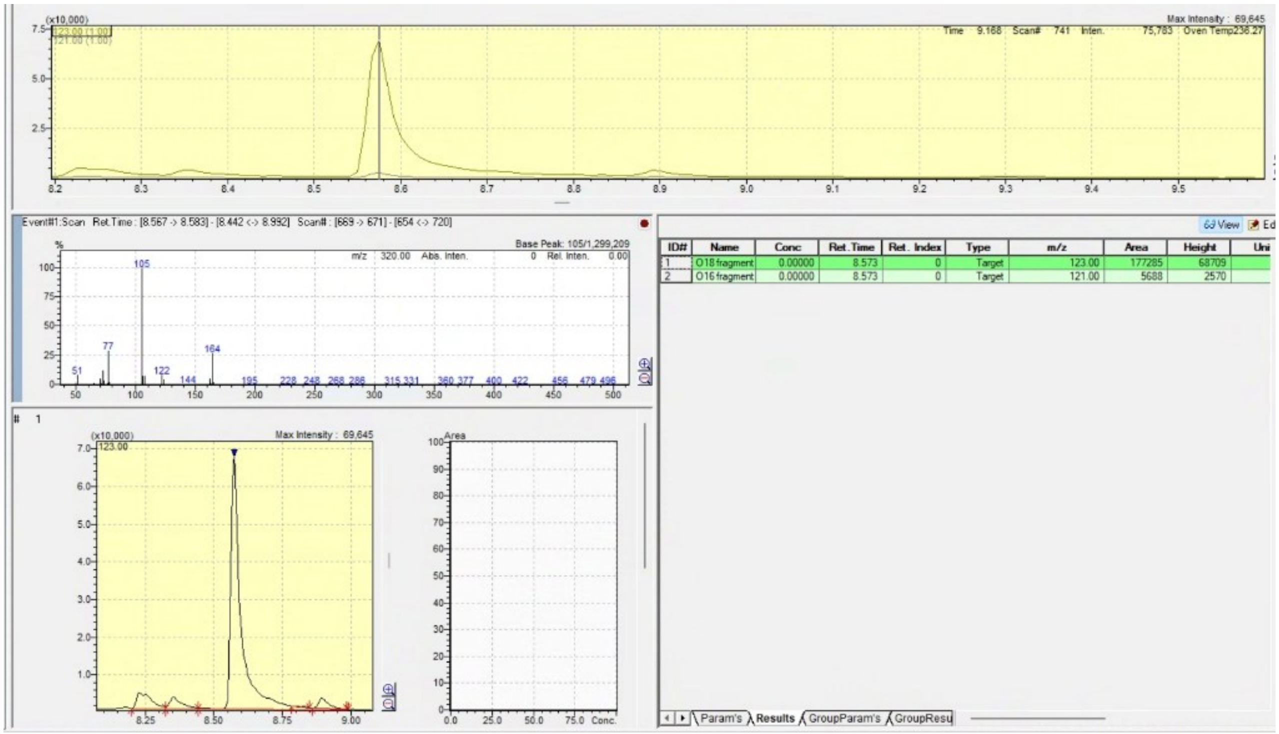

Results and Conclusion: The acyloxy migration likely proceeds via dioxolium cation intermediate **VII**

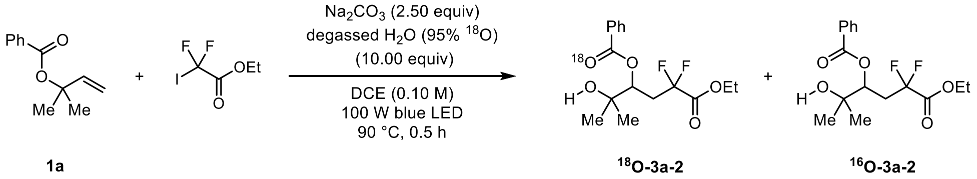

Prepared according to General Procedure D, the title compound was obtained as a colorless oil (50 mg, 0.15 mmol, 75% yield), $\mathbf{R}_f$ = 0.16 [Hexanes: EtOAc 3:1 (v/v)]. The spectroscopic data corresponds to **3a**. The ^{18}O content was determined after deprotection.

Deprotection of ^{18}O-3a-2 and ^{16}O-3a-2:

To a solution of **^{18}O-3a-2** and **^{16}O-3a-2** mixture (6.60 mg) in MeOH/H_2O (0.500 mL, 1:1, v/v) was added KOH (15.4 mg). The reaction mixture was stirred at 80 °C for 15 minutes. Then the reaction mixture was diluted with MeCN and injected to GC-MS, **^{18}O-6a** and **^{16}O-6a** ratio is 3.58:1.0.

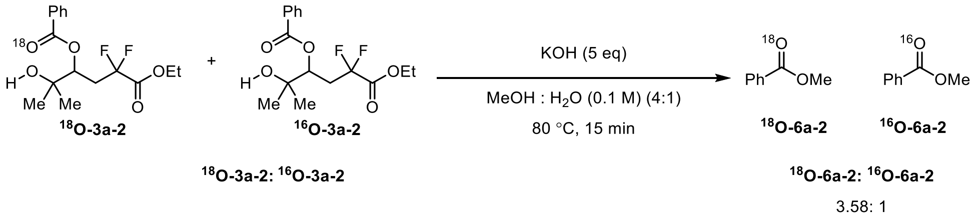

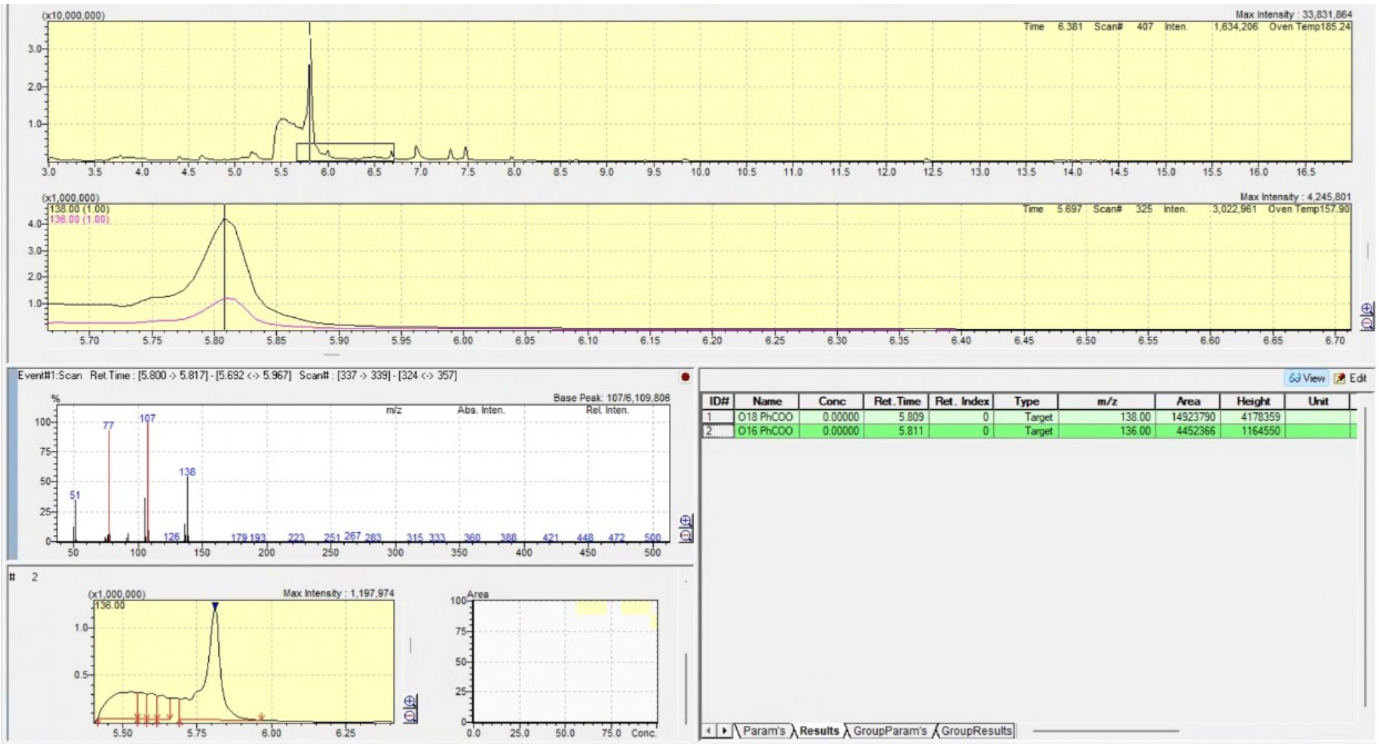

Results and Conclusion: H_2O likely attacks the benzylic position of carbocation intermediate **VII**

Effect of Water Equivalents on the Yield of the 1,3-Carbohydroxylation Reaction

Table S1 Effect of water equivalents

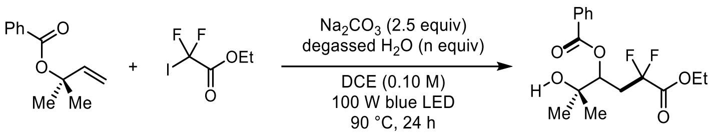

1a, 1.0 equiv **2a**, 3.5 equiv **3a**

Entry	Equivalents of H_2O (n)	NMR-Yield (%)
1	0	37
2	1	71
3	2.5	77
4	5	81
5	7.5	83
6	10	98

The procedure is based on General Procedure D. In a glovebox, to six oven-dried 4 mL screw cap vials were added Na_2CO_3 (5.3 mg, 0.05 mmol, 2.5 equiv), **1a** (3.8 mg, 0.02 mmol, 1.0 equiv), **2a** (17.5 mg, 0.07 mmol, 3.5 equiv), and DCE (0.20 mL, 0.10 M). The vial was equipped with a stir bar, capped and sealed with black tape, then taken out of the glovebox, subsequently degassed H_2O was added using syringe through septum. The reaction mixture was stirred at 90 °C and irradiated with 100 W Blue LEDs for 24 h. Then crude NMR yields were obtained using dibromomethane as internal standard.

Results and Conclusion: These studies indicate that higher water loadings are required to achieve optimal reaction efficiency, with 10 equivalents giving the best performance.

Effect of reaction temperature on the Yield of the 1,3-Carbohydroxylation Reaction

Table S2 Effect of reaction temperature

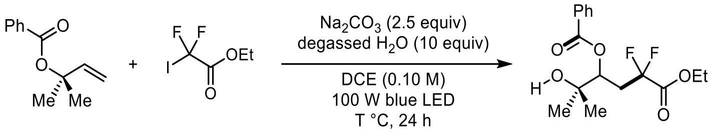

1a, 1.0 equiv **2a**, 3.5 equiv **3a**

Entry	Temperature (T)	NMR-Yield (%)
1	25	15
2	50	25
3	70	57
4	90	98

The procedure is based on General Procedure D. In a glovebox, to four oven-dried 4 mL screw cap vials were added Na_2CO_3 (5.3 mg, 0.05 mmol, 2.5 equiv), **1a** (3.8 mg, 0.02 mmol, 1.0 equiv), **2a** (17.5 mg, 0.07 mmol, 3.5 equiv), and DCE (0.20 mL, 0.10 M). The vial was equipped with a stir bar, capped and sealed with black tape, then taken out of the glovebox, subsequently degassed H_2O (3.6 µL, 0.20 mmol, 10 equiv) was added using syringe through septum. The reaction mixture was stirred at T °C and irradiated with 100 W Blue LEDs for 24 h. Then crude NMR yields were obtained using dibromomethane as internal standard.

Results and Conclusion: These studies show that higher reaction temperatures are necessary for optimal product formation, with the best yield obtained at 90 °C.

DFT Calculations

Computational Details

All density functional theory (DFT) calculations were performed with the Gaussian 16 software package.[10] Geometries of intermediates and transition states were optimized using the dispersion-corrected B3LYP[11] -D3[12] functional (UB3LYP-D3 for open-shell systems) with Becke–Johnson (BJ)[13] damping. A mixed basis set was employed, consisting of SDD[14] (Stuttgart/Dresden effective core potential with its associated basis set) for iodine atoms and 6-31G(d) for all other atoms, together with the SMD solvation model in dichloroethane (DCE). Vibrational frequency analyses were conducted to confirm that optimized structures correspond to minima (no imaginary frequencies) or transition states (one imaginary frequency).

Single-point energy refinements were performed with the M06 functional [15] (UM06 for open-shell systems) using the same mixed basis set, with SDD for iodine and 6-311+G(d,p) for all other atoms. Solvation effects were included at this stage using the SMD model[16] in DCE. Three-dimensional structures were visualized with CYLView.[17]

All structures and energies presented in the Introduction section were calculated with the same methodology described above, except that optimizations and energy evaluations were carried out in the gas phase. This approach was chosen to provide more generalizable trends independent of solvation effects.

Conformational sampling was conducted with the xTB Conformer–Rotamer Ensemble Sampling Tool (CREST),[18] and the lowest-energy conformers were subsequently used for DFT calculations. Only the lowest-energy structures are discussed in this work.

Thermochemical corrections were obtained with the GoodVibes program (v3.2).[19] The quasi-harmonic approximation was applied with a frequency cutoff of 100 cm^{-1}, and Gibbs free energies were corrected to 363.15 K. Final free energies were determined by combining single-point electronic energies with these thermal corrections.

Computational Investigation of Reaction Mechanism

We investigated the pathway by which water facilitates photolysis of the C–I bond in ethyl difluoro(iodo)acetate (**2a**) under blue-light irradiation through halogen bonding. Our experimental UV–vis spectra revealed an increased absorption upon water addition, consistent with halogen-bond formation. Computationally, we observed a significant decrease in the calculated bond dissociation energy (BDE). The C–I bond BDE in the absence of water coordination was 38.1 kcal/mol, whereas coordination of water to the iodine σ-hole reduced the BDE to 28.3 kcal/mol.

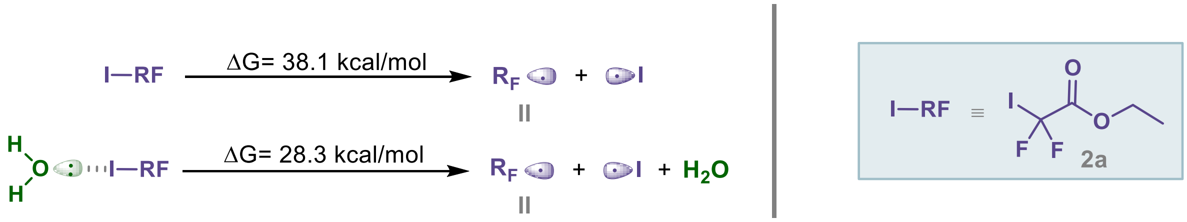

__Comparison of Water vs Carbonate as Halogen Bond Acceptor:__

To examine whether Na_2CO_3 could serve as a halogen-bond acceptor or co-mediator in place of water,

Energetics of Complex Formation:

We computed the free energy changes (ΔG) associated with the formation of **2a**-water and **2a**-carbonate complexes:

2a + water → 2a–water $\Delta G = 9.85$ kcal/mol

2a + carbonate → 2a–carbonate $\Delta G = 9.32$ kcal/mol

These results show that water destabilizes the C–I bond in **2a** to a greater extent than carbonate by approximately 0.5 kcal/mol. This is reflected in the higher energy of the **2a**–water complex relative to **2a**–carbonate.

Bond Dissociation Energies (BDEs):

This destabilization is reflected on how the presence of these acceptors impacts the BDE of the C–I bond:

2a → II + I–radical BDE = 38.10 kcal/mol

2a–water → II + I–radical + water BDE = 28.25 kcal/mol

2a–carbonate → II + I–radical + carbonate BDE = 28.78 kcal/mol

As shown, the BDE drops significantly in the presence of either water or carbonate, reflecting increased lability of the C–I bond when these acceptors are present. Notably, the BDE is lowest for the water complex, indicating that water mediates this step slightly more efficiently than carbonate (~0.53 kcal/mol difference).

Taken together, these results support the conclusion that both water and carbonate are capable of halogen-bond acceptance in principle. However, considering (i) the somewhat stronger interaction and lower BDE for the water complex, and (ii) the relative quantities present under the reaction conditions (10 equivalents of water vs. 2.5 equivalents of Na_2CO_3), water is more likely to be the predominant halogen-bond acceptor interacting with substrate 2a during the C–I activation step. Nevertheless, we cannot rule out the possibility that Na_2CO_3 may also engage in halogen bonding or otherwise contribute to halide activation.

Photolysis of the C–I bond generates an iodine radical and a difluoroethyl ester radical (**II**). Radical **II** is expected to be electron-deficient due to the presence of two fluorine substituents, enabling it to interact with electron-rich alkenes. Consistent with this, addition of **II** to the allyl benzoate substrate (**1a**) was found to be thermodynamically favorable (ΔG = –13.4 kcal/mol), reflecting the formation of a more stabilized radical intermediate. The transition state for this addition (**TS1**) was located, with a moderate barrier of $\Delta G^{\ddagger}$ = 18.1 kcal/mol.

Halogen Atom Transfer (XAT):

The generated radical (**V**) can proceed along two competing pathways: (i) abstraction of an iodine atom from substrate **2** (XAT), thereby initiating a chain process, or (ii) recombination with the iodine radical formed in the initial photolysis step. The XAT pathway was found to be slightly endergonic (ΔG = +2.3 kcal/mol), with a relatively high transition state barrier (**TS2**, $\Delta G^{\ddagger}$ = 28.1 kcal/mol). This barrier is likely associated with regeneration of the less stable radical, rendering this pathway unfavorable. This outcome aligns with experimental light on/off studies, which suggest that an extended radical chain mechanism is unlikely. Furthermore, such a high barrier implies that this process wo uld be prohibitively slow at room temperature, whereas we observe rapid formation of intermediate (**VI**) at room temperature. In contrast, the alternative recombination of (**V**) with the iodine radical is strongly exergonic (ΔG = –35.8 kcal/mol). Although a discrete transition state for this recombination could not be located, we performed a relaxed potential energy surface scan of the distance between the radical-bearing carbon in (**V**) and the iodine radical, starting from 4.0 Å to the point at which a stable open-shell singlet wavefunction was obtained (3.2 Å). For distances shorter than 3.2 Å, no stable open shell singlet wavefunction was found. The resulting curve was smooth, with no detectable energy barrier, suggesting that this process may be effectively barrierless or only associated with a negligible entropic penalty. This interpretation is consistent with experimental observations, where recombination occurs rapidly, leading to efficient formation of (**VI**).

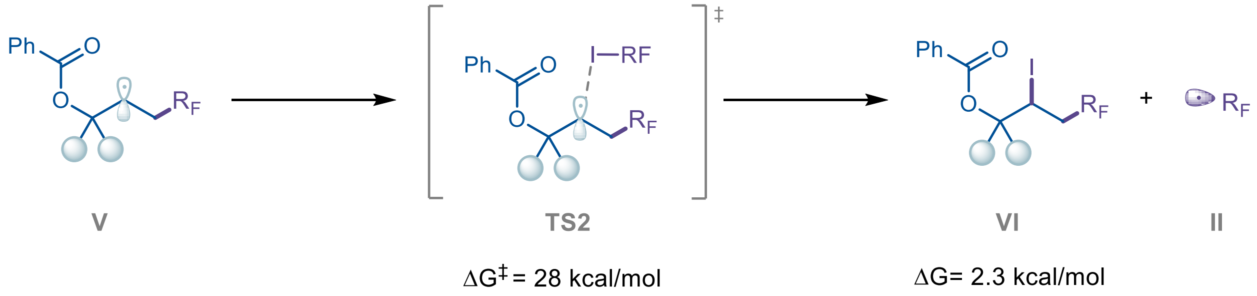

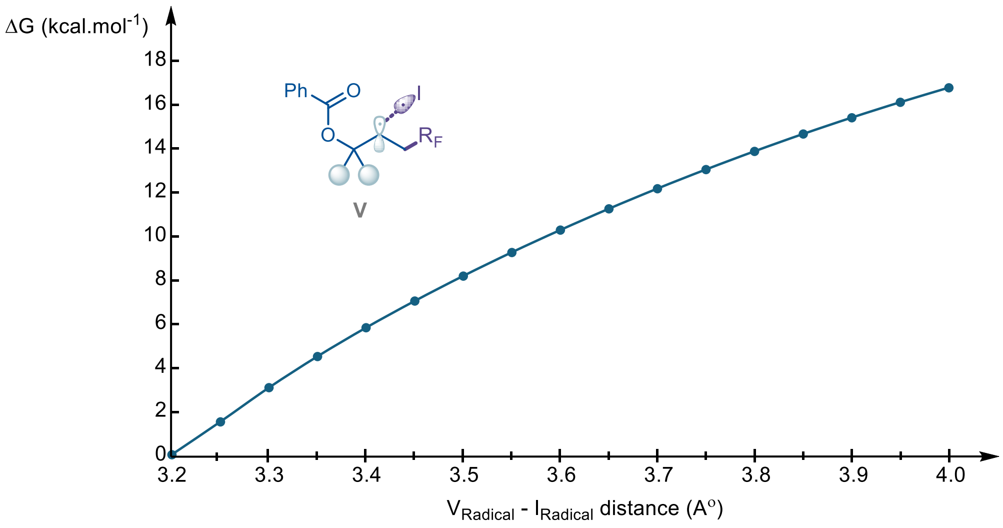

1,2-Cationic Acyloxy Migration (1,2-CAM) and Dioxolium Ring Opening:

Intermediate **VI** undergoes a 1,2-cationic acyloxy migration (**1,2-CAM**). In the optimized structure of **VI**, the iodine atom is antiperiplanar to the benzoate group, positioning the carbonyl oxygen to perform an intramolecular S_N2 displacement of iodide and generate a dioxolium ring intermediate (**VII**). This step is slightly endergonic ($\Delta G = +1.97$ kcal/mol), consistent with the formation of a charged species. However, the resulting carbocation is strongly stabilized by resonance delocalization over the phenyl ring and two oxygen atoms. Also, the oxygens' loan pairs are donating electrons to the empty p orbital through hyperconjugation. The energy barrier for this step was found to be 23.8 kcal/mol. The migration through formation of Oxiranium ring was also investigated but turned out to be kinetically inaccessible (**TS3'**, $\Delta G^{\ddagger} = 36.99$ kcal/mol).

From the calculated ΔG, the equilibrium constant (K) is estimated to be ~0.071, corresponding to a reactant-to-product ratio of ~14:1. This suggests that the system bottlenecks at this stage, with accumulation of **VI**. This prediction is consistent with experimental observations: **VI** forms rapidly in the early stages of the reaction, whereas its conversion to the final product occurs more slowly. Furthermore, both computation and experiment show that the formation of **VI** is highly favorable and proceeds readily at room temperature, yielding **VI** in high efficiency without the need for heating.

We examined potential pathways for conversion of intermediate **VII** into the final product, beginning with direct water attack at the benzylic position. However, the optimized structure consistently reverted to the stabilized carbocation, as water dissociated during optimization. This indicates that charge delocalization within the carbocationic framework is strongly favored over localization on a water adduct. Likewise, attempts to model nucleophilic attack by iodide showed the same behavior: iodine departed spontaneously during optimization, and the system relaxed back to the stabilized cation.

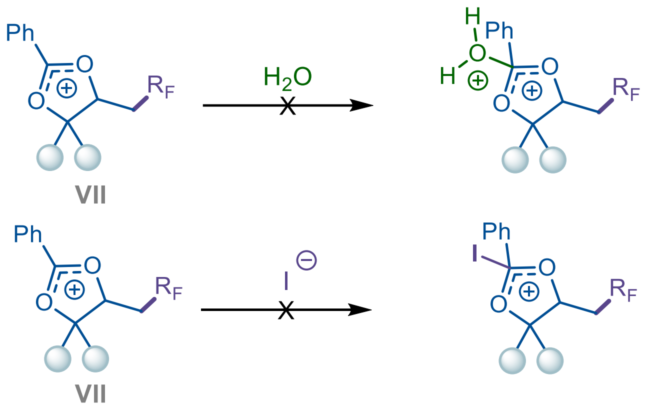

We also examined a potential ring-opening pathway involving the formation of a six-membered transition state with two water molecules bridging between the benzylic carbon and an oxygen atom. This pathway was found to be kinetically inaccessible.

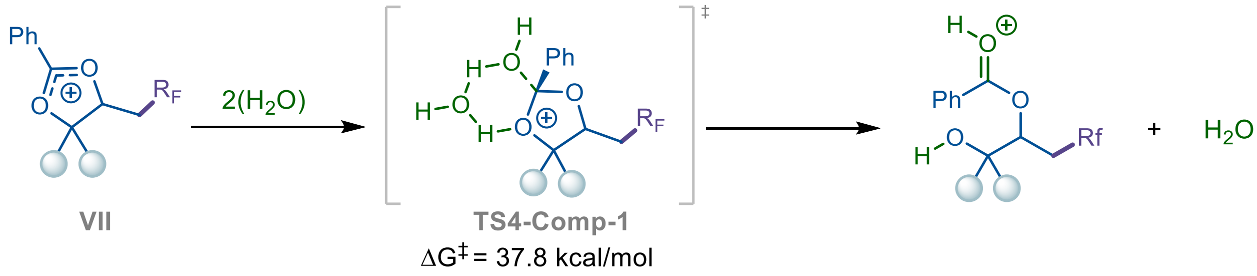

We found that carbonate base can activate water through the formation of a six-membered transition state complex involving one water molecule. In this concerted process, water adds to the benzylic position while being deprotonated, leading to hydroxyl incorporation and release of bicarbonate as a byproduct. This step is thermodynamically favorable ($\Delta G = -14.5$ kcal/mol) and kinetically accessible, with the corresponding transition state (**TS4**) located at a moderate barrier of 13.7 kcal/mol. Notably, sodium ions were found to significantly stabilize **TS4** by coordinating to the carbonyl oxygen and fluorine atoms of the ester as well as the phenyl ring. This mechanistic model is consistent with ^{18}O-labeling experiments, which confirm incorporation of water at the benzylic position.

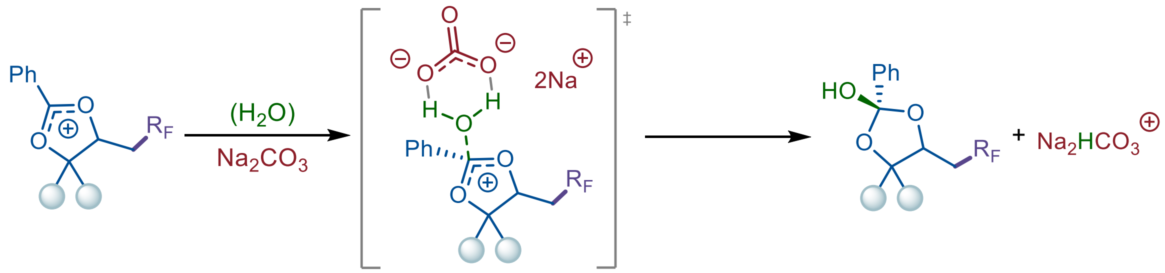

VII

TS4

$\Delta G^{\ddagger}$ = 13.7 kcal/mol

VIII

ΔG= -14.5 kcal/mol

Finally, intermediate **VIII** undergoes $NaHCO_3$-assisted ring opening and proton transfer to deliver the desired and kinetically favored 1,3-product (**3**) via **TS5** ($\Delta G^{\ddagger}$ = 15.7 kcal/mol) in an overall thermodynamically favorable step (ΔG = –13.95 kcal/mol). In contrast, the competing ring-opening pathway leading to the 1,2-product (**3a'**) is kinetically disfavored, exhibiting a higher barrier (**TS5'**, $\Delta G^{\ddagger}$ = 18.2 kcal/mol). Moreover, product **3a'** is also thermodynamically less stable by 0.44 kcal/mol (ΔG = –13.5 kcal/mol) compared to **3a**. These computational findings are consistent with experimental observations, where only the 1,3-product (**3a**) was obtained.

Spectroscopic Data

^{1}H NMR (500 MHz, $CDCl_3$, 25 ºC) of (2l)

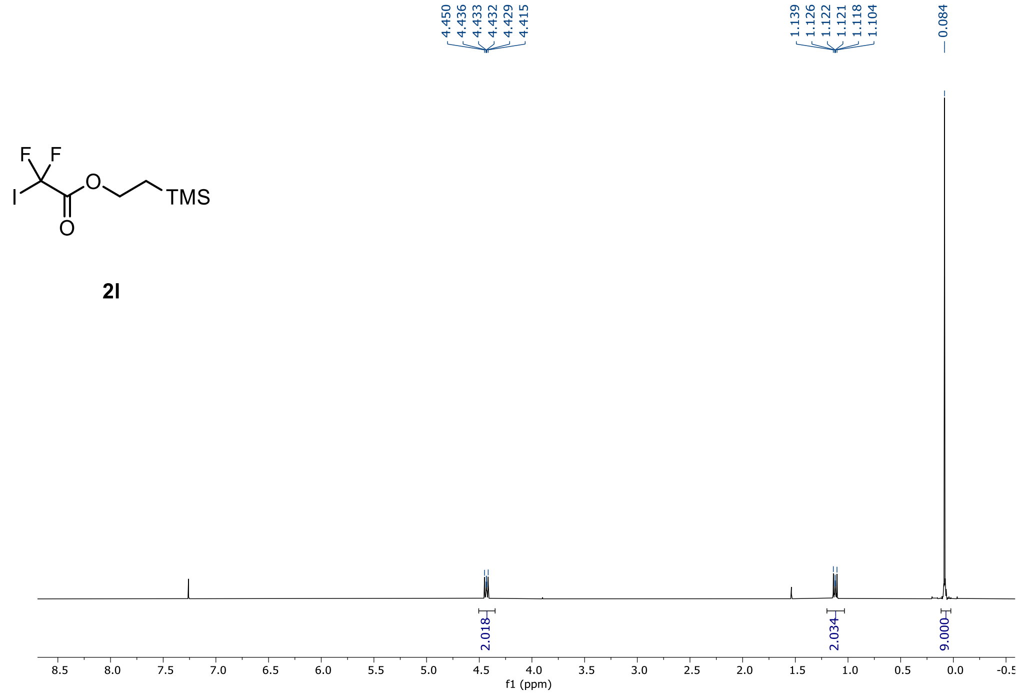

^{13}C NMR (126 MHz, $CDCl_3$, 25 ºC) of (2l)

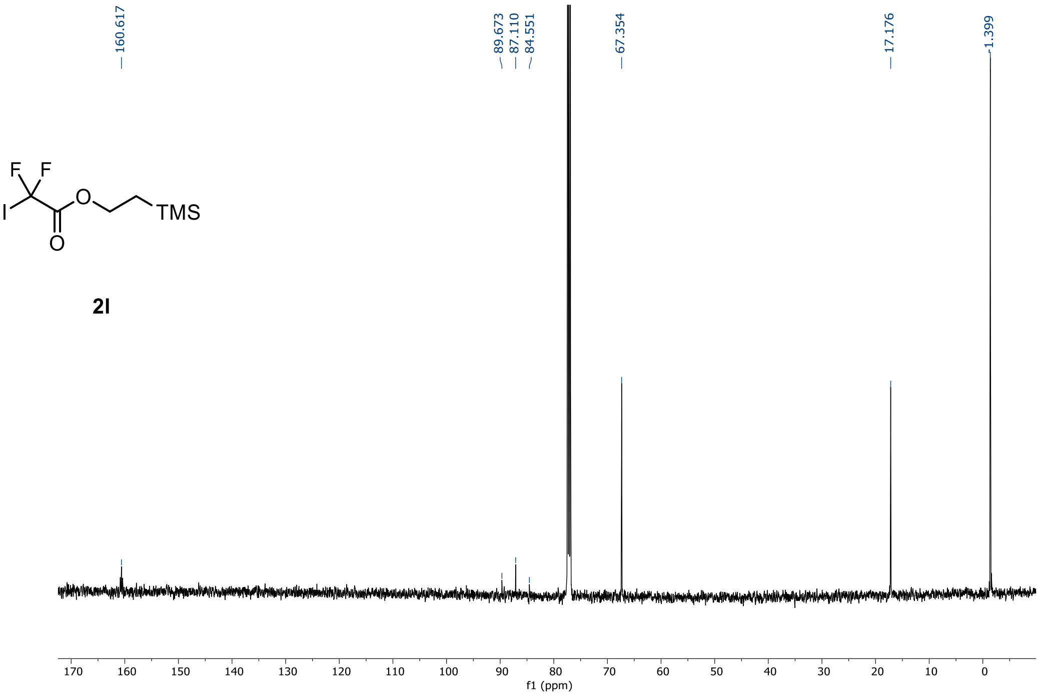

^{19}F NMR (470 MHz, $CDCl_3$, 25 ºC) of (2l)

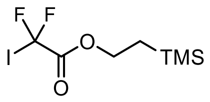

2l

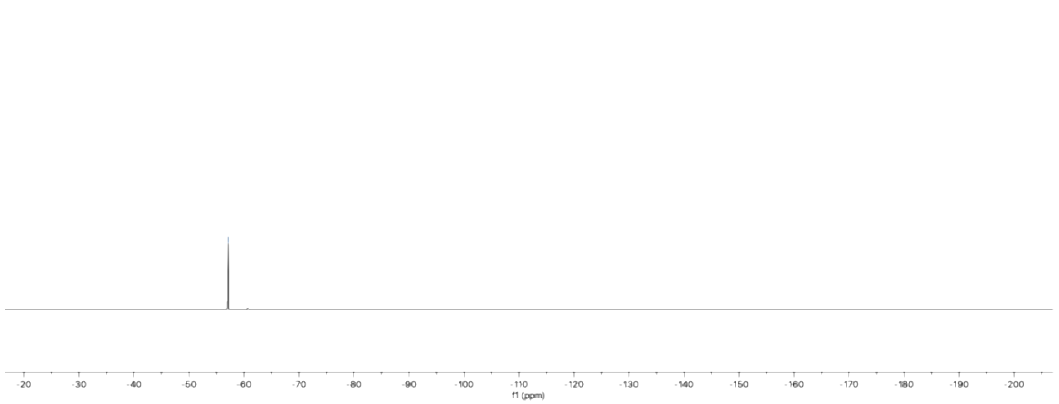

^{1}H NMR (500 MHz, $CDCl_3$, 25 ºC) of (2m)

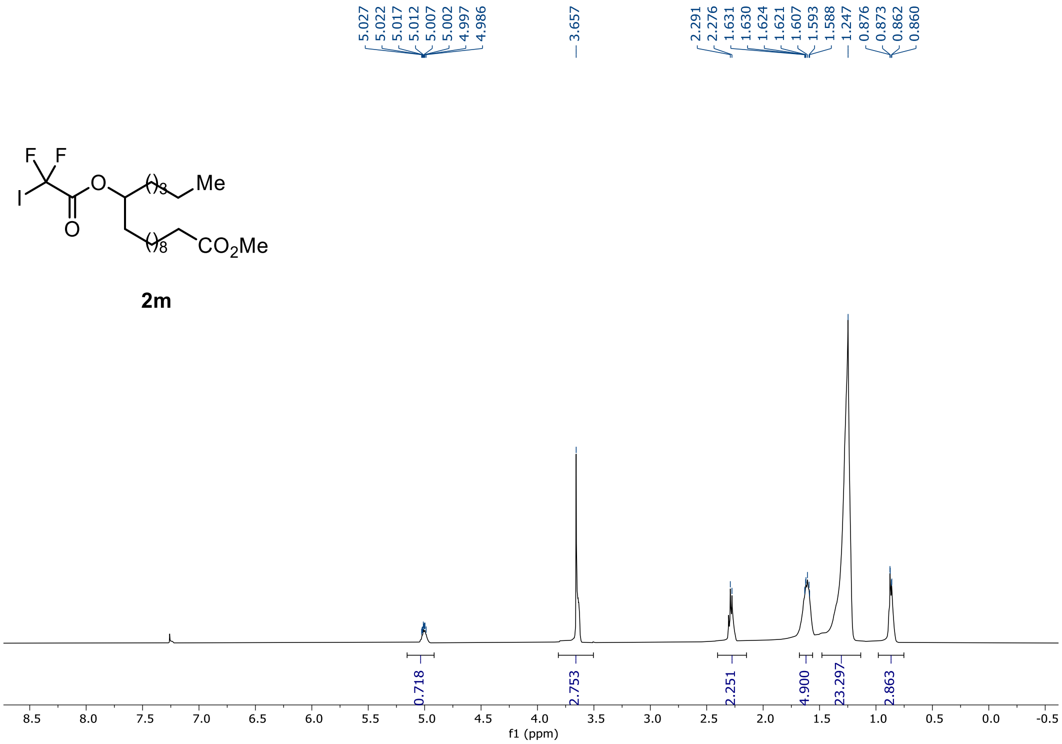

^{13}C NMR (126 MHz, $CDCl_3$, 25 ºC) of (2m)

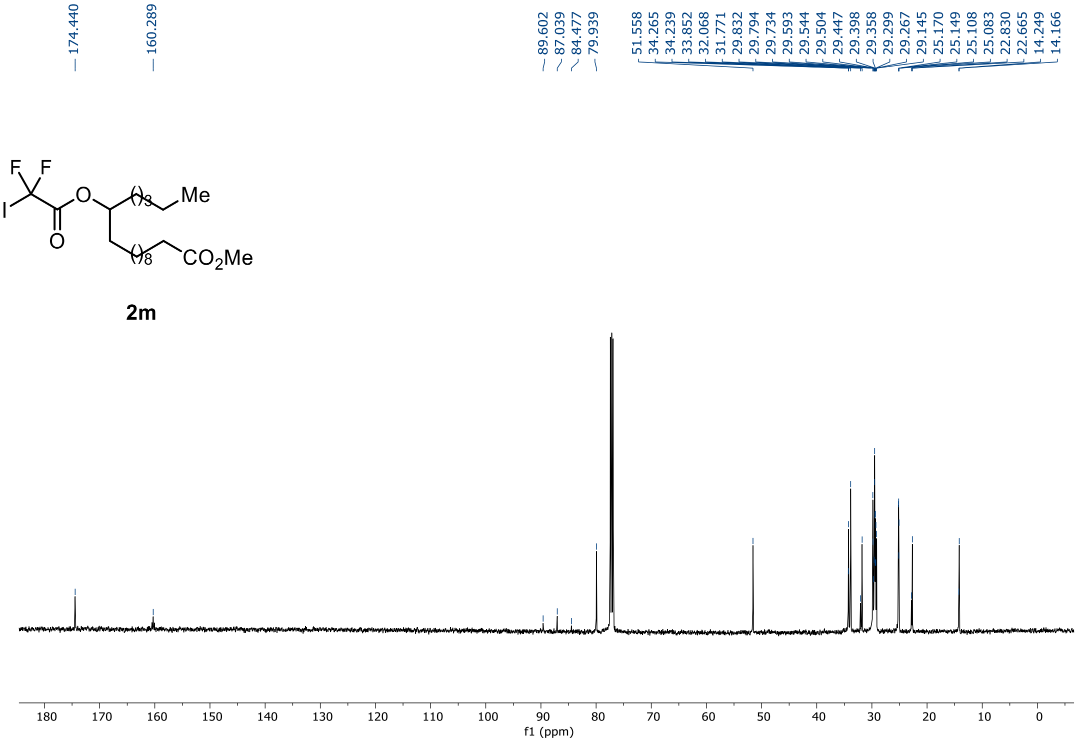

^{19}F NMR (470 MHz, $CDCl_3$, 25 ºC) of (2m)

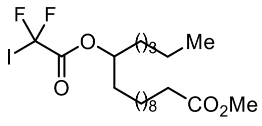

2m

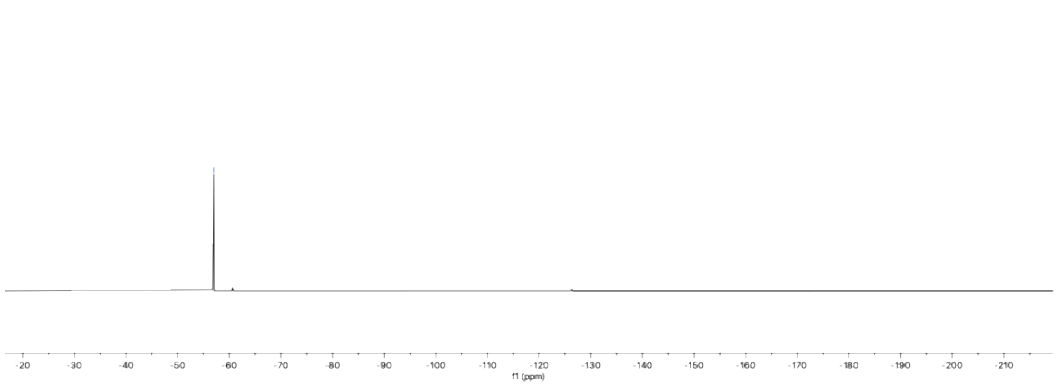

^{1}H NMR (500 MHz, $CDCl_3$, 25 ºC) of (2n)

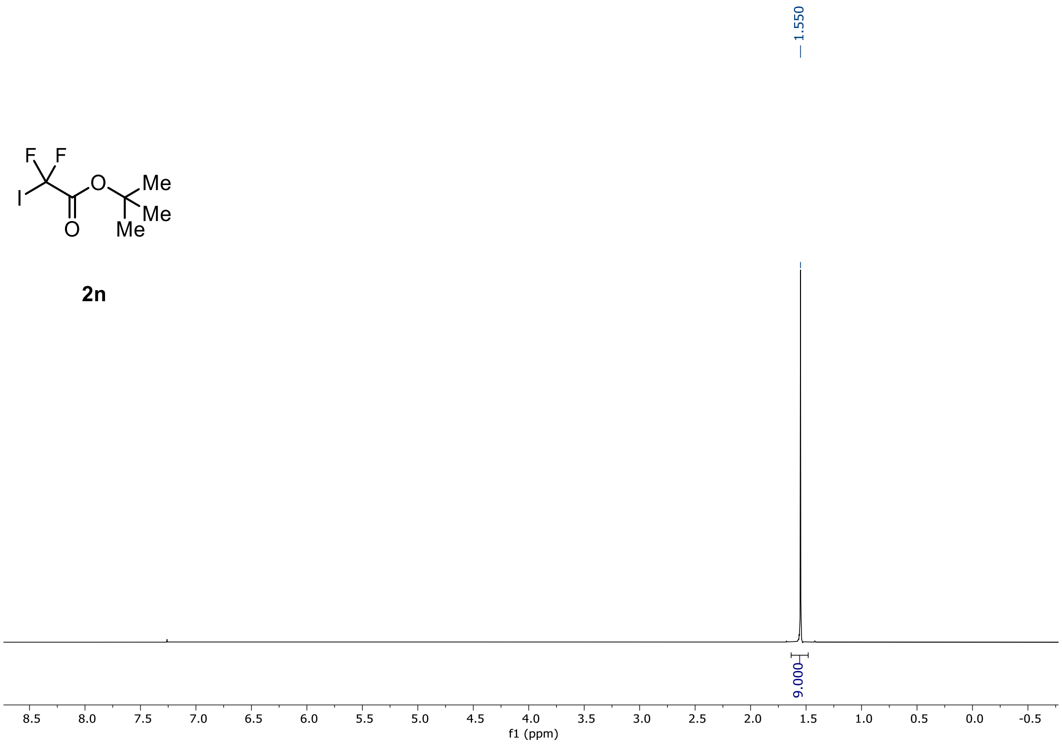

^{13}C NMR (126 MHz, $CDCl_3$, 25 ºC) of (2n)

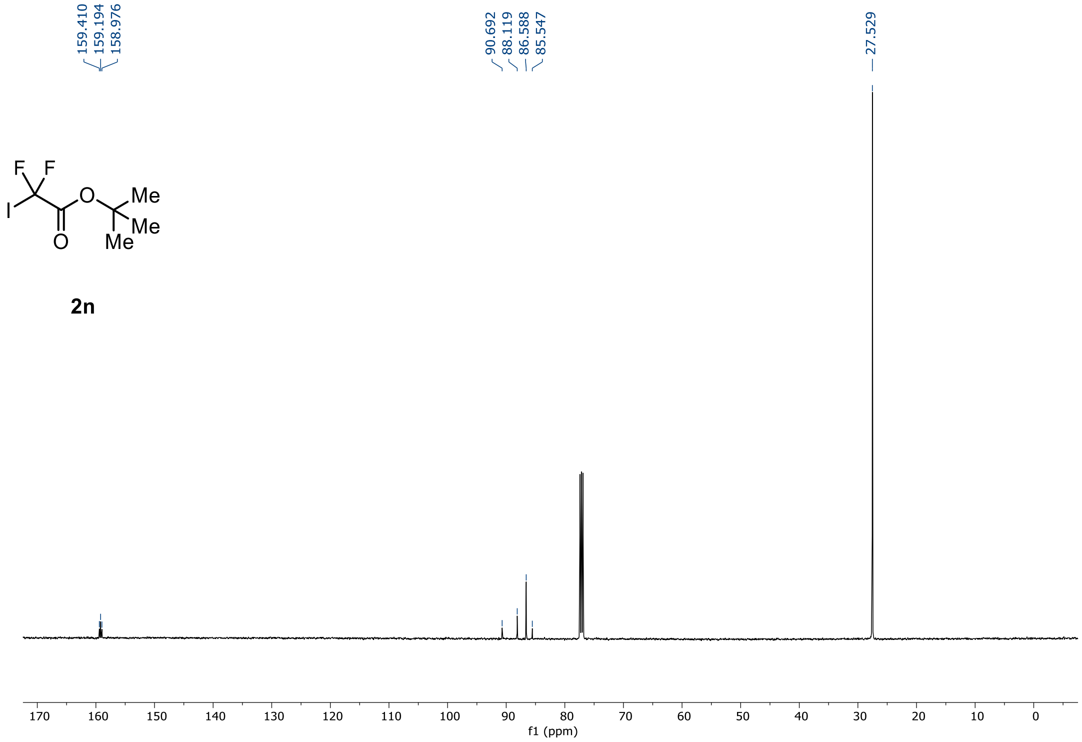

^{19}F NMR (470 MHz, CDCl$_3$, 25 ºC) of (2n)

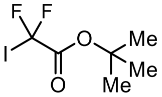

2n

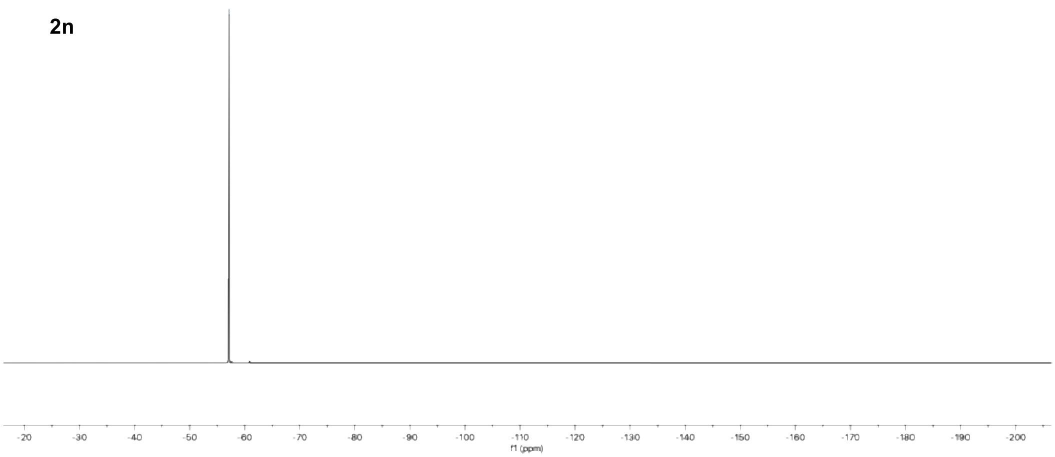

^{1}H NMR (500 MHz, CDCl$_3$**, 25 ºC) of (2o)**

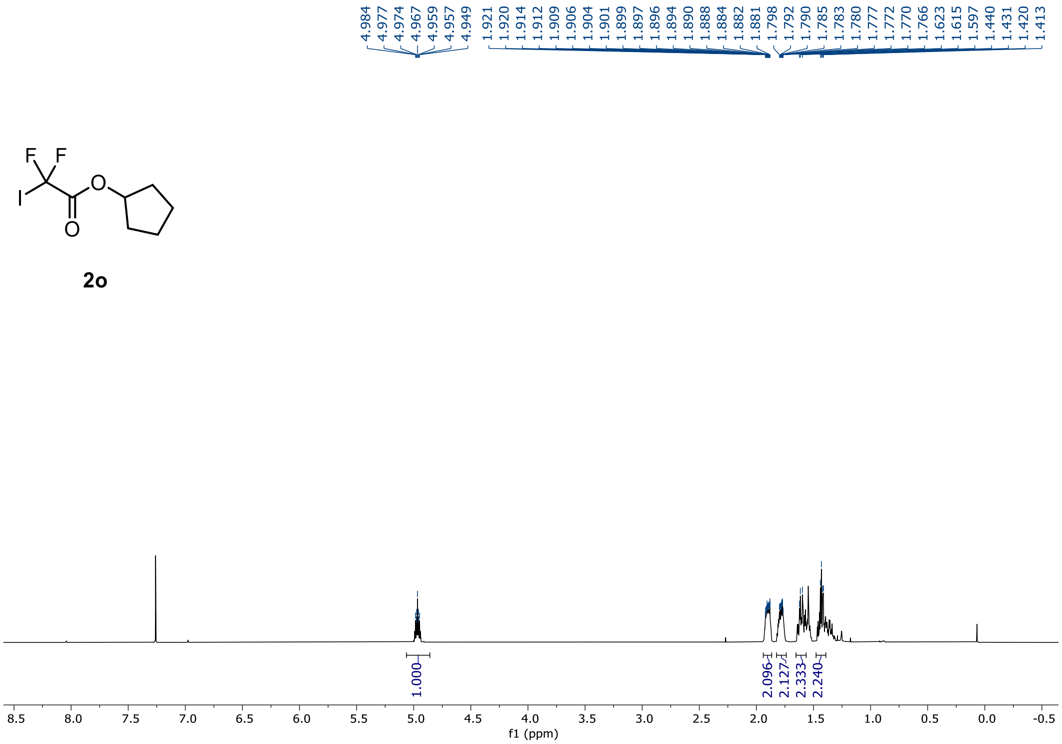

^{13}C NMR (126 MHz, CDCl$_3$**, 25 ºC) of (2o)**

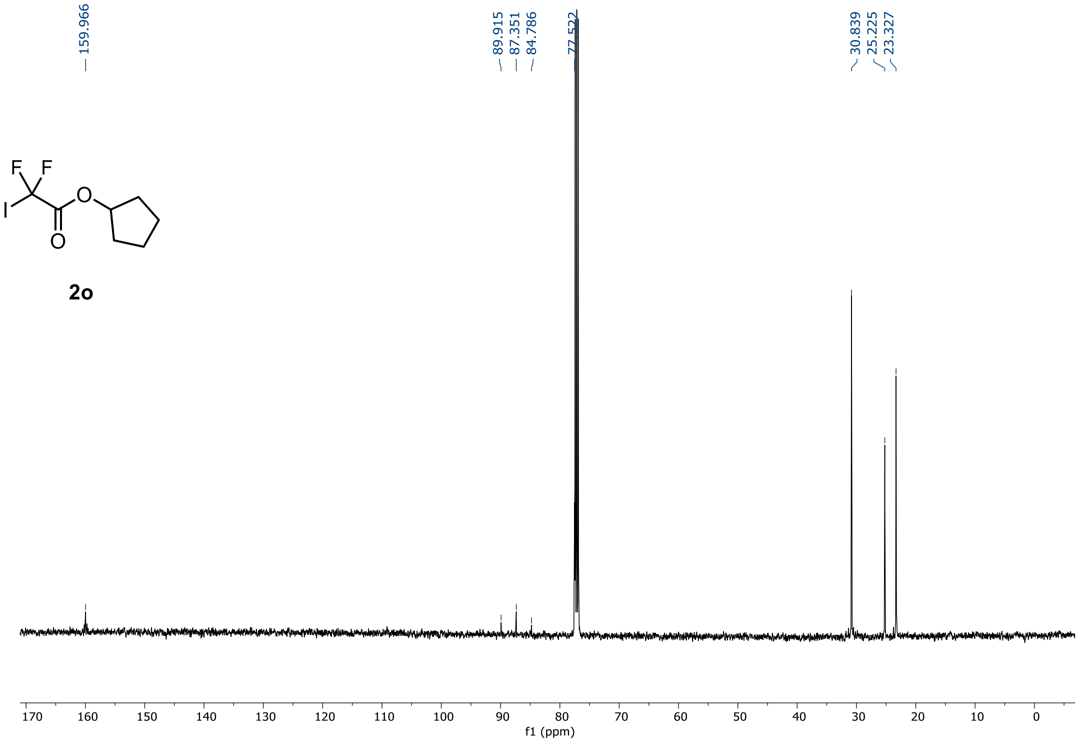

^{19}F NMR (470 MHz, $CDCl_3$**, 25 ºC) of (2o)**

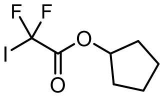

2o

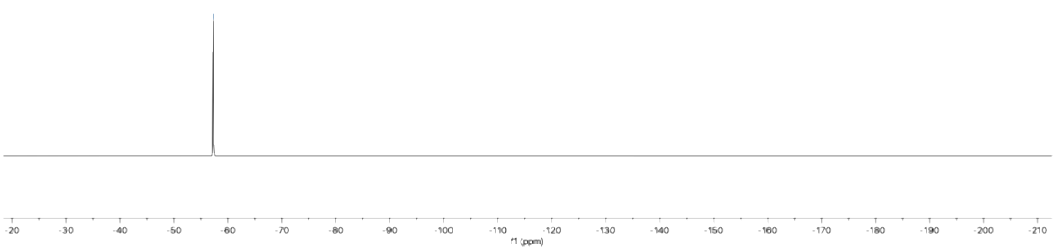

^{1}H NMR (500 MHz, $CDCl_3$, 25 ºC) of (2p)

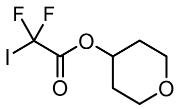

2p

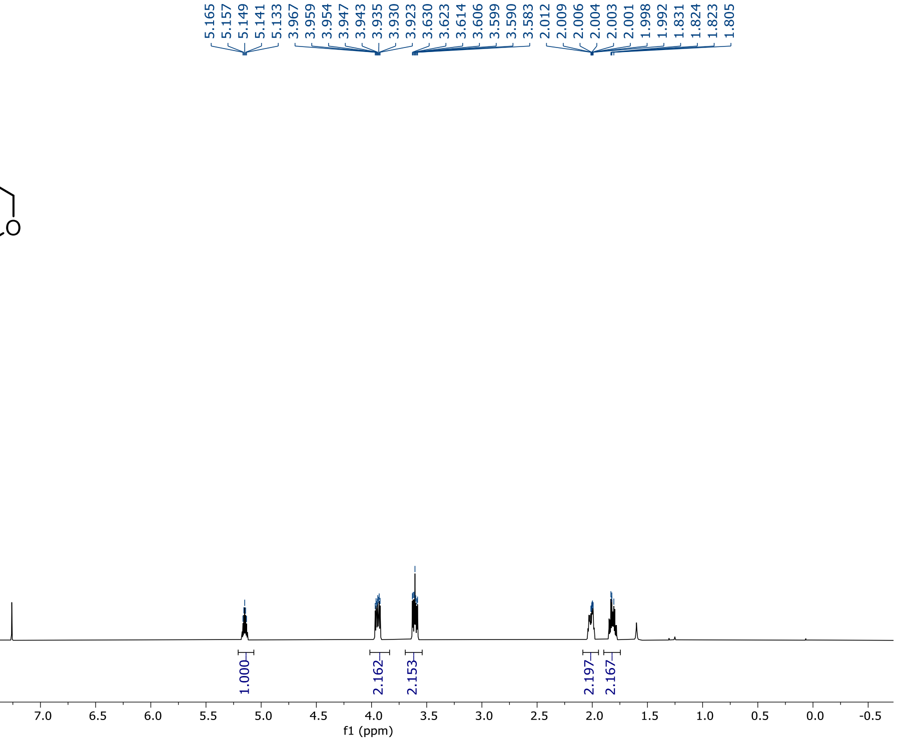

^{13}C (126 MHz, $CDCl_3$, 25 ºC) of (2p)

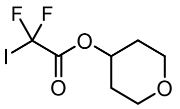

2p

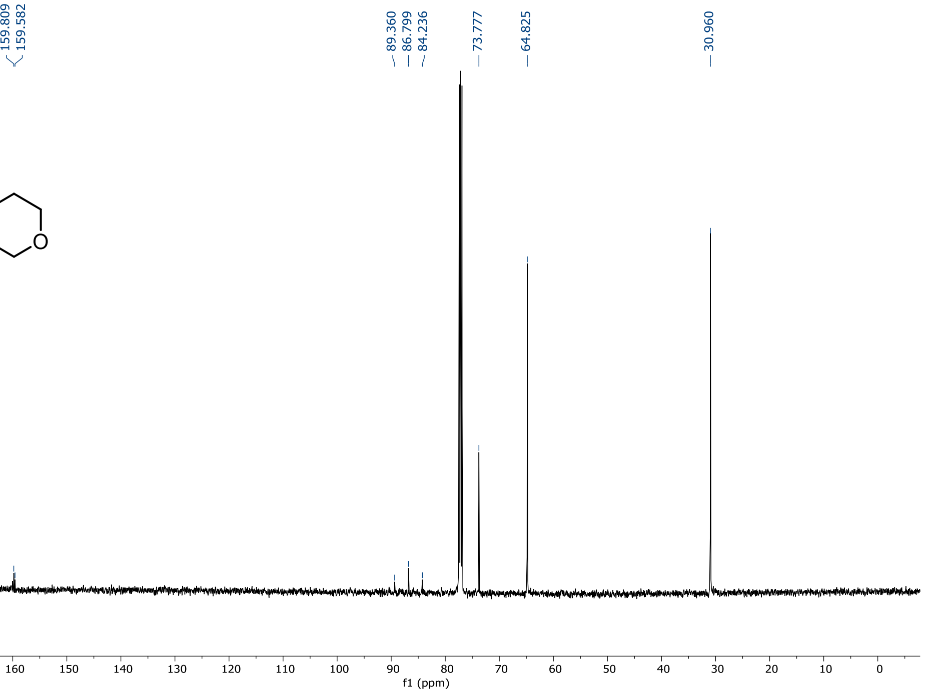

^{19}F NMR (470 MHz, $CDCl_3$**, 25 ºC) of (2p)**

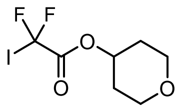

2p

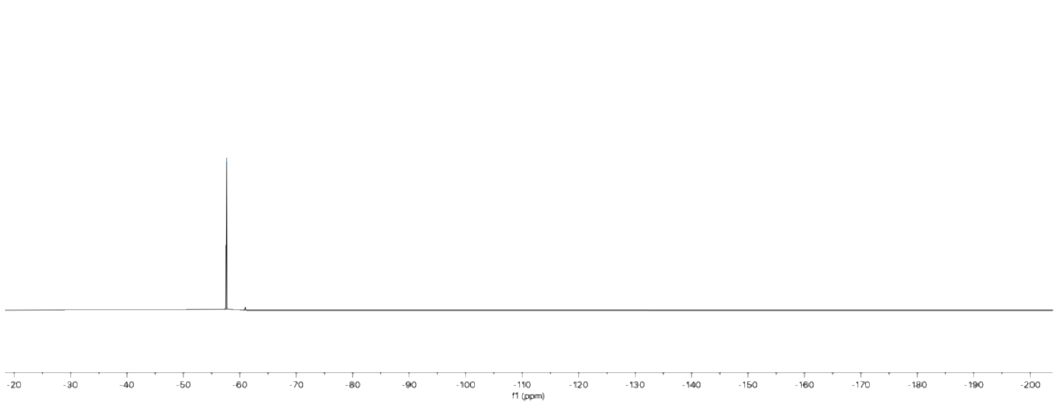

^{1}H NMR (500 MHz, CDCl$_3$, 25 ºC) of (2q)

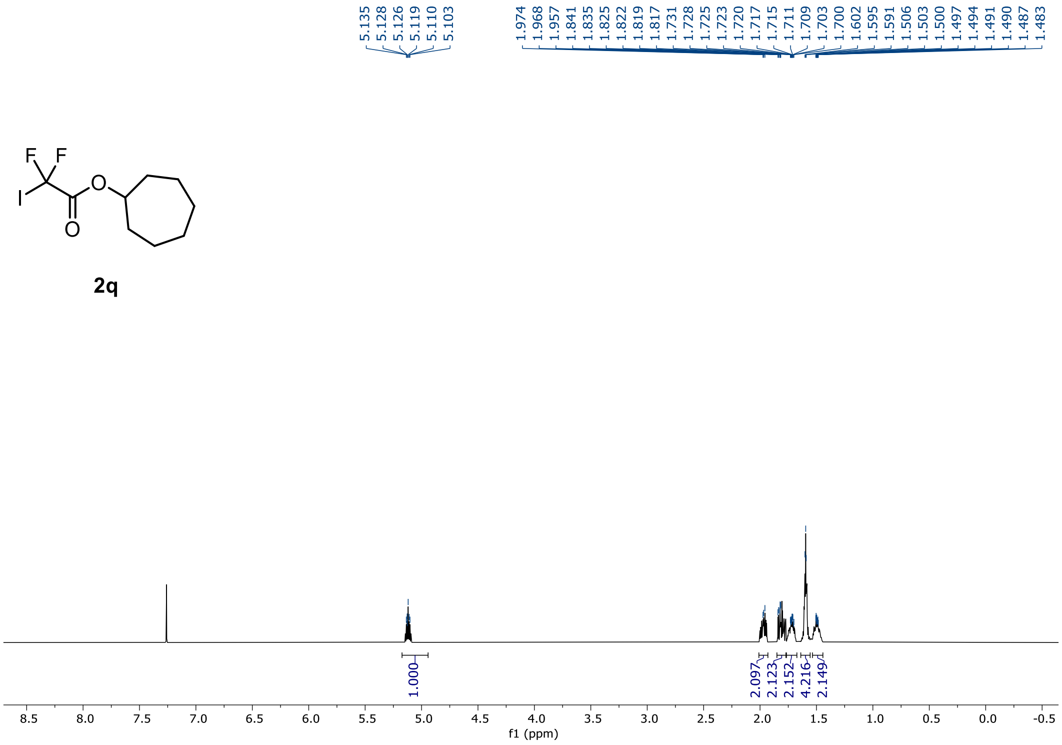

2q

^{13}C NMR (126 MHz, CDCl$_3$, 25 ºC) of (2q)

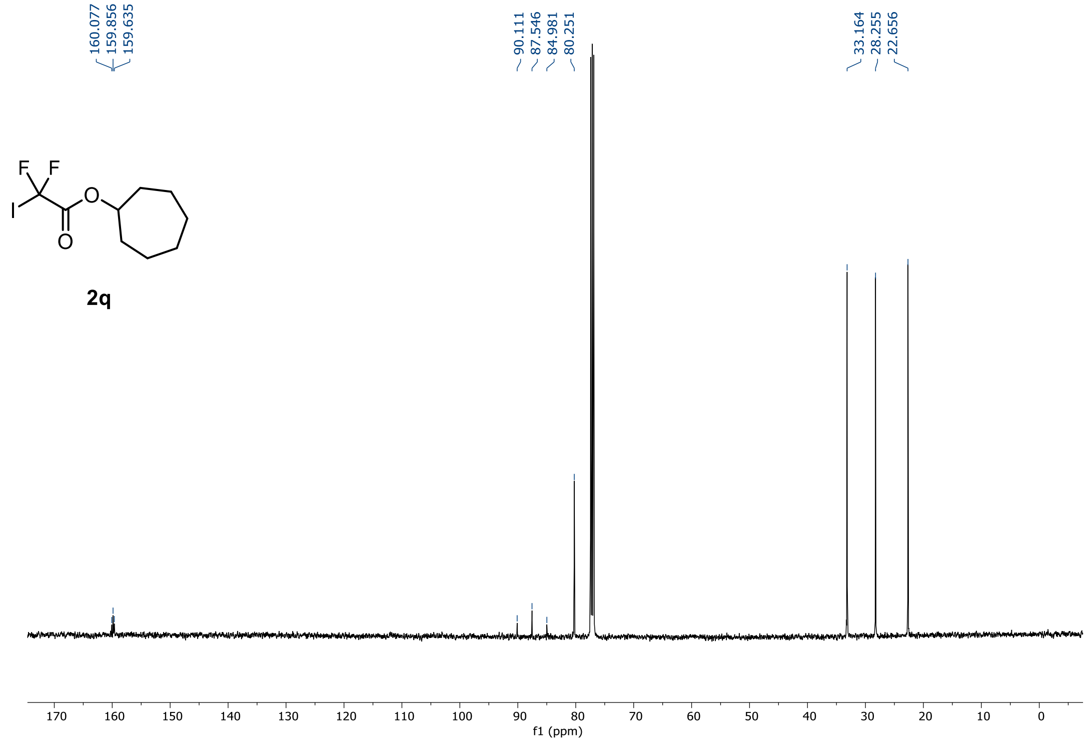

2q

^{19}F NMR (470 MHz, $CDCl_3$, 25 ºC) of (2q)

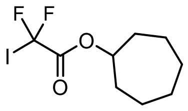

2q

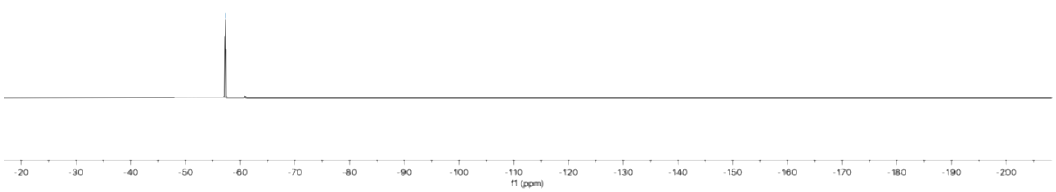

^{1}H NMR (500 MHz, $CDCl_3$, 25 ºC) of (2r)

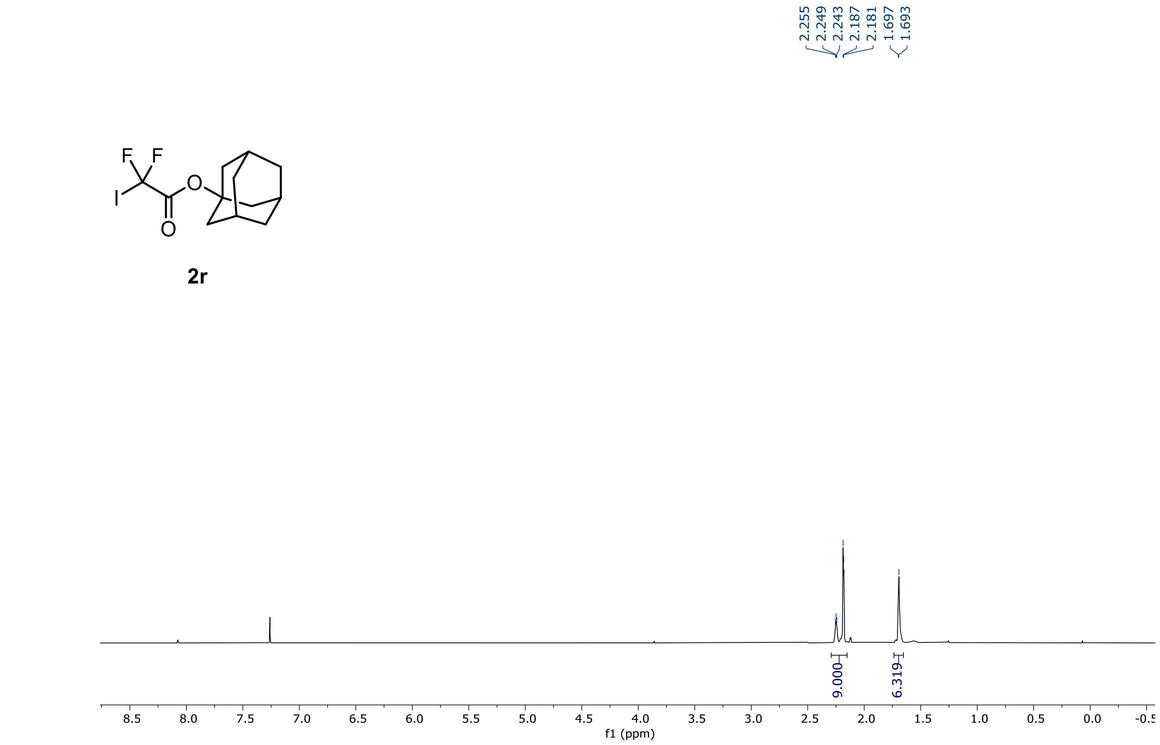

^{13}C NMR (126 MHz, $CDCl_3$, 25 ºC) of (2r)

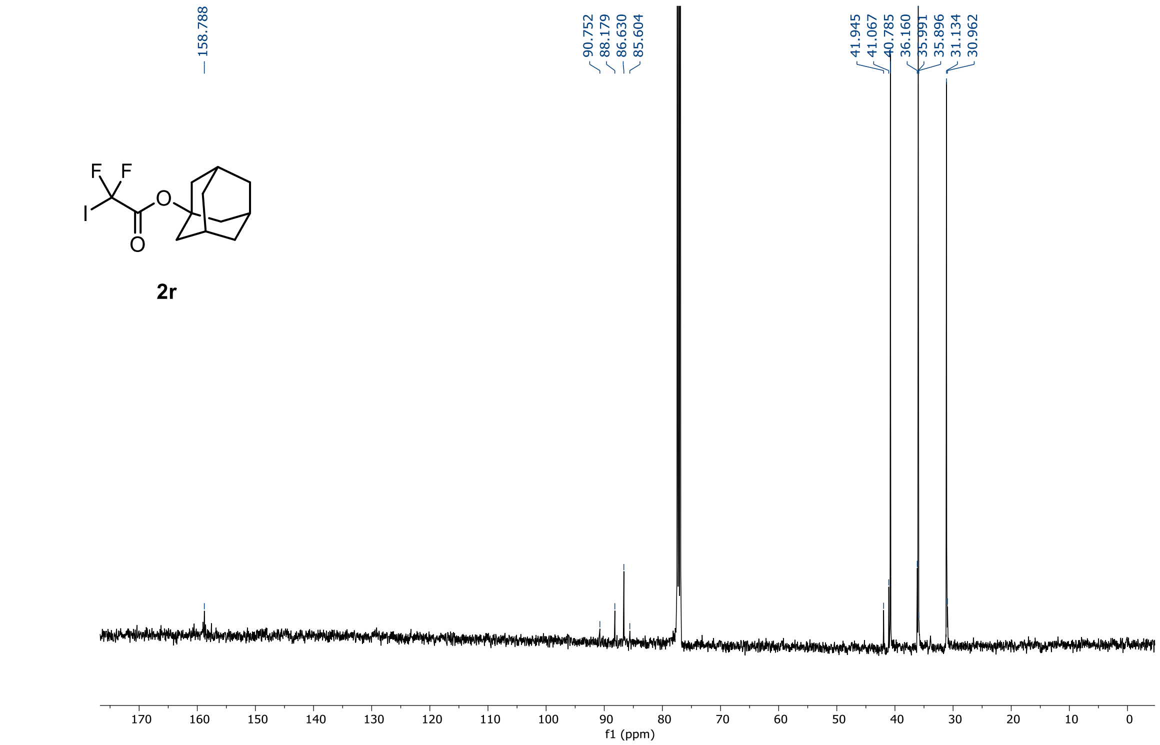

^{19}F NMR (470 MHz, $CDCl_3$**, 25 ºC) of (2r)**

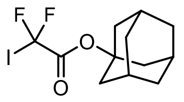

2r

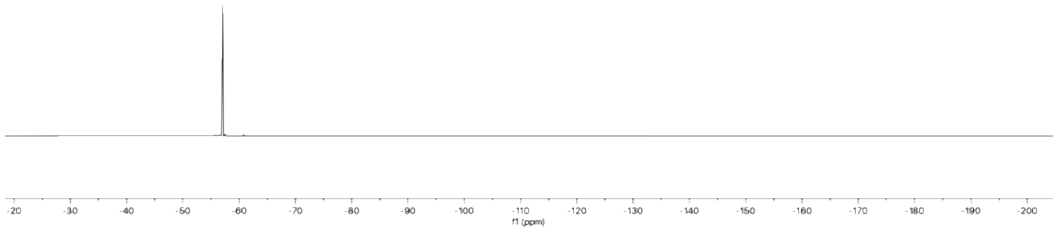

^{1}H NMR (500 MHz, $CDCl_3$, 25 ºC) of (2s)

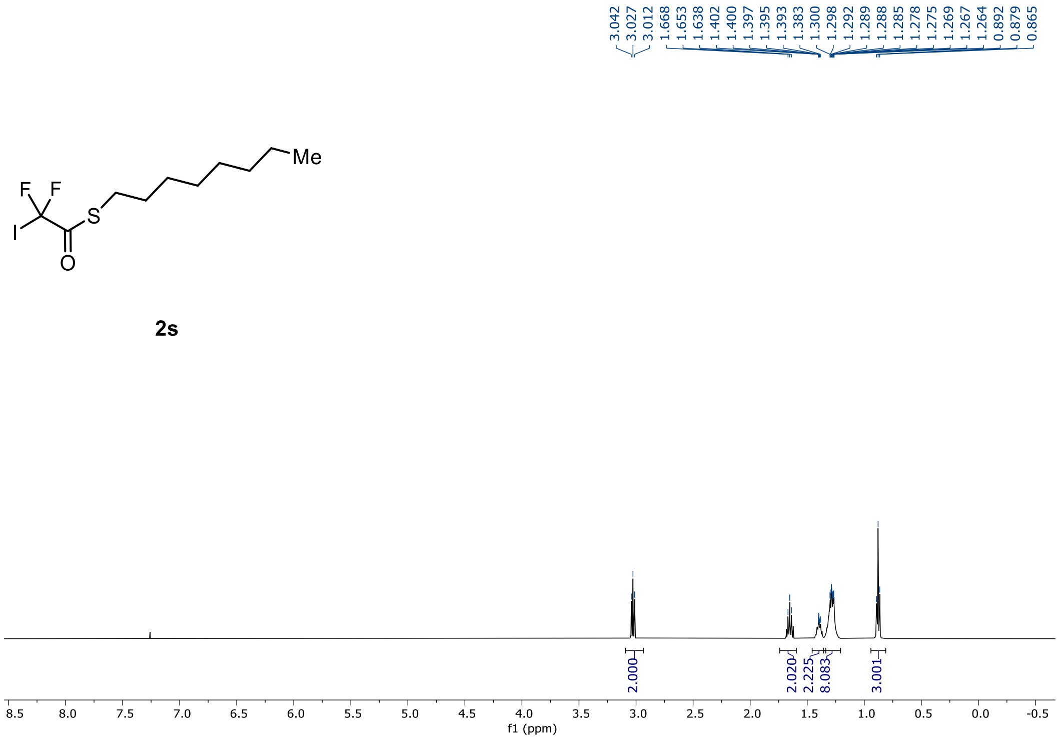

2s

^{13}C NMR (126 MHz, $CDCl_3$, 25 ºC) of (2s)

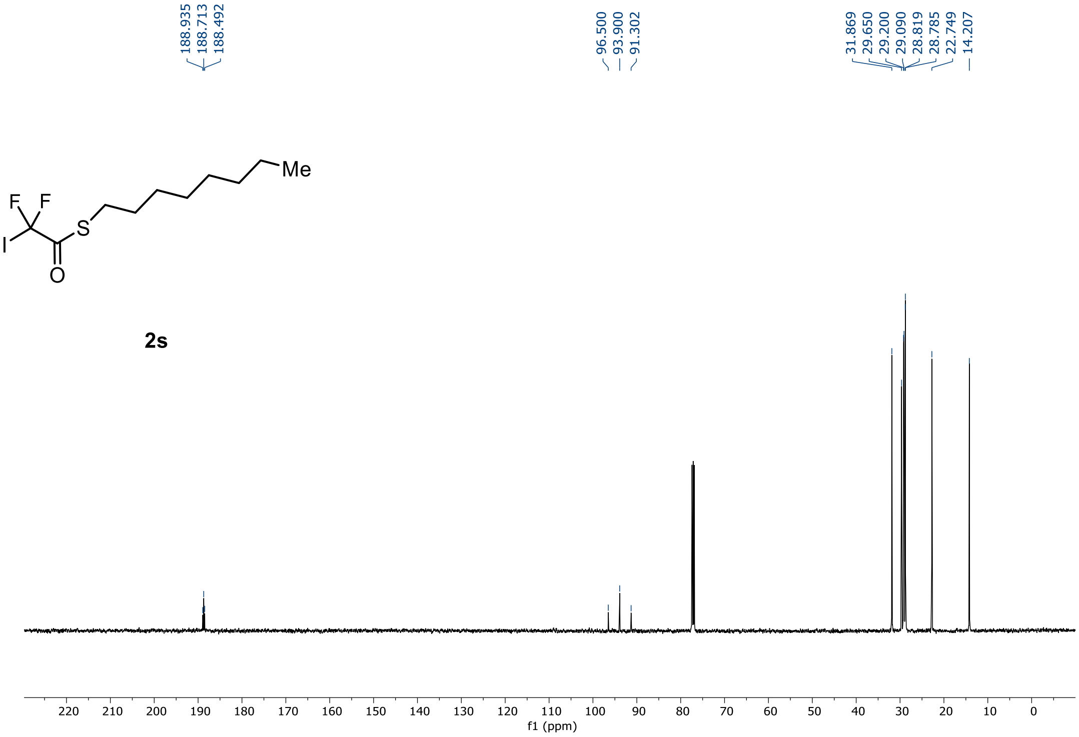

2s

^{19}F NMR (470 MHz, $CDCl_3$**, 25 ºC) of (2s)**

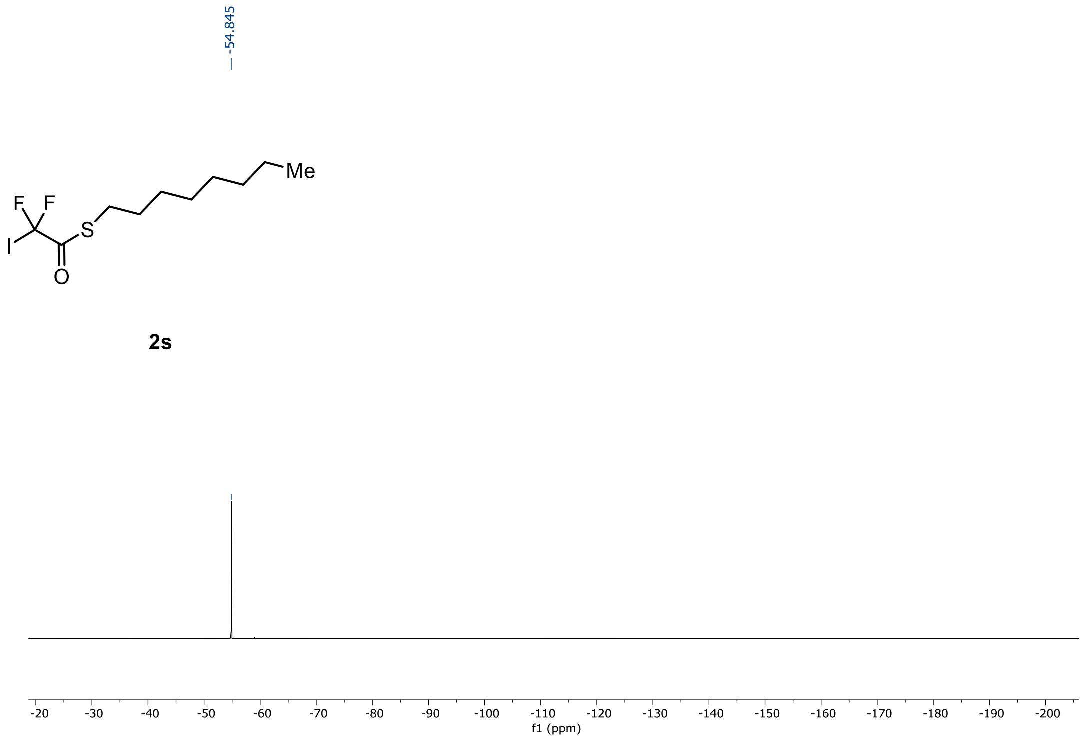

2s

^{1}H NMR (500 MHz, $CDCl_3$, 25 ºC) of (2t)

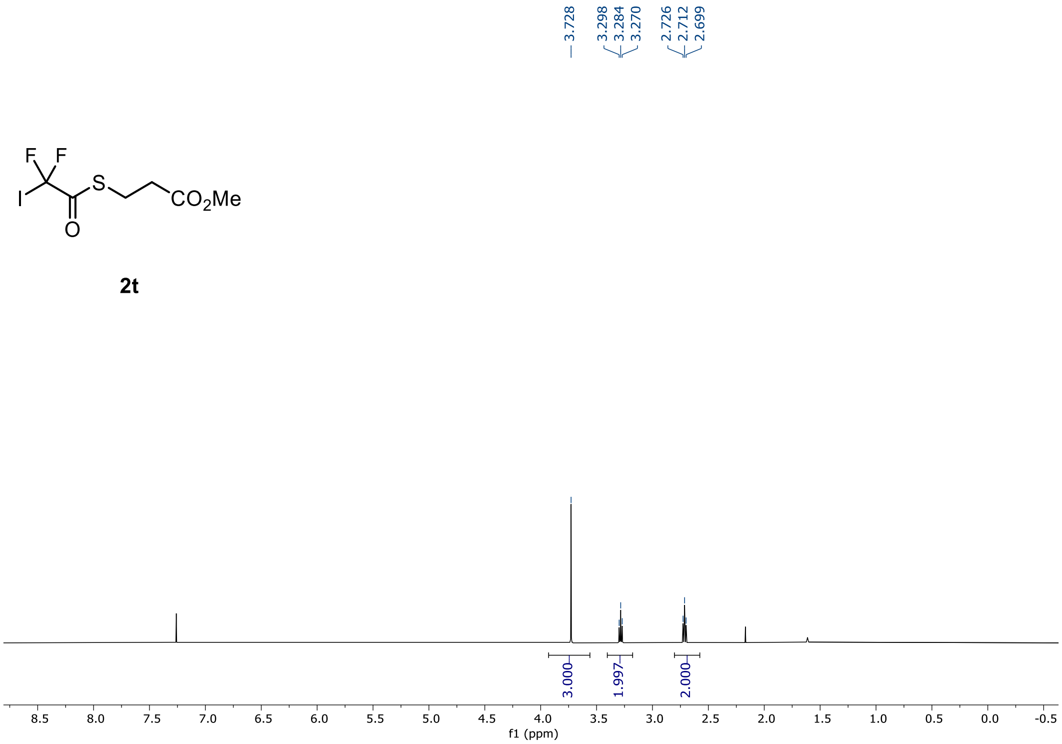

^{13}C NMR (126 MHz, $CDCl_3$, 25 ºC) of (2t)

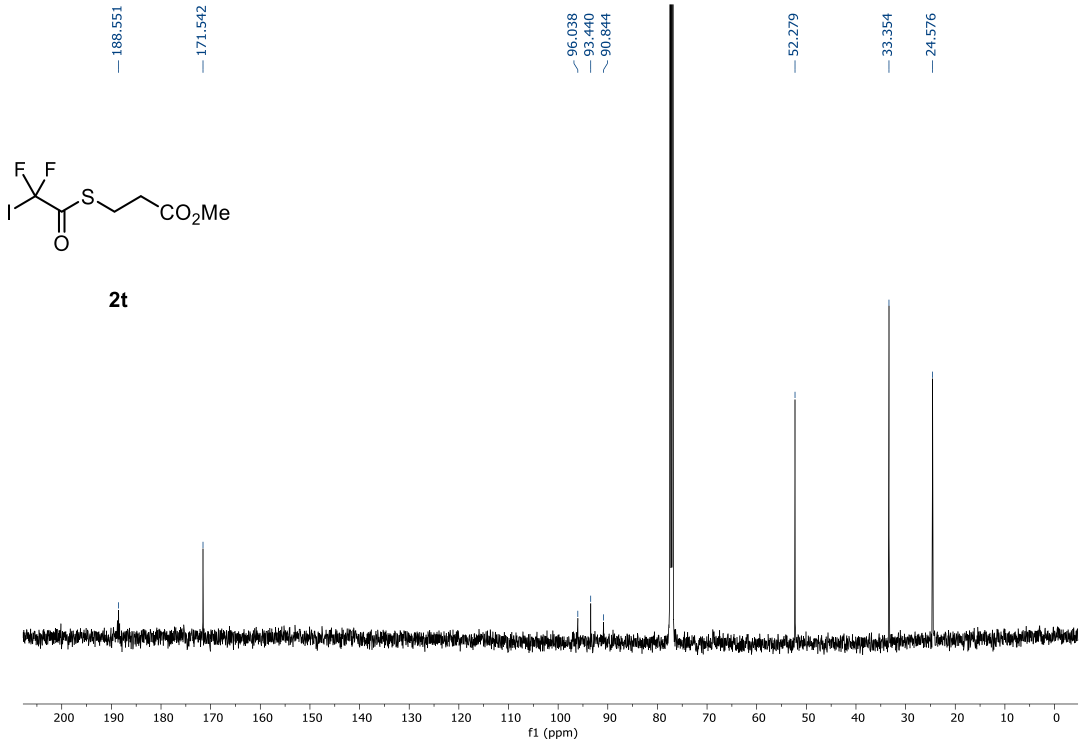

^{19}F NMR (470 MHz, $CDCl_3$**, 25 ºC) of (2t)**

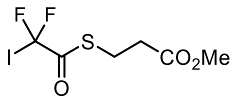

2t

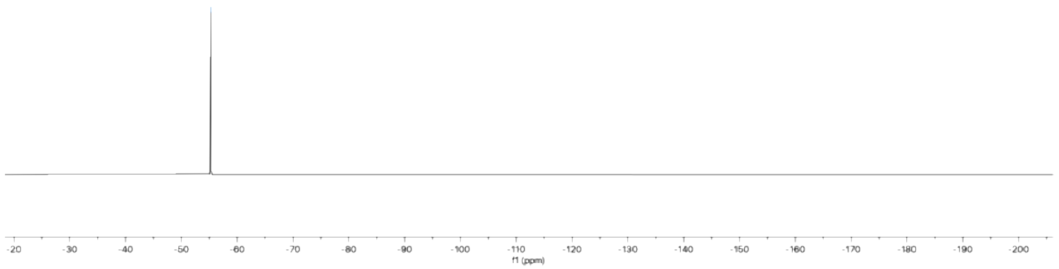

^{1}H NMR (500 MHz, $CDCl_3$, 25 ºC) of (2u)

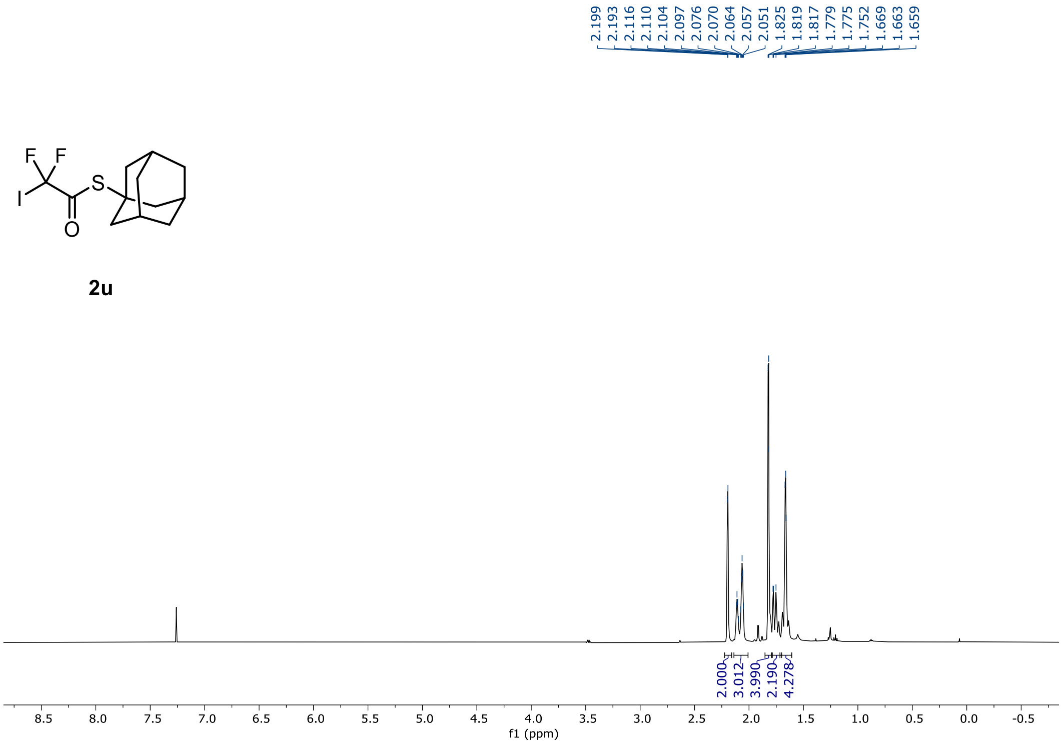

^{13}C NMR (126 MHz, $CDCl_3$, 25 ºC) of (2u)

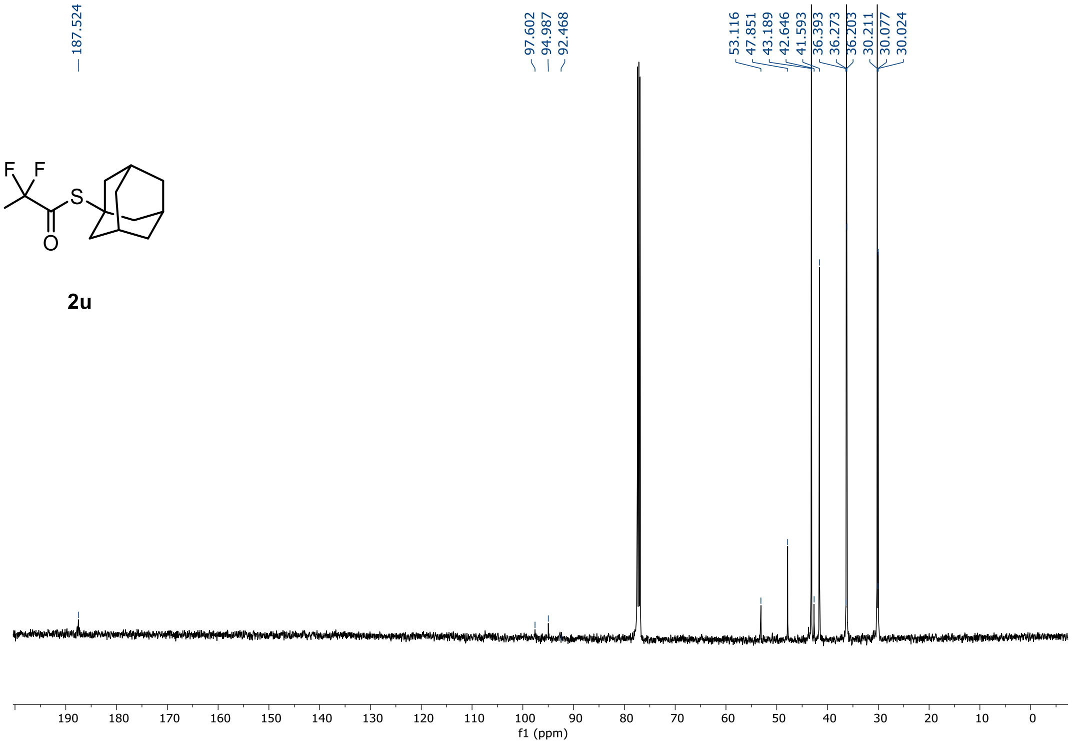

^{19}F NMR (470 MHz, $CDCl_3$**, 25 ºC) of (2u)**

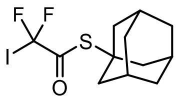

2u

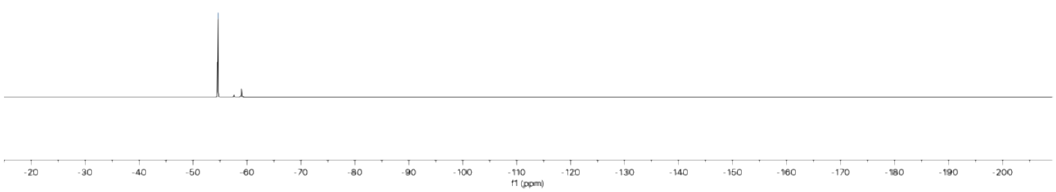

^{1}H NMR (500 MHz, CDCl$_3$**, 25 ºC) of (2v)**

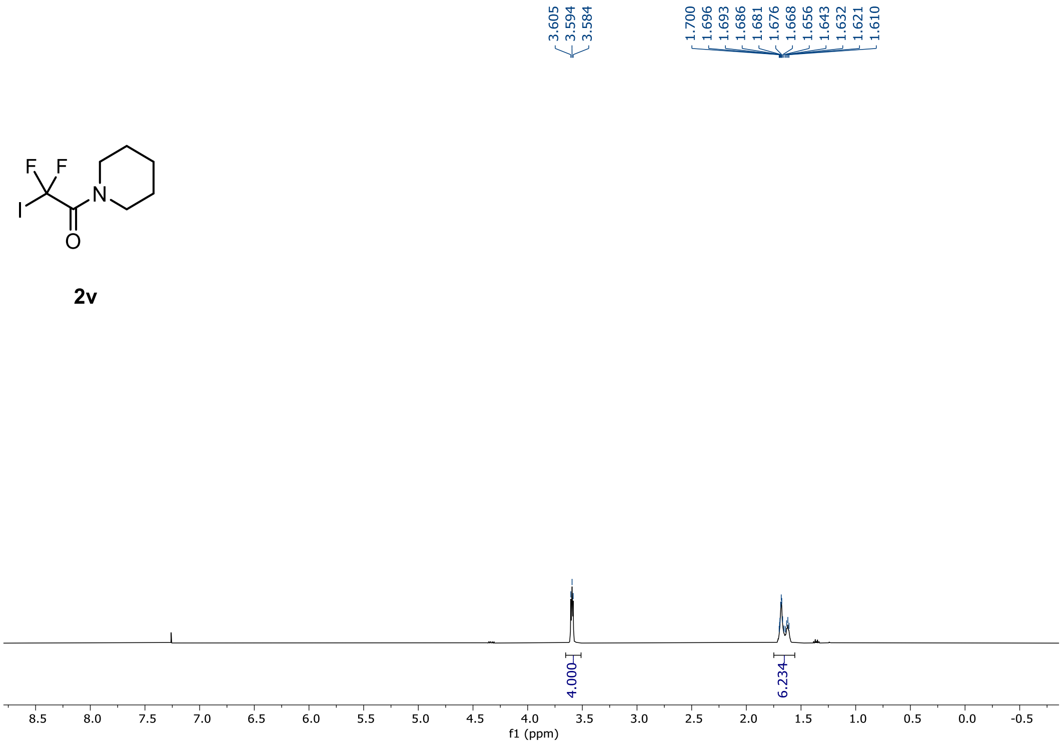

^{13}C NMR (126 MHz, CDCl$_3$**, 25 ºC) of (2v)**

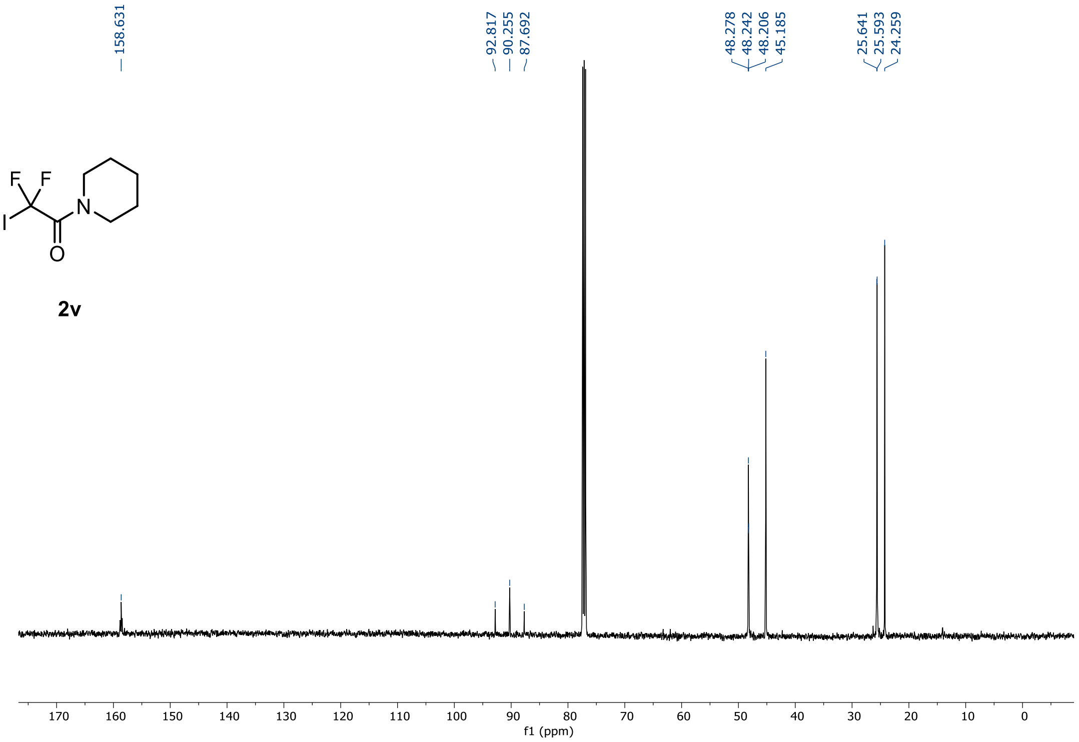

^{19}F NMR (470 MHz, $CDCl_3$**, 25 ºC) of (2v)**

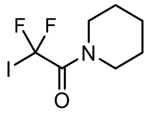

2v

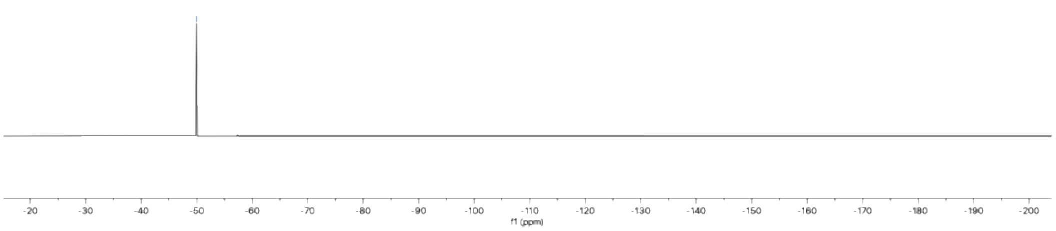

^{1}H NMR (500 MHz, $CDCl_3$, 25 ºC) of (2w)

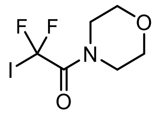

2w

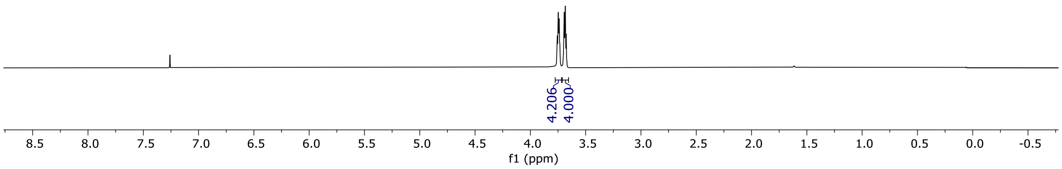

^{13}C NMR (126 MHz, $CDCl_3$, 25 ºC) of (2w)

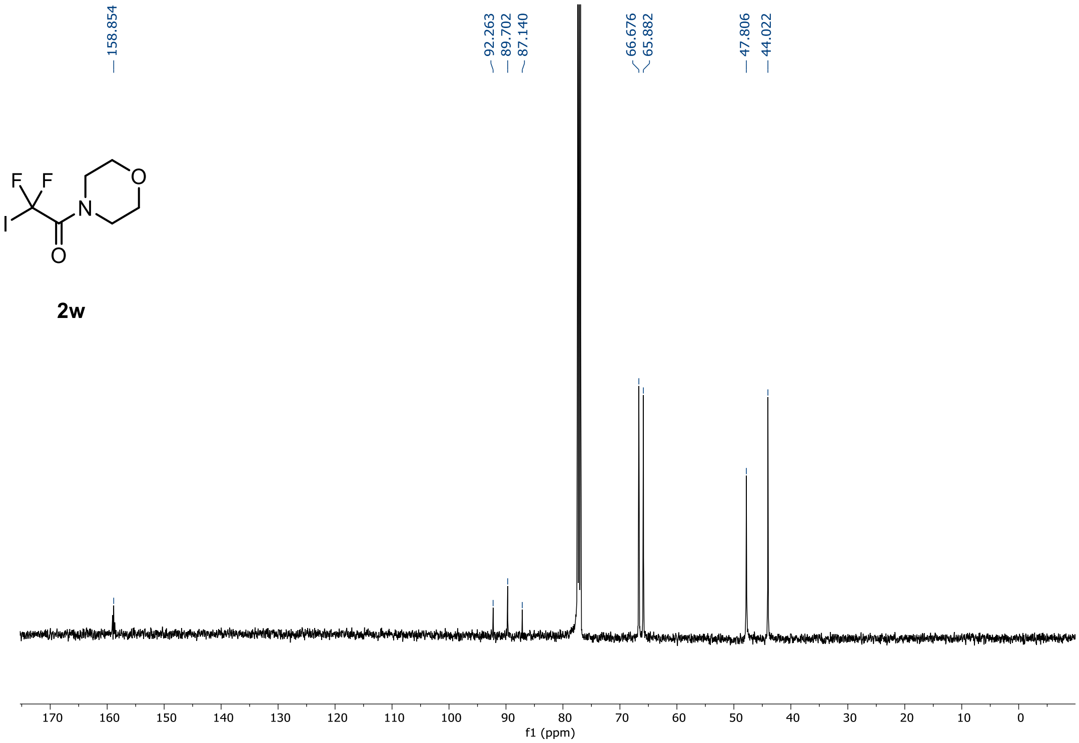

^{19}F NMR (470 MHz, $CDCl_3$**, 25 ºC) of (2w)**

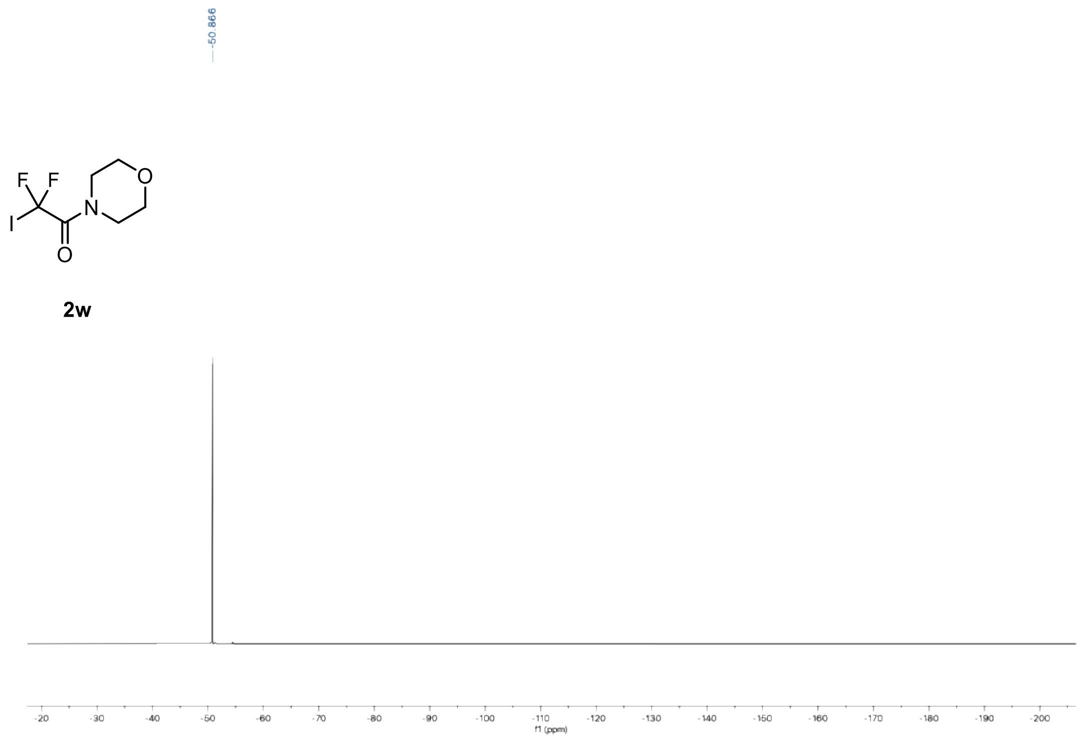

^{1}H NMR (500 MHz, CDCl3, **25 ºC) of (2x)**

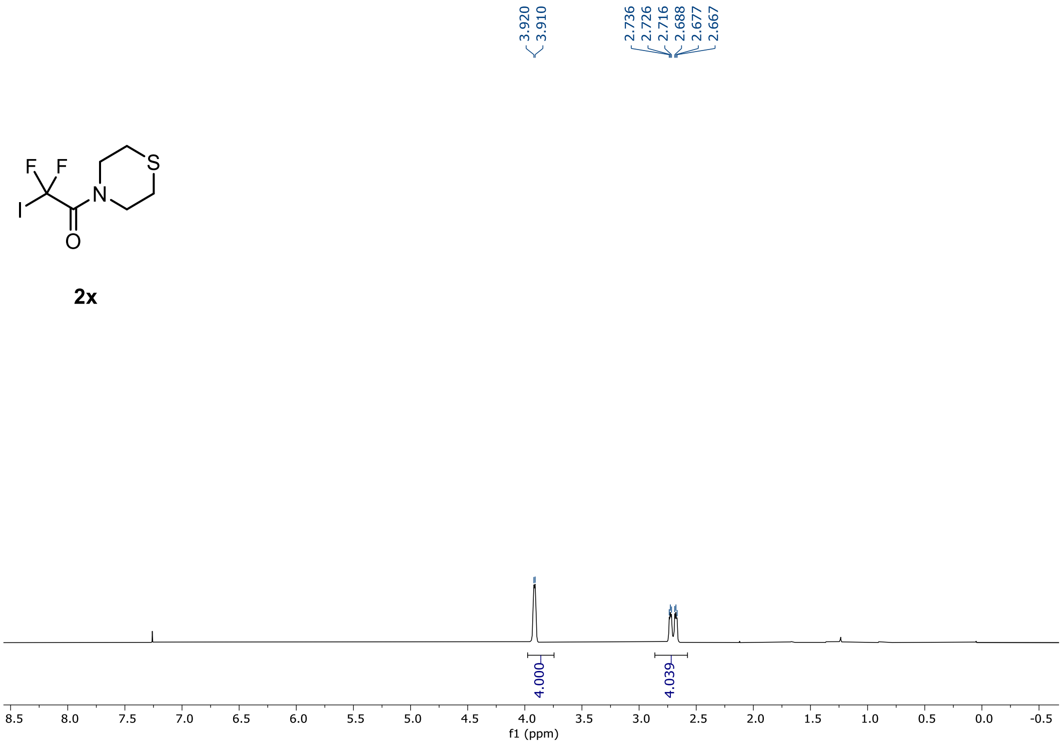

^{13}C NMR (126 MHz, CDCl3, **25 ºC) of (2x)**

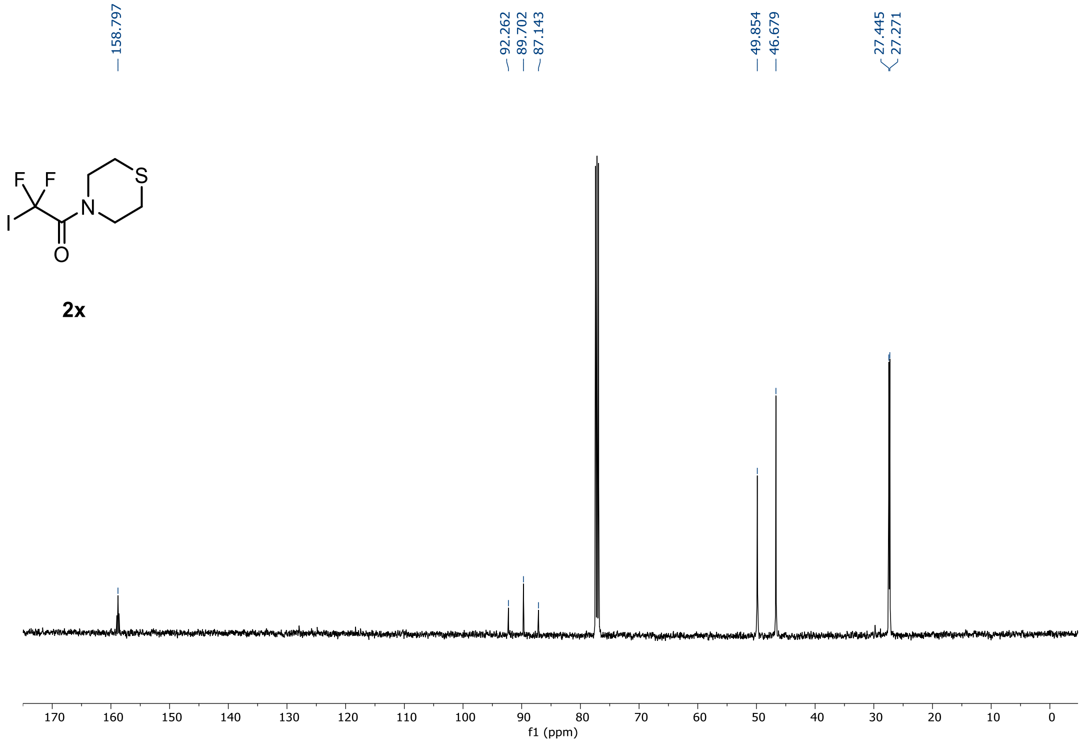

^{19}F NMR (470 MHz, $CDCl_3$**, 25 ºC) of (2x)**

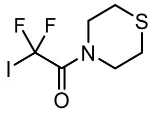

2x

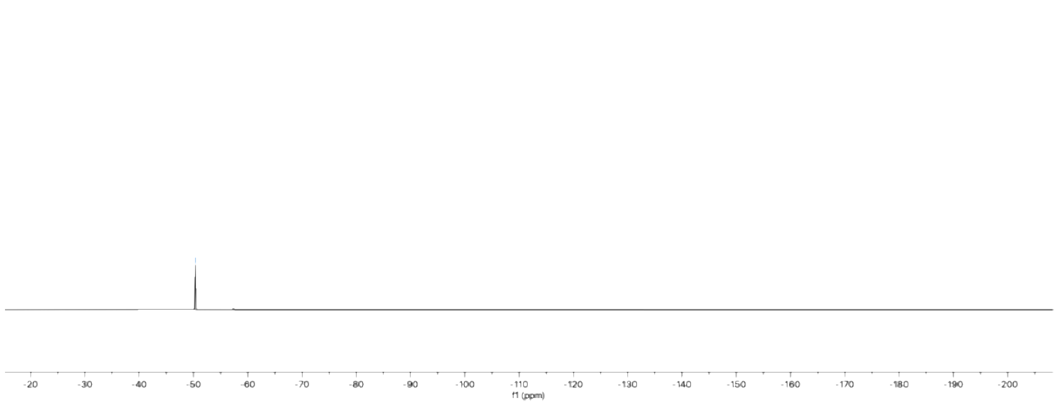

^{1}H NMR (500 MHz, $CDCl_3$, 25 ºC) of (2y)

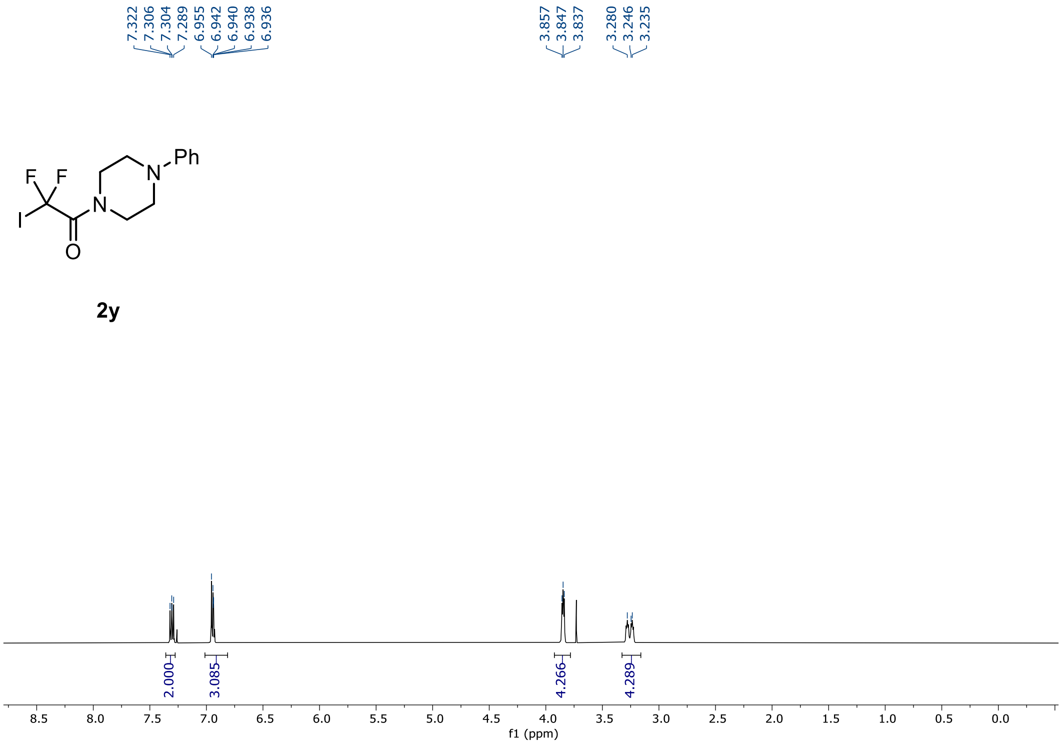

^{13}C NMR (126 MHz, $CDCl_3$, 25 ºC) of (2y)

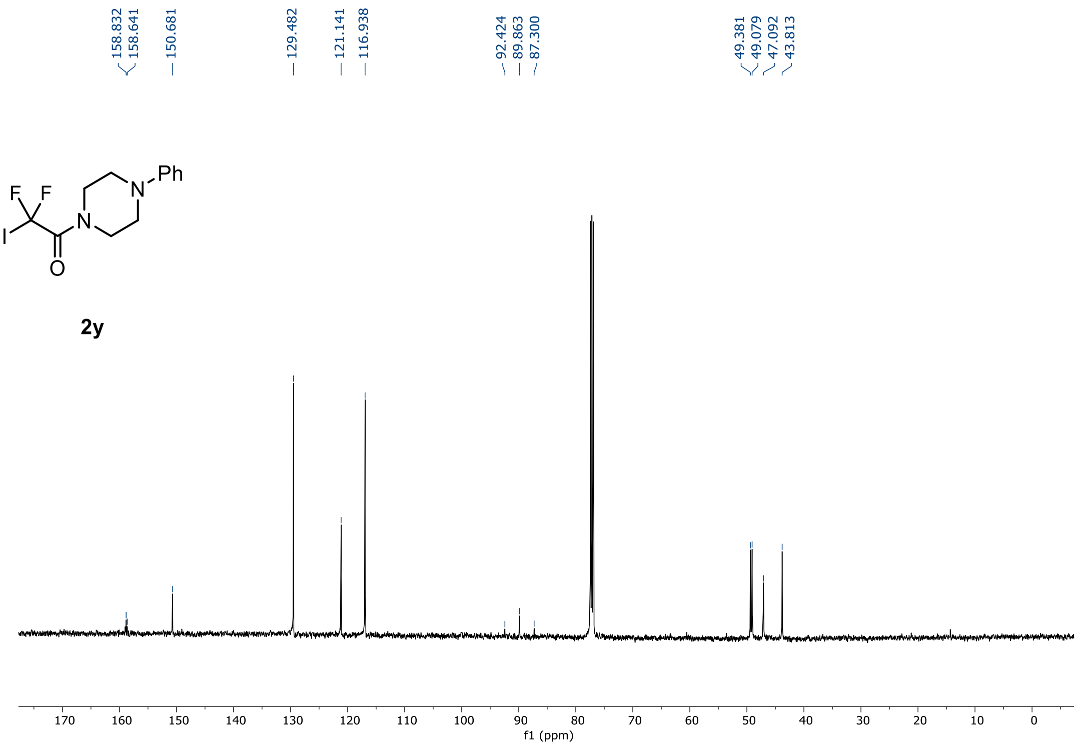

^{19}F NMR (470 MHz, CDCl$_3$**, 25 ^{o}C) of (2y)**

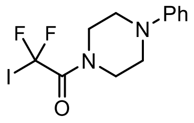

2y

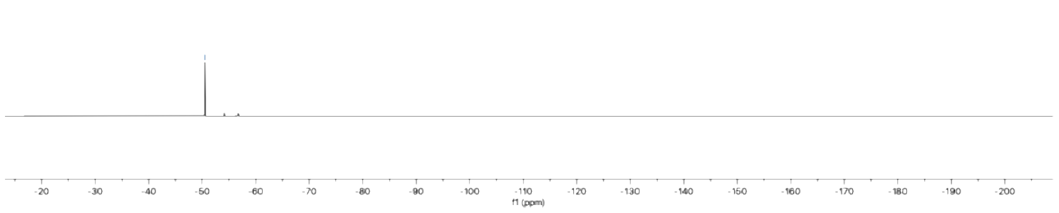

^{1}H NMR (500 MHz, CDCl$_3$, 25 ºC) of (2z)

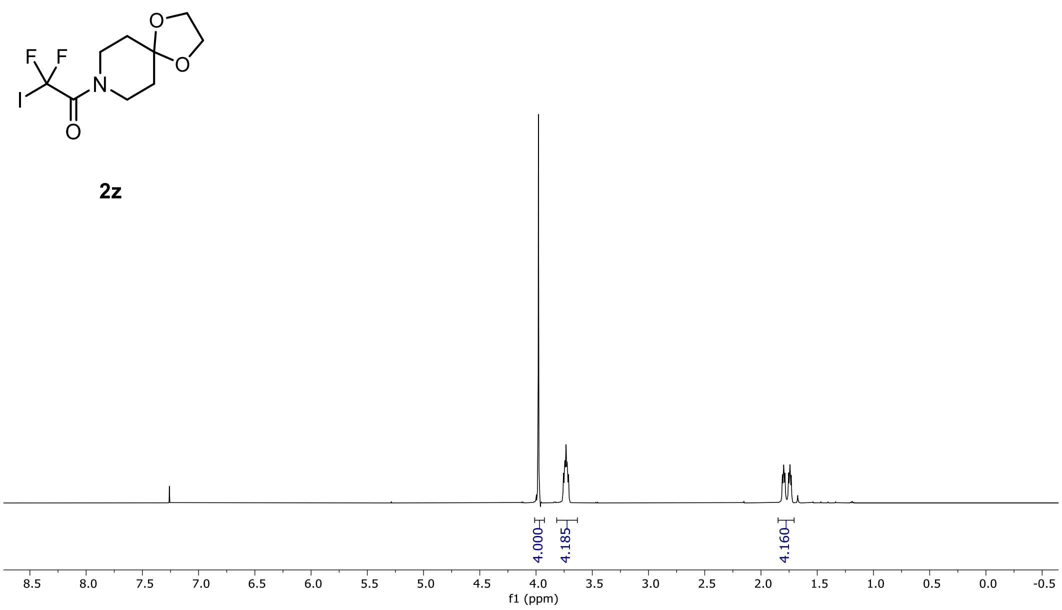

^{13}C NMR (126 MHz, CDCl$_3$, 25 ºC) of (2z)

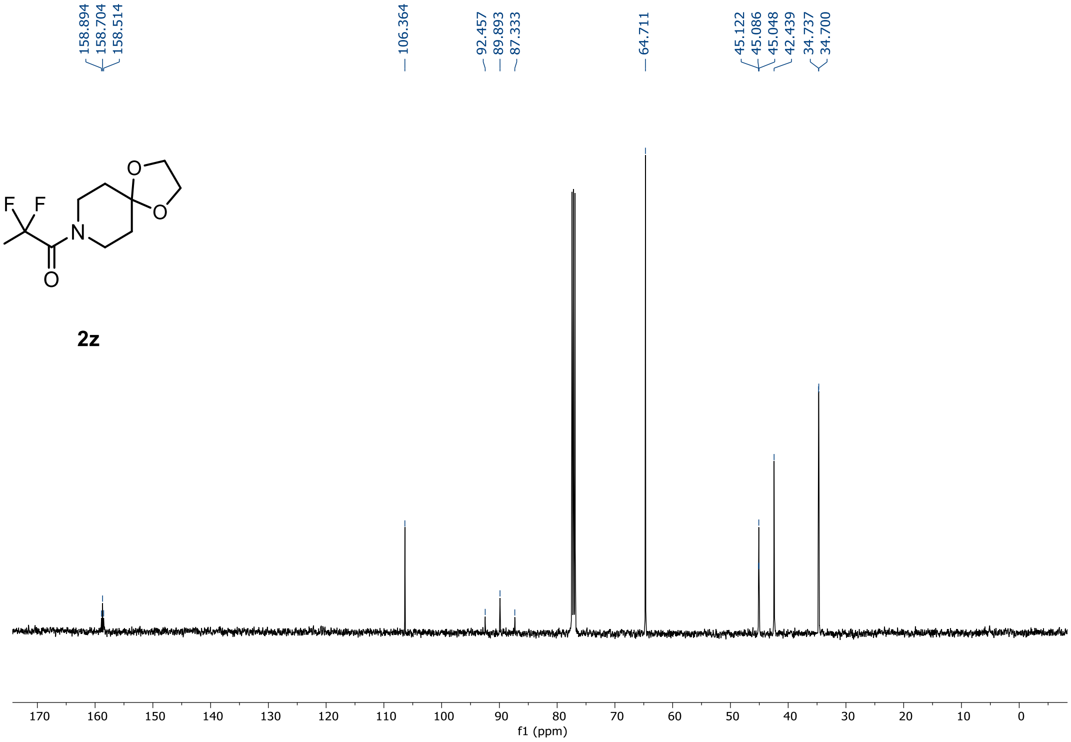

^{19}F NMR (470 MHz, CDCl$_3$, 25 ºC) of (2z)

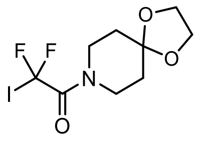

2z

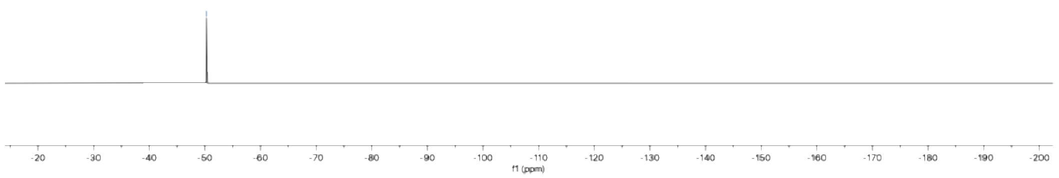

^{1}H NMR (500 MHz, CDCl$_3$**, 25 ºC) of (2aa)**

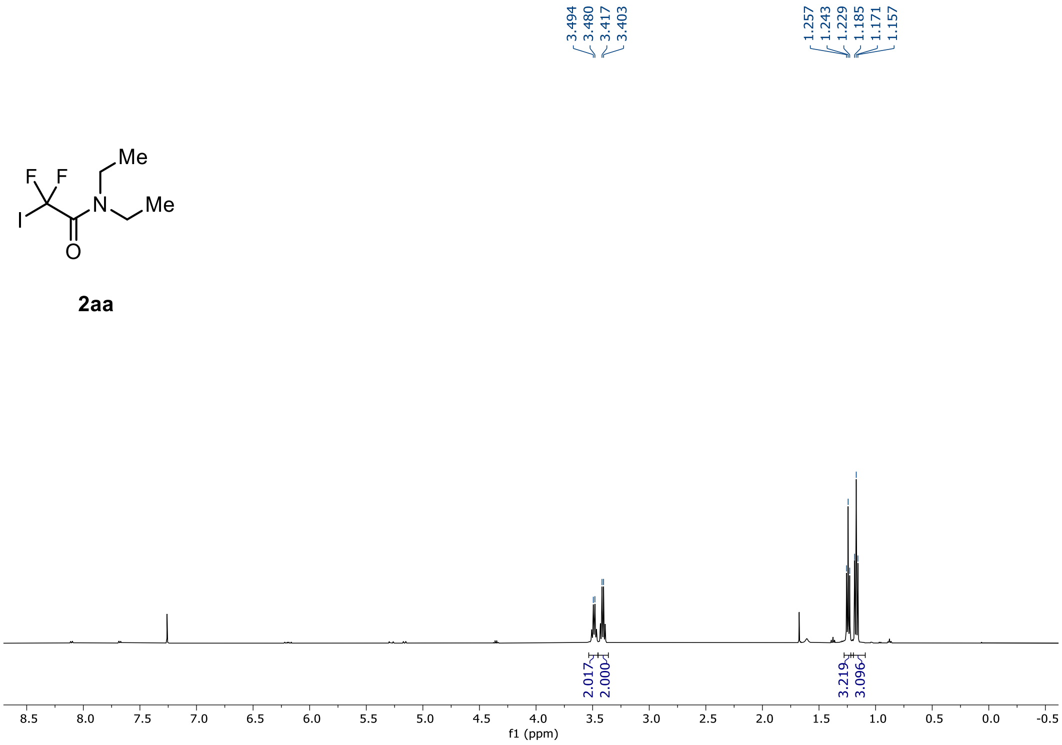

^{13}C NMR (126 MHz, CDCl$_3$**, 25 ºC) of (2aa)**

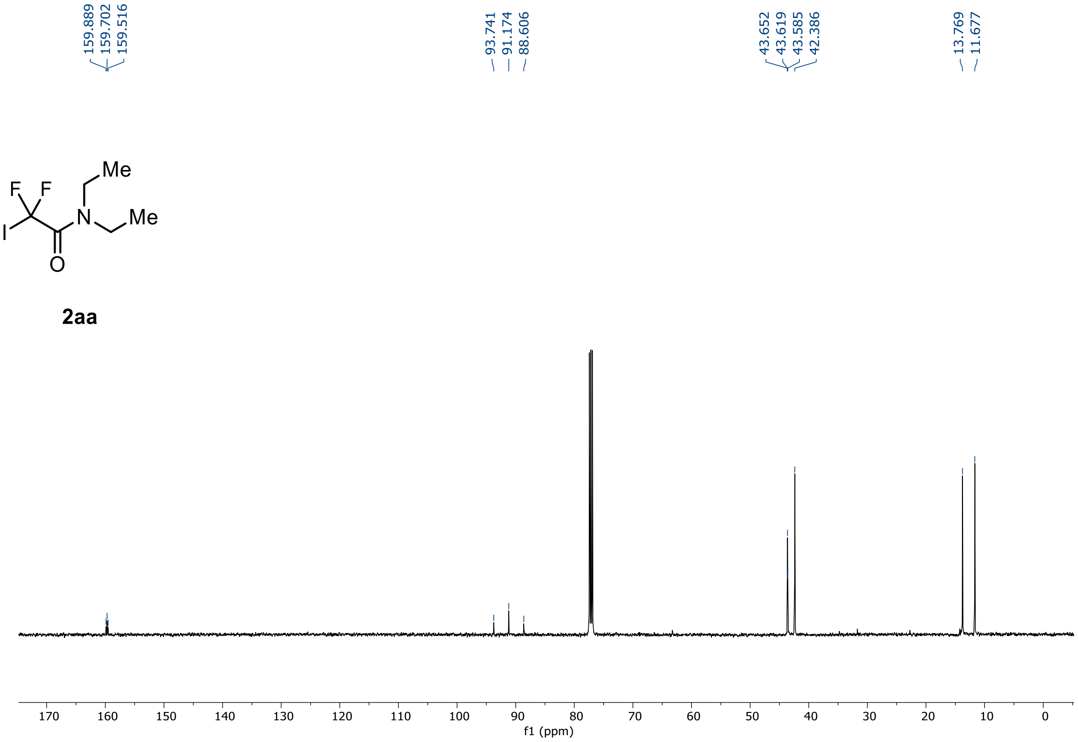

^{19}F NMR (470 MHz, CDCl$_3$**, 25 ºC) of (2aa)**

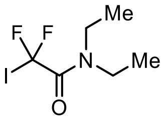

2aa

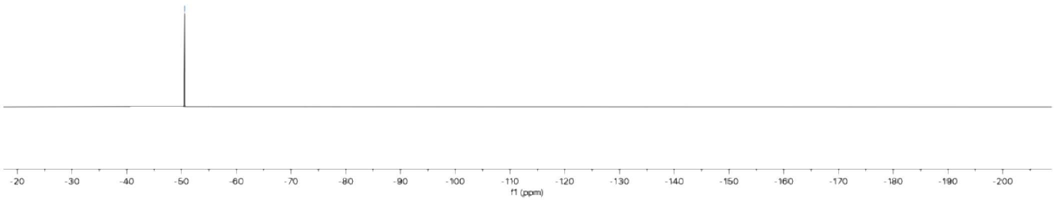

^{1}H NMR (500 MHz, $CDCl_3$, 25 ºC) of (2ab)

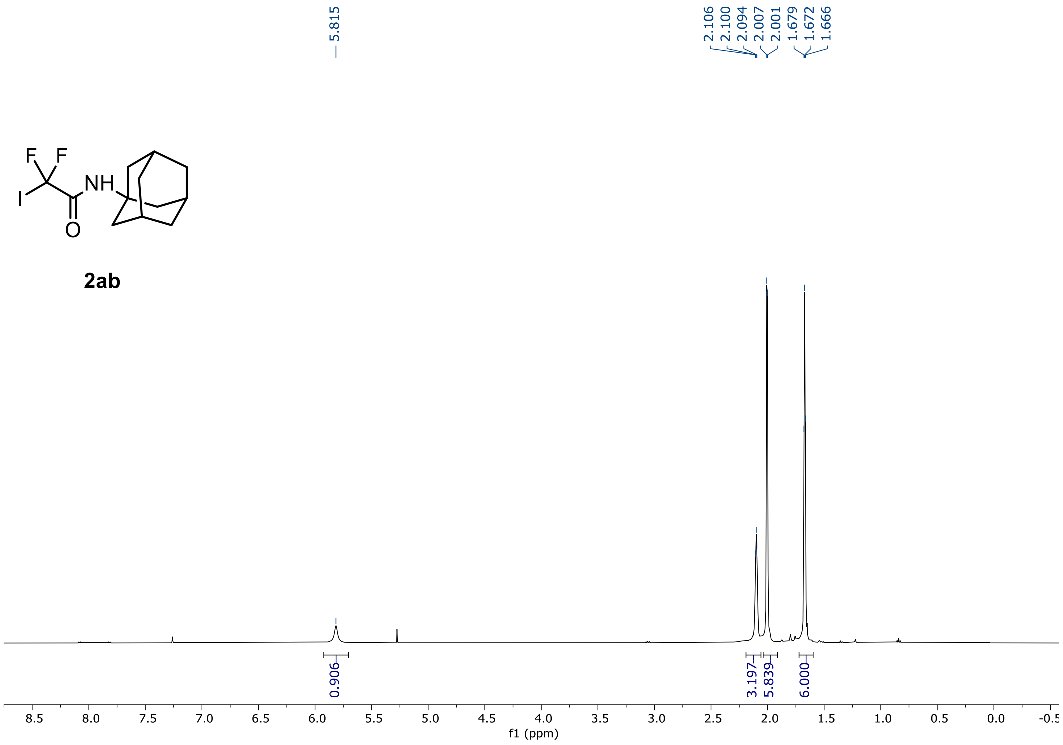

2ab

^{13}C NMR (126 MHz, $CDCl_3$, 25 ºC) of (2ab)

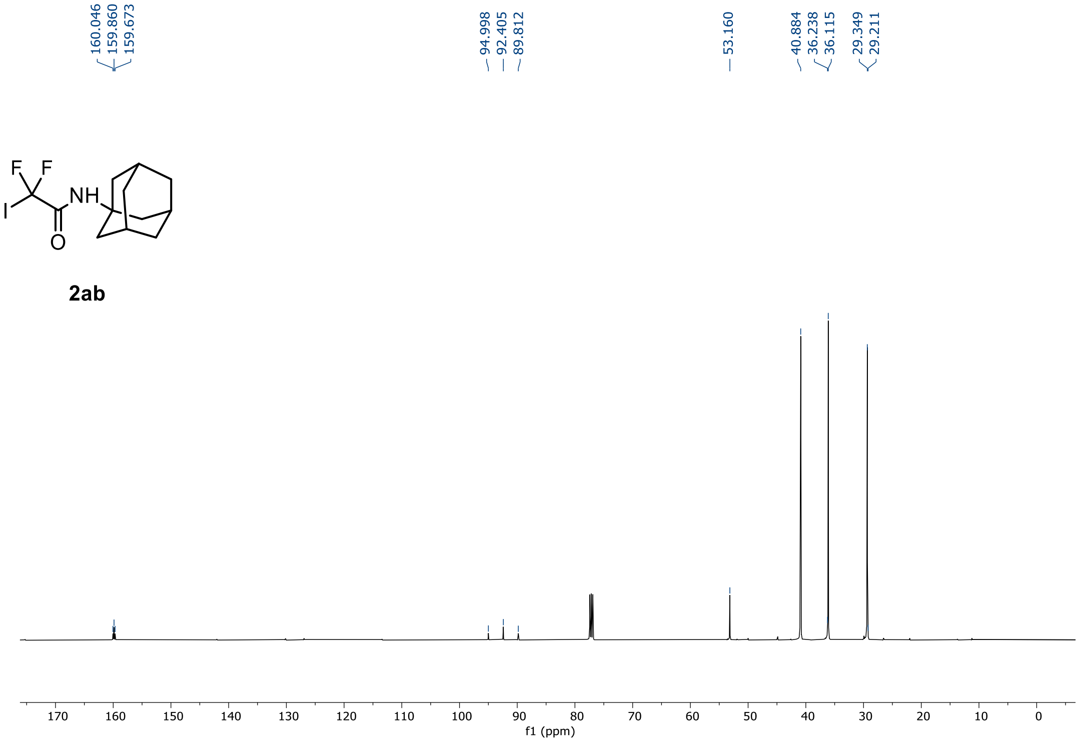

2ab

^{19}F NMR (470 MHz, $CDCl_3$**, 25 ºC) of (2ab)**

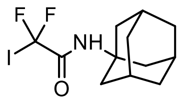

2ab

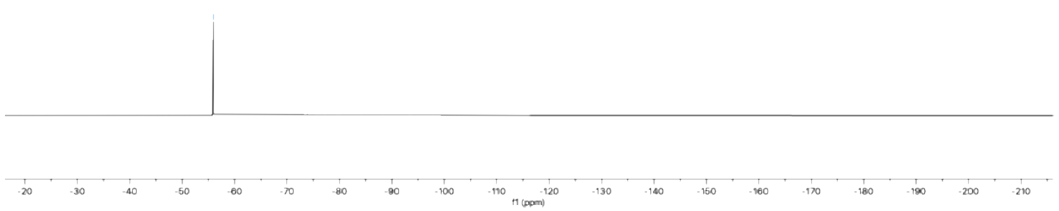

^{1}H NMR (500 MHz, $CDCl_3$, 25 ºC) of (2ac)

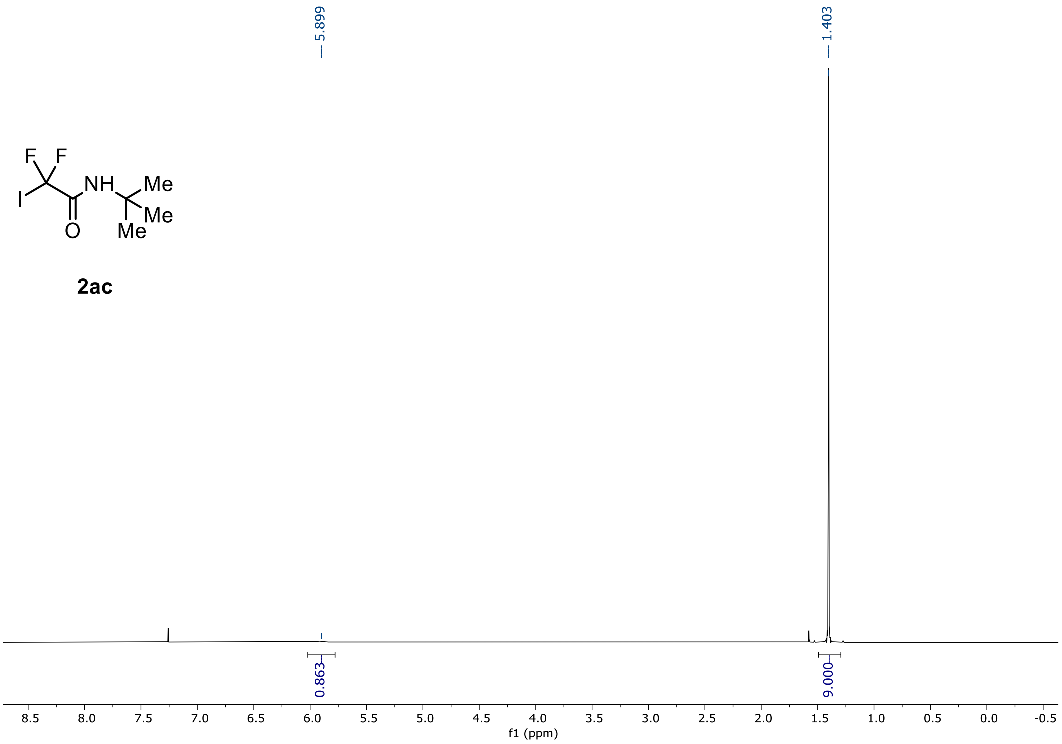

^{13}C NMR (126 MHz, $CDCl_3$, 25 ºC) of (2ac)

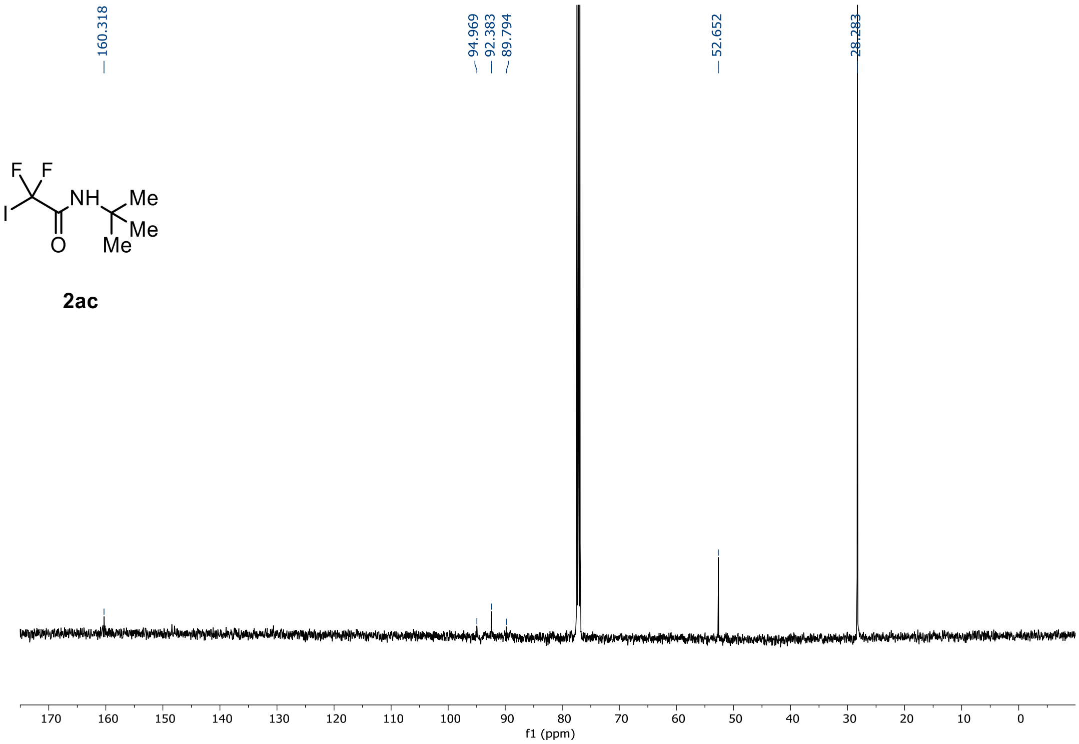

^{19}F NMR (470 MHz, $CDCl_3$**, 25 ºC) of (2ac)**

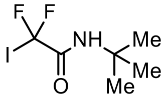

2ac

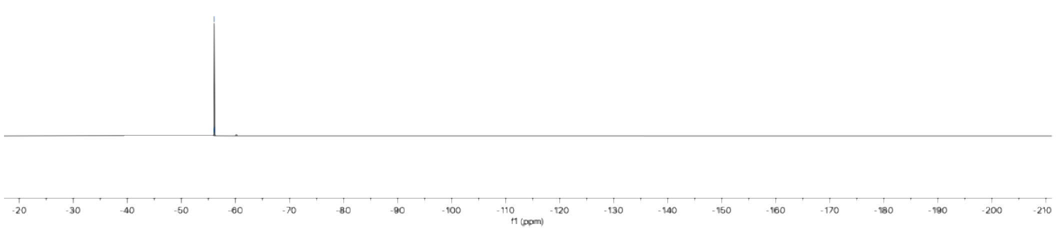

^{1}H NMR (500 MHz, $CDCl_3$, 25 ºC) of (2ad)

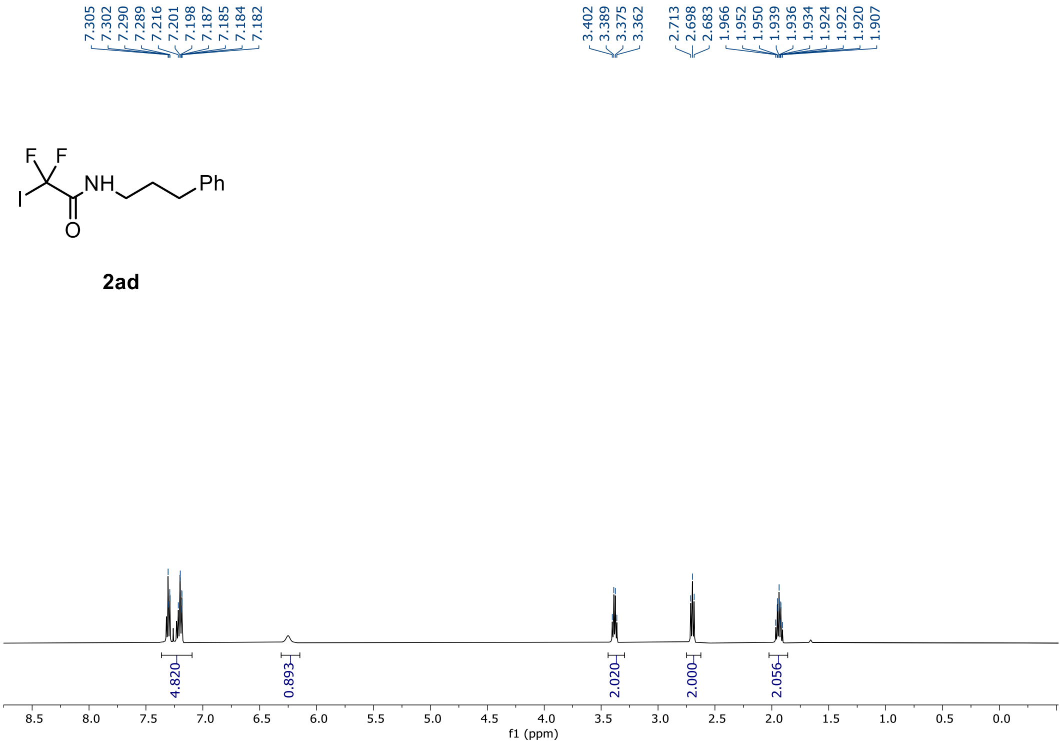

^{13}C NMR (126 MHz, $CDCl_3$, 25 ºC) of (2ad)

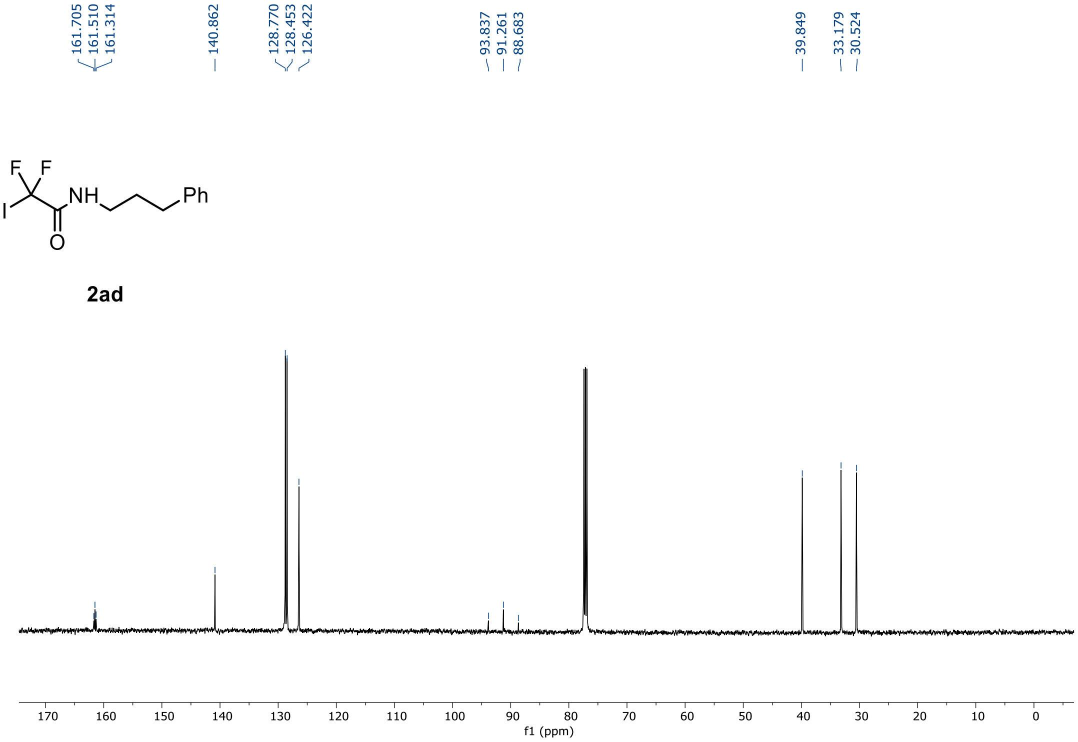

^{19}F NMR (470 MHz, CDCl$_3$**, 25 ºC) of (2ad)**

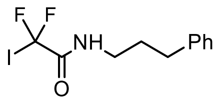

2ad

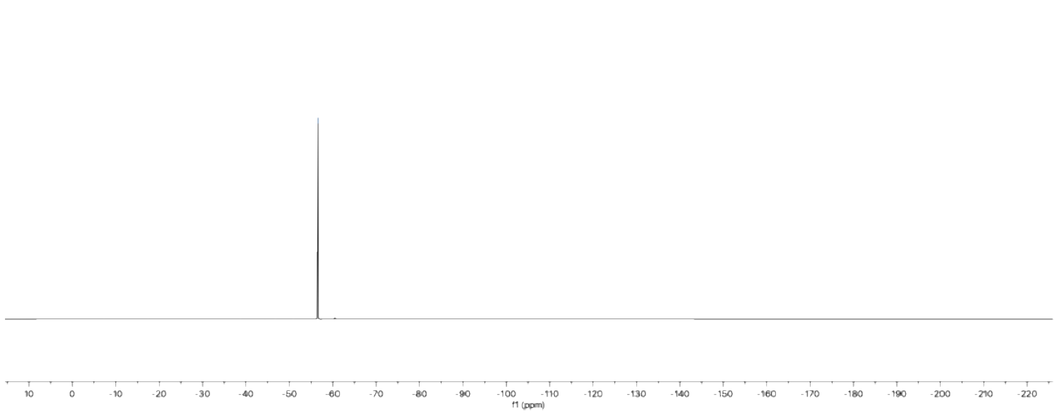

^{1}H NMR (500 MHz, $CDCl_3$, 25 ºC) of (2ae)

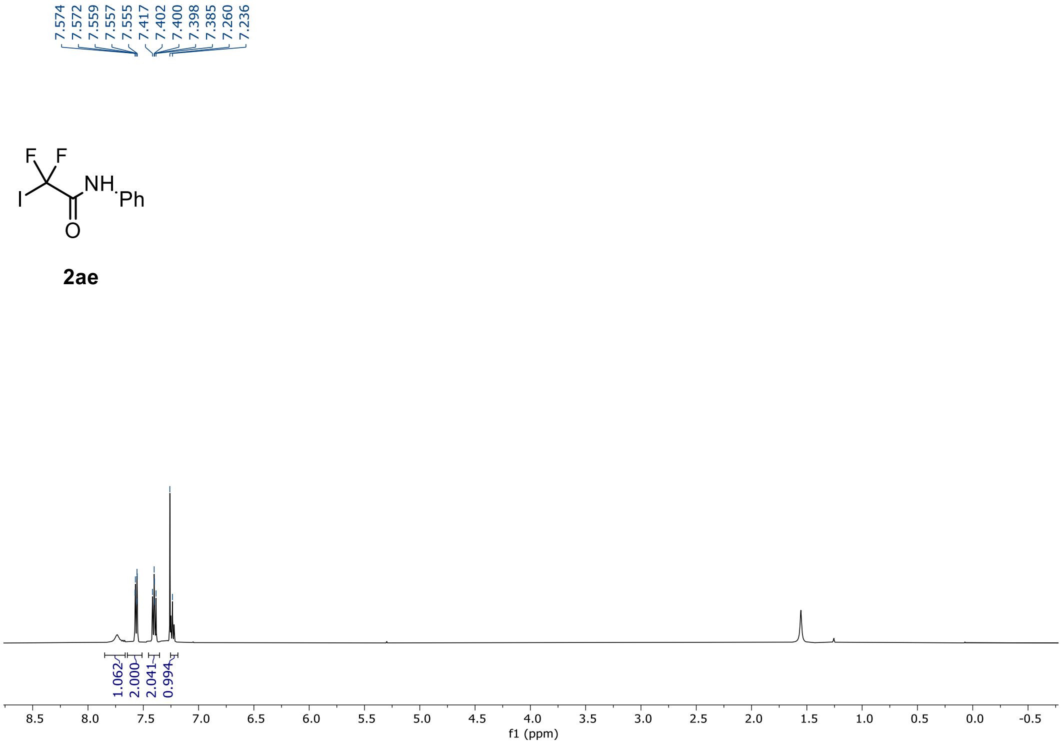

^{13}C NMR (126 MHz, $CDCl_3$, 25 ºC) of (2ae)

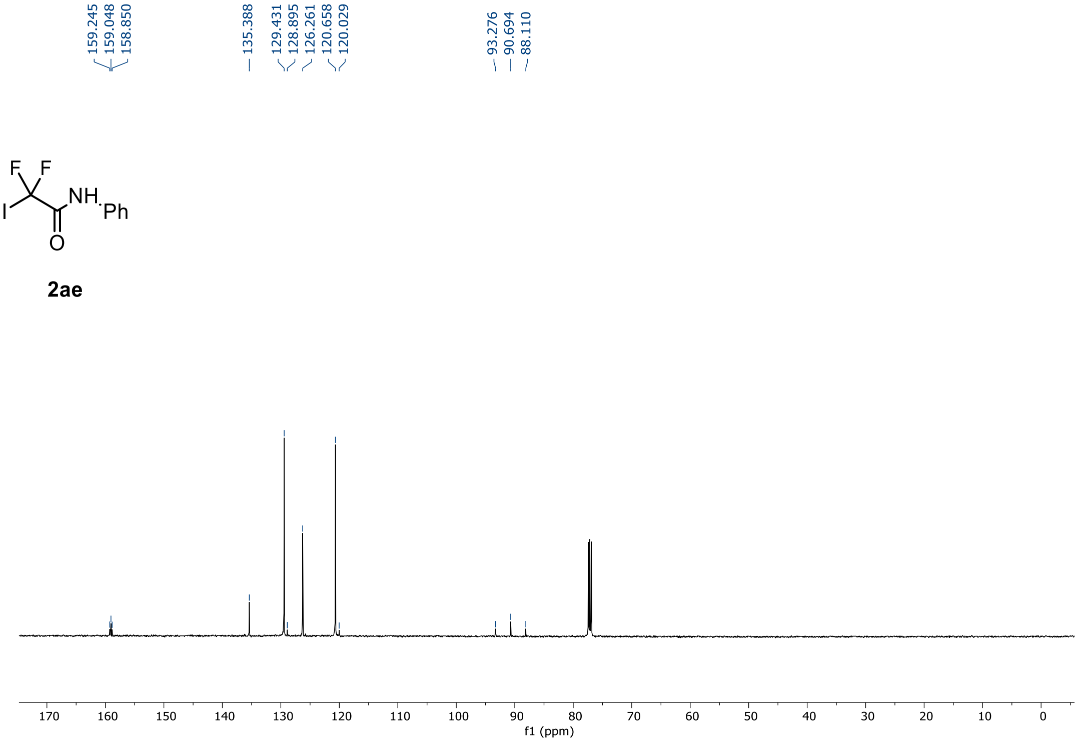

^{19}F NMR (470 MHz, CDCl$_3$**, 25 ºC) of (2ae)**

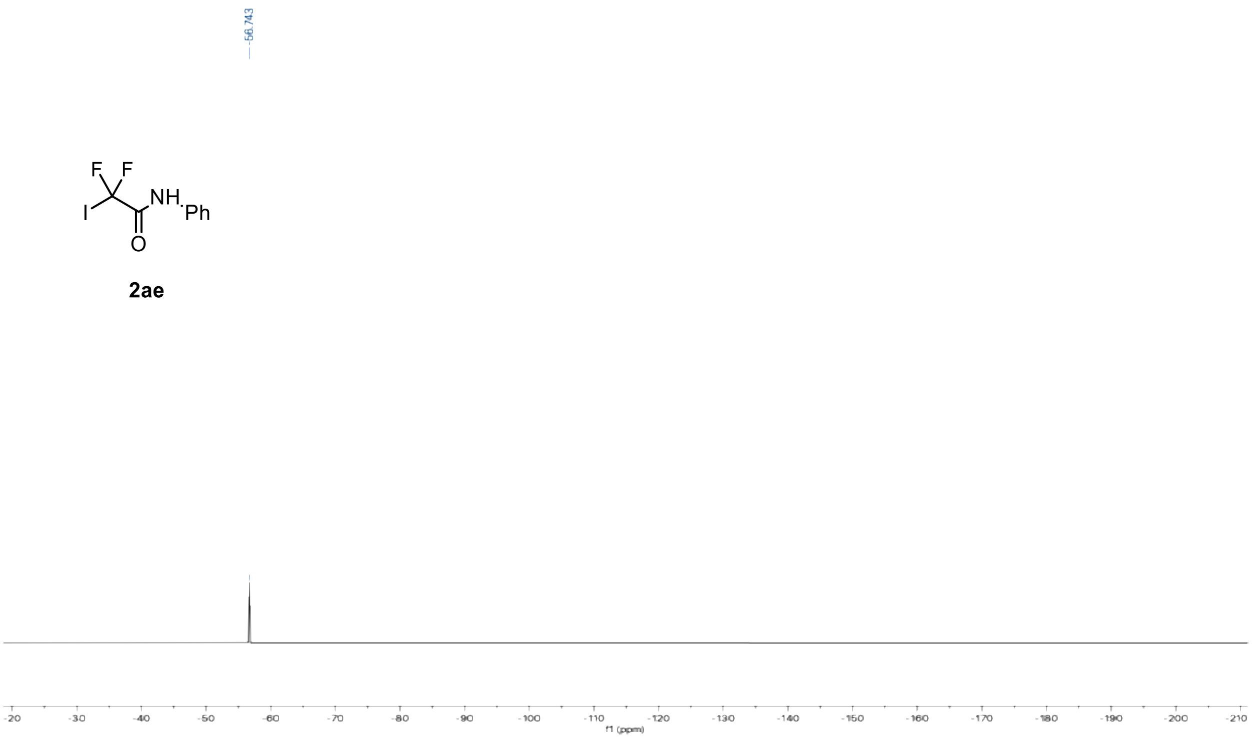

^{1}H NMR (500 MHz, $CDCl_3$, 25 ºC) of (2aj)

2aj

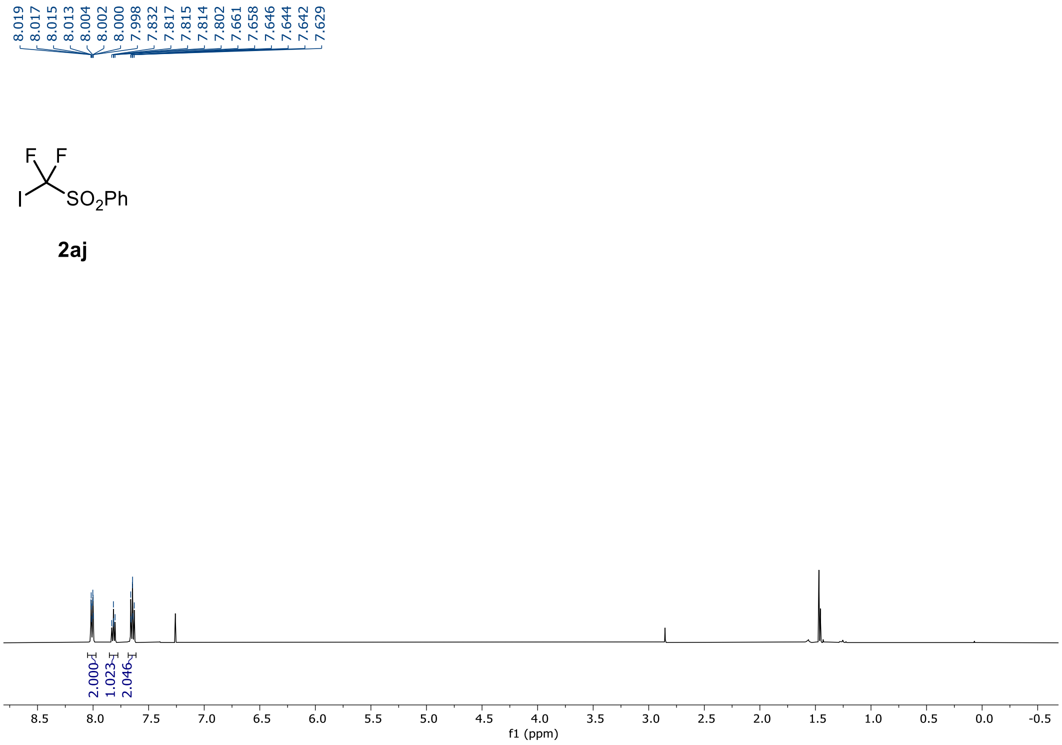

^{13}C NMR (126 MHz, $CDCl_3$, 25 ºC) of (2aj)

2aj

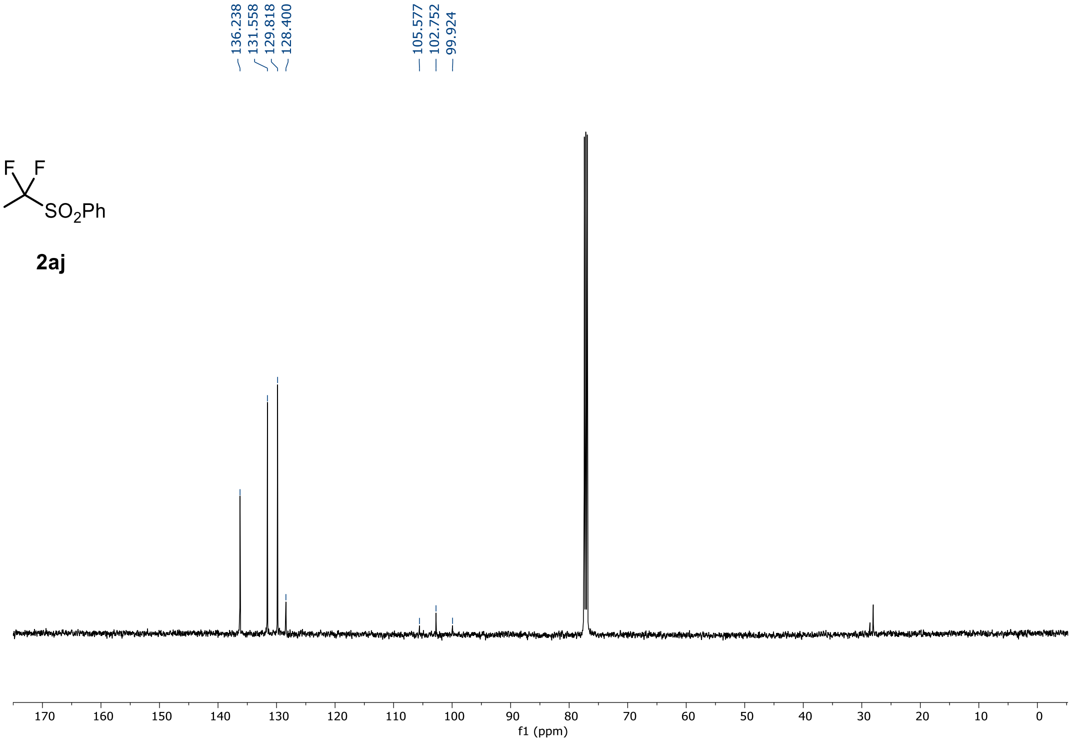

^{19}F NMR (470 MHz, $CDCl_3$**, 25 ºC) of (2aj)**

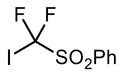

2aj

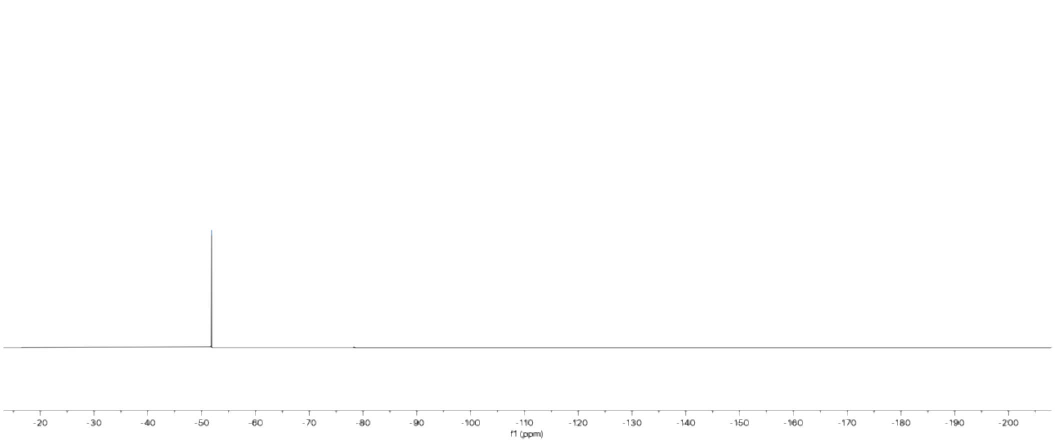

^{1}H NMR (500 MHz, $CDCl_3$, 25 ºC) of (2aaa)

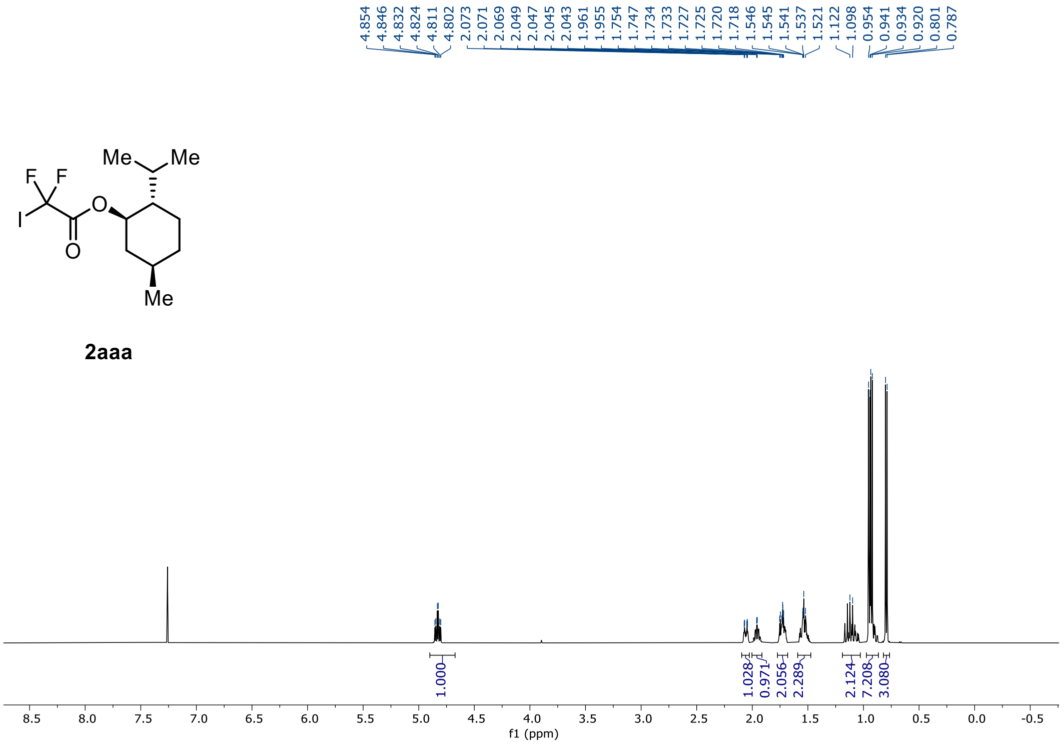

^{13}C NMR (126 MHz, $CDCl_3$, 25 ºC) of (2aaa)

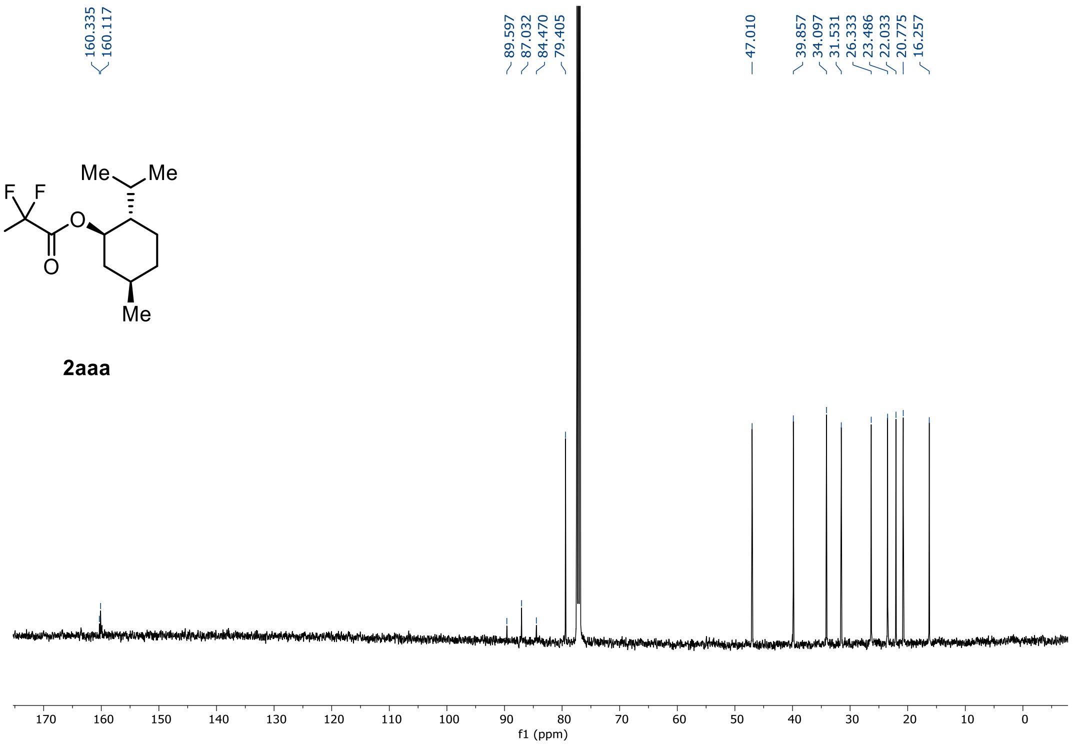

^{19}F NMR (470 MHz, $CDCl_3$**, 25 ºC) of (2aaa)**

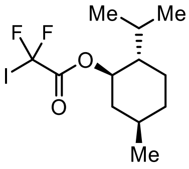

2aaa

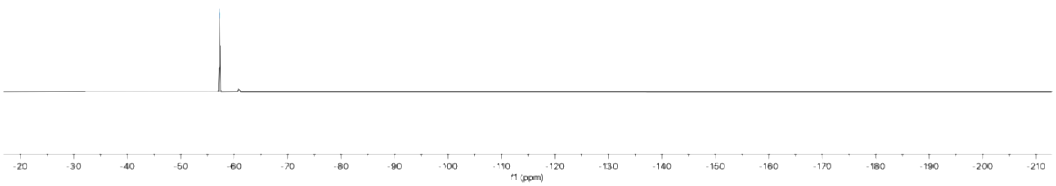

^{1}H NMR (500 MHz, $CDCl_3$, 25 ºC) of (2aab)

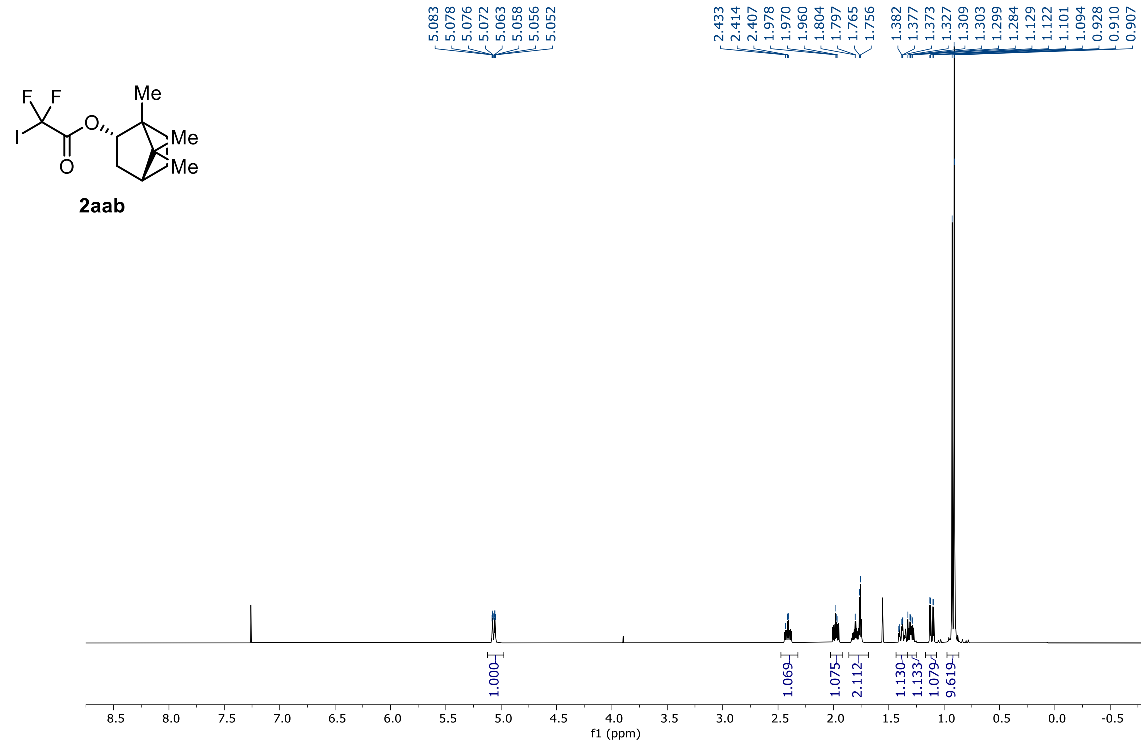

^{13}C NMR (126 MHz, $CDCl_3$, 25 ºC) of (2aab)

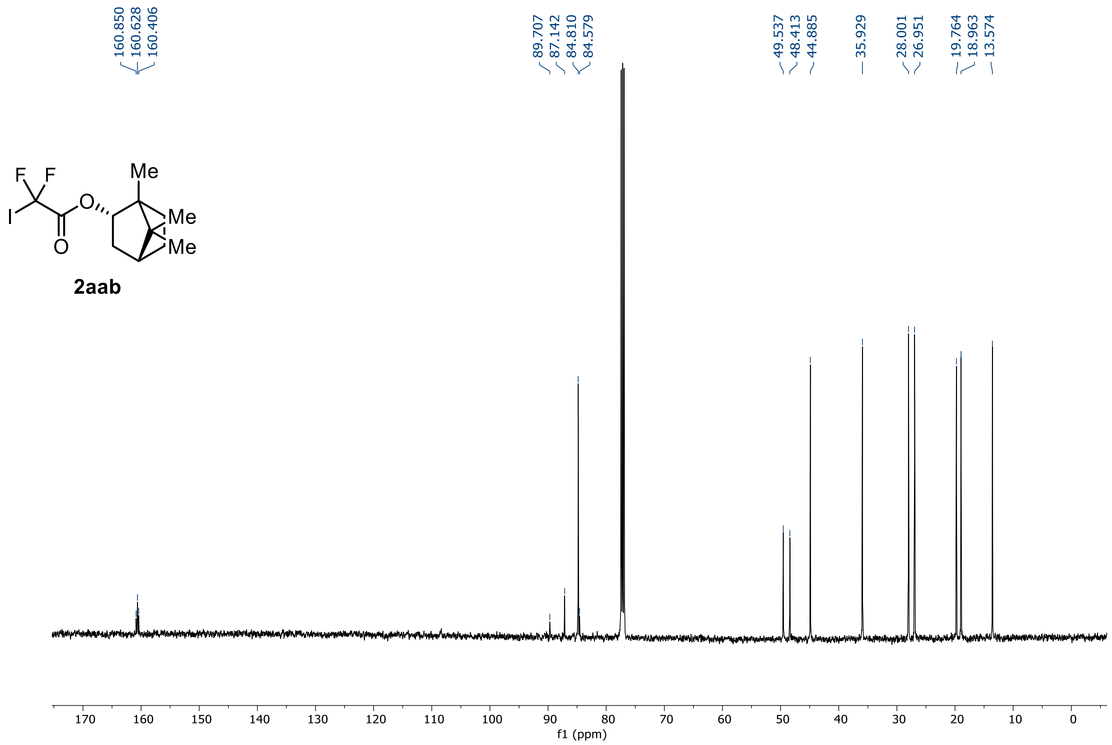

^{19}F NMR (470 MHz, $CDCl_3$**, 25 ºC) of (2aab)**

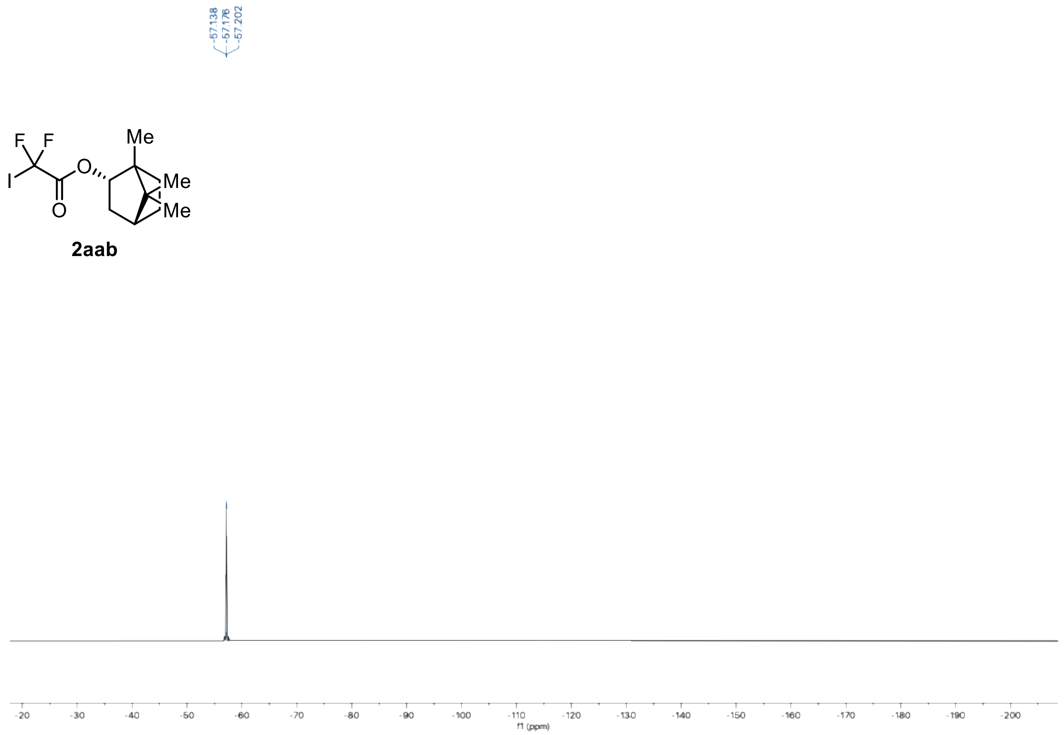

^{1}H NMR (500 MHz, CDCl$_3$, **25 ºC) of (2aac)**

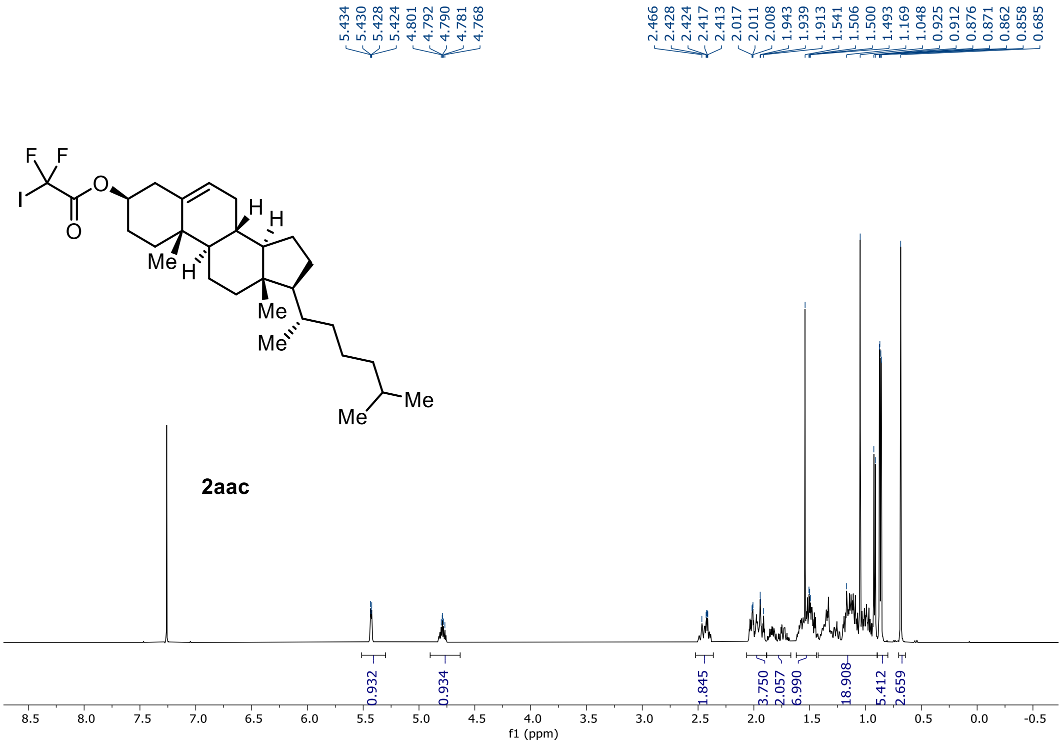

^{13}C NMR (126 MHz, CDCl$_3$, **25 ºC) of (2aac)**

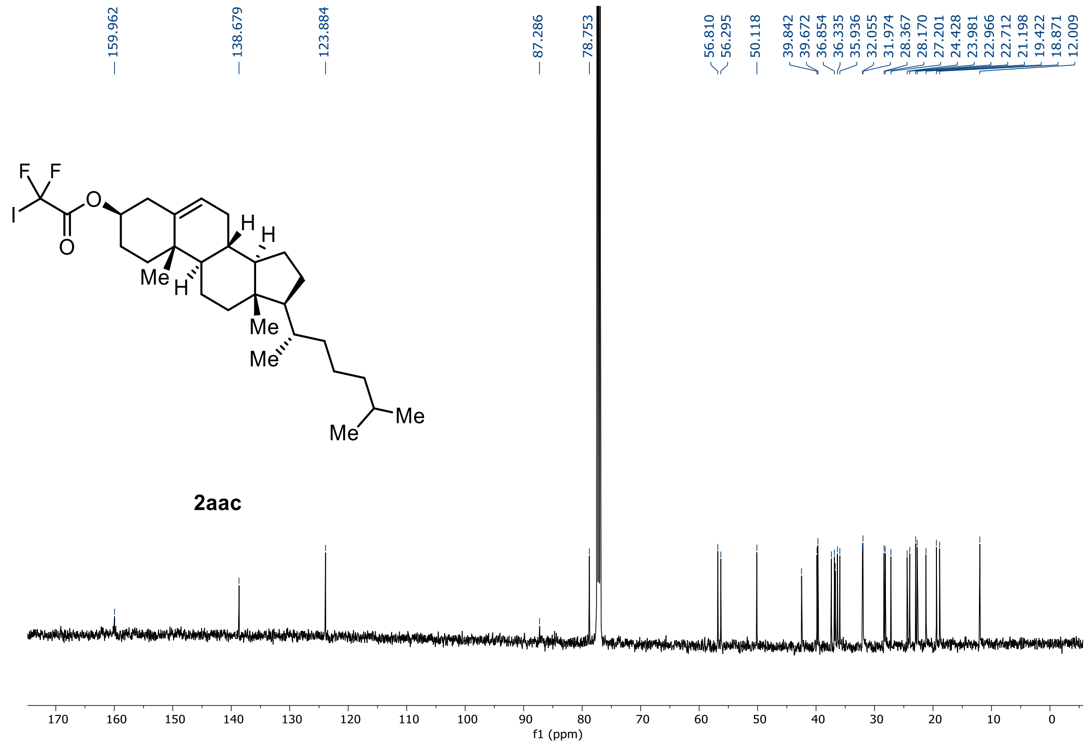

^{19}F NMR (470 MHz, $CDCl_3$**, 25 ºC) of (2aac)**

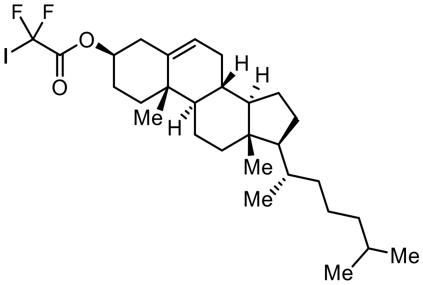

2aac

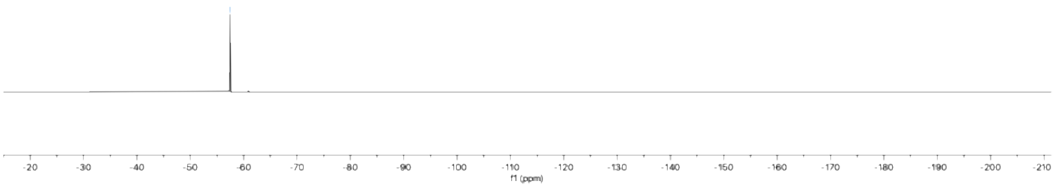

^{1}H NMR (500 MHz, CDCl$_3$, **25 ºC) of (2aad)**

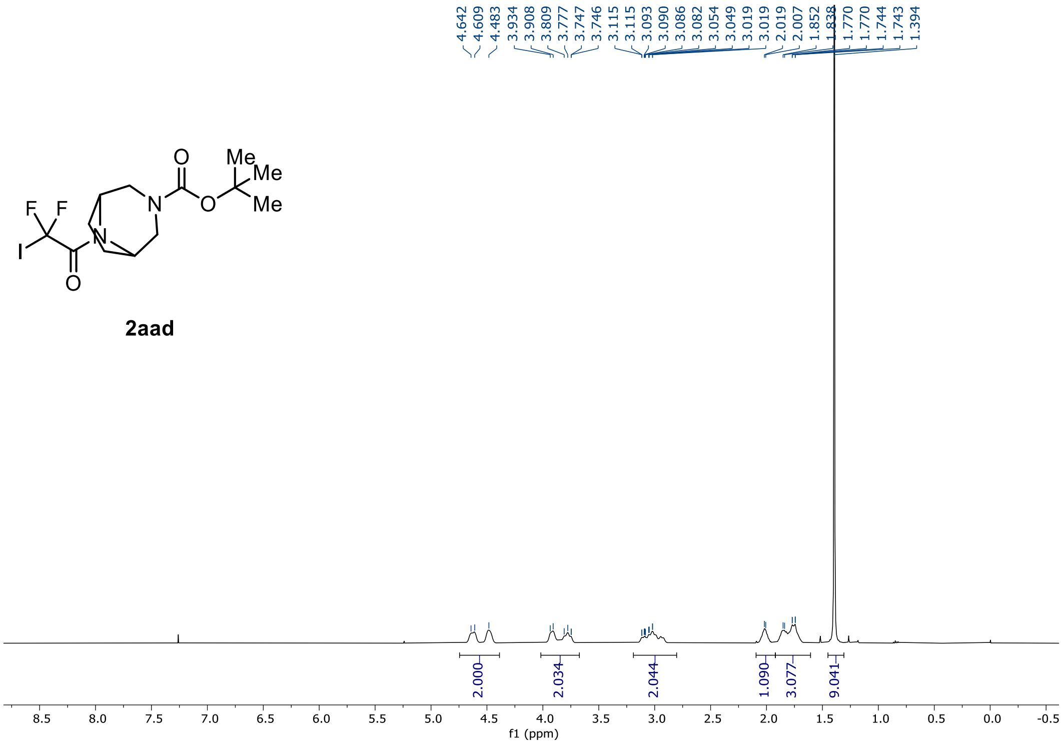

^{13}C NMR (126 MHz, CDCl$_3$, **25 ºC) of (2aad)**

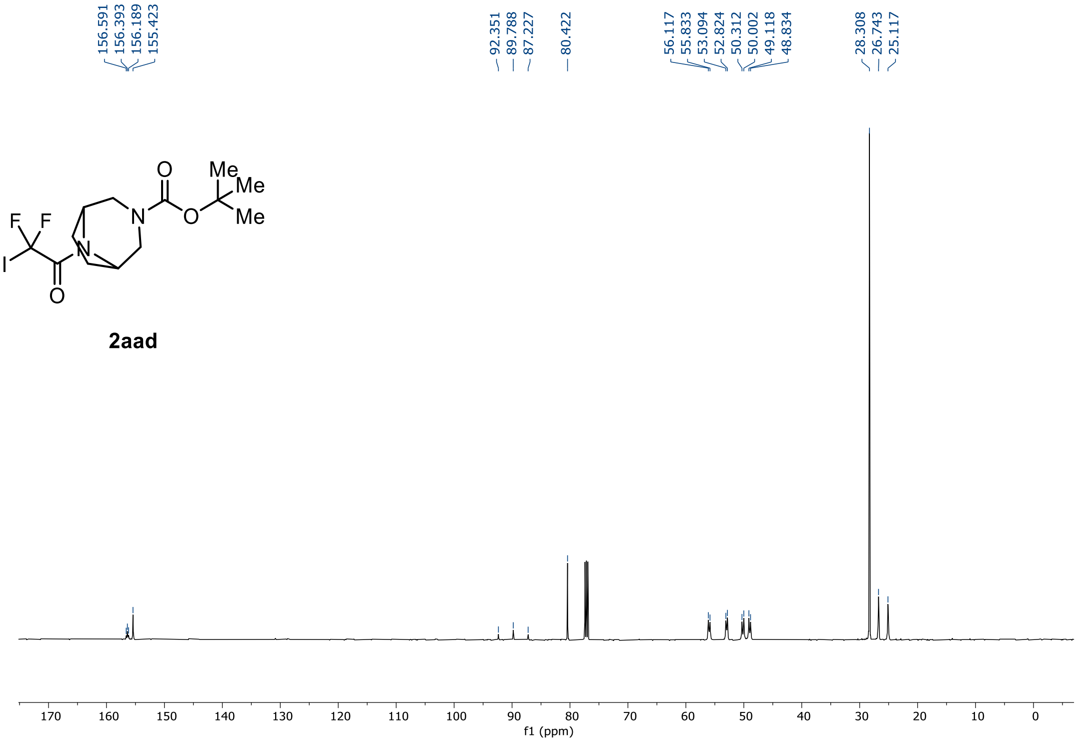

^{19}F NMR (470 MHz, CDCl$_3$, 25 ºC) of (2aad)

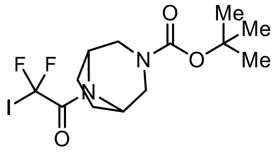

2aad

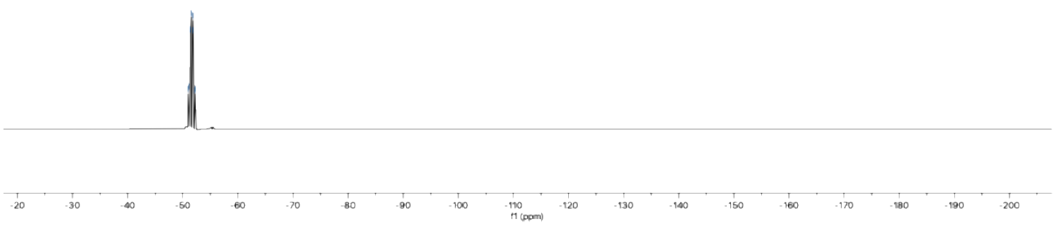

^{1}H NMR (500 MHz, $CDCl_3$**, 25 ºC) of (5)**

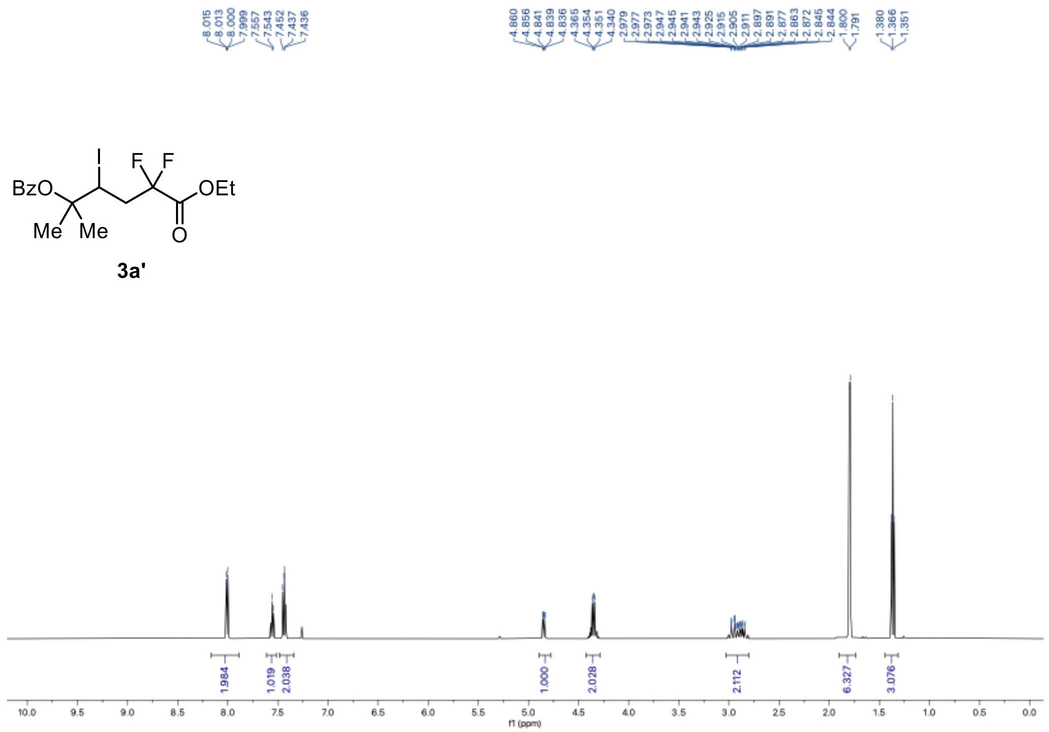

^{13}C NMR (126 MHz, $CDCl_3$**, 25 ºC) of (5)**

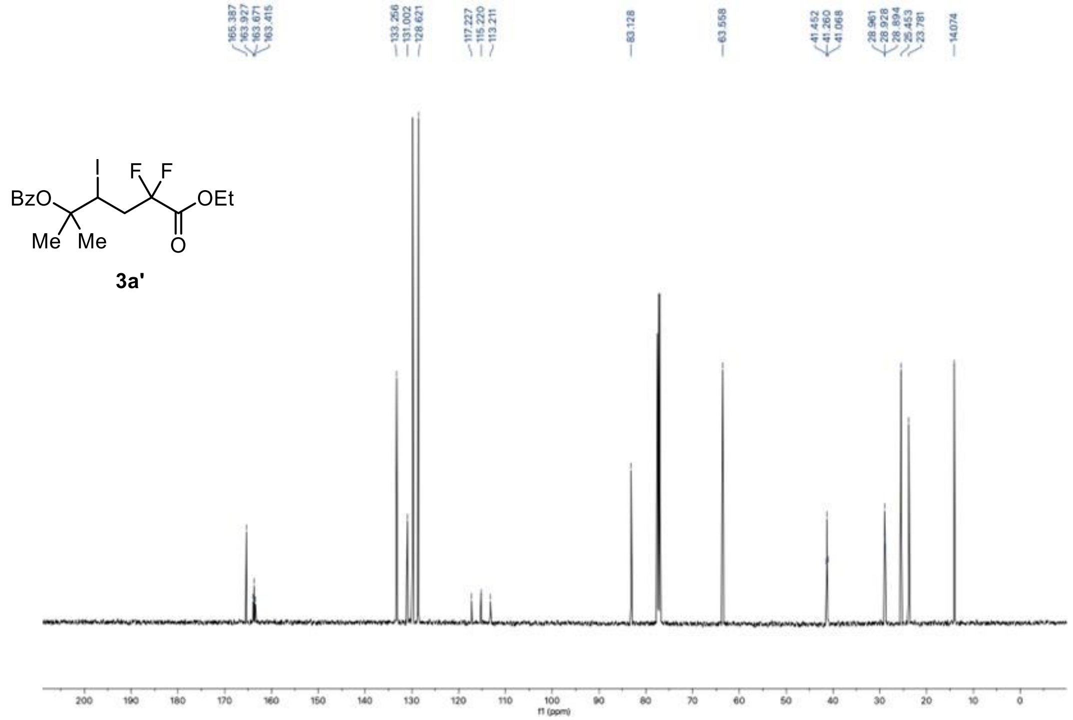

^{19}F NMR (470 MHz, $CDCl_3$**, 25 ºC) of (5)**

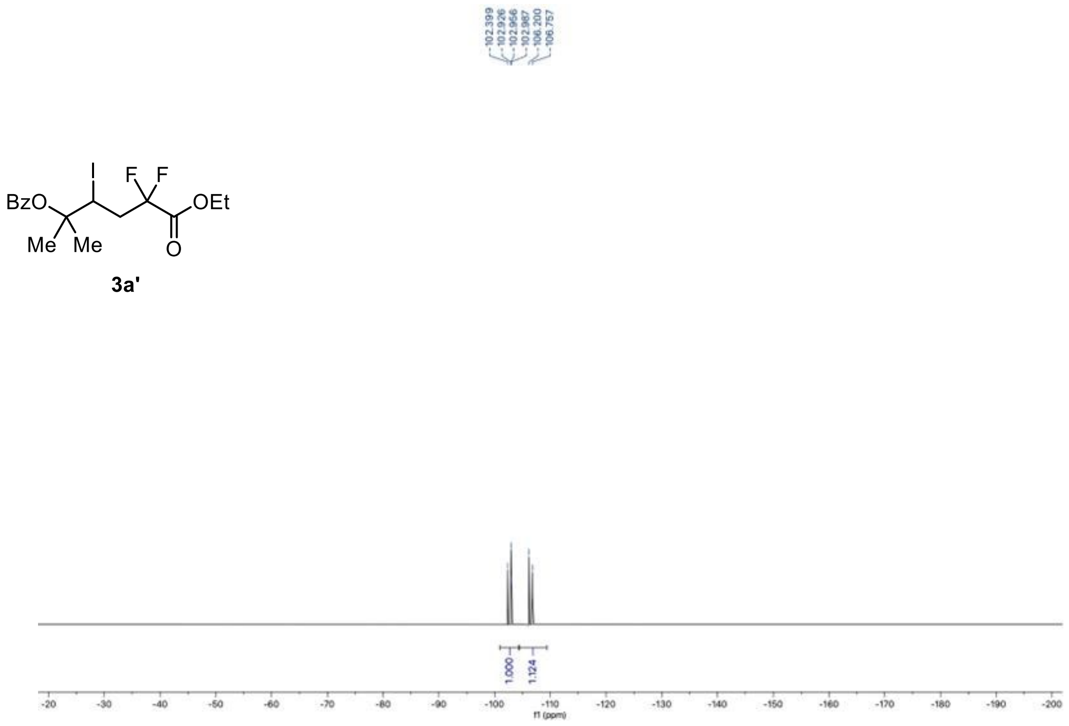

^{1}H NMR (500 MHz, CDCl$_3$, 25 ºC) of (3a)

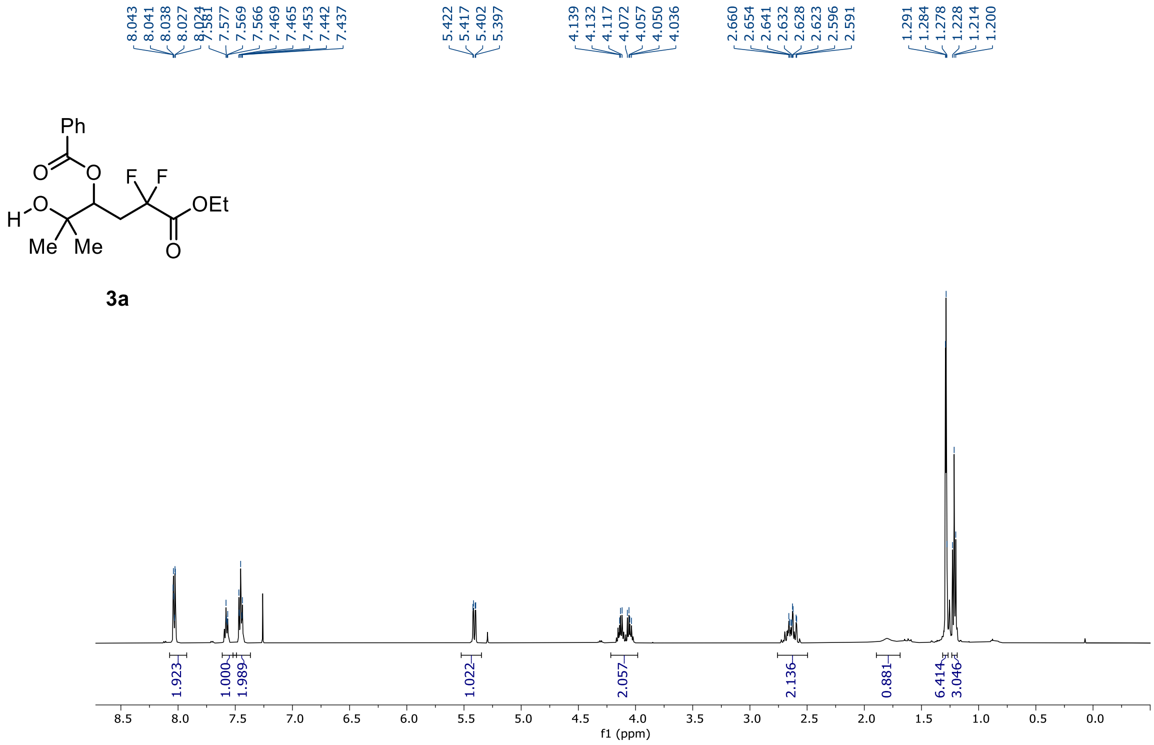

^{13}C NMR (126 MHz, CDCl$_3$, 25 ºC) of (3a)

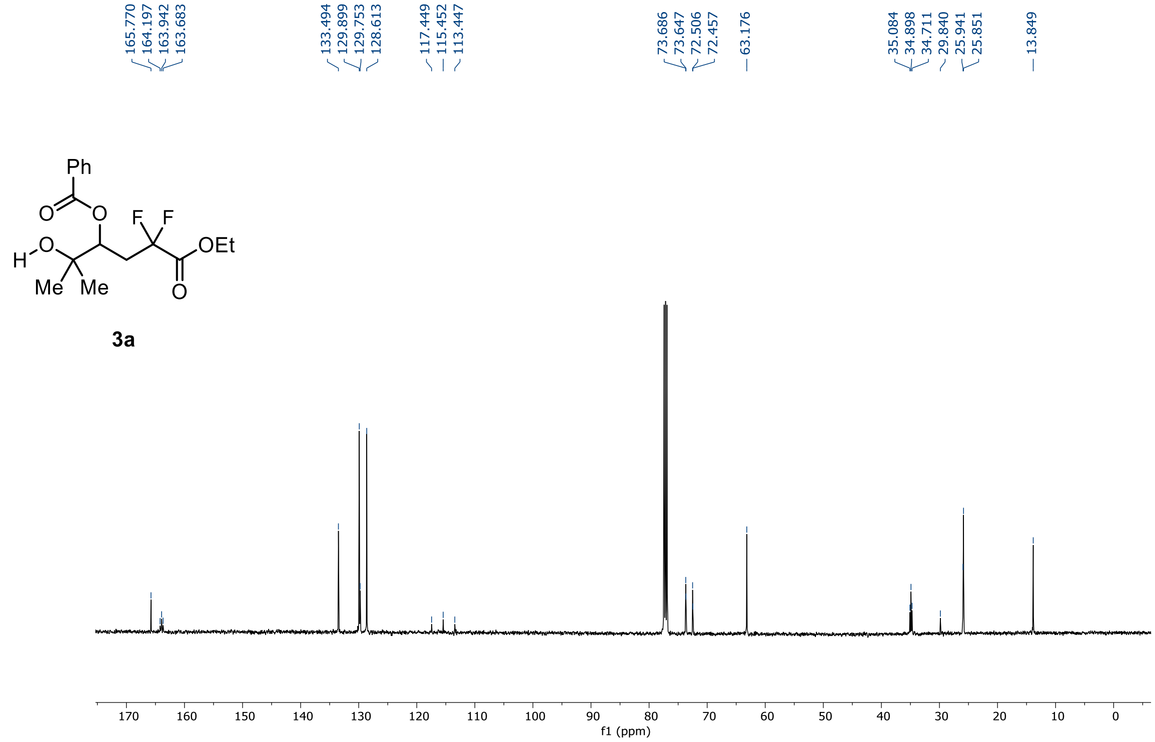

^{19}F NMR (470 MHz, CDCl$_3$, 25 ºC) of (3a)

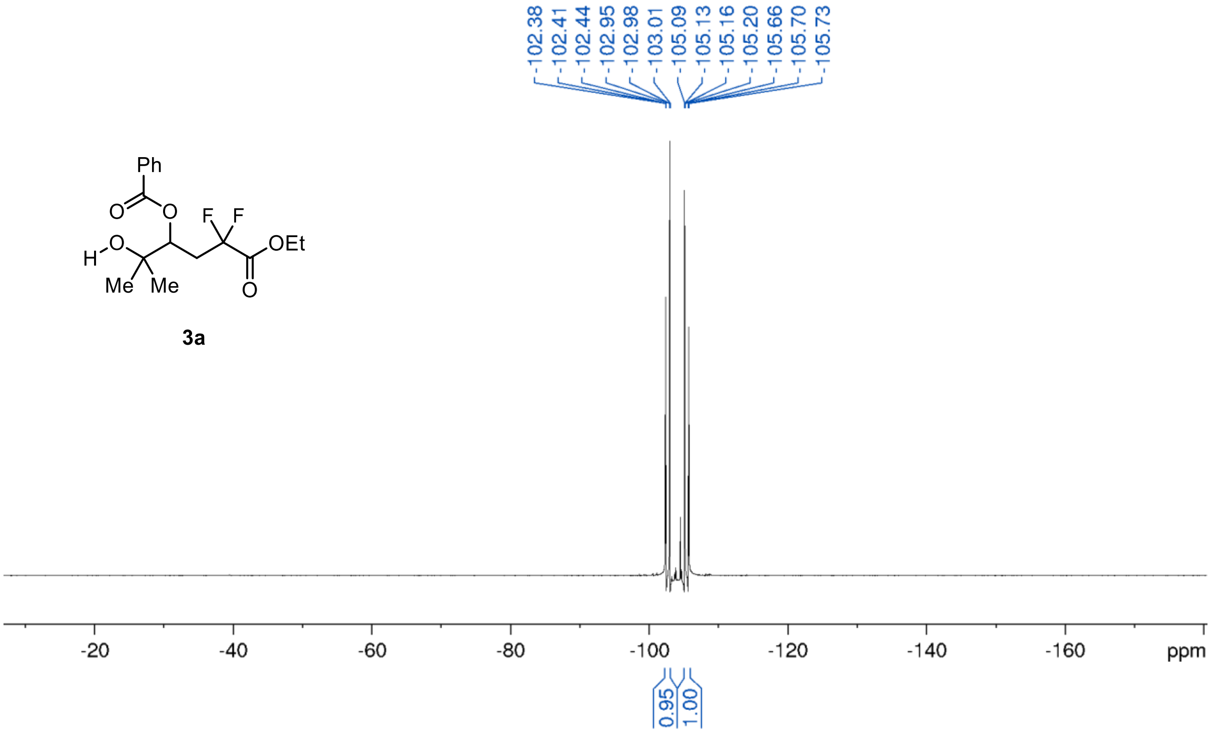
-102.38
-102.41
-102.44
-102.95
-102.98
-103.01
-105.09
-105.13
-105.16
-105.20
-105.66
-105.70
-105.73
Ph
O
O
F
F
H
O
OEt
Me
Me
O
-20
-40
-60
-80
-100
-120
-140
-160
ppm
0.95
1.00

3a

^{1}H NMR (500 MHz, $CDCl_3$, 25 ºC) of (3b)

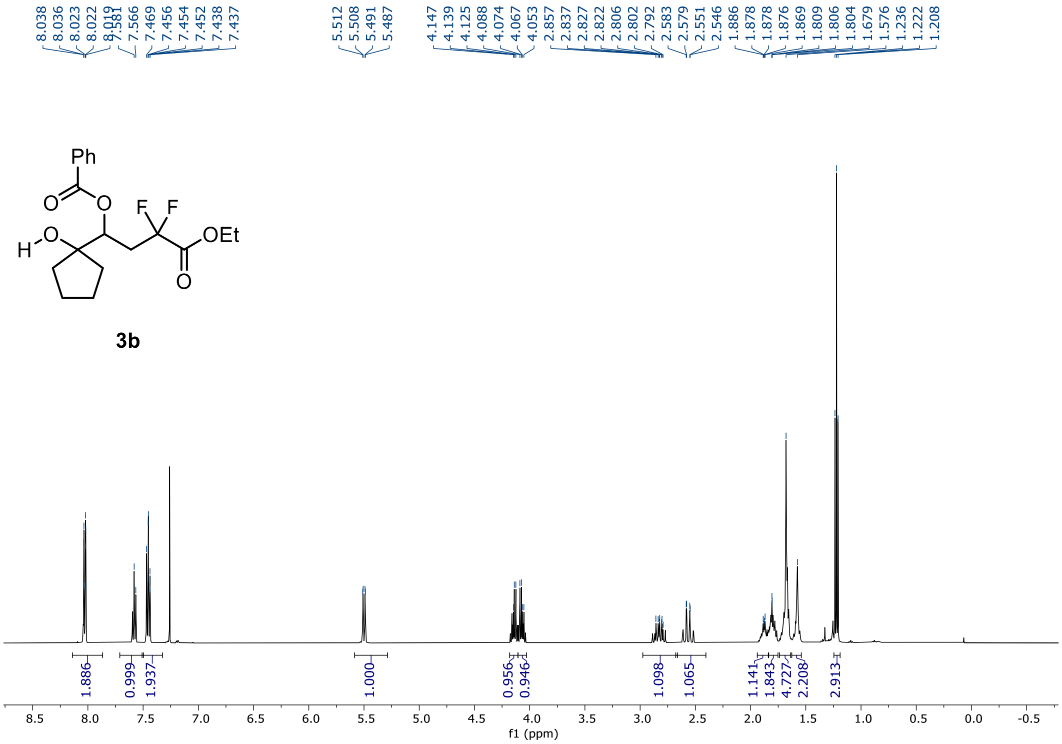

^{13}C NMR (126 MHz, $CDCl_3$, 25 ºC) of (3b)

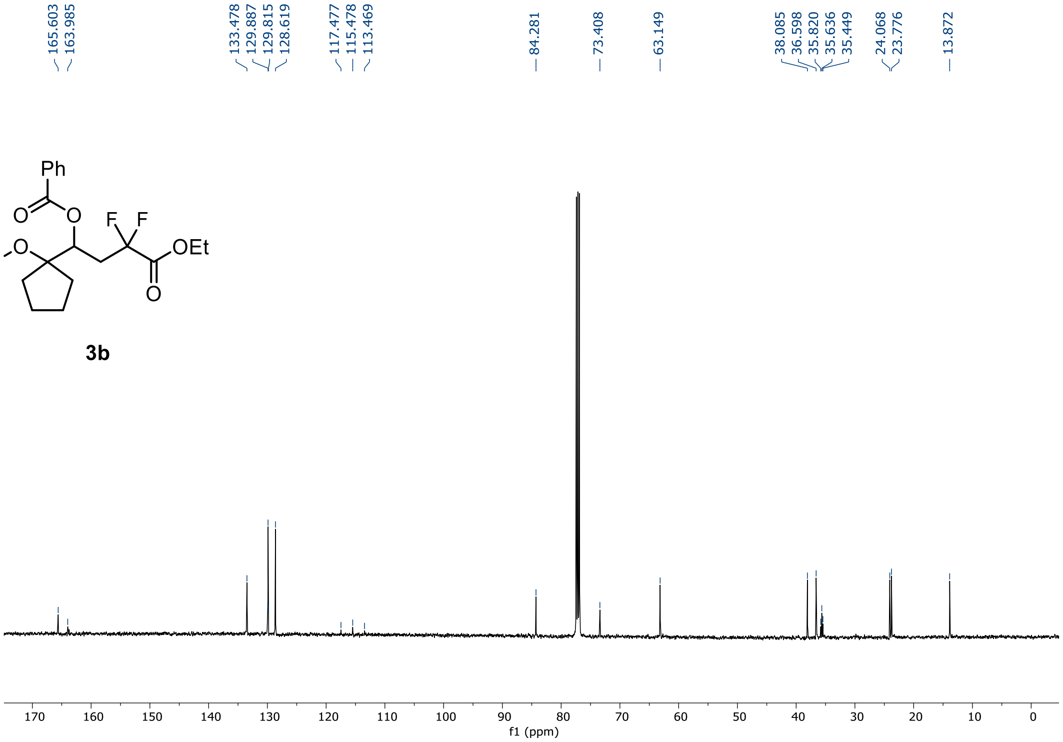

^{19}F NMR (470 MHz, CDCl$_3$**, 25 °C) of (3b)**

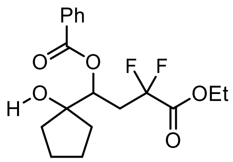

3b

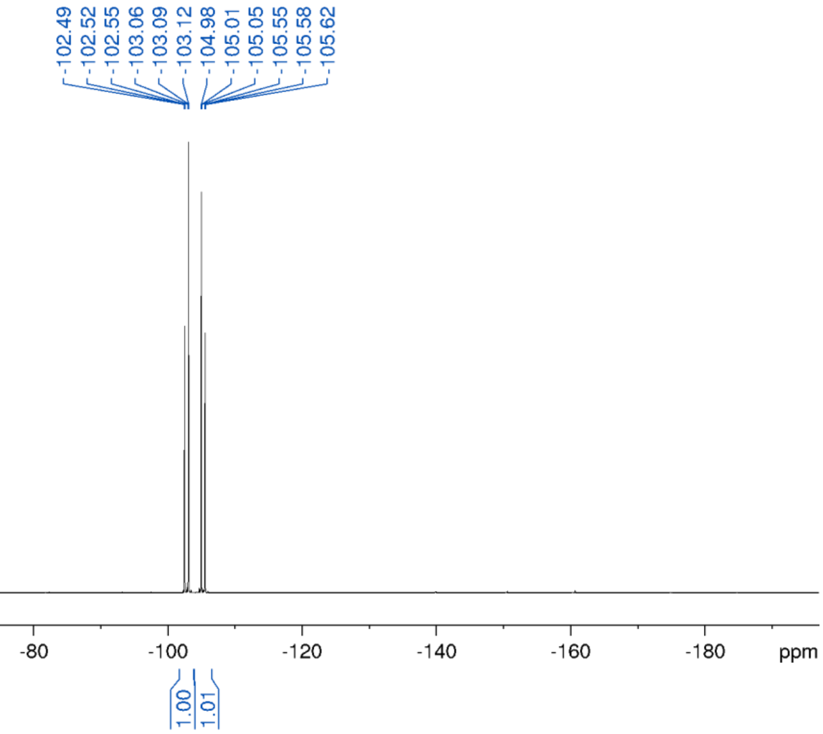

^{1}H NMR (500 MHz, CDCl$_3$**, 25 ºC) of (3c)**

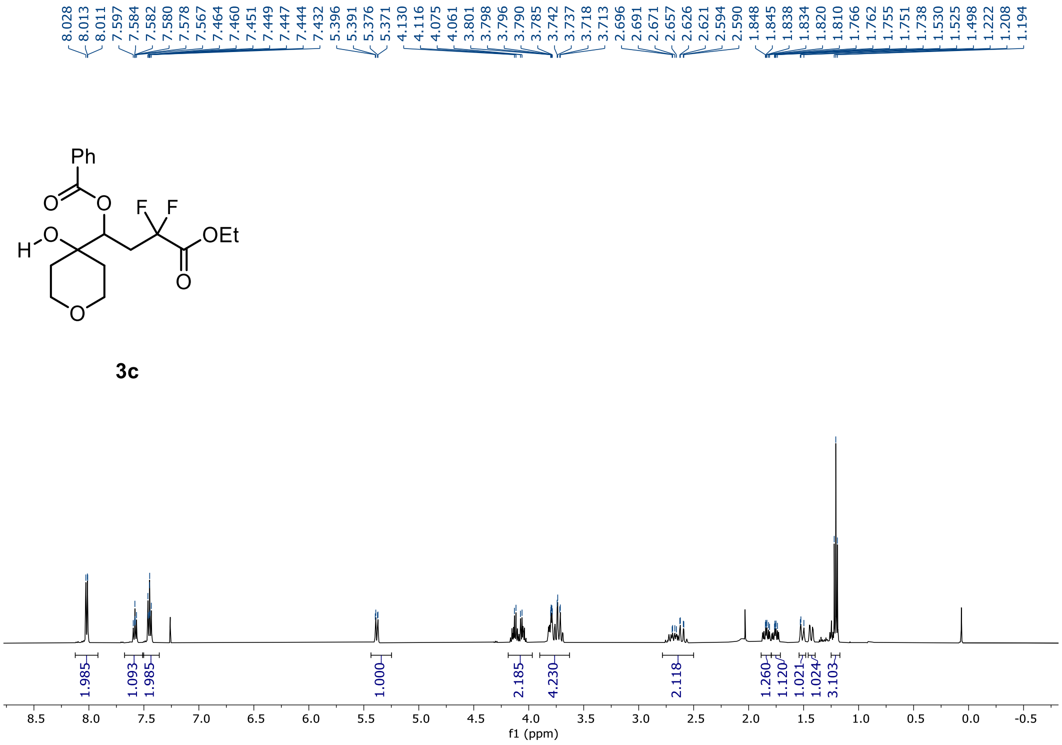

^{13}C NMR (126 MHz, CDCl$_3$**, 25 ºC) of (3c)**

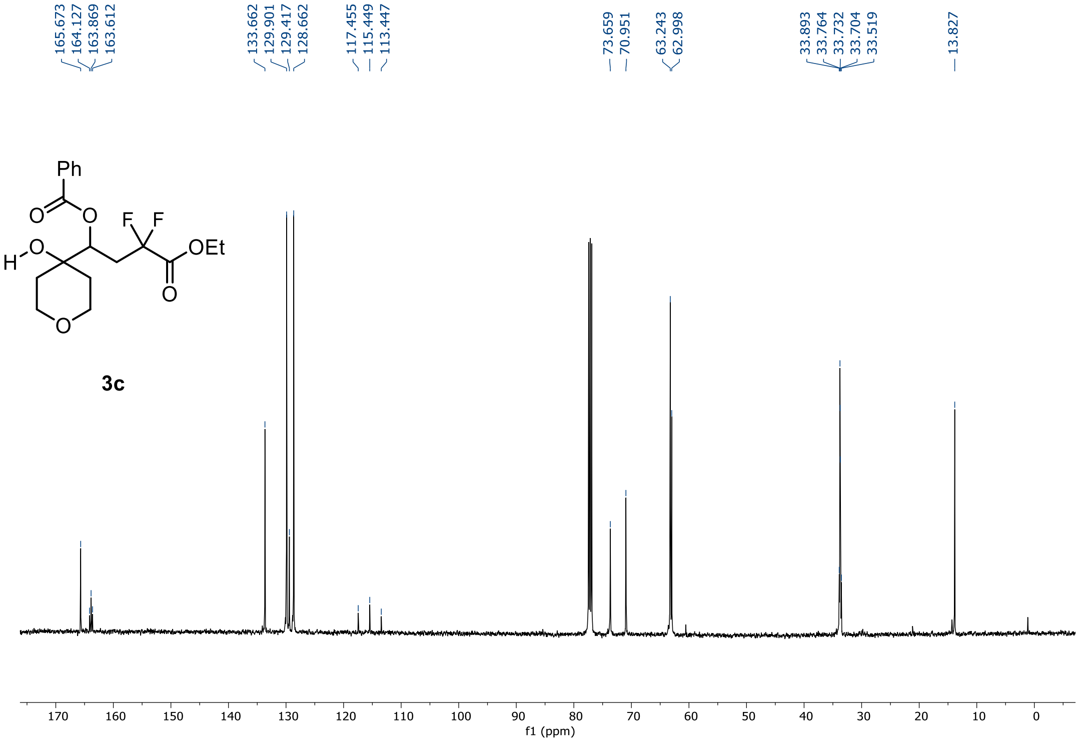

^{19}F NMR (470 MHz, $CDCl_3$**, 25 ºC) of (3c)**

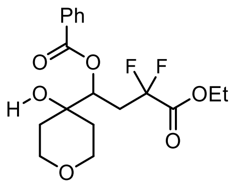

3c

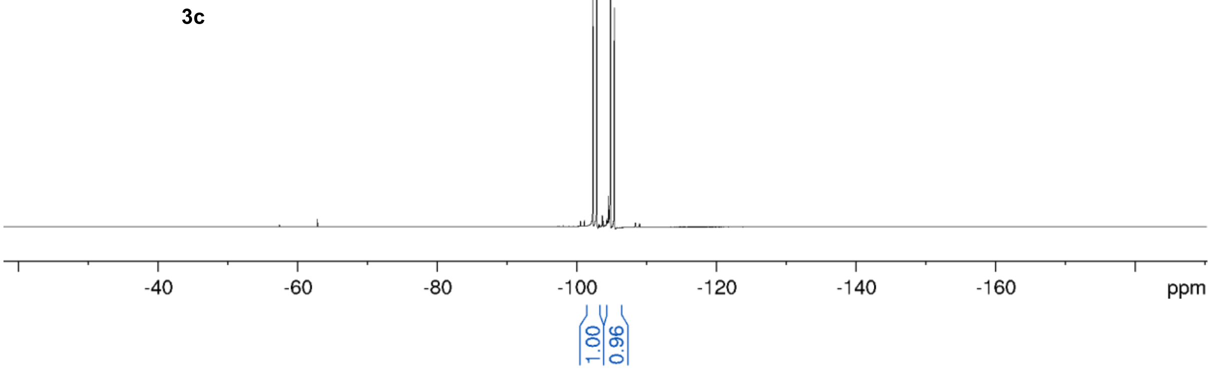

^{1}H NMR (500 MHz, $CDCl_3$**, 25 ºC) of (3d)**

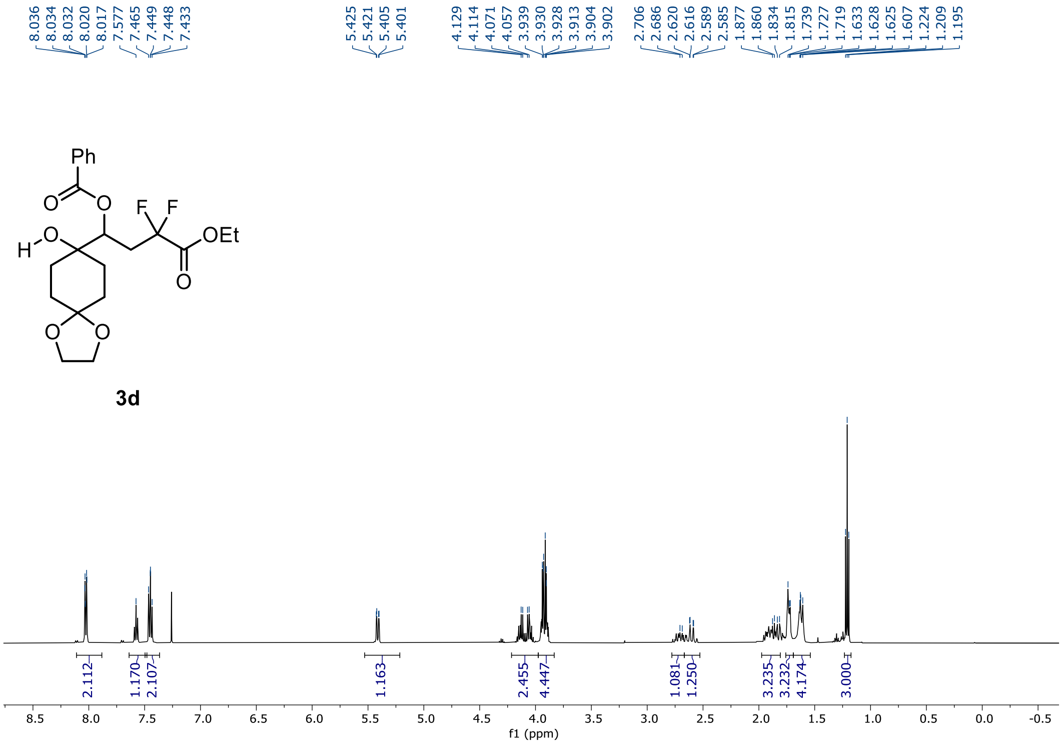

^{13}C NMR (125 MHz, $CDCl_3$**, 25 ºC) of (3d)**

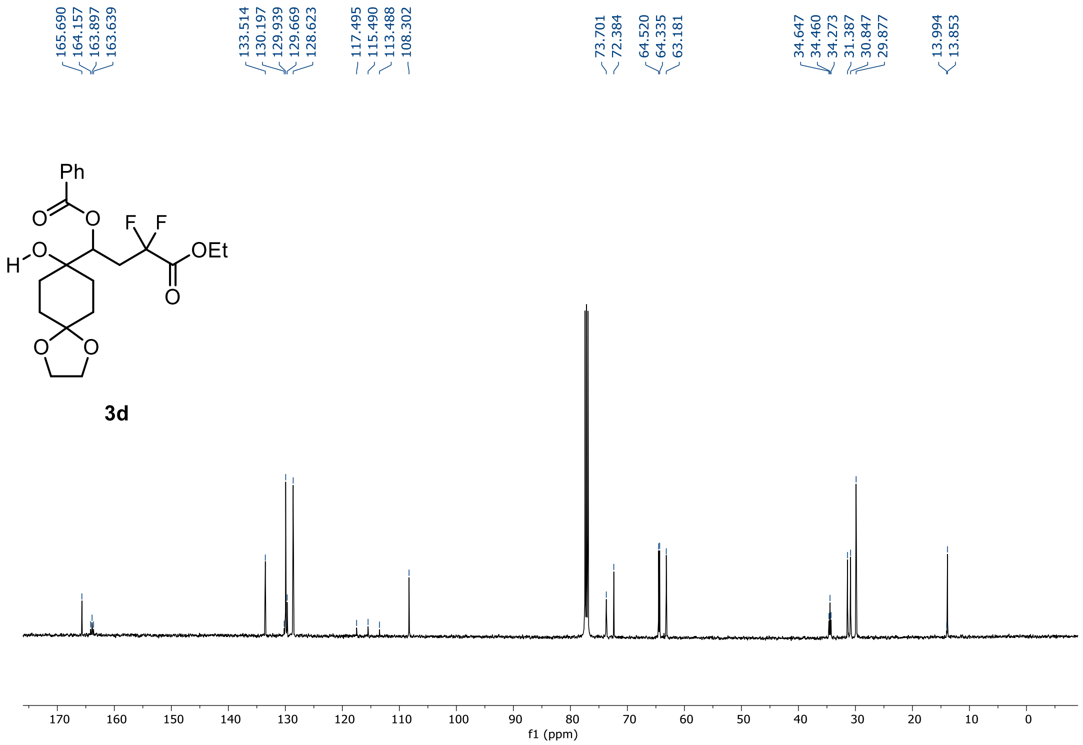

^{19}F NMR (470 MHz, CDCl$_3$**, 25 ºC) of (3d)**

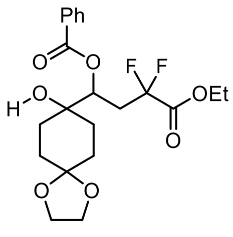

3d

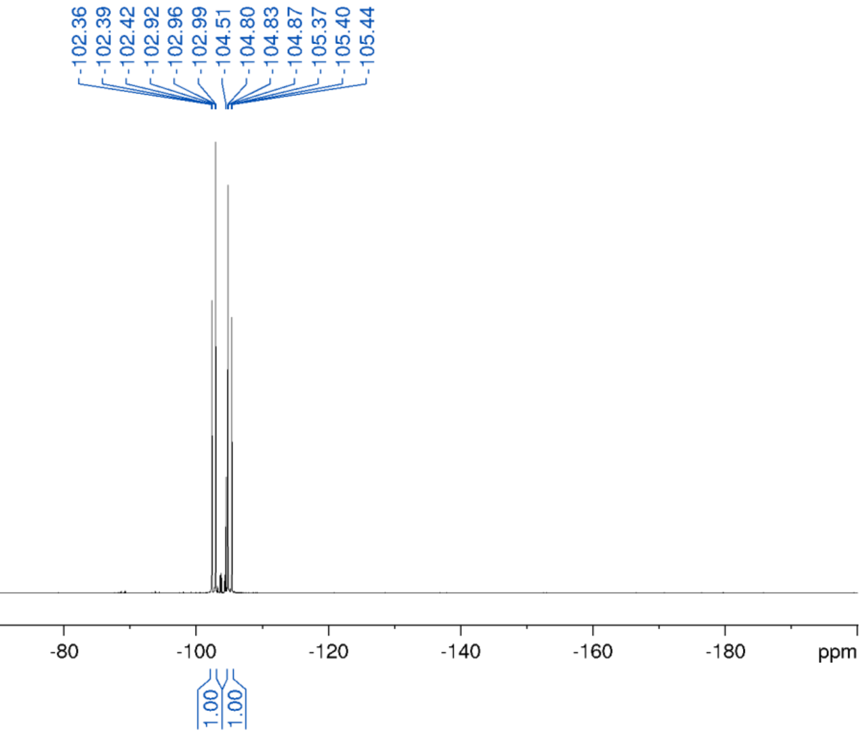

^{1}H NMR (500 MHz, $CDCl_3$, 25 ºC) of (3e)

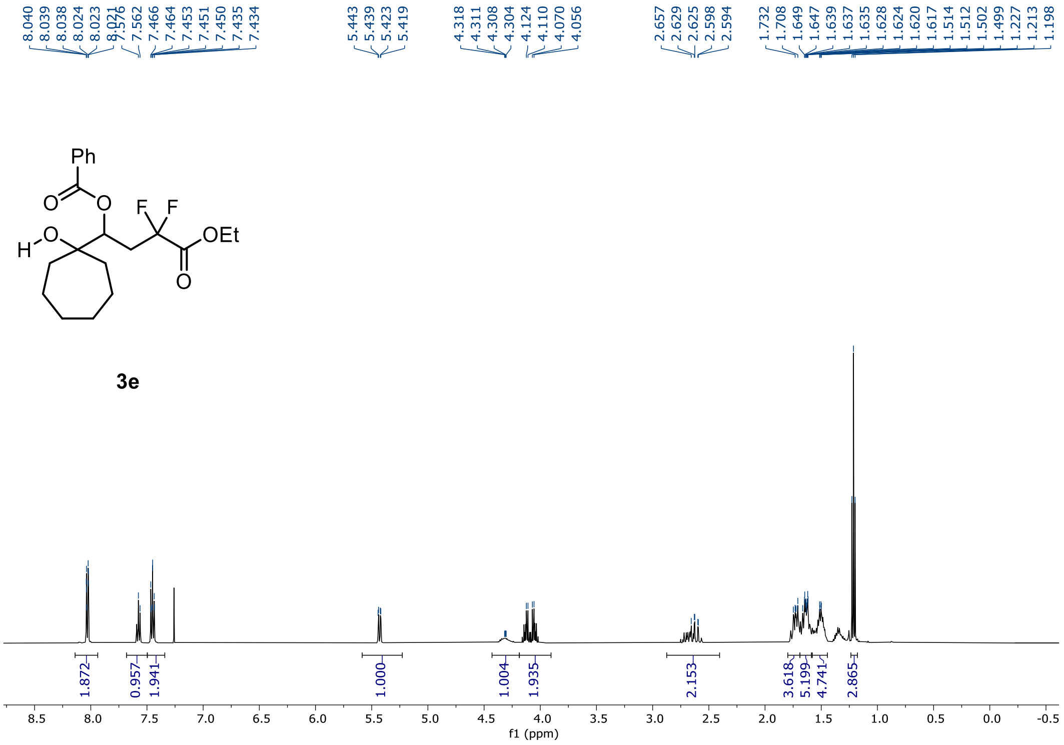

^{13}C NMR (126 MHz, $CDCl_3$, 25 ºC) of (3e)

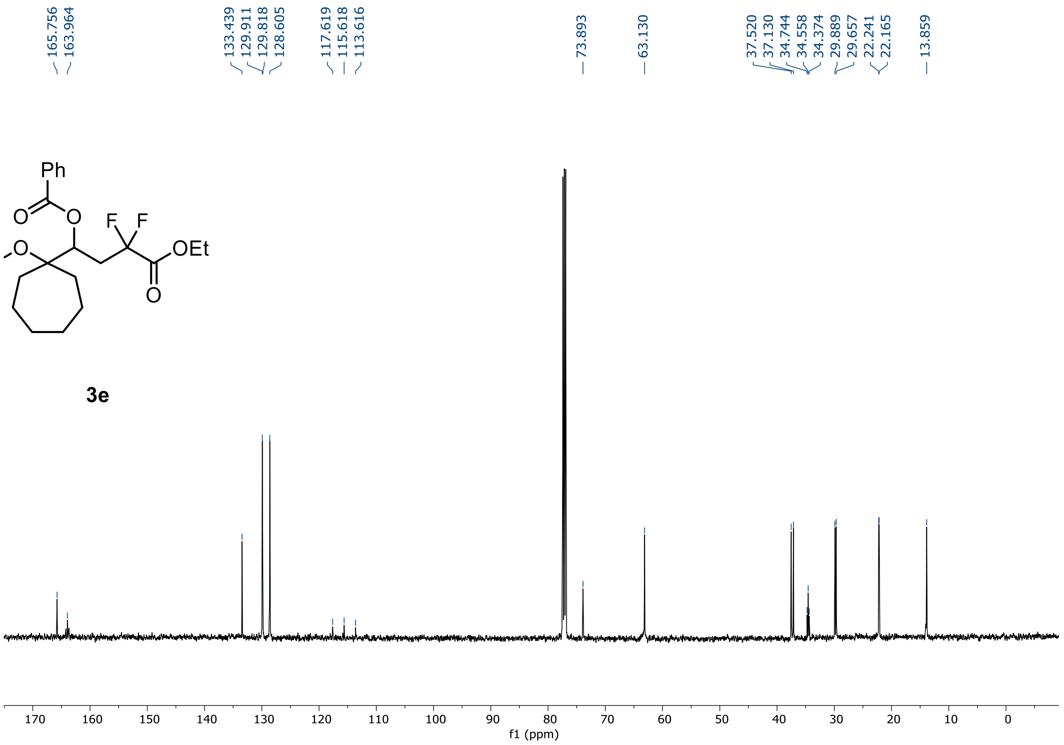

^{19}F NMR (470 MHz, $CDCl_3$, 25 ºC) of (3e)

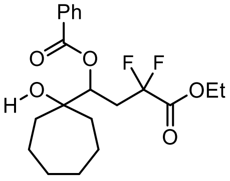
Ph
O
O
F
F
H
O
OEt
O

3e

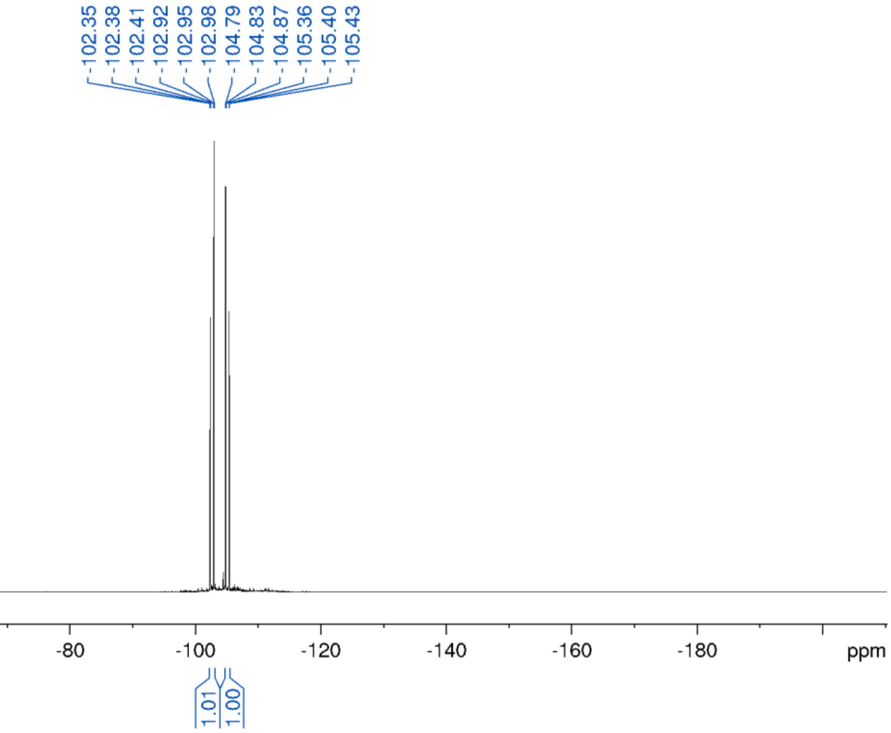
-102.35
-102.38
-102.41
-102.92
-102.95
-102.98
-104.79
-104.83
-104.87
-105.36
-105.40
-105.43
-40
-60
-80
-100
-120
-140
-160
-180
ppm
1.01
1.00

^{1}H NMR (500 MHz, $CDCl_3$, 25 ºC) of (3f)

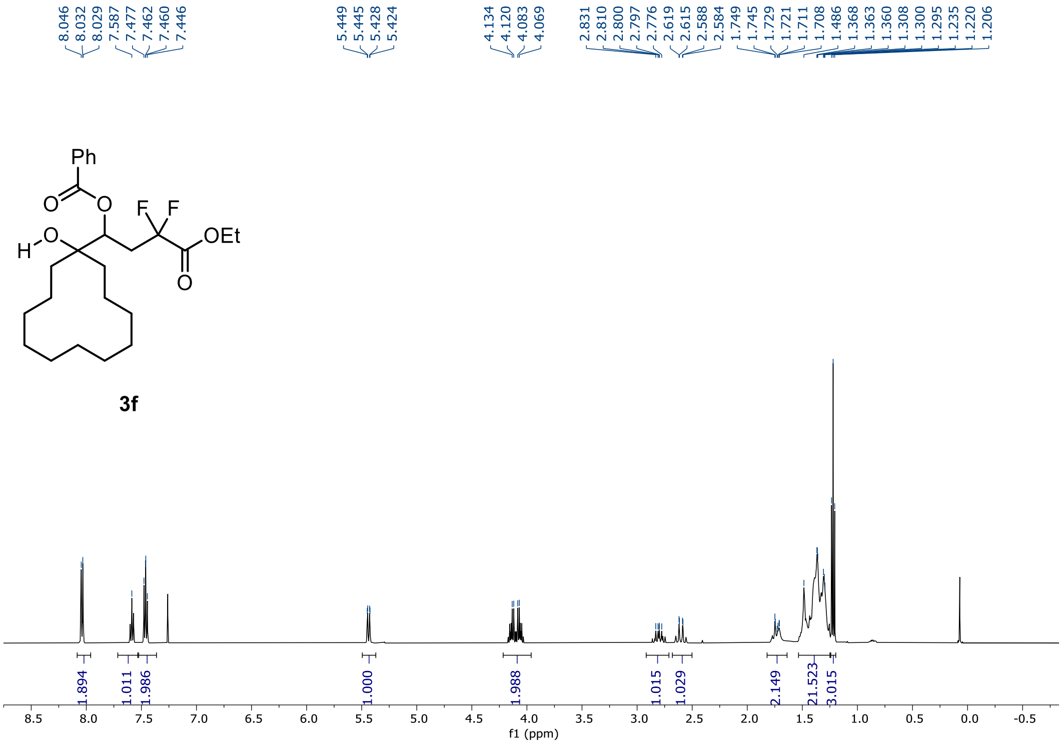

^{13}C NMR (126 MHz, $CDCl_3$, 25 ºC) of (3f)

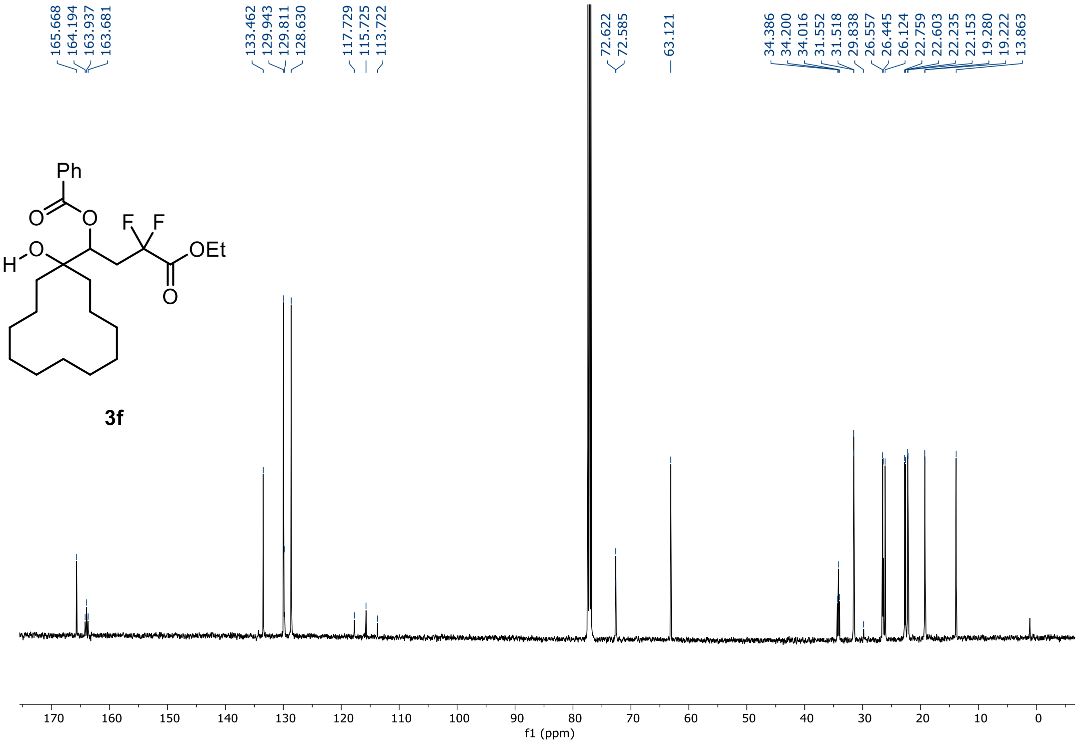

^{19}F NMR (376 MHz, $CDCl_3$**, 25 ºC) of (3f)**

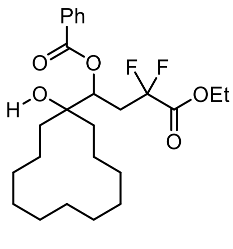

3f

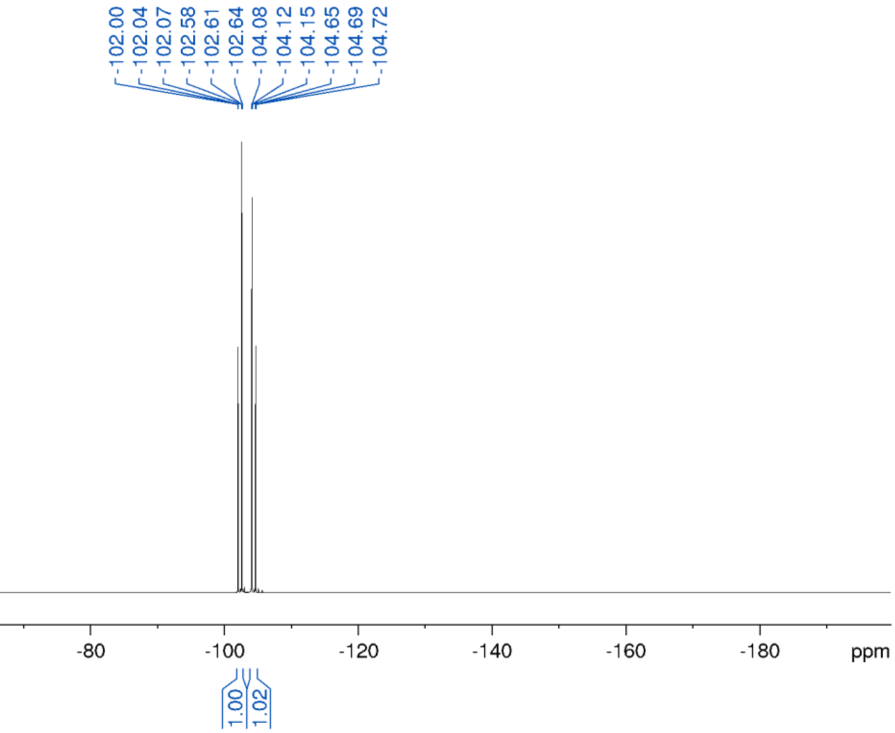

^{1}H NMR (500 MHz, CDCl$_3$, 25 ºC) of (3g)

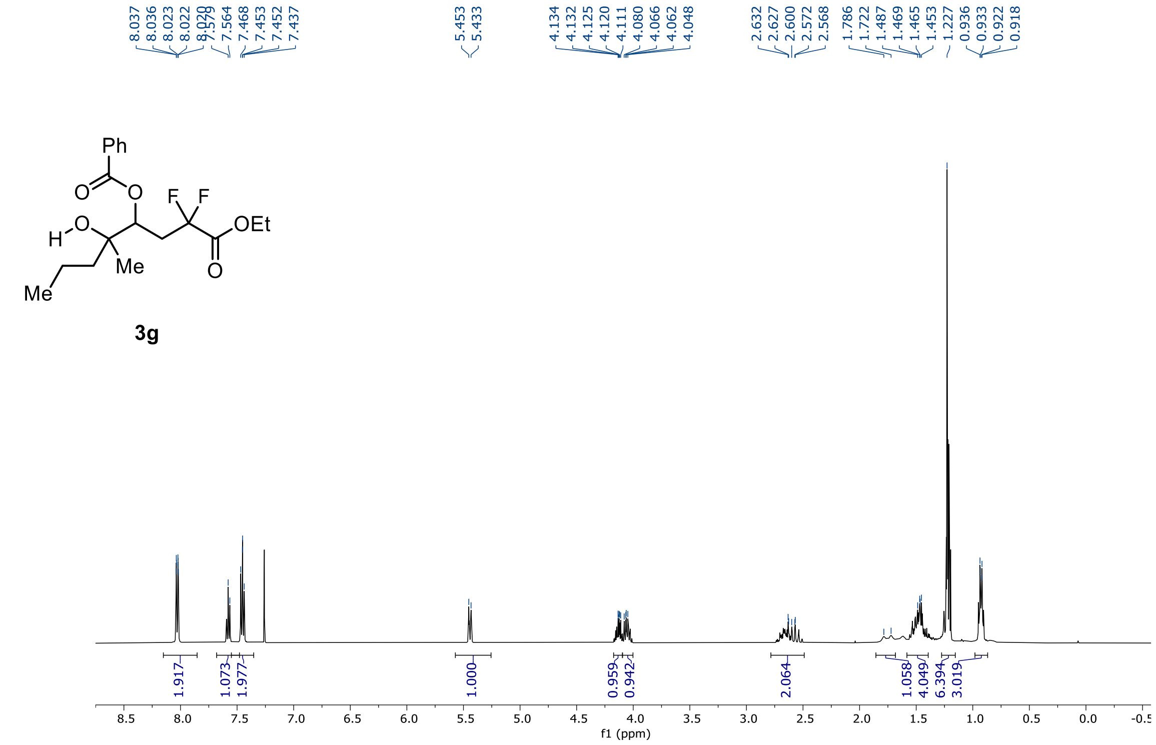

^{13}C NMR (126 MHz, CDCl$_3$, 25 ºC) of (3g)

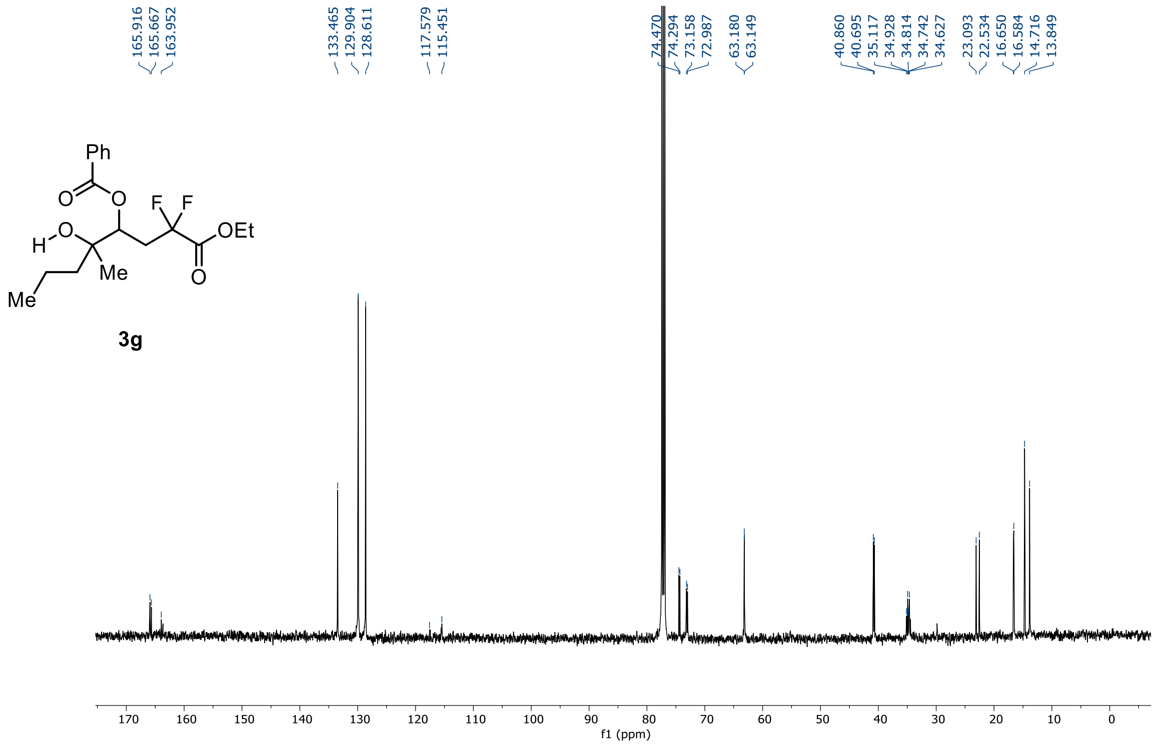

^{19}F NMR (470 MHz, CDCl$_3$**, 25 ºC) of (3g)**

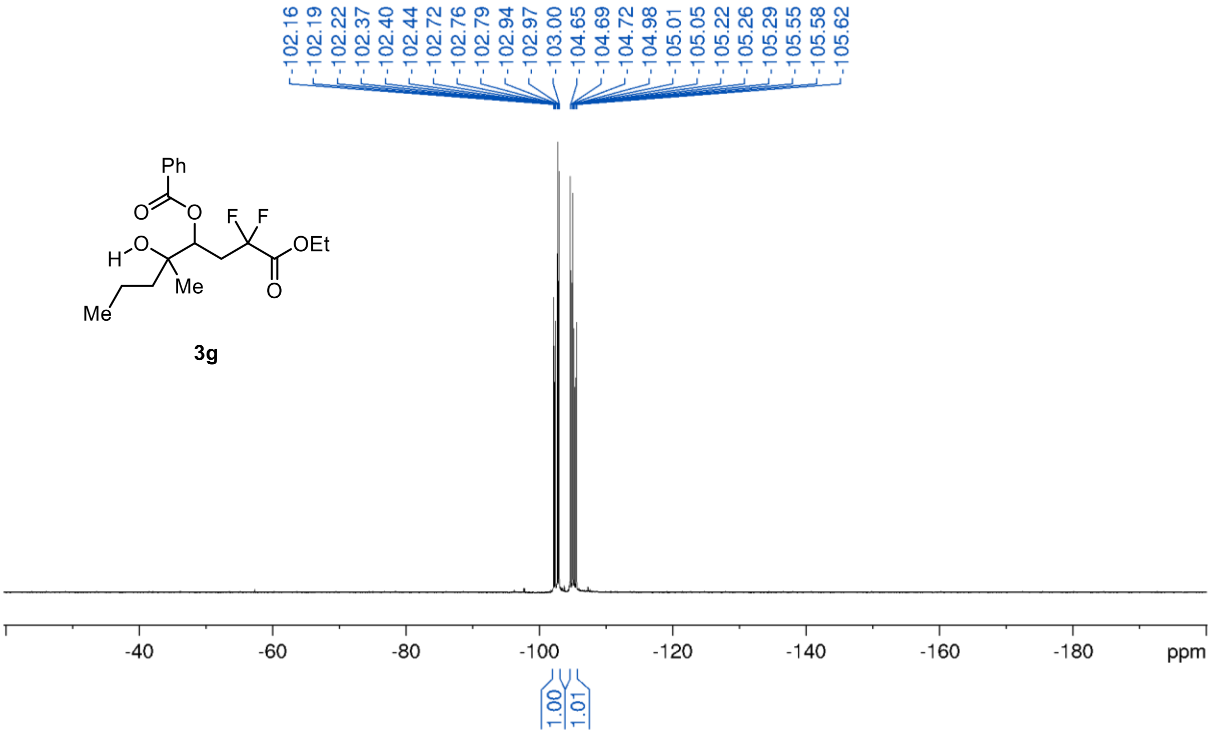

3g

^{1}H NMR (500 MHz, CDCl$_3$, **25 ºC) of (3h)**

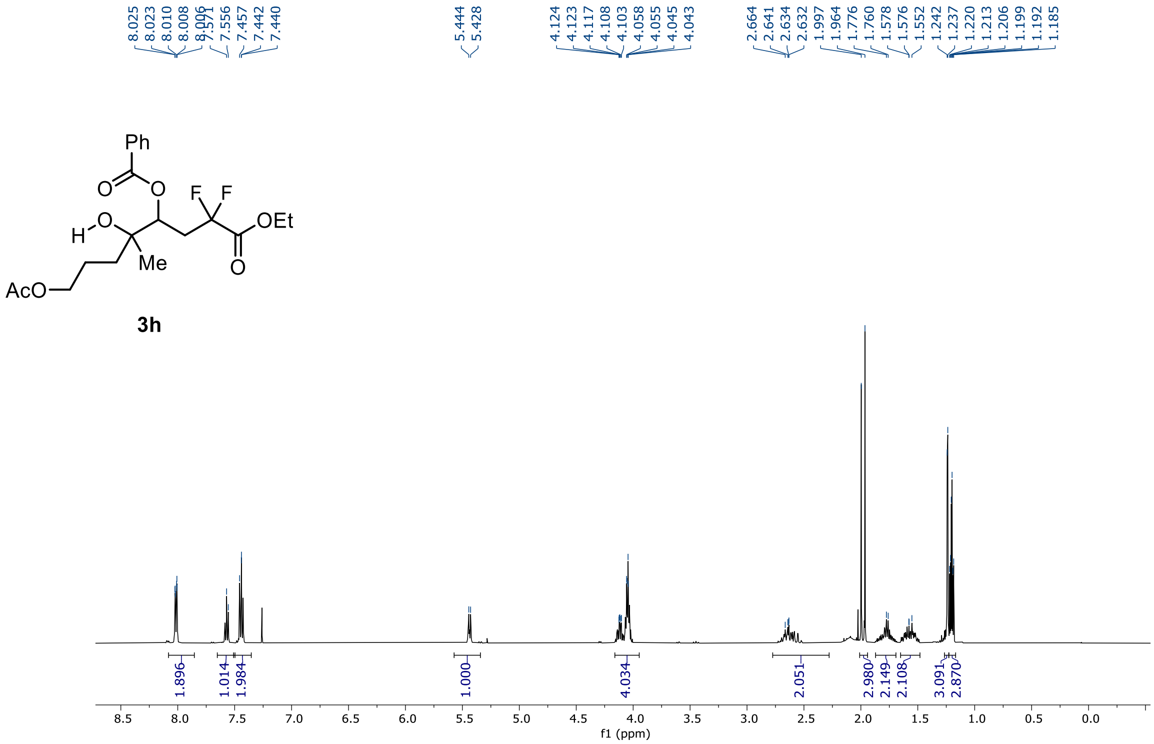

^{13}C NMR (126 MHz, CDCl$_3$, **25 ºC) of (3h)**

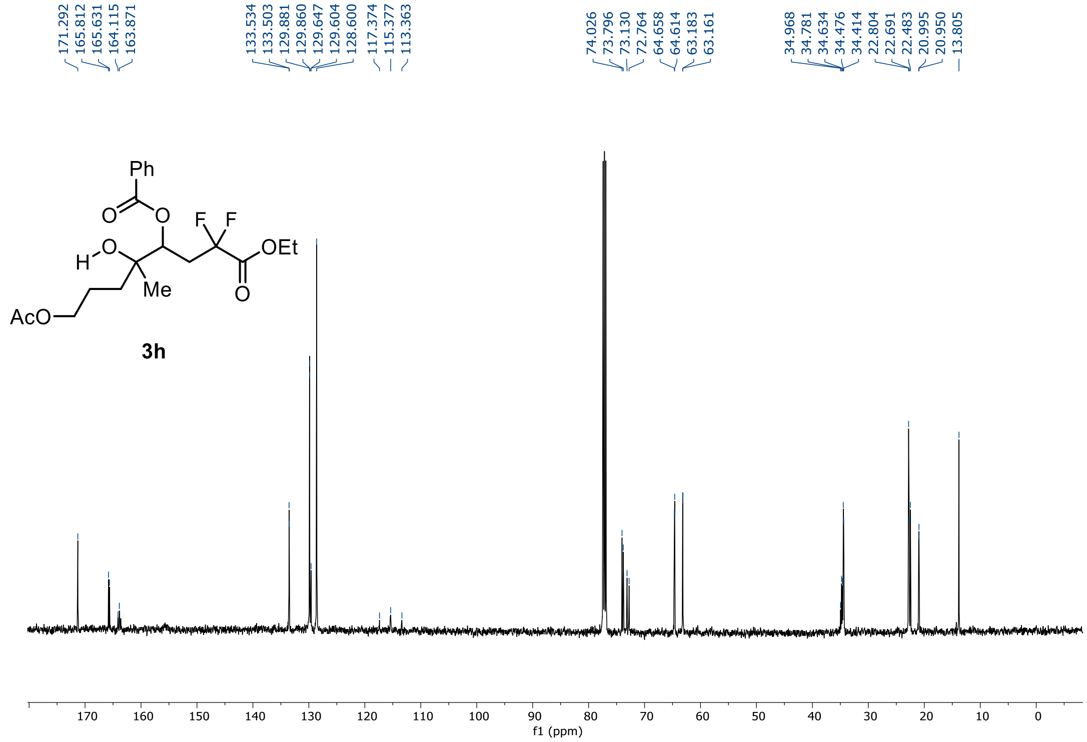

^{19}F NMR (470 MHz, CDCl$_3$**, 25 ºC) of (3h)**

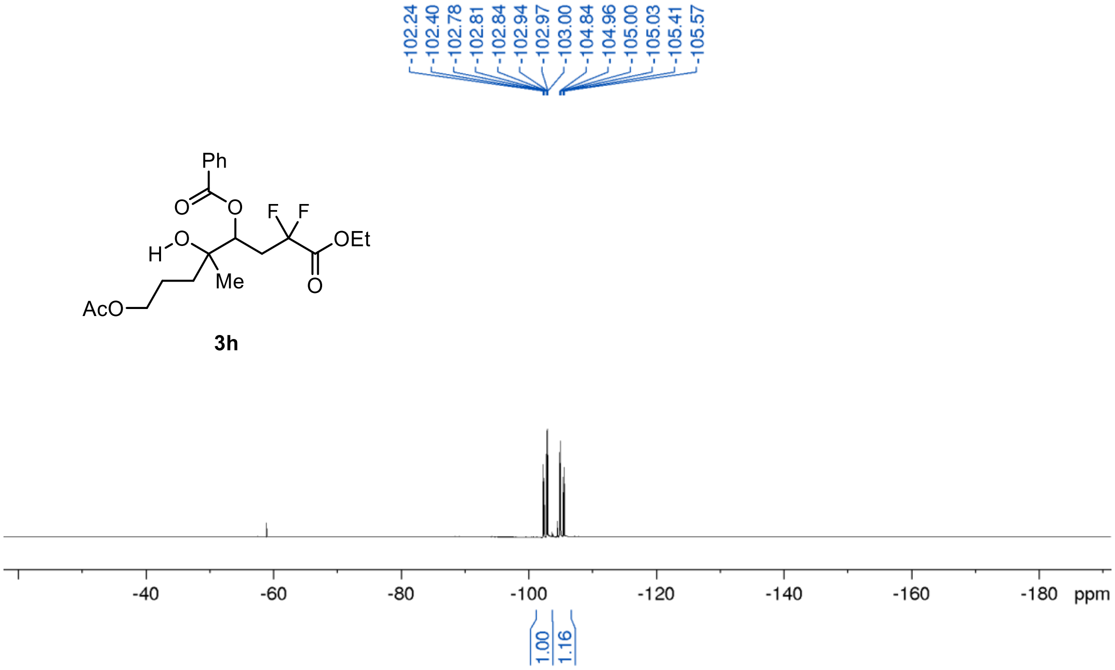

^{1}H NMR (500 MHz, $CDCl_3$, 25 ºC) of (3i)

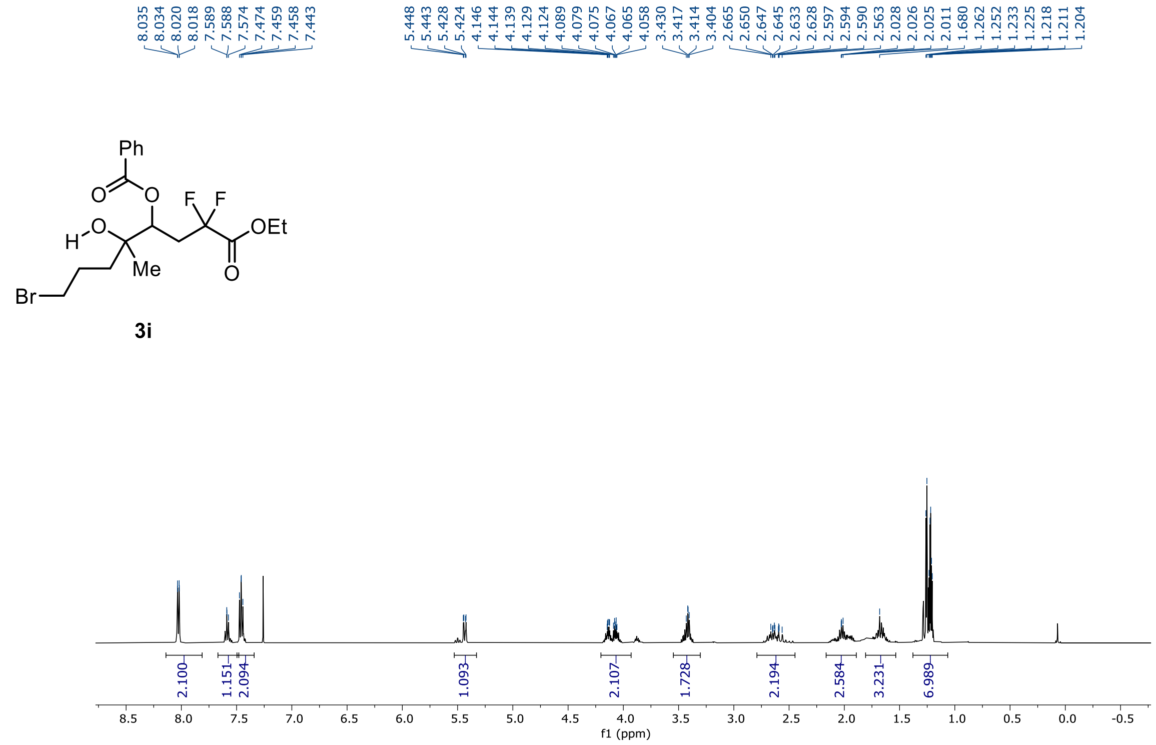

^{13}C NMR (126 MHz, $CDCl_3$, 25 ºC) of (3i)

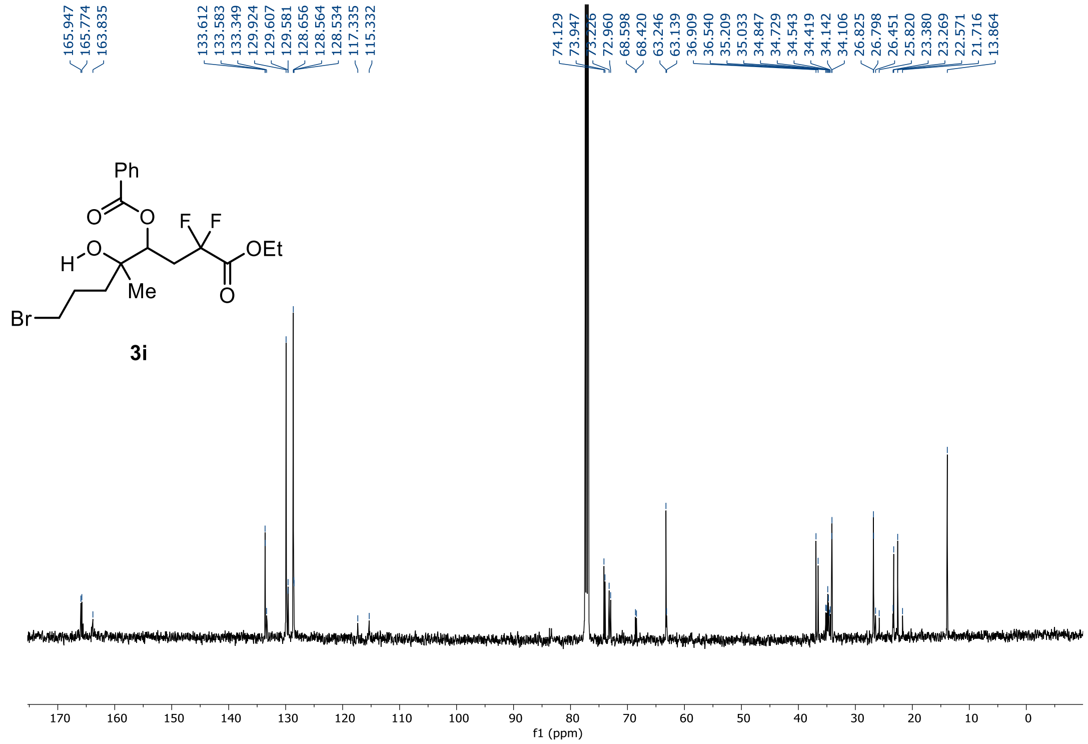

^{19}F NMR (376 MHz, CDCl$_3$**, 25 ºC) of (3i)**

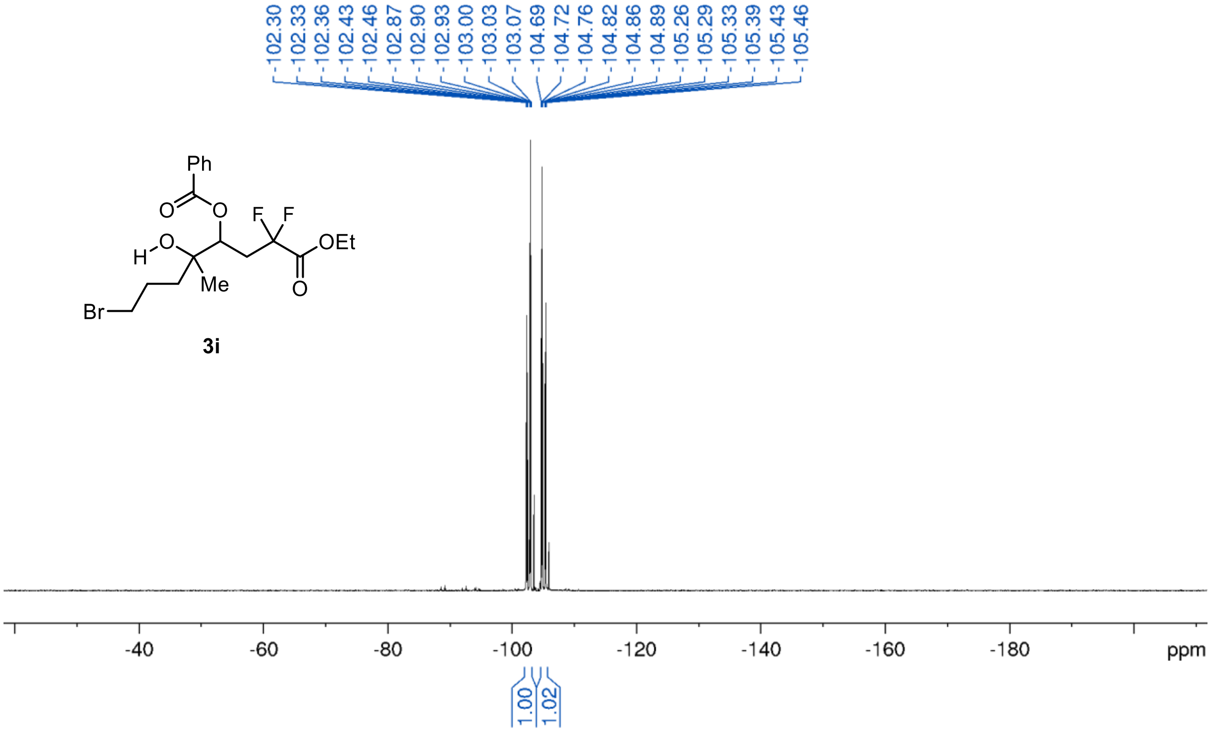

^{1}H NMR (500 MHz, $CDCl_3$, 25 ºC) of (3j)

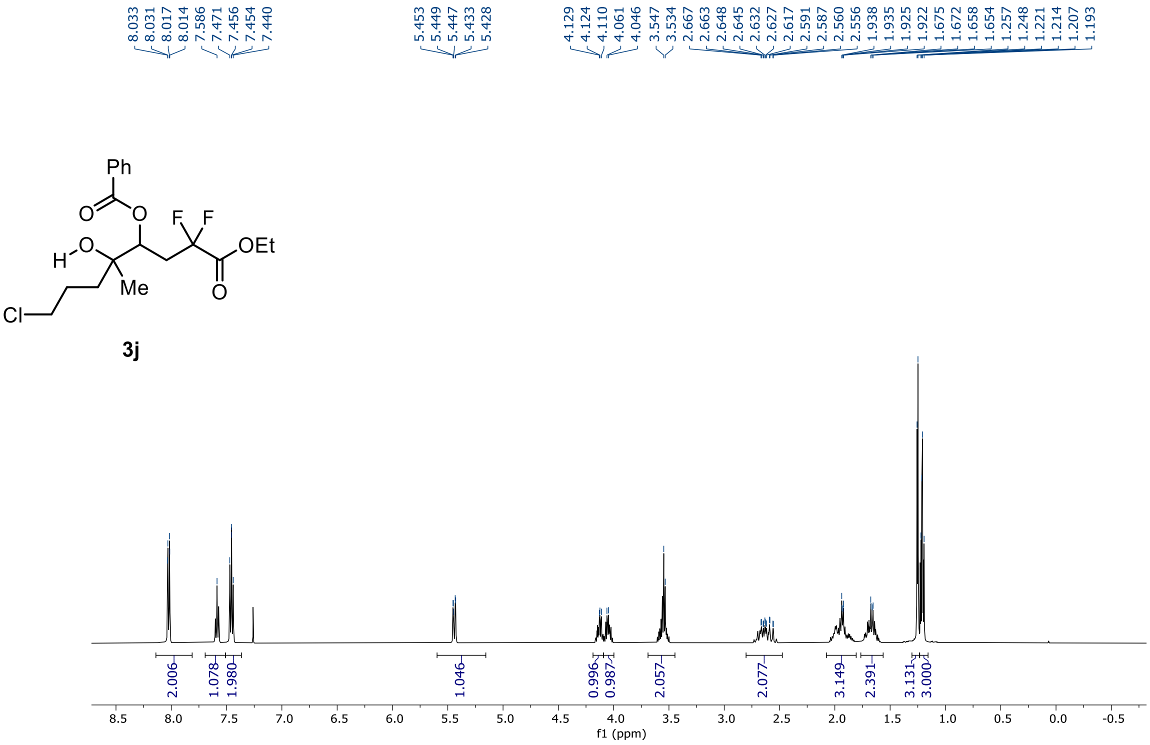

^{13}C NMR (126 MHz, $CDCl_3$, 25 ºC) of (3j)

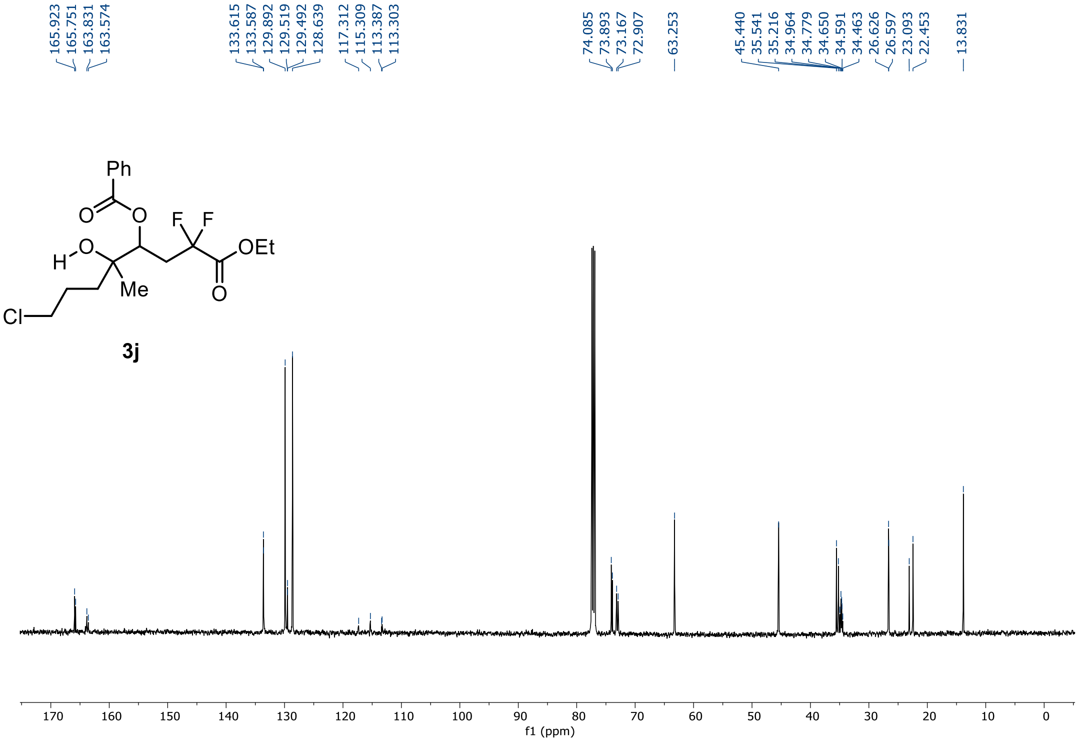

^{19}F NMR (470 MHz, CDCl$_3$, 25 ºC) of (3j)

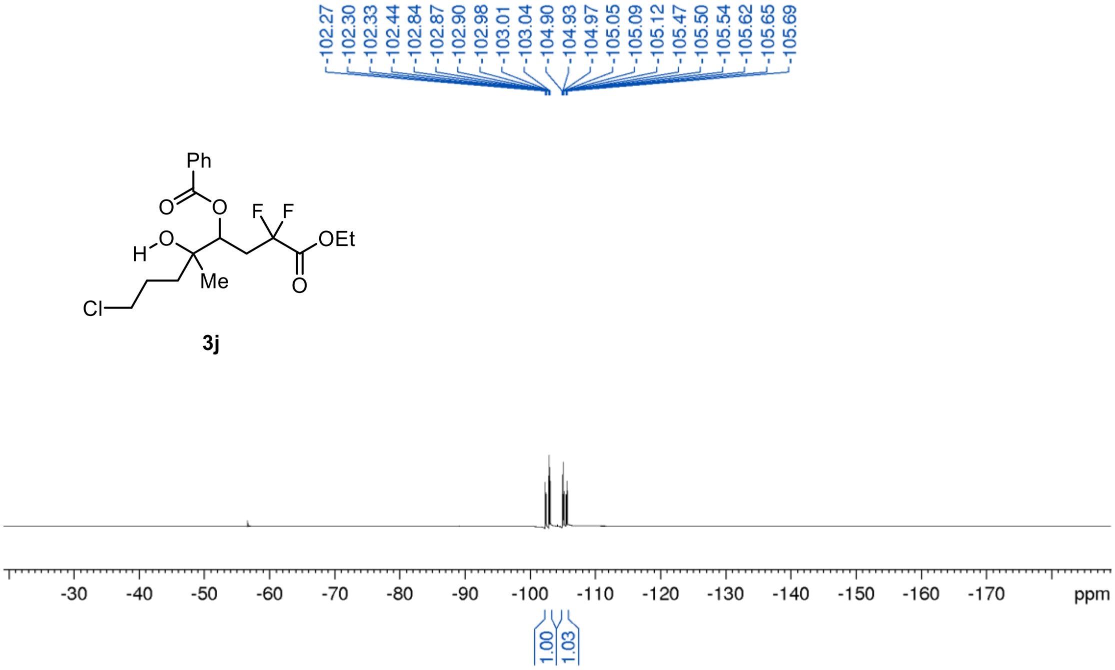

^{1}H NMR (500 MHz, $CDCl_3$, 25 ºC) of (3k)

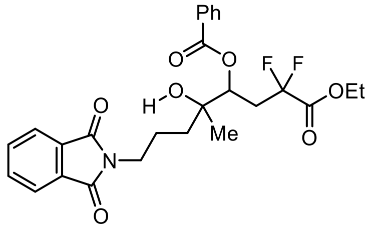

3k

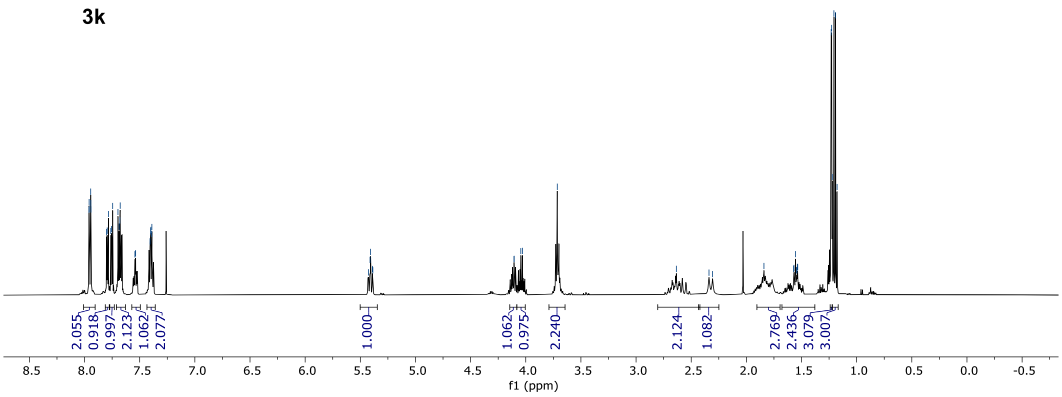

^{13}C NMR (126 MHz, $CDCl_3$, 25 ºC) of (3k)

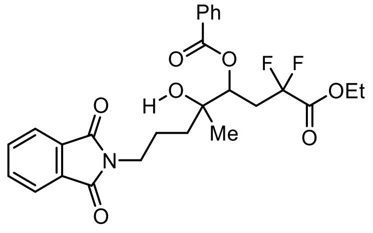

3k

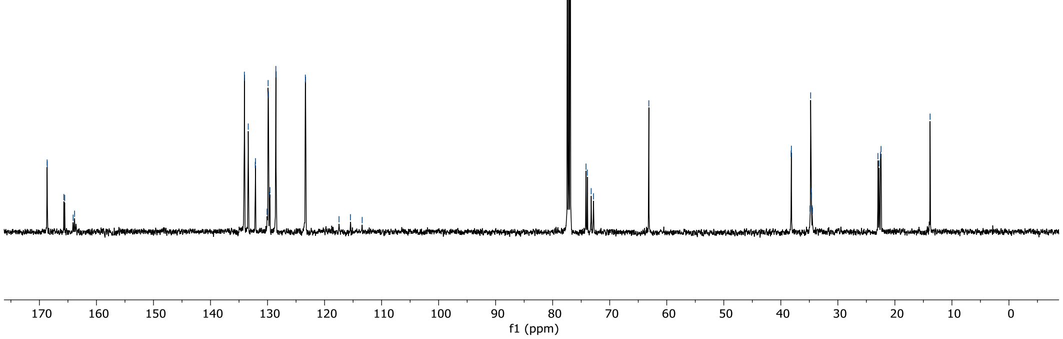

^{19}F NMR (470 MHz, CDCl$_3$**, 25 ºC) of (3k)**

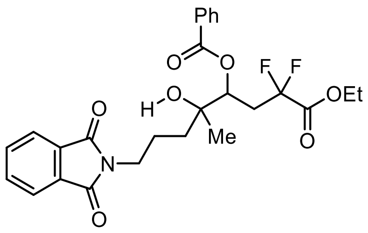

3k

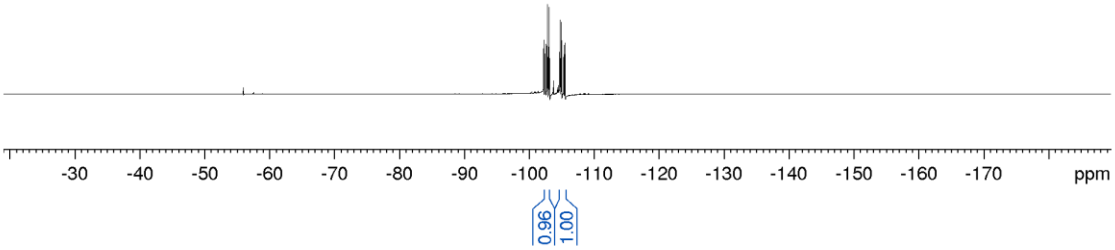

^{1}H NMR (500 MHz, $CDCl_3$, 25 ºC) of (3l)

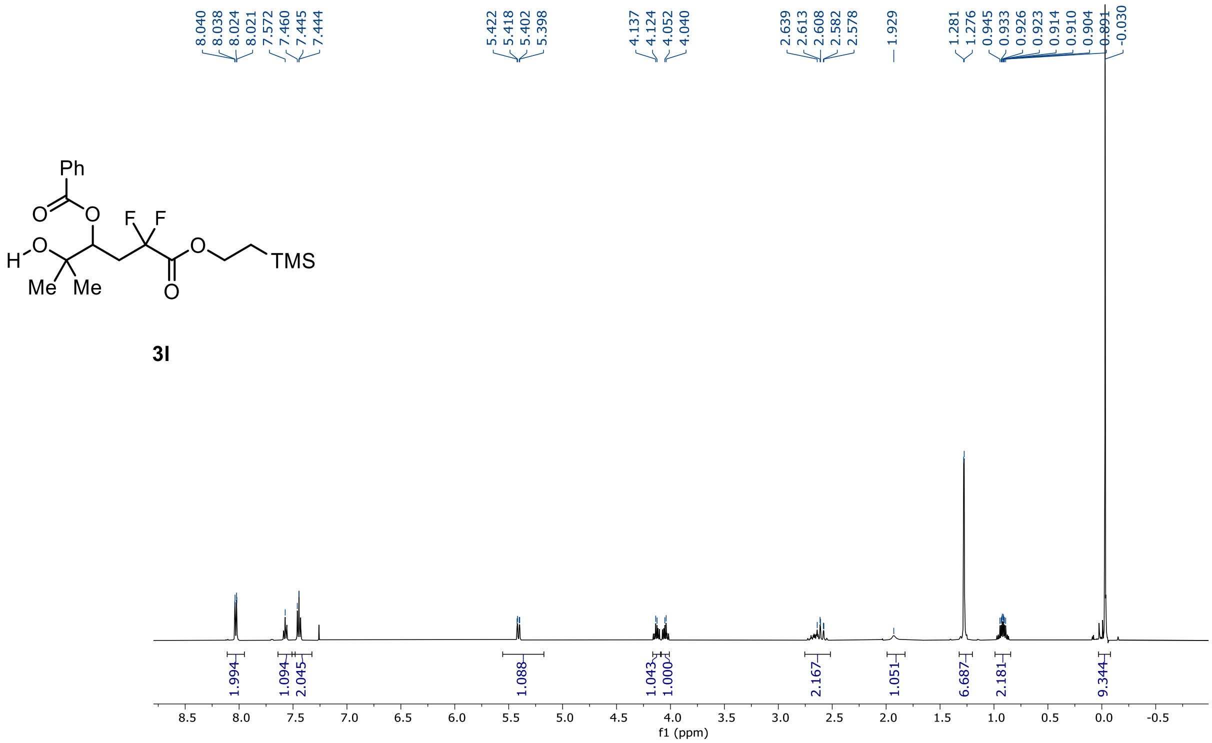

^{13}C NMR (126 MHz, $CDCl_3$, 25 ºC) of (3l)

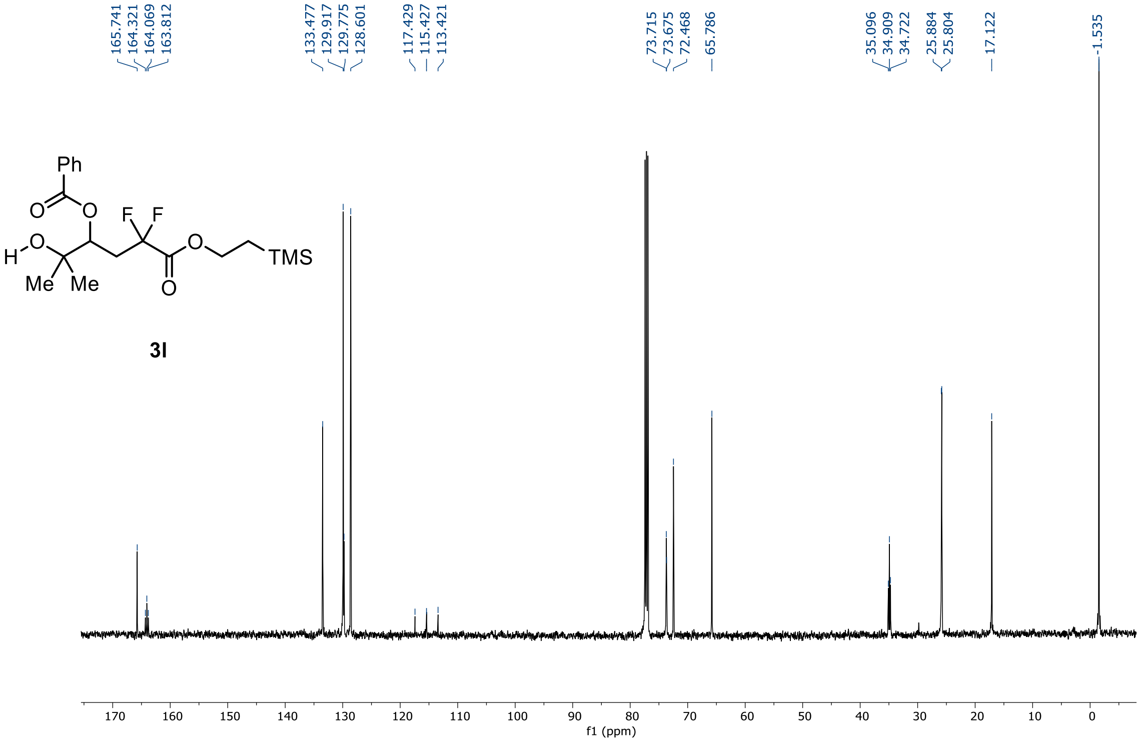

^{19}F NMR (470 MHz, CDCl$_3$**, 25 ºC) of (3l)**

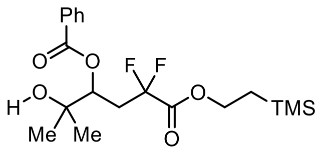

3l

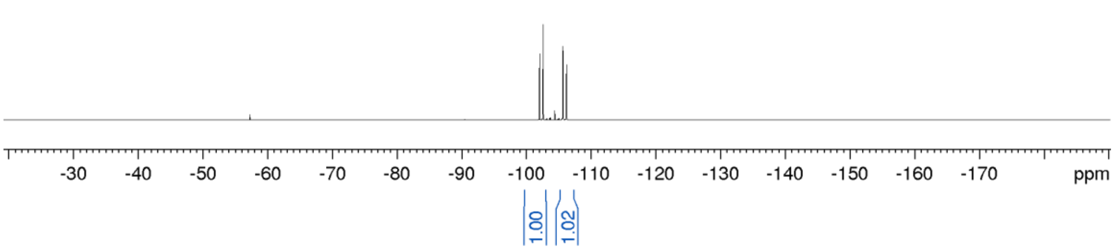

^{1}H NMR (500 MHz, $CDCl_3$, 25 ºC) of (3m)

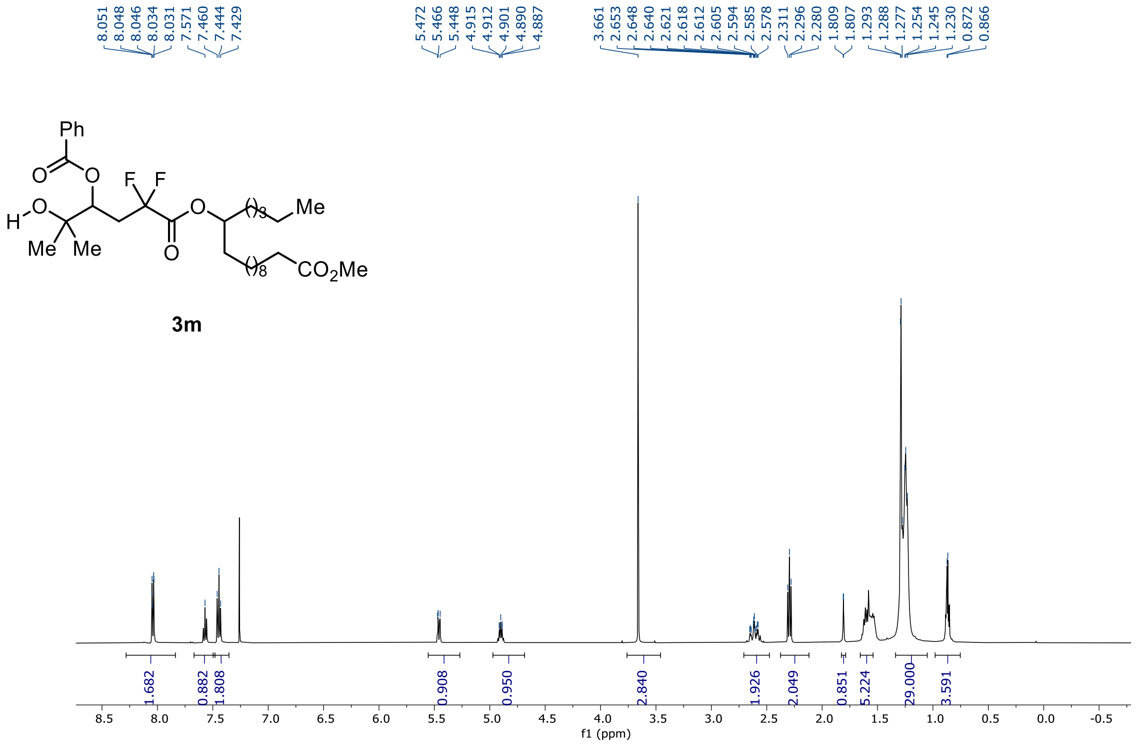

^{13}C NMR (126 MHz, $CDCl_3$, 25 ºC) of (3m)

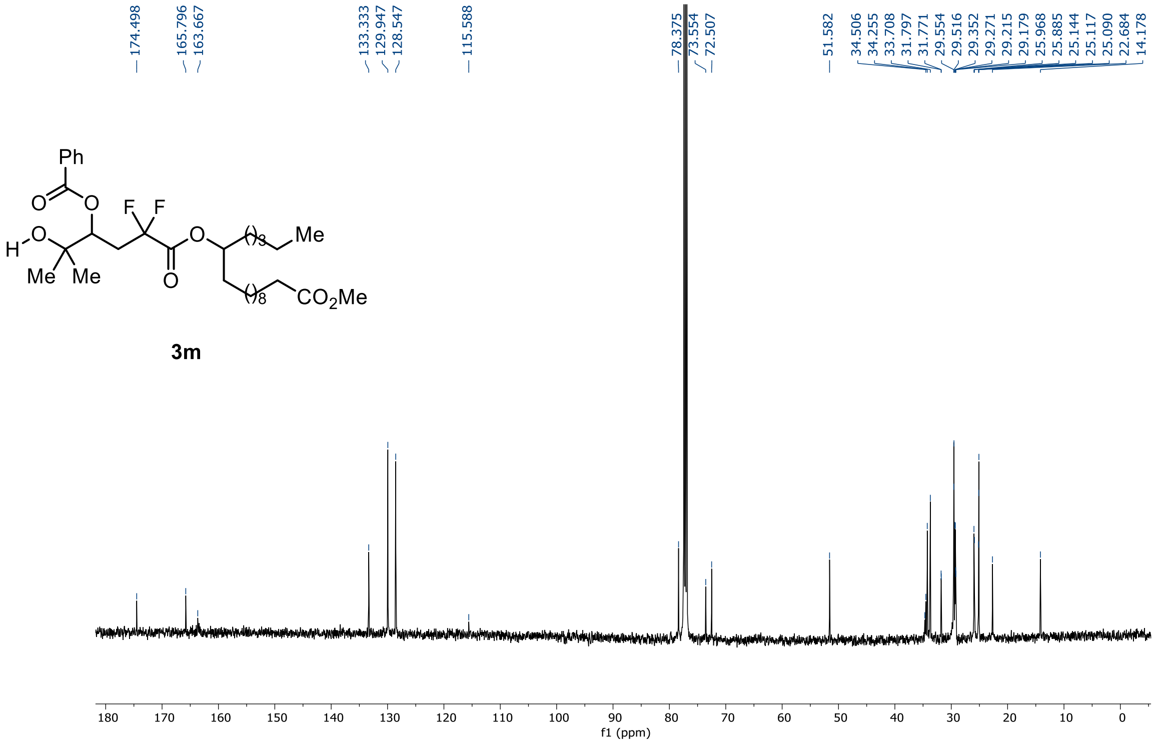

^{19}F NMR (470 MHz, CDCl$_3$**, 25 ºC) of (3m)**

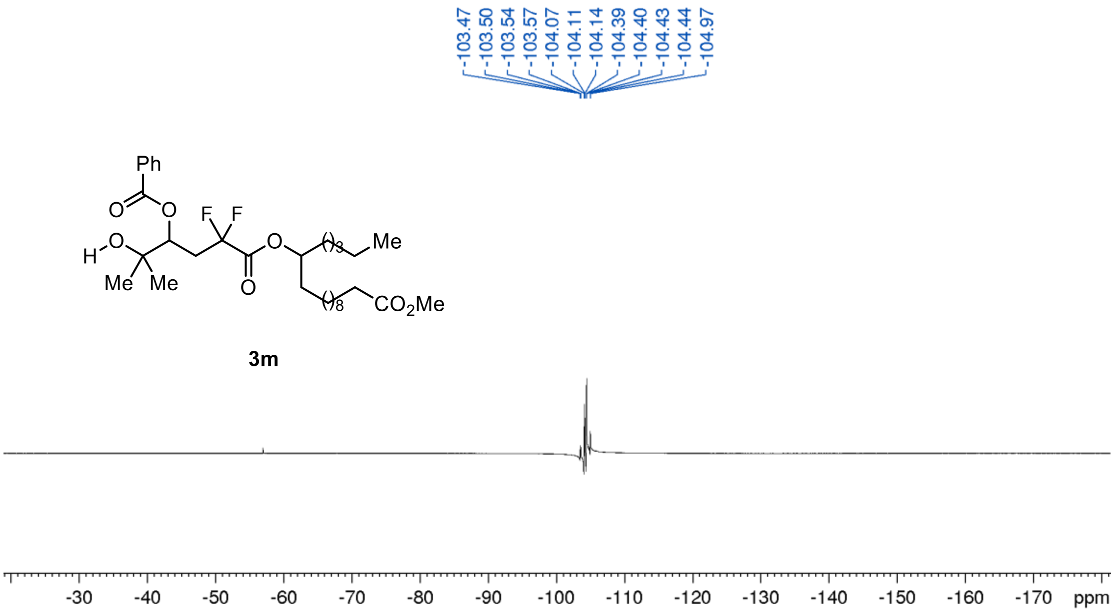

^{1}H NMR (500 MHz, $CDCl_3$, 25 ºC) of (3n)

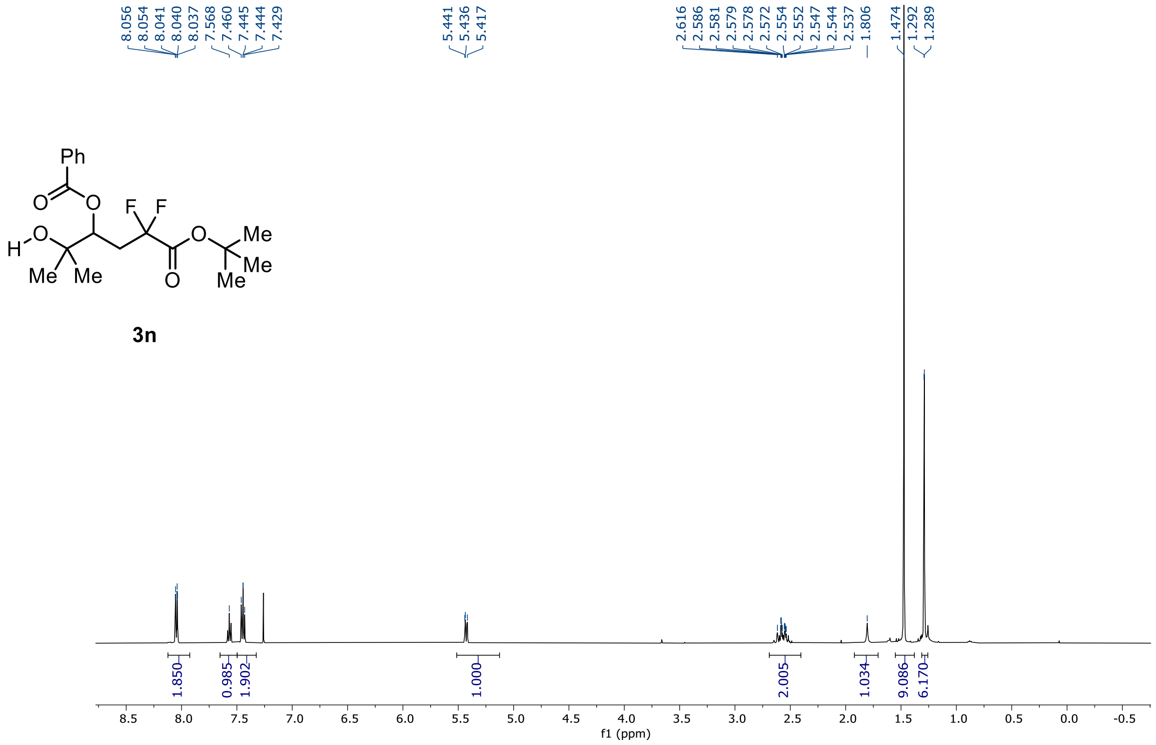

^{13}C NMR (126 MHz, $CDCl_3$, 25 ºC) of (3n)

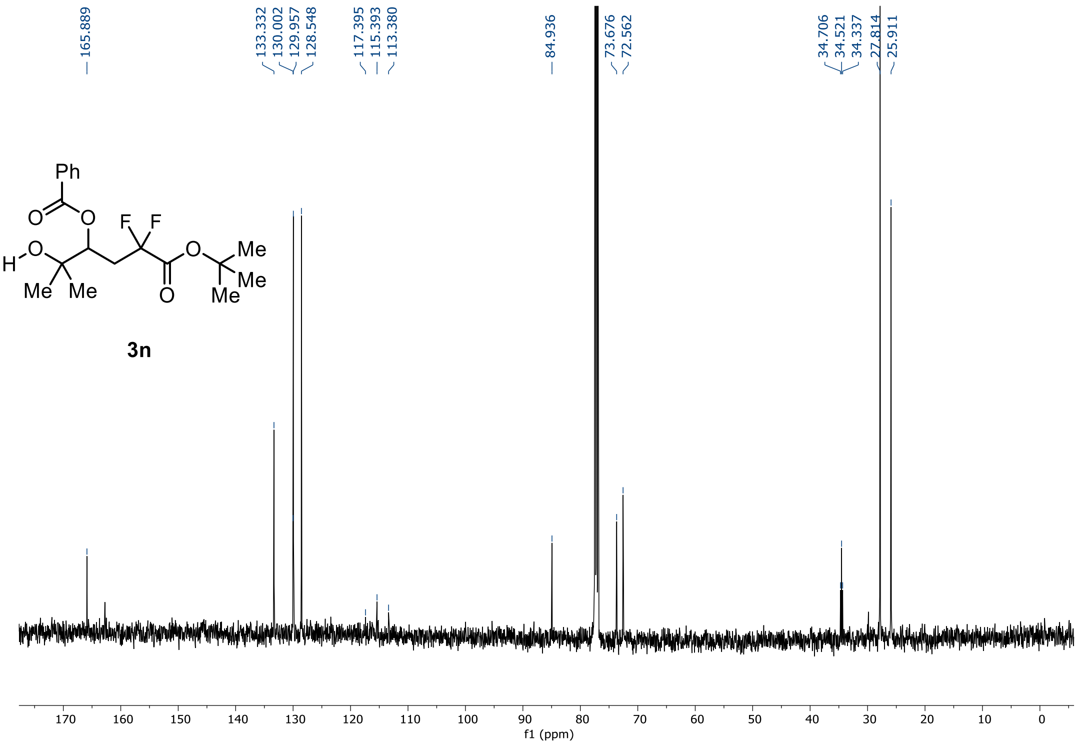

^{19}F NMR (470 MHz, CDCl$_3$**, 25 ºC) of (3n)**

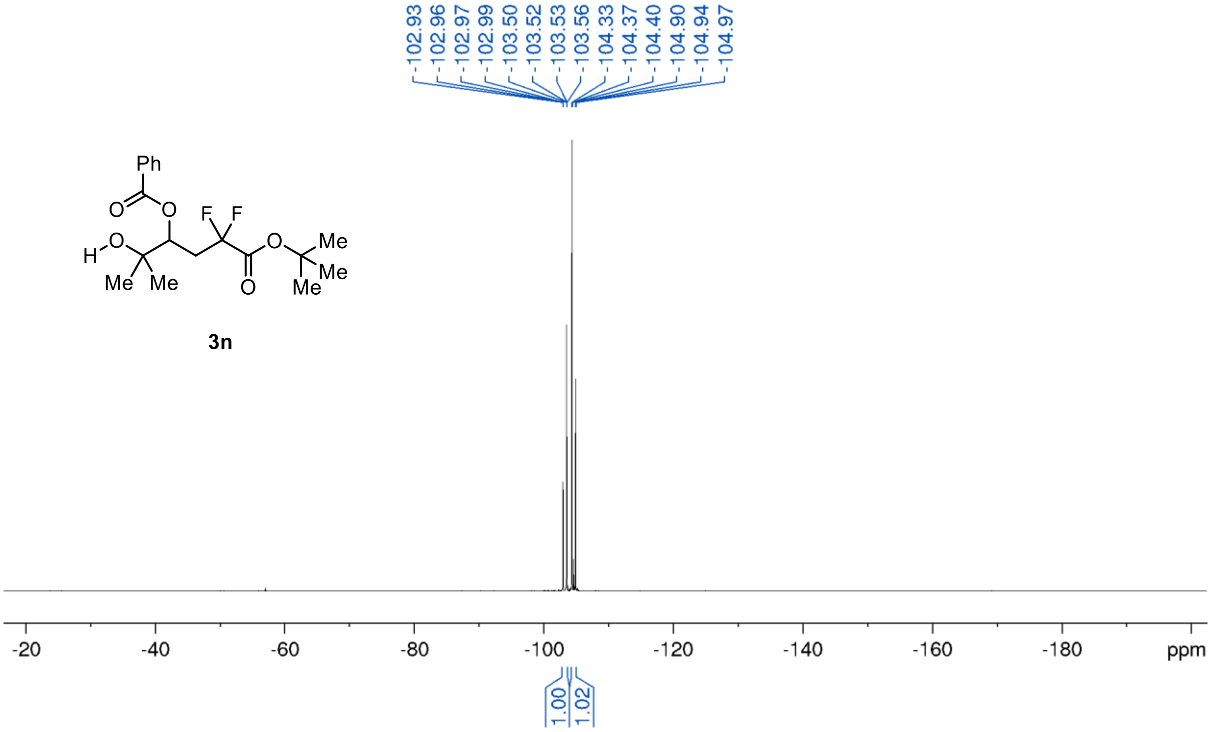

3n

^{1}H NMR (500 MHz, CDCl$_3$**, 25 ºC) of (3o)**

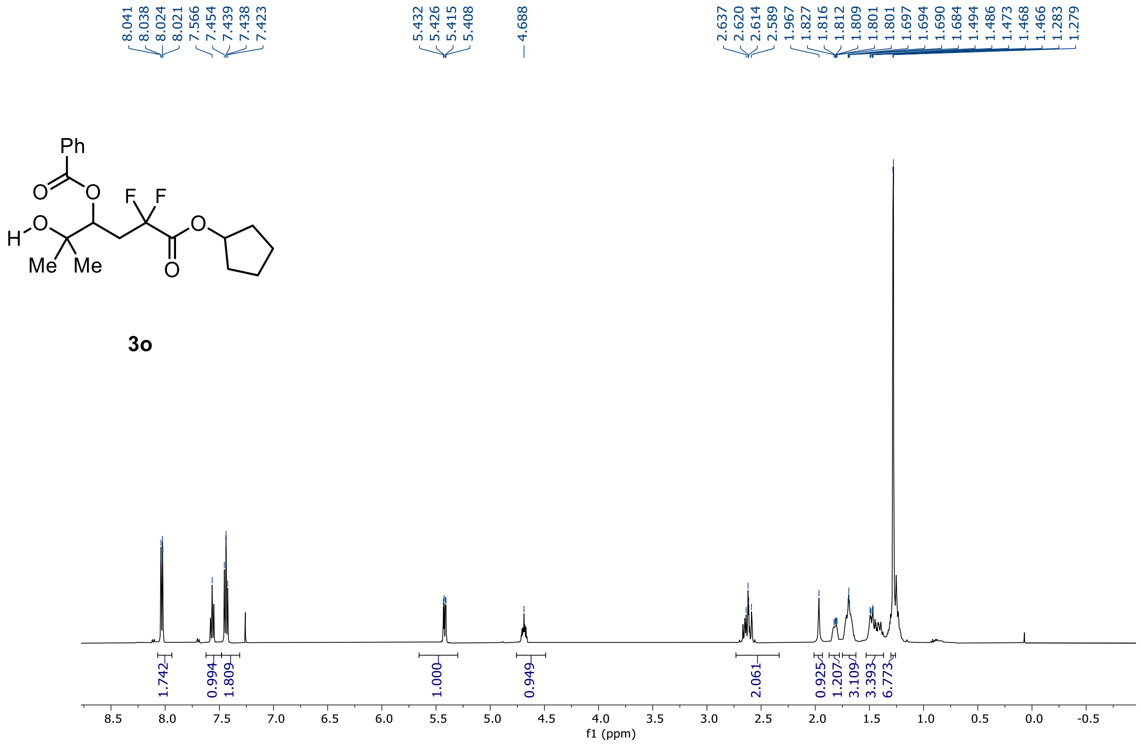

^{13}C NMR (126 MHz, CDCl$_3$**, 25 ºC) of (3o)**

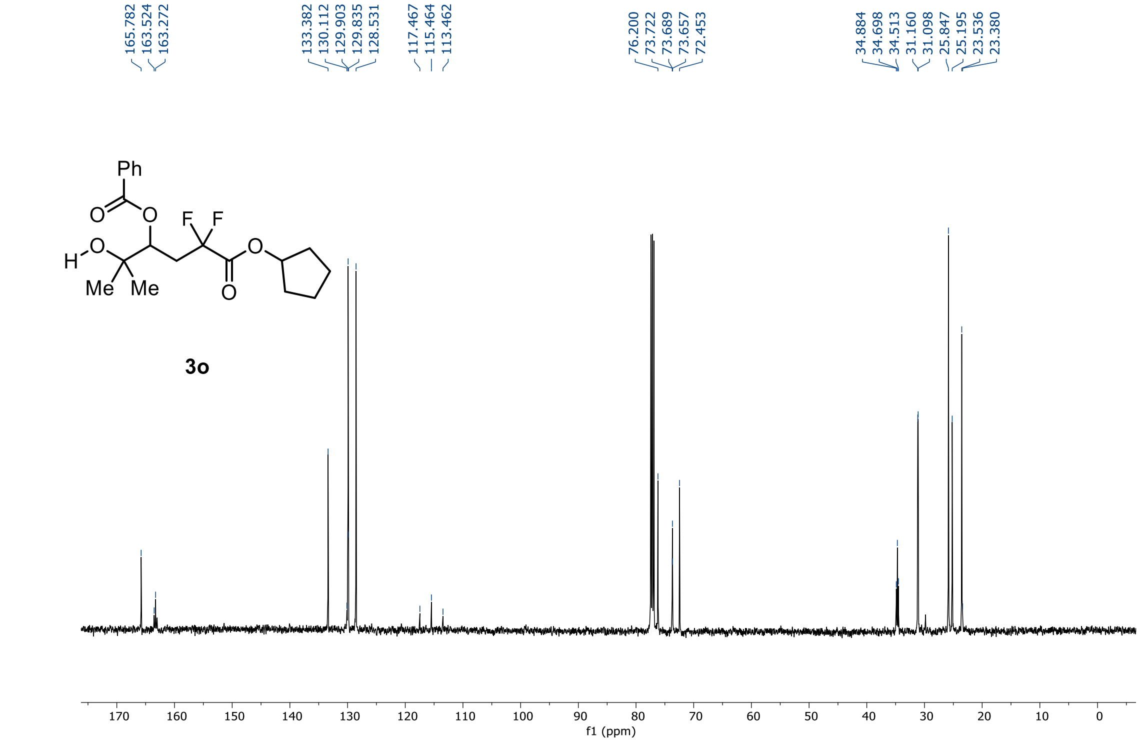

^{19}F NMR (470 MHz, $CDCl_3$**, 25 ºC) of (3o)**

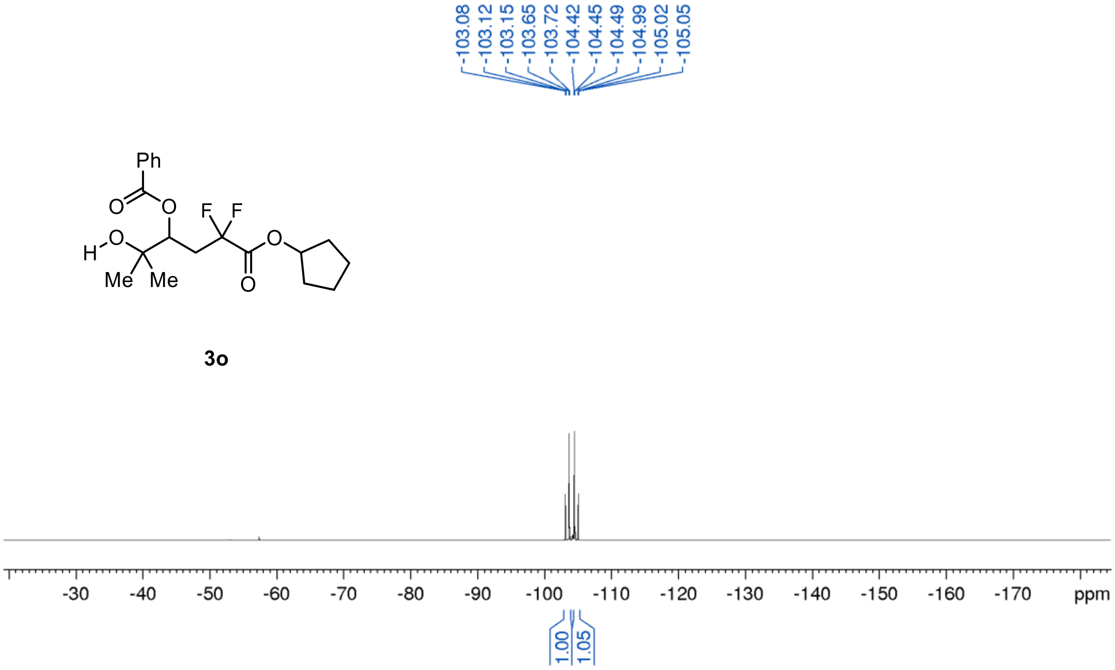

^{1}H NMR (500 MHz, CDCl$_3$**, 25 ºC) of (3p)**

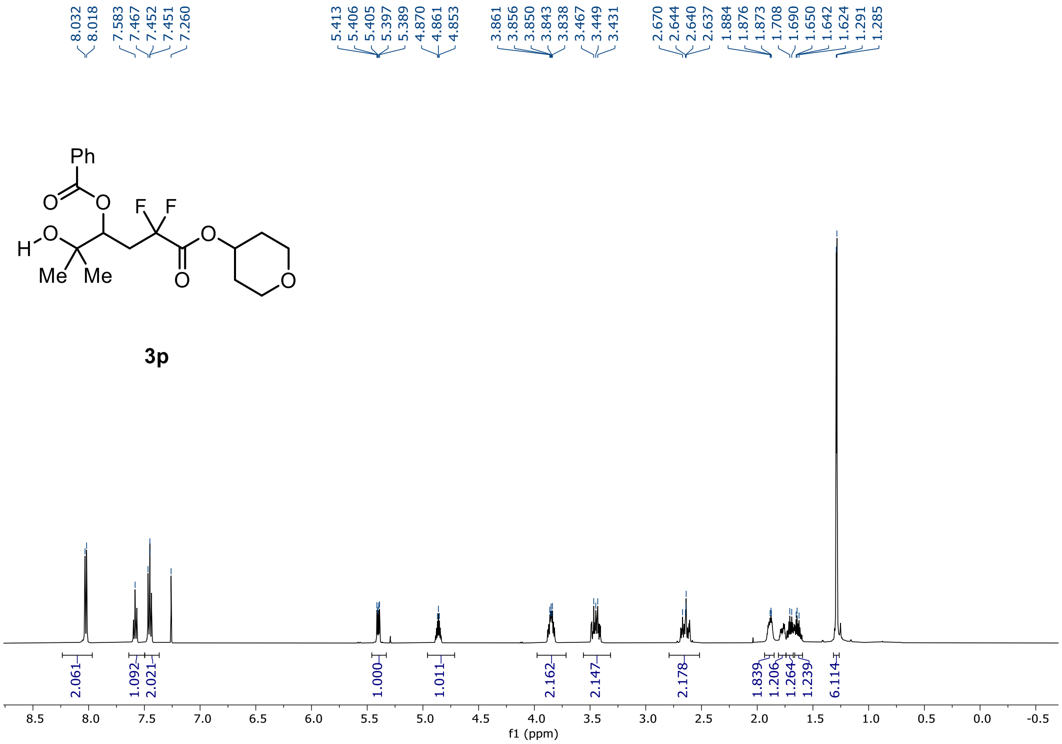

^{13}C NMR (126 MHz, CDCl$_3$**, 25 ºC) of (3p)**

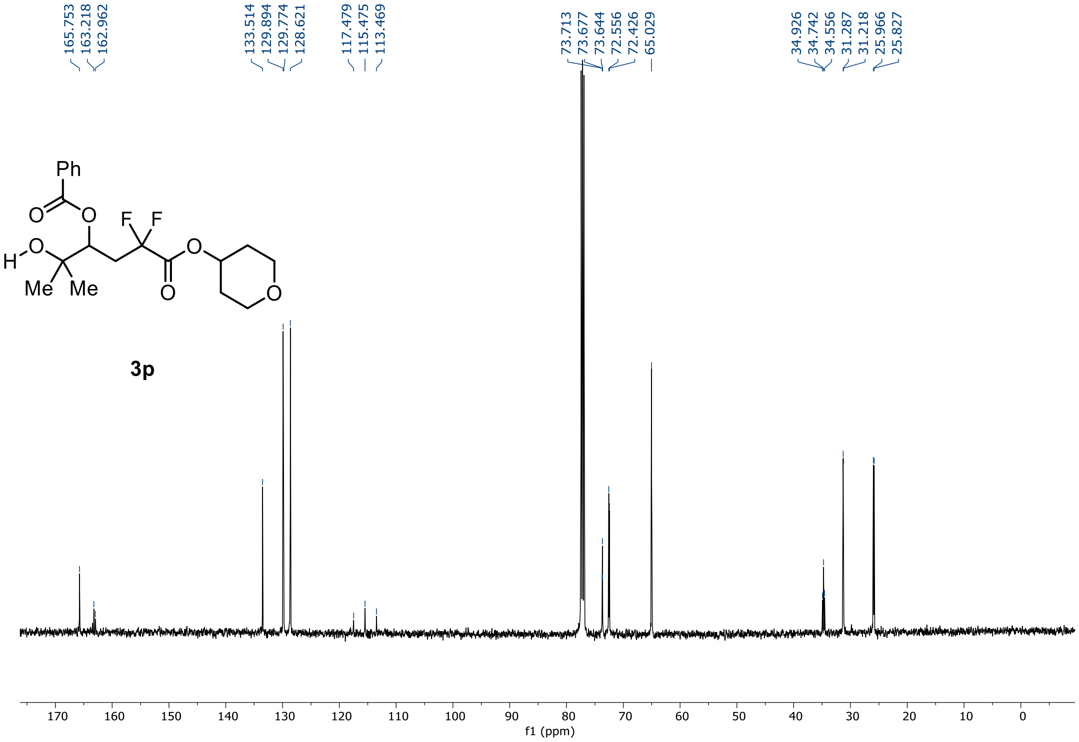

^{19}F NMR (470 MHz, CDCl$_3$**, 25 ºC) of (3p)**

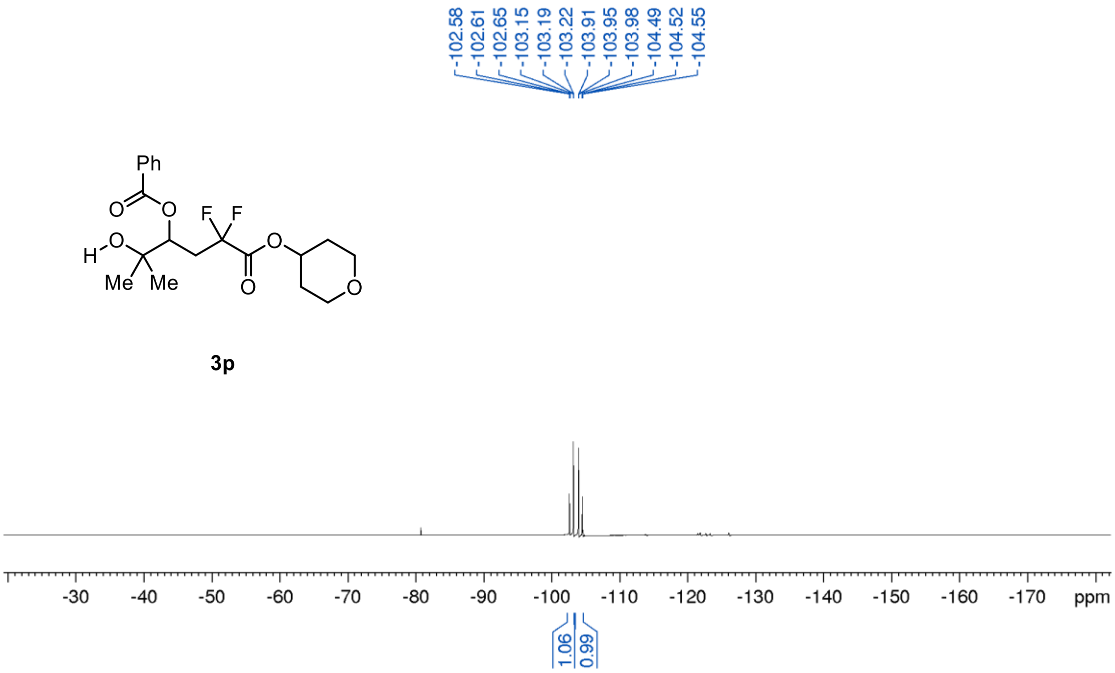

^{1}H NMR (500 MHz, $CDCl_3$, 25 ºC) of (3q)

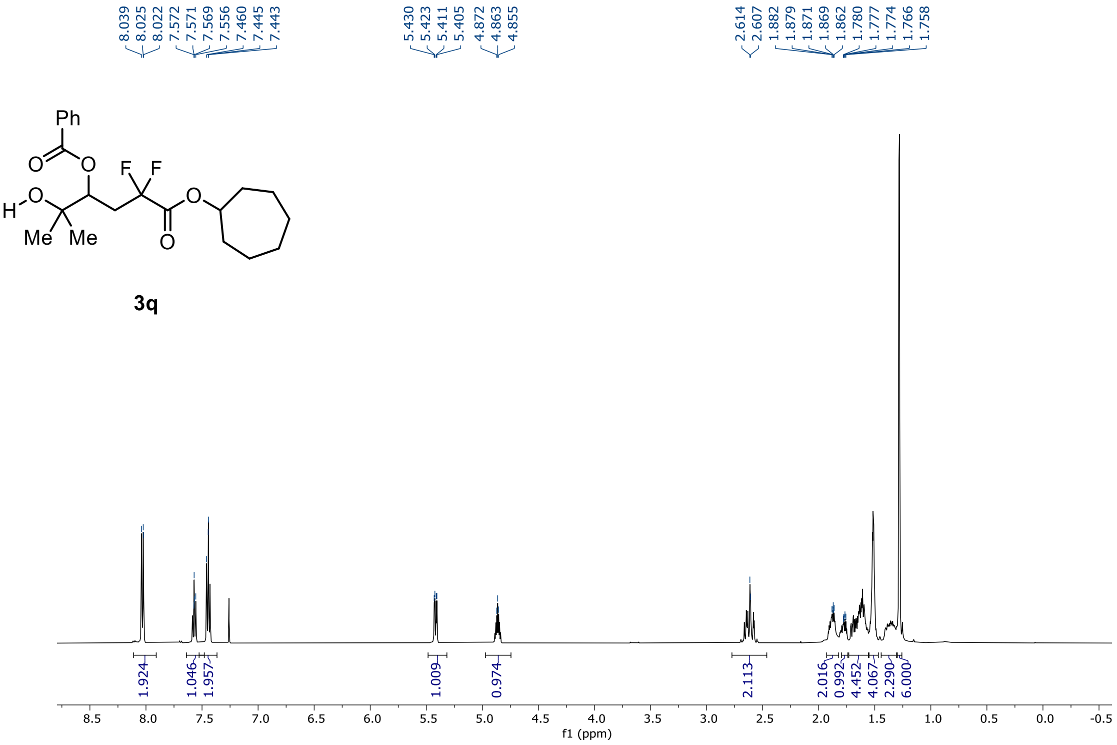

^{13}C NMR (125 MHz, $CDCl_3$, 25 ºC) of (3q)

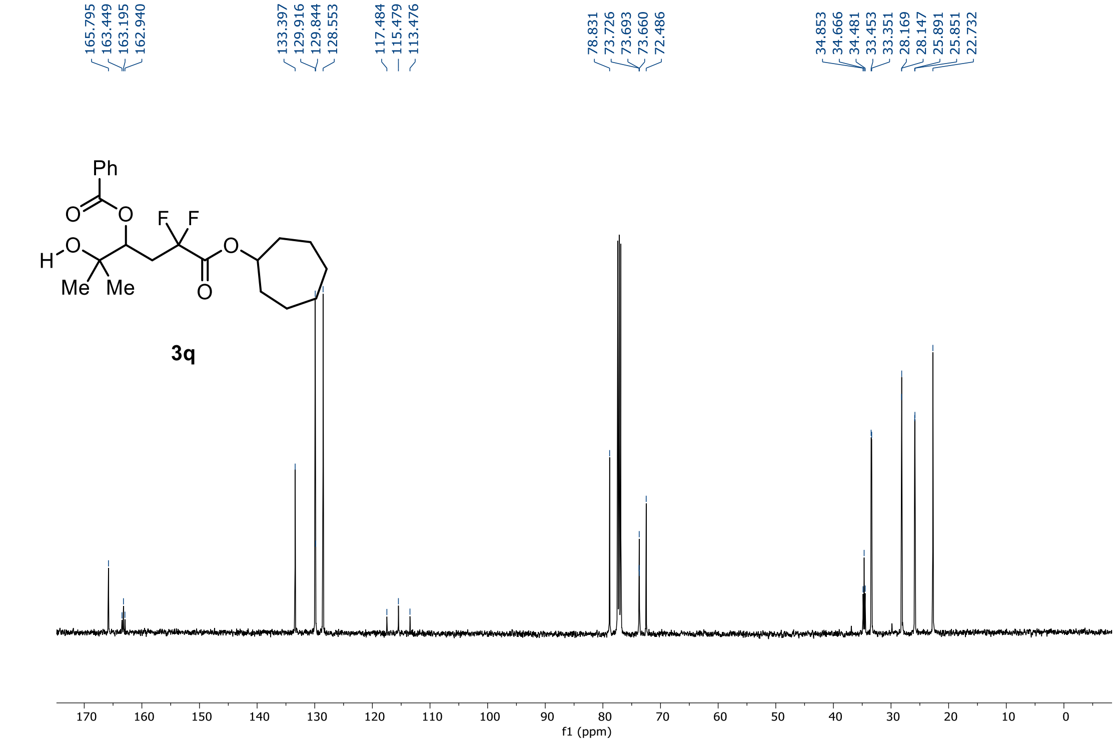

^{19}F NMR (470 MHz, CDCl$_3$**, 25 ºC) of (3q)**

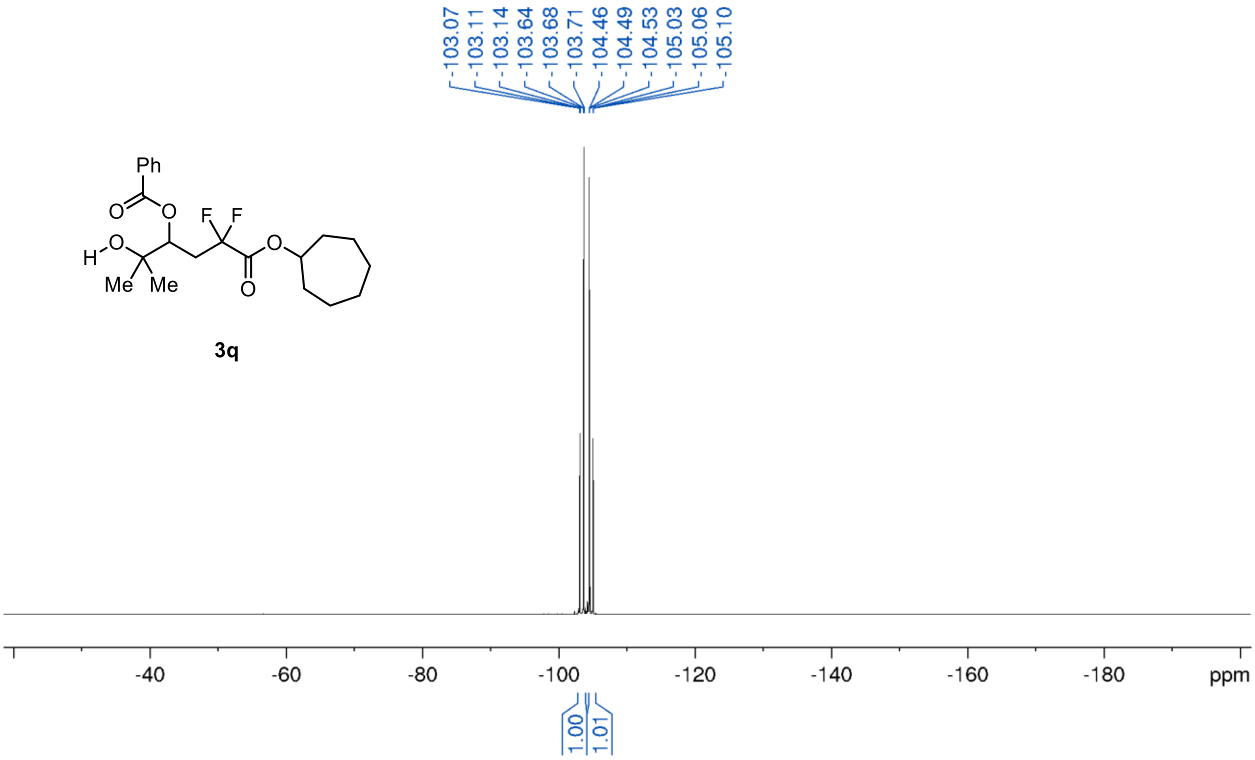

^{1}H NMR (500 MHz, $CDCl_3$, 25 ºC) of (3r)

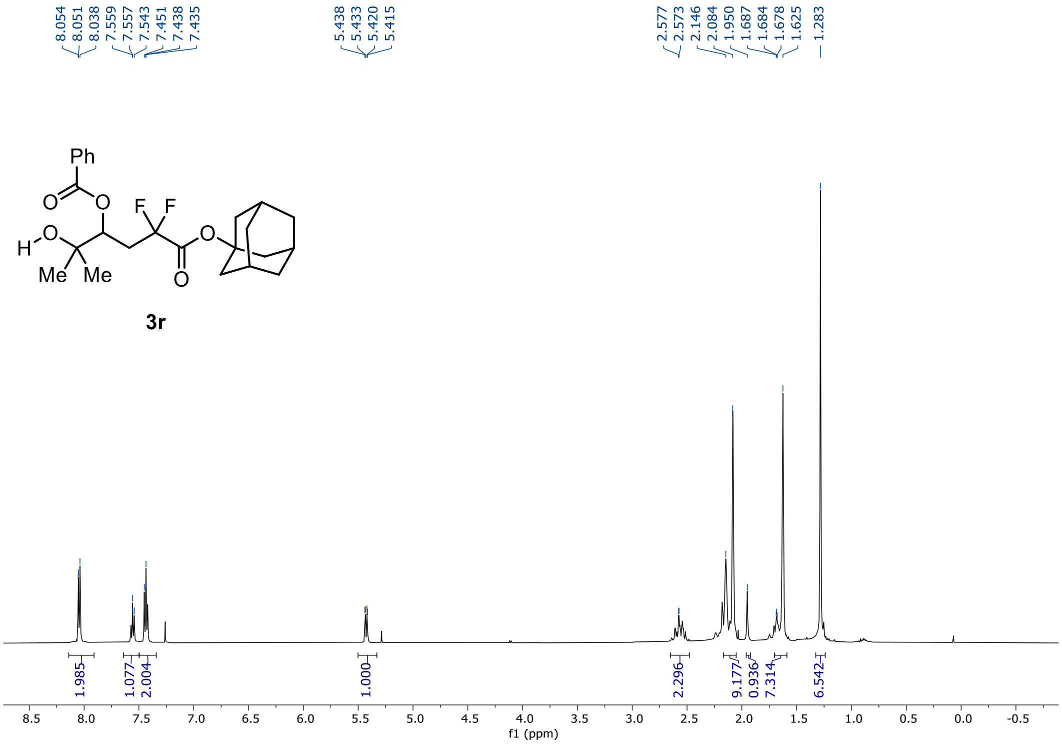

^{13}C NMR (126 MHz, $CDCl_3$, 25 ºC) of (3r)

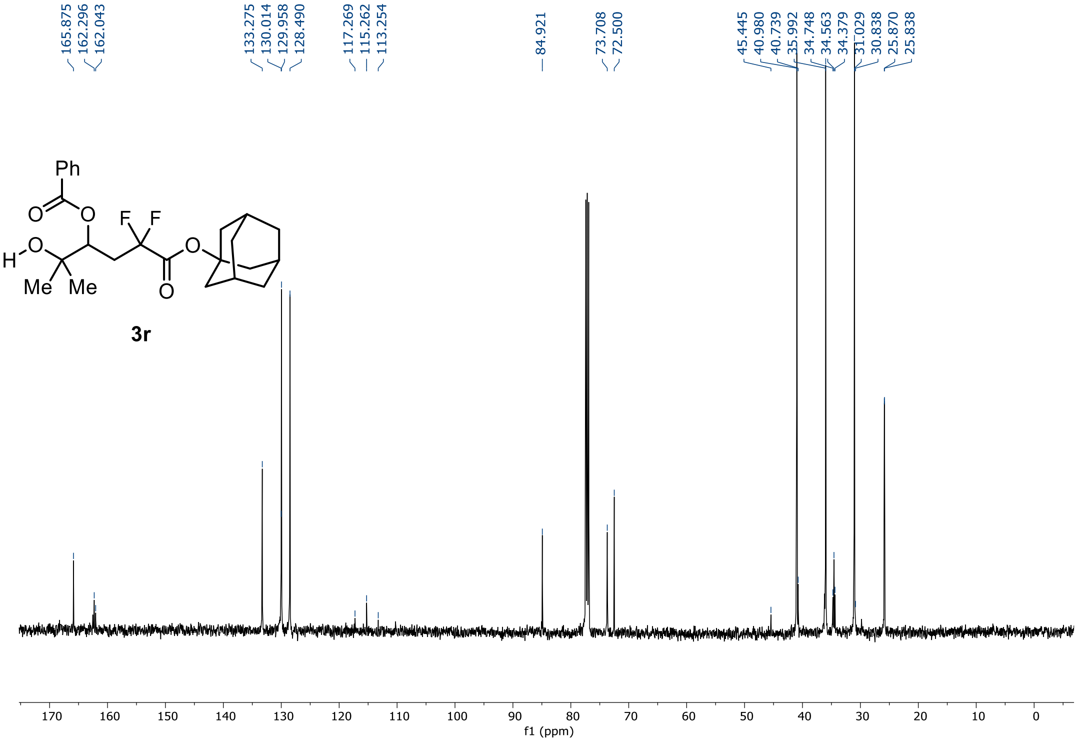

^{19}F NMR (470 MHz, CDCl$_3$**, 25 ºC) of (3r)**

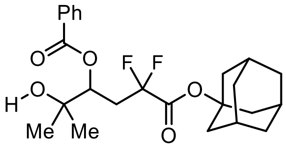

3r

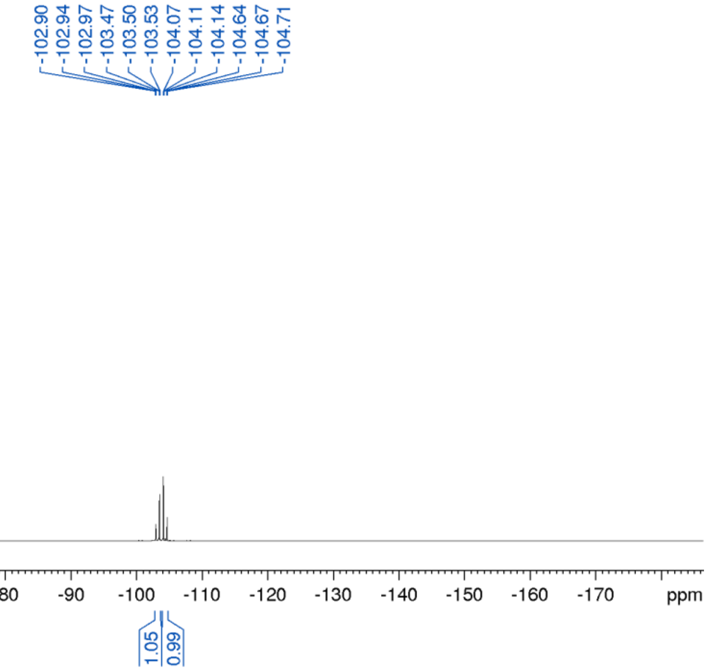

^{1}H NMR (500 MHz, $CDCl_3$**, 25 ºC) of (3s)**

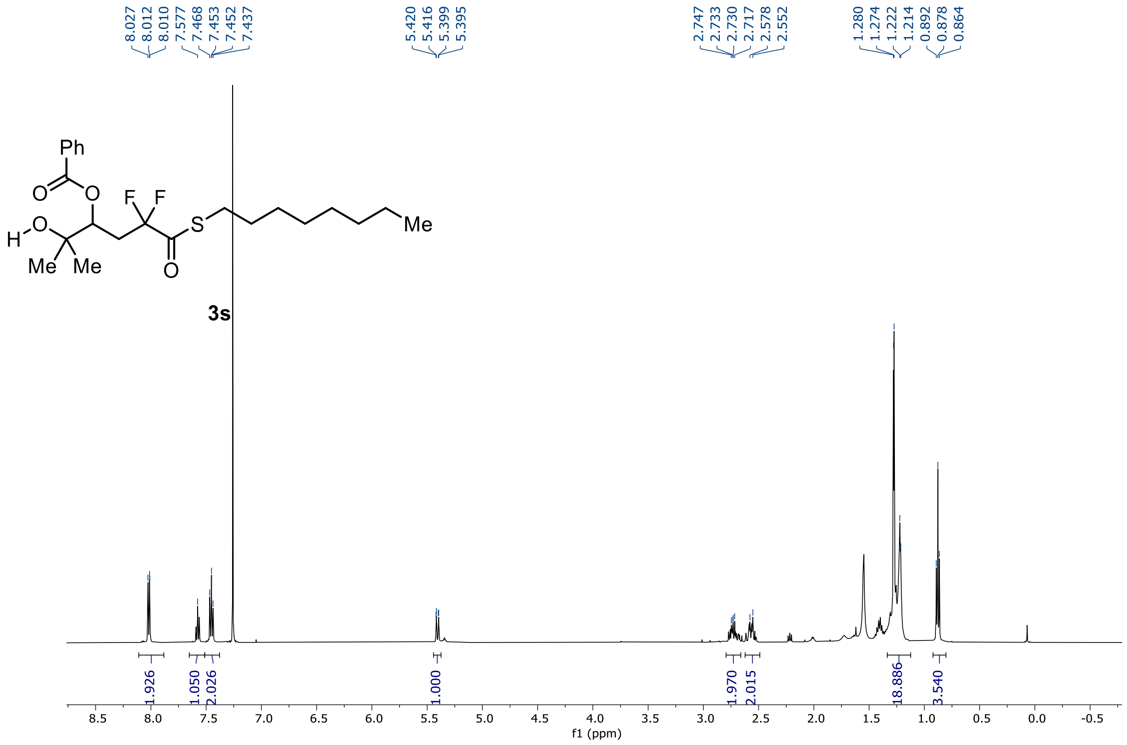

^{13}C NMR (126 MHz, $CDCl_3$**, 25 ºC) of (3s)**

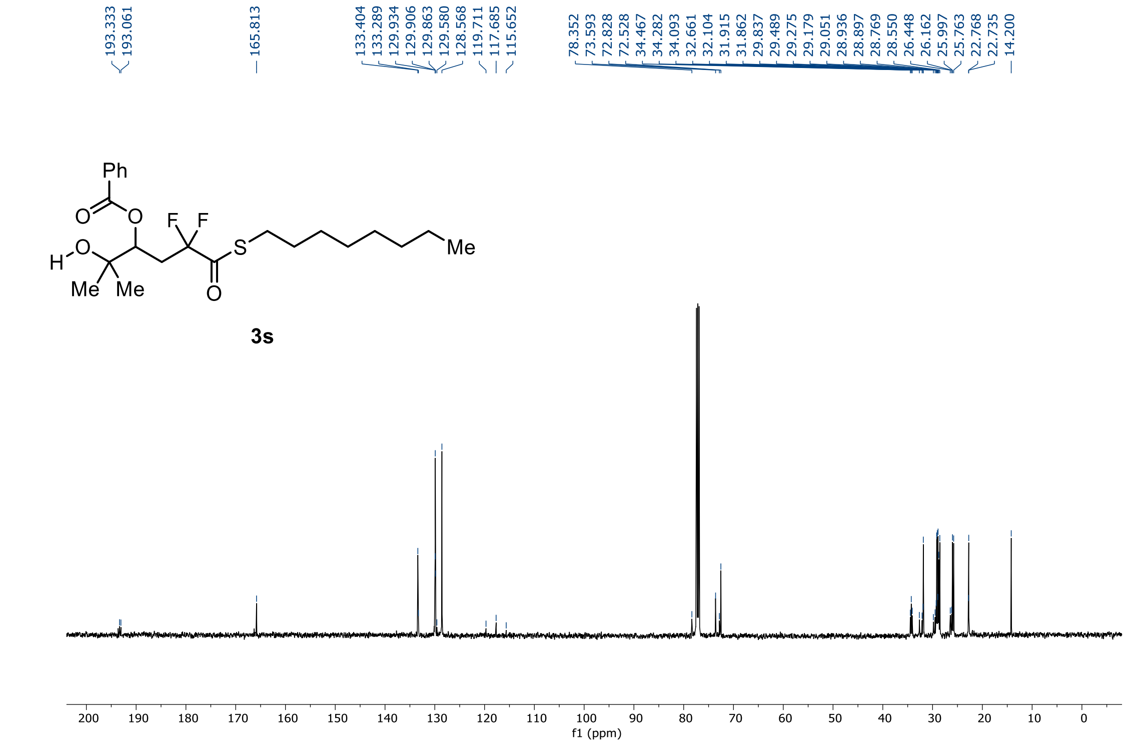

^{19}F NMR (376 MHz, CDCl$_3$**, 25 ºC) of (3s)**

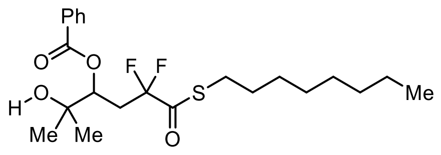

3s

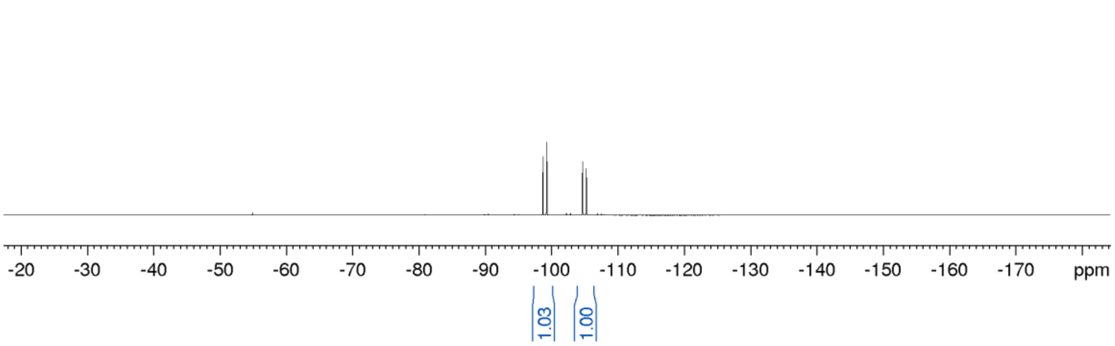

^{1}H NMR (500 MHz, CDCl3, 25 ºC) of (3t)

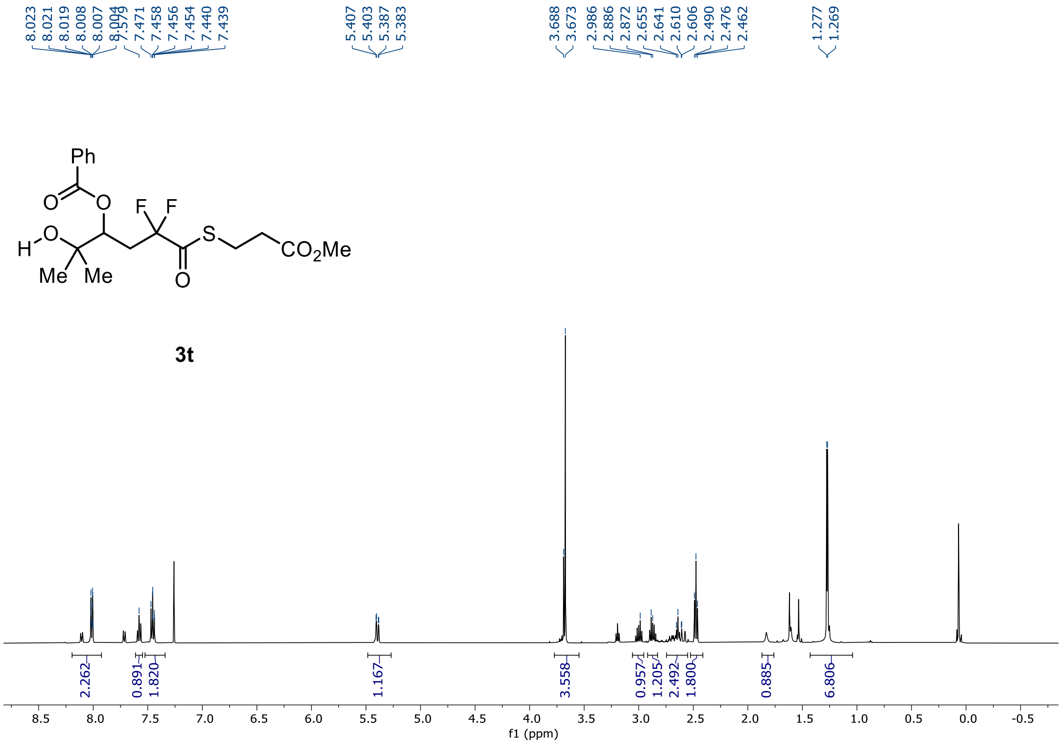

^{13}C NMR (126 MHz, CDCl3, 25 ºC) of (3t)

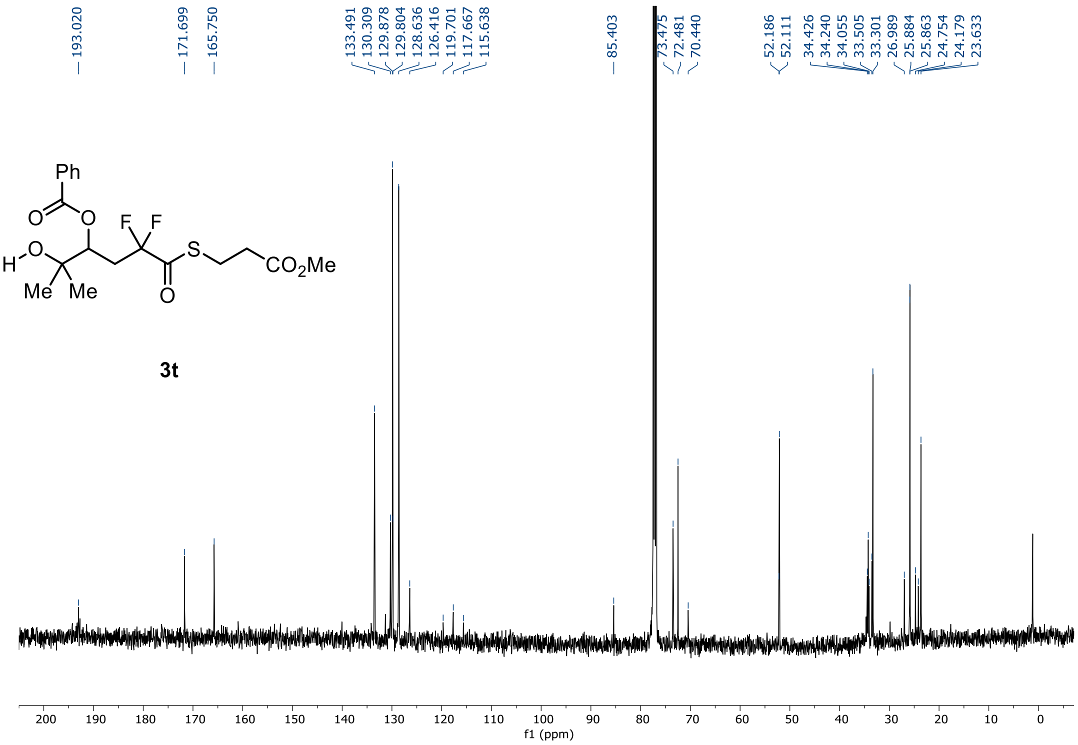

^{19}F NMR (470 MHz, $CDCl_3$**, 25 ºC) of (3t)**

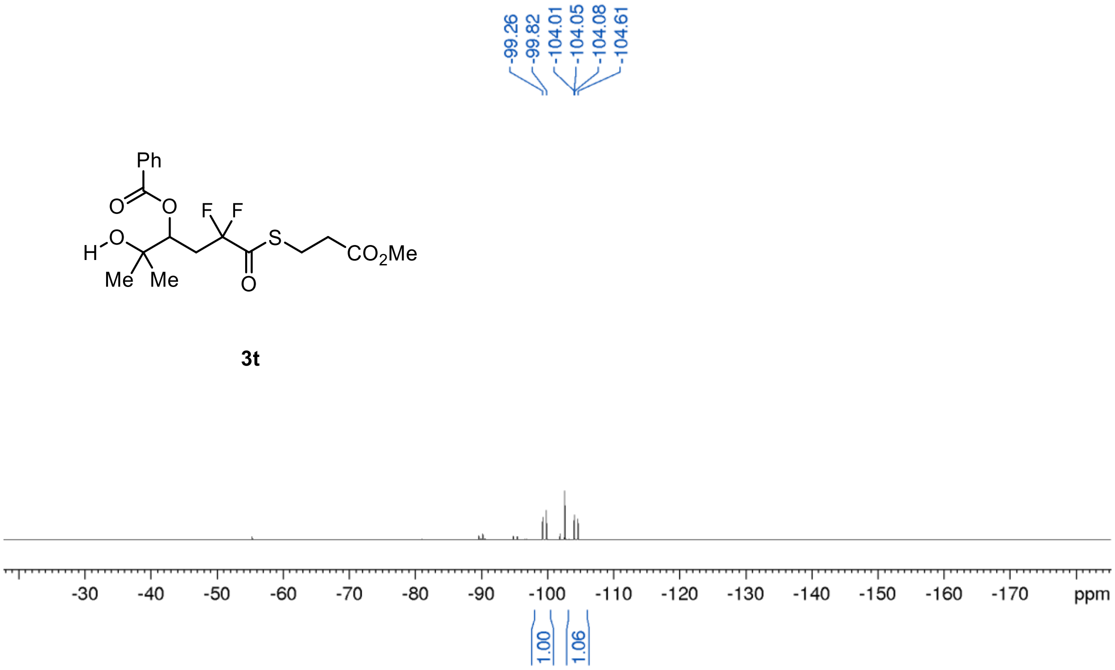

3t

^{1}H NMR (500 MHz, CDCl$_3$, 25 ºC) of (3u)

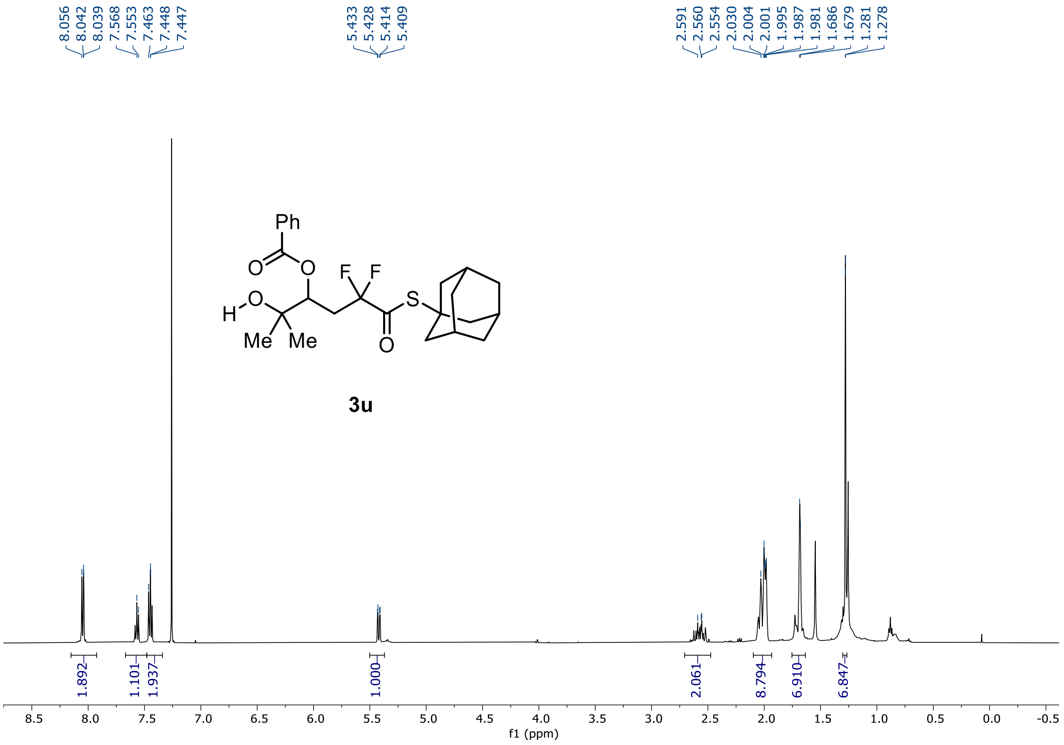

^{13}C NMR (126 MHz, CDCl$_3$, 25 ºC) of (3u)

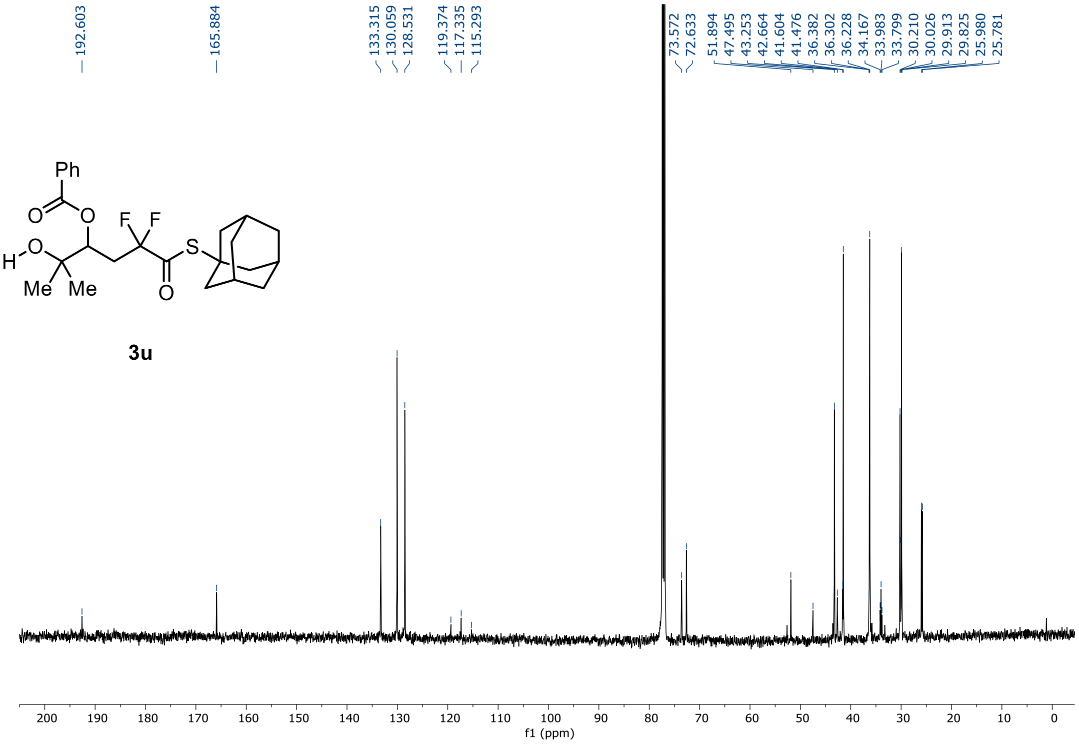

^{19}F NMR (470 MHz, CDCl3**, 25 ºC) of (3u)**

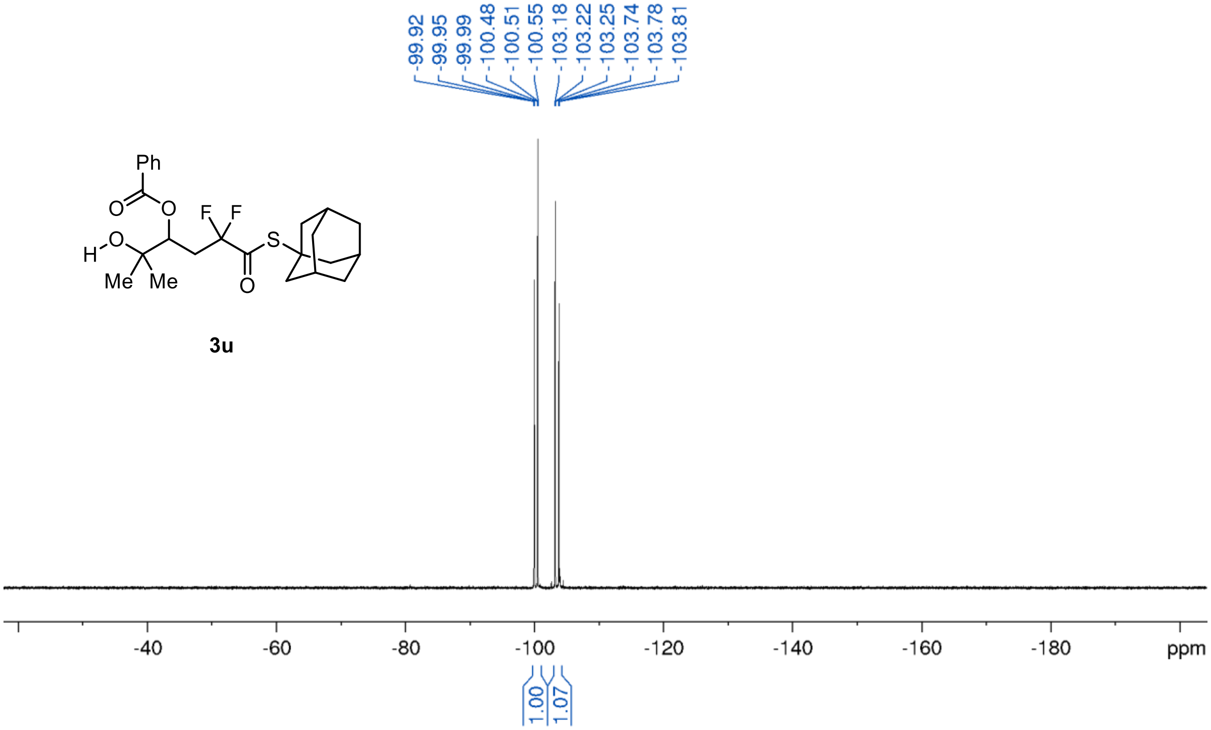

^{1}H NMR (500 MHz, $CDCl_3$, 25 ºC) of (3v)

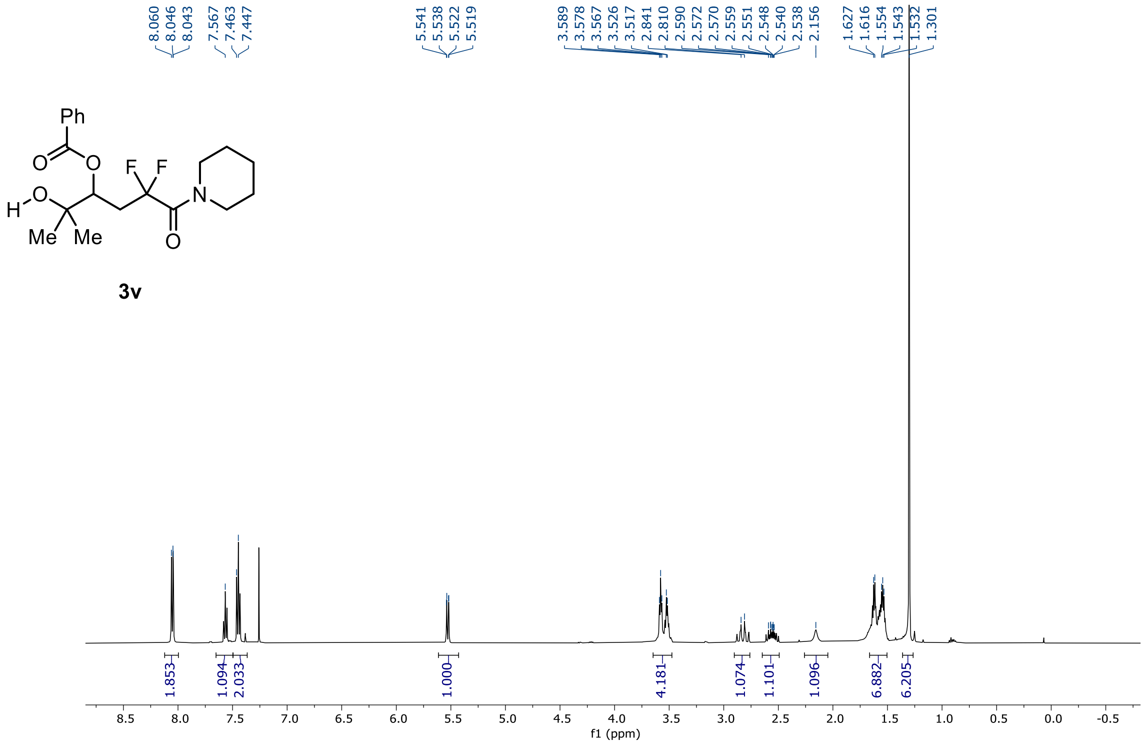

^{13}C NMR (126 MHz, $CDCl_3$, 25 ºC) of (3v)

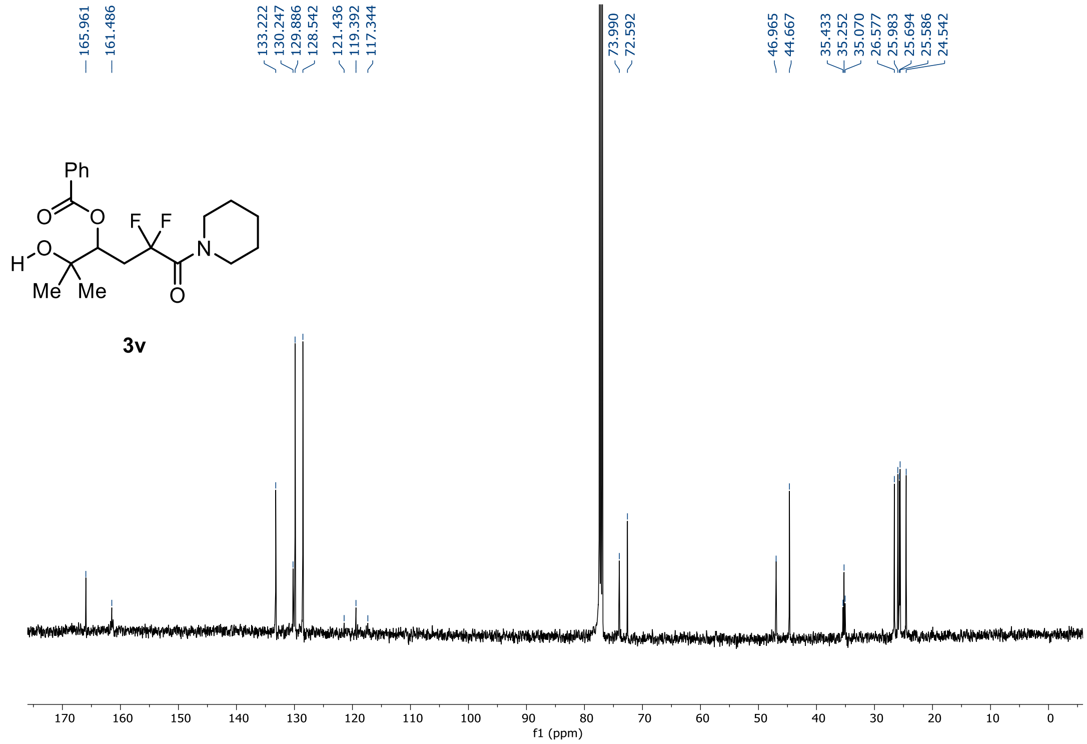

^{19}F NMR (470 MHz, CDCl$_3$**, 25 ºC) of (3v)**

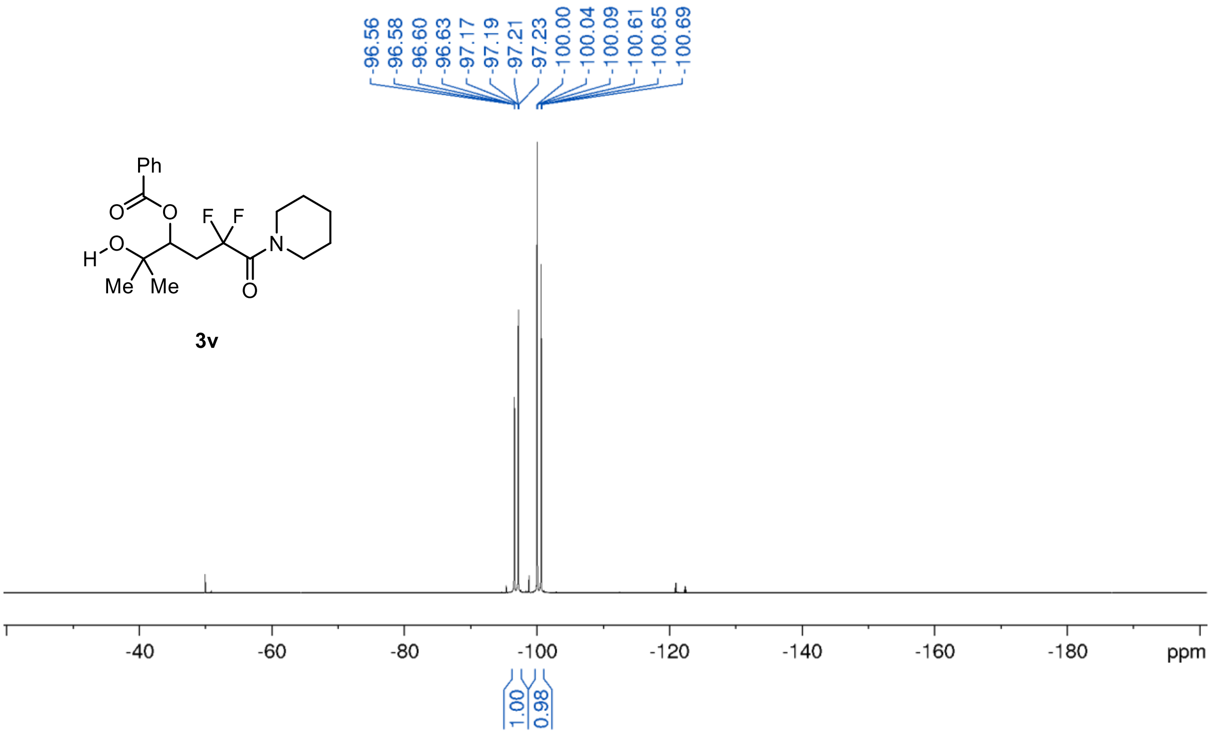

^{1}H NMR (500 MHz, $CDCl_3$, 25 ºC) of (3w)

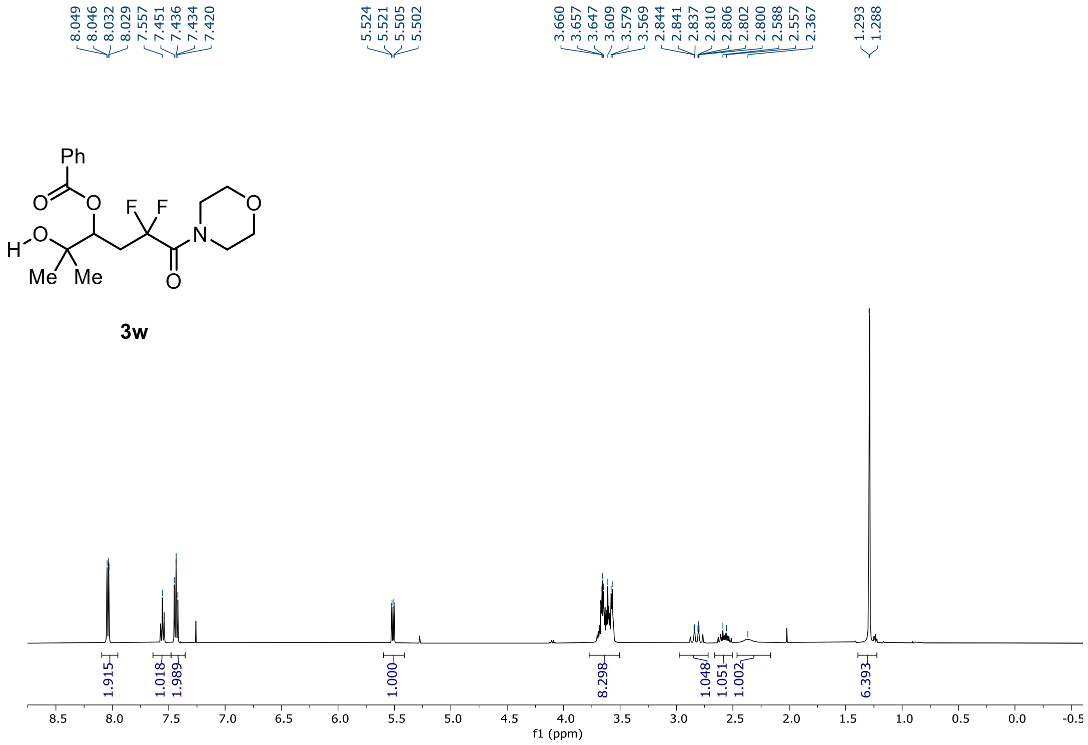

^{13}C NMR (126 MHz, $CDCl_3$, 25 ºC) of (3w)

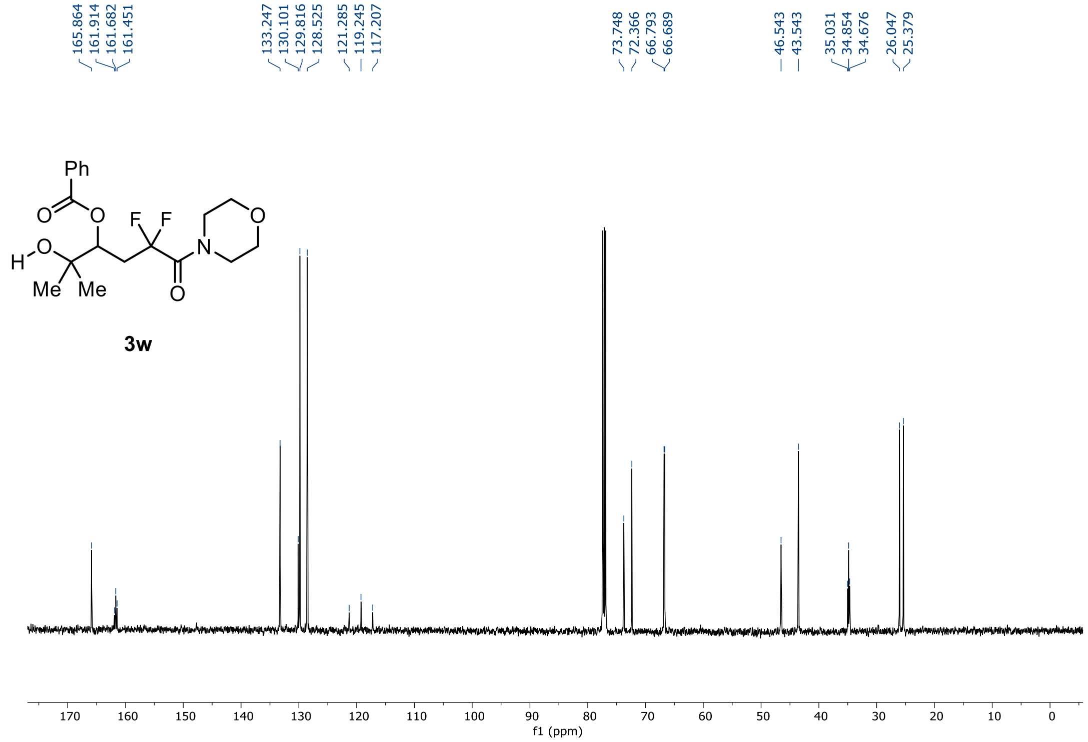

^{19}F NMR (470 MHz, CDCl$_3$**, 25 ºC) of (3w)**

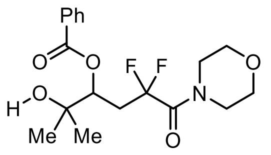

3w

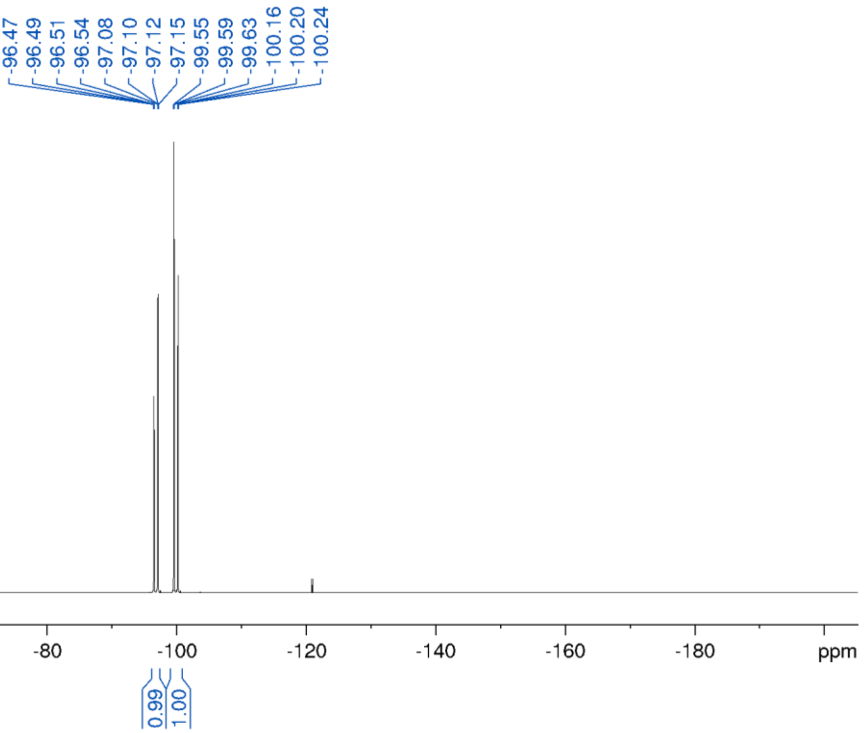

^{1}H NMR (500 MHz, $CDCl_3$, 25 ºC) of (3x)

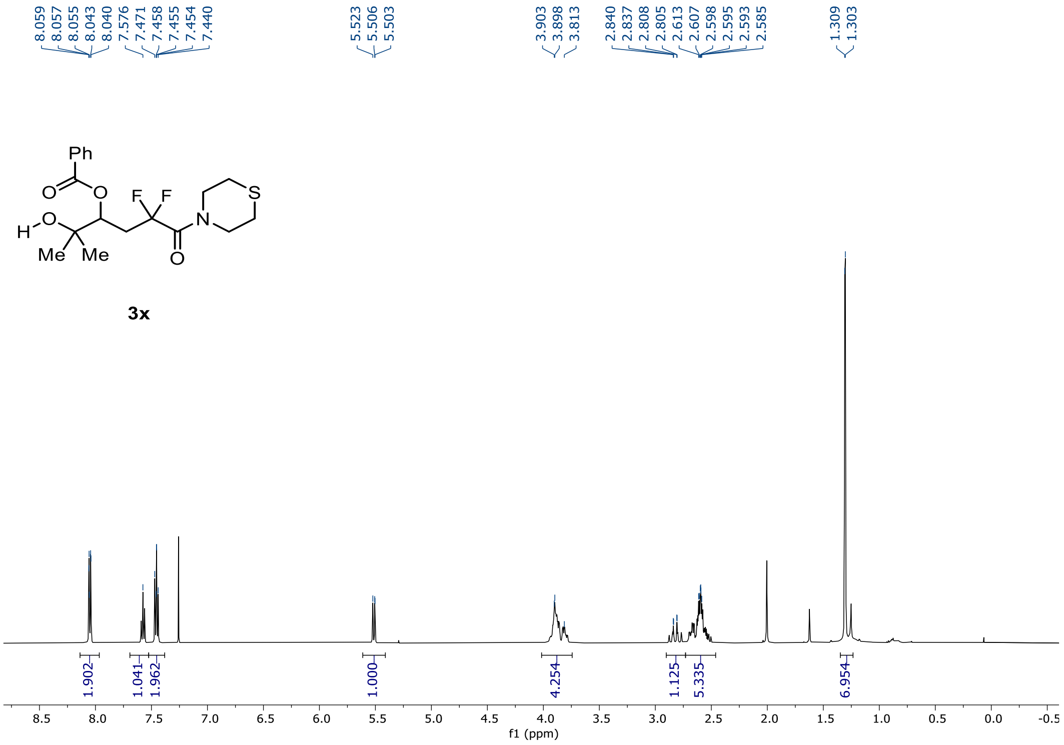

^{13}C NMR (126 MHz, $CDCl_3$, 25 ºC) of (3x)

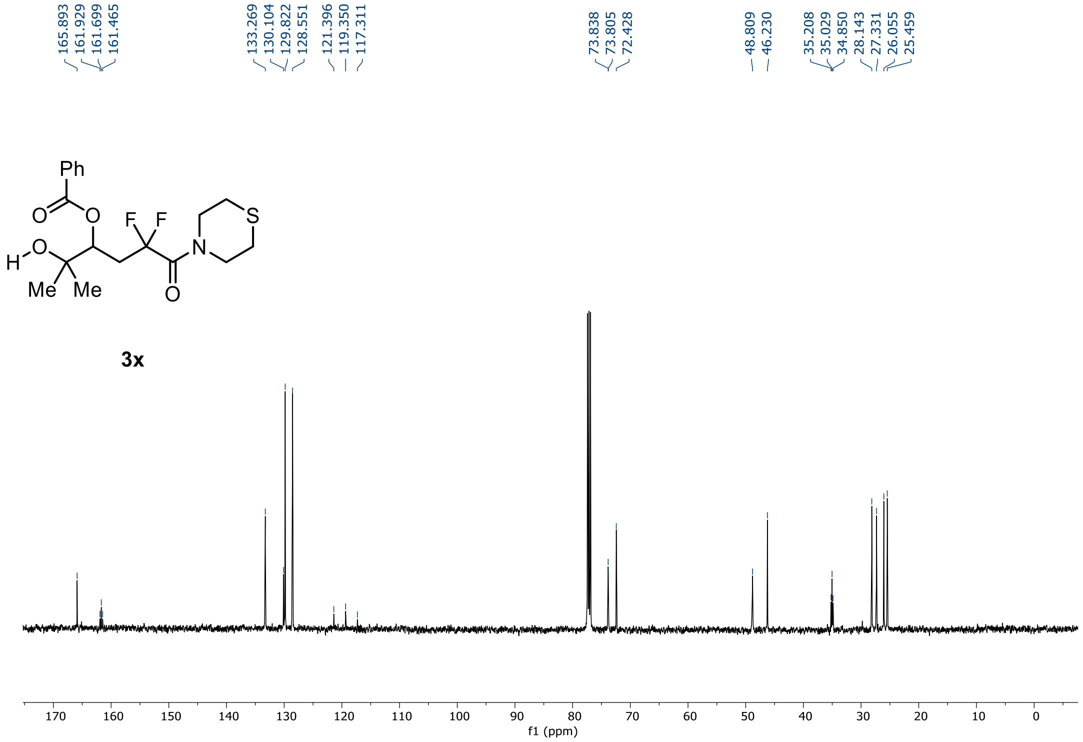

^{19}F NMR (376 MHz, CDCl$_3$**, 25 °C) of (3x)**

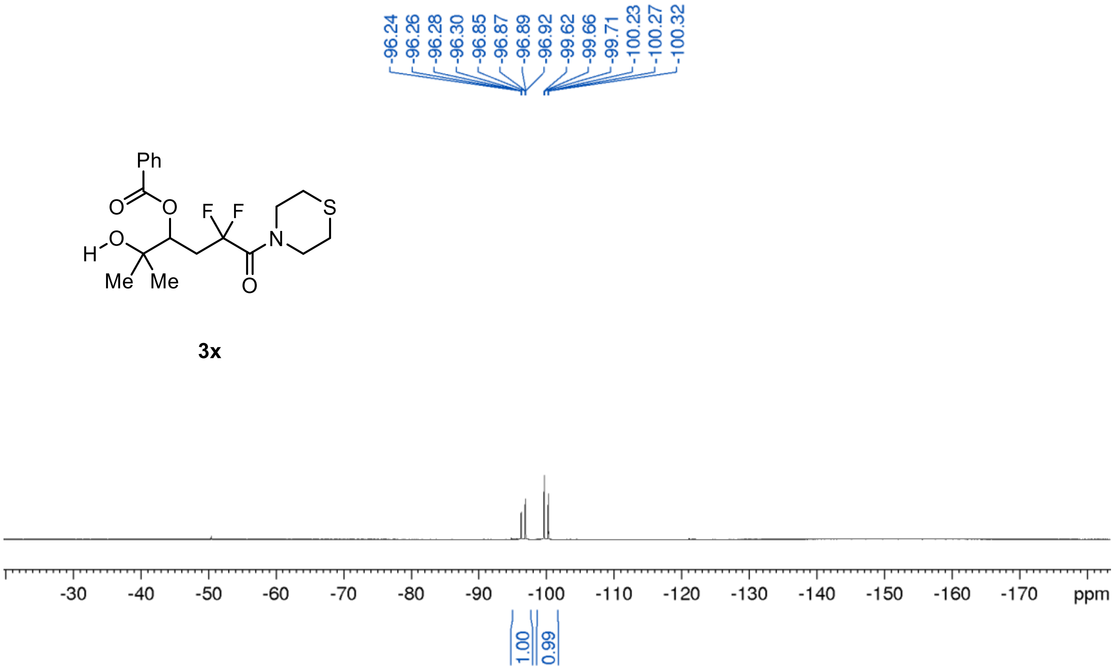

^{1}H NMR (500 MHz, $CDCl_3$, 25 ºC) of (3y)

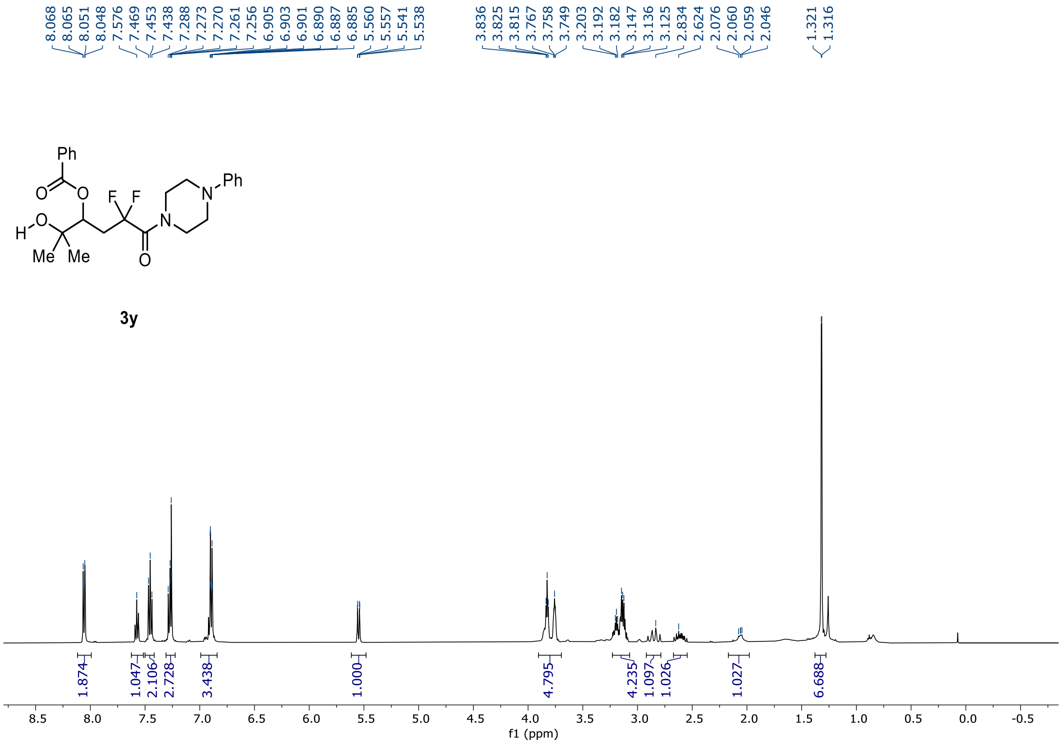

^{13}C NMR (100 MHz, $CDCl_3$, 25 ºC) of (3y)

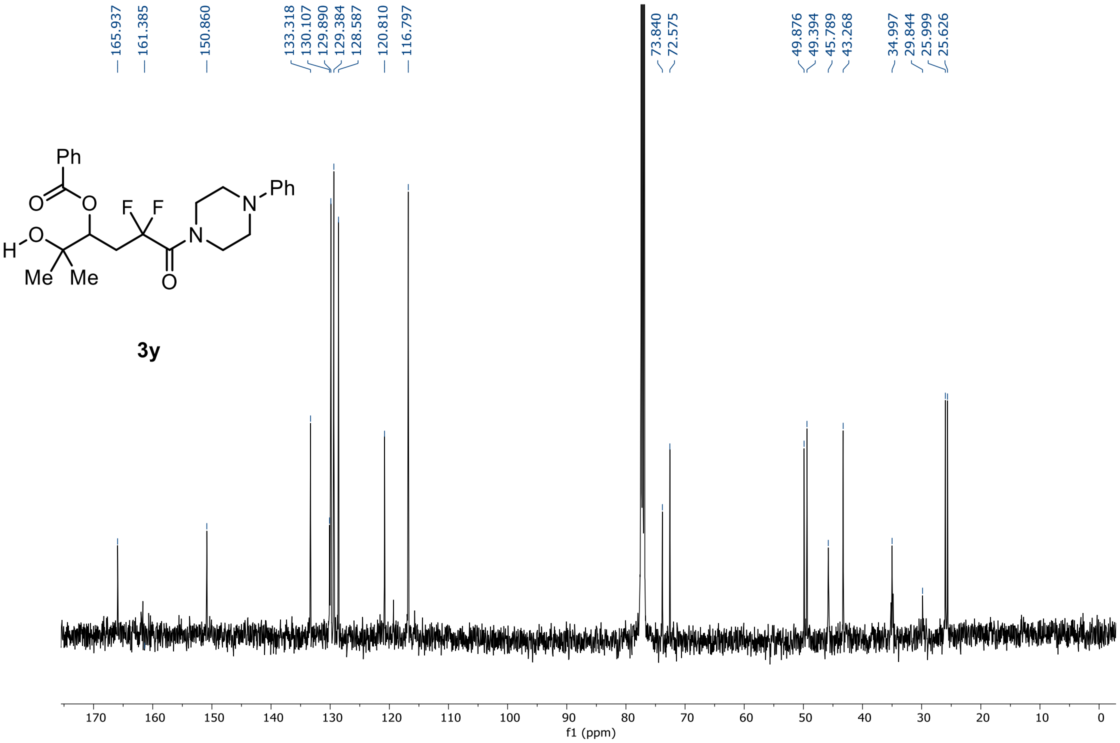

^{19}F NMR (376 MHz, $CDCl_3$**, 25 ºC) of (3y)**

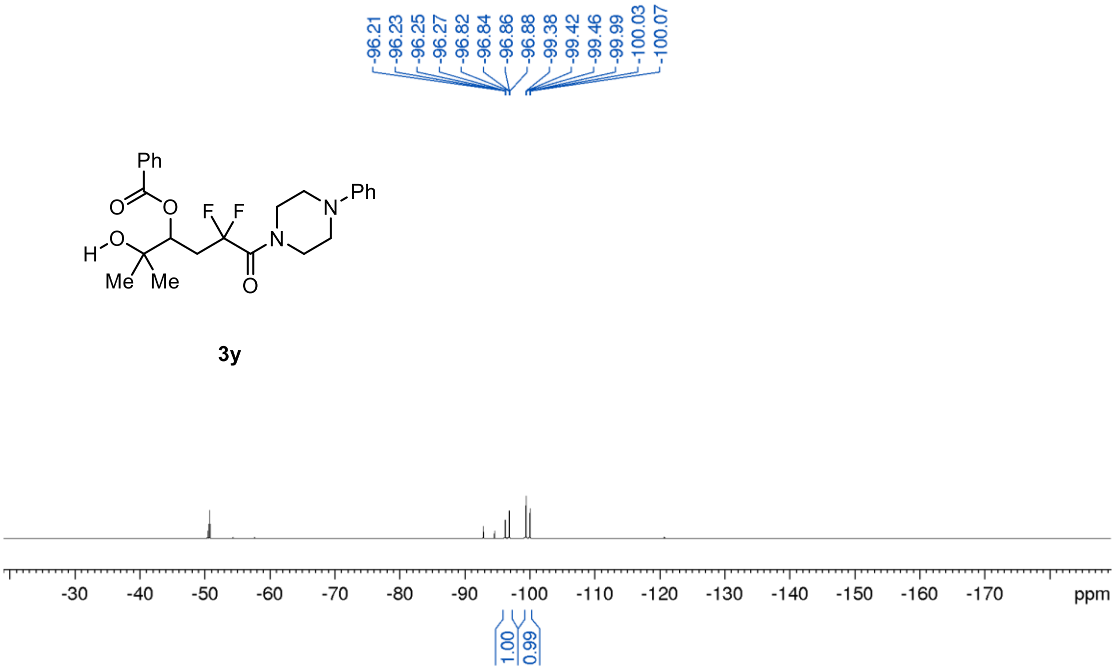

^{1}H NMR (500 MHz, CDCl3, 25 ºC) of (3z)

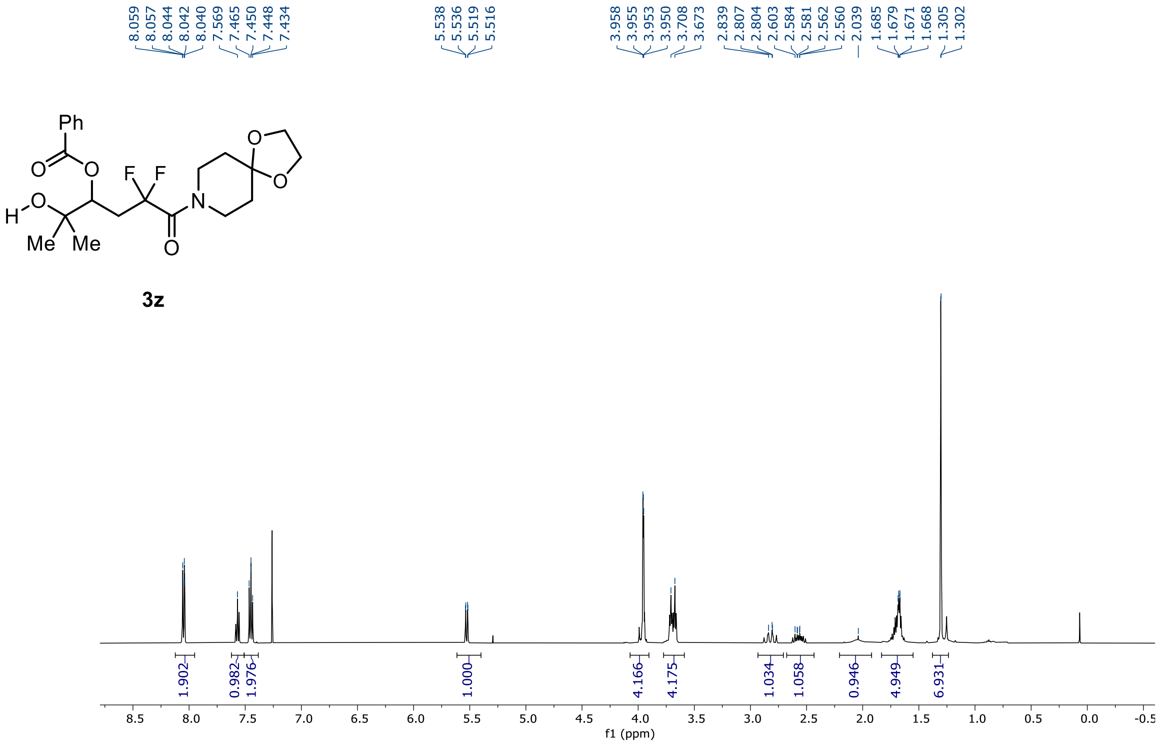

^{13}C NMR (126 MHz, CDCl3, 25 ºC) of (3z)

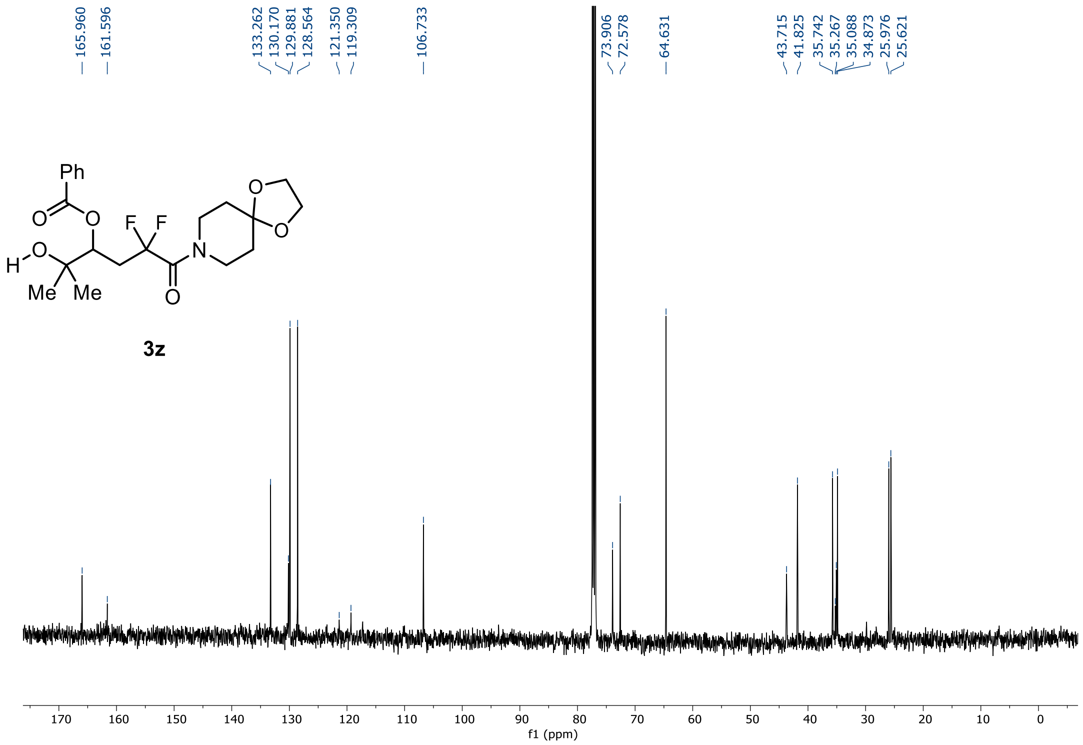

^{19}F NMR (470 MHz, $CDCl_3$**, 25 ºC) of (3z)**

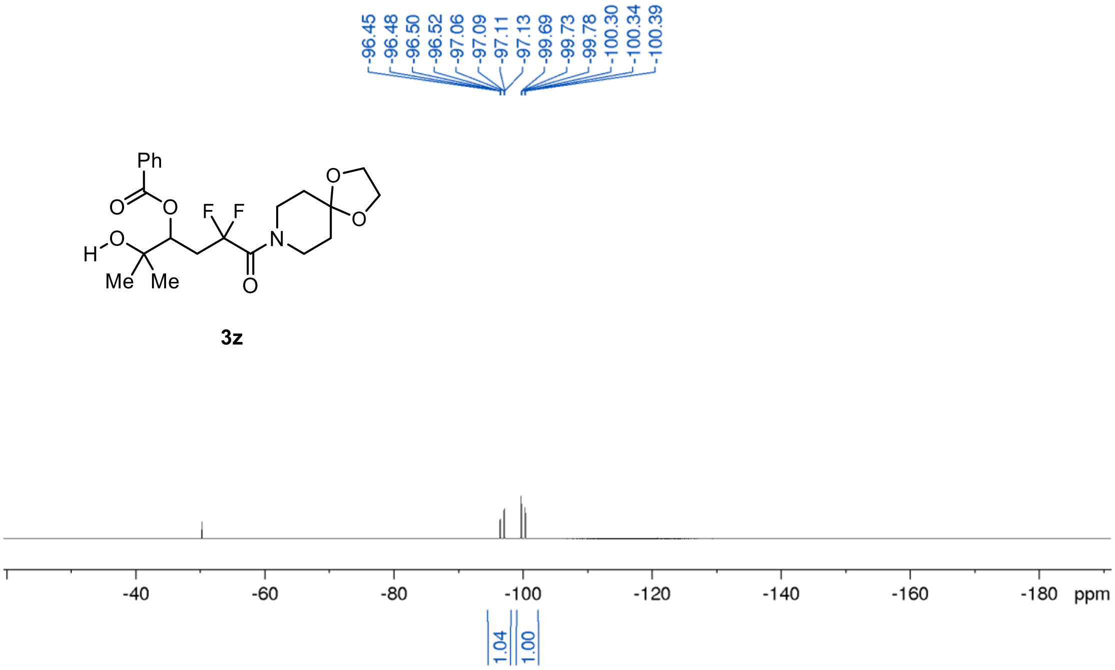

^{1}H NMR (500 MHz, CDCl$_3$, 25 ºC) of (3aa)

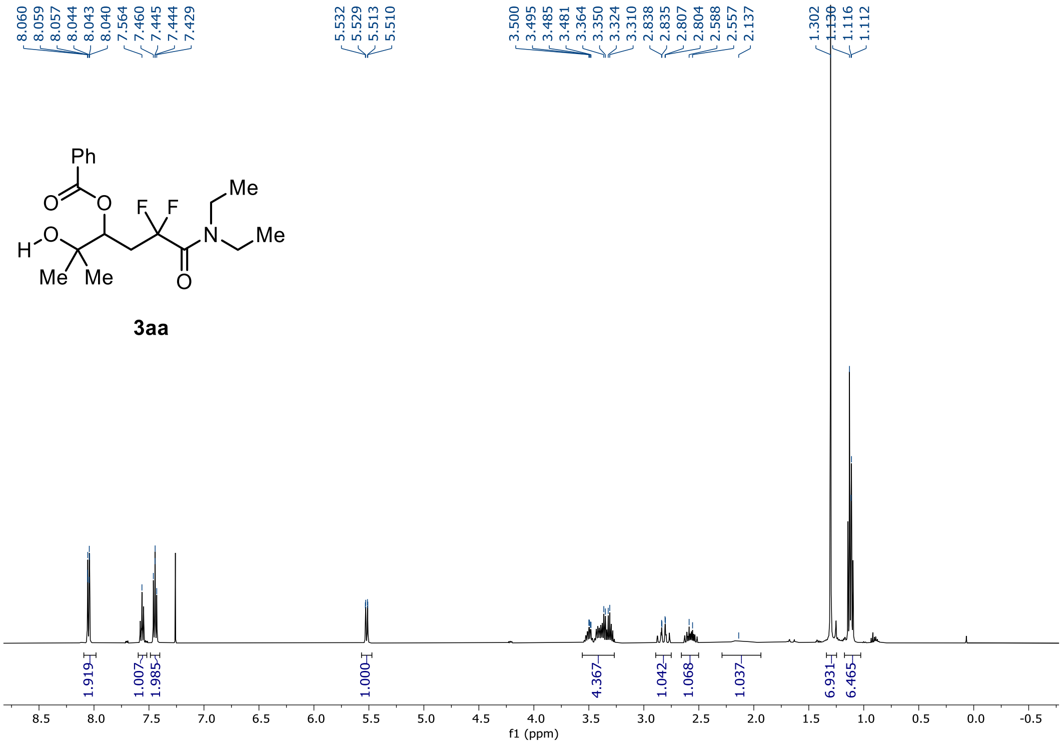

^{13}C NMR (126 MHz, CDCl$_3$, 25 ºC) of (3aa)

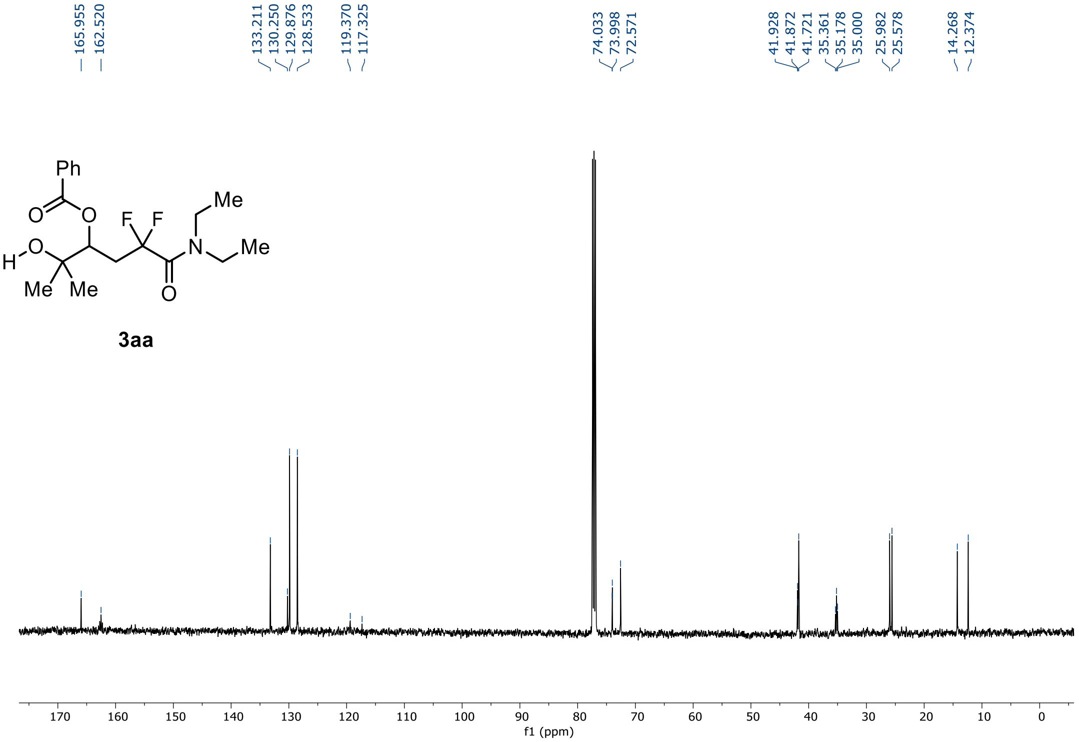

^{19}F NMR (470 MHz, $CDCl_3$**, 25 ºC) of (3aa)**

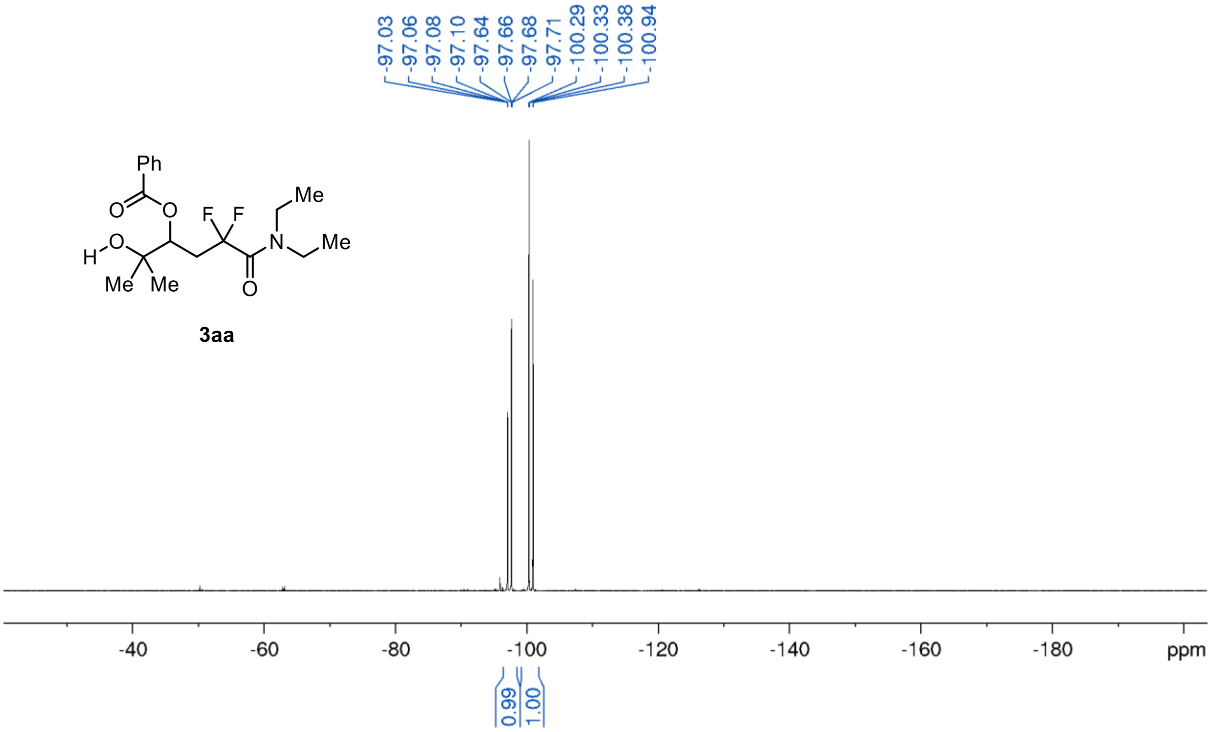

^{1}H NMR (500 MHz, $CDCl_3$, 25 ºC) of (3ab)

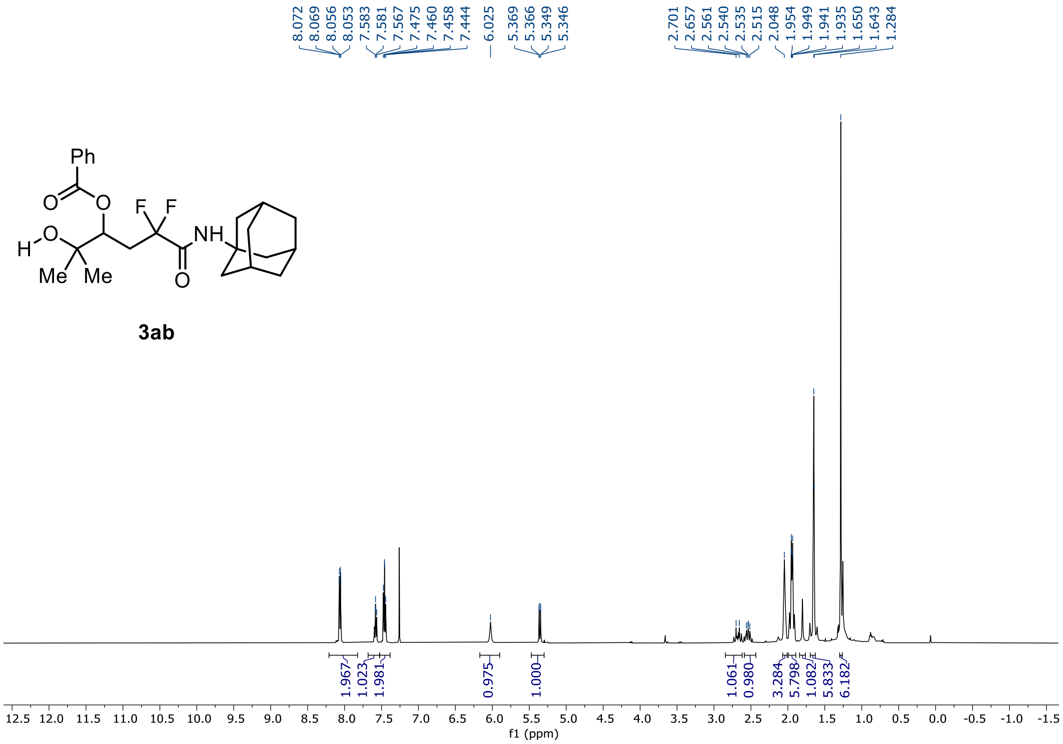

^{13}C NMR (126 MHz, $CDCl_3$, 25 ºC) of (3ab)

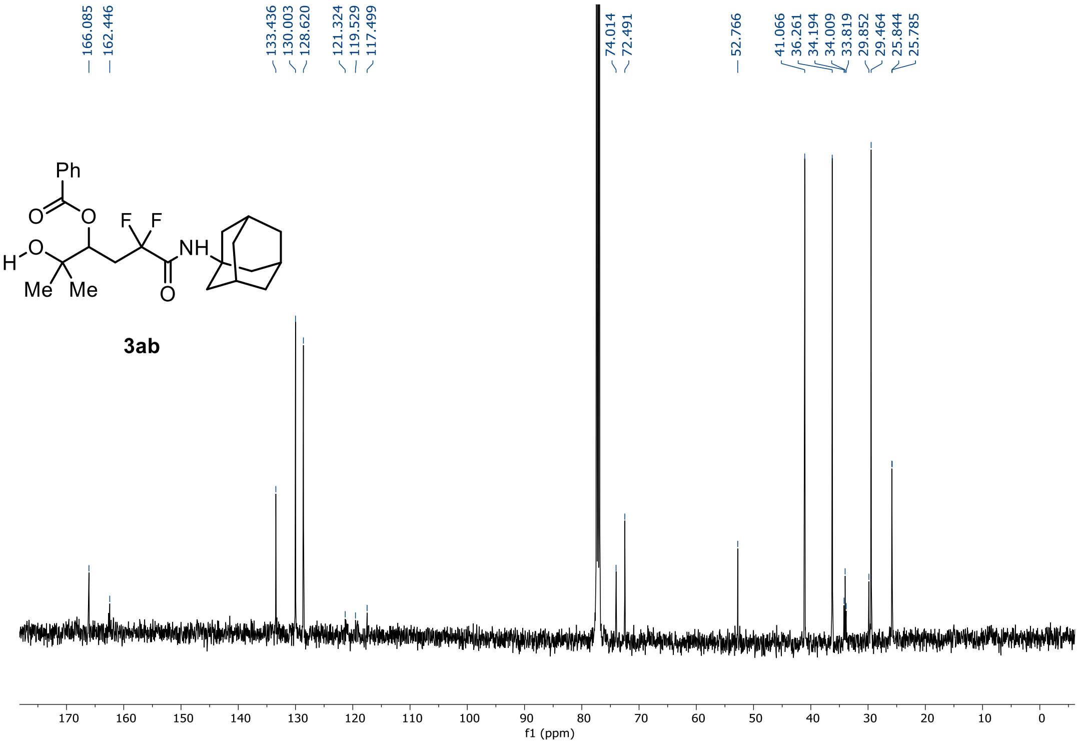

^{19}F NMR (470 MHz, CDCl$_3$**, 25 ºC) of (3ab)**

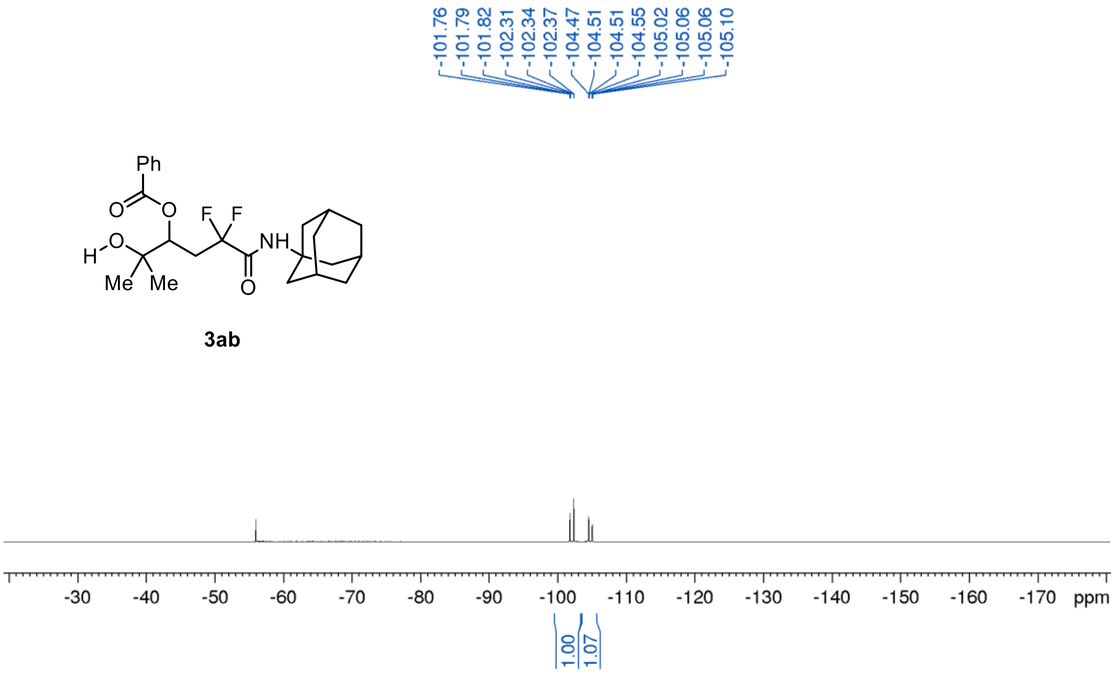

^{1}H NMR (500 MHz, $CDCl_3$, 25 ºC) of (3ac)

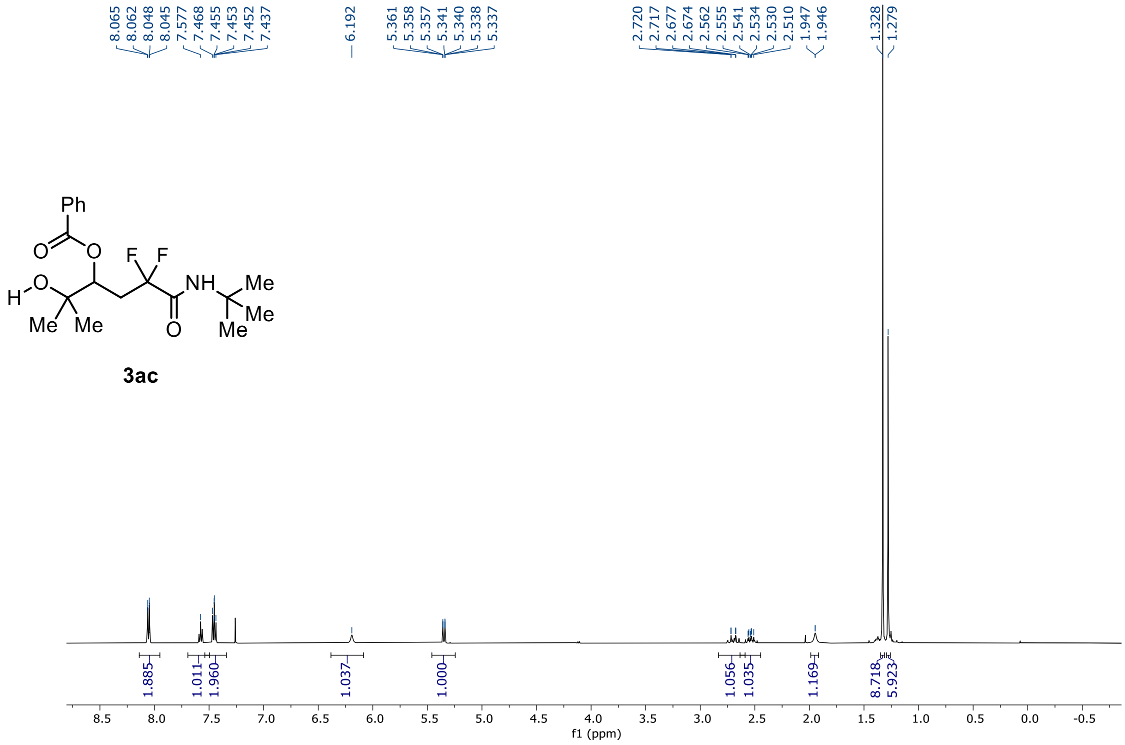

^{13}C NMR (126 MHz, $CDCl_3$, 25 ºC) of (3ac)

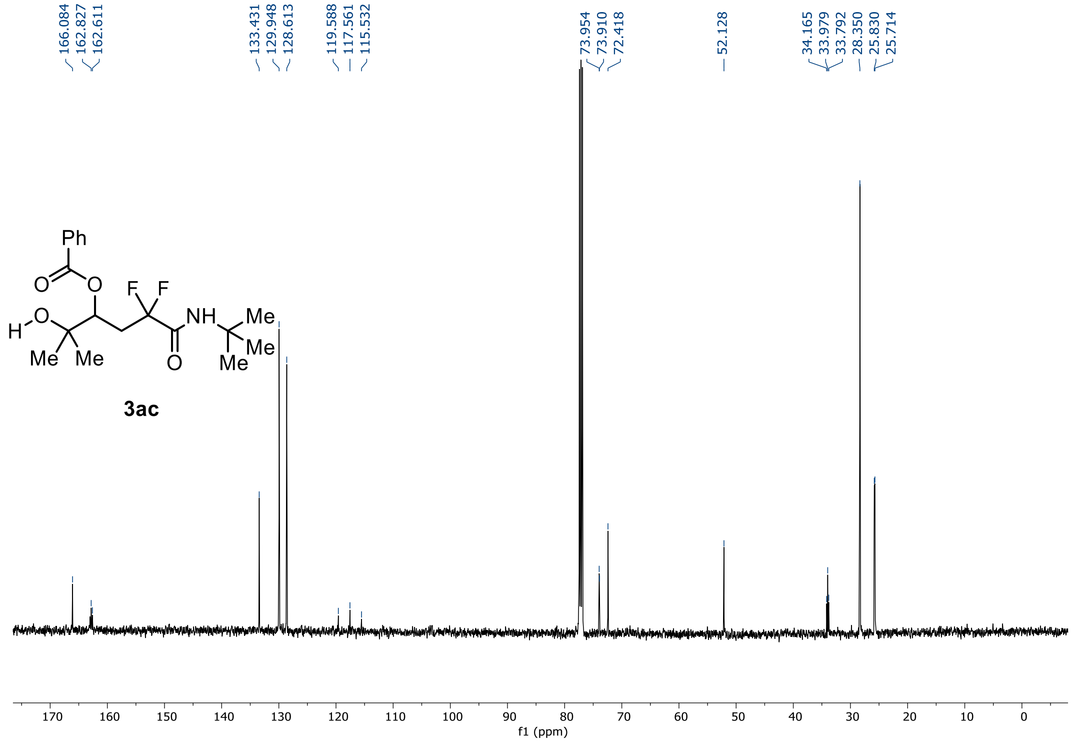

^{19}F NMR (470 MHz, CDCl$_3$**, 25 ºC) of (3ac)**

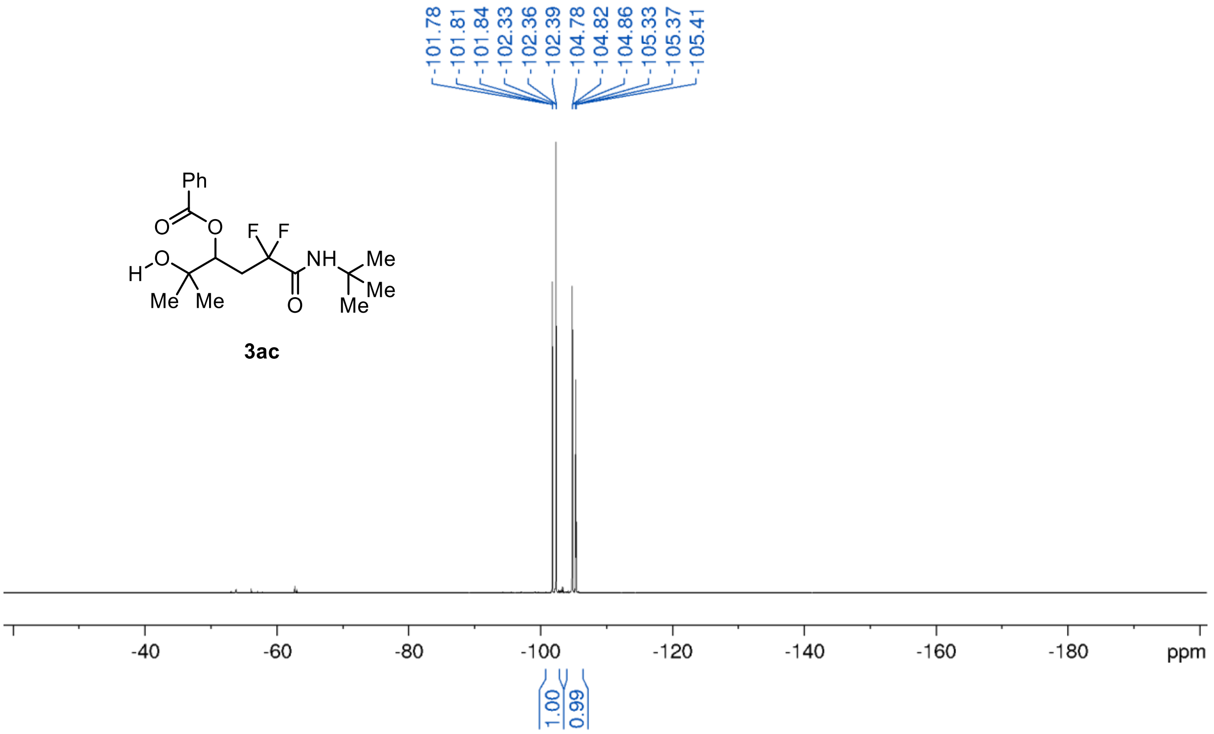

^{1}H NMR (500 MHz, $CDCl_3$, 25 ºC) of (3ad)

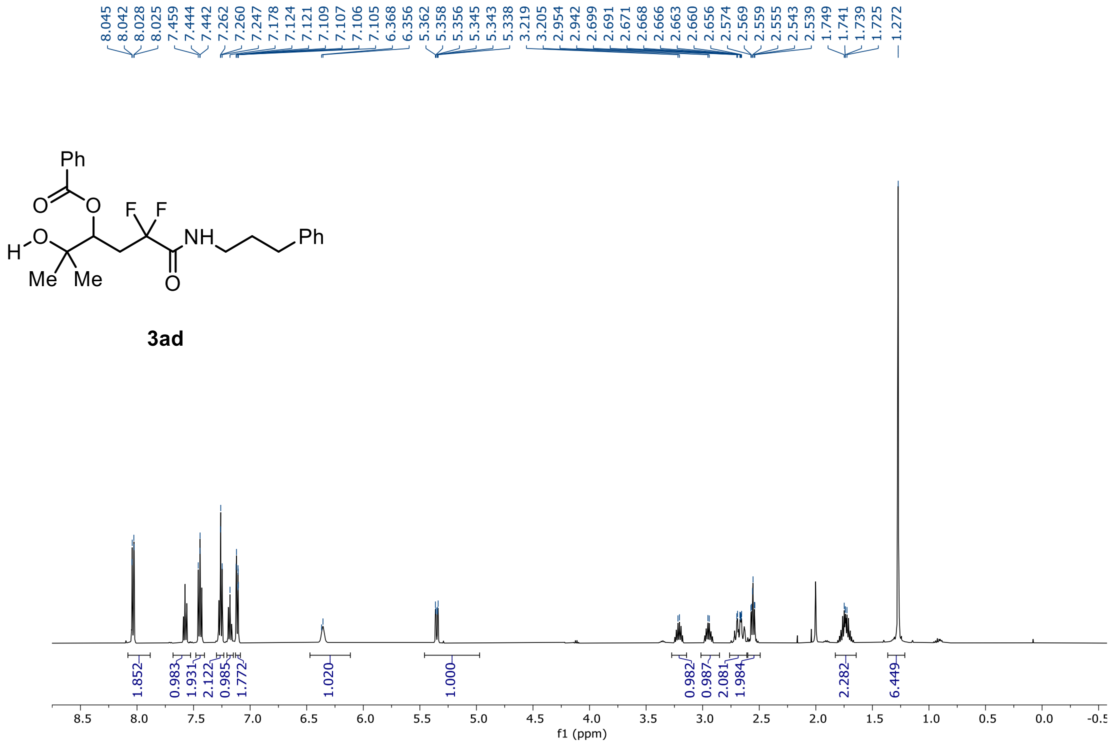

^{13}C NMR (126 MHz, $CDCl_3$, 25 ºC) of (3ad)

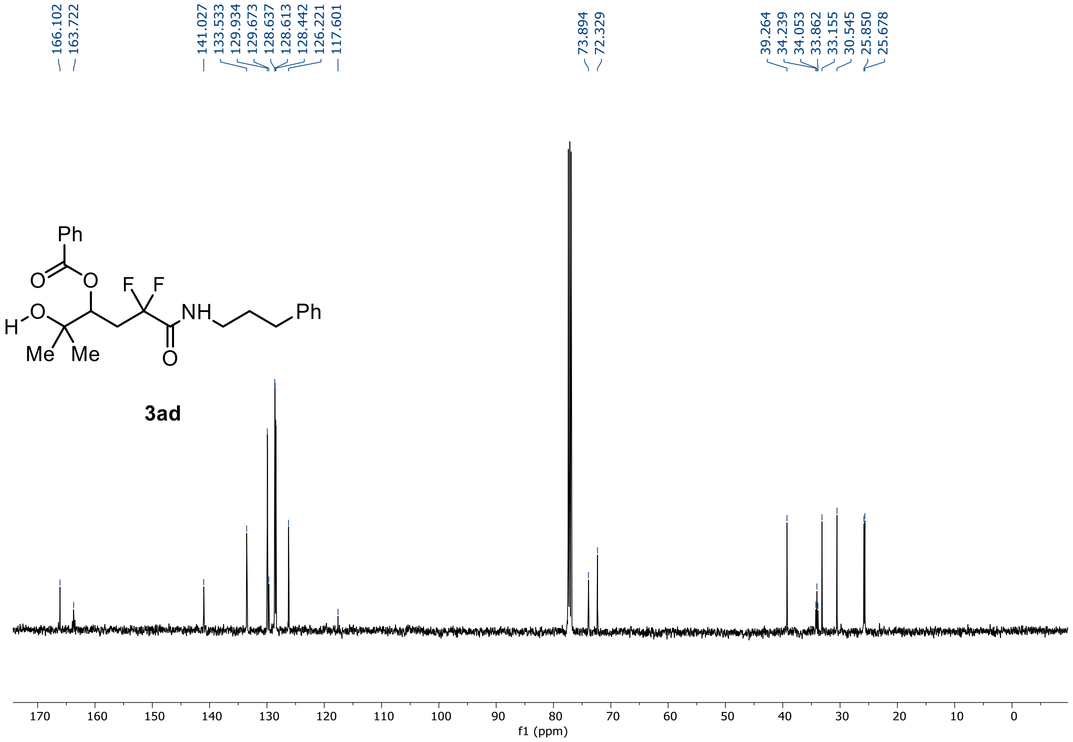

^{19}F NMR (470 MHz, $CDCl_3$**, 25 ºC) of (3ad)**

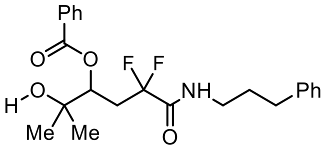

3ad

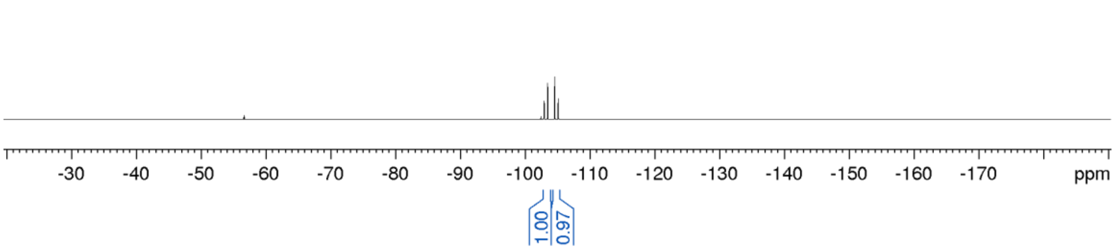

^{1}H NMR (500 MHz, $CDCl_3$, 25 ºC) of (3ae)

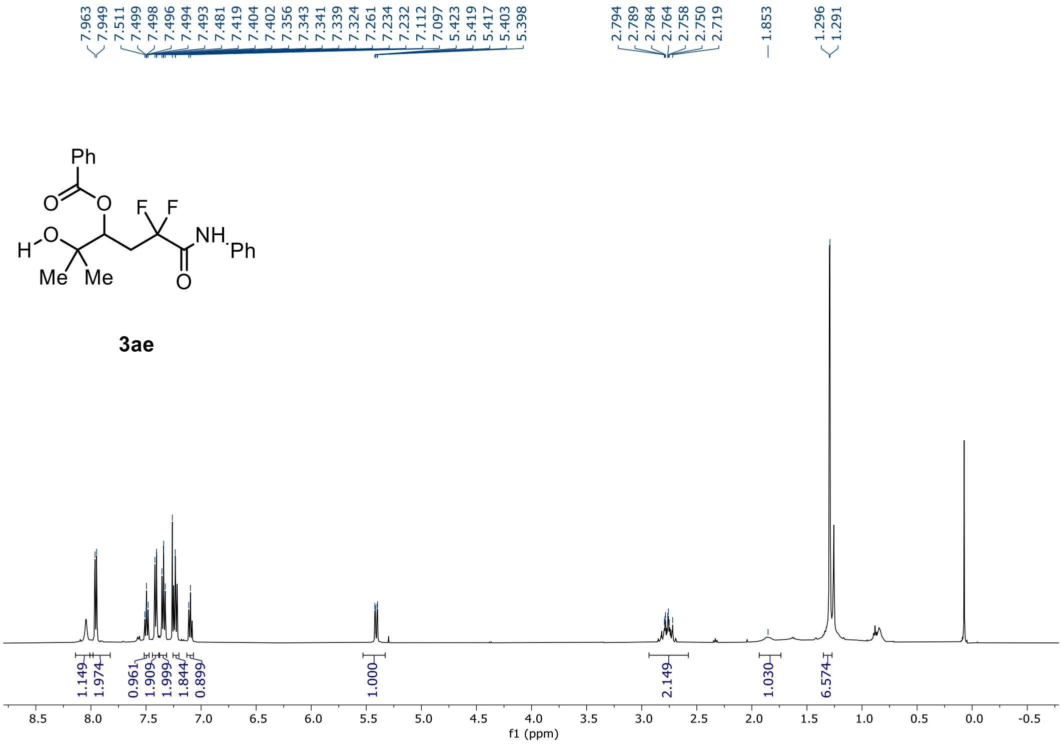

^{13}C NMR (175 MHz, $CDCl_3$, 25 ºC) of (3ae)

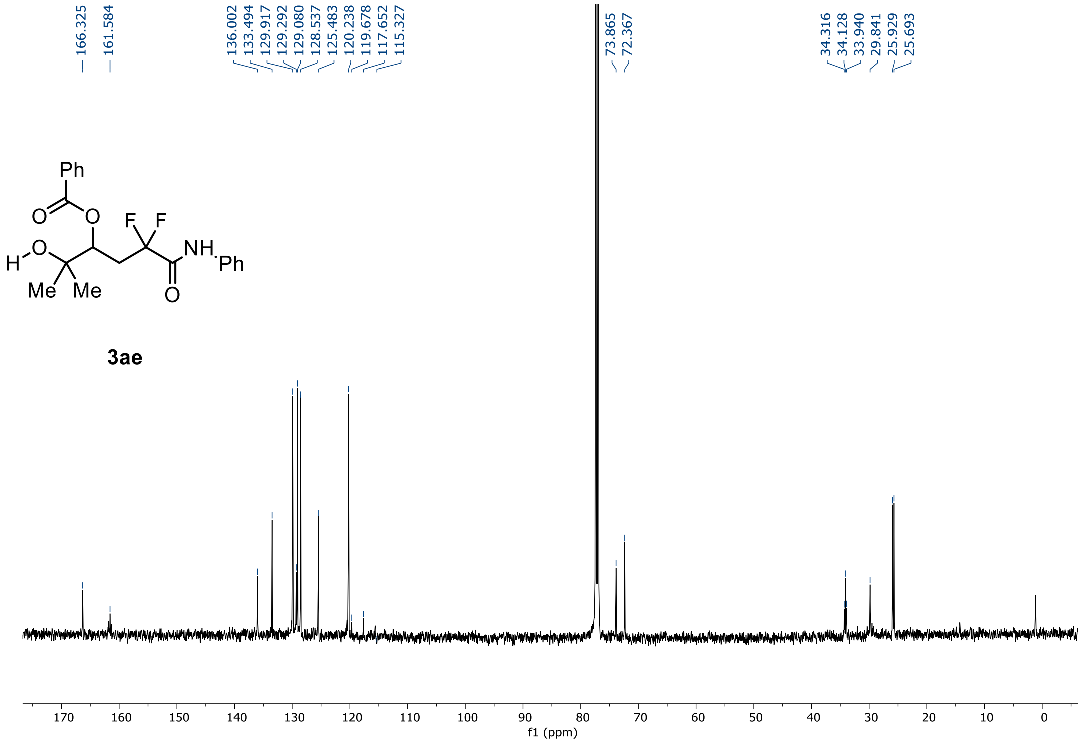

^{19}F NMR (376 MHz, CDCl3**, 25 ºC) of (3ae)**

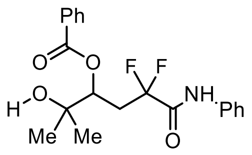

3ae

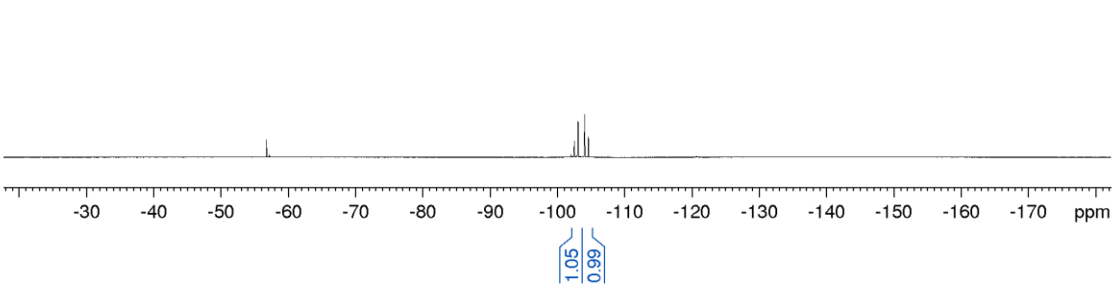

¹H NMR (500 MHz, CDCl$_3$**, 25 ºC) of (3af)**

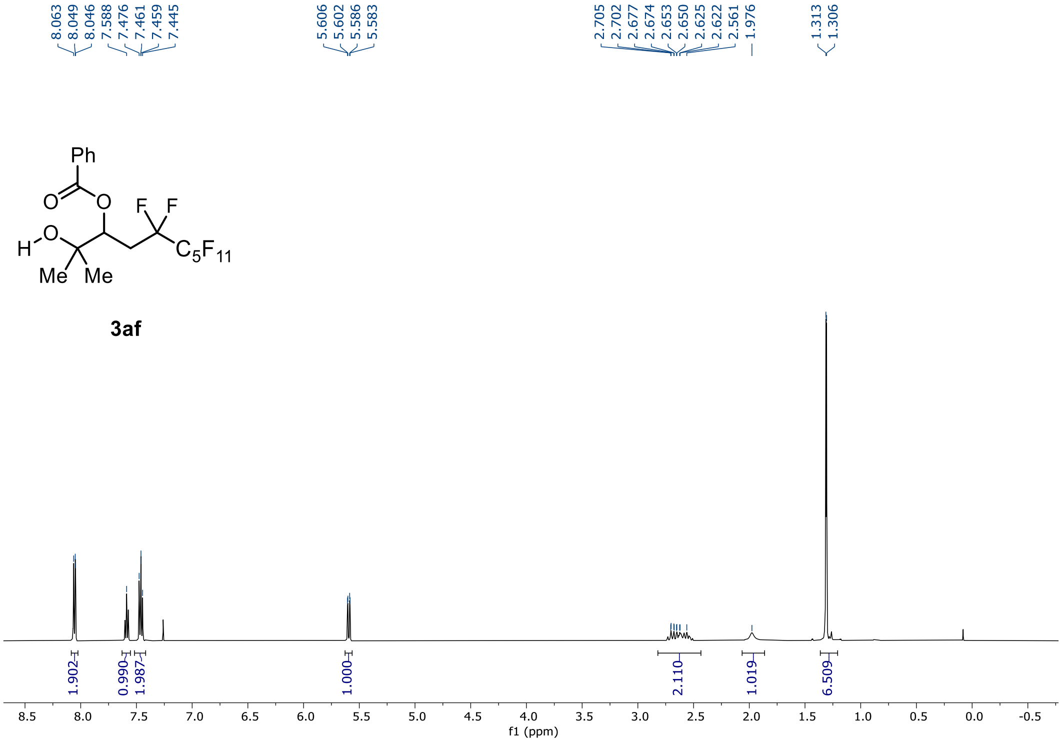

¹³C NMR (126 MHz, CDCl$_3$**, 25 ºC) of (3af)**

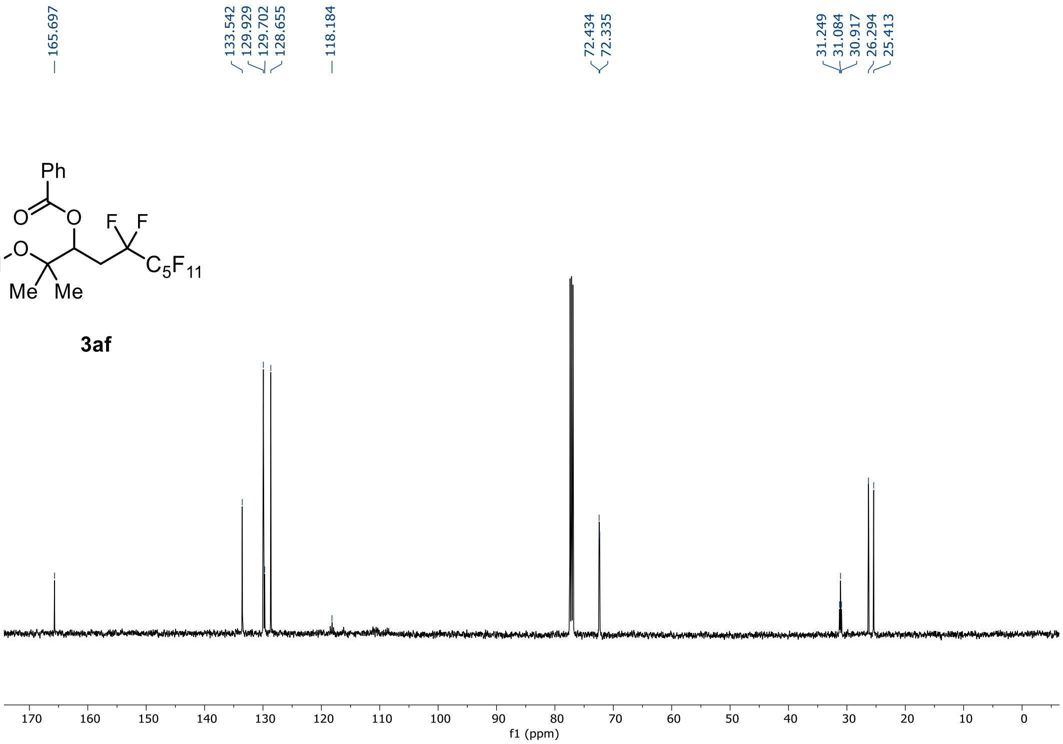

^{19}F NMR (470 MHz, CDCl$_3$, 25 ºC) of (3af)

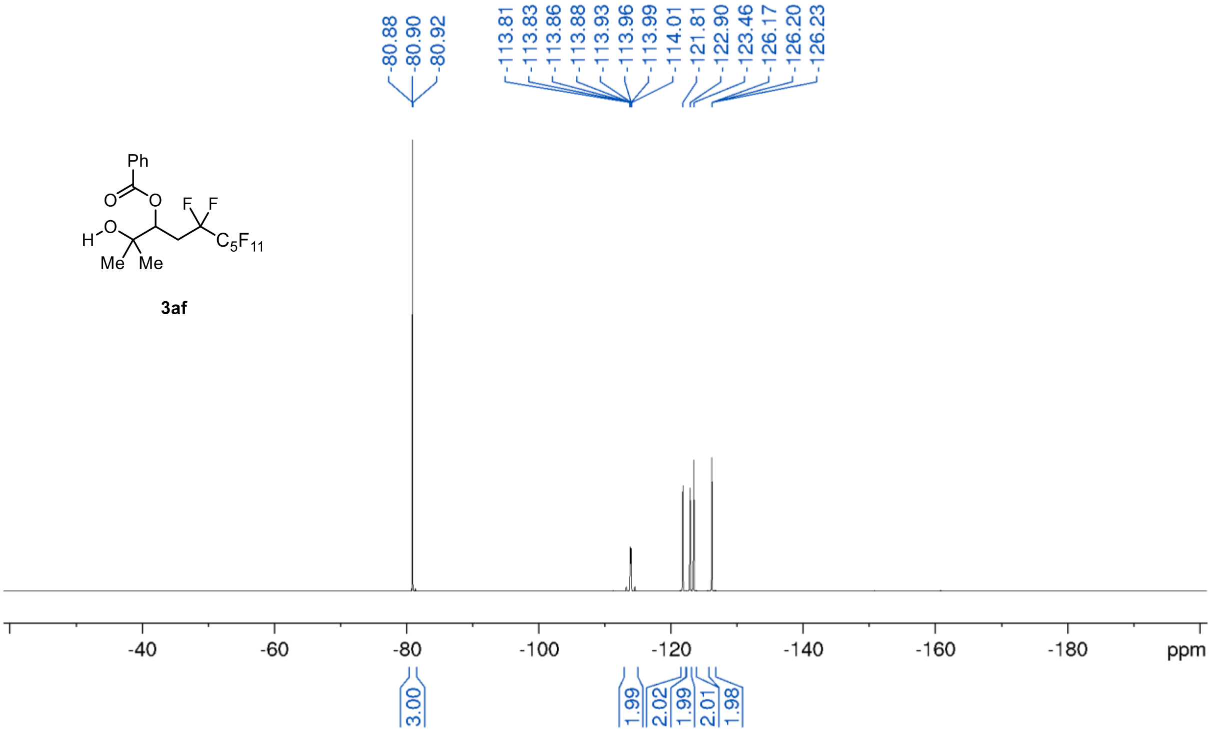

3af

^{1}H NMR (500 MHz, $CDCl_3$, 25 ºC) of (3ag)

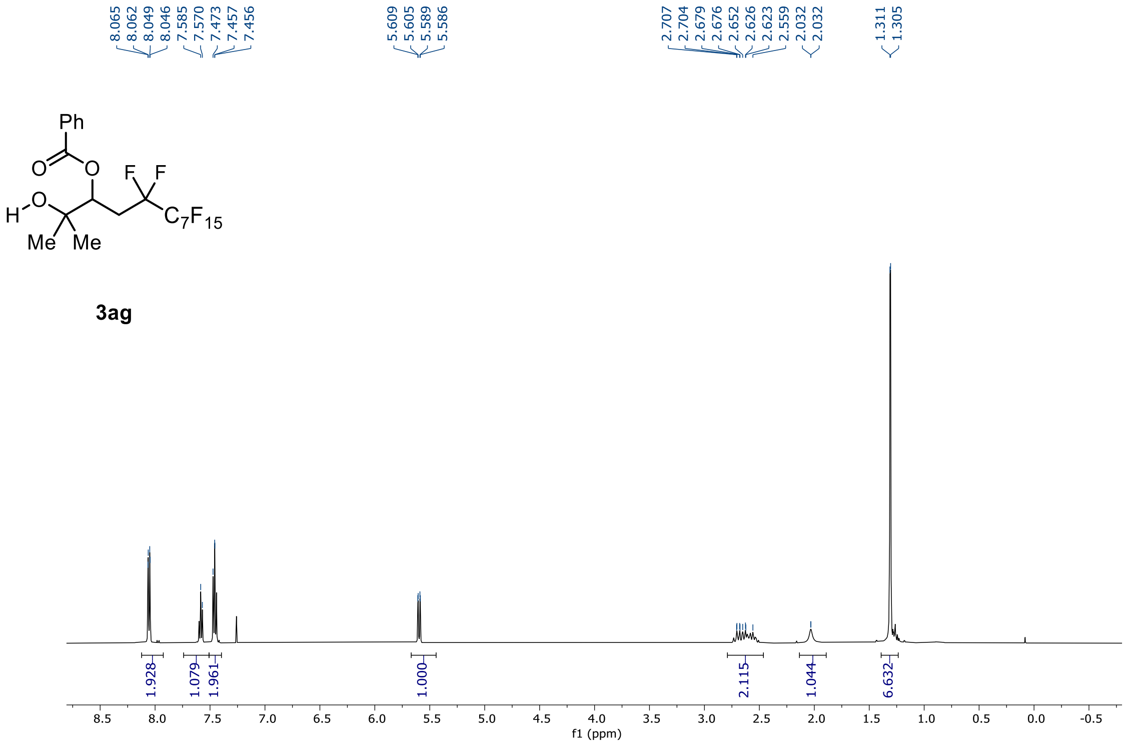

^{13}C NMR (126 MHz, $CDCl_3$, 25 ºC) of (3ag)

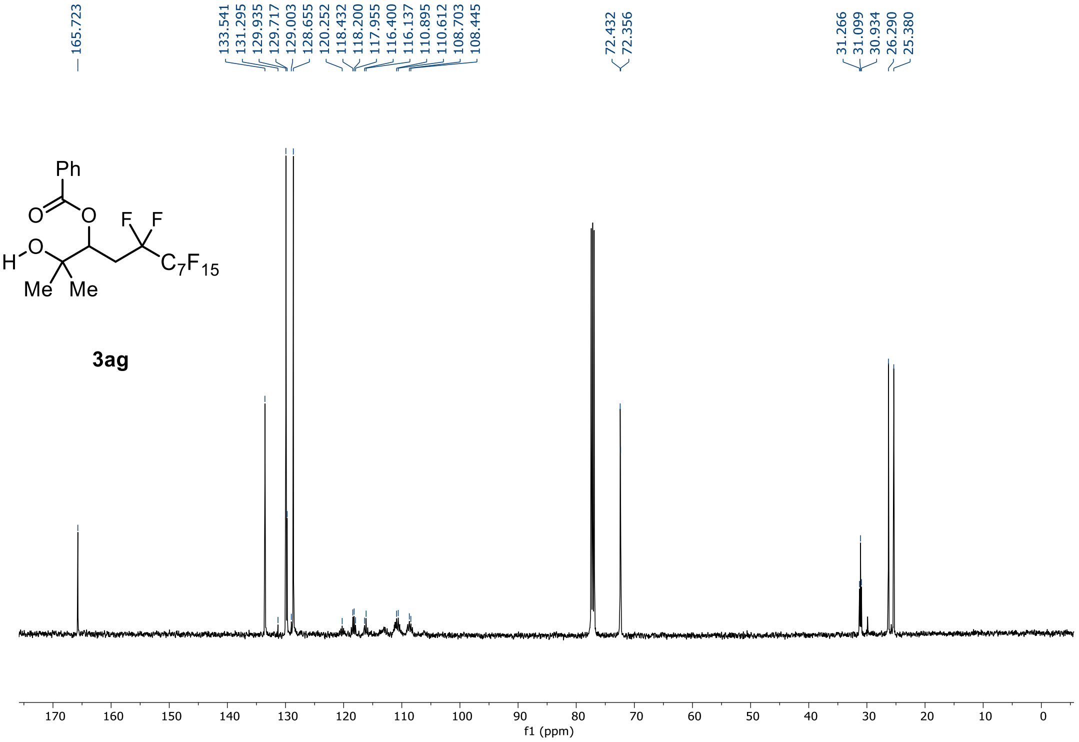

^{19}F NMR (470 MHz, $CDCl_3$**, 25 ºC) of (3ag)**

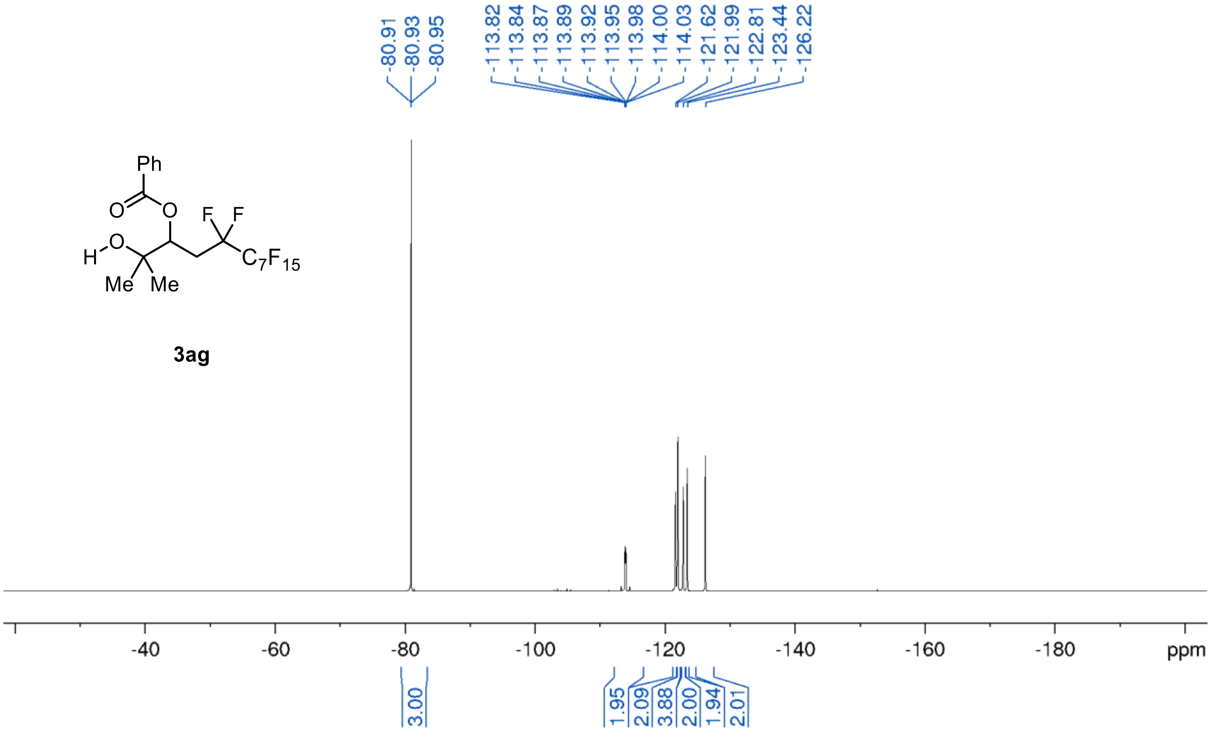

^{1}H NMR (500 MHz, $CDCl_3$, 25 ºC) of (3ah)

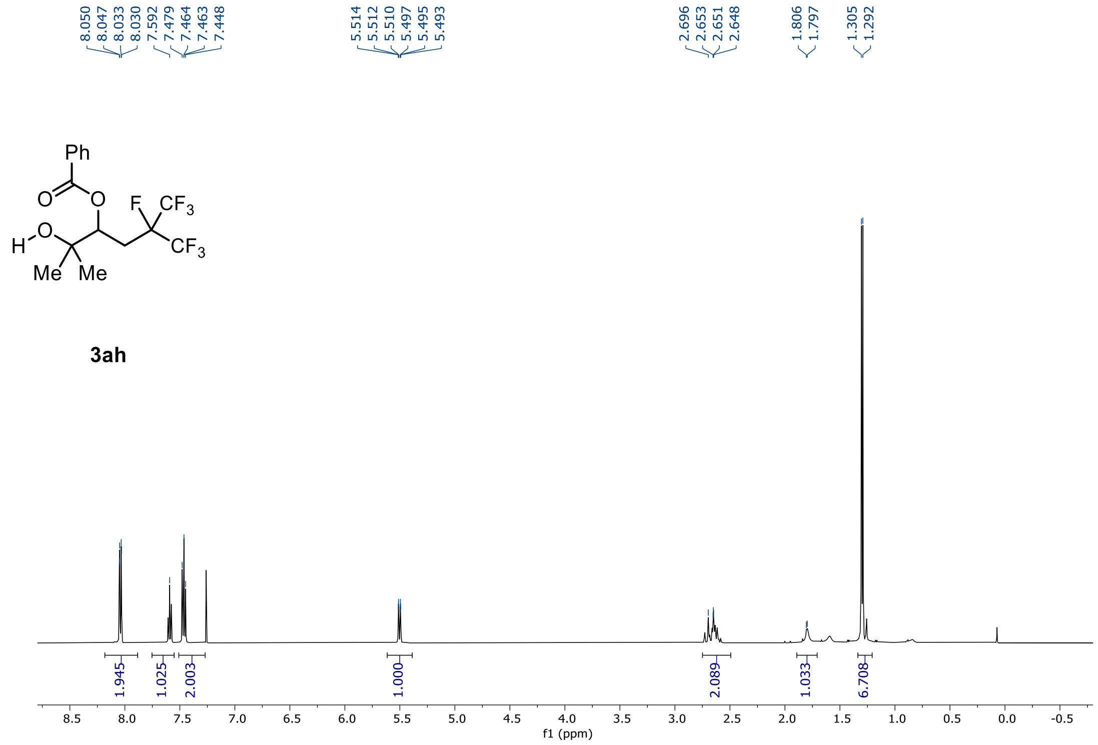

^{13}C NMR (126 MHz, $CDCl_3$, 25 ºC) of (3ah)

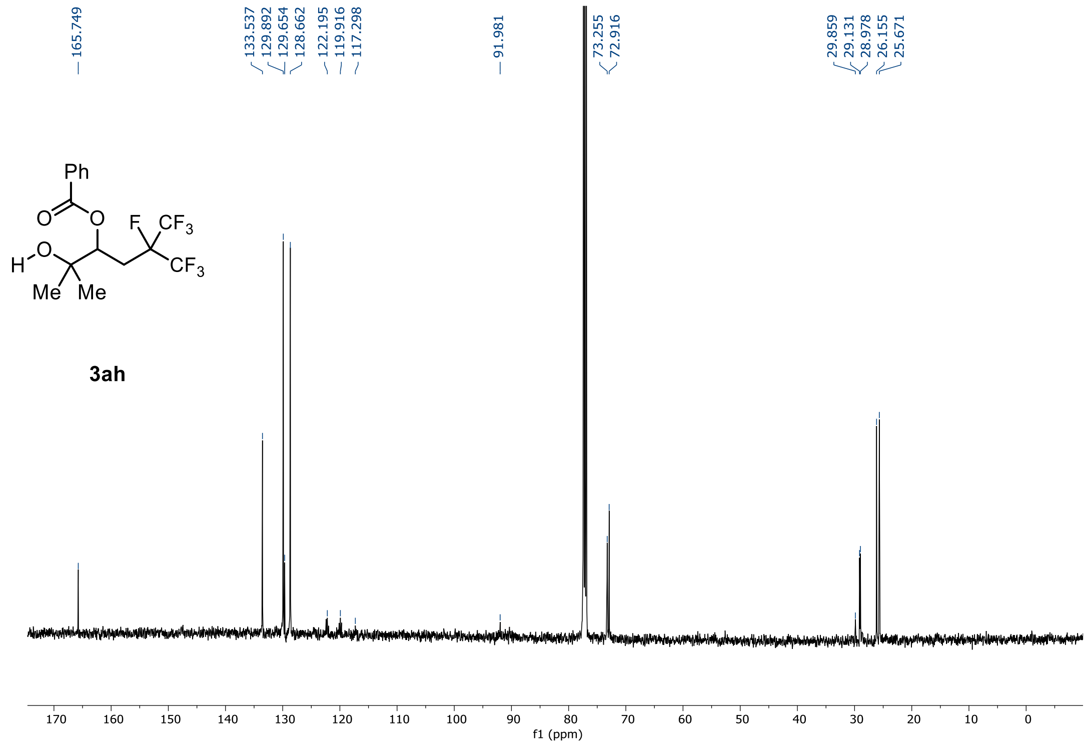

^{19}F NMR (470 MHz, CDCl$_3$**, 25 ºC) of (3ah)**

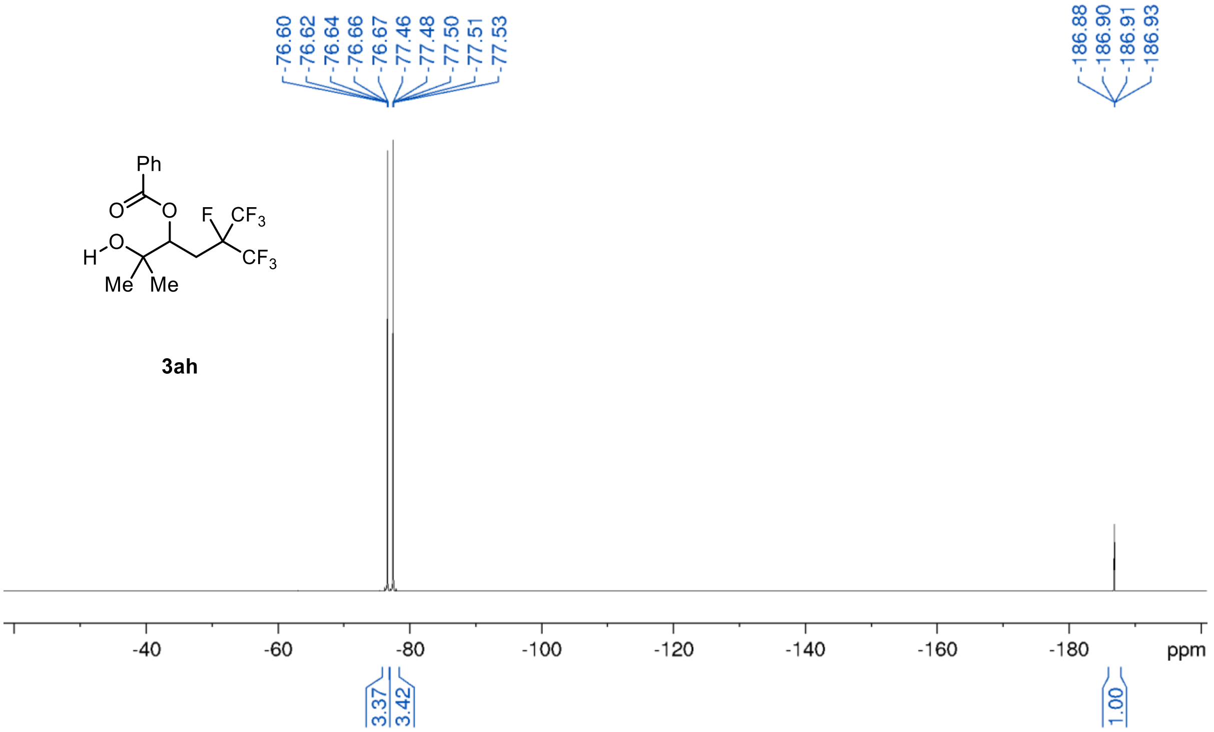

^{1}H NMR (500 MHz, CDCl$_3$**, 25 ºC) of (3ai)**

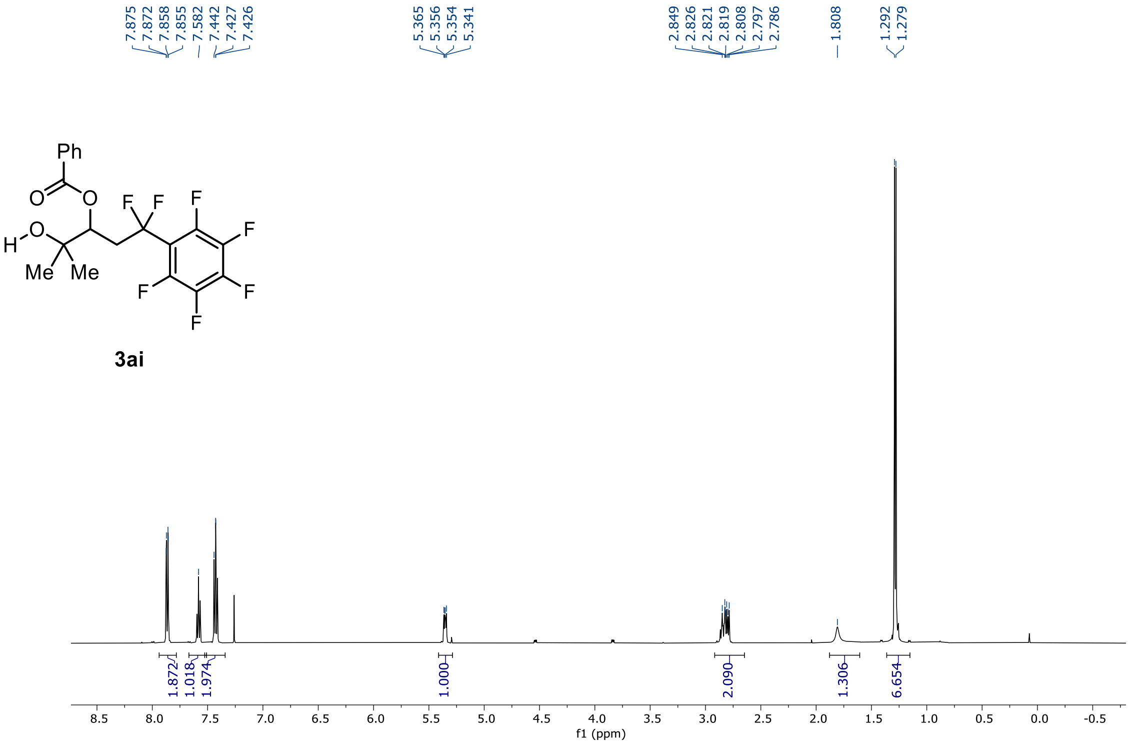

^{13}C NMR (126 MHz, CDCl$_3$**, 25 ºC) of (3ai)**

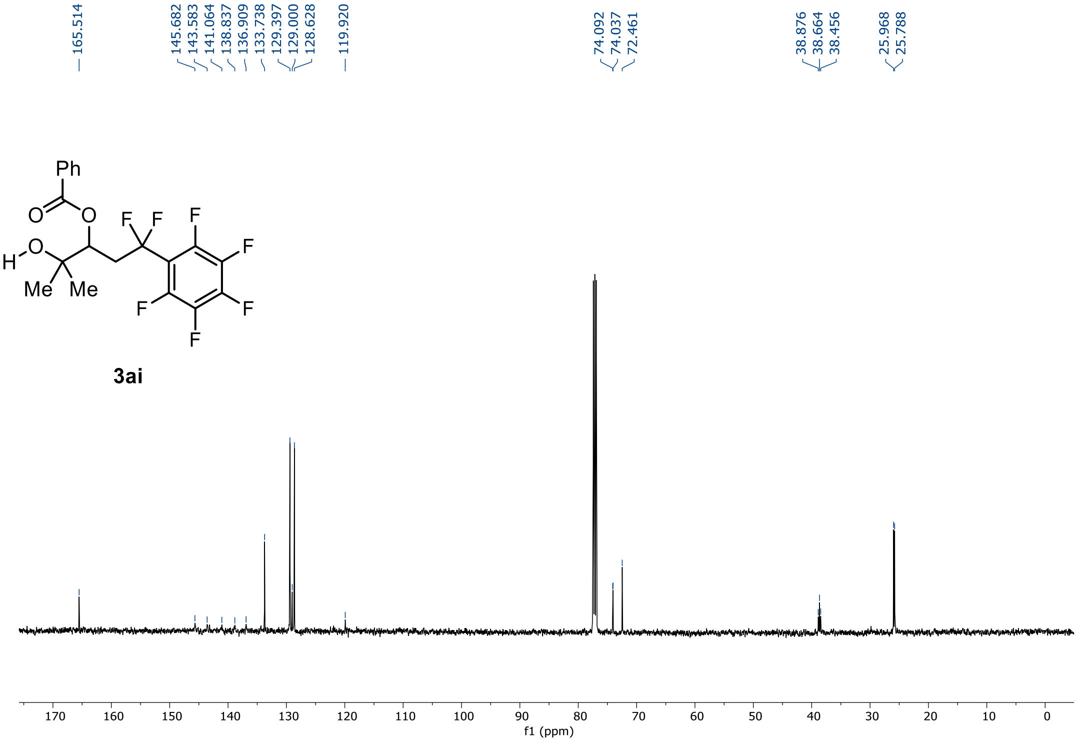

^{19}F NMR (470 MHz, CDCl$_3$**, 25 ºC) of (3ai)**

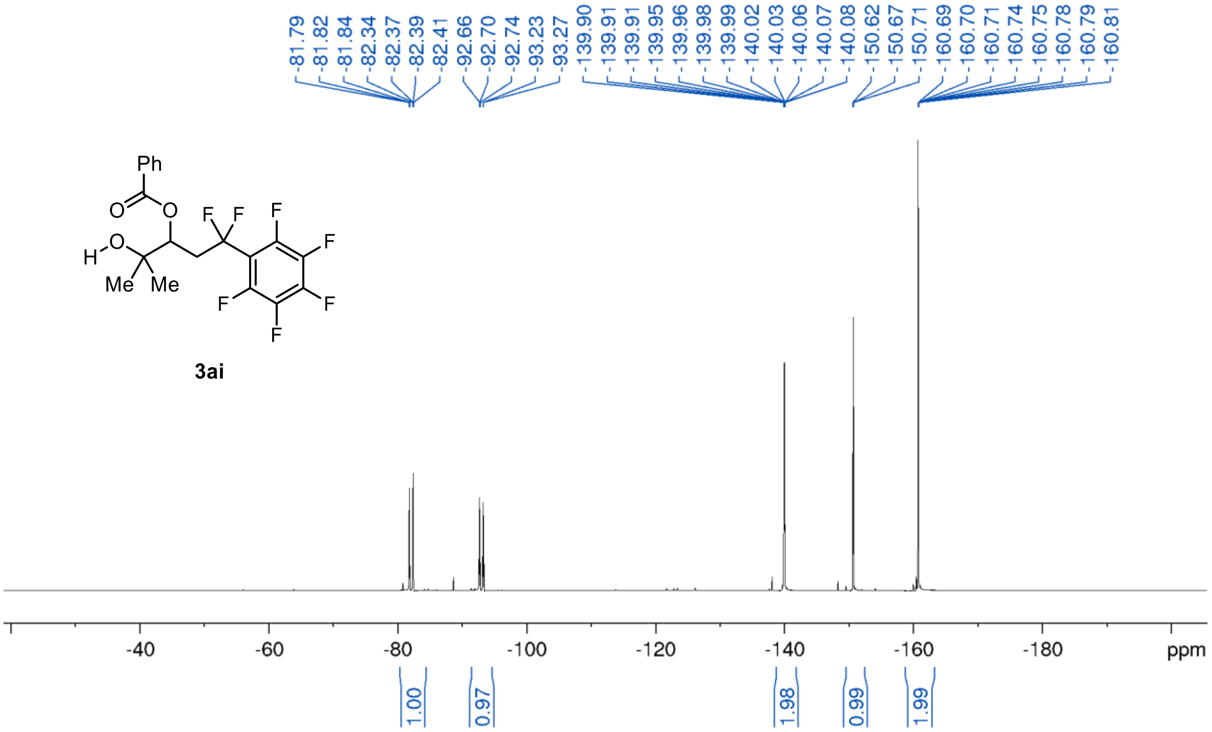

3ai

^{1}H NMR (500 MHz, $CDCl_3$, 25 ºC) of (3aj)

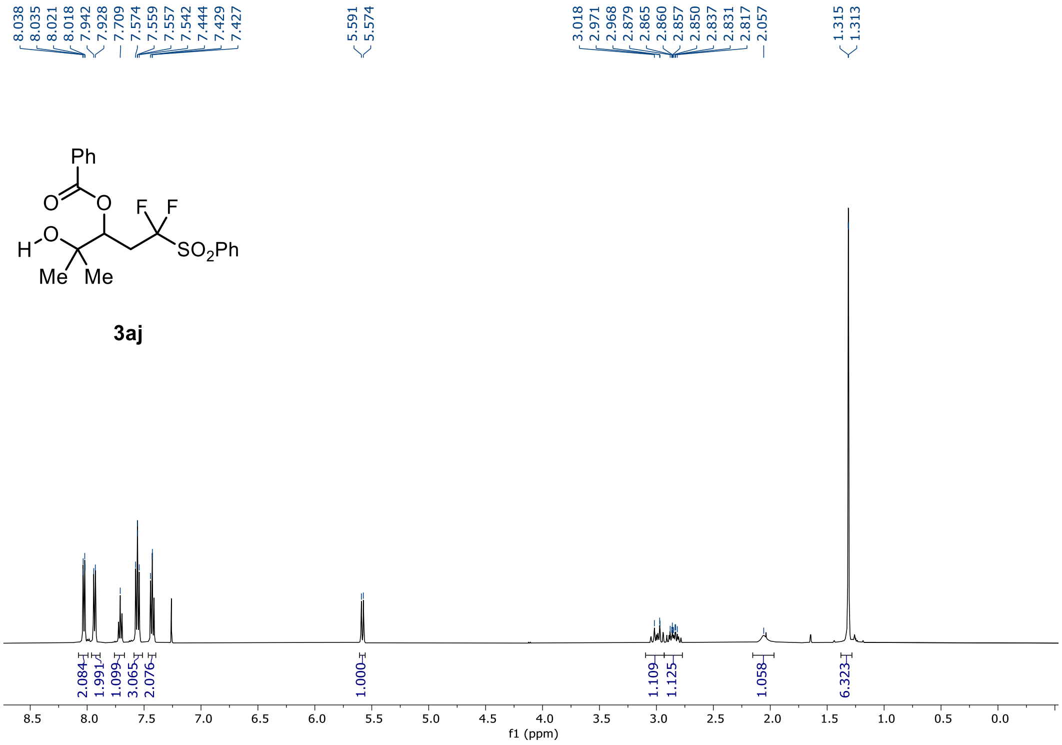

^{13}C NMR (126 MHz, $CDCl_3$, 25 ºC) of (3aj)

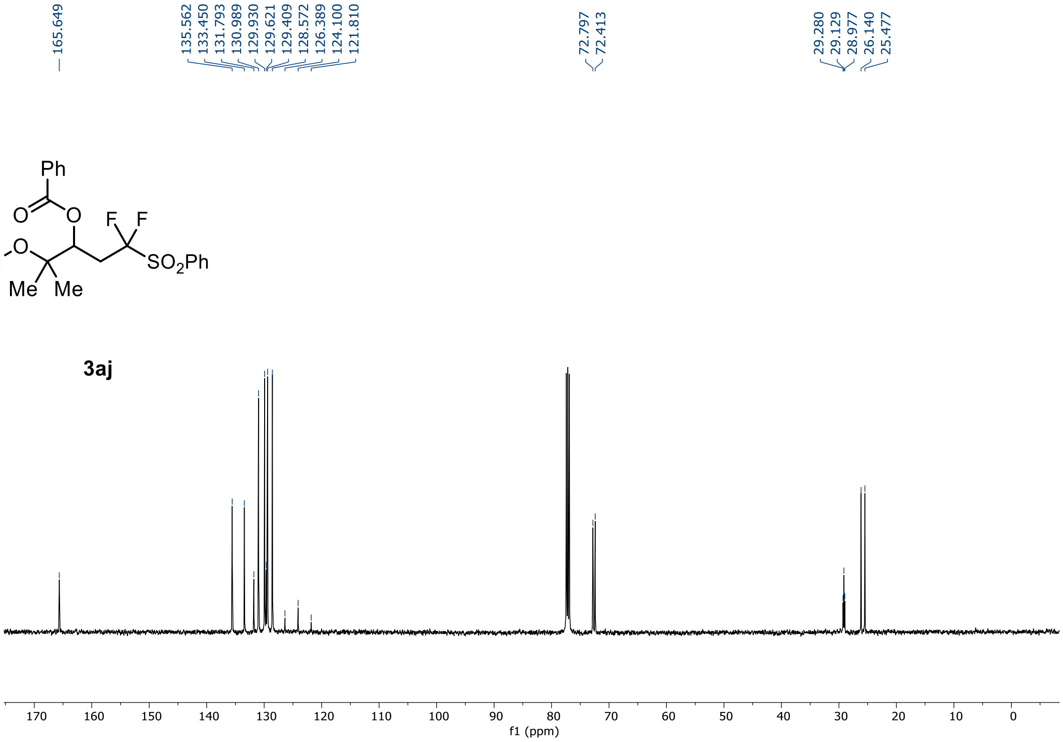

^{19}F NMR (470 MHz, CDCl$_3$**, 25 ºC) of (3aj)**

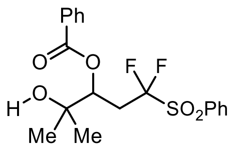

3aj

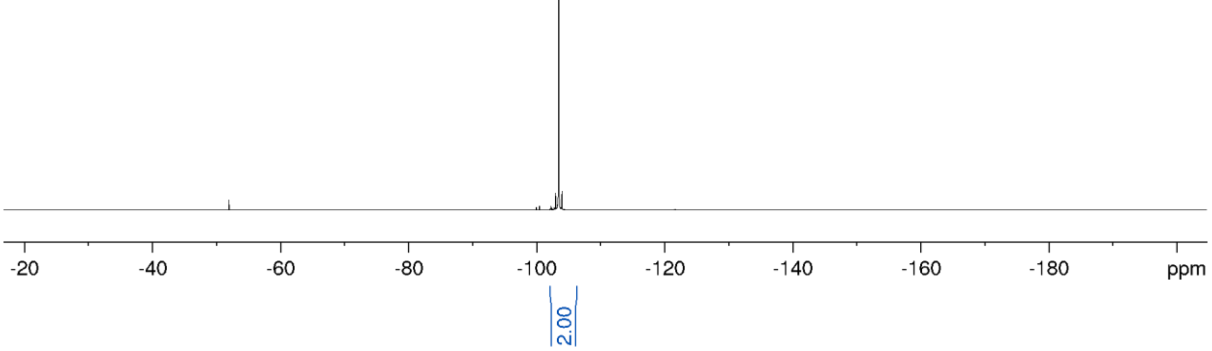

^{1}H NMR (500 MHz, $CDCl_3$, 25 ºC) of (3ak)

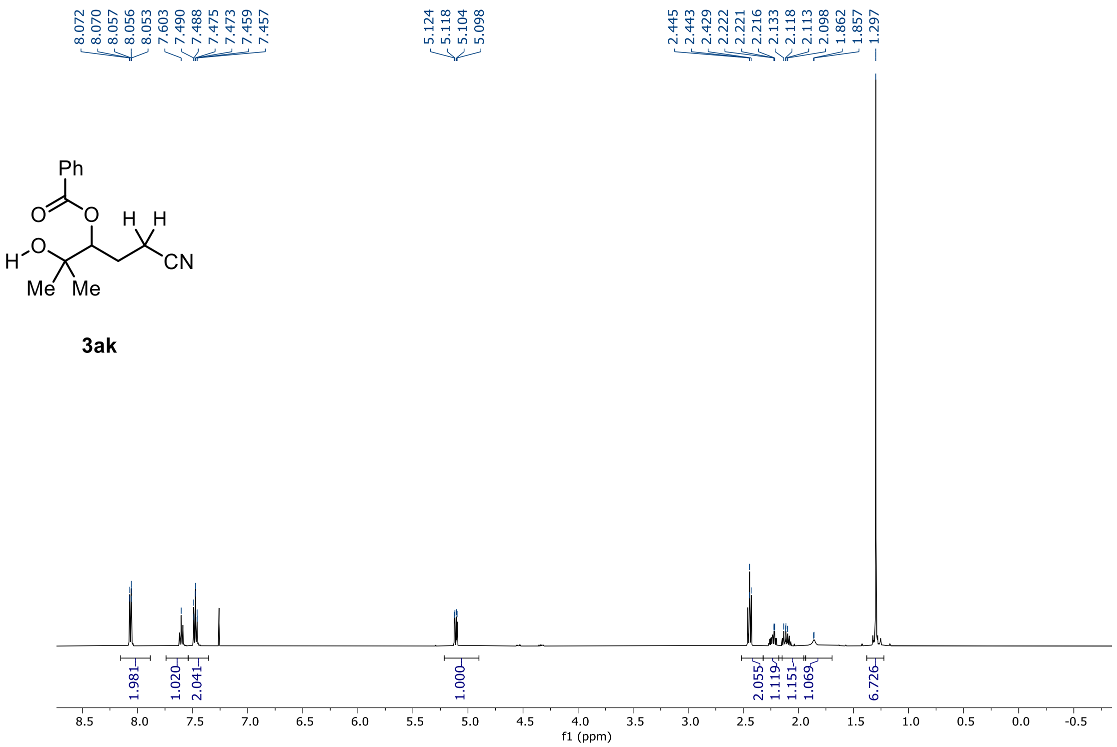

^{13}C NMR (126 MHz, $CDCl_3$, 25 ºC) of (3ak)

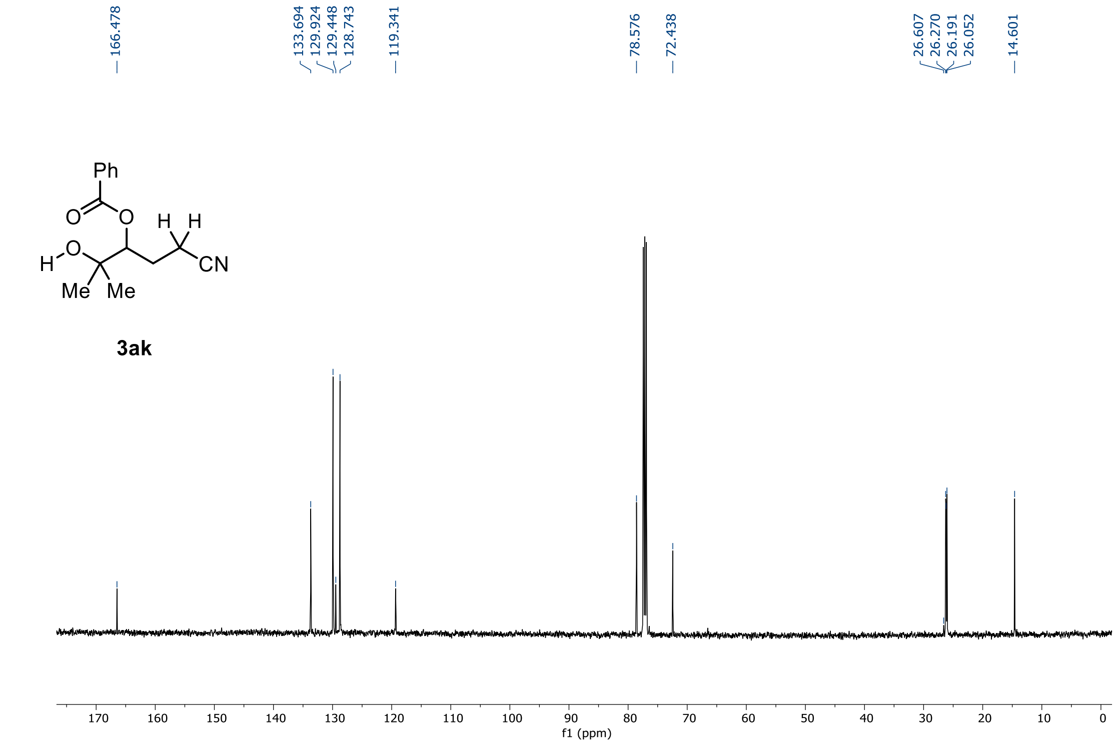

^{1}H NMR (500 MHz, CDCl3, **25 ºC) of (3al)**

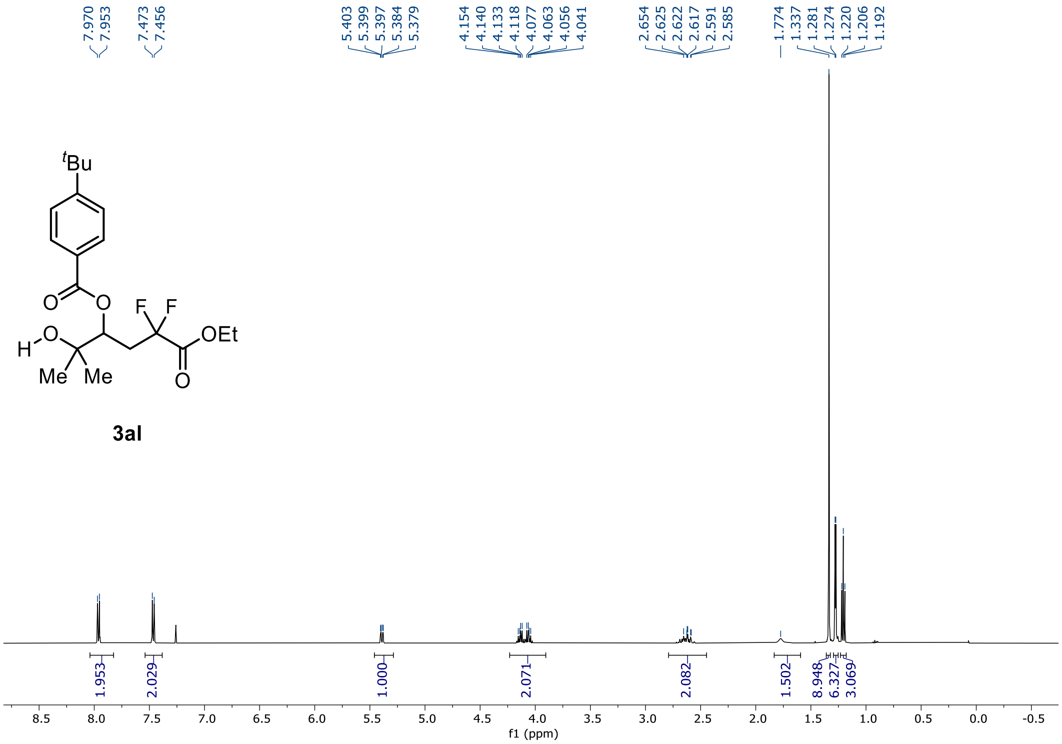

^{13}C NMR (126 MHz, CDCl3, **25 ºC) of (3al)**

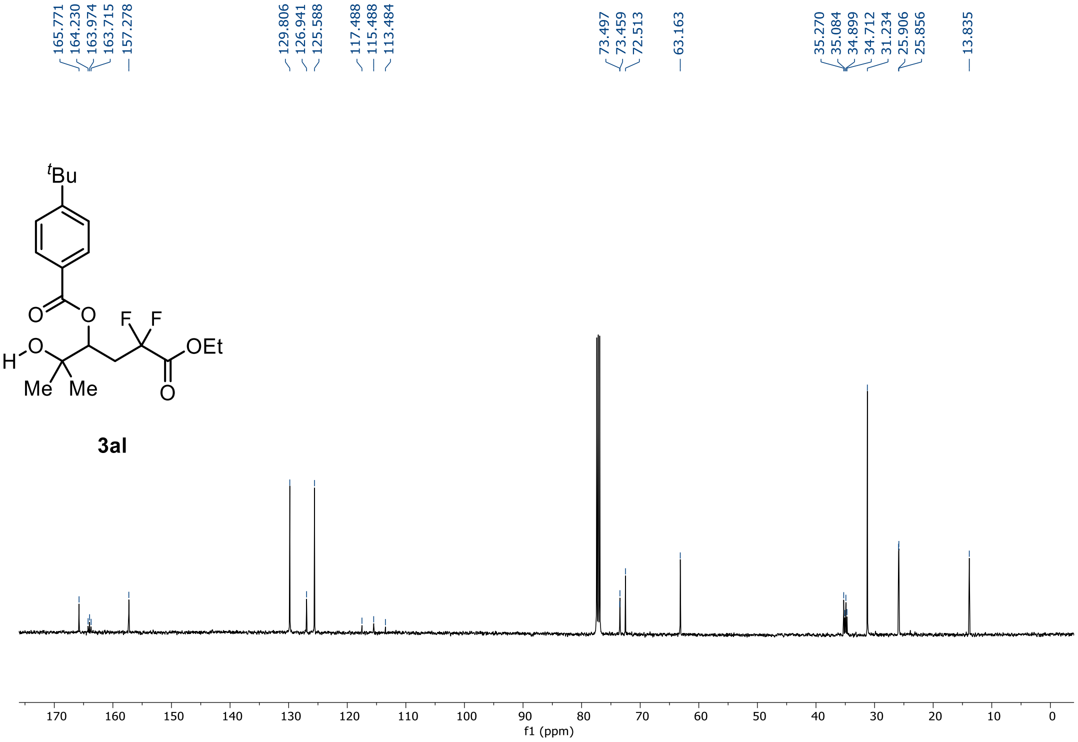

^{19}F NMR (470 MHz, $CDCl_3$**, 25 ºC) of (3al)**

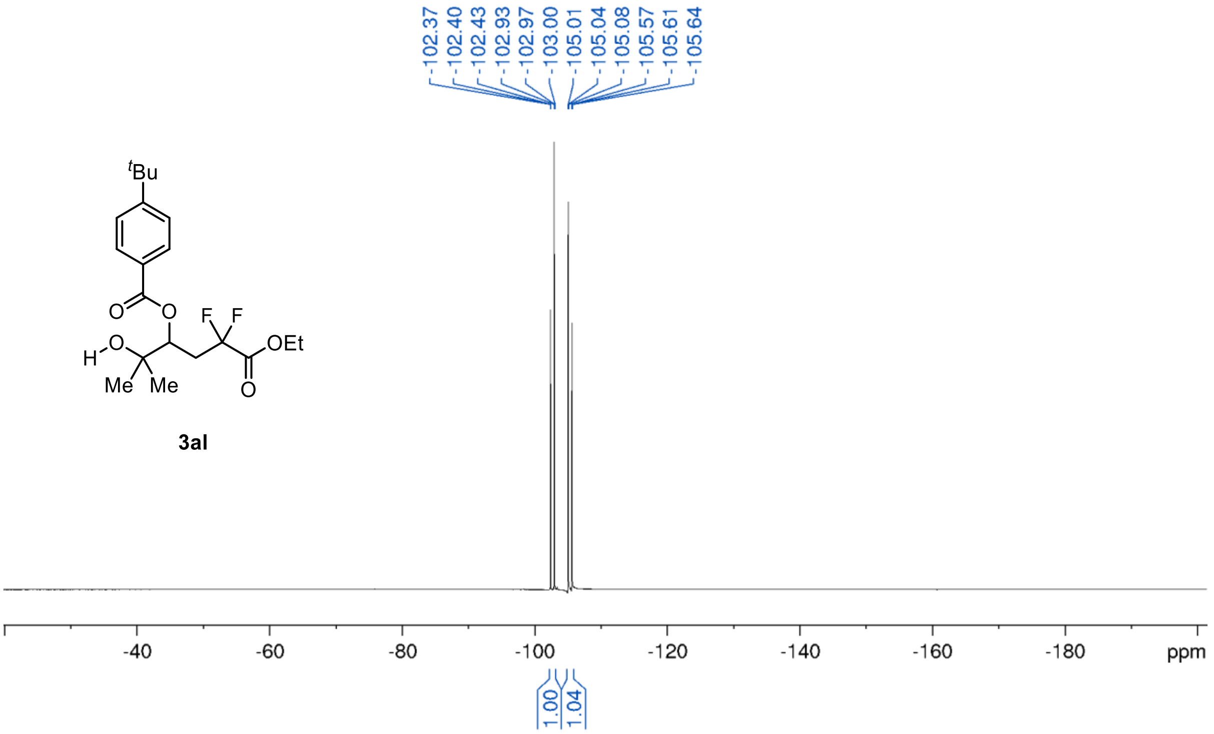

^{1}H NMR (500 MHz, $CDCl_3$, 25 ºC) of (3am)

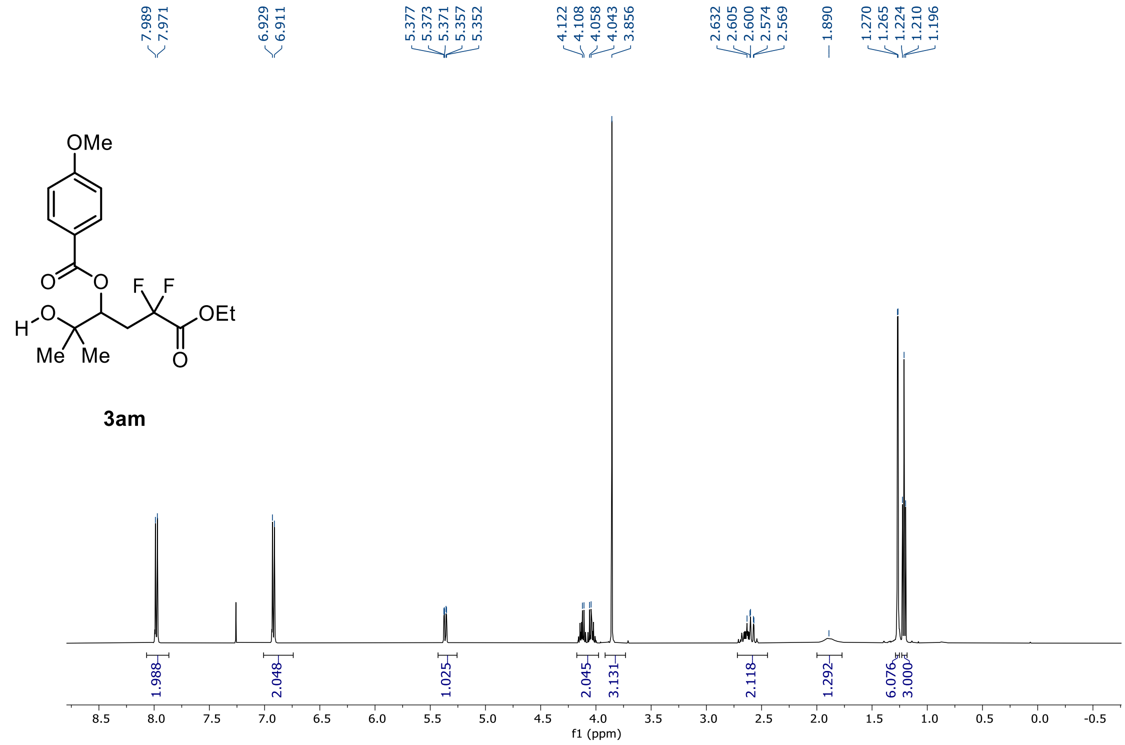

^{13}C NMR (126 MHz, $CDCl_3$, 25 ºC) of (3am)

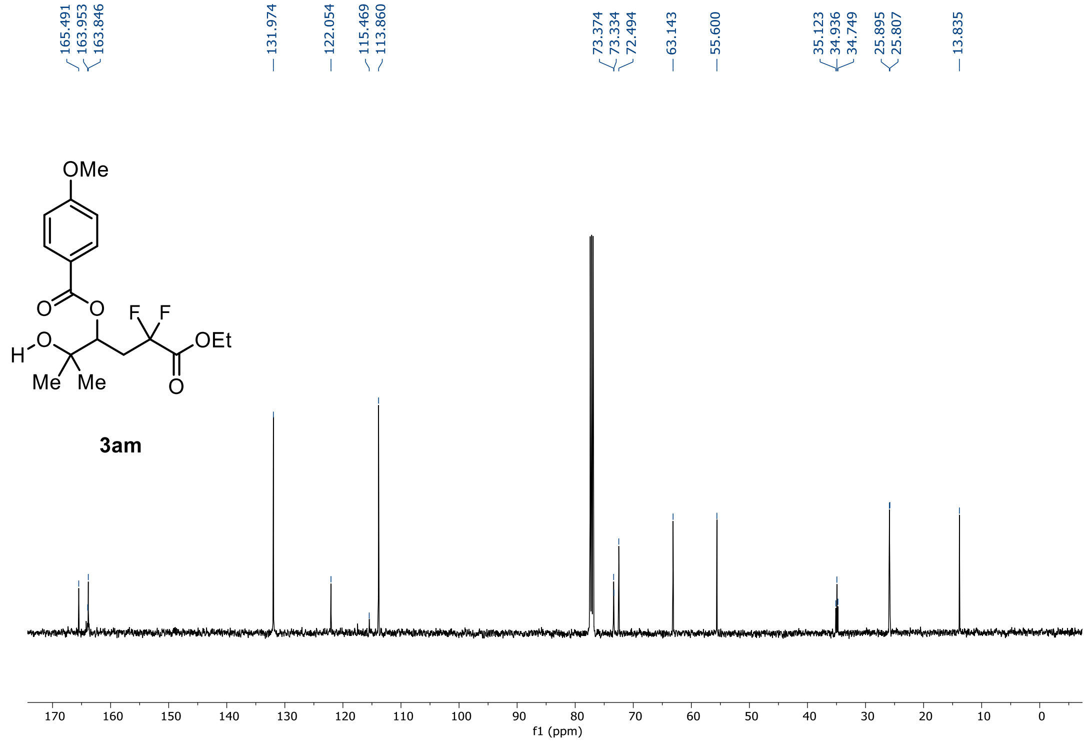

^{19}F NMR (376 MHz, CDCl$_3$**, 25 ºC) of (3am)**

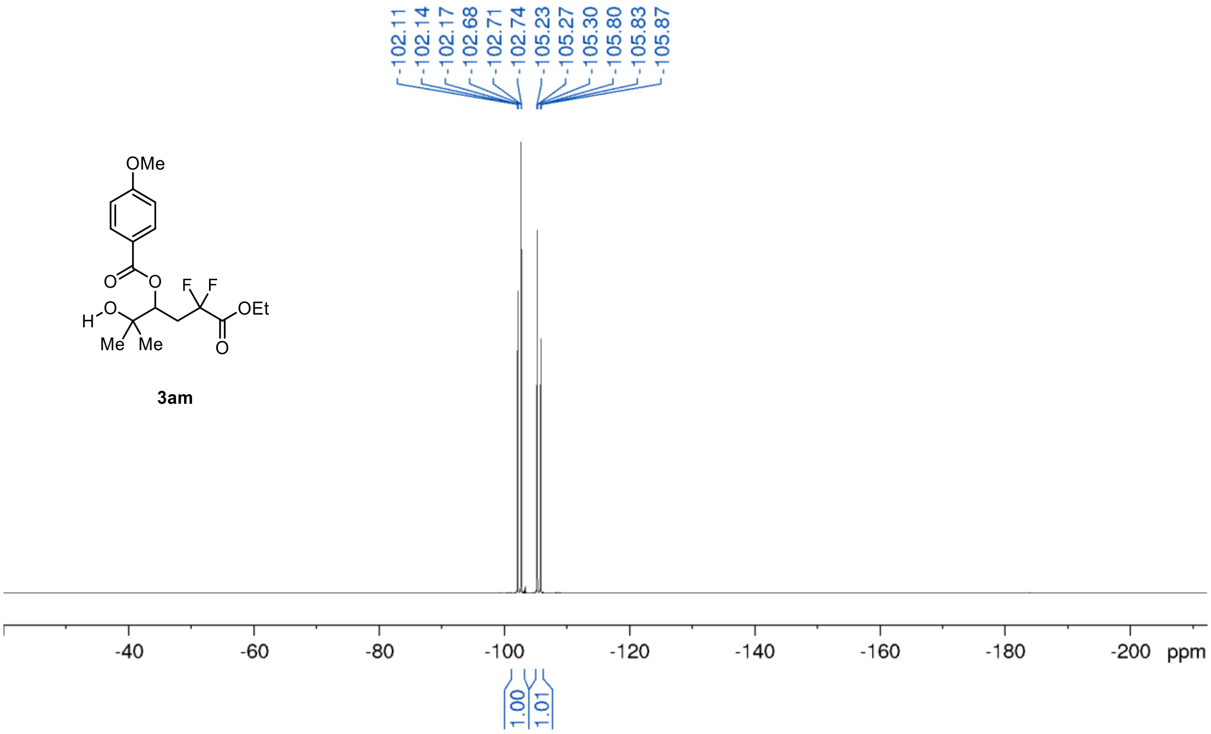

^{1}H NMR (500 MHz, CDCl3**, 25 ºC) of (3an)**

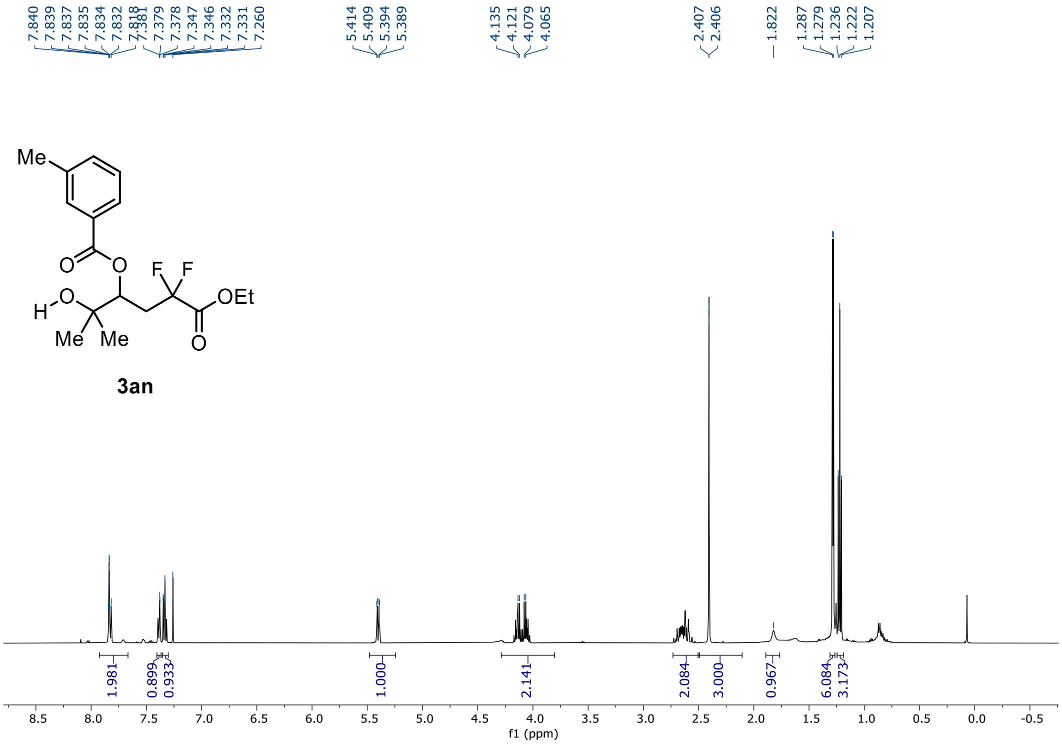

^{13}C NMR (175 MHz, CDCl3**, 25 ºC) of (3an)**

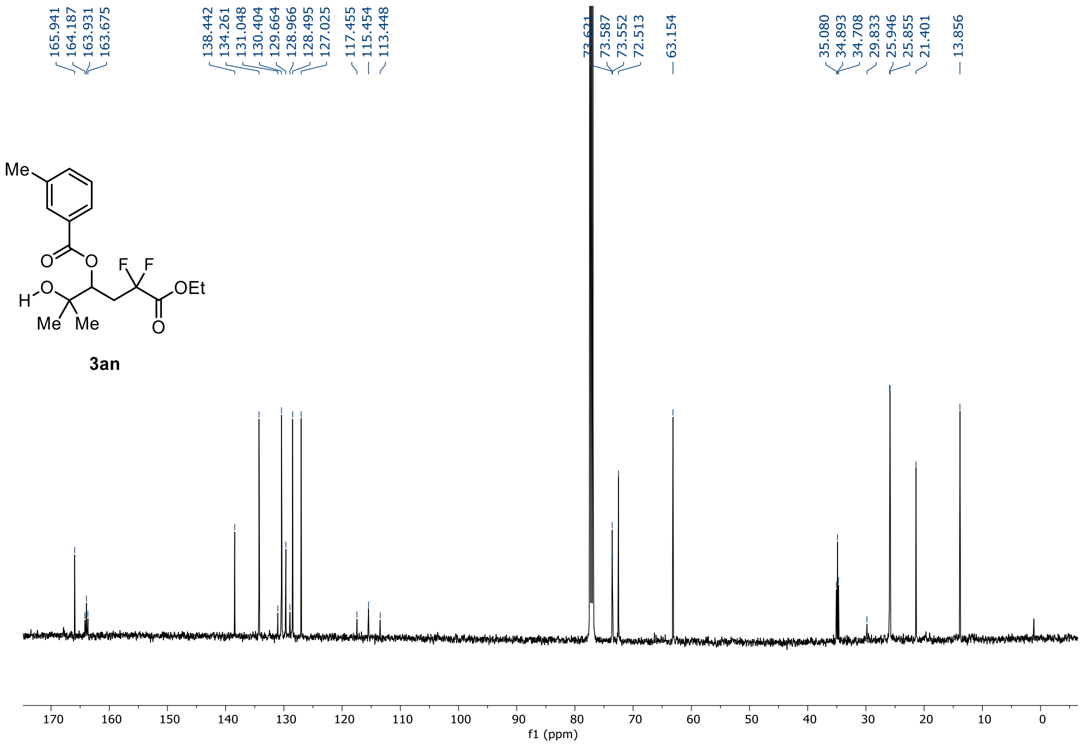

^{19}F NMR (376 MHz, CDCl3, 25 ºC) of (3an)

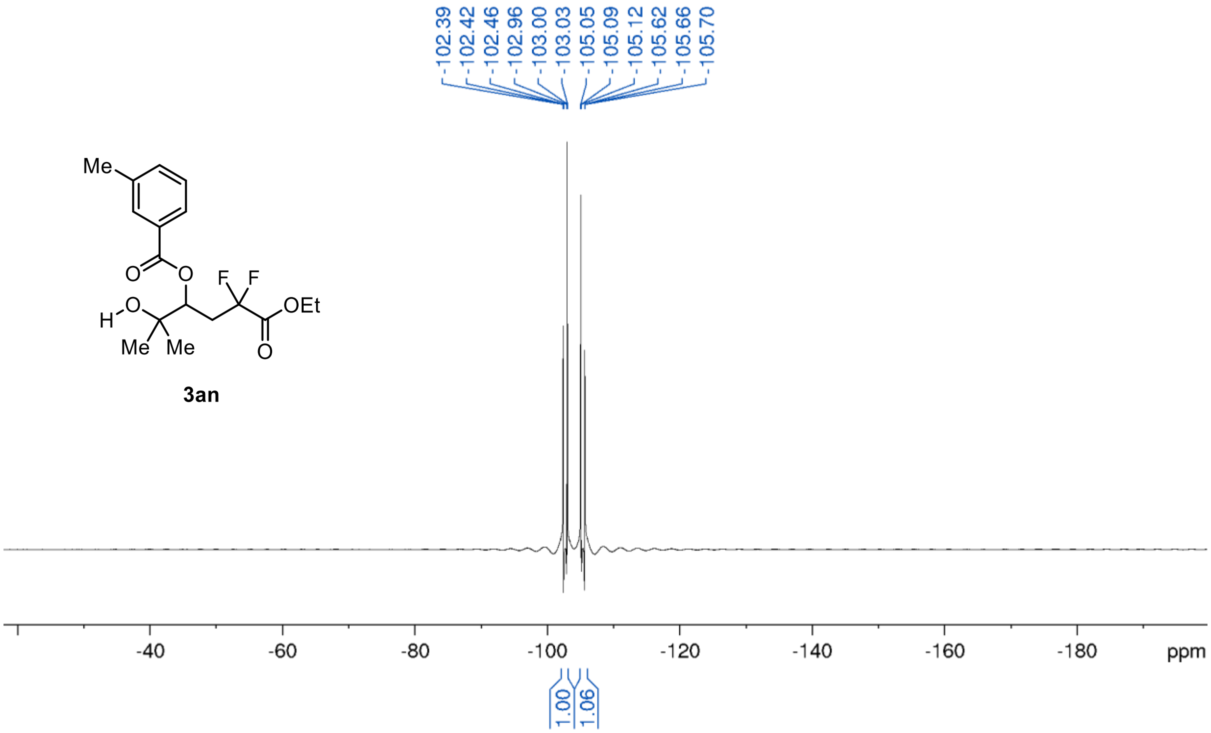

^{1}H NMR (500 MHz, $CDCl_3$, 25 ºC) of (3ao)

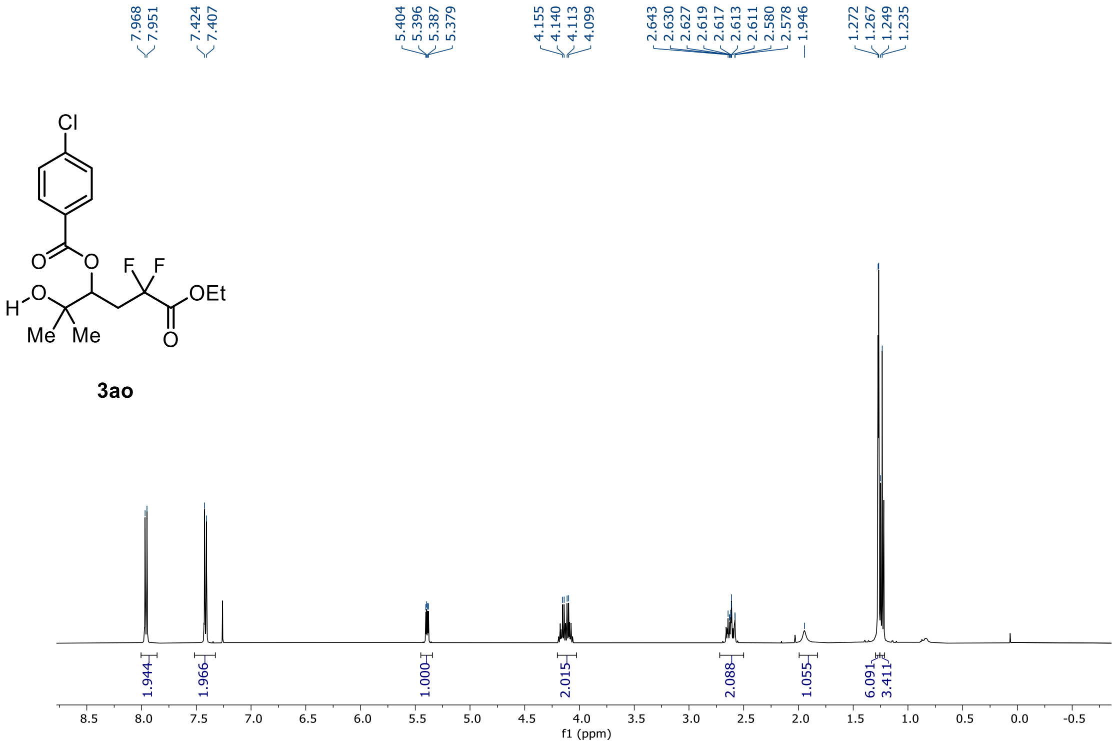

^{13}C NMR (126 MHz, $CDCl_3$, 25 ºC) of (3ao)

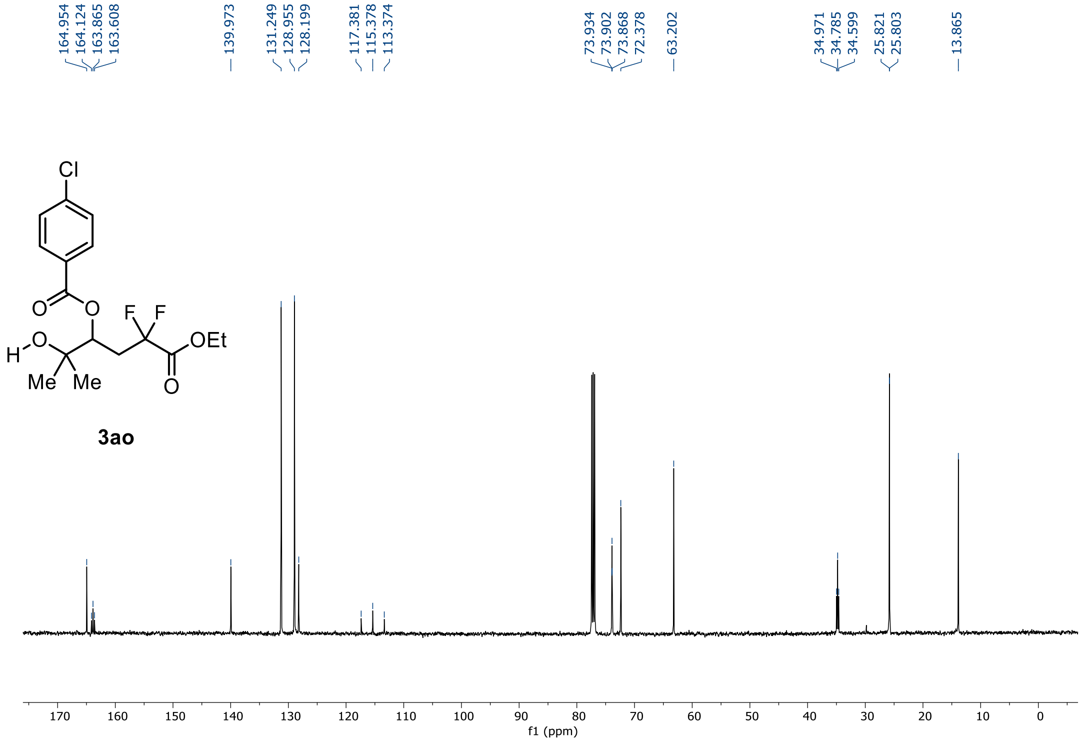

^{19}F NMR (470 MHz, $CDCl_3$**, 25 ºC) of (3ao)**

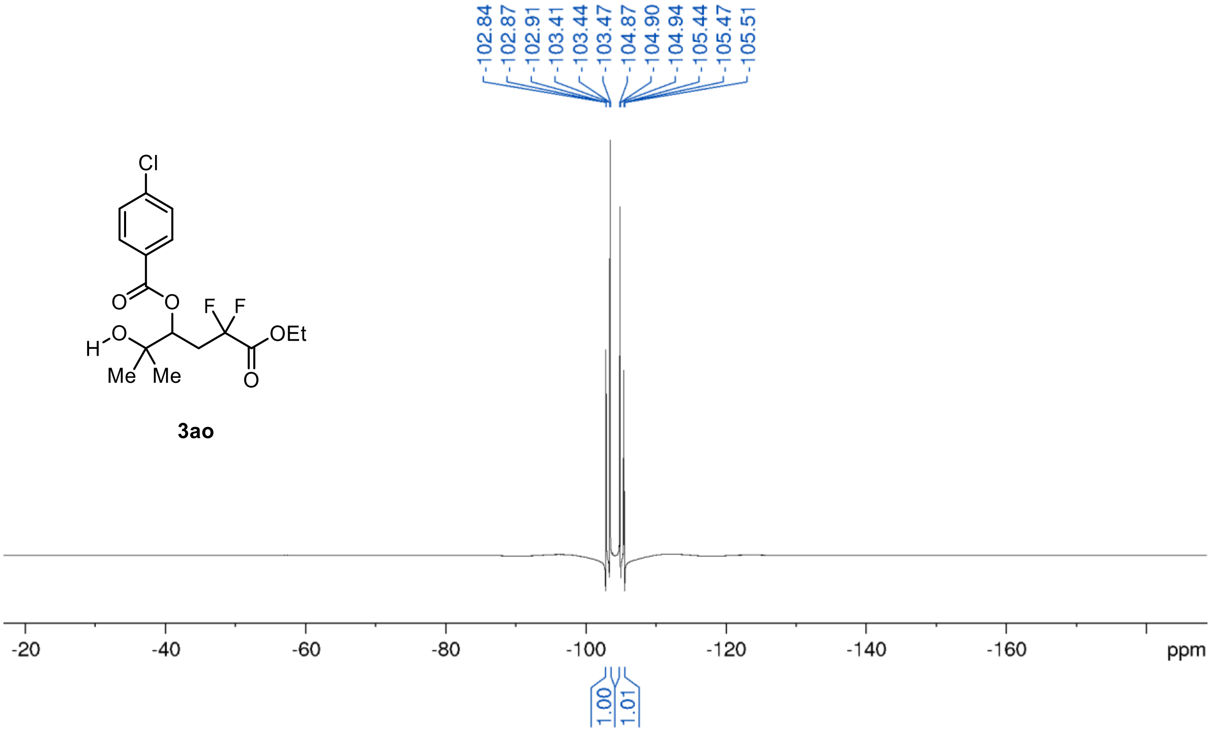

^{1}H NMR (500 MHz, $CDCl_3$, 25 ºC) of (3ap)

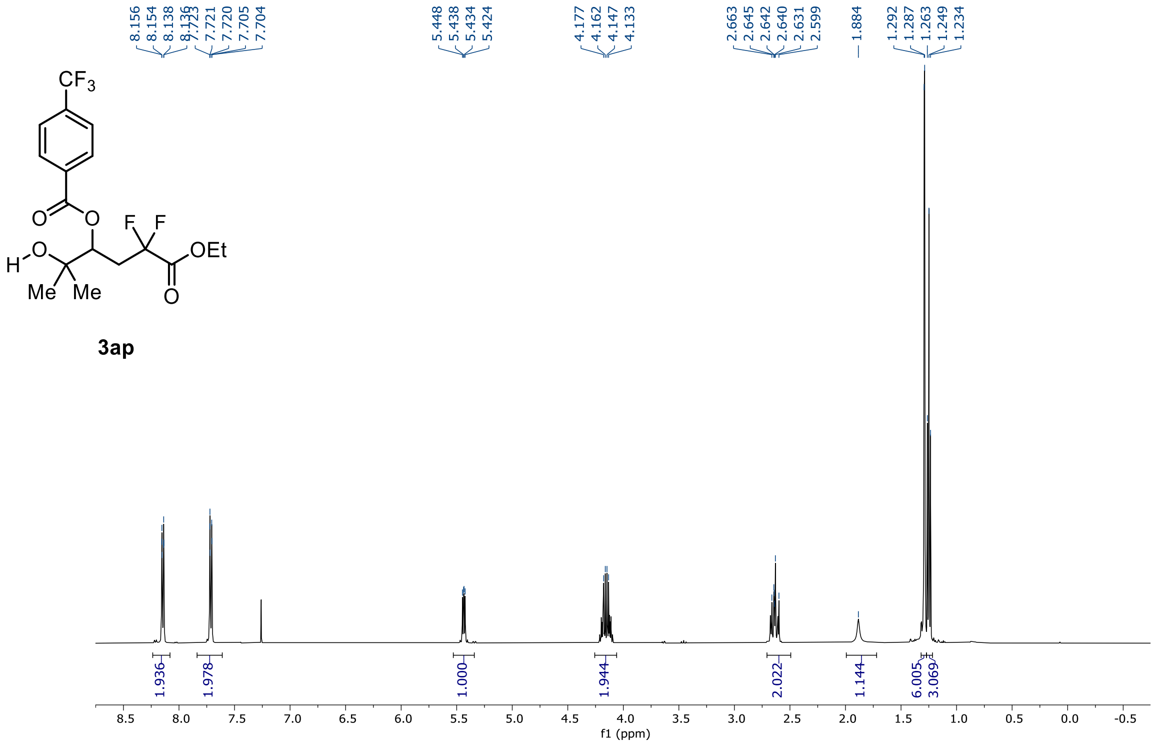

^{13}C NMR (126 MHz, $CDCl_3$, 25 ºC) of (3ap)

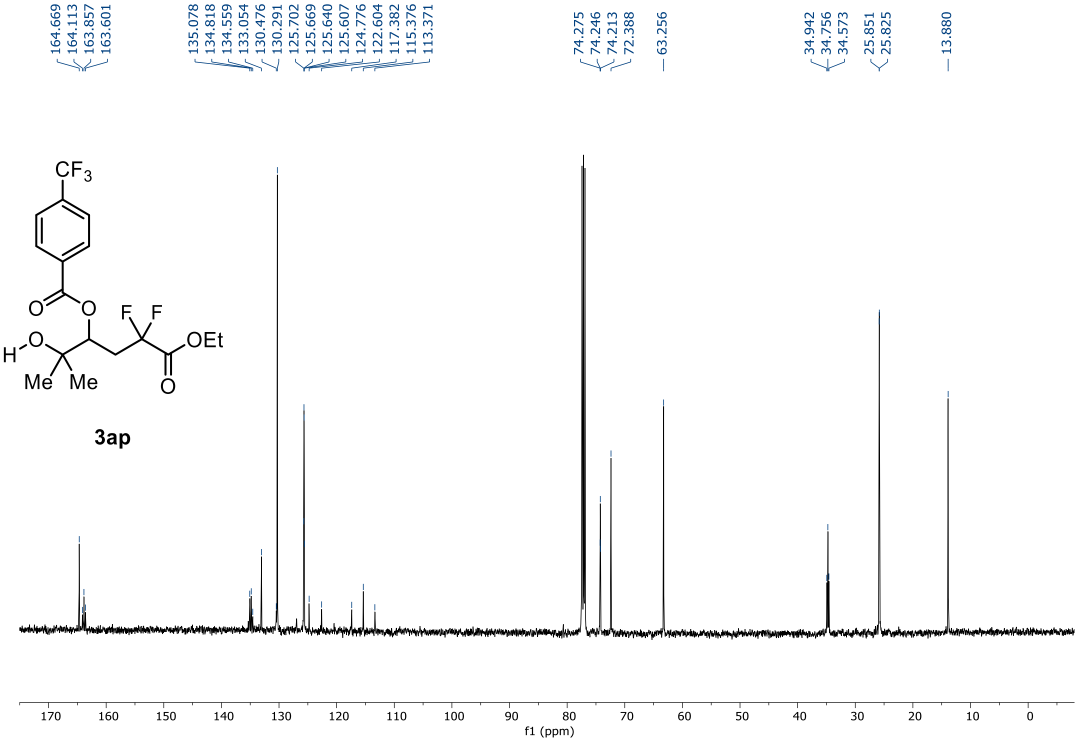

^{19}F NMR (470 MHz, $CDCl_3$**, 25 ºC) of (3ap)**

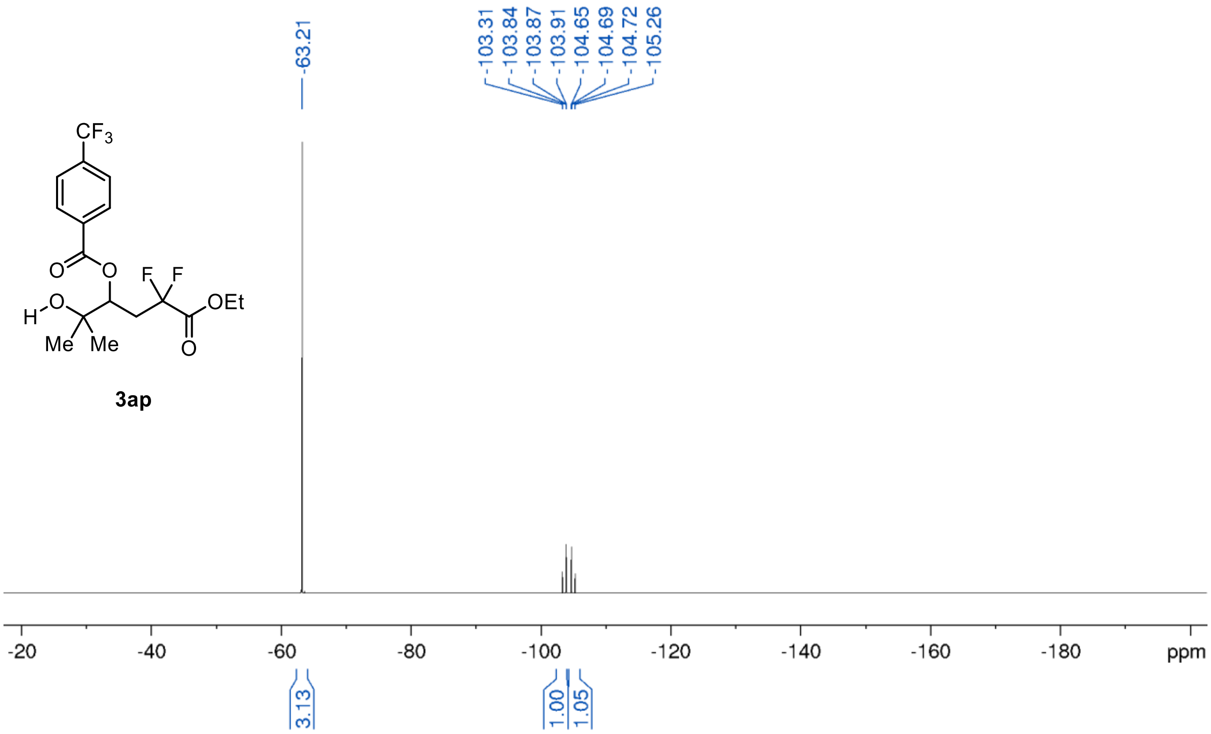

^{1}H NMR (500 MHz, CDCl$_3$**, 25 ºC) of (3aq)**

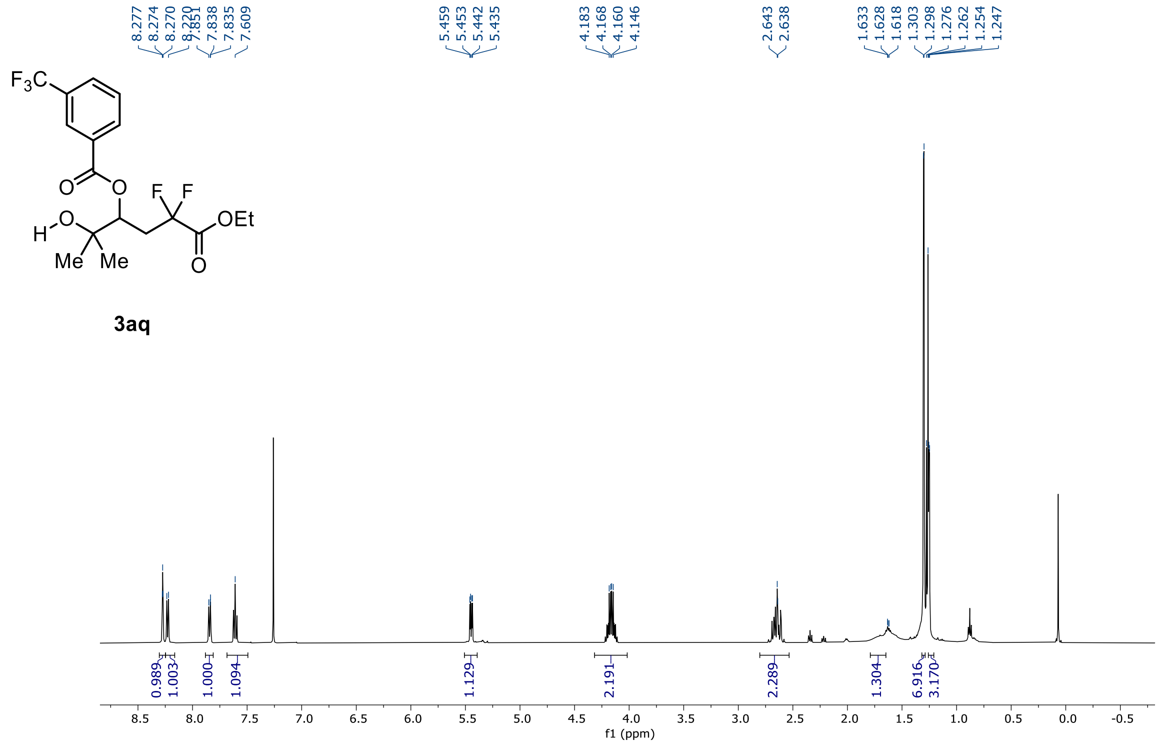

^{13}C NMR (126 MHz, CDCl$_3$**, 25 ºC) of (3aq)**

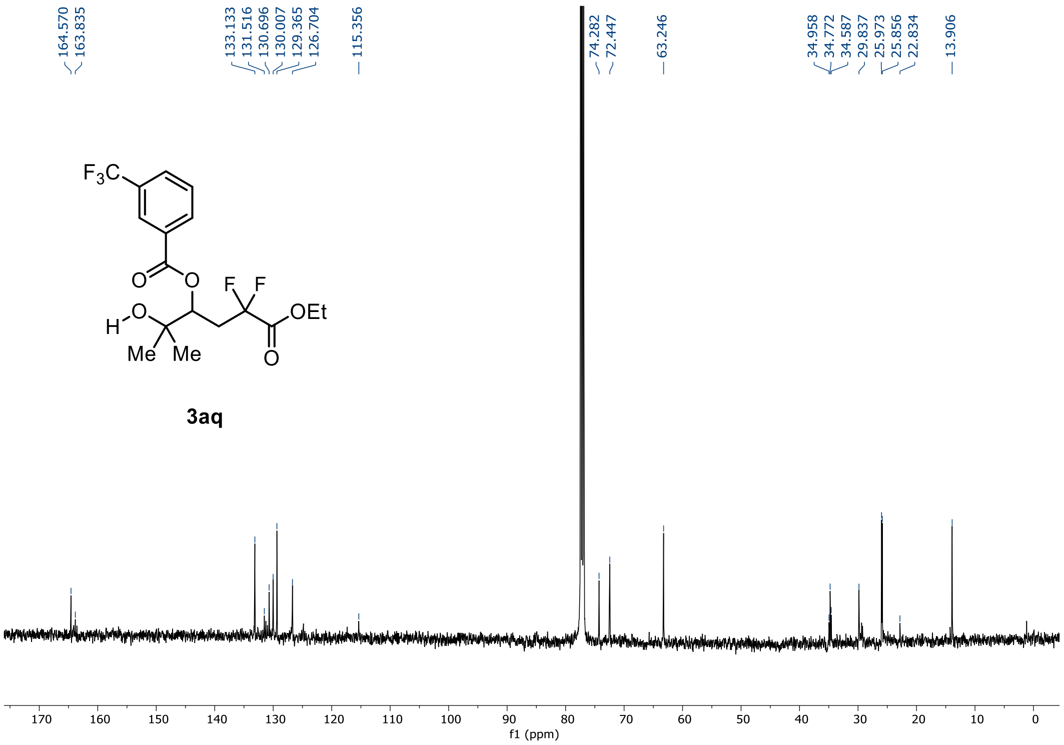

^{19}F NMR (470 MHz, CDCl$_3$**, 25 ºC) of (3aq)**

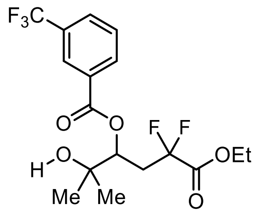

3aq

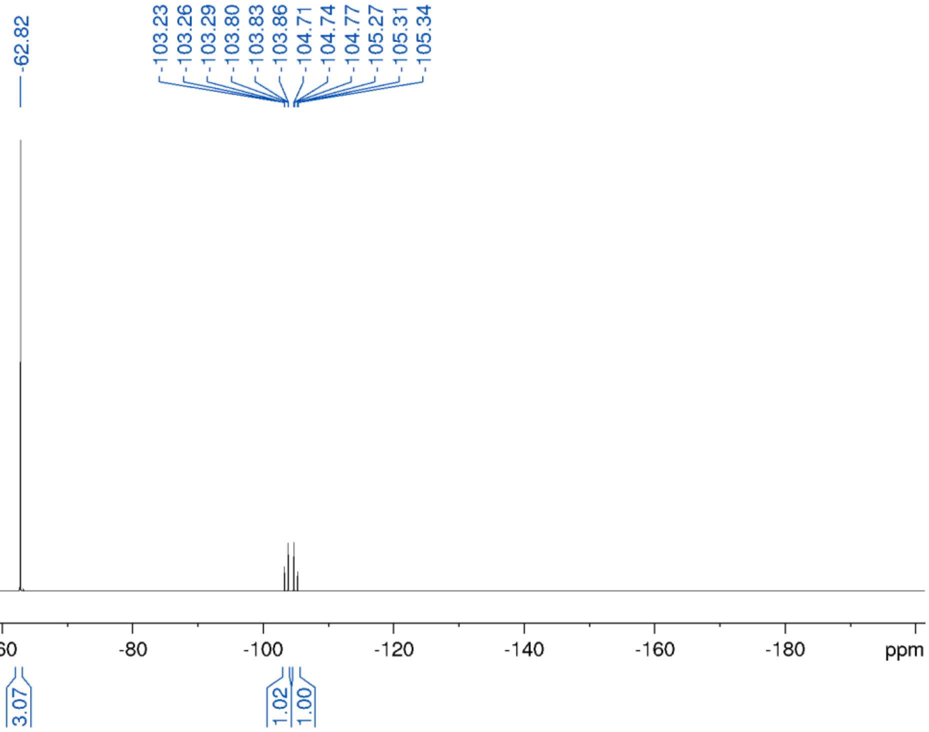

^{1}H NMR (500 MHz, $CDCl_3$, 25 ºC) of (3ar)

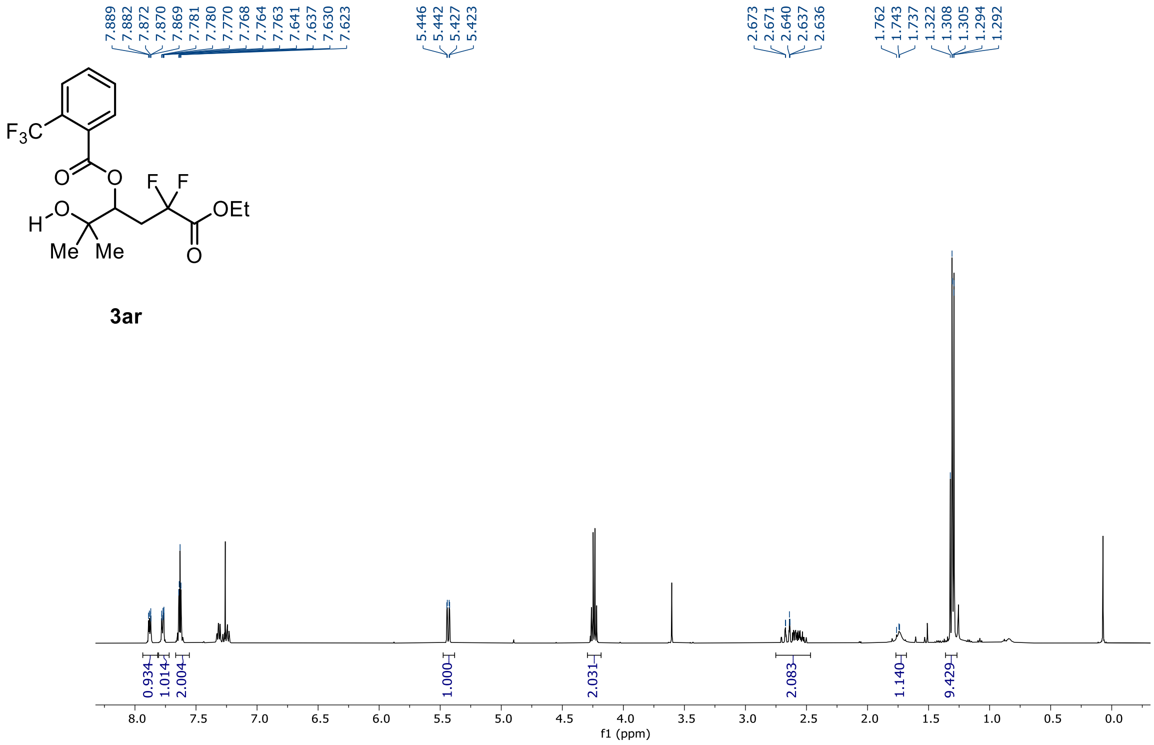

^{13}C NMR (126 MHz, $CDCl_3$, 25 ºC) of (3ar)

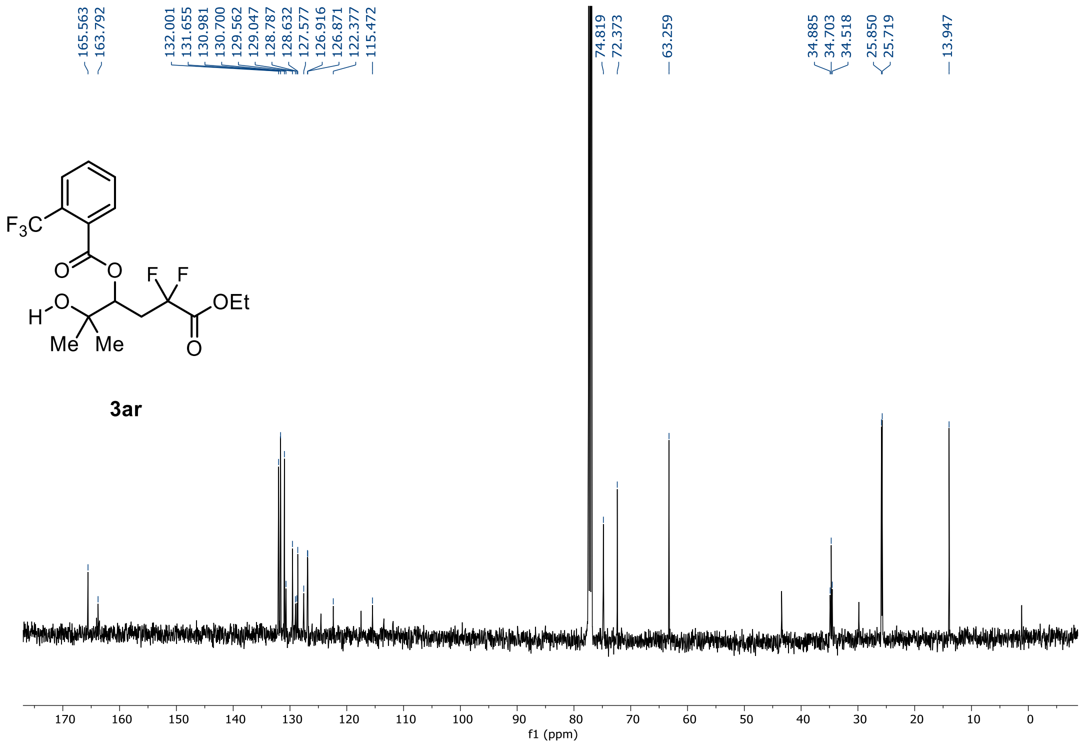

^{19}F NMR (470 MHz, CDCl$_3$**, 25 ºC) of (3ar)**

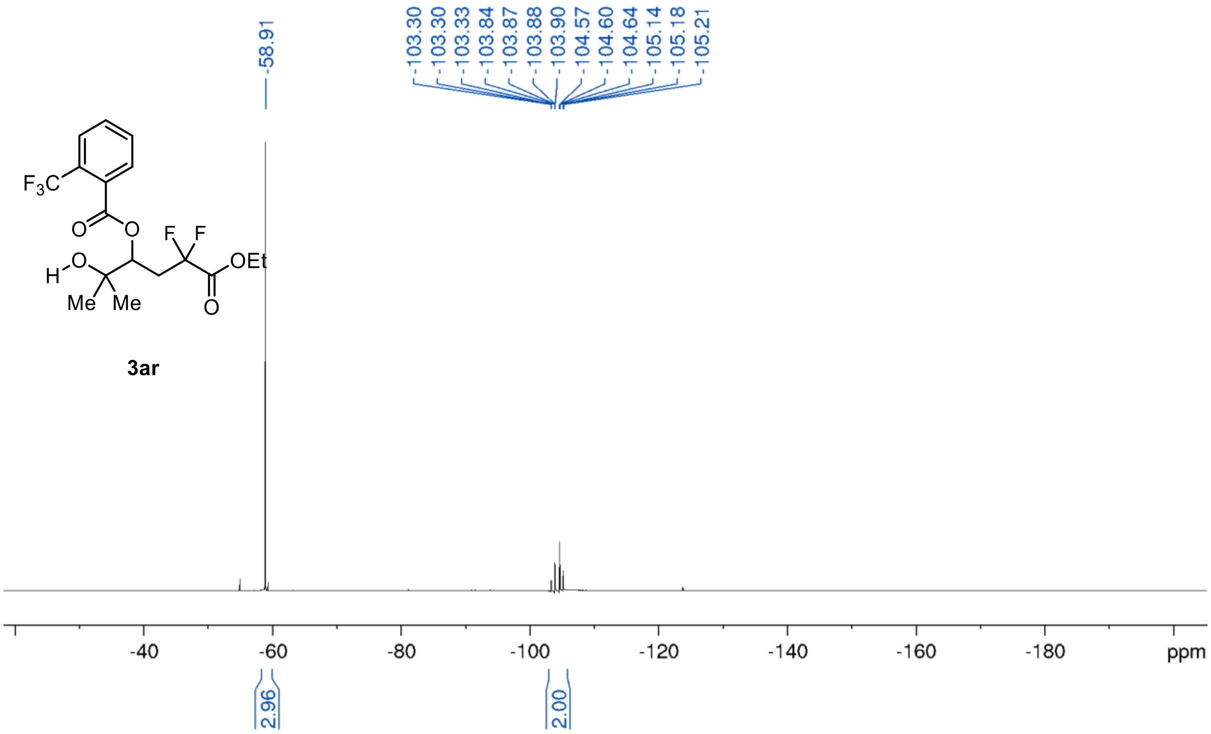

^{1}H NMR (500 MHz, $CDCl_3$, 25 ºC) of (3as)

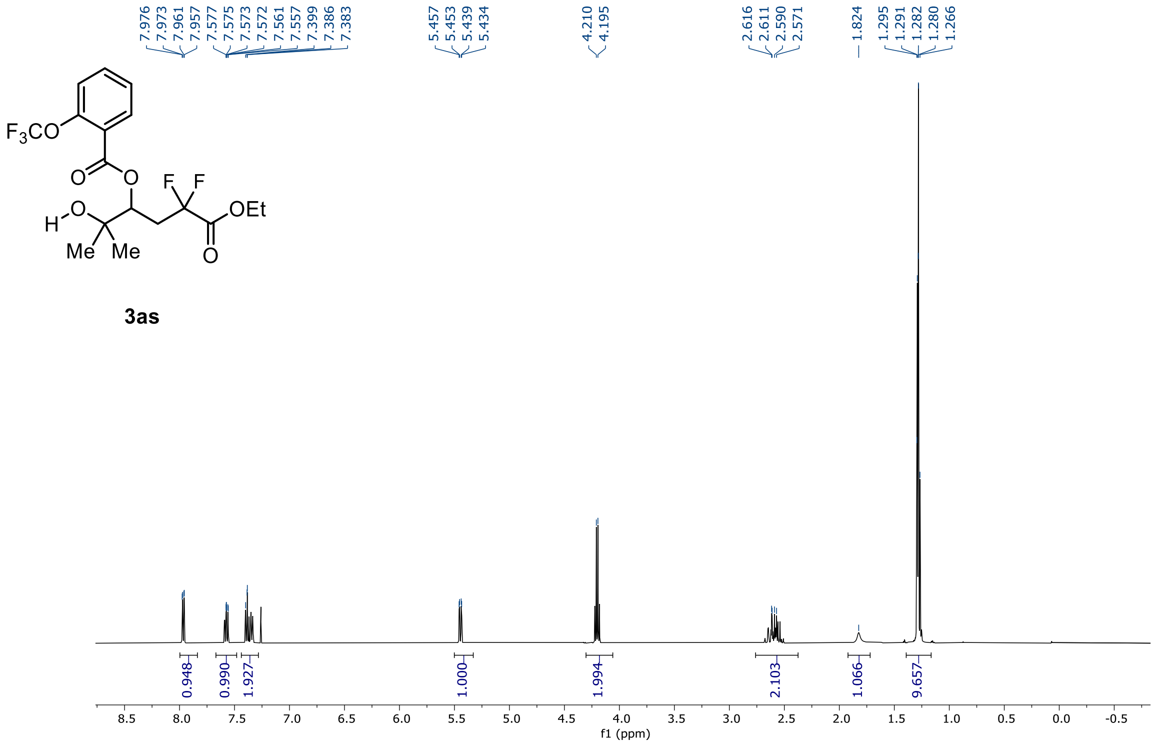

3as

^{13}C NMR (126 MHz, $CDCl_3$, 25 ºC) of (3as)

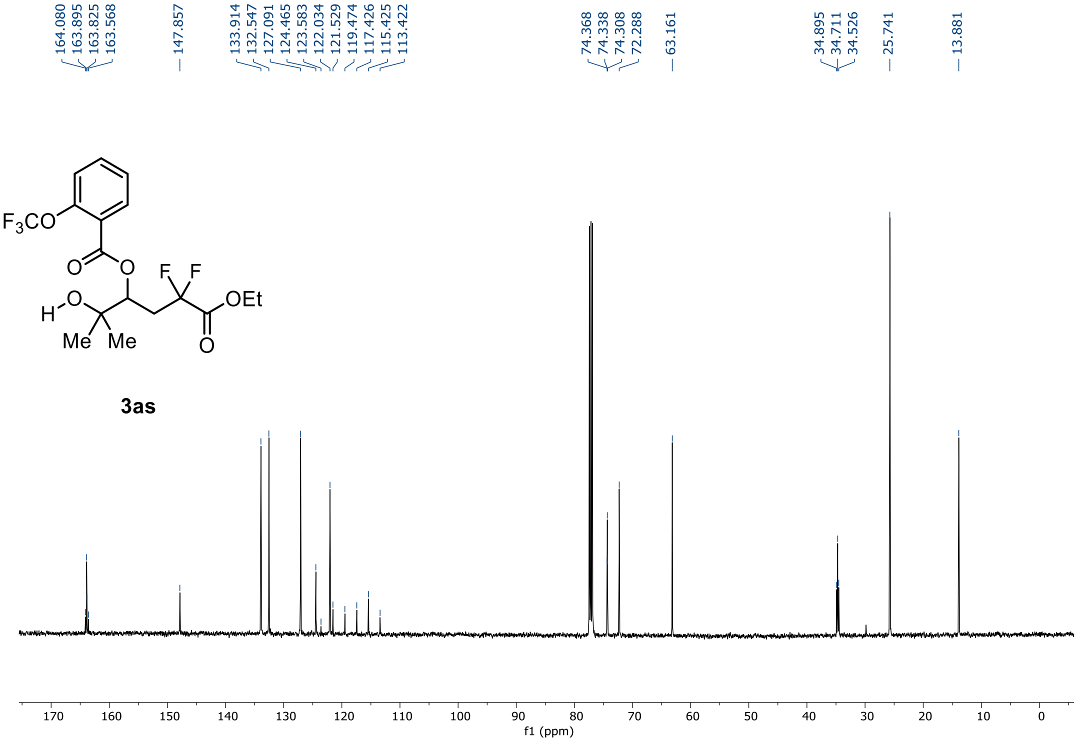

3as

^{19}F NMR (470 MHz, $CDCl_3$**, 25 ºC) of (3as)**

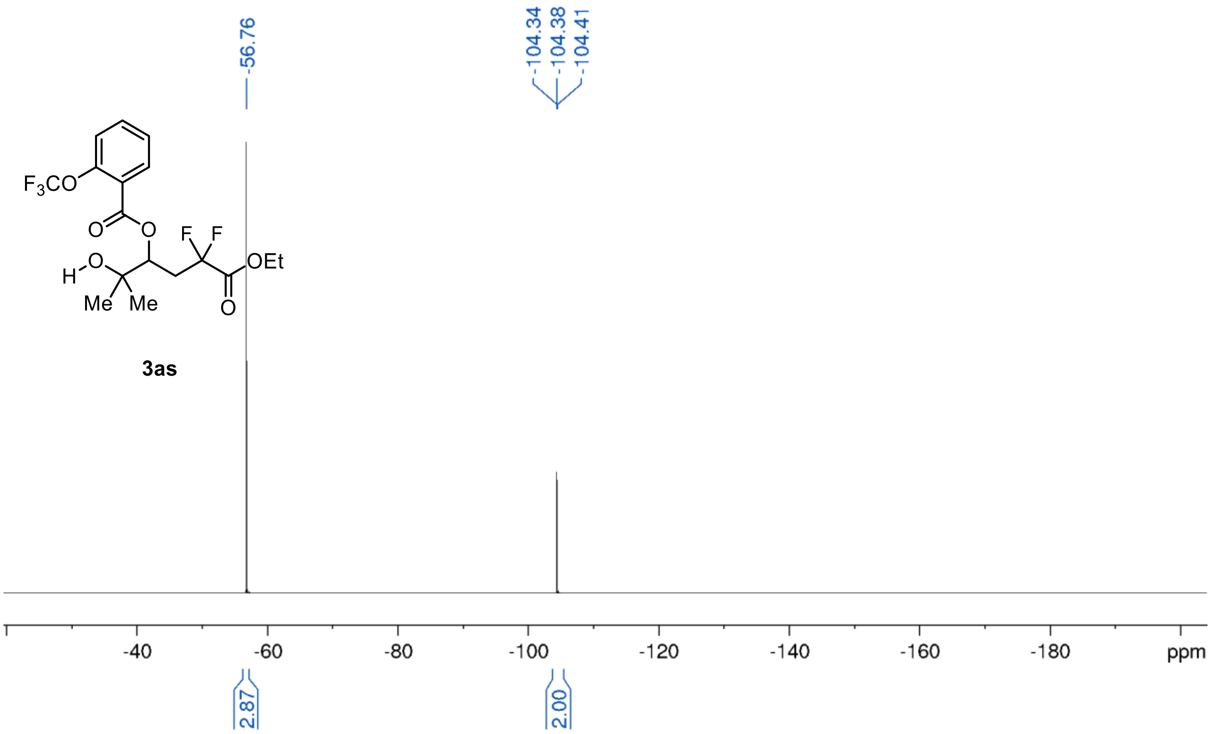

^{1}H NMR (500 MHz, $CDCl_3$, 25 ºC) of (3at)

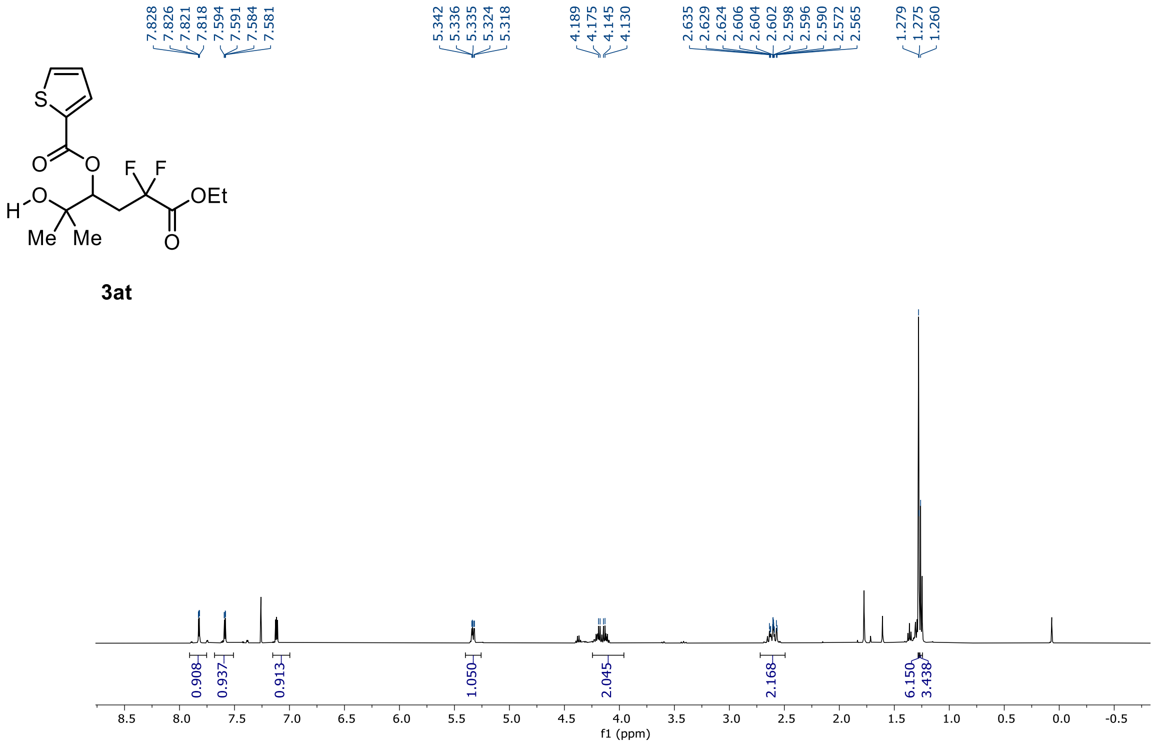

^{13}C NMR (126 MHz, $CDCl_3$, 25 ºC) of (3at)

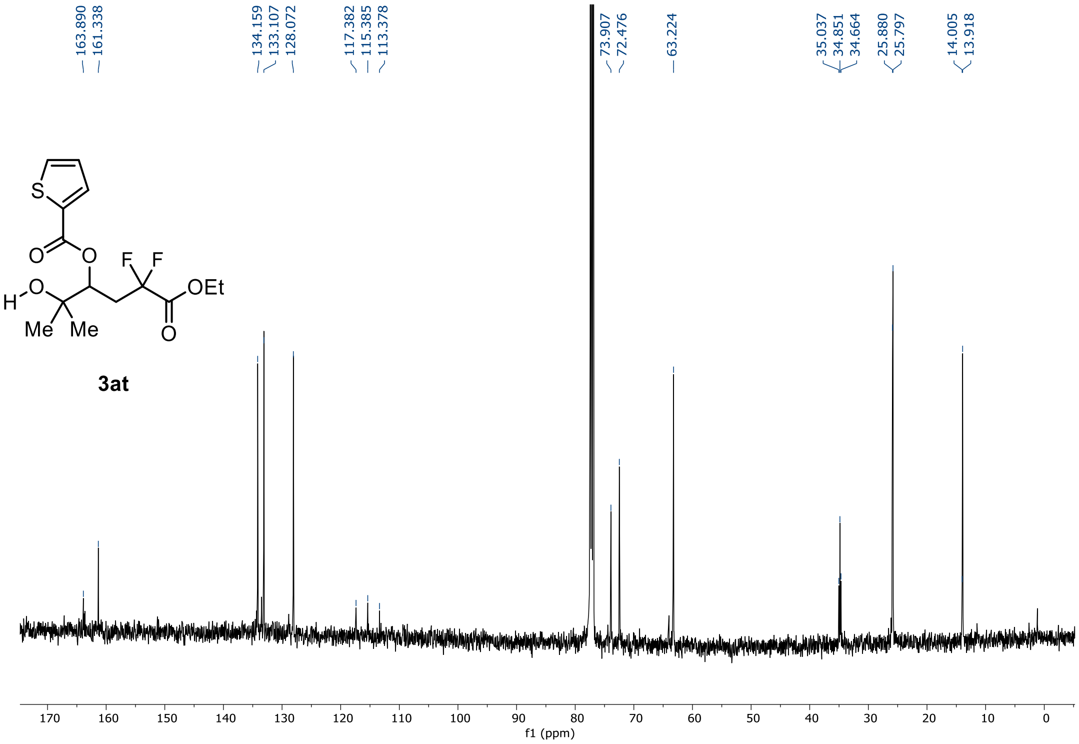

^{19}F NMR (470 MHz, $CDCl_3$**, 25 ºC) of (3at)**

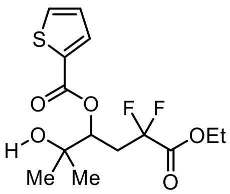

3at

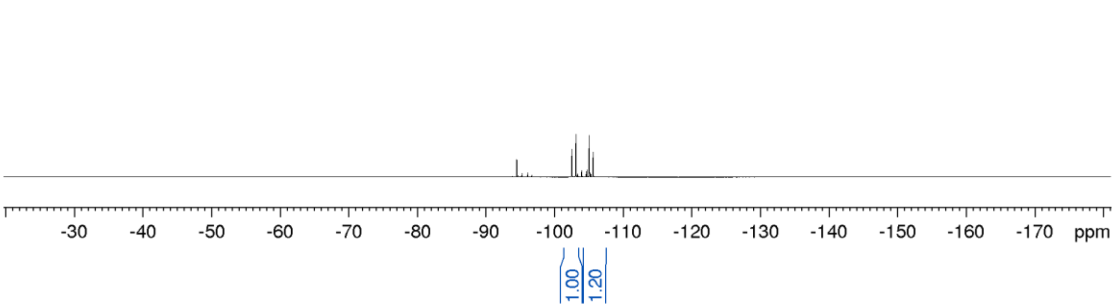

^{1}H NMR (500 MHz, $CDCl_3$, 25 ºC) of (3au)

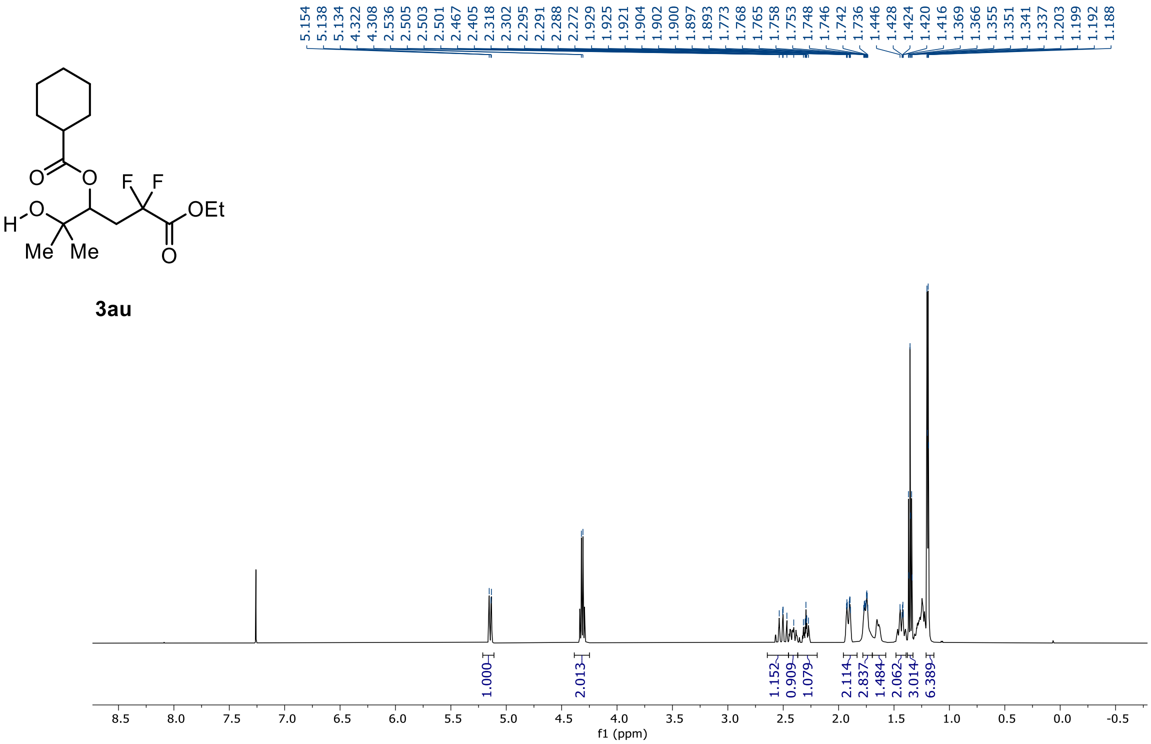

^{13}C NMR (126 MHz, $CDCl_3$, 25 ºC) of (3au)

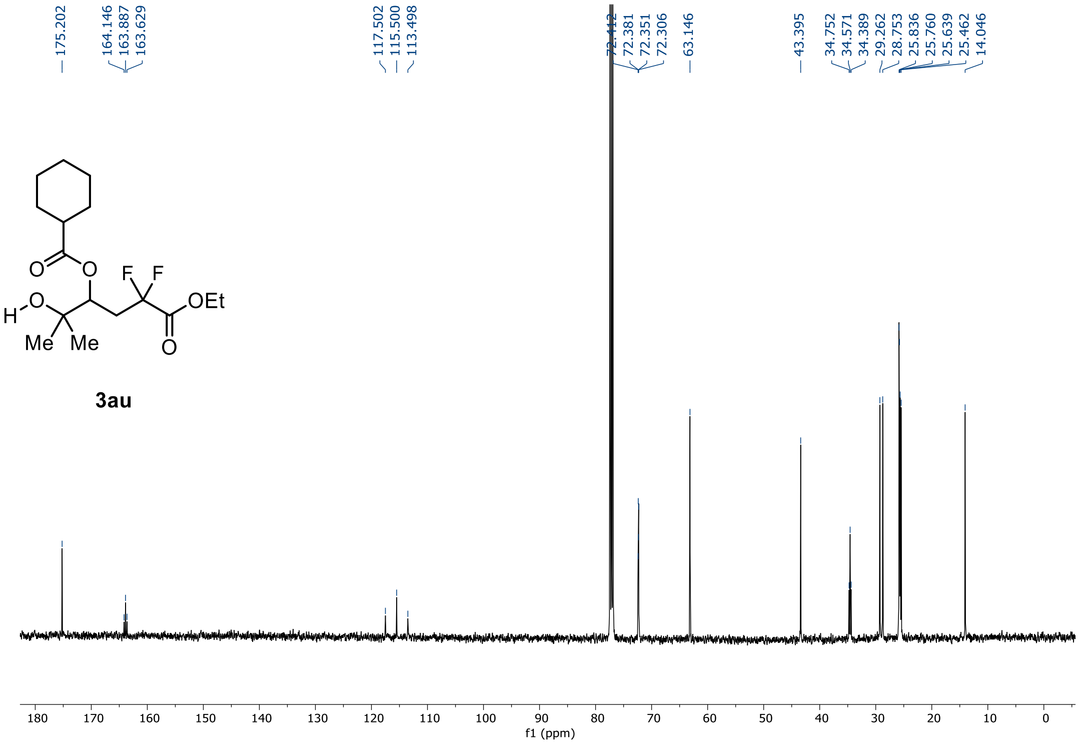

^{19}F NMR (470 MHz, CDCl$_3$**, 25 ºC) of (3au)**

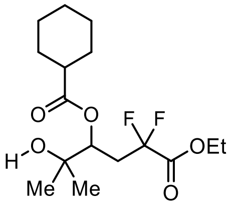

3au

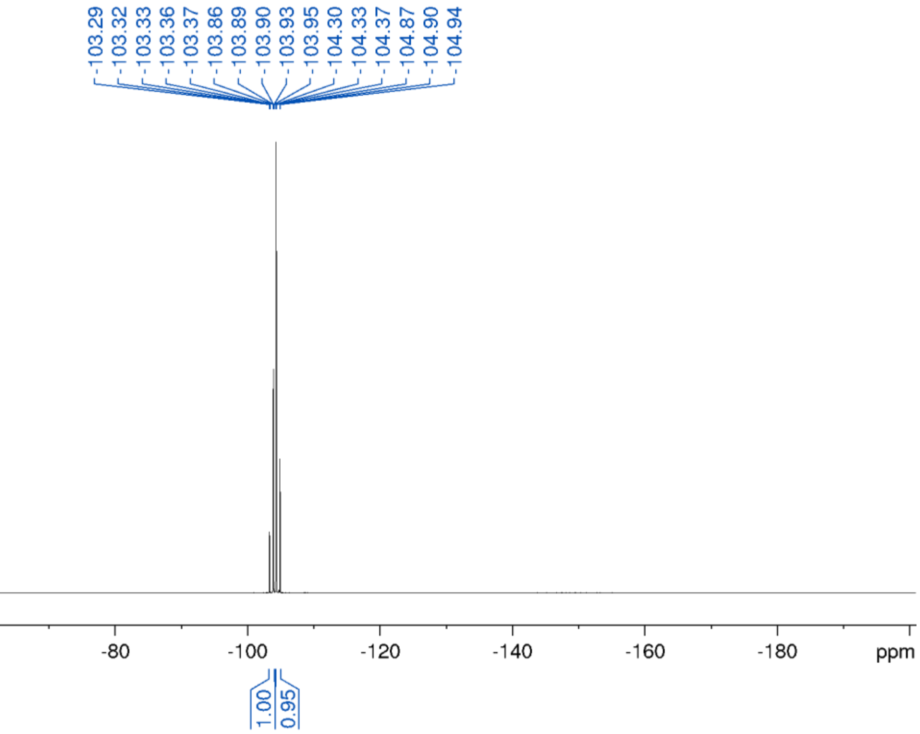

^{1}H NMR (500 MHz, $CDCl_3$, 25 ºC) of (3av)

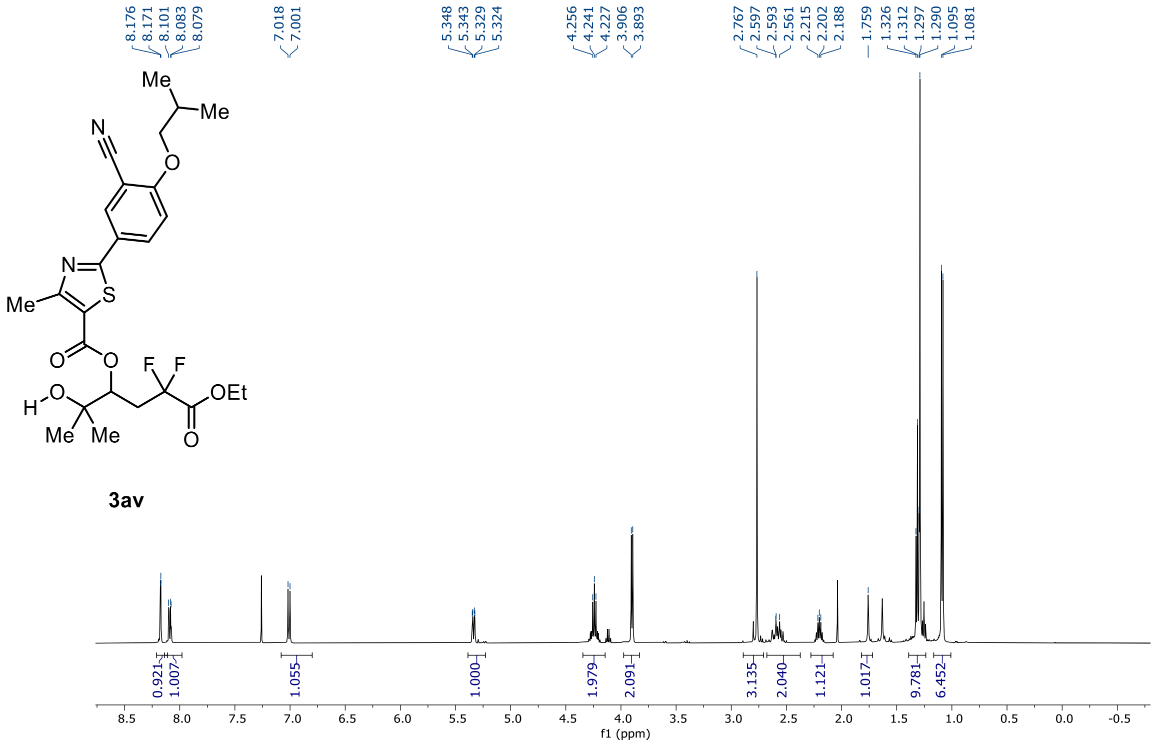

^{13}C NMR (126 MHz, $CDCl_3$, 25 ºC) of (3av)

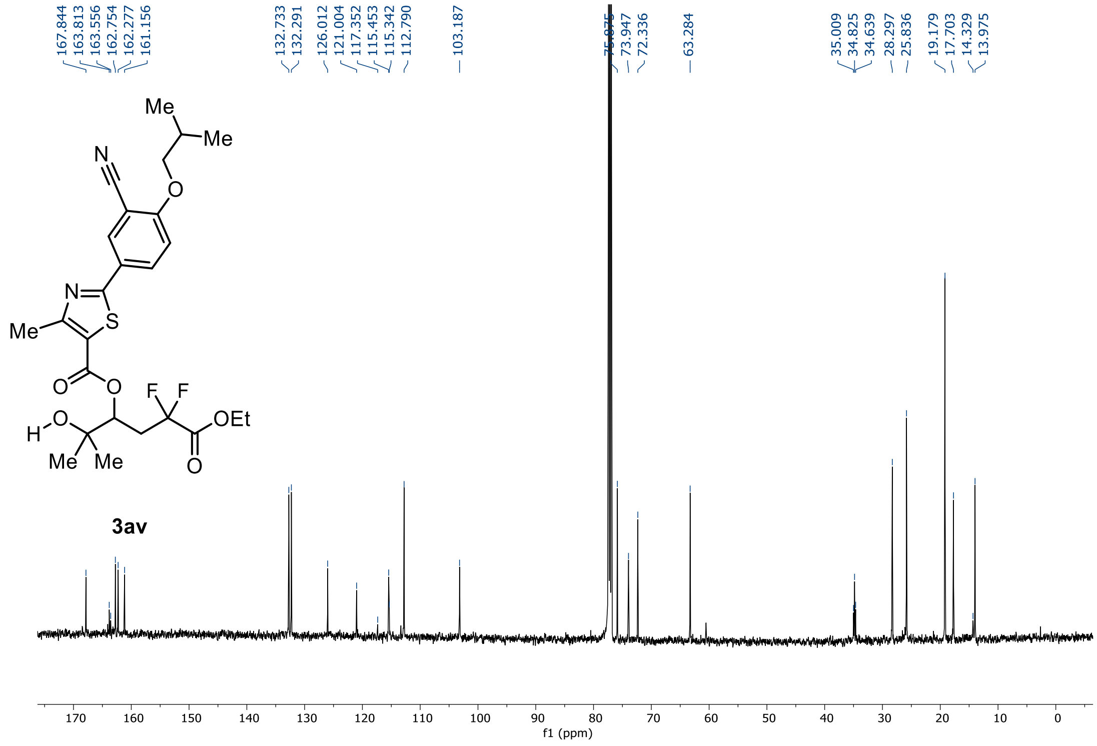

^{19}F NMR (470 MHz, CDCl$_3$, 25 ºC) of (3av)

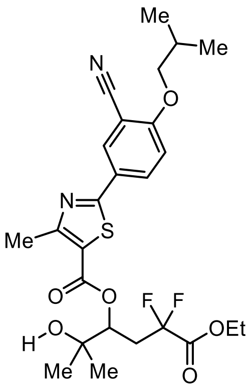

3av

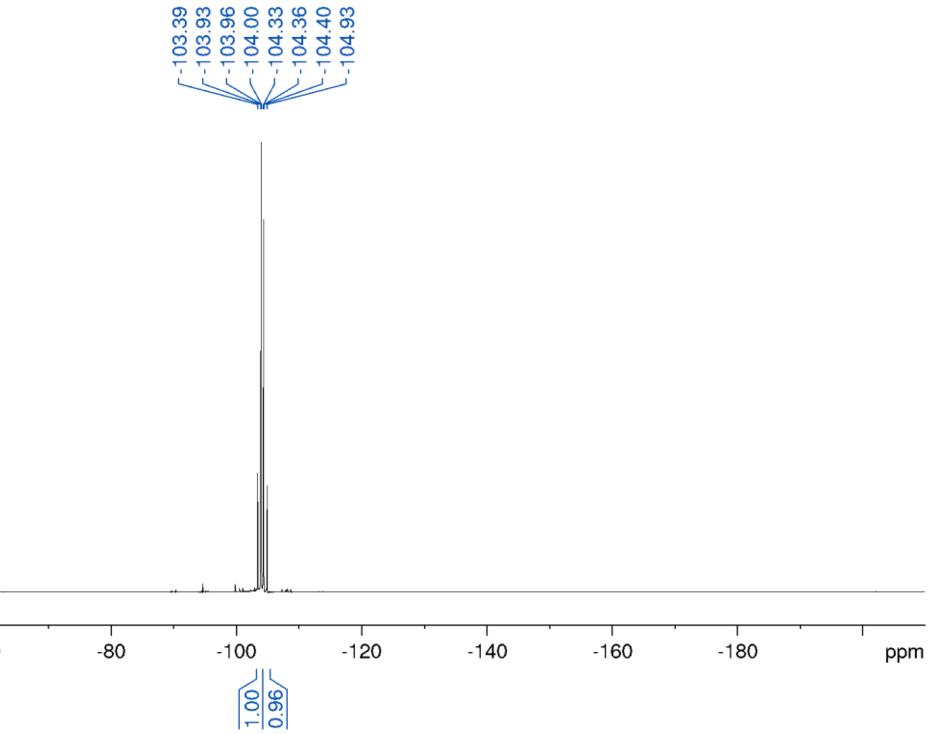

^{1}H NMR (500 MHz, CDCl$_3$**, 25 ºC) of (3aw)**

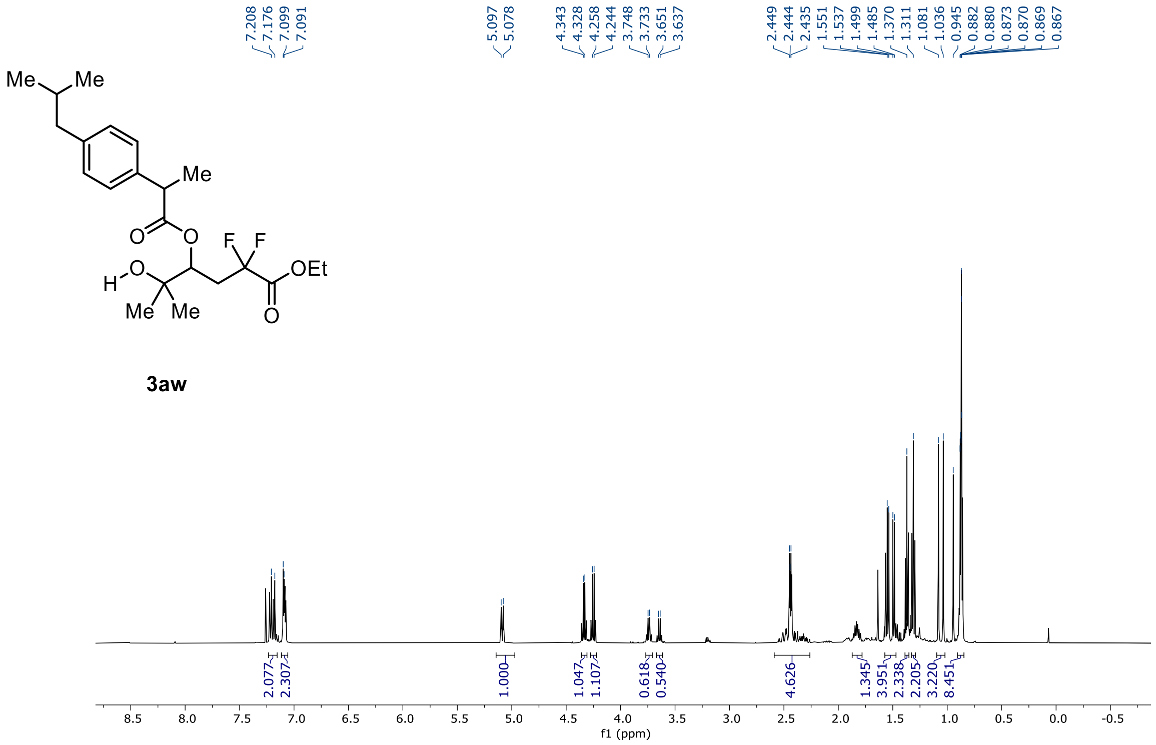

^{13}C NMR (126 MHz, CDCl$_3$**, 25 ºC) of (3aw)**

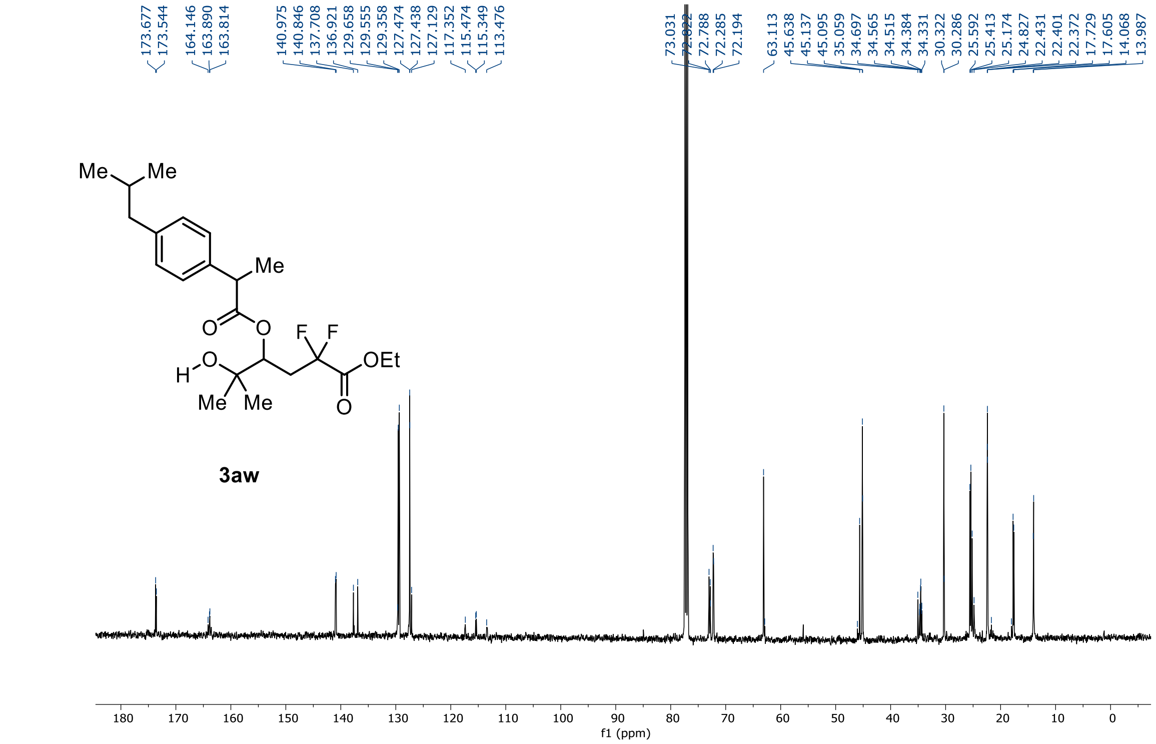

^{19}F NMR (470 MHz, CDCl3**, 25 ºC) of (3aw)**

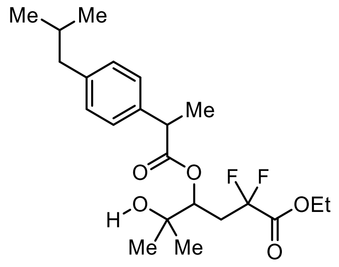

3aw

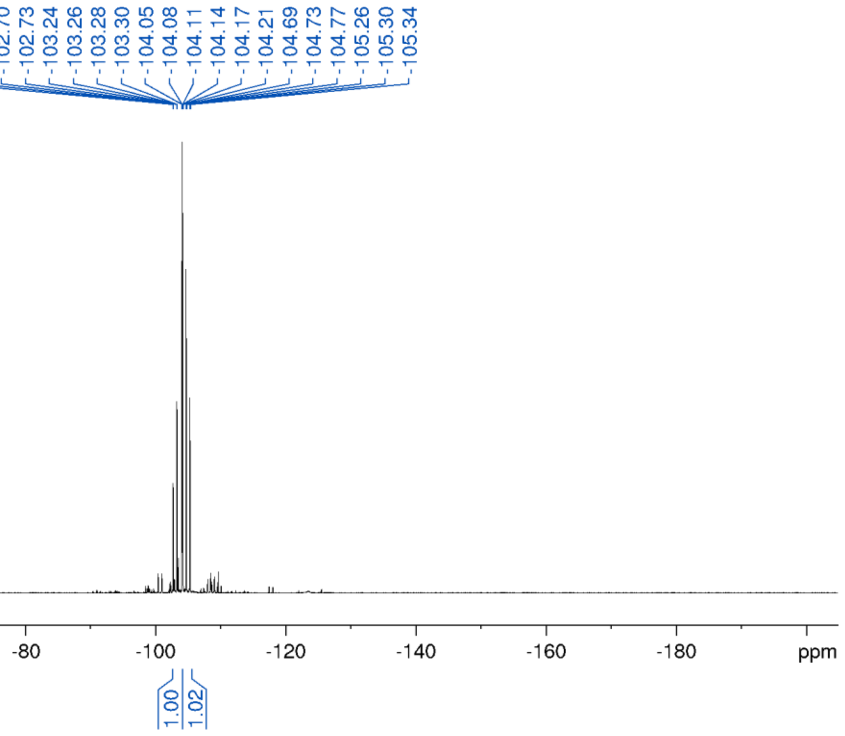

^{1}H NMR (500 MHz, CDCl$_3$, **25 ºC) of (3ax)**

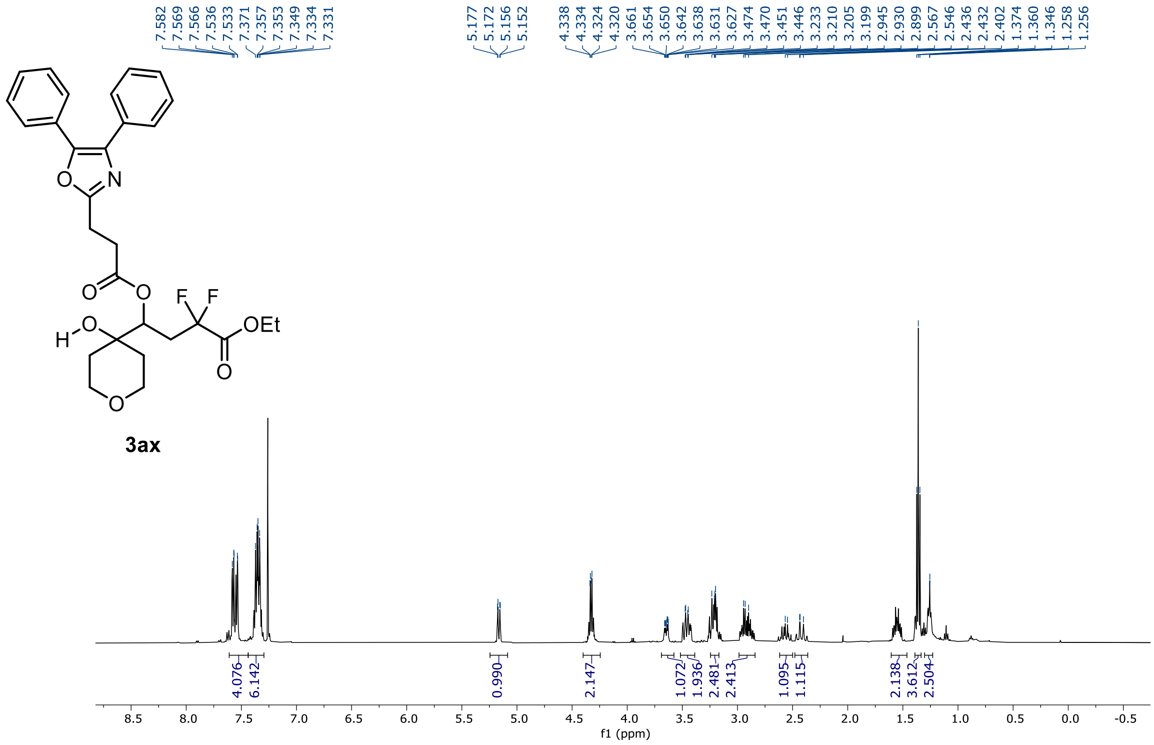

^{13}C NMR (126 MHz, CDCl$_3$, **25 ºC) of (3ax)**

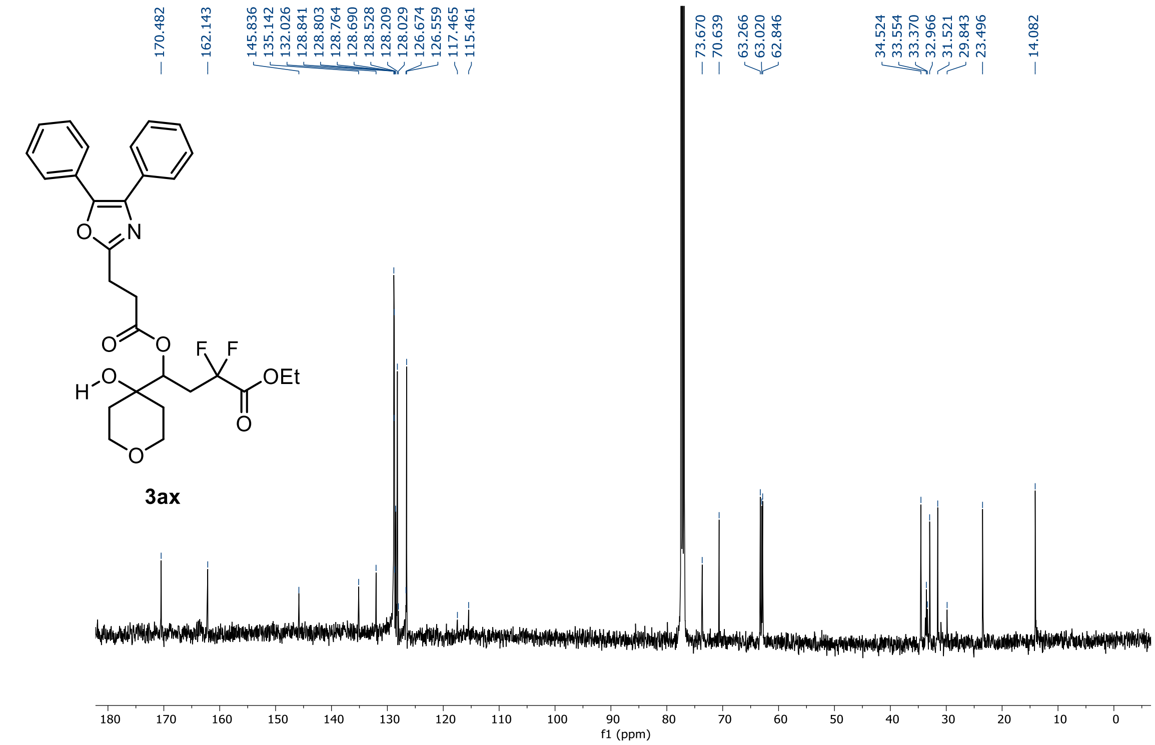

^{19}F NMR (470 MHz, CDCl$_3$, 25 ºC) of (3ax)

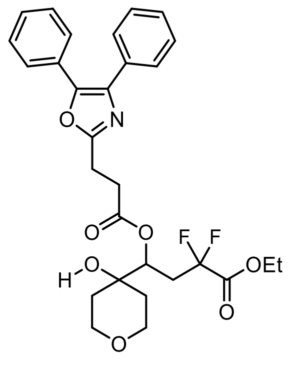

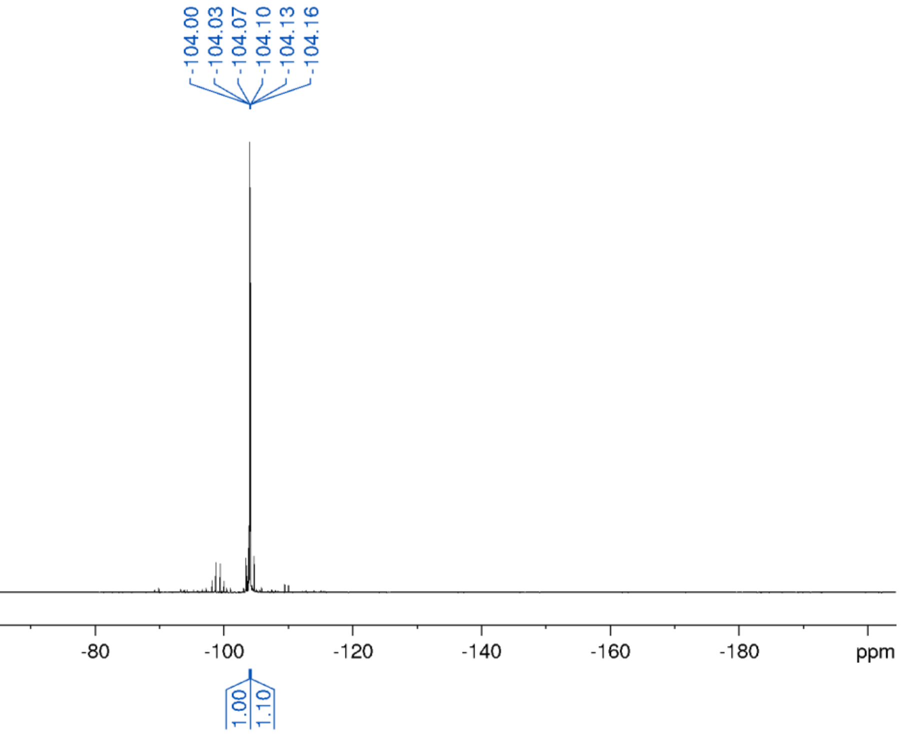

^{1}H NMR (500 MHz, CDCl$_3$**, 25 ºC) of (3ay)**

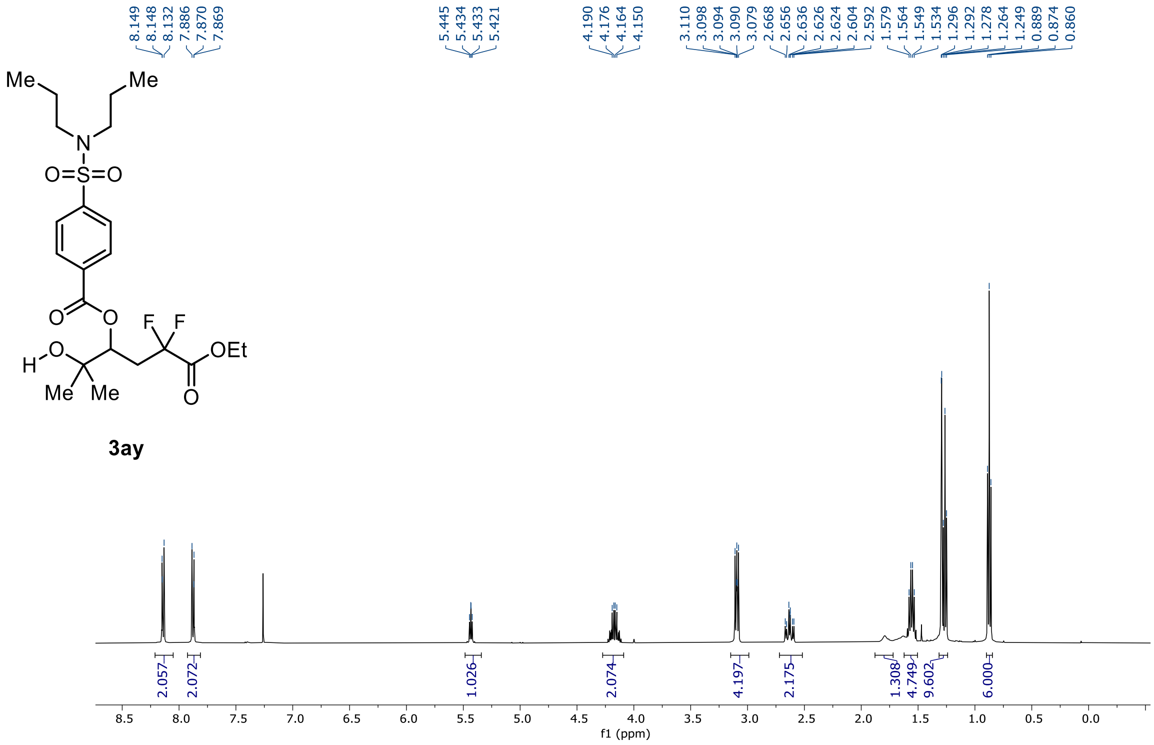

^{13}C NMR (126 MHz, CDCl$_3$**, 25 ºC) of (3ay)**

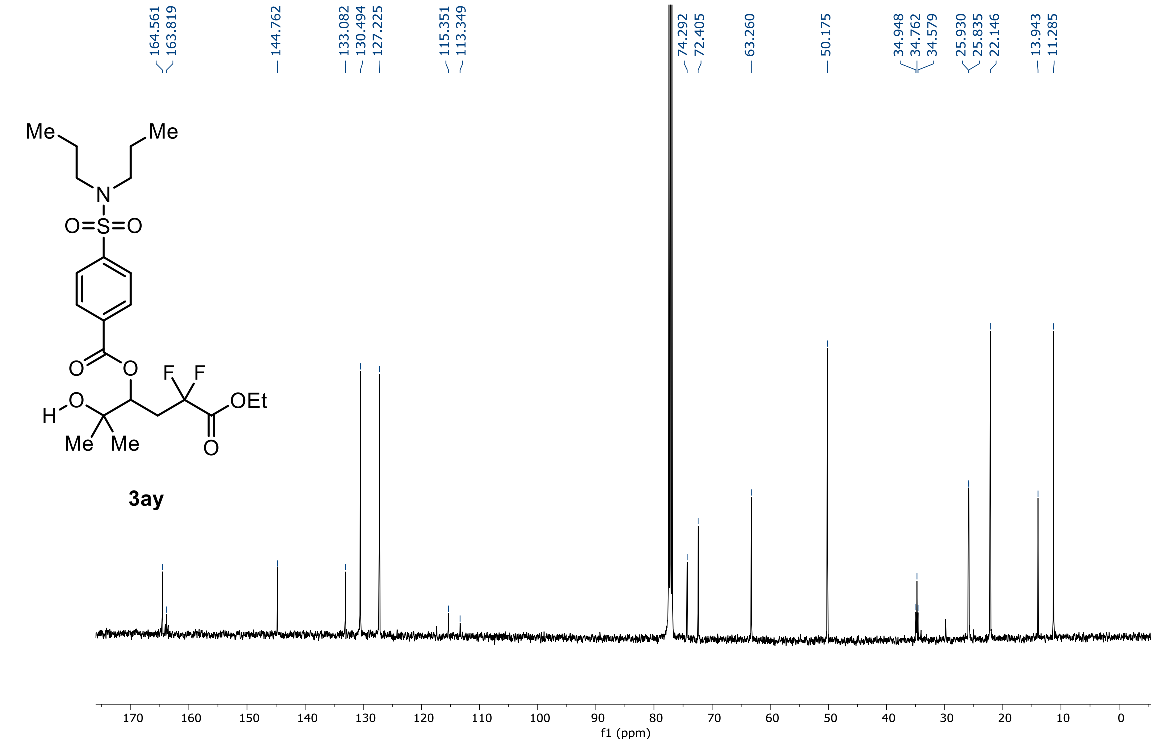

^{19}F NMR (470 MHz, CDCl$_3$**, 25 ºC) of (3ay)**

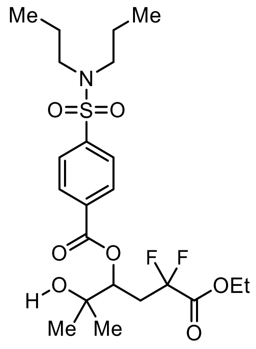

3ay

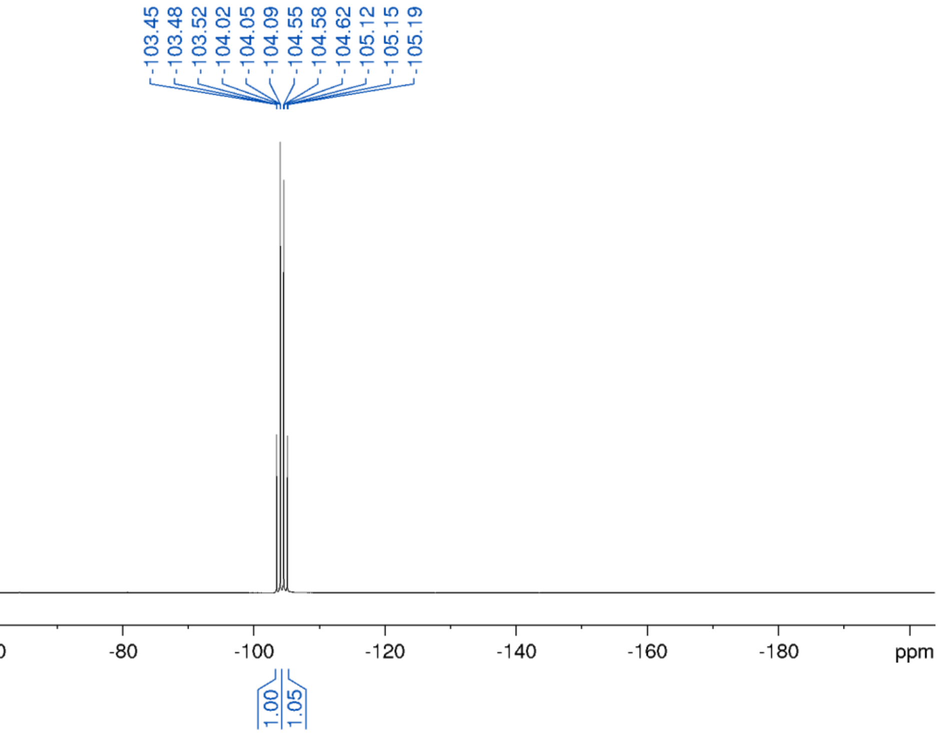

^{1}H NMR (500 MHz, CDCl$_3$, 25 ºC) of (3az)

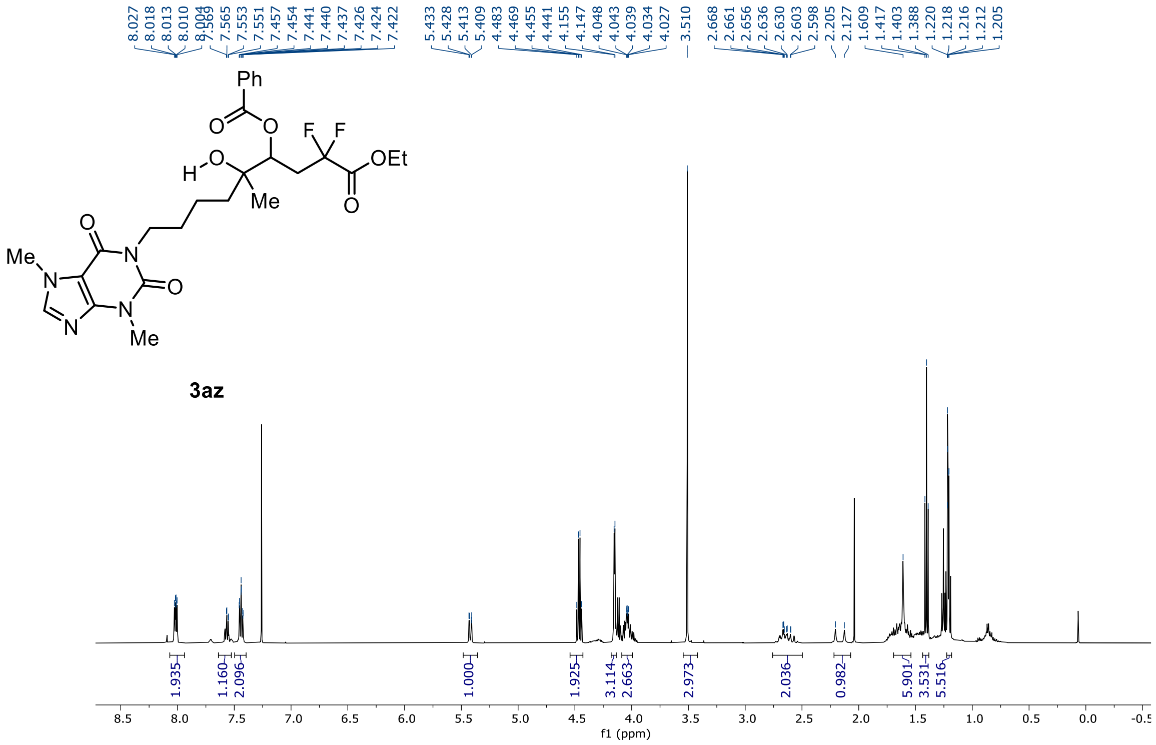

^{13}C NMR (126 MHz, CDCl$_3$, 25 ºC) of (3az)

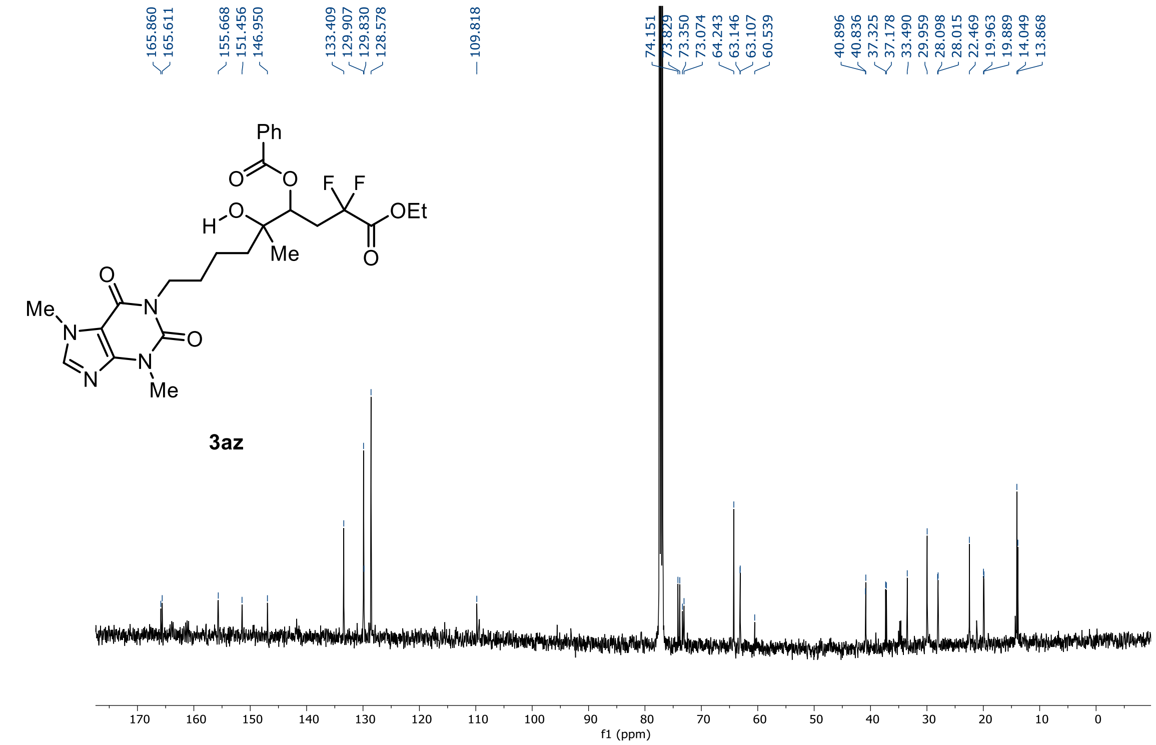

^{19}F NMR (470 MHz, CDCl$_3$**, 25 ºC) of (3az)**

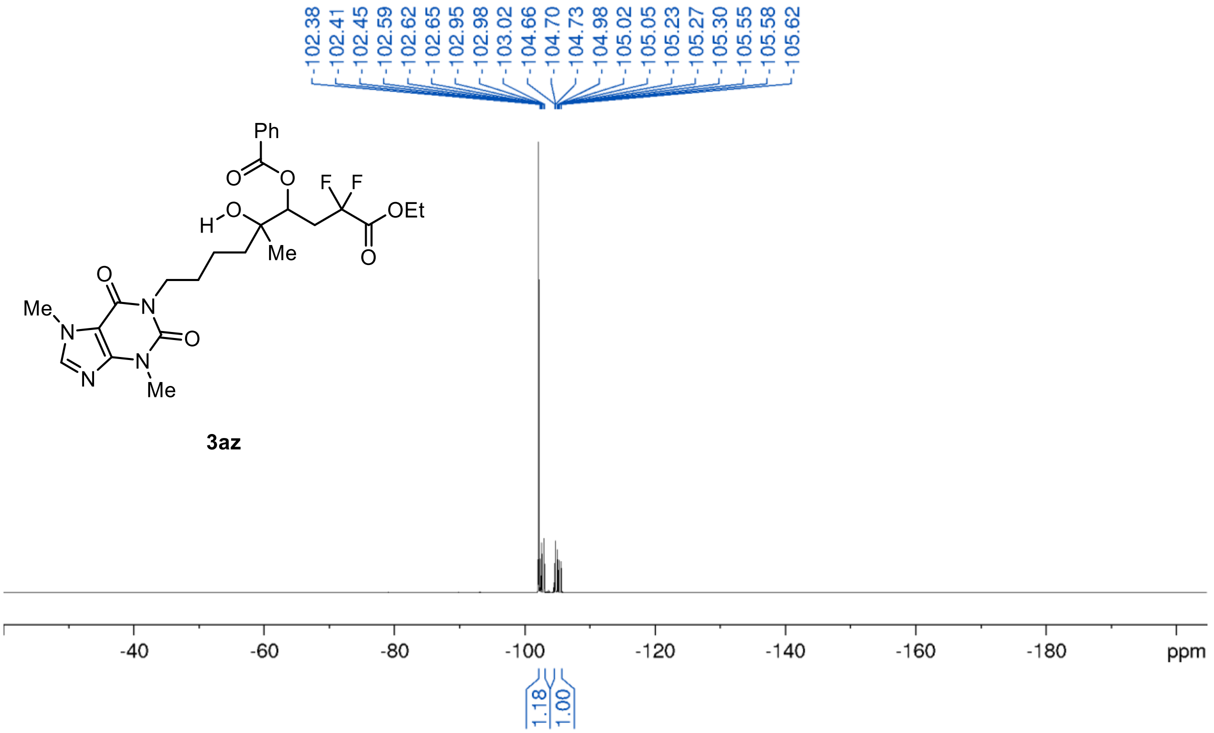

^{1}H NMR (500 MHz, CDCl$_3$, **25 ºC) of (3aaa)**

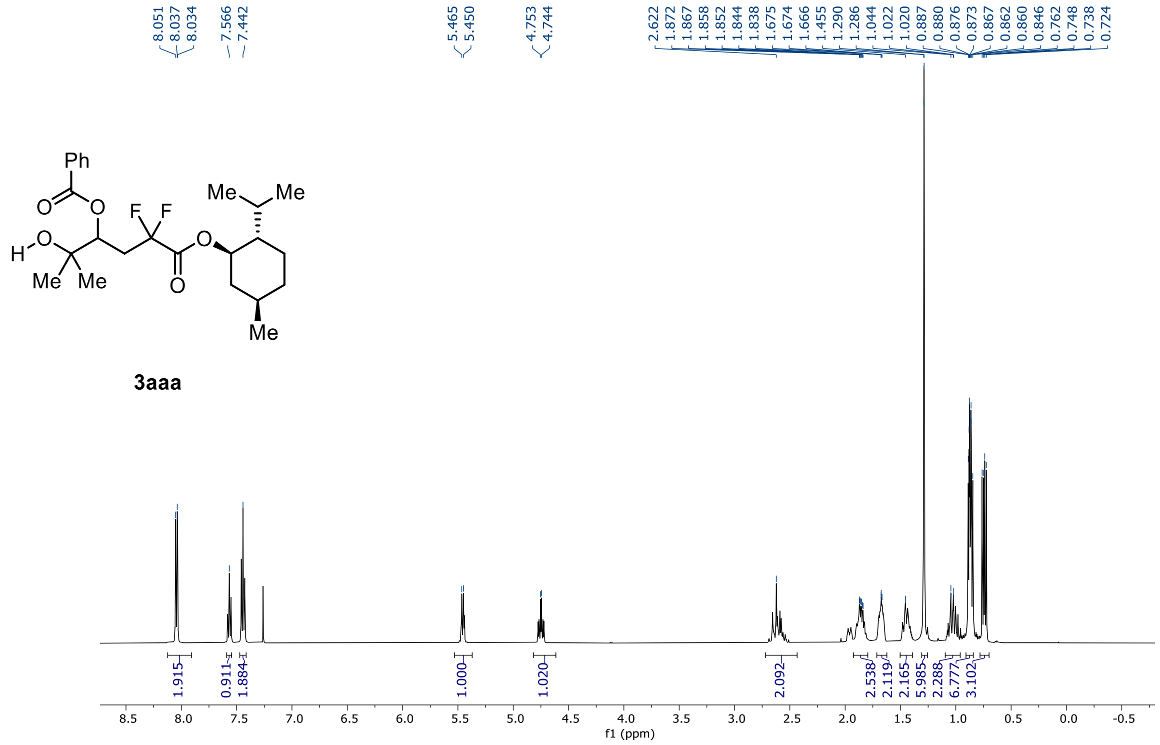

^{13}C NMR (126 MHz, CDCl$_3$, **25 ºC) of (3aaa)**

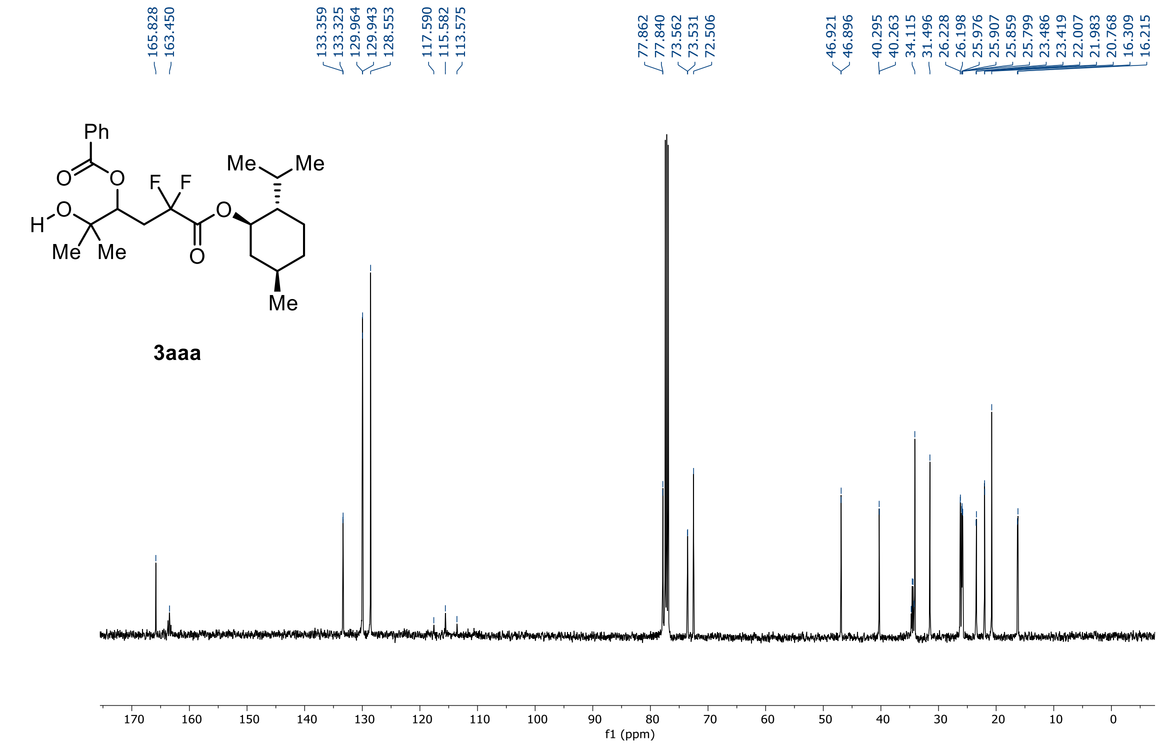

^{19}F NMR (470 MHz, CDCl3**, 25 ºC) of (3aaa)**

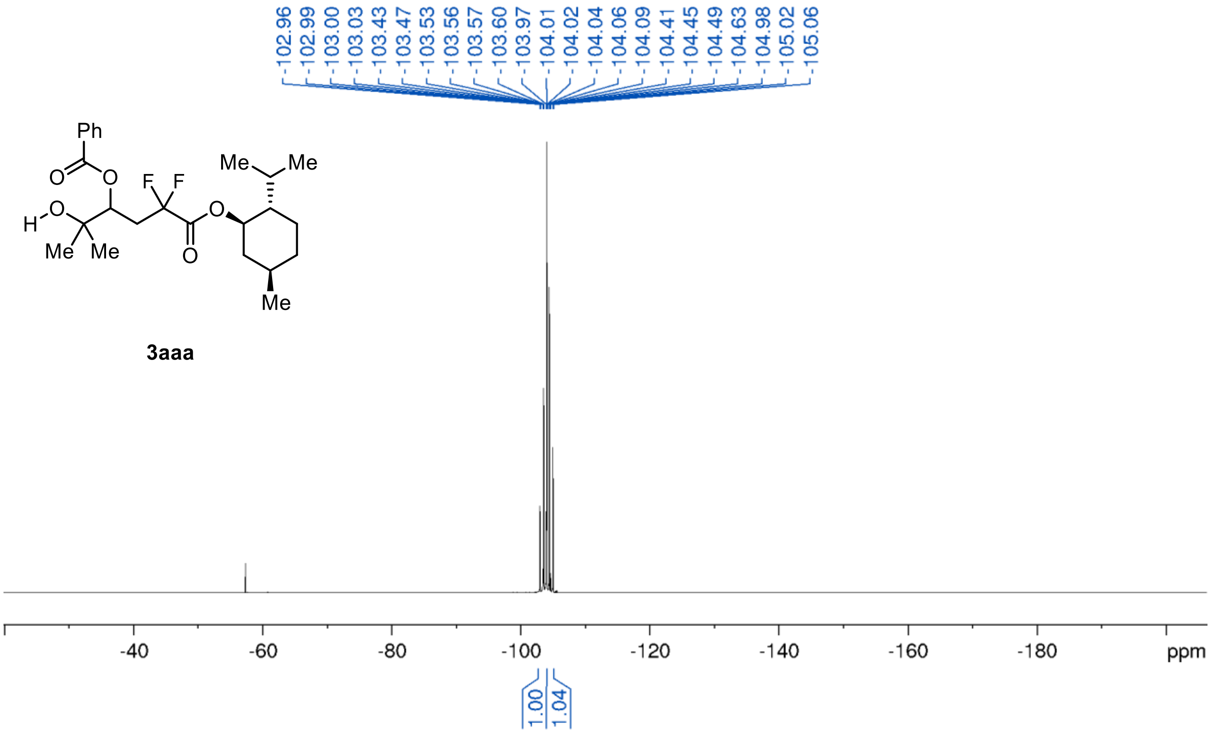

^{1}H NMR (500 MHz, $CDCl_3$, 25 ºC) of (3aab)

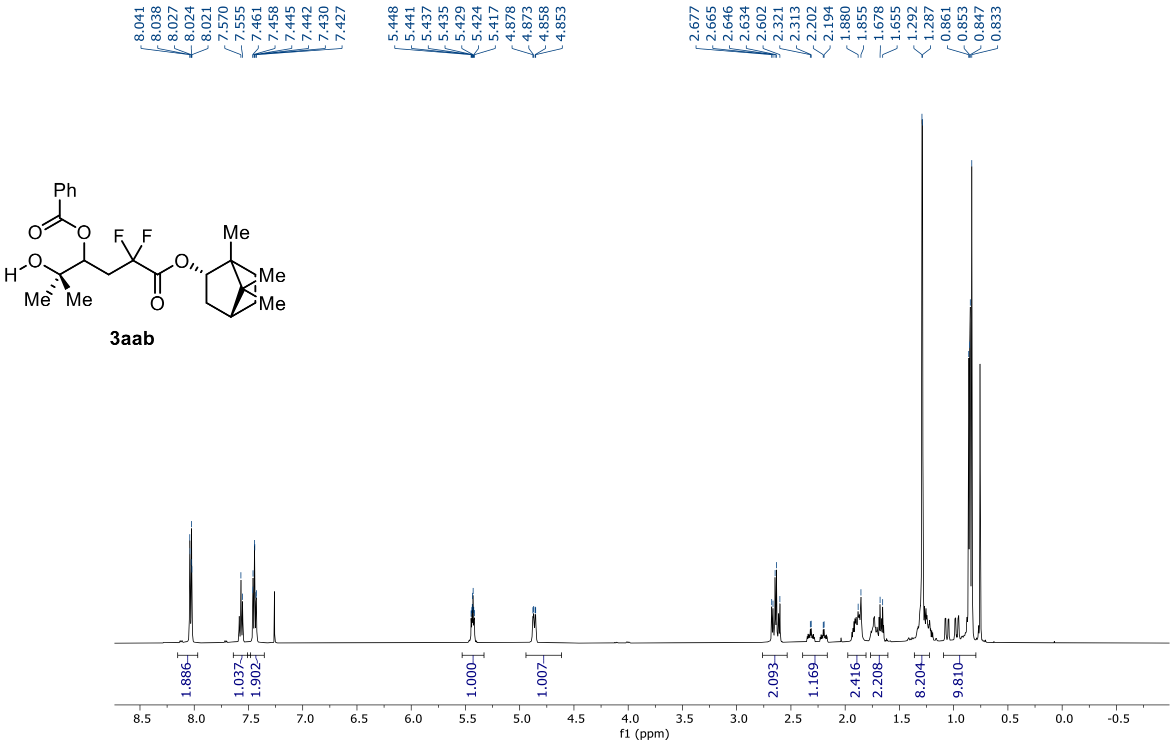

^{13}C NMR (126 MHz, $CDCl_3$, 25 ºC) of (3aab)

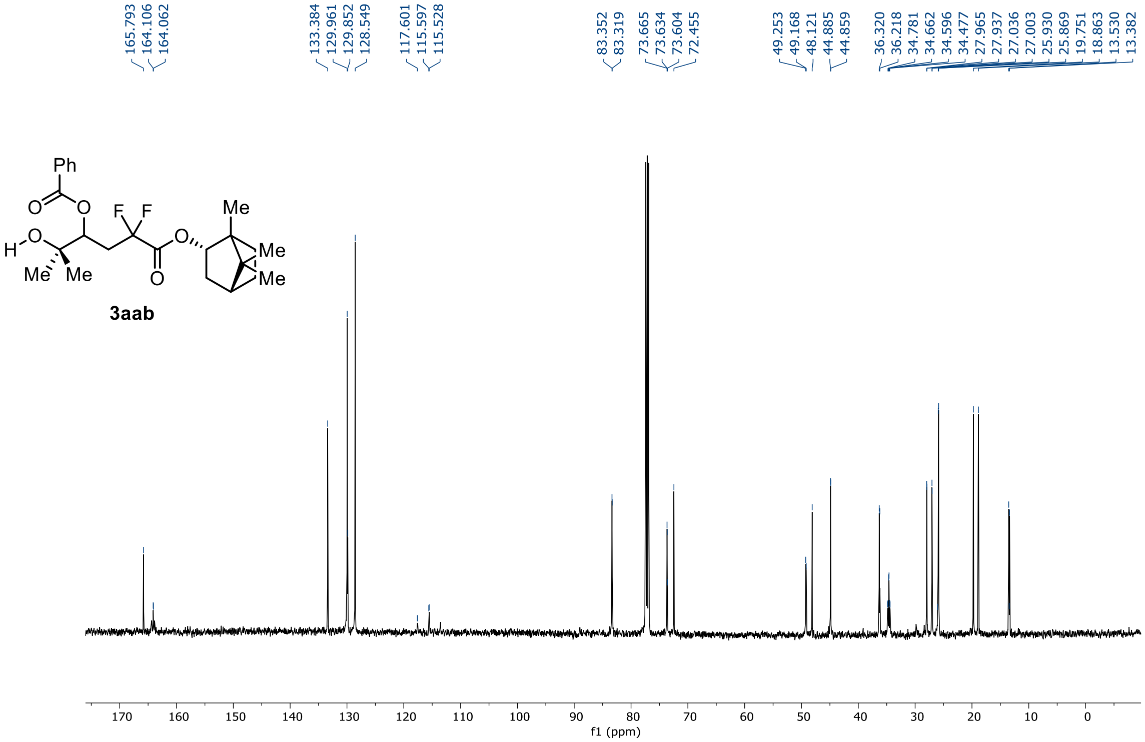

^{19}F NMR (470 MHz, $CDCl_3$**, 25 ºC) of (3aab)**

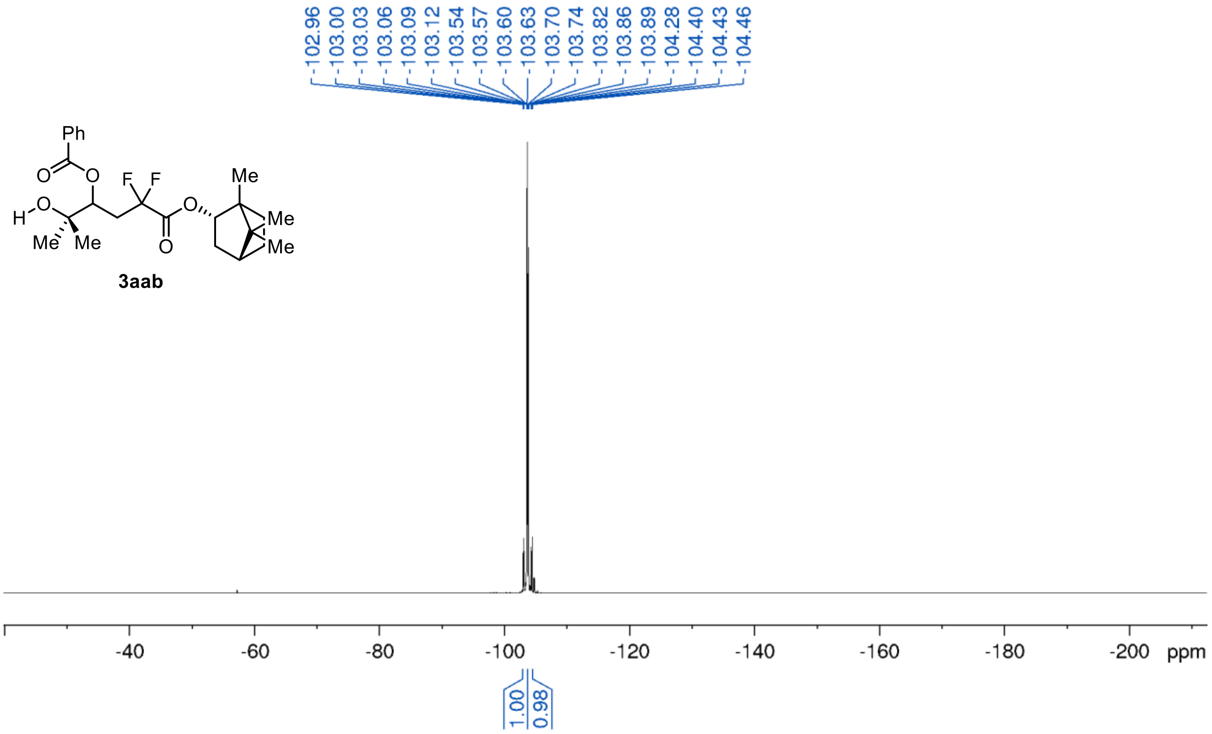

^{1}H NMR (500 MHz, CDCl$_3$, 25 ºC) of (3aac)

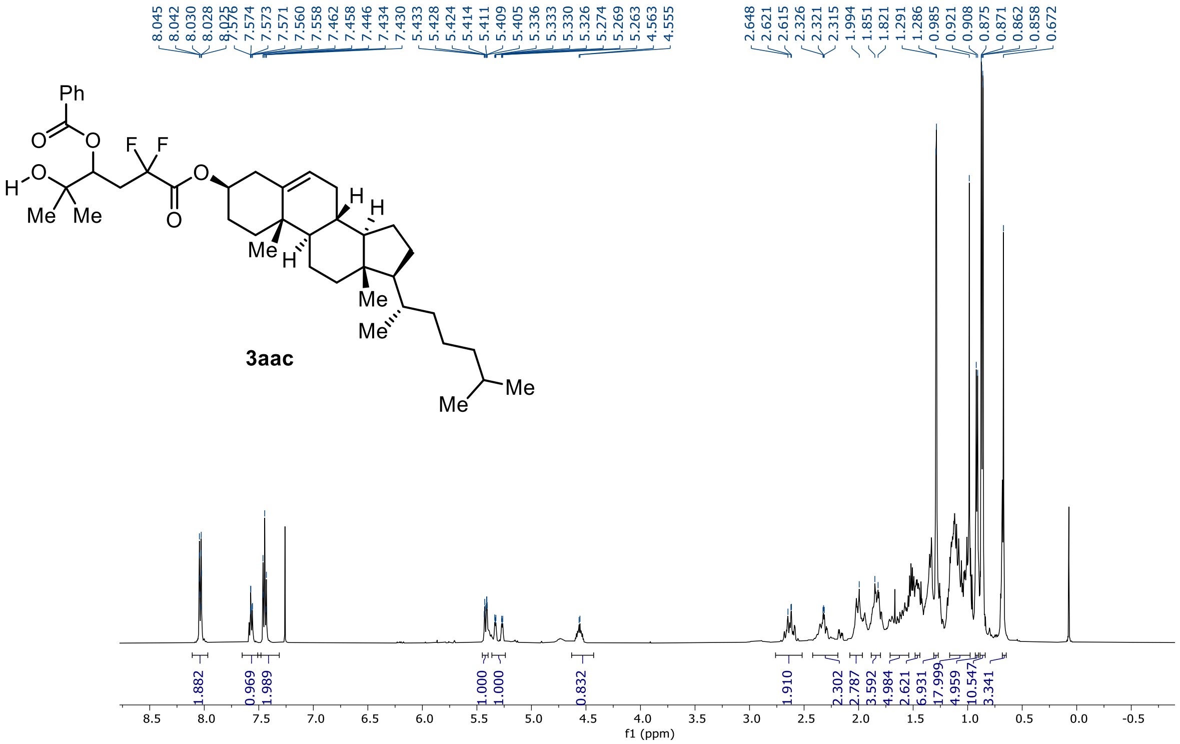

^{13}C NMR (126 MHz, CDCl$_3$, 25 ºC) of (3aac)

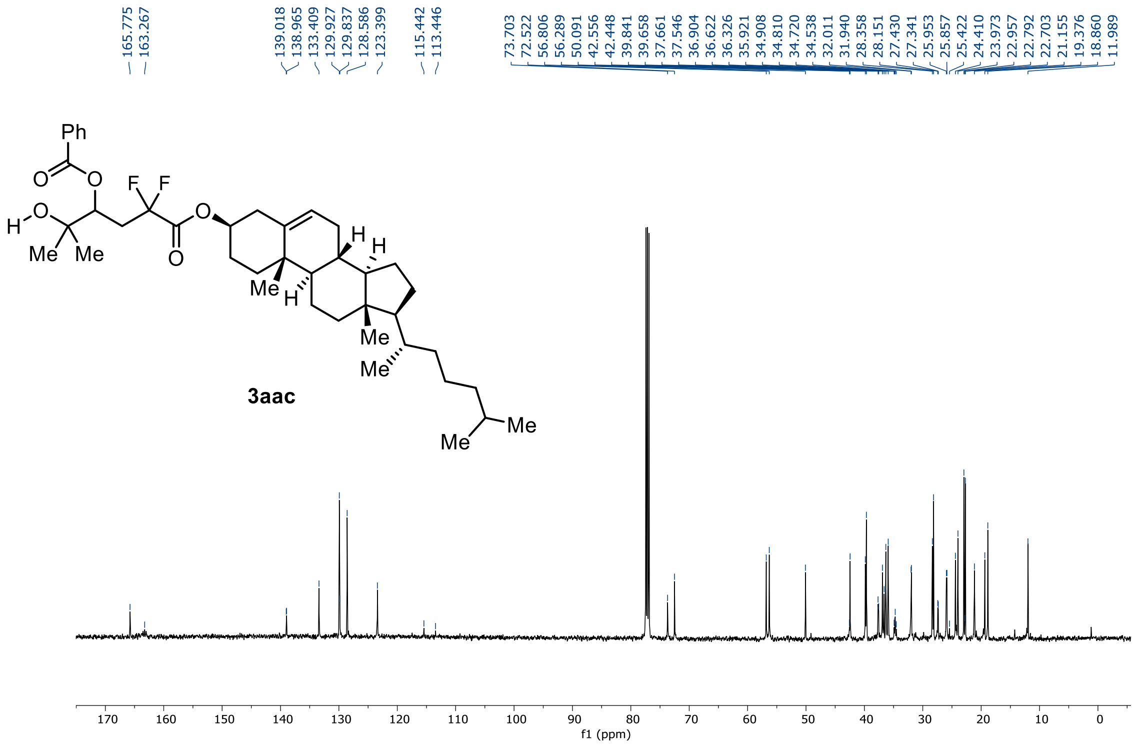

^{19}F NMR (470 MHz, CDCl$_3$**, 25 ºC) of (3aac)**

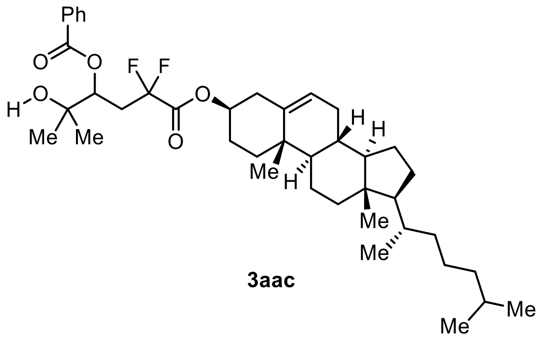

3aac

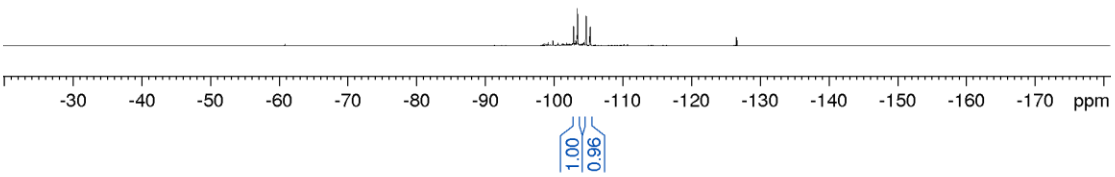

^{1}H NMR (500 MHz, CDCl$_3$, 25 ºC) of (3aad)

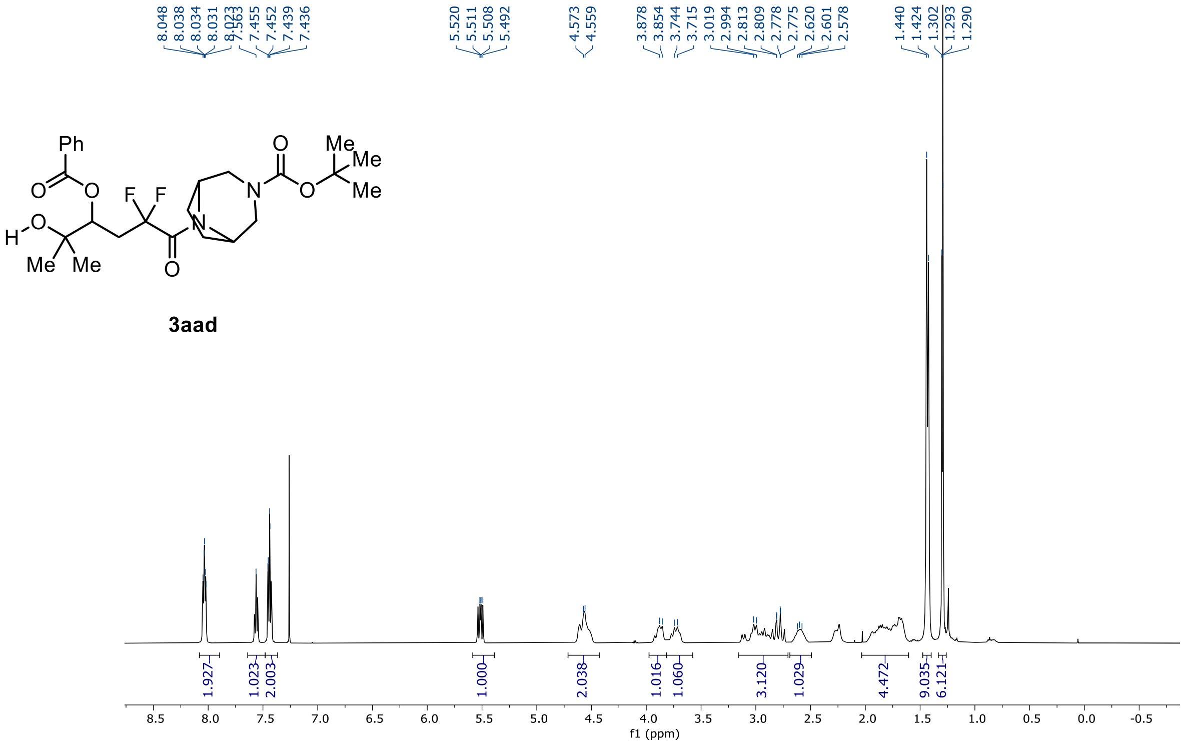

^{13}C NMR (126 MHz, CDCl$_3$, 25 ºC) of (3aad)

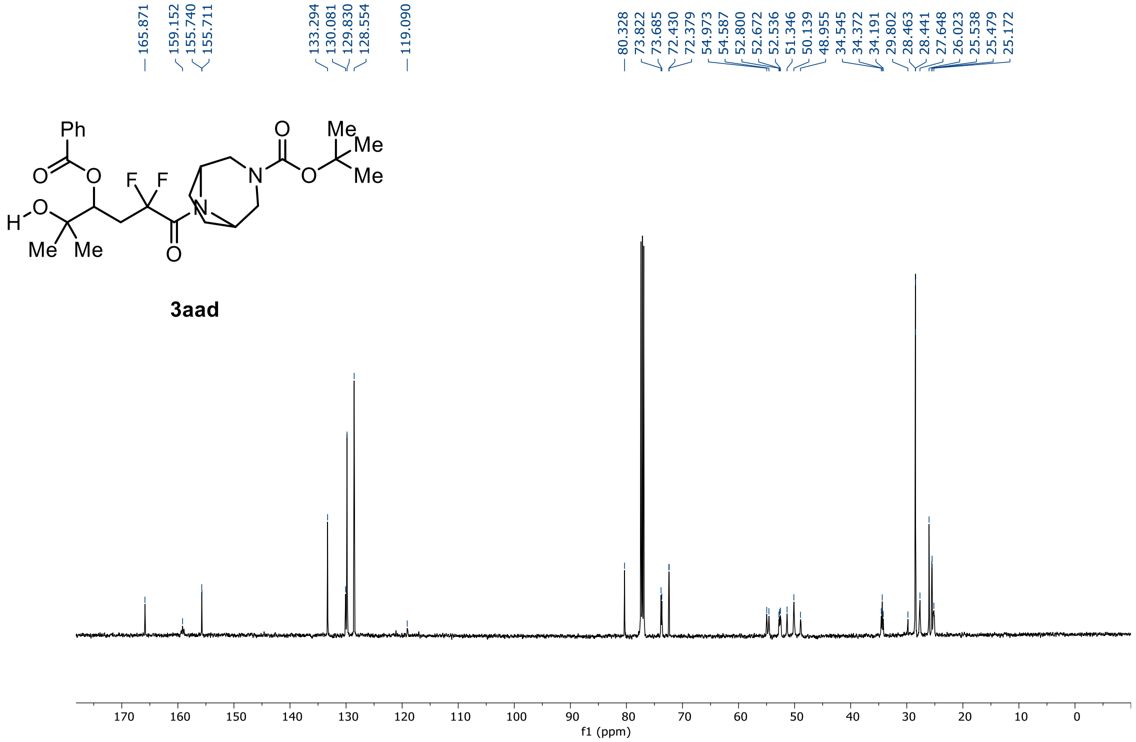

^{19}F NMR (470 MHz, CDCl$_3$**, 25 ºC) of (3aad)**

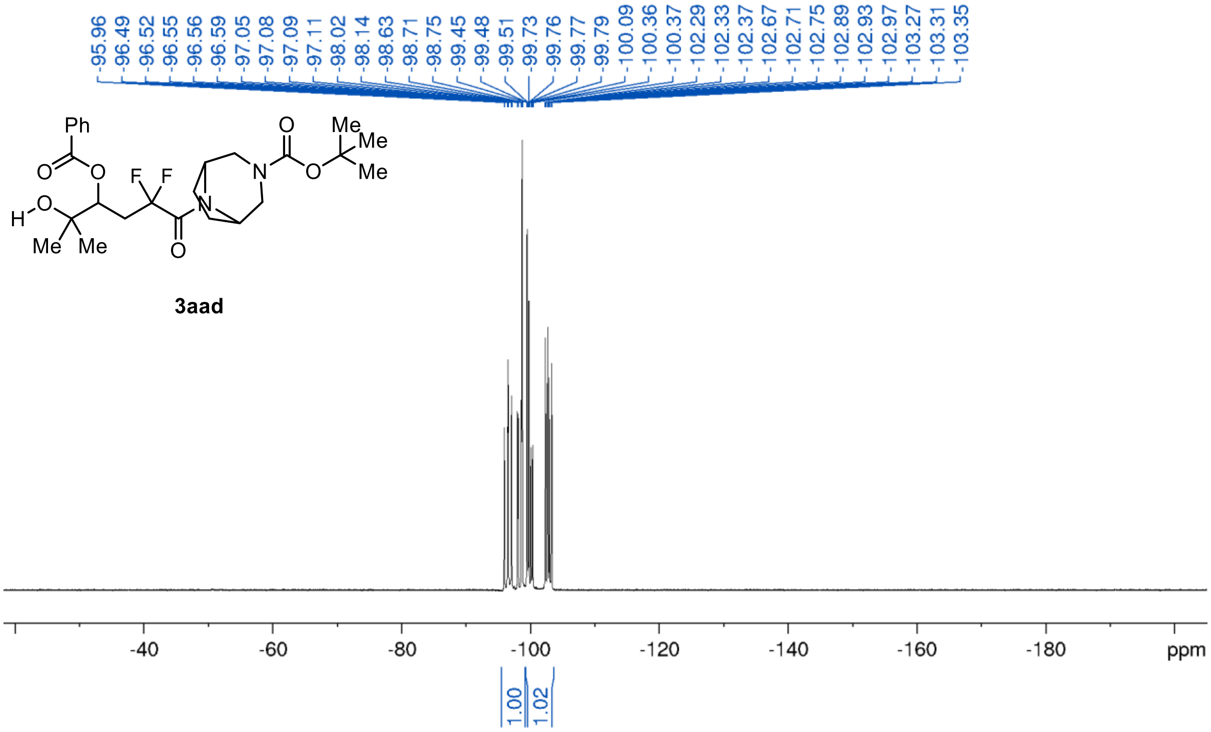

Cartesian Coordinates

Table S3: Calculated total (SCF) energies, enthalpies, and Gibbs free energies of intermediates and transition states.

	Level of theory: M06/6-311+G(d,p)/SMD(Dichloroethane)//B3LYP-D3BJ/SDD-6-31G(d)		
	SCF energy (a.u.)	Enthalpy (a.u.)	Free energy (a.u.)
**1a**	-615.891678	-615.642989	-615.715071
**II**	-505.418419	-505.316741	-505.374383
**3a**	-1197.242345	-1196.869201	-1196.97161
**2a**	-516.864446	-516.756858	-516.819083
**H_2O**	-76.425872	-76.400844	-76.427893
**III**	-588.268962	-588.243823	-588.298229
**VII**	-1121.224647	-1120.866828	-1120.961171
**V**	-1121.364816	-1121.011311	-1121.11078
**VI**	-1132.814992	-1132.448626	-1132.551809
**TS1**	-1121.312537	-1120.960817	-1121.060422
**TS2**	-1638.2236	-1637.753554	-1637.885104
**TS3**	-1132.777492	-1132.412407	-1132.51391
**Iodine-radical**	-11.362096	-11.359221	-11.383981
**VIII**	-1197.226314	-1196.853404	-1196.951164
**Na_2CO_3**	-588.480378	-588.4538	-588.503125
**Na_2HCO_3**	-588.953581	-588.914634	-588.965907
**2a+H_2O**	-593.293903	-593.158284	-593.231278
**2a+ Na_2CO_3**	-1105.355401	-1105.218772	-1105.307359
**Iodine-anion**	-11.566408	-11.563533	-11.587497
**NaI**	-173.775411	-173.770184	-173.806071
**TS4**	-1623.976735	-1623.570298	-1623.683031
**TS4-Comp-1**	-1274.062970	-1273.652389	-1273.756679
**3a’**	-1197.244101	-1196.870507	-1196.97091
**TS3'**	-1132.75285	-1132.388841	-1132.492867
**CF_3I+H_2O (gas phase)**	-425.421796	-425.375899	-425.41863
**CF_3I (gas phase)**	-348.997434	-348.977659	-349.013747
**CF_3-Radical (gas phase)**	-337.541041	-337.524473	-337.554558
**CH_3-Radical (gas phase)**	-39.810762	-39.776876	-39.799716
**Iodine-Radical (gas phase)**	-11.359466	-11.357106	-11.376969
**I-radical+H_2O (gas phase)**	-87.785712	-87.757501	-87.789861
**CH_3I (gas phase)**	-51.273514	-51.231213	-51.267627

**H_2O (gas phase)**	-76.41764	-76.392377	-76.419414
**TS5**	-1623.963743	-1623.55991	-1623.675205
**TS5'**	-1623.959968	-1623.55644	-1623.672127

Cartesian Coordinates

1a

C	-3.74774300	1.20907900	-0.93897000
H	-4.31922200	1.37154100	-0.02887600
H	-4.07383500	1.75489300	-1.82065800
C	-2.69726900	0.39153700	-0.98919000
H	-2.15917800	0.25356500	-1.92698300
C	-2.20494600	-0.49074100	0.13505600
O	-0.73591100	-0.59054500	0.06012900
C	0.02871100	0.51153100	0.14901000
O	-0.39989900	1.64256200	0.30256500
C	1.48063600	0.18870200	0.03642600
C	2.39787800	1.24464900	0.12946400
C	1.94340500	-1.12080100	-0.15713900
C	3.76459100	0.99514000	0.03044500
H	2.02419100	2.25204900	0.27887600
C	3.31205700	-1.36686200	-0.25623100
H	1.23306300	-1.93628500	-0.22904000
C	4.22334900	-0.31131700	-0.16257900
H	4.47180700	1.81624000	0.10348900
H	3.66820400	-2.38193000	-0.40630600
H	5.28929000	-0.50653800	-0.23986900
C	-2.64744200	-1.93428800	-0.12789200
H	-3.73850400	-1.99854500	-0.08190100
H	-2.32011000	-2.26653500	-1.11851500
H	-2.21996800	-2.60539300	0.62495600
C	-2.61871300	-0.04084200	1.53391700
H	-2.14154800	-0.68399200	2.28036100
H	-2.33210500	0.99538800	1.71893100
H	-3.70268700	-0.13531700	1.65007300

2a

C	0.20437000	1.01791500	0.38016700
C	-1.11874700	0.82528900	-0.38082200
O	-1.88222200	-0.05564600	0.24223600
C	-3.16013800	-0.36762700	-0.39746500
C	-3.83735300	-1.42242500	0.44660600
H	-4.80186500	-1.67702600	-0.00571200
H	-3.23009500	-2.33188000	0.50028300

H	-4.01795600	-1.05673400	1.46263300
H	-3.74129700	0.55646400	-0.45560700
H	-2.95090900	-0.71615200	-1.41242900
O	-1.35953200	1.41971800	-1.40524700
F	0.01057000	1.03057800	1.70833800
F	0.77399500	2.17213700	0.01676800
I	1.60540200	-0.65718100	-0.12456500

2a+H_2O

C	0.40082100	1.19381100	-0.26296200
C	1.56972500	0.58060600	0.52083200
O	2.20096800	-0.32058200	-0.21595200
C	3.29908800	-1.02931900	0.43647500
C	3.85428300	-2.01522700	-0.56571300
H	4.68241400	-2.56568100	-0.10638200
H	3.08892900	-2.73530200	-0.87327100
H	4.23268300	-1.50042300	-1.45489900
H	4.04159100	-0.28988800	0.74810200
H	2.89963300	-1.52161400	1.32755800
O	1.81585900	0.89674600	1.66224600
F	0.70449100	1.32866200	-1.57026400
F	0.12435800	2.41350000	0.23322600
I	-1.40642000	-0.09606600	-0.06098700
O	-3.79030400	-1.75854700	0.26845700
H	-4.49114500	-1.18978500	-0.09550200
H	-3.92917600	-1.70542400	1.23024700

2a+ Na_2CO_3

C	1.75500700	-1.22589800	0.53642600
C	2.96141500	-0.55858600	-0.12837400
O	3.17823800	0.64100600	0.39678900
C	4.25004100	1.42355100	-0.21027800
C	4.27576200	2.76511800	0.48628800
H	5.07414600	3.37901900	0.05562700
H	3.32543200	3.29369400	0.35458400
H	4.46956000	2.64826900	1.55762600
H	5.18621200	0.87289000	-0.08453900
H	4.03906100	1.51484500	-1.27949800
O	3.57436000	-1.07677600	-1.03433300
F	1.73256100	-0.97419000	1.86719800
F	1.83470400	-2.56190200	0.36572100
I	-0.14001300	-0.47956200	-0.37781200
C	-3.41855700	0.36256600	-0.47598300

O	-4.12065900	-0.69190500	-0.63839200
O	-3.65503500	1.15757300	0.55639700
O	-2.45296800	0.69027700	-1.28708300
Na	-1.90634700	2.37429000	0.04375500
Na	-5.17661700	-0.26783500	1.24065300

3a’

C	-0.94156900	0.78844200	1.09282500
H	-0.63597800	1.52986000	1.83661700
H	-0.26200100	-0.06461700	1.16024700
C	-0.94376300	1.37942000	-0.32389400
C	0.41211700	2.02702200	-0.71301700
O	1.57153200	1.24464100	-0.22616400
C	1.81037800	-0.01585400	-0.59428600
O	-1.41709200	0.42686800	-1.26355200
C	3.14092100	-0.49641100	-0.14067700
C	4.04949000	0.34710400	0.51567300
C	3.48053900	-1.83463800	-0.38645600
C	5.28683300	-0.14881700	0.92133400
H	3.78466500	1.38128200	0.70232700
C	4.71819300	-2.32551100	0.02104700
H	2.76708800	-2.47479300	-0.89384500
C	5.62220700	-1.48326200	0.67523800
H	5.99008700	0.50504100	1.42849800
H	4.97844700	-3.36245100	-0.16929300
H	6.58758100	-1.86671000	0.99321800
C	0.59099000	3.36061400	0.01089200
H	-0.18207800	4.06407300	-0.31183800
H	0.52499700	3.24404600	1.09635800
H	1.56976000	3.78634300	-0.22950000
C	0.54224100	2.21405700	-2.22227900
H	-0.27694900	2.84475900	-2.58180300
H	1.49015000	2.71142200	-2.45057200
H	0.49899500	1.26526600	-2.75771000
C	-2.31883200	0.29959000	1.49016200
C	-2.80014700	-0.92630700	0.69071700
O	-3.97046100	-0.72159200	0.10009300
C	-4.49247000	-1.81795300	-0.70512100
C	-3.86024800	-1.83329600	-2.08410900
H	-3.99428500	-0.86910900	-2.58397200
H	-2.79088900	-2.05159400	-2.02484200
H	-4.34117800	-2.61029800	-2.68953700
H	-4.32304300	-2.75474300	-0.16941400

H	-5.56432500	-1.61826500	-0.75394200
O	-2.15858700	-1.95588000	0.69287800
F	-2.29069600	-0.09673100	2.81067500
F	-3.22864900	1.32077600	1.41003500
O	1.02855400	-0.72602500	-1.22203300
H	-0.68657800	-0.21951400	-1.37074800
H	-1.67320800	2.19429600	-0.35471500

3a

C	2.20289600	0.79087100	0.40002000
H	3.28849000	0.90981400	0.34562400
H	1.88950400	0.92368800	1.43832300
C	1.83666700	-0.59885600	-0.10528500
C	2.67479800	-1.73392100	0.53860400
O	4.04979700	-1.47908500	0.21747200
C	-0.38102300	-1.26146600	-0.78595100
O	0.43399800	-0.79820900	0.19348200
C	-1.78559700	-1.38127100	-0.31768300
C	-2.75882900	-1.77519500	-1.24609300
C	-2.15386800	-1.10491200	1.00719600
C	-4.09045300	-1.88867900	-0.85467300
H	-2.45748300	-1.98317000	-2.26712000
C	-3.48747100	-1.22188200	1.39522300
H	-1.40030200	-0.79636400	1.72190700
C	-4.45601700	-1.61212400	0.46619700
H	-4.84316200	-2.19095500	-1.57667200
H	-3.77144600	-1.00751400	2.42130600
H	-5.49494300	-1.70048200	0.77103700
C	2.24986900	-3.09451300	-0.02115800
H	2.29428600	-3.09905400	-1.11625000
H	2.92549900	-3.86667300	0.36083800
H	1.22972500	-3.35408200	0.27803100
C	2.58629600	-1.72389100	2.06235100
H	1.54349200	-1.79021200	2.38674600
H	3.13262400	-2.58046300	2.46997600
H	3.02629200	-0.81274800	2.47876500
C	1.58734400	1.90410900	-0.42411800
C	0.04593300	1.97321400	-0.41010100
O	-0.38348400	2.26695400	0.81430900
C	-1.82486800	2.42070100	0.98623300
C	-2.25578700	3.83110300	0.63416300
H	-3.32716800	3.94298900	0.83595400
H	-1.71169300	4.56745300	1.23484400

H	-2.08237900	4.04043200	-0.42590600
H	-2.32849200	1.67327200	0.37232700
H	-1.98992300	2.19457000	2.04073700
O	-0.63780300	1.78555900	-1.39118200
F	2.05438400	3.11358200	0.03893900
F	1.98111700	1.79854600	-1.72925200
O	-0.00051400	-1.54146300	-1.90670100
H	4.16021200	-1.63777300	-0.73733800
H	1.96207000	-0.65166800	-1.18890500

TS4’

C	0.11130800	-1.93840800	0.96475300
H	-0.13162000	-2.88330100	1.46065300
H	-0.01980600	-1.12573900	1.68408200
C	-0.79568800	-1.76176400	-0.24701600
C	-2.29502200	-1.60013800	0.06949600
O	-2.34836400	-0.11997500	0.22203200
C	-1.37595700	0.46825700	-0.62515500
O	-0.47004100	-0.53960400	-0.95010300
C	-0.70928000	1.66086800	0.02724900
C	-1.00283400	2.02217000	1.34379900
C	0.23129500	2.39567200	-0.70701800
C	-0.36007000	3.11854200	1.92310100
H	-1.72074200	1.44714600	1.91758600
C	0.86761100	3.48991100	-0.12502000
H	0.49345500	2.10833400	-1.72236300
C	0.57318300	3.85346400	1.19189600
H	-0.58911800	3.39331600	2.94837400
H	1.59925800	4.05267500	-0.69688100
H	1.07348900	4.70367600	1.64577900
C	-2.78167700	-2.18130200	1.38306500
H	-2.22670100	-1.77865500	2.23300000
H	-3.84448600	-1.95609300	1.51715600
H	-2.66673200	-3.26925500	1.36701400
C	-3.16271200	-2.03134300	-1.10846700
H	-2.80064400	-1.61148800	-2.05013500
H	-3.13987200	-3.12306000	-1.18322900
H	-4.20298000	-1.72640400	-0.95541900
C	1.58118500	-2.01022200	0.59332300
C	2.21439200	-0.65096000	0.23931300
O	2.71050700	-0.62644100	-0.99018700
C	3.40468700	0.59443000	-1.38784900
C	4.85790500	0.54192800	-0.95790900

H	5.37687300	1.43453900	-1.32519700
H	4.94244400	0.52003100	0.13301100
H	5.35576400	-0.34179000	-1.37019000
H	3.30067000	0.61493000	-2.47394700
H	2.88259000	1.44703400	-0.95214800
O	2.24470500	0.24289300	1.05689300
F	2.28365900	-2.47409400	1.68152300
F	1.77195700	-2.91580800	-0.41490700
H	-0.63992800	-2.57771000	-0.95383100
H	-1.55734200	1.49945000	-2.31621900
H	-4.53701600	2.00077100	-0.08400600
H	-3.66504700	0.57703600	-0.00771800
O	-4.32352100	1.16980800	-0.57336100
O	-2.09328400	0.85999300	-1.80827600
H	-3.69302600	1.38363800	-1.33627200

H_2O(GAS Phase)

O	0.00000000	0.00000000	0.11942600
H	-0.00000000	0.76260200	-0.47770600
H	-0.00000000	-0.76260200	-0.47770600

H_2O

O	0.00000000	-0.00000000	0.12058200
H	0.00000000	0.76148400	-0.48233000
H	-0.00000000	-0.76148400	-0.48233000

II

C	1.51948000	-0.27313300	-0.10714300
C	0.31321100	0.51707700	-0.00993600
O	-0.77649400	-0.26768600	-0.00408300
C	-2.05069600	0.43011800	0.02624400
C	-3.13929500	-0.61976400	-0.01353800
H	-4.11747300	-0.12735900	0.01113700
H	-3.07587700	-1.21643000	-0.92962600
H	-3.06899900	-1.29133300	0.84862900
H	-2.09453100	1.03309100	0.93830700
H	-2.09756000	1.10471000	-0.83393600
O	0.32844900	1.74012900	0.01186500
F	1.54196900	-1.58255600	0.03989900
F	2.70054300	0.29277700	0.01893100

NaI

I	0.00000000	0.00000000	0.49755500
Na	0.00000000	0.00000000	-2.39731200

V

C	2.44825500	0.82122300	-0.85848900
H	3.42841500	1.22225000	-1.16417800
H	1.73002100	1.08060500	-1.64087500
C	2.03145000	1.34909800	0.47385000
H	2.50841200	0.95078400	1.36212200
C	0.98927300	2.40697800	0.62743100
O	-0.33232500	1.90483700	0.13825700
C	-0.85335400	0.78837100	0.66551700
O	-0.31504300	0.11640700	1.53332300
C	-2.18197800	0.45583900	0.07881200
C	-2.68626100	1.12353900	-1.04551100
C	-2.93144300	-0.56689600	0.67695900
C	-3.92661500	0.76261000	-1.56952600
H	-2.10366900	1.91277600	-1.50643000
C	-4.17289800	-0.92128700	0.15386700
H	-2.53147000	-1.06957400	1.55103400
C	-4.67080400	-0.25831800	-0.97177000
H	-4.31257300	1.27648600	-2.44509700
H	-4.75190500	-1.71215900	0.62171900
H	-5.63742200	-0.53664100	-1.38216100
C	1.23556700	3.60425300	-0.29548800
H	2.18414100	4.08319000	-0.03382700
H	1.27753500	3.29622500	-1.34418100
H	0.42999400	4.33739300	-0.18275200
C	0.84085600	2.86415900	2.07757600
H	0.00825800	3.56937600	2.16684100
H	0.66456700	2.01729700	2.74294900
H	1.75824300	3.37301200	2.39217500
C	2.59878900	-0.69105900	-0.86692900
C	1.25469900	-1.42871000	-0.72737300
O	1.23711700	-2.27051400	0.29269500
C	-0.01185300	-2.98556500	0.53276300
C	-0.06545500	-3.32545900	2.00487500
H	-0.07201100	-2.40978300	2.60320800
H	-0.98293800	-3.88747500	2.21287300
H	0.79008800	-3.94097600	2.30275000
H	-0.83958300	-2.34660600	0.22694400

H	0.00004600	-3.87737900	-0.10151000
O	0.35329300	-1.21714900	-1.51273300
F	3.13445800	-1.08848900	-2.07214600
F	3.47867000	-1.08930000	0.10177700

VI

C	0.37679300	0.47680800	0.93866100
H	1.12522500	0.39122100	1.72770700
H	-0.60434000	0.54770100	1.42026400
C	0.37051100	-0.72793500	-0.00401400
C	-0.61888100	-1.83801500	0.41778200
O	-1.93453200	-1.17503200	0.50686500
C	-2.52875700	-0.61283600	-0.56750800
O	-2.06422000	-0.60862500	-1.69418000
C	-3.82945000	0.01421300	-0.20702500
C	-4.55299700	0.65186300	-1.22464900
C	-4.33620500	-0.01016600	1.10052200
C	-5.77267000	1.25966800	-0.93840900
H	-4.14602800	0.66371900	-2.22999500
C	-5.55745600	0.59950600	1.38258600
H	-3.77571000	-0.50332500	1.88610000
C	-6.27611400	1.23419300	0.36565600
H	-6.33001300	1.75359800	-1.72882600
H	-5.94857300	0.58024000	2.39554700
H	-7.22715500	1.70933800	0.58917900
C	-0.39635200	-2.34016900	1.84057700
C	-0.69517600	-3.00425600	-0.56713000
H	-0.72823400	-2.66185600	-1.60189500
H	0.16890100	-3.65985400	-0.43622100
H	-1.59809300	-3.58606600	-0.35623600
C	0.55917400	1.80244500	0.22194800
C	1.90561700	2.00252200	-0.49923100
O	2.88915200	2.05329100	0.39293300
C	4.24145200	2.15486000	-0.14041600
C	5.19379800	2.04662600	1.02890700
H	6.22370700	2.12353300	0.66396200
H	5.02285700	2.85053400	1.75250600
H	5.07776500	1.08368700	1.53743600
H	4.38132200	1.34662600	-0.86306700
H	4.33054300	3.11154400	-0.66280700
O	2.01576800	2.08701700	-1.70112000
F	-0.43544100	1.96256100	-0.70206400
F	0.42350400	2.81607800	1.14126400

I	2.43918800	-1.54744700	-0.23639100
H	0.14230900	-0.43379200	-1.02379900
H	0.57291300	-2.83886200	1.91568900
H	-1.17920900	-3.05904700	2.10014300
H	-0.42679200	-1.52116600	2.56473200

VIII

C	2.09291000	-1.34156300	0.27819900
H	2.81628200	-2.14532700	0.10791400
H	1.85712300	-1.30417000	1.34172900
C	0.83635600	-1.57032600	-0.56543300
C	-0.08291500	-2.76770700	-0.15764900
O	-1.27807400	-2.10599100	0.34954600
C	-1.01434000	-0.74660100	0.50242500
O	-0.04085800	-0.43416800	-0.48350300
C	-2.26048100	0.07455300	0.22421300
C	-2.85181700	0.85011500	1.22228900
C	-2.82163600	0.04703700	-1.05831700
C	-3.99753800	1.60001000	0.94087300
H	-2.41636400	0.86057900	2.21527400
C	-3.96360100	0.79425300	-1.33878000
H	-2.35698100	-0.55690600	-1.83110200
C	-4.55406000	1.57401700	-0.33825400
H	-4.45310100	2.20154400	1.72248200
H	-4.39387300	0.77076600	-2.33613600
H	-5.44447200	2.15675000	-0.55763100
C	0.47605000	-3.68161400	0.92806100
H	0.68163700	-3.12721600	1.84635600
H	-0.25726300	-4.46174000	1.15693200
H	1.39717700	-4.17021800	0.59215200
C	-0.51049700	-3.56177200	-1.38857900
H	-0.90366000	-2.89191700	-2.16069700
H	0.34122300	-4.11153100	-1.80430000
H	-1.29079700	-4.28198200	-1.12186600
C	2.77967600	-0.03950100	-0.08556600
C	1.92815200	1.21269500	0.21358400
O	1.76397800	1.98307500	-0.84249400
C	0.89890200	3.15038100	-0.67810900
C	1.66408800	4.31759900	-0.08699200
H	1.00761500	5.19431000	-0.04998900
H	1.99960700	4.09449600	0.92978300
H	2.53456800	4.56480000	-0.70356500
H	0.55570500	3.35923900	-1.69224100

H	0.04768800	2.85847200	-0.05974200
O	1.49774000	1.42369800	1.33551600
F	3.92871400	0.09123300	0.66024600
F	3.16088800	-0.05105100	-1.39984500
H	1.12613500	-1.65298900	-1.61509200
O	-0.49399400	-0.54906600	1.79396800
H	0.03949600	0.27157700	1.78008500

VII

C	1.22149600	-1.97758000	0.49031600
H	1.66264600	-2.97406200	0.58575900
H	0.87727100	-1.65397400	1.47431000
C	0.07625000	-2.06566700	-0.50632300
C	-1.23570000	-2.72170100	-0.01566500
O	-1.99318300	-1.47966200	0.42636700
C	-1.47463400	-0.45205900	-0.15862700
O	-0.37847600	-0.68874400	-0.82119800
C	-2.05143200	0.85766000	-0.09826700
C	-3.25014600	1.05443100	0.61654400
C	-1.40625600	1.93237600	-0.74440800
C	-3.79358300	2.32947300	0.68613600
H	-3.73431500	0.21681800	1.10619900
C	-1.96509400	3.19996400	-0.66756400
H	-0.48991200	1.76612900	-1.29806100
C	-3.15253700	3.39765300	0.04754000
H	-4.71445800	2.49591900	1.23476700
H	-1.47992000	4.03524800	-1.16114500
H	-3.58264200	4.39283000	0.10616000
C	-1.13860300	-3.63699800	1.18470800
H	-0.69209100	-3.13934600	2.04784700
H	-2.13666600	-3.99115400	1.45625600
H	-0.52772000	-4.50535700	0.91971300
C	-2.03701800	-3.31231300	-1.16237400
H	-2.10469700	-2.61534700	-2.00388100
H	-1.53807200	-4.22396600	-1.50580500
H	-3.04483700	-3.56983600	-0.82625500
C	2.31883700	-1.02531300	0.04990700
C	1.97523500	0.46541800	0.26818900
O	2.59446500	1.24054200	-0.60698200
C	2.43190000	2.68566900	-0.43611900
C	3.39571500	3.21463600	0.60712100
H	3.30047500	4.30482200	0.66267000
H	3.17434900	2.79935600	1.59479300

H	4.42945800	2.97124200	0.34131100
H	2.64119200	3.08737100	-1.42829000
H	1.39150300	2.88353400	-0.17196100
O	1.25202100	0.82339900	1.17514200
F	3.43959100	-1.26421800	0.80676200
F	2.65824700	-1.25082300	-1.25466600
H	0.40269200	-2.48302000	-1.45655100

CF_3-Radical (Gas Phase)

C	0.00000000	-0.00000000	0.32922500
F	-0.00000000	1.26431500	-0.07316100
F	1.09492900	-0.63215700	-0.07316100
F	-1.09492900	-0.63215700	-0.07316100

CF_3I-H_2O (Gas Phase)

C	1.62746200	0.00206100	0.03763400
I	-0.57973800	-0.00313900	-0.05734800
F	2.14680600	0.25804400	-1.16485700
F	2.05484000	0.93659200	0.89133100
F	2.07922500	-1.18511000	0.45057200
O	-3.51145900	0.00338000	0.01225700
H	-3.73921600	-0.74573100	0.58441100
H	-3.73560800	0.78694900	0.53778100

CF_3I (Gas Phase)

C	1.22663600	-0.00006300	-0.00000800
I	-0.99300300	0.00001600	-0.00000800
F	1.67676000	0.62452700	-1.08346400
F	1.67664700	0.62619400	1.08253500
F	1.67652300	-1.25077000	0.00097900

CH_3-Radical (Gas Phase)

C	-0.00000000	-0.00000000	0.00001100
H	-0.00000000	1.08279400	-0.00002200
H	0.93772700	-0.54139700	-0.00002200
H	-0.93772700	-0.54139700	-0.00002200

CH_3I (Gas Phase)

C	0.00000000	0.00000000	-1.86052500

I	-0.00000000	-0.00000000	0.33408500
H	0.00000000	1.03957100	-2.18111800
H	0.90029500	-0.51978500	-2.18111800
H	-0.90029500	-0.51978500	-2.18111800

TS4

C	-2.31000000	-0.97630900	-1.04834700
H	-3.12794800	-1.63203400	-1.35994900
H	-1.67547900	-0.77942700	-1.91364200
C	-1.49847900	-1.64673200	0.04663400
C	-0.88959100	-3.05203400	-0.23488200
O	0.53557900	-2.82252800	0.16766600
C	0.76891100	-1.53784200	0.21924000
O	-0.32472000	-0.80133300	0.29074900
C	1.99287800	-1.00669000	0.83604400
C	3.19229700	-1.72477900	0.74887900
C	1.94265900	0.23032800	1.49441700
C	4.35026100	-1.19426500	1.31163400
H	3.21643100	-2.67776900	0.23095300
C	3.10422500	0.74537100	2.07309800
H	1.00924000	0.77821600	1.55581000
C	4.30950800	0.03902900	1.97170400
H	5.28372300	-1.74303300	1.24104800
H	3.06620300	1.68884400	2.61234500
H	5.21229500	0.44358900	2.41955700
C	-0.90358700	-3.51294900	-1.68298400
H	-0.45772300	-2.77881300	-2.35457500
H	-0.34186100	-4.44757900	-1.76680300
H	-1.93594600	-3.70559200	-1.99076000
C	-1.43826900	-4.10041600	0.71570700
H	-1.33157900	-3.77822100	1.75589500
H	-2.50156300	-4.25441000	0.50431400
H	-0.91660900	-5.05148400	0.57739700
C	-2.29539600	1.13541700	0.53830400
O	-2.57704500	0.57805900	1.69412900
C	-1.97890100	1.16749400	2.90179100
C	-2.81891600	2.32281500	3.40335300
H	-2.39128700	2.68827900	4.34352700
H	-2.82950600	3.14886200	2.68620500
H	-3.84765400	2.00123700	3.59396500
H	-1.96207100	0.33415200	3.60432900
H	-0.95700900	1.46501200	2.65939300
O	-1.61424700	2.12993500	0.36353700

F	-2.93141700	1.20850200	-1.71669600
F	-4.25976400	0.16588800	-0.29802800
H	-2.04843700	-1.66599700	0.98713200
C	1.55787200	1.57110300	-1.91500000
O	0.49413000	1.03358300	-2.43500600
O	1.49605500	2.77799400	-1.43501400
O	2.66762200	0.90276100	-1.82866300
Na	3.45172300	2.38633900	-0.40883800
O	1.31283500	-1.29276300	-1.71460300
H	0.72487200	-0.55510800	-2.08602900
H	2.13054500	-0.70696700	-1.70179000
C	-2.95018200	0.35572800	-0.62977400
Na	-0.75458000	2.69743000	-1.66271300

Na_2HCO_3

C	0.09641500	0.52158300	0.00450300
O	-0.01142800	-0.74655900	0.01456200
O	-1.12726800	1.16423300	0.00297100
O	1.14338300	1.19234600	-0.00444300
Na	2.27488300	-0.79185700	-0.00442400
Na	-2.24139800	-0.85719500	-0.00687800
H	-0.98431200	2.12991200	-0.00741700

Na_2CO_3

C	-0.00017000	0.56660700	0.00037300
O	1.12969700	1.18067300	0.00309300
O	0.00032400	-0.77059400	0.00124000
O	-1.12997800	1.17987700	-0.00326000
Na	-2.17927000	-0.73266400	0.00070600
Na	2.17933200	-0.73272500	-0.00169000

TS1

C	-3.01398700	0.98712100	-0.74687400
H	-3.56904500	0.56509300	-1.58011100
H	-3.60574600	1.34396100	0.09030300
C	-1.73313200	1.40229100	-0.94361300
H	-1.22434200	1.15192200	-1.86819600
C	-0.91374600	2.18365700	0.05208500
O	0.33571000	1.43954400	0.35047000
C	1.25027100	1.16334700	-0.58915300
O	1.14102400	1.42837200	-1.77742500

C	2.43976700	0.47106300	-0.01301300
C	2.52824800	0.14896000	1.34926200
C	3.48189900	0.11967700	-0.88174900
C	3.64976200	-0.52351200	1.83225600
H	1.71698600	0.41440000	2.01674300
C	4.60186700	-0.55049700	-0.39495400
H	3.39726000	0.37492900	-1.93241600
C	4.68616000	-0.87421600	0.96252300
H	3.71423500	-0.77605900	2.88654500
H	5.40685400	-0.82221300	-1.07148500
H	5.55797700	-1.39975300	1.34193000
C	-1.56276300	2.29291300	1.42862000
H	-2.48781400	2.87312900	1.36870900
H	-1.78755500	1.30252300	1.83363900
H	-0.88118700	2.80570300	2.11386700
C	-0.55779100	3.57125100	-0.49429300
H	0.14180300	4.07114100	0.18511500
H	-0.10708000	3.50906700	-1.48580200
H	-1.46930200	4.17430600	-0.55874100
C	-3.11626000	-1.06897100	0.23479100
C	-1.72512100	-1.48224300	0.53062500
O	-1.13671200	-1.95796400	-0.56903300
C	0.25932600	-2.35304600	-0.44655300
C	0.87582100	-2.29113500	-1.82661500
H	0.84648700	-1.27045700	-2.21966700
H	1.92280400	-2.60696500	-1.76704600
H	0.35249600	-2.95494600	-2.52305900
H	0.75244800	-1.68120400	0.25567300
H	0.27900200	-3.36606800	-0.03168100
O	-1.20506000	-1.30853600	1.61772300
F	-3.87714200	-0.85574000	1.31115200
F	-3.78262500	-1.77756900	-0.68318200

TS2

C	0.38209400	0.13723900	-1.22348000
H	-0.38308600	-0.14788500	-1.95354100
H	1.34812600	0.06119900	-1.73887900
C	0.37954000	-0.76576900	-0.01467600
H	0.43064400	-0.27703700	0.95061500
C	1.17066200	-2.05815800	-0.10364000
O	2.58286300	-1.68545900	-0.40623300
C	3.27110700	-0.85649500	0.39856100
O	2.82348400	-0.35536000	1.41888400

C	4.64852000	-0.60638300	-0.10506300
C	5.10802300	-1.13984300	-1.31791300
C	5.49468600	0.20086600	0.66840800
C	6.40347800	-0.86266200	-1.75075000
H	4.45194800	-1.76259400	-1.91485700
C	6.78863300	0.47396700	0.23282600
H	5.12653300	0.60237900	1.60627200
C	7.24397400	-0.05675500	-0.97779100
H	6.75740300	-1.27399300	-2.69147200
H	7.44162100	1.09978600	0.83397100
H	8.25286900	0.15794700	-1.31864700
C	0.80038400	-2.91517700	-1.31171800
H	-0.22431000	-3.28106700	-1.21163000
H	0.88014000	-2.34738900	-2.24321300
H	1.47547300	-3.77416700	-1.37141400
C	1.13247500	-2.87121300	1.18784400
H	1.85305100	-3.69263300	1.12465900
H	1.37035500	-2.25391600	2.05568600
H	0.13458800	-3.29856900	1.32207800
C	0.17674900	1.60790800	-0.89687300
C	1.33982200	2.19737000	-0.07402400
O	1.04820500	2.24260700	1.21818400
C	2.12344000	2.66129200	2.11291000
C	1.71867800	2.26467900	3.51401800
H	1.61365000	1.17790100	3.58889900
H	2.49317900	2.58742500	4.21857000
H	0.77249700	2.73456700	3.80250600
H	3.04032700	2.16524400	1.79397600
H	2.24216000	3.74321000	2.00228200
O	2.38791400	2.51305700	-0.59351700
F	0.10892600	2.31261400	-2.07228800
F	-1.01029100	1.81630200	-0.24647700
C	-4.56983300	-0.95546900	0.15469400
C	-4.68230900	0.55491700	0.23660800
O	-4.33459700	1.10490400	-0.91937700
C	-4.18382800	2.55677100	-0.92472900
C	-3.55234300	2.94141700	-2.24357800
H	-3.46915700	4.03277300	-2.29325400
H	-2.54874400	2.51718000	-2.33588000
H	-4.16409200	2.60466500	-3.08704900
H	-5.17447300	3.00078400	-0.79304600
H	-3.55819700	2.83398000	-0.07277500
O	-4.97734200	1.12728500	1.26312000
F	-4.97075300	-1.47780900	-1.00969200

F	-5.18864000	-1.57084800	1.16356500
I	-2.15138900	-1.35017800	0.33947000

TS3

C	0.85280300	0.60700600	0.99044100
H	1.76645900	0.78884800	1.55937400
H	0.12596900	0.18945600	1.69242400
C	1.13955300	-0.36348800	-0.11794900
C	0.97508800	-1.86385400	0.12559000
O	-0.47204900	-2.07526000	0.43397900
C	-1.29648500	-1.29455500	-0.24527300
O	-0.84414500	-0.33697500	-0.89348500
C	-2.72234600	-1.62444900	-0.15999400
C	-3.16942900	-2.72755400	0.58419600
C	-3.63800800	-0.80701600	-0.84130200
C	-4.53225200	-3.00289600	0.64868500
H	-2.45524200	-3.35559700	1.10429600
C	-4.99777000	-1.09096200	-0.77302900
H	-3.27336200	0.03601900	-1.41669100
C	-5.44447100	-2.18662100	-0.02719300
H	-4.88416200	-3.85319200	1.22435200
H	-5.70928400	-0.46150700	-1.29824000
H	-6.50683100	-2.40599000	0.02619800
C	1.66380300	-2.39666300	1.37875200
H	1.44395700	-1.77750200	2.25250600
H	1.29805600	-3.41008000	1.56754700
H	2.74211400	-2.42927000	1.22721700
C	1.28663700	-2.68749800	-1.11672600
H	0.71713900	-2.32920100	-1.97940000
H	2.35196700	-2.61249100	-1.34639500
H	1.03692400	-3.73594300	-0.93321200
C	0.34456800	1.96356600	0.51541700
C	-1.19151900	2.05681200	0.39666100
O	-1.56632900	2.76023300	-0.66031700
C	-2.99547700	3.03425800	-0.79070700
C	-3.37248900	4.26953200	0.00351200
H	-4.43231500	4.49488100	-0.16046300
H	-3.21712400	4.10877200	1.07482600
H	-2.78135800	5.13417400	-0.31531400
H	-3.13857500	3.17707400	-1.86276400
H	-3.54857300	2.15385600	-0.46066400
O	-1.91202400	1.57983100	1.24829000
F	0.67689400	2.90443700	1.46456800

F	0.95167900	2.33855000	-0.64565900
H	1.37284500	-0.00939400	-1.10493800
I	4.02598800	-0.14388900	-0.23915200

TS3'

C	1.27016700	1.47733500	0.04057400
H	2.13651800	1.85749900	-0.50393300
H	1.38539800	1.72821900	1.09443400
C	1.20376400	-0.00270600	-0.20084600
C	0.90063100	-1.03315100	0.79948300
O	-0.43696900	-0.48522400	0.13970700
C	-1.14105100	-1.21040300	-0.88071900
O	-0.64014000	-1.38205100	-1.95834300
C	-2.46981800	-1.61280900	-0.41513900
C	-2.87132900	-1.46326700	0.92325300
C	-3.34842100	-2.16650900	-1.36320000
C	-4.14551300	-1.87172800	1.30643000
H	-2.19760000	-1.02809600	1.65137000
C	-4.62184200	-2.56262700	-0.97187800
H	-3.02268400	-2.27378000	-2.39207300
C	-5.01987700	-2.41639100	0.36173300
H	-4.45861500	-1.76046800	2.33963000
H	-5.30491500	-2.98529500	-1.70176200
H	-6.01529000	-2.72770100	0.66441500
C	0.85845100	-0.69183300	2.25944100
H	0.38624600	0.27402900	2.44588100
H	0.32162100	-1.47052500	2.80747200
H	1.89145800	-0.65975600	2.62249300
C	1.16031700	-2.47435400	0.46738600
H	1.26121400	-2.64964700	-0.60451900
H	2.09664500	-2.75957400	0.95480000
H	0.35867500	-3.09643100	0.87774000
C	0.05515000	2.21249600	-0.51972000
C	-1.22109200	2.09168800	0.34025000
O	-2.29210500	1.83941200	-0.39782300
C	-3.58238900	1.82339800	0.29056200
C	-4.14026100	3.22864900	0.39622400
H	-5.13439700	3.18705000	0.85529800
H	-3.49982200	3.86016300	1.01971400
H	-4.23533900	3.68623300	-0.59379000
H	-4.20252100	1.17945800	-0.33342100
H	-3.44660600	1.36155200	1.26902100
O	-1.18344000	2.25688400	1.54115600

F	0.34882700	3.55144700	-0.55945600
F	-0.18350300	1.81070100	-1.80172900
H	1.37124200	-0.35215000	-1.21146100
I	4.30039200	-0.40432100	-0.03040100

TS5

C	-1.01421800	-1.42478100	1.89967100
H	-1.12763200	-1.40092300	2.98816000
H	-0.40885300	-2.29266600	1.62928100
C	-0.36878200	-0.13570200	1.42233800
C	1.04778300	0.19348600	1.96890400
O	1.55908600	1.05915100	0.94979800
C	0.50193900	0.87311800	-0.49484800
O	-0.19943100	-0.22287800	0.00545600
C	-0.24587600	2.18517300	-0.47884000
C	0.47633400	3.37277400	-0.64771500
C	-1.64032800	2.23206300	-0.38642000
C	-0.18704800	4.59598700	-0.70806800
H	1.55810400	3.32744100	-0.72419600
C	-2.30516700	3.46016600	-0.45344700
H	-2.20673600	1.31528900	-0.26621300
C	-1.58235000	4.64296400	-0.61090400
H	0.38137300	5.51371400	-0.83182000
H	-3.38914000	3.48803700	-0.38293800
H	-2.09992500	5.59706500	-0.65913300
C	1.92225800	-1.05547600	2.11754300
H	1.93862900	-1.63629700	1.19038400
H	2.94810800	-0.76581700	2.36382100
H	1.55311800	-1.69630100	2.92464600
C	0.95409100	0.96748300	3.28218500
H	0.39367900	1.89625900	3.13466700
H	0.45085300	0.37263100	4.05375700
H	1.95546100	1.22108700	3.64633000
C	-2.39818700	-1.62756400	1.30772300
C	-2.37419000	-1.87681900	-0.21337700
O	-2.97351000	-0.90670700	-0.89292900
C	-2.85537600	-0.95531300	-2.34514800
C	-3.92485500	-1.84167300	-2.95281800
H	-3.85507300	-1.79449200	-4.04556500
H	-3.79550800	-2.88272900	-2.64291100
H	-4.92371400	-1.50408100	-2.65671400
H	-2.96899500	0.08662100	-2.64872100

H	-1.84750400	-1.29537100	-2.59288700
O	-1.84267200	-2.86395100	-0.67305900
F	-2.97271300	-2.73710000	1.88112400
F	-3.20761900	-0.56374100	1.61019000
H	-1.02867700	0.70503000	1.65975700
O	1.23491600	0.65064500	-1.50344500
H	2.19465000	-0.34812200	-1.27295400
C	4.05070200	-0.45766900	-0.45674900
O	5.16105900	-1.01302300	-0.56057500
O	3.02141800	-1.01022600	-1.13889800
O	3.83261300	0.59485900	0.25718500
Na	4.40719600	-2.56283300	-1.98987500
H	2.73338600	0.80008700	0.55158400

TS5'

C	0.26756700	-2.06594200	1.23983400
H	0.38200600	-2.47657400	2.24845800
H	1.18763900	-2.25417100	0.68144400
C	-0.03451100	-0.56824100	1.29252500
C	0.78276700	0.24778200	2.31407400
O	0.61938100	1.61127300	1.84204300
C	0.39282800	1.80765500	0.47341100
O	0.18645900	0.04671600	0.03985100
C	-1.00264500	2.29436200	0.16838800
C	-2.03201700	2.23940100	1.11364600
C	-1.27487000	2.78258700	-1.11590900
C	-3.32194900	2.65360000	0.77391300
H	-1.81914800	1.88401300	2.11510100
C	-2.56110500	3.19792300	-1.45519400
H	-0.46800200	2.82646500	-1.83948500
C	-3.59082100	3.13085500	-0.51086000
H	-4.11498000	2.60814600	1.51532100
H	-2.76244600	3.57227500	-2.45521700
H	-4.59438600	3.45367500	-0.77402400
C	2.27075000	-0.10194200	2.34592200
H	2.71245800	-0.08867000	1.34876200
H	2.80245400	0.63072700	2.96168000
H	2.42833100	-1.09399100	2.78207900
C	0.17880700	0.17844300	3.71088200
H	-0.87554900	0.47182200	3.69665600
H	0.24873100	-0.84238100	4.10235600
H	0.71732200	0.84261200	4.39477600
C	-0.86311900	-2.84227200	0.58340400
C	-1.30882100	-2.25396300	-0.76897500

O	-2.32167500	-1.40945100	-0.60359300
C	-2.68688200	-0.58874700	-1.74966500
C	-3.64579600	-1.32188900	-2.66760400
H	-3.95903300	-0.64938900	-3.47457100
H	-3.17002900	-2.19959400	-3.11472300
H	-4.53942700	-1.64242600	-2.12126500
H	-3.14725200	0.29184900	-1.30125800
H	-1.76955500	-0.29388200	-2.26234800
O	-0.74624600	-2.52517900	-1.80597100
F	-0.46865200	-4.13676500	0.37180600
F	-1.93922400	-2.89133300	1.43716000
H	-1.09416900	-0.43470800	1.54353100
O	1.33367400	2.25050200	-0.24423800
H	2.51862100	1.48375700	-0.44033800
C	3.22210600	-0.23968200	-1.28411300
O	4.21861300	-0.75685100	-1.82205000
O	3.40137200	0.97794100	-0.72061600
O	2.06216600	-0.79775800	-1.23123200
Na	5.54501700	1.00164500	-1.42745100
H	1.20841300	-0.33188800	-0.60130000

TS4-Comp-1

C	0.11130800	-1.93840800	0.96475300
H	-0.13162000	-2.88330100	1.46065300
H	-0.01980600	-1.12573900	1.68408200
C	-0.79568800	-1.76176400	-0.24701600
C	-2.29502200	-1.60013800	0.06949600
O	-2.34836400	-0.11997500	0.22203200
C	-1.37595700	0.46825700	-0.62515500
O	-0.47004100	-0.53960400	-0.95010300
C	-0.70928000	1.66086800	0.02724900
C	-1.00283400	2.02217000	1.34379900
C	0.23129500	2.39567200	-0.70701800
C	-0.36007000	3.11854200	1.92310100
H	-1.72074200	1.44714600	1.91758600
C	0.86761100	3.48991100	-0.12502000
H	0.49345500	2.10833400	-1.72236300

C	0.57318300	3.85346400	1.19189600
H	-0.58911800	3.39331600	2.94837400
H	1.59925800	4.05267500	-0.69688100
H	1.07348900	4.70367600	1.64577900
C	-2.78167700	-2.18130200	1.38306500
H	-2.22670100	-1.77865500	2.23300000
H	-3.84448600	-1.95609300	1.51715600
H	-2.66673200	-3.26925500	1.36701400
C	-3.16271200	-2.03134300	-1.10846700
H	-2.80064400	-1.61148800	-2.05013500
H	-3.13987200	-3.12306000	-1.18322900
H	-4.20298000	-1.72640400	-0.95541900
C	1.58118500	-2.01022200	0.59332300
C	2.21439200	-0.65096000	0.23931300
O	2.71050700	-0.62644100	-0.99018700
C	3.40468700	0.59443000	-1.38784900
C	4.85790500	0.54192800	-0.95790900
H	5.37687300	1.43453900	-1.32519700
H	4.94244400	0.52003100	0.13301100
H	5.35576400	-0.34179000	-1.37019000
H	3.30067000	0.61493000	-2.47394700
H	2.88259000	1.44703400	-0.95214800
O	2.24470500	0.24289300	1.05689300
F	2.28365900	-2.47409400	1.68152300
F	1.77195700	-2.91580800	-0.41490700
H	-0.63992800	-2.57771000	-0.95383100
H	-1.55734200	1.49945000	-2.31621900
H	-4.53701600	2.00077100	-0.08400600
H	-3.66504700	0.57703600	-0.00771800
O	-4.32352100	1.16980800	-0.57336100
O	-2.09328400	0.85999300	-1.80827600
H	-3.69302600	1.38363800	-1.33627200

References

1. W. C. Still, M. Kahn and A. Mitra, *J. Org. Chem.*, 1978, **43**, 2923–2925.
2. G. R. Fulmer, A. J. M. Miller, N. H. Sherden, H. E. Gottlieb, A. Nudelman, B. M. Stoltz, J. E. Bercaw and K. I. Goldberg, *Organometallics*, 2010, **29**, 2176–2179.
3. a) S. Vaas, M. O. Zimmermann, D. Schollmeyer, J. Stahlecker, M. U. Engelhardt, J. Rheinganz, B. Drotleff, M. Olfert, M. Lämmerhofer and M. Kramer, *J. Med. Chem.*, 2023, **66**, 10202–10225; b) S. Wang, X. Luo, Y. Wang, Z. Liu, Y. Yu, X. Wang, D. Ren, P. Wang, Y.-H. Chen and X. Qi, *Nat. Chem.*, 2024, 1–9.
4. G. Zhao, S. Lim, D. G. Musaev and M.-Y. Ngai, *J. Am. Chem. Soc.*, 2023, **145**, 8275–8284.
5. G. Zhao, A. Khosravi, S. Sharma, D. G. Musaev and M.-Y. Ngai, *J. Am. Chem. Soc.*, 2024, **146**, 31391–31399.
6. A. Khosravi, Y. Zhang, G. Zhao, K. J. Radefeld, S. Sharma, N. A. Pannilawithana, Y. Zhang, P. Liu and M.-Y. Ngai, *J. Am. Chem. Soc.*, 2025, **147**, 27197–27206.
7. M. Mamone, G. Gentile, J. Dosso, M. Prato and G. Filippini, *Beilstein J. Org. Chem.*, 2023, **19**, 575–581.
8. a) S. Wang, J. Zhang, L. Kong, Z. Tan, Y. Bai and G. Zhu, *Org. Lett.*, 2018, **20**, 5631–5635; b) N. Zhou, Z. Xia, K. Kuang, Q. Xu, F. Zhao, L. Wang and M. Zhang, *Org. Lett.*, 2022, **24**, 5791–5796.
9. M. A. Cismesia and T. P. Yoon, *Chem. Sci.*, 2015, **6**, 5426–5434.
10. M. J. Frisch, G. W. Trucks, H. B. Schlegel, G. E. Scuseria, M. A. Robb, J. R. Cheeseman, G. Scalmani, V. Barone, G. A. Petersson, H. Nakatsuji, X. Li, M. Caricato, A. V. Marenich, J. Bloino, B. G. Janesko, R. Gomperts, B. Mennucci, H. P. Hratchian, J. V. Ortiz, A. F. Izmaylov, J. L. Sonnenberg, Williams, F. Ding, F. Lipparini, F. Egidi, J. Goings, B. Peng, A. Petrone, T. Henderson, D. Ranasinghe, V. G. Zakrzewski, J. Gao, N. Rega, G. Zheng, W. Liang, M. Hada, M. Ehara, K. Toyota, R. Fukuda, J. Hasegawa, M. Ishida, T. Nakajima, Y. Honda, O. Kitao, H. Nakai, T. Vreven, K. Throssell, J. A. Montgomery Jr., J. E. Peralta, F. Ogliaro, M. J. Bearpark, J. J. Heyd, E. N. Brothers, K. N. Kudin, V. N. Staroverov, T. A. Keith, R. Kobayashi, J. Normand, K. Raghavachari, A. P. Rendell, J. C. Burant, S. S. Iyengar, J. Tomasi, M. Cossi, J. M. Millam, M. Klene, C. Adamo, R. Cammi, J. W. Ochterski, R. L. Martin, K. Morokuma, O. Farkas, J. B. Foresman and D. J. Fox, *Journal*, 2016.
11. a) C. Lee, W. Yang and R. G. Parr, *Phy. Rev. B*, 1988, **37**, 785; b) A. Becke, *J. Chem. Phys*, 1993, **98**, 5648–5652.
12. S. Grimme, S. Ehrlich and L. Goerigk, *J. Comput. Chem.*, 2011, **32**, 1456–1465.
13. a) A. D. Becke and E. R. Johnson, *J. Chem. Phys.*, 2005, **123**, 154101; b) A. D. Becke and E. R. Johnson, *J. Chem. Phys.*, 2006, **124**; c) E. R. Johnson and A. D. Becke, *J. Chem. Phys.*, 2006, **124**, 174104.
14. L. E. Roy, P. J. Hay and R. L. Martin, *J. Chem. Theory Comput.*, 2008, **4**, 1029–1031.

15. Y. Zhao and D. G. Truhlar, *Theor. Chem. Acc.*, 2008, **120**, 215–241.

16. A. V. Marenich, C. J. Cramer and D. G. Truhlar, *J. Phys. Chem. B*, 2009, **113**, 6378–6396.

17. C. Legault, *Journal*, 2009.

18. P. Pracht, S. Grimme, C. Bannwarth, F. Bohle, S. Ehlert, G. Feldmann, J. Gorges, M. Müller, T. Neudecker, C. Plett, S. Spicher, P. Steinbach, P. A. Wesołowski and F. Zeller, *J. Chem. Phys.*, 2024, **160**, 114110.

19. G. Luchini, J. V. Alegre-Requena, I. Funes-Ardoiz and R. S. Paton, *F1000Research*, 2020, **9**, 291.
